# Stereoselective dearomative formal [3 + 2] cycloaddition of indole with allenols: access to structurally diverse cyclopenta[*b*]indoles

**DOI:** 10.1039/d6sc03994d

**Published:** 2026-06-29

**Authors:** Kavneet Kaur, Puja Singh, Rahul D. Thombare, Adithya K. P., Manoj V. Mane, Aslam C. Shaikh

**Affiliations:** a Department of Chemistry, Indian Institute of Technology Ropar Rupnagar Punjab 140001 India aslam.shaikh@iitrpr.ac.in; b Centre of Nano and Material Sciences, Jain (Deemed-to-be-University) Bangalore 562112 Karnataka India

## Abstract

Dearomative cycloaddition reactions of indoles offer a powerful strategy for rapidly increasing molecular complexity, yet intermolecular variants that proceed with high regio- and diastereocontrol remain uncommon. Herein, we report an iron(iii) chloride-mediated dearomative formal [3 + 2] cycloaddition of indoles with arylsulfonyl allenols, providing direct access to densely functionalized cyclopenta[*b*]indoles with excellent diastereoselectivity. The reaction proceeds under mild Lewis acidic conditions without the use of precious metal catalysts and displays broad substrate scope, tolerating diverse substitution patterns on the indole core and allenols, including electron-rich, electron-deficient, sterically hindered, and fused systems, affording the desired tricyclic scaffold in moderate to excellent yields. The synthetic utility of this protocol is demonstrated through late-stage functionalization of pharmaceutically relevant indole-containing molecules, double cascade dearomative cycloaddition, gram-scale synthesis, and downstream product diversification. Mechanistic studies, including DFT energy calculations and isotopic labeling experiments, support an ionic, non-radical pathway involving Lewis acid activation of the allenol to generate a transient electrophilic intermediate, followed by dearomative cyclization. This operationally simple and sustainable method enables rapid construction of structurally complex, nonplanar cyclopenta[*b*]indole frameworks of potential relevance in medicinal chemistry.

## Abstract 2

Dearomative formal [3 + 2] cycloaddition of indoles with arylsulfonyl allenols is reported for the direct synthesis of densely functionalized cyclopenta[*b*]indoles with excellent diastereoselectivity.

## Graphical abstract

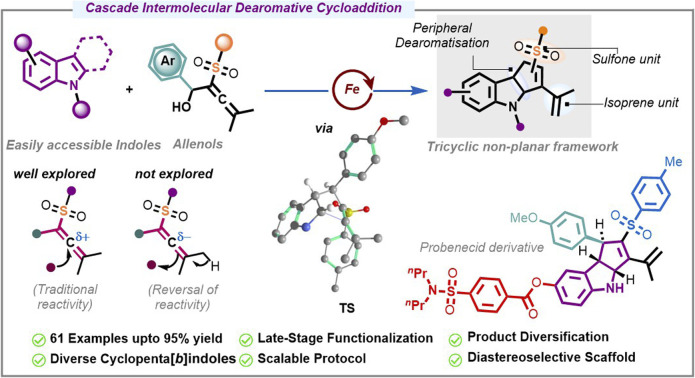

## Introduction

**Fig. 1:**
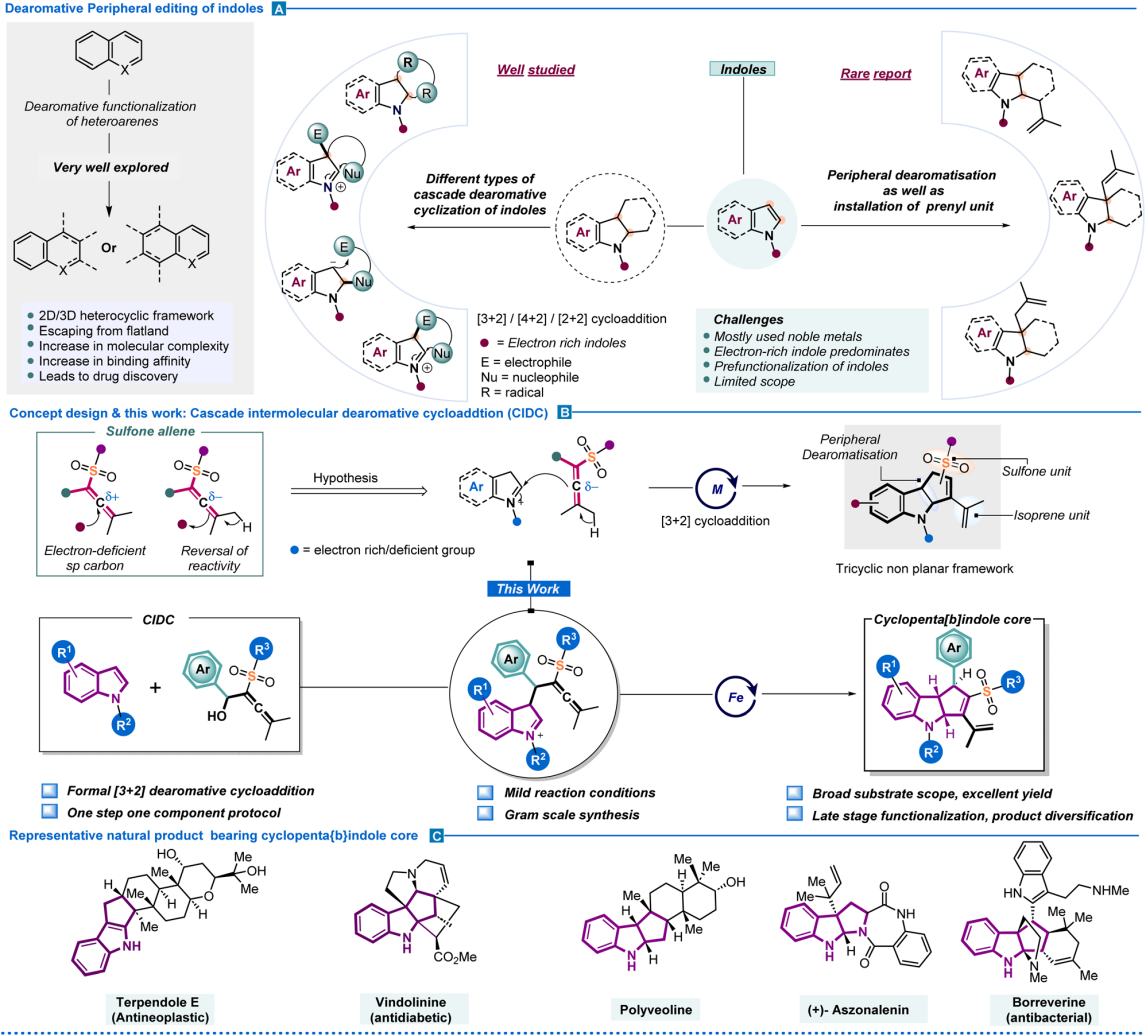
(A) Dearomative peripheral editing of indoles. (B) Concept design & this work: Cascade Intermolecular Dearomative Cycloaddition (CIDC). (C) Representative natural products bearing a cyclopenta[*b*]indole core.

Dearomative functionalization of heteroarenes^1–3^ represents a powerful strategy to “escape from flatland” by generating saturated or partially saturated frameworks. The enhanced sp^3^ character and conformational rigidity improve the potential for specific interactions with biological targets, thus increasing their significance in medicinal chemistry (Fig. 1A).^4^ In this context, Cascade Intermolecular Dearomative Cycloaddition (CIDC) reactions are widely employed to create polycyclic frameworks that are both complex and non-planar.^5,6^ Owing to their potent biological activities, widespread occurrence in natural products, and significant pharmaceutical relevance, indoline alkaloids have attracted sustained interest, making the dearomatization of indoles a longstanding and important topic in synthetic chemistry.^7–11^ In particular, cycloadditions such as formal [3 + 2],^12–15^ [2 + 2],^16–19^ [5 + 2],^20^ and [4 + 2]^21–23^ processes can transform the indole core into complex ring systems in a single step, which mostly rely on peripheral editing of indoles.^24,25^ However, controlling regioselectivity, enabling intermolecular reactivity, and avoiding competitive pathways remain challenging, especially when using electron-rich heteroarenes. Achieving these transformations generally requires either specialized activation modes or reagents capable of reversing typical reactivity trends.^26^ Consequently, the development of efficient, general, and stereoselective dearomative transformations of indoles remains a central objective in synthetic chemistry.

In contrast, prenyl units are privileged structural motifs widely embedded in natural products and biologically active small molecules, where they play a decisive role in enhancing molecular complexity, conformational rigidity, and functional diversity.^27,28^ In complex molecular frameworks, prenyl substituents frequently act as latent synthetic handles, enabling further structural elaboration through cyclization, oxidation, rearrangement, or skeletal reorganization, thereby facilitating rapid access to higher-order architectures.^29,30^ Moreover, prenylation is often correlated with increased lipophilicity, membrane affinity, and enhanced biological activity, particularly in indole-derived and heteroaromatic systems.^31,32^ Despite these advantages, strategies for the direct incorporation of prenyl units during dearomative cycloaddition processes, especially under intermolecular conditions, are limited (Fig. 1A).^33–35^ This emphasizes the synthetic potential of combining dearomatization of indoles along with the installation of a prenyl unit in one step.

Building on our continued efforts and interest in advancing sulfonyl allene reactivity,^36,37^ where allenes are useful synthetic precursors in organic chemistry because they facilitate a variety of synthetic transformations,^38,39^ we realize that the strong electron-withdrawing sulfonyl group polarizes the cumulene framework, rendering sulfonyl allenes highly reactive toward nucleophilic attack and cycloaddition processes and therefore acting as an electrophile (Fig. 1B).^40,41^ Moreover, activating cumulated double bonds with Brønsted or Lewis acids allows for nucleophilic attacks, leading to the formation of new C–C or C–heteroatom bonds, either intermolecularly or intramolecularly.^42,43^ We hypothesized that sulfone-substituted allenes could serve as effective coupling partners due to their ability to modulate the intrinsic polarity of the allene framework. Herein, we developed an iron-mediated dearomative formal [3 + 2] cycloaddition between indoles and sulfone allenols. This intermolecular cascade process induces peripheral dearomatization of the indole nucleus and provides direct access to structurally diverse, nonplanar tricyclic cyclopenta[*b*]indole frameworks. Notably, the cyclopenta[*b*]indole core is present in a range of natural products that exhibit diverse biological activities (Fig. 1C).^44–46^ Furthermore, the resulting formal [3 + 2] cycloadduct incorporates both sulfone and isoprene structural motifs, leading to a pronounced increase in molecular complexity in a single step. The transformation proceeds under mild conditions, displays broad substrate compatibility, high stereoselectivity, and can be readily performed on a gram scale along with late-stage functionalization and product diversification. Moreover, stereoselectivity and the possible transition state for the formation of the desired product were predicted through DFT studies.

## Results and discussion

### Reaction development

To evaluate the feasibility of the proposed formal [3 + 2] cycloaddition reaction, we chose N–H indole (1a) and sulfonyl allenol (2a) as model substrates. The details of our findings are summarized in Table 1 and the SI. Gratifyingly, after extensive screening of various reaction parameters, we discovered suitable conditions for the formal [3 + 2] cycloaddition reaction to afford tricyclic non-planar scaffold 3a. In the presence of 20 mol% FeCl_3_, a mixture of 1a (1.3 equiv.) and 2a (1.0 equiv.) in dichloroethane (DCE) at 80 °C for 18 h smoothly undergoes cycloaddition for the formation of 3a in excellent yield with more than 99 : 1 diastereoselectivity (88%, Table 1, entry 1). Attempts to further optimize the reaction included screening a varied panel of Lewis acids and Brønsted acids. A drop in the yield of 3a was noted, whereas only *p*-TsOH·H_2_O afforded a comparable yield of 3a in 72% (entries 2–6). In search of an alternative iron catalyst, we examined Fe(acac)_3_ and Fe(OTf)_3_, but this resulted in reduced yield of cycloaddition adduct 3a (entries 7 and 8). After screening several reaction media, it was revealed that DCE remained the most ideal solvent for the formal [3 + 2] cycloaddition reaction (entries 9–12). Furthermore, to see the impact of the inclusion of acid and base additives to standard reaction conditions, we tested AcOH and K_2_CO_3_; however, a diminished formation of the desired product 3a was noted (entries 13 and 14). Lowering the reaction temperature leads to a reduction in the yield of the desired product 3a (entries 15 and 16). Following this, the influence of reaction time was analyzed, showing that maximum transformation occurs within 18 hours (entry 17). Finally, reducing the FeCl_3_ concentration by half from 20 mol% to 10 mol% resulted in a significant drop in the yield of 3a to 72%, whereas no conversion occurred in the absence of FeCl_3_, indicating the critical role of FeCl_3_ in achieving optimal yield (entries 18 and 19).

**Table 1:** Optimization of reaction conditions^a^^,^^b^

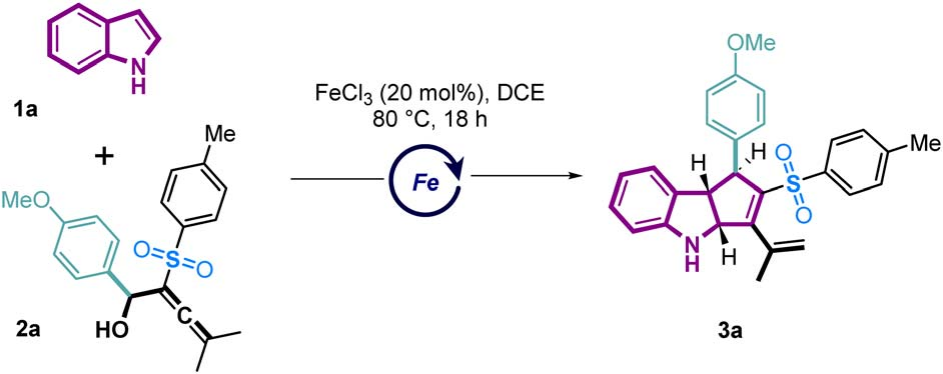			
Entry	Deviation from standard conditions	Yield of 3a^b^	d.r.
1	None	88^a^	>99 : 1
2	*p*-TsOH.H_2_O instead of FeCl_3_	72	>99 : 1
3	TfOH instead of FeCl_3_	<5	—
4	Sc(OTf)_3_ instead of FeCl_3_	58	>99 : 1
5	ZnCl_2_ instead of FeCl_3_	50	>99 : 1
6	AlCl_3_ instead of FeCl_3_	n.d.	—
7	Fe(acac)_3_ instead of FeCl_3_	65	>99 : 1
8	Fe(OTf)_3_ instead of FeCl_3_	45	>99 : 1
9	DMSO instead of 1,2-DCE	Traces	—
10	CH_3_CN instead of 1,2-DCE	50	>99 : 1
11	THF instead of 1,2-DCE	<5	—
12	Toluene instead of 1,2-DCE	70	>99 : 1
13	AcOH as an additive	55	>99 : 1
14	K_2_CO_3_ as an additive	20	>99 : 1
15	25 °C instead of 80 °C	n.d.	—
16	50 °C instead of 80 °C	55	>99 : 1
17	12 h instead of 18 h	60	>99 : 1
18	10 mol% of FeCl_3_ employed	72	>99 : 1
19	Without FeCl_3_	n.d.	—

a Reaction conditions: 1a (0.071 mmol, 1.3 equiv.), 2a (0.055 mmol, 1.0 equiv.), 20 mol% FeCl_3_, DCE (0.5 ml), 80 °C, 18 h.

b NMR yields are given with 2,4,6-trimethoxy benzene as the internal standard. n.d. = not detected. d.r. = diastereomeric ratio. A single diastereomer was observed in all cases.

**Scheme 1:**
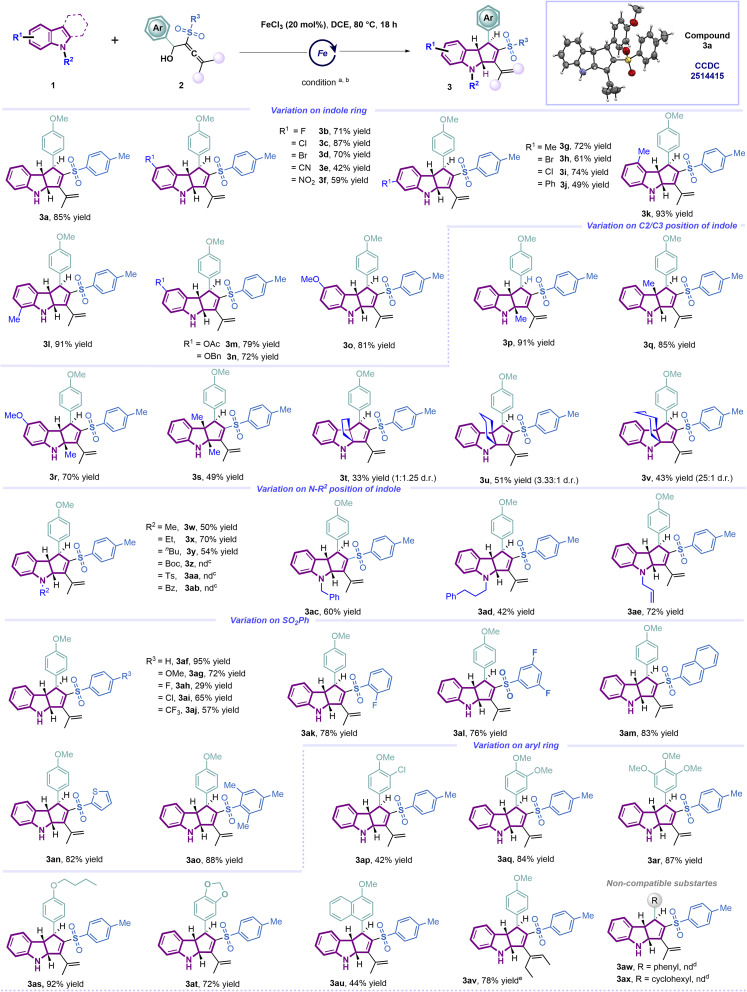
Substrate scope. ^*a*^Indole 1 (1.3 equiv.), aryl sulfonyl allenol 2 (1.0 equiv.), 20 mol% FeCl_3_, DCE (0.1 M, 3.0 ml), 80 °C, 18 h. ^*b*^Isolated yields are given. ^*c*^Indole fully recovered. ^*d*^Starting material recovered completely. ^*e*^Reaction performed for 24 h. Single diastereomer observed and isolated in most cases.

The reaction's generality was demonstrated through a broad substrate scope evaluation that included variations of the indole ring and the sulfonyl allenol fragment of template substrates (Scheme 1). The applicability of this method was initially assessed using various indole derivatives. To our surprise, variations on the indole ring with electron-donating (–Me, –OMe and –OBn), weakly electron-donating (–OAc), and halo substituents (F, –Cl and –Br) at different positions were well tolerated to afford the desired [3 + 2] cycloaddition product in good to excellent yields (3a–3o), up to 93% yield. It is worth noting that the reaction was compatible with halogen atoms on the indole ring, opening the door for further functionalization of the tricyclic scaffold. However, as expected, strong electron-withdrawing groups (–CN and –NO_2_) on indole offered moderate conversion with 42% and 59% yield, respectively. In the next stage, we achieved a significant transformation of C2 and C3-substituted indoles (2p, 2q, and 2r). This variation not only resulted in impressive yields but also markedly increased three-dimensional complexity, showcasing the remarkable potential of our approach (3p–3r, 70–91% yields). Moreover, sterically hindered fused indoles (2s, 2t, 2u, and 2v) were converted to the target dearomative cyclopenta[*b*]indole derivatives, albeit in moderate yield (up to 51%). However, the desired product formed non-separable diastereomers for 3t and 3u in ratios of 1.25 : 1 and 3.33 : 1, respectively. This strategy has demonstrated noteworthy effectiveness with several *N*-substituted indoles, specifically compounds 3w, 3x, 3y, 3ac, and 3ad, which were obtained in moderate to good yields (42–70%). However, it falls short when applied to bulkier *N*-protected electron-withdrawing groups such as boc (3z), tosyl (3aa), and benzoyl (3ab). This distinction highlights the strategy's potential while also pointing out the challenges it faces with electron-deficient substituents. Also, reactive functionality such as an allyl group on *N*-substitution works well to furnish the desired product 3ae with a yield of 72%.

Next, we focused our attention on the scope of the sulfonyl moiety, which revealed that *ortho*, *meta*, and *para*-substituted aryl sulfonyl groups with various functional groups such as –Me, –OMe, –F, –Cl, and –CF_3_ substituents were well tolerated to deliver the desired product with appreciable yields (3af–3al, up to 95%), increasing the synthetic utility of this method. Along with this, naphthyl, heteroaryl, and the sterically crowded mesityl group demonstrated effective participation in the established reaction protocol (3am–3ao, 82–88% yield). This finding highlights their potential significance within the framework of our research. In subsequent investigations, we evaluated the effect of substituents on the aryl units attached to aryl sulfonyl allenols (2). Substrates with strong electron-donating groups, either monomethoxy, dimethoxy, or trimethoxy substituents, only underwent dearoamative formal [3 + 2] cycloaddition with indoles, yielding the desired products 3ap–3au in commendable yields of up to 92%. Replacing the dimethyl unit on allenols with a diethyl unit lengthens the reaction time for smooth conversion, delivering the corresponding product 3av in a yield of 78%. Furthermore, phenyl- and cyclohexyl-substituted allenols didn't lead to the formation of the desired product (3aw–3ax).

**Scheme 2:**
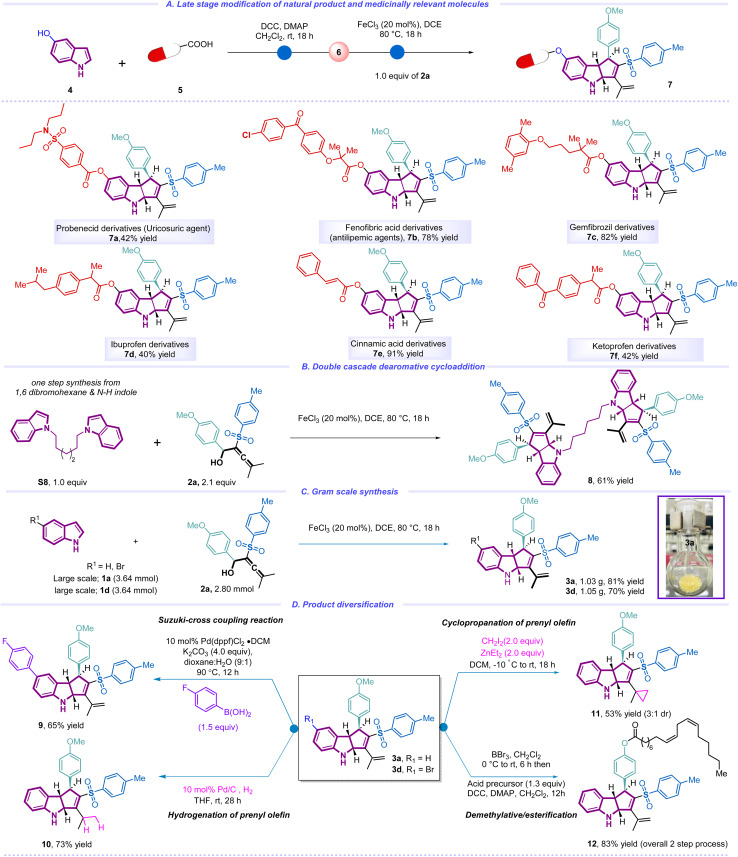
A) Late-stage modification of natural products and medicinally relevant molecules. (B) Double cascade dearomative formal [3 + 2] cycloaddition. (C) Gram scale reaction. (D) Product diversification.

The FeCl_3_-mediated formal [3 + 2] cycloaddition protocol showcases its significant potential for the late-stage functionalization of complex, pharmaceutically relevant molecules (Scheme 2A).^47,48^ By efficiently targeting a diverse array of drug-derived indoles, including probenecid (6a), fenofibric acid (6b), gemfibrozil (6c), ibuprofen (6d), cinnamic acid (6e), and ketoprofen derivatives (6f), this innovative approach facilitates the generation of valuable dearomative cyclopenta[*b*]indole derivatives 7a–7f in moderate to excellent yields (40–91%). More interestingly, the substrate holding two indole units (S8) undergoes effective double cascade dearomative cycloaddition to furnish the corresponding product 8 in 61% yield (Scheme 2B).^49^ This result highlights the strategic application of this protocol, which not only enhances synthetic utility but also contributes to the advancement of drug development in a highly effective manner through the rapid generation of molecular complexity in a single step. Additionally, we were able to scale up the formal [3 + 2] cycloaddition reaction (2a; 2.8 mmol scale), delivering the tricyclic cyclopenta[*b*]indole adducts 3a and 3d in 81% and 70% yield, respectively (Scheme 2C). This indicates the robustness, scalability, and operational practicality of this methodology. To effectively demonstrate the synthetic utility of our method, we undertook product diversification of the cyclopenta[*b*]indole derivative (3a and 3d) through cross-coupling reactions and modification of the isoprenyl unit (Scheme 2D). The bromo group of 3d successfully engaged for Suzuki coupling with 4-fluorophenylboronic acid, leading to the corresponding cross-coupled product 9 in an acceptable yield of 65%. Alternatively, the isoprenyl unit of substrate 3a was hydrogenated under Pd/C conditions to give the corresponding analogue 10 in 73% yield. Also, the Simmons–Smith cyclopropanation reaction^50^ was successfully carried out on 3a to generate the strained cyclopropyl-embedded product 11 in 53% yield with a 3 : 1 d.r. Furthermore, the methoxy group bearing an electron-rich aryl fragment of cyclopenta[*b*]indole derivative (3a) has been demethylated in the presence of BBr_3_ and transformed into another ester analog 12 by using linoleic acid as a coupling partner. Collectively, these results highlight the wide applicability, functional-group tolerance, and robustness of the developed transformation.

### Mechanistic studies

**Fig. 2:**
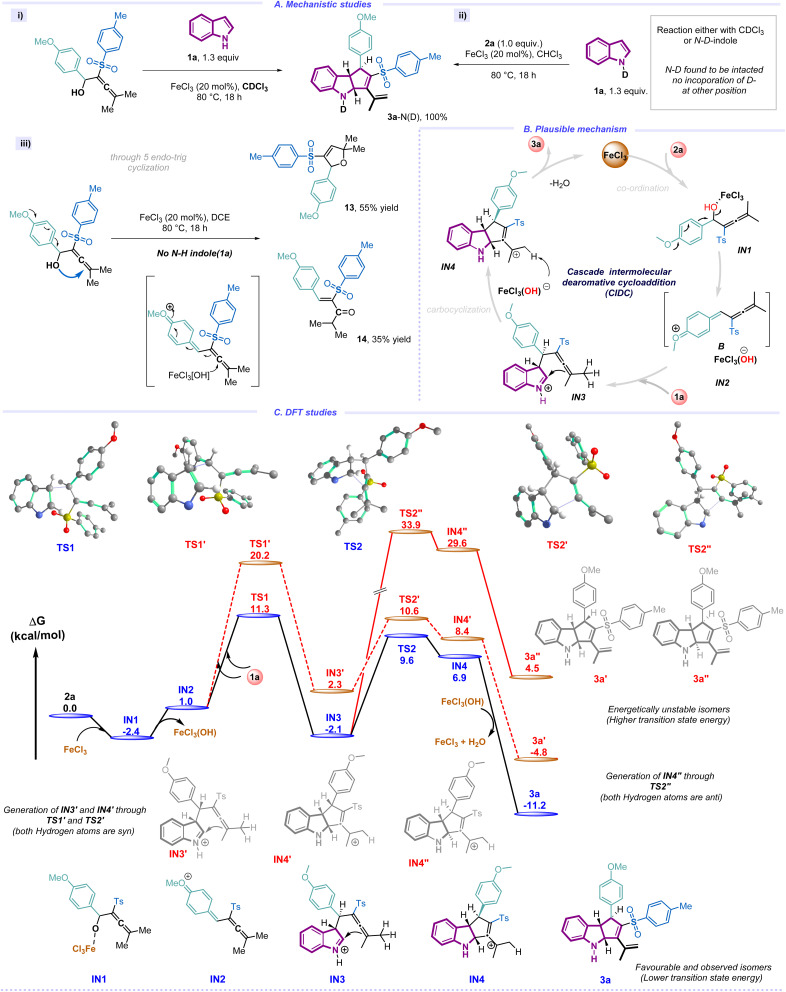
Mechanistic insight and DFT studies. (A) Mechanistic investigation: (i) reaction with deuterated solvent; (ii) reaction with N–D indole under standard conditions; (iii) reaction without N–H indole. (B) Proposed mechanistic cycle. (C) DFT analysis: free energy profile for the formation of 3a. The values are at the M06(SMD)/SDD/def2-TZVP//BP86-D3(BJ)/SDD/def2-SVP level of theory. Non-essential hydrogen atoms are omitted for clarity.

Next, we proceeded to conduct control experiments to gain deeper insights into the underlying mechanism (Fig. 2A). When the reaction was carried out in a deuterated solvent, deuterium was incorporated only at the N–H position of the product, with no labeling observed elsewhere (Fig. 2A(i)). In addition, using an N–D-labeled indole under the standard conditions led to retention of the N–D bond in the final product (Fig. 2A(ii)); both experiments support a stepwise mechanistic pathway. To validate the possible intermediacy of paraquinone methide species, the reaction of 2a without indole led to the formation of a mixture of five-membered oxy-cyclization dihydrofuran^51^ and alpha–beta unsaturated ketone, which indicates the existence of possible paraquinone methide species (Fig. 2A(iii)).^52,53^ Furthermore, when the reaction was carried out in the presence of a TEMPO radical, a slight lowering of desired product formation was noted, which suggested that the reaction follows an ionic pathway. With these control experiments and based on the existing literature,^54^ we proposed a mechanistic cycle, as illustrated in Fig. 2B. The first step involves the coordination of FeCl_3_ to the hydroxy group of aryl sulfonyl allenol, which yields the possible intermediate IN1.^55^ Subsequently, intermediate IN1 transforms into a transient paraquinone-type species IN2 by losing FeCl_3_[OH].^56^ Next, indole 1a immediately reacts with species IN2 to generate another intermediate IN3, driven by nucleophilic attack from the C-3 position of the indole moiety.^57,58^ Instantly, the desired cyclopenta[*b*]indole core intermediate IN4 is formed through nucleophilic attack on the C2-position of indole by the sp-carbon of allene.^59,60^ Furthermore, the abstraction of the methyl proton by FeCl_3_[OH], expelling H_2_O along with FeCl_3_, thus completes the catalytic cycle, which indicates a greener, sustainable, and economical approach for the construction of desired cyclopenta[*b*] indole derivatives.^61^

### Computational studies

To gain deeper insight into the reaction mechanism and the formation of an exclusive stereoselective single isomer, quantum chemical calculations using density functional theory (DFT) were performed, guided by the control experiments and previous literature (Fig. 2C).^62,63^ Efforts to identify a transition state for a concerted reaction were unsuccessful; however, energy surfaces for different stepwise processes were discovered. At first, FeCl_3_ aids in the coordination of sulfonyl allenol, resulting in the formation of the Fe-coordinated intermediate, IN1, through an exergonic process with an energy change of −2.4 kcal mol^−1^. Immediately, the loosely bound adduct IN1 dissociates to generate FeCl_3_(OH), which then forms the intermediate IN2 with an increase in energy of 3.4 kcal mol^−1^. Next, the addition of indole can occur in two distinct orientations to IN2, which leads to the formation of various diastereomers. In one orientation, both hydrogen atoms are positioned above the plane, while in another, one hydrogen atom is above the plane and the other is below. Formation of expected product 3a could occur by construction of the C–C bond at C3 of indole 1a, yielding the stable intermediate IN3*via* transition state TS1, which has an energy barrier of 13.7 kcal mol^−1^. From intermediate IN3, cyclization occurs to form another C–C bond, resulting in the production of two different diastereomers. The observed product is achieved *via* transition state TS2, in which backside attack of the allene pi-electron, with an energy barrier of 11.7 kcal mol^−1^ from IN3, leads to the formation of possible cationic intermediate IN4. The subsequent abstraction of a proton from IN4 by FeCl_3_(OH) produces the stable final product 3a, which is exergonic by 11.2 kcal mol^−1^ with the elimination of FeCl_3_ and water. Moreover, intermediate IN3 can also yield another isomeric product 3a″, through IN4″, *via* transition states TS2″, in which a possible frontside attack of the allene pi-electron occurs at the C-2 position of indole. This opposite orientation leads to the highest energy barrier of 33.9 kcal mol^−1^*via*TS2″, which could transform to unfavourable isomeric product 3a″ (4.5 kcal mol^−1^) *via* the intermediate IN4″. Alternatively, if both hydrogen atoms are positioned above the plane, indole addition leads to the formation of the alternative intermediate IN3′ through transition state TS1′, which presents a higher energy barrier of 22.6 kcal mol^−1^. Moreover, transition state TS2′, which leads to endergonic intermediate IN4′, is further converted into another possible isomer 3a′. Product 3a is 6.4 kcal mol^−1^ and 15.7 kcal mol^−1^ more stable than 3a′ and 3a″, respectively. However, the transition state leading to 3a′ and 3a″ lies higher in energy than TS1/TS2 (leads to 3a). From these data, we conclude that the preference for the formation of product 3a over 3a′ and 3a″ is energetically favourable.^64,65^

## Conclusions

In summary, we have developed a dearomative formal [3 + 2] cycloaddition reaction between indole and aryl sulfonyl allenol for the peripheral editing of indoles along with the installation of diverse functionality such as consecutive three quaternary centers, sulfone, and prenyl units. The transformation proceeds under mild conditions, avoids precious metal catalysts, and demonstrates compatibility with a diverse range of functionalities with consistently high regio- and diastereocontrolled products. The protocol is compatible with a wide range of pharmaceutically relevant substrates, gram-scale scalability, and double cascade dearomative cyclization that provides rapid access to a structurally complex molecular scaffold. Late-stage functionalization and product diversification further highlight the synthetic versatility of the strategy. Additionally, we have undertaken extensive mechanistic investigations to unravel the underlying processes. These included a range of control experiments, such as reactions with deuterated solvents/additives and density functional theory (DFT) analysis, which have collectively supported the formation of diastereoselective cyclopenta[*b*]indole derivatives. Overall, this operationally simple strategy enables efficient construction of nonplanar, tricyclic cyclopenta[*b*]indole derivatives of potential relevance to medicinal chemistry and complex molecule synthesis.

## Author contributions

A. C. S. K. K. and P. S. discovered and developed the reaction, designed the experiments, and analyzed the results. K. K., P. S., and R. D. T. performed the synthesis. A. K. P. and M. V. M. performed the DFT analysis. The manuscript was written through the contributions of all authors. All authors have given approval to the final version of the manuscript.

## Conflicts of interest

There are no conflicts to declare.

## Supplementary Material

SC-OLF-D6SC03994D-s001

SC-OLF-D6SC03994D-s002

## Data Availability

CCDC 2514415 (3a) contains the supplementary crystallographic data for this paper.^66^ The data supporting this article have been included as part of the supplementary information (SI). Supplementary information: experimental procedures, optimization studies, mechanistic studies, and characterization data of new compounds. See DOI: https://doi.org/10.1039/d6sc03994d.
