# Supplementary material for: Stereoselective dearomative formal [3 + 2] cycloaddition of indole with allenols: access to structurally diverse cyclopenta[b]indoles
Source: Chem Sci. 2026 Jun 29;17(33):15897–905. doi: 10.1039/d6sc03994d (PMC13347296; doi:10.1039/d6sc03994d)
Supplement: Supplementary file 1 [file SC-017-D6SC03994D-s001.pdf]

Supplementary Information (SI) for Chemical Science.
This journal is © The Royal Society of Chemistry 2026

Supporting Information for

Stereoselective Dearomative Formal [3+2] Cycloaddition of Indole with Allenols: Access to Structurally Diverse Cyclopenta[b]indoles

Kavneet Kaur,[a$] Puja Singh,[a$] Rahul D. Thombare, [a] Adithya K. P.,[b] Manoj V. Mane,[b] and Aslam C. Shaikh,[a*]

[a] Department of Chemistry, Indian Institute of Technology Ropar, Rupnagar, Punjab 140001, India.

[b] Centre of Nano and Material Sciences, Jain (Deemed-to-be-University), Bangalore 562112 Karnataka, India.

[$] Both authors equally contributed to this work.

E-mail: aslam.shaikh@iitrpr.ac.in.

Table of Contents

1. General Information S2
2 Preparation of starting material substrates S3
2.1 General procedure for the preparation of substituted indoles S3
2.2 General procedure for the preparation of Sulfonyl allenol Substrates 2a-2ao (GP2) S7
2.3 Detailed optimization studies S18
2.4 General procedure for Dearomative Formal [3+2] Cycloaddition Reactions (GP3) S20
2.5 General procedure for the gram-scale reaction S51
2.6 Double dearomative formal [3+2] cycloaddition reaction S52
2.7 Product diversification S53
2.7 Control experiments: S58
3. X-ray Crystallographic Data for 3a S60
4. Computational studies on the mechanism S62
5. NMR Spectra for all compounds S74

1. General Information

Unless otherwise specified, all reactions were carried out in oven-dried vials or reaction vessels with magnetic stirring under an argon atmosphere. Oven-dried syringes or hypodermic syringes were transferred to dried solvents and liquid reagents cooled to ambient temperature in a desiccator. All experiments were monitored by analytical thin-layer chromatography (TLC). TLC was performed on pre-coated silica gel plates. After elution, the plate was visualized under UV illumination at 254 nm and 365 nm for UV-active materials. Further visualization was achieved by staining $KMnO_4$ and charring on a hot air gun. Solvents were removed in vacuo and heated with a water bath at 35 °C. Silica gel finer than 200 mesh was used for flash column chromatography. Columns were packed as a slurry of silica gel in hexane and equilibrated with the appropriate solvent mixture prior to use. The compounds were loaded solid or as a concentrated solution using the appropriate solvent system. The elution was assisted by applying pressure with an air pump.

NMR spectroscopy. NMR spectra were recorded on JEOL 400 spectrometer in deuterated solvents using TMS as internal standard, or the solvent residue signals as secondary standards, and the chemical shifts are shown in δ scales. Deuterated solvents were degassed by three freeze-pump-thaw cycles and then dried by storing over molecular sieves (3 or 4 Å) for at least one day before use. Multiplicities of the 1H NMR signals are denoted by s(singlet), d (doublet), dd (doublet of doublet), dt (doublet of the triplet), t (triplet), quin (quintet), m (multiplet), br.s (broad singlet) etc. Compounds were drawn using ChemDraw and the assignments of NMR spectra were done on MestReNova.

Mass spectrometry. High-resolution mass spectra (HRMS) were recorded on an Agilent 6538 UHD Q-TOF electron spray ionization (ESI) mode and atmospheric pressure chemical ionization (APCI) modes.

X-ray crystallography. Single-crystal X-ray diffraction data were collected on Bruker D8 Venture X-ray diffractometer having a micro-focus sealed X-ray tube MoKα (λ = 0.71073 Å) source of X-rays along with a PHOTON 100 detector with inclining Phi and Omega (width of 0.5 for one frame) working at a scan speed of 10 s per frame. The crystal was kept at 298 K during data collection.

2 Preparation of starting material substrates

2.1 General procedure for the preparation of substituted indoles

a. Most of the substituted indoles (**1a**-**1s**) are purchased from Sigma-Aldrich.

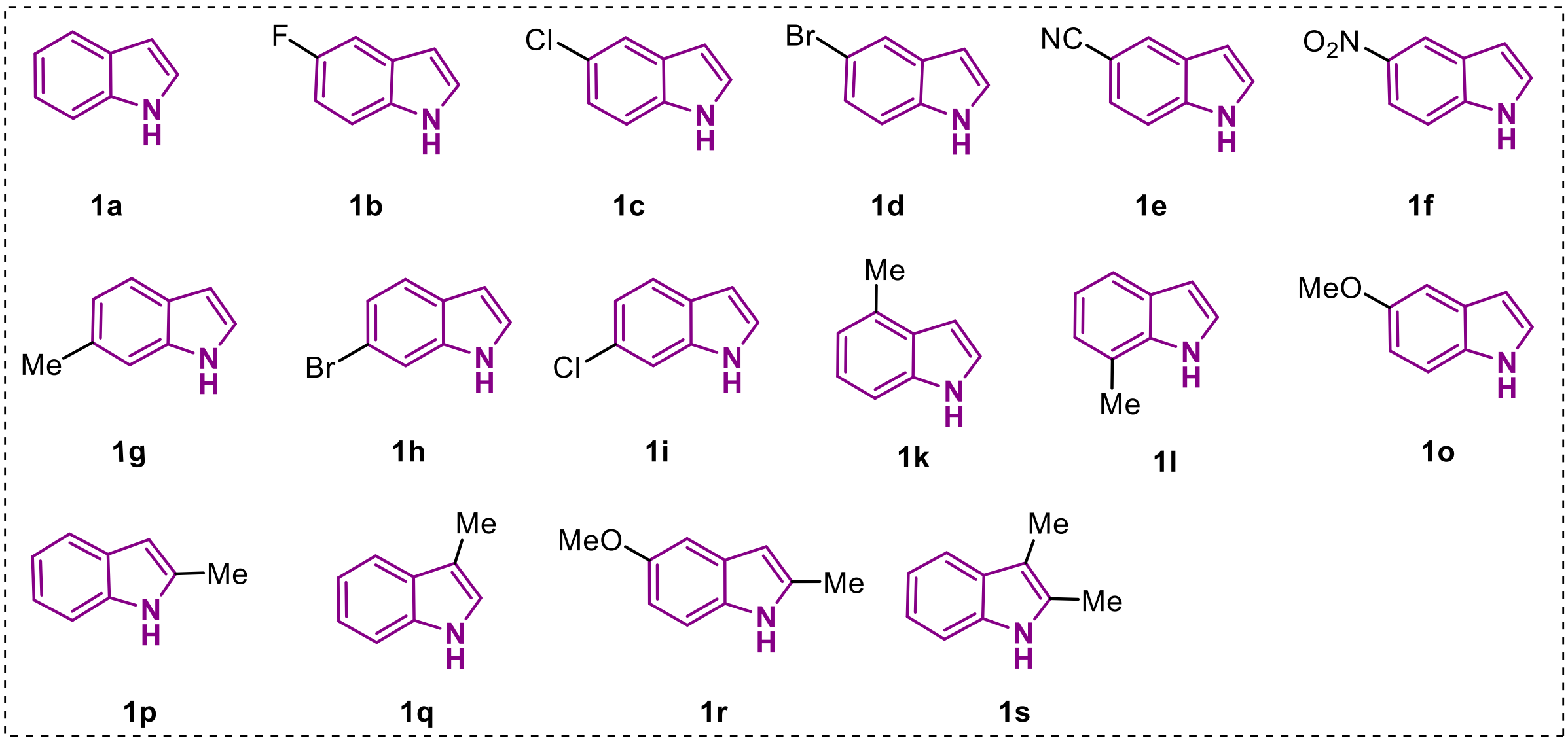

b. According to the previous literature,[1] All the *N*-alkyl (aryl) protected indoles (**S1**) have been synthesised (**1w**-**1ae**).

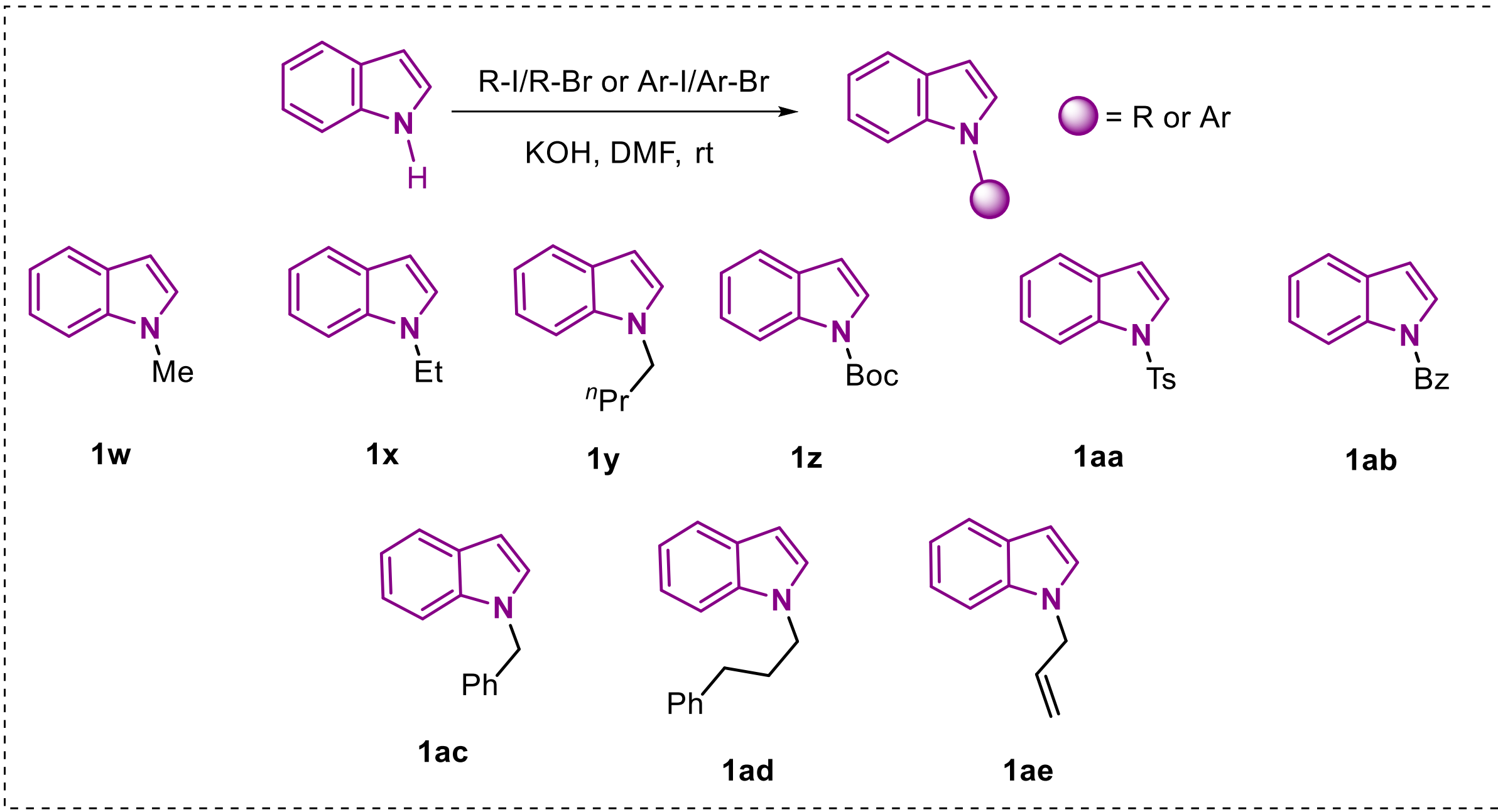

[1] a) S.-p. Ge, X.-h. Zhang, J.-s. Han, P. Zhong. *J. Chem. Res.*, **2012**, *36*, 356-359; b) B. Liu, M. Liu, Q. Li, Y. Li, K. Fenga, Y. Zhou, *Org. Biomol. Chem.*, **2020**, *18*, 6108-6114.

c. According to previous literature,[2] Indole **1j-1v** has been synthesized, and the characterization data match the reported data.[3]

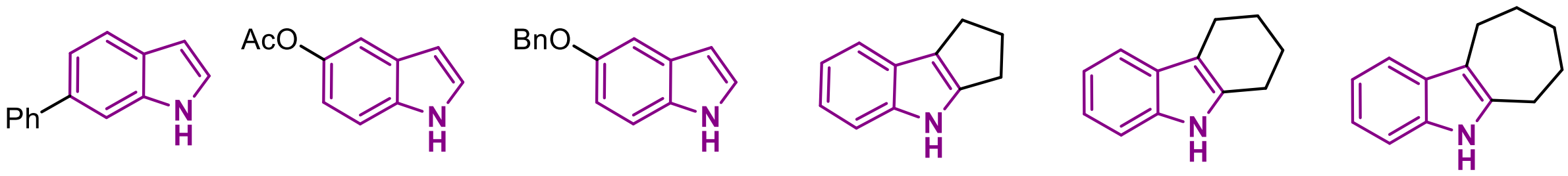

1j **1m** **1n** **1t** **1u** **1v**

d. General procedure for the preparation of substituted indoles through Dicyclohexylcarbodiimide (DCC) and catalytic dimethylaminopyridine (DMAP) coupling (GP1):

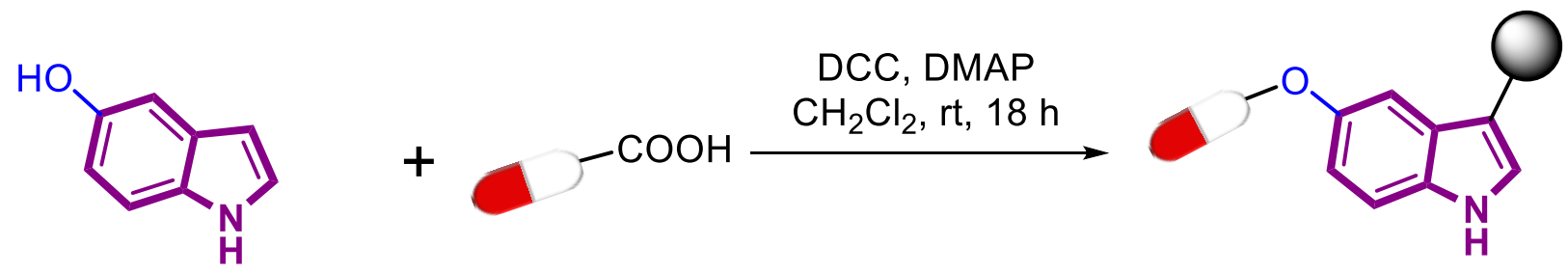

4 **5** **6**

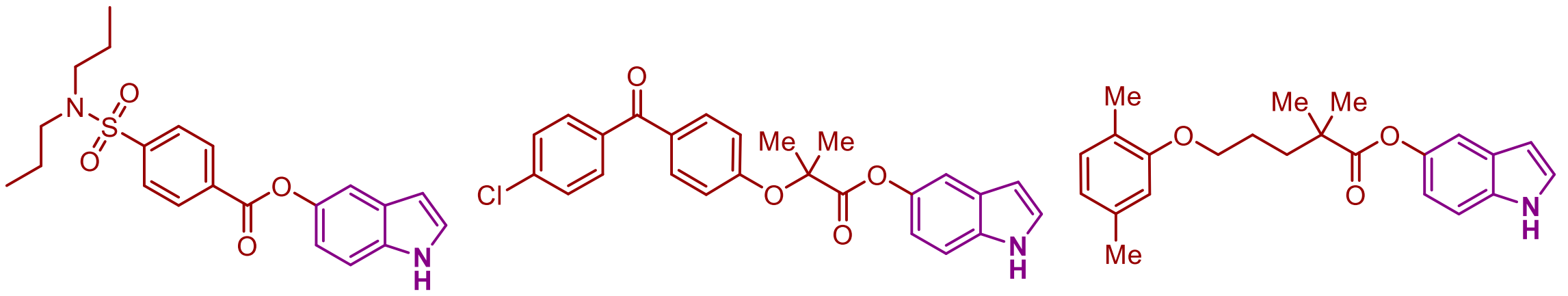

6a **6b** **6c**

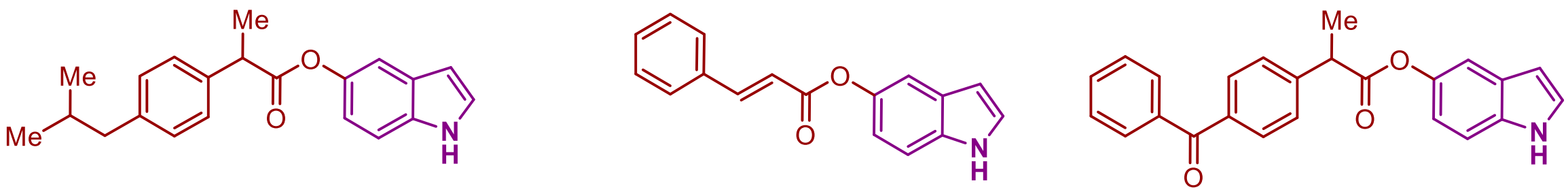

6d **6e** **6f**

Under air atmosphere, a 5 mL glass vial equipped with a magnetic stir bar was charged with an acid analogue (2.00 mmol, 1.00 equiv.), 1H-indol-5-ol (2.00 mmol, 1.00 equiv.), DCC (3.00 mmol, 1.50 equiv.), DMAP (0.400 mmol, 20 mol%), and DCM (2.0 mL). The vial was sealed with a Teflon cap; the reaction mixture was stirred at room temperature for 12 h. After that, the reaction mixture was diluted with DCM (ca. 20 mL) and H_2O (ca. 10 mL), transferred into a separatory funnel, and the layers were separated. The organic layer was collected, and the aqueous layer was further extracted with DCM (2 × ca. 20 mL). The combined organic layer was dried over Na_2SO_4, filtered, and concentrated under reduced pressure to remove all

[2] a) S.-p. Ge, X.-h. Zhang, J.-s. Han, P. Zhong. *J. Chem. Res.*, **2012**, *36*, 356-359; b) B. Liu, M. Liu, Q. Li, Y. Li, K. Fenga, Y. Zhou, *Org. Biomol. Chem.*, **2020**, *18*, 6108-6114.

[3] Hayashi, M.; Miki, K.; Matsubara, R. Non-aerobic and One-Pot Synthesis of Carbazoles from Cyclohexanones and Arylhydrazines. *Synthesis* **2025**, 57 (09), 1599–1606.

volatiles. The residue was then purified by chromatography on silica gel (EA/Hexane = 1/6) to afford **6a-6f**. The characterization data for **6a**, **6c**, and **6d** matched the literature report.

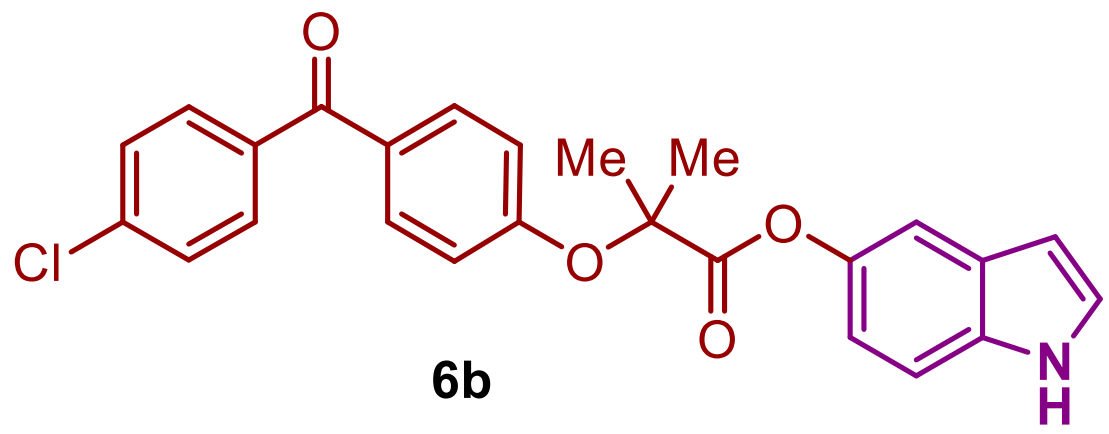

6b

1H-indol-5-yl 2-(4-(4-chlorobenzoyl)phenoxy)-2-methylpropanoate (6b): Prepared according to GP-1 on 3.37 mmol (500 mg)

Light brown solid (625 mg, 70% yield)

Rf = 0.38 (25% EA/hexane)

Melting point = 134-136°C

NMR Spectroscopy:

^{1}H NMR (400 MHz, CHLOROFORM-D) δ 8.33 (s, 1H), 7.88 – 7.78 (m, 2H), 7.77 – 7.67 (m, 2H), 7.51 – 7.42 (m, 2H), 7.31 (dd, *J* = 8.7, 0.5 Hz, 1H), 7.22 (t, *J* = 2.8 Hz, 2H), 7.10 – 6.98 (m, 2H), 6.78 (dd, *J* = 8.7, 2.3 Hz, 1H), 6.58 – 6.44 (m, 1H), 1.86 (s, 6H) ppm.

^{13}C NMR (101 MHz, CHLOROFORM-D) δ 194.52, 173.38, 159.84, 144.18, 138.58, 136.47, 133.91, 132.32, 131.36, 130.6, 128.7, 128.2, 125.9, 117.4, 115.5, 112.4, 111.6, 103.0, 79.6, 25.6 ppm.

HRMS (ESI) m/z: [M+H]+ Calcd for $C_{25}H_{21}ClNO_4^+$ 434.8915; Found 434.8925.

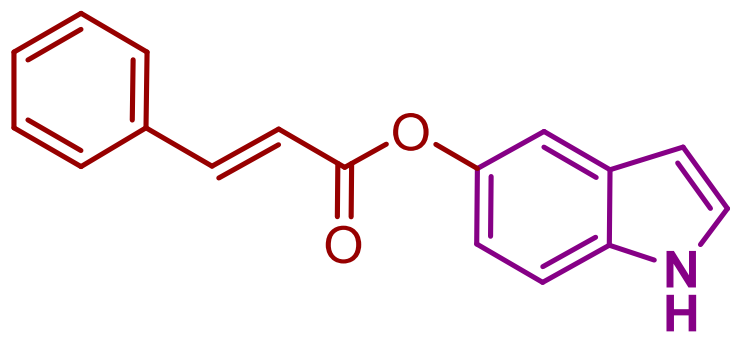

6e

1H-indol-5-yl cinnamate (6e): Prepared according to GP-1 on 4.03 mmol (500 mg)

Brown solid (520 mg, 72% yield)

Rf = 0.38 (25% EA/hexane)

Melting point = 148-150°C

NMR Spectroscopy:

^{1}H NMR (400 MHz, CHLOROFORM-D) δ 8.23 (s, 1H), 7.90 (d, *J* = 16.0 Hz, 1H), 7.66 – 7.57 (m, 2H), 7.47 – 7.36 (m, 5H), 7.25 – 7.23 (m, 1H), 7.00 (dd, *J* = 8.7, 2.3 Hz, 1H), 6.69 (d, *J* = 16.0 Hz, 1H), 6.56 (t, *J* = 2.2 Hz, 1H) ppm.

^{13}C NMR (101 MHz, CHLOROFORM-D) δ 166.5, 146.2, 144.5, 134.4, 133.8, 130.7, 129.1, 128.4, 128.3, 125.6, 117.8, 116.3, 112.9, 111.6, 103.1 ppm.

HRMS (ESI) m/z: [M+H]+ Calcd for $C_{17}H_{14}NO_2^+$ 264.2986; Found 264.2990.

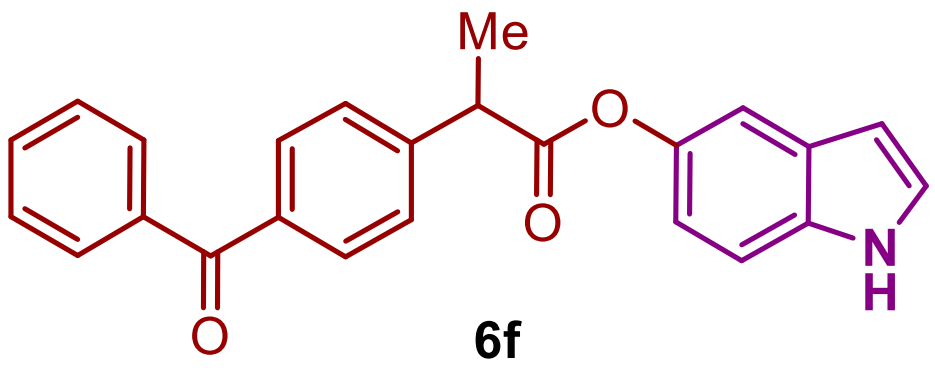

6f

1H-indol-5-yl 2-(4-benzoylphenyl)propanoate (6f): Prepared according to GP-1 on 1.96 mmol (500 mg)

White solid (570 mg, 78% yield)

Rf = 0.38 (25% EA/hexane)

Melting point = 165-167°C

NMR Spectroscopy:

^{1}H NMR (400 MHz, CHLOROFORM-D) δ 8.56 (s, 1H), 7.91 (t, *J* = 1.7 Hz, 1H), 7.87 – 7.79 (m, 2H), 7.79 – 7.71 (m, 1H), 7.68 (dd, *J* = 6.5, 1.3 Hz, 1H), 7.57 (dd, *J* = 10.6, 4.3 Hz, 1H), 7.48 (dt, *J* = 13.9, 7.6 Hz, 3H), 7.24 (t, *J* = 5.7 Hz, 1H), 7.17 (d, *J* = 8.7 Hz, 1H), 7.11 – 7.04 (m, 1H), 6.76 (dd, *J* = 8.7, 2.3 Hz, 1H), 6.44 (t, *J* = 2.3 Hz, 1H), 4.08 (q, *J* = 7.2 Hz, 1H), 1.67 (d, *J* = 7.2 Hz, 3H) ppm.

^{13}C NMR (101 MHz, CHLOROFORM-D) δ 196.9, 173.7, 144.2, 140.7, 138.0, 137.4, 133.8, 132.7, 131.7, 130.2, 129.4, 129.2, 128.9, 128.4, 128.0, 125.8, 115.5, 112.3, 111.6, 102.5, 45.6, 33.9, 24.9, 18.7 ppm.

HRMS (ESI) m/z: [M+H]+ Calcd for $C_{24}H_{20}NO_3^+$ 370.1443; Found 370.1449.

e. General procedure for the preparation of sulfonyl allene (S):

Sulfonyl Allene **S** was synthesized according to the previously established literature and our protocol [4] in two steps.[5]

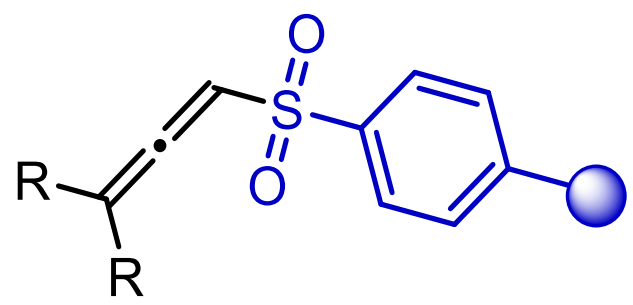

The spectroscopic data for all the sulfonyl allenes have been compared and matched with the reported literature data.[6,7, 8]

[4] a) M. Harmata, C. F. Huang. *Adv. Synth. Catal*., **2008**, *350*, 972 – 974. b) P. Singh, Deepshikha, N. Lal, M. V. Mane, A. C. Shaikh, *Org. Lett.* **2025**, *27*, 1153–1158.

[5] a) M. Yoshida, M. Hayashi, K. Shishido, *Org. Lett.* **2007**, *9*, 1643-1646; b) L. Mao, R. Bertermann, K. Emmert, K. J. Szabó, T. B. Marder, *Org. Lett*. **2017**, *19*, 6586–6589.

[6] C. S. Hampton, M. Harmata, *Adv. Synth. Catal*. **2015**, *357*, 549 – 552.

[7] a) R. R. Tata, C. S. Hampton, E. F. Altenhofer, M. Topinka, W. Ying, X. Gao, M. Harmata, *Chem. Eur. J.* **2014**, *20*, 13547-13550; b) G. S. Ghotekar, R. A. Shinde, S. S. Saswade, M. Muthukrishnan, *J. Org. Chem.* **2023**, *88*, 4112-4122

[8] a) R. R. Tata, C. S. Hampton, E. F. Altenhofer, M. Topinka, W. Ying, X. Gao, M. Harmata, *Chem. Eur. J.* **2014**, *20*, 13547-13550; b) G. S. Ghotekar, R. A. Shinde, S. S. Saswade, M. Muthukrishnan, *J. Org. Chem.* **2023**, *88*, 4112-4122.

2.2 General procedure for the preparation of Sulfonyl allenol Substrates 2a-2ao (GP2)

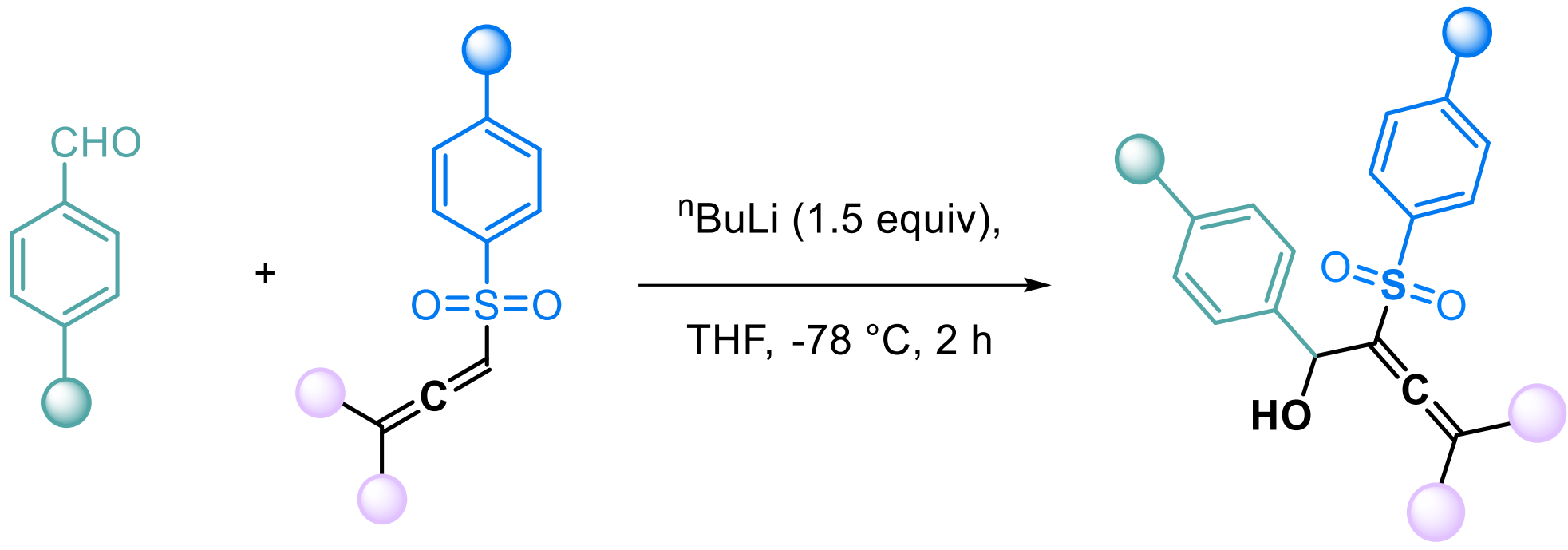

(1 equiv) **S** (1.5 equiv) **2a-2ao**

According to the previous literature,[9] and our work,[10] Dissolve allene sulfone **S** (9.4 mmol, 1.5 equiv) in dry THF (20 ml) under a nitrogen atmosphere. Subsequently, cool the reaction mixture to -78 °C. Gradually, add a solution of n-BuLi (1.6 M in hexane, 9.4 mmol, 1.5 equiv) dropwise to the reaction mixture at -78 °C. Stir the reaction mixture for 20 minutes at -78 °C. Slowly introduce a solution of p-anisaldehyde (6.3 mmol, 1 equiv) in THF to the reaction mixture at -78 °C. Stir the reaction mixture at -78 °C for 2 hours. Quench the reaction mixture with saturated NH_4Cl. Extract the reaction mixture with dichloromethane. Subsequently, dry the combined organic layer over Na_2SO_4. Concentrate the combined organic layer by rotary evaporation. Purify the combined organic layer by column chromatography over silica gel.

The spectroscopic data for the previously synthesized compounds have been compared and matched with the reported literature data.

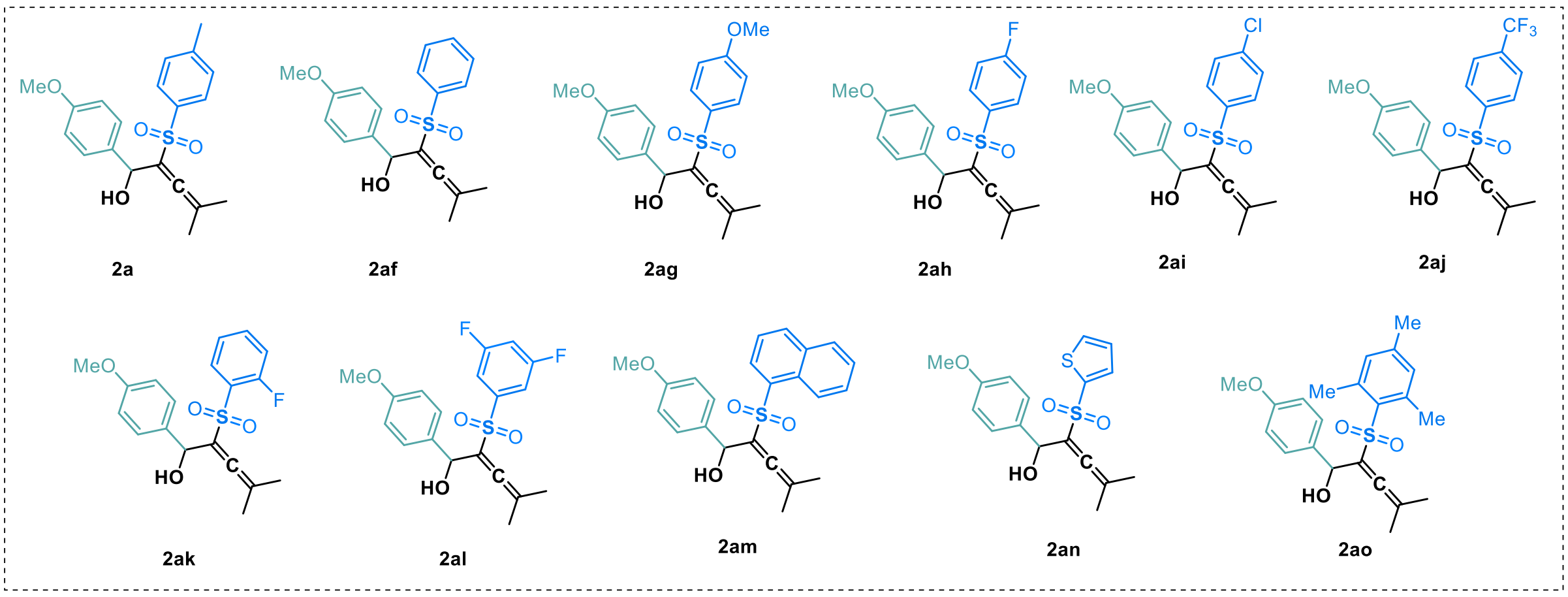

[9] a) R. R. Tata, C. S. Hampton, E. F. Altenhofer, M. Topinka, W. Ying, X. Gao, M. Harmata, *Chem. Eur. J.* **2014**, *20*, 13547-13550; b) G. S. Ghotekar, R. A. Shinde, S. S. Saswade, M. Muthukrishnan, *J. Org. Chem.* **2023**, *88*, 4112-4122.

[10] P. Singh, M. V. Mane, S. Mahto, A. C. Shaikh, *Org. Lett*. **2025**, https://doi.org/10.1021/acs.orglett.5c02333.

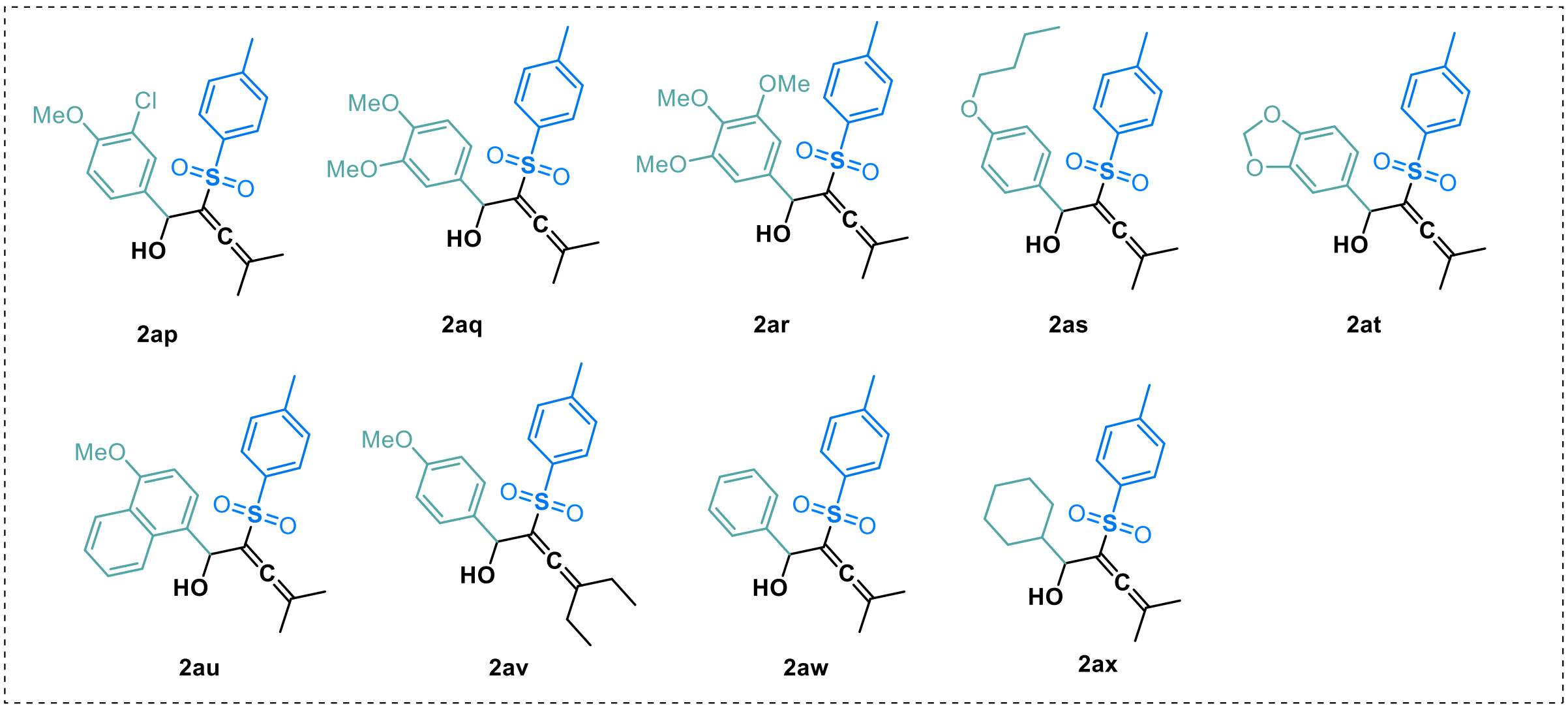

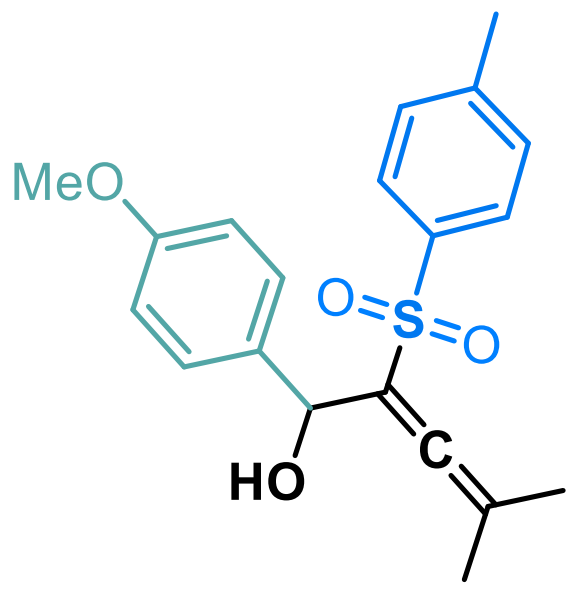

2a

1-(4-methoxyphenyl)-4-methyl-2-tosylpenta-2,3-dien-1-ol (2a): Prepared according to GP-2 on 7.34 mmol (1000 mg)

Pale Yellow solid (2000 mg, 76% yield)

Rf = 0.35 (25% EA/hexane)

Melting point = 115-117°C

NMR Spectroscopy:

^{1}H NMR (400 MHz, CHLOROFORM-D) δ 7.66 (d, *J* = 8.3 Hz, 2H), 7.27 – 7.24 (m, 2H), 7.12 (d, *J* = 8.7 Hz, 2H), 6.76 (d, *J* = 8.7 Hz, 2H), 5.56 (d, *J* = 3.9 Hz, 1H), 3.77 (s, 3H), 3.36 (d, *J* = 4.3 Hz, 1H), 2.42 (s, 3H), 1.66 (s, 3H), 1.55 (s, 3H) ppm.

^{13}C NMR (101 MHz, CHLOROFORM-D) δ 202.8, 158.9, 143.9, 137.9, 132.4, 129.4, 127.5, 127.4, 114.7, 113.2, 108.3, 70.4, 55.1, 21.4, 19.1, 18.9 ppm.

HRMS (ESI) m/z: [M+Na]+ Calcd for $C_{20}H_{22}NaO_4S^+$ 381.1136; Found 381.1136.

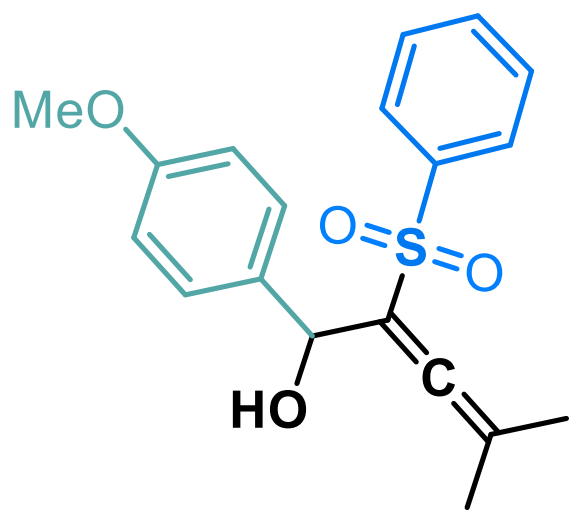

2af

1-(4-methoxyphenyl)-4-methyl-2-(phenylsulfonyl)penta-2,3-dien-1-ol (2af): Prepared according to GP-2 on 4.03 mmol (550 mg)
Yellow Thick Liquid (1000 mg, 72% yield)
Rf = 0.38 (25% EA/hexane)
NMR Spectroscopy:
^{1}H NMR (400 MHz, CHLOROFORM-D) δ 7.75 (d, *J* = 7.3 Hz, 2H), 7.56 (t, *J* = 7.3 Hz, 1H), 7.45 (t, *J* = 7.6 Hz, 2H), 7.11 (d, *J* = 8.6 Hz, 2H), 6.74 (d, *J* = 8.7 Hz, 2H), 5.57 (d, *J* = 4.5 Hz, 1H), 3.75 (s, 3H), 3.32 (s, 1H), 1.65 (s, 3H), 1.54 (s, 3H) ppm.
^{13}C NMR (101 MHz, CHLOROFORM-D) δ 203.2, 159.2, 141.1, 133.2, 132.3, 129.0, 127.7, 127.6, 114.7, 113.5, 108.7, 70.9, 55.3, 55.3, 19.3, 19.2 ppm.
HRMS (ESI) m/z: [M+Na]+ Calcd for $C_{19}H_{20}NaO_4S^+$ 367.0980; Found 367.0972.

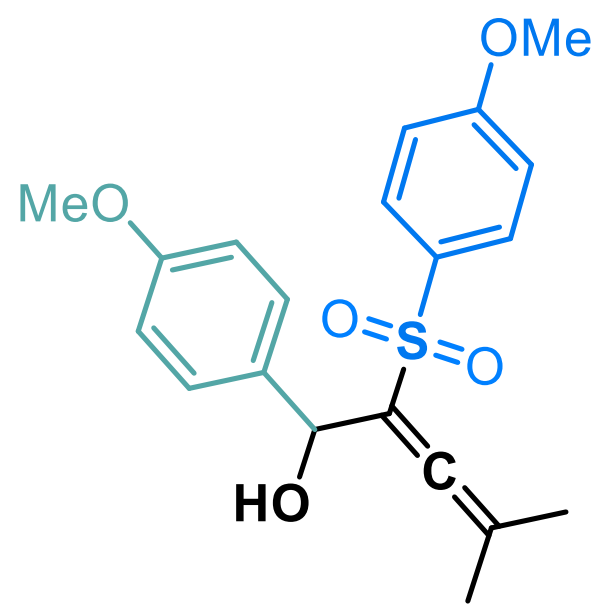

2ag

1-(4-methoxyphenyl)-2-((4-methoxyphenyl)sulfonyl)-4-methylpenta-2,3-dien-1-ol(2ag):
Prepared according to GP-2 on 3.67mmol (500 mg)
Yellow Thick Liquid (248 mg, 99% yield)
Rf = 0.50 (20% EA/hexane)
NMR Spectroscopy:
^{1}H NMR (400 MHz, CHLOROFORM-D) δ 7.67 (d, *J* = 9.0 Hz, 2H), 7.12 (d, *J* = 8.7 Hz, 2H), 6.90 (d, *J* = 9.0 Hz, 2H), 6.75 (d, *J* = 8.7 Hz, 2H), 5.55 (s, 1H), 3.85 (s, 3H), 3.76 (s, 3H), 3.40 (s, 1H), 1.66 (s, 3H), 1.55 (s, 3H) ppm.
^{13}C NMR (101 MHz, CHLOROFORM-D) δ 202.7, 163.4, 159.2, 132.6, 132.4, 129.9, 127.6, 114.9, 114.2, 113.5, 108.3, 70.9, 55.8, 55.7, 55.3, 19.4, 19.3 ppm.
HRMS (ESI) m/z: [M+Na]+ Calcd for $C_{20}H_{22}NaO_5S^+$ 397.1086; Found 397.1082.

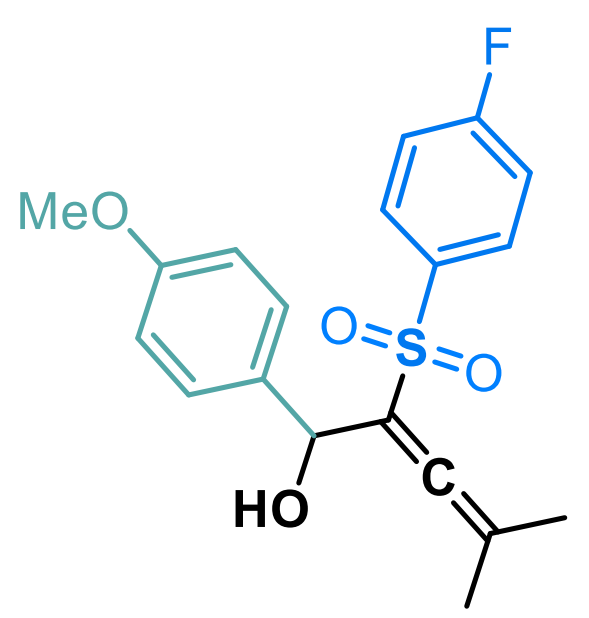

2ah

2-((4-fluorophenyl)sulfonyl)-1-(4-methoxyphenyl)-4-methylpenta-2,3-dien-1-ol(2ah):
Prepared according to GP-2 on 3.67 mmol (500 mg)

Orange solid (800 mg, 60% yield)
Rf = 0.40 (25% EA/hexane)
Melting point = 88-90°C
NMR Spectroscopy:
^{1}H NMR (400 MHz, CHLOROFORM-D) δ 7.70 (dd, *J* = 9.0, 5.1 Hz, 2H), 7.16 – 6.99 (m, 4H), 6.72 (d, *J* = 8.8 Hz, 2H), 5.54 (s, 1H), 3.72 (s, 3H), 1.65 (s, 3H), 1.56 (s, 3H) ppm.
^{13}C NMR (101 MHz, CHLOROFORM-D) δ 203.2, 166.7-164.2 (d, *J* = 255.7 Hz), 159.3, 137.4, 137.3, 132.2, 130.5-130.5 (d, *J* = 9.5 Hz), 127.6, 116.3-116.1 (d, *J* = 22.7 Hz), 114.6, 113.6, 108.8, 70.9, 55.3, 19.3, 19.3 ppm.
^{19}F NMR (376 MHz, CHLOROFORM-D) δ -104.31 ppm.
HRMS (ESI) m/z: [M+Na]+ Calcd for $C_{19}H_{19}FNaO_4S^+$ 385.0886; Found 385.0877.

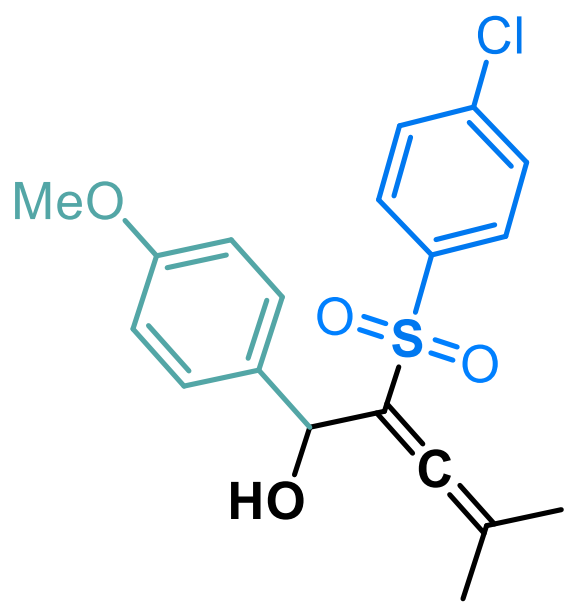

2ai

2-((4-chlorophenyl)sulfonyl)-1-(4-methoxyphenyl)-4-methylpenta-2,3-dien-1-ol (2ai):
Prepared according to GP-2 on 2.93 mmol (400 mg)
Yellow solid (800 mg, 72% yield)
Rf = 0.40 (25% EA/hexane)
Melting point = 88-90°C
NMR Spectroscopy:
^{1}H NMR (400 MHz, CHLOROFORM-D) δ 7.65 (d, *J* = 8.6 Hz, 2H), 7.40 (d, *J* = 8.7 Hz, 2H), 7.11 (d, *J* = 8.6 Hz, 2H), 6.75 (d, *J* = 8.8 Hz, 2H), 5.58 (d, *J* = 4.8 Hz, 1H), 3.77 (s, 3H), 3.19 (d, *J* = 4.9 Hz, 1H), 1.71 (s, 3H), 1.62 (s, 3H) ppm.
^{13}C NMR (101 MHz, CHLOROFORM-D) δ 203.1, 152.9, 144.3, 138.2, 137.3, 135.8, 129.6, 127.7, 114.4, 108.4, 103.2, 71.4, 60.9, 56.0, 21.7, 19.4, 19.3 ppm.
HRMS (ESI) m/z: [M+Na]+ Calcd for $C_{19}H_{19}ClNaO_4S^+$ 401.0590; Found 401.0582.

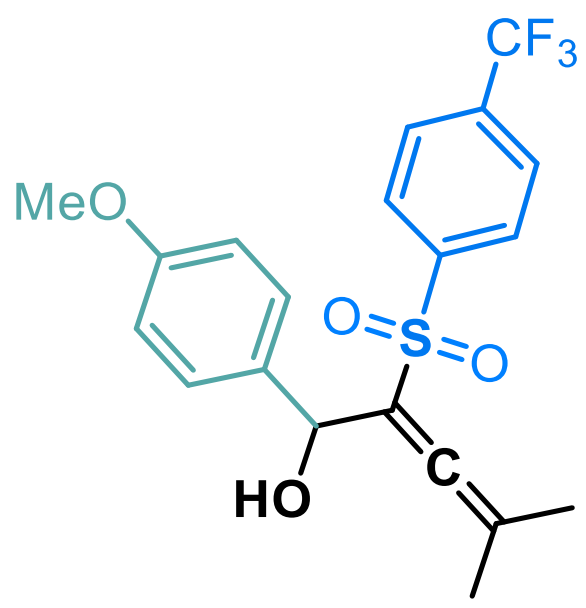

2aj

1-(4-methoxyphenyl)-4-methyl-2-((4-(trifluoromethyl)phenyl)sulfonyl)penta-2,3-dien-1-ol (2aj): Prepared according to GP-2 on 1.80 mmol (250 mg)

Pale Yellow solid (700 mg, 78% yield)

Rf = 0.40 (30% EA/hexane)

Melting point = 89-91°C

NMR Spectroscopy:

^{1}H NMR (400 MHz, CHLOROFORM-D) δ 7.86 – 7.80 (m, 2H), 7.68 (d, J = 8.3 Hz, 2H), 7.14 – 7.07 (m, 2H), 6.78 – 6.70 (m, 2H), 5.61 (d, J = 5.0 Hz, 1H), 3.77 (s, 3H), 3.07 (d, J = 5.2 Hz, 1H), 1.76 (s, 3H), 1.68 (s, 3H) ppm.

^{13}C NMR (101 MHz, CHLOROFORM-D) δ 203.8, 159.5, 145.1, 131.8, 128.1, 127.7, 114.1, 113.6, 109.2, 71.1, 55.3, 19.4, 19.3 ppm.

^{19}F NMR (376 MHz, CHLOROFORM-D) δ = -63.15 ppm.

HRMS (ESI) m/z: [M+Na]+ Calcd for $C_{20}H_{19}O_4NaSF_3^+$ 435.0854; Found 435.0850.

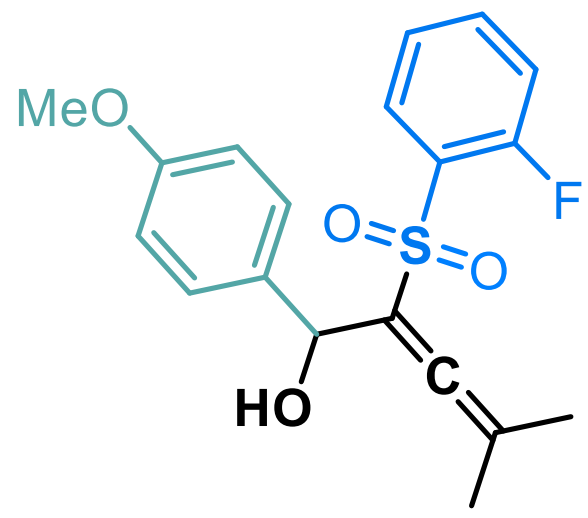

2ak

2-((2-fluorophenyl)sulfonyl)-1-(4-methoxyphenyl)-4-methylpenta-2,3-dien-1-ol(2ak): Prepared according to GP-2 on 1.79 mmol (500 mg)

Yellow solid (700 mg, 78% yield)

Rf = 0.40 (25% EA/hexane)

Melting point = 75-77°C

NMR Spectroscopy:

^{1}H NMR (400 MHz, CHLOROFORM-D) δ 7.79 – 7.70 (m, 1H), 7.59 – 7.50 (m, 1H), 7.23 – 7.08 (m, 4H), 6.71 (d, *J* = 8.7 Hz, 2H), 5.56 (d, *J* = 4.4 Hz, 1H), 3.74 (s, 3H), 3.17 (d, *J* = 4.7 Hz, 1H), 1.77 (s, 3H), 1.65 (s, 3H) ppm.

^{13}C NMR (101 MHz, CHLOROFORM-D) δ 203.7, 160.4, 159.2, 157.9, 135.7, 135.6, 132.1, 130.4, 128.9, 128.8, 127.5, 124.4, 124.4, 117.1, 116.8, 113.7, 113.5, 109.4, 70.9, 55.3, 19.4, 19.2 ppm.

^{19}F NMR (376 MHz, CHLOROFORM-D) δ = -107.68 ppm.

HRMS (ESI) m/z: [M+Na]+ Calcd for $C_{19}H_{19}FNaO_4S^+$ 385.0886; Found 385.0883.

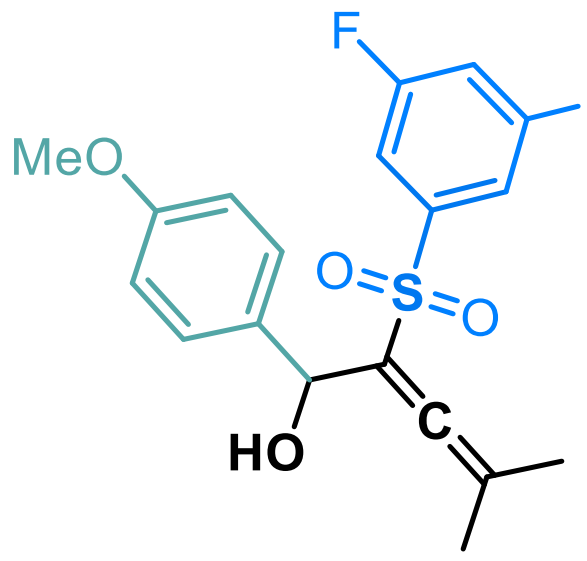

2al

2-((3,5-difluorophenyl)sulfonyl)-1-(4-methoxyphenyl)-4-methylpenta-2,3-dien-1-ol(2al):
Prepared according to GP-2 on 2.49 mmol (340 mg)
White solid (600 mg, 63% yield)
Rf = 0.38 (25% EA/hexane)
Melting point = 80-82°C
NMR Spectroscopy:
^{1}H NMR (400 MHz, CHLOROFORM-D) δ 7.18 (dd, *J* = 6.4, 2.2 Hz, 2H), 7.12 (d, *J* = 8.7 Hz, 2H), 6.97 (tt, *J* = 8.3, 2.3 Hz, 1H), 6.76 (d, *J* = 8.8 Hz, 2H), 5.59 (s, 1H), 3.78 (s, 3H), 3.03 (s, 1H), 1.79 (s, 3H), 1.71 (s, 3H) ppm.
^{13}C NMR (101 MHz, CHLOROFORM-D) δ 203.9, 163.9-161.3 (d, *J* = 253.0 Hz) 163.8-161.2 (d, *J* = 256.0 Hz), 159.5, 145.0, 131.8, 127.7, 114.0, 113.7, 111.3, 111.0, 109.4, 108.8-108.4 (t, *J* = 25.0 Hz), 71.2, 55.3, 19.4, 19.3 ppm.
^{19}F NMR (376 MHz, CHLOROFORM-D) δ = -106.02 ppm.
HRMS (ESI) m/z: [M+Na]+ Calcd for $C_{19}H_{18}F_2NaO_4S^+$ 403.0792; Found 403.0783.

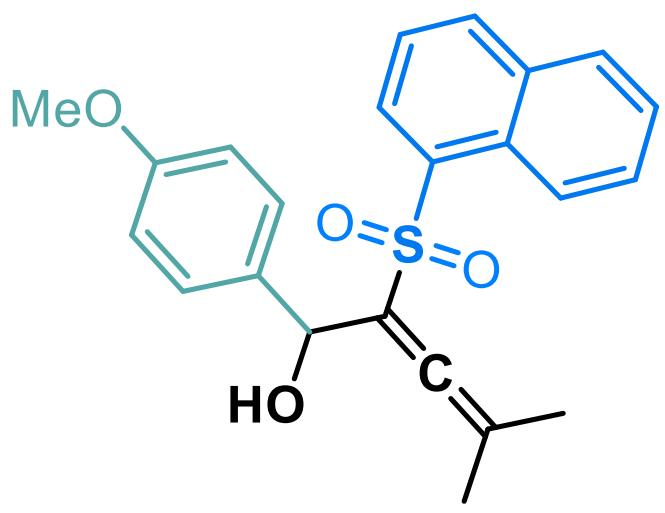

2am

1-(4-methoxyphenyl)-4-methyl-2-(naphthalen-1-ylsulfonyl)penta-2,3-dien-1-ol(2am):
Prepared according to GP-2 on 2.20 mmol (300 mg)
Pale Yellow solid (800 mg, 92% yield)
Rf = 0.39 (25% EA/hexane)
Melting point = 105-107°C
NMR Spectroscopy:
^{1}H NMR (400 MHz, CHLOROFORM-D) δ 8.19 (s, 1H), 7.90 – 7.83 (m, 3H), 7.69 (dd, J = 8.6, 1.9 Hz, 1H), 7.60 (dddd, J = 21.2, 8.0, 6.9, 1.4 Hz, 2H), 7.07 (d, J = 8.6 Hz, 2H), 6.62 (d, J = 8.8 Hz, 2H), 5.59 (d, J = 4.7 Hz, 1H), 3.61 (s, 3H), 3.37 (d, J = 4.9 Hz, 1H), 1.70 (s, 3H), 1.61 (s, 3H) ppm.
^{13}C NMR (101 MHz, CHLOROFORM-D) δ 203.4, 159.1, 138.0, 134.9, 132.1, 129.5, 129.4, 129.3, 129.1, 127.9, 127.6, 127.6, 122.4, 114.4, 113.4, 108.6, 71.1, 55.2, 19.4, 19.4 ppm.
HRMS (ESI) m/z: [M+Na]+ Calcd for $C_{23}H_{22}NaO_4S^+$ 417.1136; Found 417.1130.

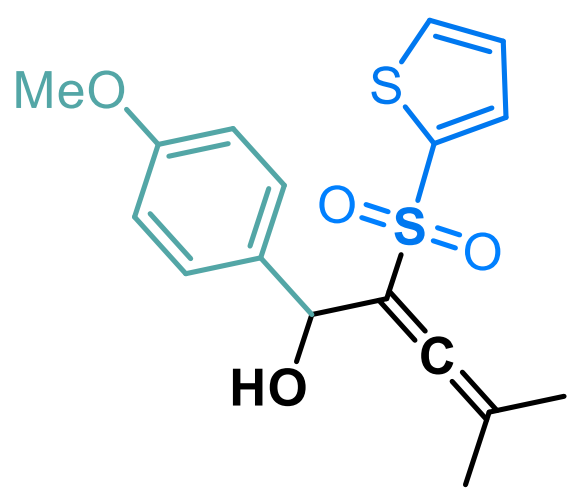

2an

1-(4-methoxyphenyl)-4-methyl-2-(thiophen-2-ylsulfonyl)penta-2,3-dien-1-ol (2an):
Prepared according to GP-2 on 3.67 mmol (500 mg)
Yellow Thick Liquid (700 mg, 78% yield)
Rf = 0.34 (25% EA/hexane)
NMR Spectroscopy:
^{1}H NMR (400 MHz, CHLOROFORM-D) δ 7.64 (dd, *J* = 5.0, 1.3 Hz, 1H), 7.49 (dt, *J* = 3.9, 1.2 Hz, 1H), 7.15 (d, *J* = 8.6 Hz, 2H), 7.05 – 7.00 (m, 1H), 6.78 (d, *J* = 8.5 Hz, 2H), 5.62 (d, *J* = 4.4 Hz, 1H), 3.77 (d, *J* = 0.6 Hz, 3H), 3.25 (dd, *J* = 8.9, 4.5 Hz, 1H), 1.72 (s, 3H), 1.60 (s, 3H) ppm.
^{13}C NMR (101 MHz, CHLOROFORM-D) δ 202.9, 149.4, 143.7, 138.2, 136.3, 132.6, 129.2, 127.9, 127.8, 127.4, 126.2, 121.0, 116.8, 114.0, 113.8, 113.2, 109.5, 108.3, 66.6, 41.0, 32.9, 21.7, 19.7, 19.5 ppm.
HRMS (ESI) m/z: [M+Na]+ Calcd for $C_{17}H_{18}NaO_4S_2^+$ 373.0544; Found 373.0536.

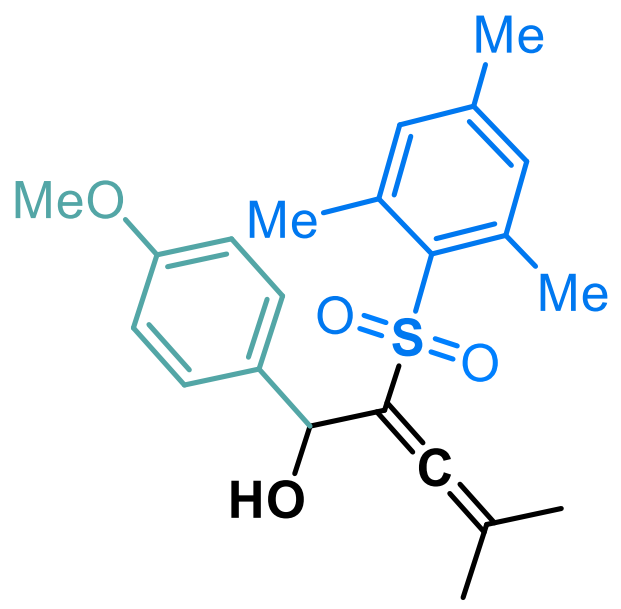

2ao

2-(mesitylsulfonyl)-1-(4-methoxyphenyl)-4-methylpenta-2,3-dien-1-ol(2ao): Prepared according to GP-2 on 1.79 mmol (500 mg)
White solid (700 mg, 78% yield)
Rf = 0.39 (25% EA/hexane)
Melting point = 131-133°C
NMR Spectroscopy:
^{1}H NMR (400 MHz, CHLOROFORM-D) δ 7.19 (d, *J* = 8.6 Hz, 2H), 6.92 (s, 2H), 6.80 (d, *J* = 8.8 Hz, 2H), 5.63 (d, *J* = 3.4 Hz, 1H), 3.78 (s, 3H), 3.36 (d, *J* = 3.3 Hz, 1H), 2.61 (s, 6H), 2.29 (s, 3H), 1.52 (s, 3H), 1.40 (s, 3H) ppm.
^{13}C NMR (101 MHz, CHLOROFORM-D) δ 200.3, 159.1, 143.3, 140.3, 133.1, 132.8, 131.9, 127.4, 115.6, 113.4, 108.8, 69.9, 55.3, 22.8, 21.1, 19.2, 19.0 ppm.
HRMS (ESI) m/z: [M+Na]+ Calcd for $C_{22}H_{26}NaO_4S^+$ 409.1449; Found 409.1442.

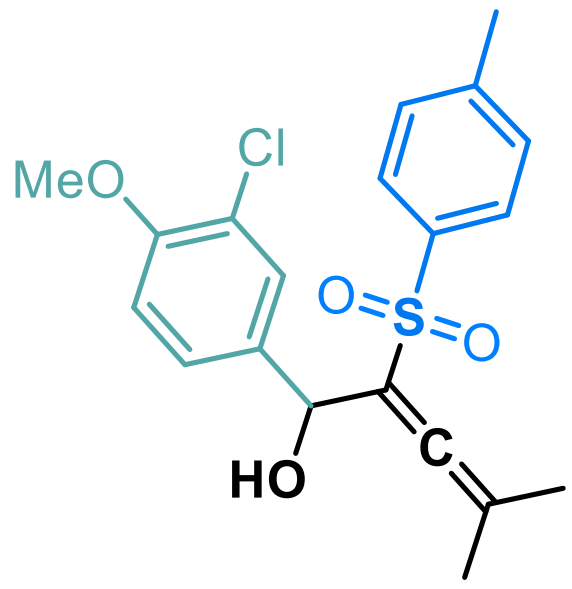

2ap

1-(3-chloro-4-methoxyphenyl)-4-methyl-2-tosylpenta-2,3-dien-1-ol (2ap): Prepared according to GP-2 on 2.93 mmol (500 mg)

Pale Yellow solid (800 mg, 70% yield)

Rf = 0.37 (25% EA/hexane)

Melting point = 86-88°C

NMR Spectroscopy:

^{1}H NMR (400 MHz, CHLOROFORM-D) δ 7.60 (d, J = 5.5 Hz, 2H), 7.22 (dd, J = 4.4, 3.5 Hz, 2H), 7.10 (t, J = 1.7 Hz, 1H), 7.06 (dt, J = 8.4, 2.4 Hz, 1H), 6.75 (dd, J = 8.5, 2.5 Hz, 1H), 5.49 (s, 1H), 3.82 (d, J = 3.0 Hz, 3H), 3.53 (s, 1H), 2.38 (d, J = 2.2 Hz, 3H), 1.66 (d, J = 1.6 Hz, 3H), 1.56 (d, J = 1.7 Hz, 3H) ppm.

^{13}C NMR (101 MHz, CHLOROFORM-D) δ 202.9, 154.5, 144.4, 137.9, 133.4, 129.7, 128.3, 127.7, 125.8, 121.9, 114.4, 111.4, 108.8, 70.3, 56.2, 56.2, 21.7, 19.3, 19.2 ppm.

HRMS (ESI) m/z: [M+Na]+ Calcd for $C_{20}H_{21}ClNaO_4S^+$ 415.0747; Found 415.0741.

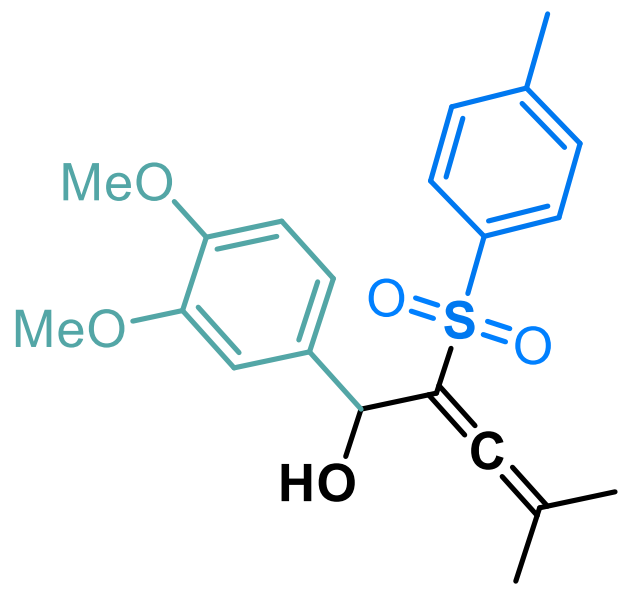

2aq

1-(3,4-dimethoxyphenyl)-4-methyl-2-tosylpenta-2,3-dien-1-ol (2aq): Prepared according to GP-2 on 1.79 mmol (500 mg)

White solid (700 mg, 78% yield)

Rf = 0.41 (25% EA/hexane)

Melting point =143-145 °C

NMR Spectroscopy:

^{1}H NMR (400 MHz, CHLOROFORM-D) δ 7.55 (s, 2H), 7.40 (s, 2H), 7.36 – 7.29 (m, 2H), 7.18 (dd, J = 7.8, 3.2 Hz, 1H), 7.11 – 7.01 (m, 3H), 6.85 (s, 2H), 5.86 (s, 1H), 3.72 (s, 3H), 3.32 (s, 1H), 3.02 (s, 6H), 2.31 (s, 3H), 1.74 (s, 3H), 1.60 (s, 3H) ppm.

^{13}C NMR (101 MHz, CHLOROFORM-D) δ 202.9, 149.4, 143.7, 138.2, 136.3, 132.6, 129.2, 127.9, 127.8, 127.4, 126.2, 121.0, 116.8, 114.0, 113.8, 113.2, 109.5, 108.3, 66.6, 41.0, 32.9, 21.7, 19.7, 19.5 ppm.

HRMS (ESI) m/z: [M+H]+ Calcd for $C_{21}H_{25}O_5S^+$ 389.1423; Found 389.1429.

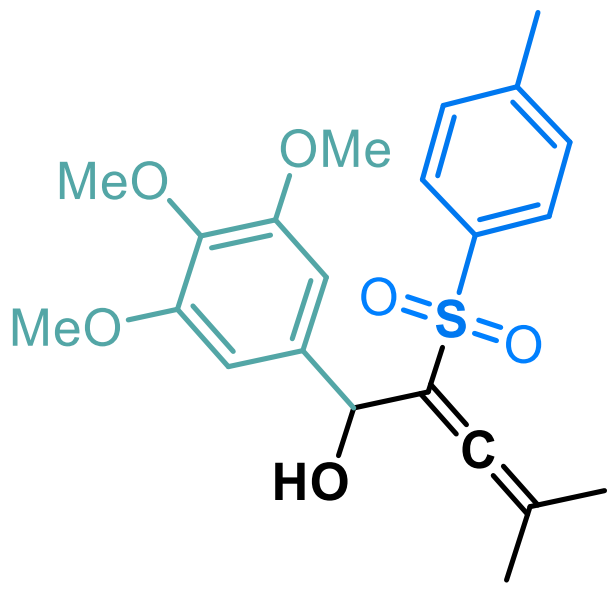

2ar

4-methyl-2-tosyl-1-(3,4,5-trimethoxyphenyl)penta-2,3-dien-1-ol (2ar): Prepared according to GP-2 on 2.55 mmol (500 mg)

White solid (700 mg, 78% yield)

Rf = 0.39 (25% EA/hexane)

Melting point = 118-120°C

NMR Spectroscopy:

^{1}H NMR (400 MHz, CHLOROFORM-D) δ 7.59 (d, *J* = 8.3 Hz, 2H), 7.45 – 7.14 (m, 2H), 6.41 (s, 2H), 5.55 (d, *J* = 5.0 Hz, 1H), 3.77 (s, 3H), 3.72 (s, 6H), 3.44 (d, *J* = 5.0 Hz, 1H), 2.39 (s, 3H), 1.68 (s, 3H), 1.60 (s, 3H) ppm.

^{13}C NMR (101 MHz, CHLOROFORM-D) δ 203.4, 159.4, 139.9, 139.8, 132.1, 129.2, 129.2, 127.7, 114.5, 113., 108.9, 77.1, 77.1, 71.1, 55.4, 19.4, 19.3 ppm.

HRMS (ESI) m/z: [M+H]+ Calcd for $C_{22}H_{27}O_6S^+$ 419.1528; Found 419.1519.

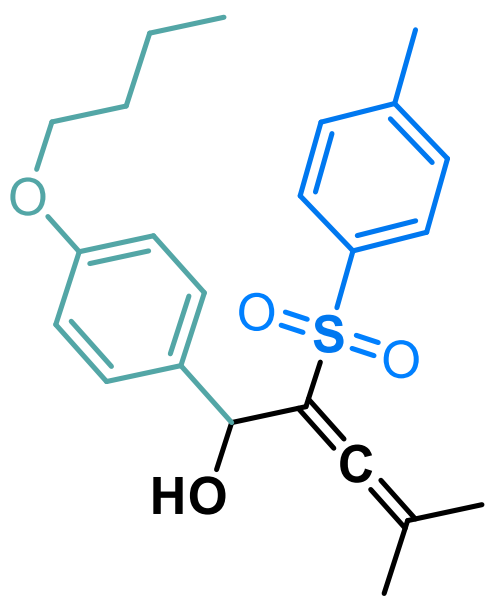

2as

1-(4-butoxyphenyl)-4-methyl-2-tosylpenta-2,3-dien-1-ol (2as): Prepared according to GP-2 on 2.80 mmol (500 mg)

White Solid (700 mg, 78% yield)

Melting point = 85-87°C

Rf = 0.38 (25% EA/hexane)

NMR Spectroscopy:

^{1}H NMR (400 MHz, CHLOROFORM-D) δ 7.65 (d, *J* = 8.3 Hz, 2H), 7.31 – 7.22 (m, 2H), 7.10 (d, *J* = 8.7 Hz, 2H), 6.82 – 6.70 (m, 2H), 5.55 (s, 1H), 3.91 (t, *J* = 6.6 Hz, 2H), 3.31 (s, 1H), 2.42 (s, 3H), 1.80 – 1.70 (m, 2H), 1.67 (s, 3H), 1.56 (s, 3H), 1.48 (dd, *J* = 15.1, 7.5 Hz, 2H), 0.97 (t, *J* = 7.4 Hz, 3H) ppm.

^{13}C NMR (101 MHz, CHLOROFORM-D) δ 202.9, 149.4, 143.7, 138.2, 136.3, 132.6, 129.2, 127.9, 127.8, 127.4, 126.2, 121.0, 116.8, 114.0, 113.8, 113.2, 109.5, 108.3, 66.6, 41.0, 32.9, 21.7, 19.7, 19.5 ppm.
HRMS (ESI) m/z: [M+H]+ Calcd for $C_{23}H_{29}O_4S^+$ 401.1786; Found 401.1781.

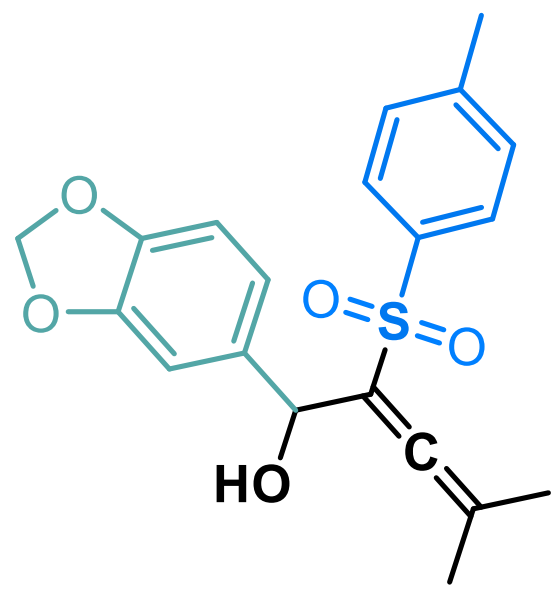

2at

1-(benzo[d][1,3]dioxol-5-yl)-4-methyl-2-tosylpenta-2,3-dien-1-ol (2at): Prepared according to GP-2 on 3.33 mmol (500 mg)
Pale Yellow solid (450 mg, 36% yield)
Rf = 0.32 (25% EA/hexane)
Melting point = 84-86°C
NMR Spectroscopy:
^{1}H NMR (400 MHz, CHLOROFORM-D) δ 7.62 (d, *J* = 8.3 Hz, 2H), 7.23 (d, *J* = 8.5 Hz, 2H), 6.88 – 6.39 (m, 3H), 5.87 (d, *J* = 1.1 Hz, 2H), 5.48 (d, *J* = 4.5 Hz, 1H), 3.49 (d, *J* = 4.5 Hz, 1H), 2.39 (s, 3H), 1.67 (s, 3H), 1.58 (s, 3H) ppm.
^{13}C NMR (101 MHz, CHLOROFORM-D) δ 202.9, 147.4, 147.1, 144.2, 138.0, 134.3, 129.6, 127.7, 120.0, 114.7, 108.7, 107.8, 106.9, 101.0, 70.8, 21.7, 19.3, 19.2 ppm.
HRMS (ESI) m/z: [M+Na]+ Calcd for $C_{20}H_{20}NaO_5S^+$ 395.0929; Found 395.0923.

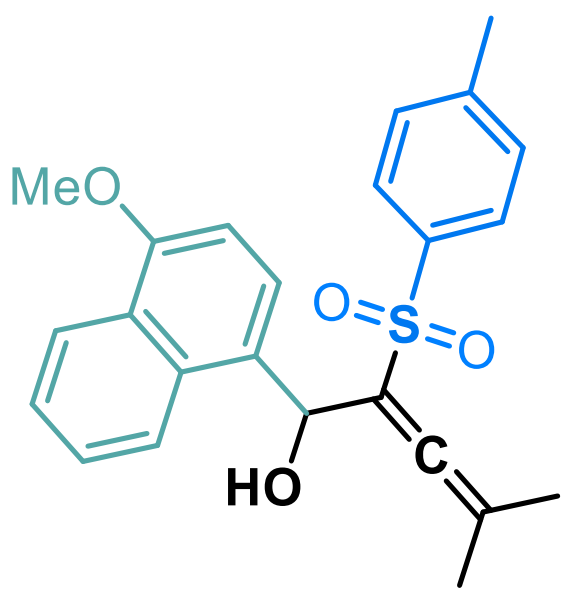

2au

1-(4-methoxynaphthalen-1-yl)-4-methyl-2-tosylpenta-2,3-dien-1-ol (2au): Prepared according to GP-2 on 2.68 mmol (500 mg)
Orange solid (1000 mg, 91% yield)
Rf = 0.34 (25% EA/hexane)
Melting point = 88-90°C
NMR Spectroscopy:

^{1}**H NMR (400 MHz, CHLOROFORM-D)** δ 8.17 (d, *J* = 8.2 Hz, 1H), 7.67 (d, *J* = 8.2 Hz, 2H), 7.57 (d, *J* = 8.1 Hz, 1H), 7.37 – 7.29 (m, 2H), 7.22 (t, *J* = 7.8 Hz, 3H), 6.72 (d, *J* = 8.1 Hz, 1H), 6.13 (s, 1H), 3.97 (s, 3H), 3.70 (s, 1H), 2.42 (s, 3H), 1.50 (s, 3H), 1.11 (s, 3H) ppm.
^{13}**C NMR (101 MHz, CHLOROFORM-D)** δ 203.5, 155., 144.2, 137.6, 130.8, 129.6, 128.3, 127.7, 127.0, 126.1, 125.5, 124.6, 124.3, 122.8, 122.5, 113.8, 108.1, 102.9, 69.1, 55.5, 21.7, 18.9, 18.4 ppm.
HRMS (ESI) m/z: [M+Na]+ Calcd for $C_{24}H_{24}NaO_4S^+$ 431.1293; Found 431.1291.

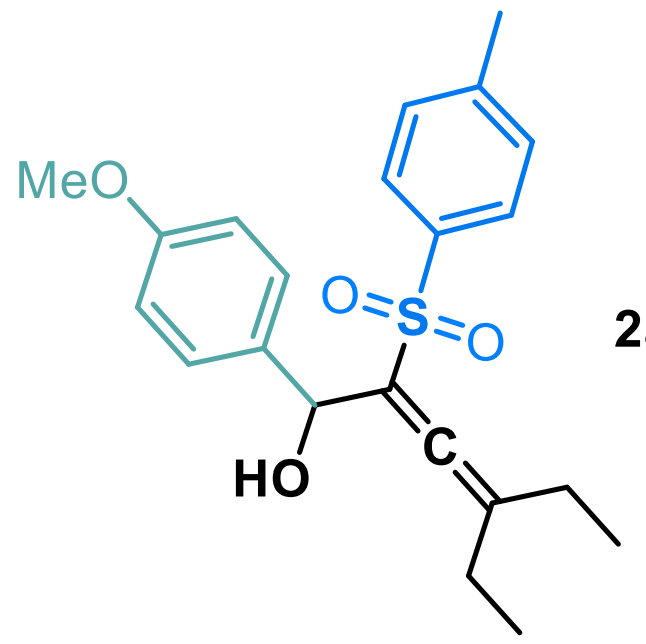

2av

4-ethyl-1-(4-methoxyphenyl)-2-tosylhexa-2,3-dien-1-ol (2av): Prepared according to previous literature protocol on 1.92 mmol (300 mg)
Yellow solid (500 mg, 58% yield)
Rf = 0.39 (25% EA/hexane)
Melting point = 85-87°C
NMR Spectroscopy:
^{1}**H NMR (400 MHz, CHLOROFORM-D)** δ 7.72 (d, *J* = 8.3 Hz, 2H), 7.29 (d, *J* = 7.9 Hz, 2H), 7.15 (d, *J* = 8.6 Hz, 2H), 6.77 (d, *J* = 8.7 Hz, 2H), 5.62 (s, 1H), 3.76 (s, 3H), 2.43 (s, 3H), 1.97 – 1.71 (m, 4H), 0.90 (t, *J* = 7.3 Hz, 3H), 0.64 (t, *J* = 7.5 Hz, 3H) ppm.
^{13}**C NMR (101 MHz, CHLOROFORM-D)** δ 202.3, 159.2, 144.3, 138.0, 132.4, 129.6, 128.0, 127.6, 121.4, 119.0, 113.4, 70.9, 55.3, 25.6, 25.5, 21.7, 11.7, 11.5 ppm.
HRMS (ESI) m/z: [M+Na]+ Calcd for $C_{22}H_{26}NaO_4S^+$ 409.1449; Found 409.1447.

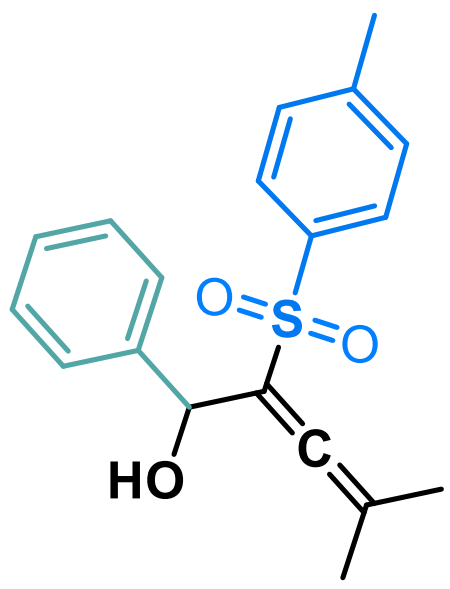

2aw

4-methyl-1-phenyl-2-tosylpenta-2,3-dien-1-ol (2aw): Prepared according to GP-2 on 2.55 mmol (500 mg)

Pale Yellow solid (700 mg, 78% yield). The 1H NMR and 13C NMR spectra matched the previous report's data.[7]

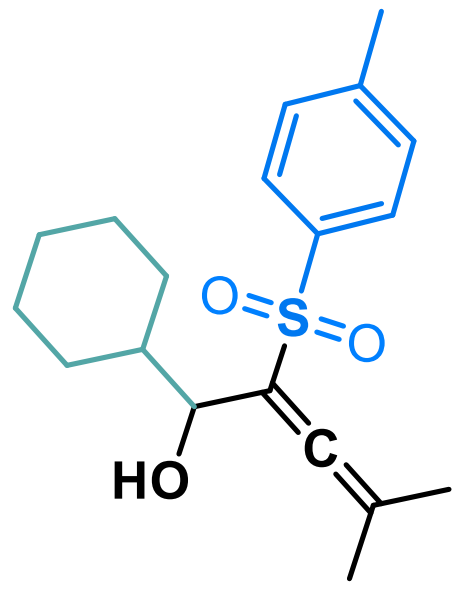

2ax

1-cyclohexyl-4-methyl-2-tosylpenta-2,3-dien-1-ol (2ax): Prepared according to GP-2 on 1.78 mmol (200 mg)

Pale Yellow solid (700 mg, 78% yield)

Rf = 0.45 (25% EA/hexane)

Melting point = 59-61°C

NMR Spectroscopy:

^{1}H NMR (400 MHz, CHLOROFORM-D) δ 7.84 – 7.63 (m, 2H), 7.32 (d, *J* = 8.1 Hz, 2H), 4.07 (d, *J* = 8.1 Hz, 1H), 2.69 (s, 1H), 2.44 (s, 3H), 1.96 (d, *J* = 12.9 Hz, 1H), 1.74 (d, *J* = 6.1 Hz, 6H), 1.56 (ddd, *J* = 45.5, 23.5, 18.3 Hz, 5H), 1.13 (dd, *J* = 18.5, 6.2 Hz, 3H), 0.96 (ddd, *J* = 24.0, 12.3, 3.4 Hz, 1H), 0.84 (qd, *J* = 12.3, 3.3 Hz, 1H) ppm.

^{13}C NMR (101 MHz, CHLOROFORM-D) δ 203.4, 159.4, 139.9, 139.8, 132.1, 129.2, 129.2, 127.7, 114.5, 113., 108.9, 77.1, 77.1, 71.1, 55.4, 19.4, 19.3 ppm.

HRMS (ESI) m/z: [M+H]+ Calcd for $C_{19}H_{27}O_3S^+$ 335.1680; Found 335.1690.

2.3 Detailed optimization studies

A Teflon-capped clear glass screw vial equipped with a magnetic stir bar was charged with 3-1H-indole (**1a**, 8.5 mg, 0.07 mmol, 1.3 equiv), the metal catalyst (20 mol%), and then substrate **2a** (20 mg, 0.05 mmol, 1equiv). Subsequently, 0.2 ml of dry solvent was added to the reaction mixture, following which the vial was sealed. The reaction was stirred for 48 h at 80 °C with the help of an aluminium heating block. After completion, the reaction mixture was concentrated in vacuo. The reaction mixture was filtered through Celite, extracted with dichloromethane, and dried over anhydrous sodium sulfate. Removal of the solvent under vacuum afforded the crude product, which was purified by column chromatography to afford **3a,** with the isolated yield calculated accordingly. Initial investigations focused on the identification of an effective catalyst (Entries 1–8). When the reaction was conducted in 1,2-dichloroethane (DCE) at 80 °C for 18 h, Brønsted acids such as *p*-TsOH·H_2O afforded the desired product in 72% yield (Entry 1), whereas the superacid TfOH resulted in less than 5% yield (Entry 4), likely due to rapid substrate degradation or catalyst deactivation. Lewis acids exhibited variable performance: $ZnCl_2$ delivered 50% yield (Entry 3), and $Sc(OTf)_3$ provided 58% yield (Entry 5), while $AlCl_3$ failed to promote the reaction, giving no detectable product

(Entry 2). Iron-based catalysts showed improved reactivity. $Fe(acac)_3$ furnished product 3aa in 65% yield (Entry 6), whereas $Fe(OTf)_3$ resulted in a diminished 45% yield (Entry 7). Notably, $FeCl_3$ proved to be the most efficient catalyst, delivering 3aa in 88% yield (Entry 8). The influence of $FeCl_3$ loading on reaction efficiency was subsequently investigated (Entries 9–13). When the catalyst loading was reduced to 5mol%, the yield of 3aa decreased to 72% (Entry 9), while a marginal improvement to 73% yield was observed at 10 mol% (Entry 10). Increasing the $FeCl_3$ concentration to 15 mol% further enhanced the yield to 76% (Entry 11). Optimal performance was achieved using 20 mol% $FeCl_3$, delivering the desired product in 88% yield (Entry 12). However, increasing the catalyst loading to 50 mol% led to a diminished yield of 80% (Entry 13). Temperature was found to exert a pronounced effect on the reaction outcome (Entries 20–23). At ambient temperature (25 °C), no conversion was observed. Increasing the temperature to 40 °C resulted in only 20% yield, while further elevation to 50 °C and 60 °C improved the yield to 55% and 65%, respectively. The maximum yield was obtained when the reaction vial was kept at 80 °C.

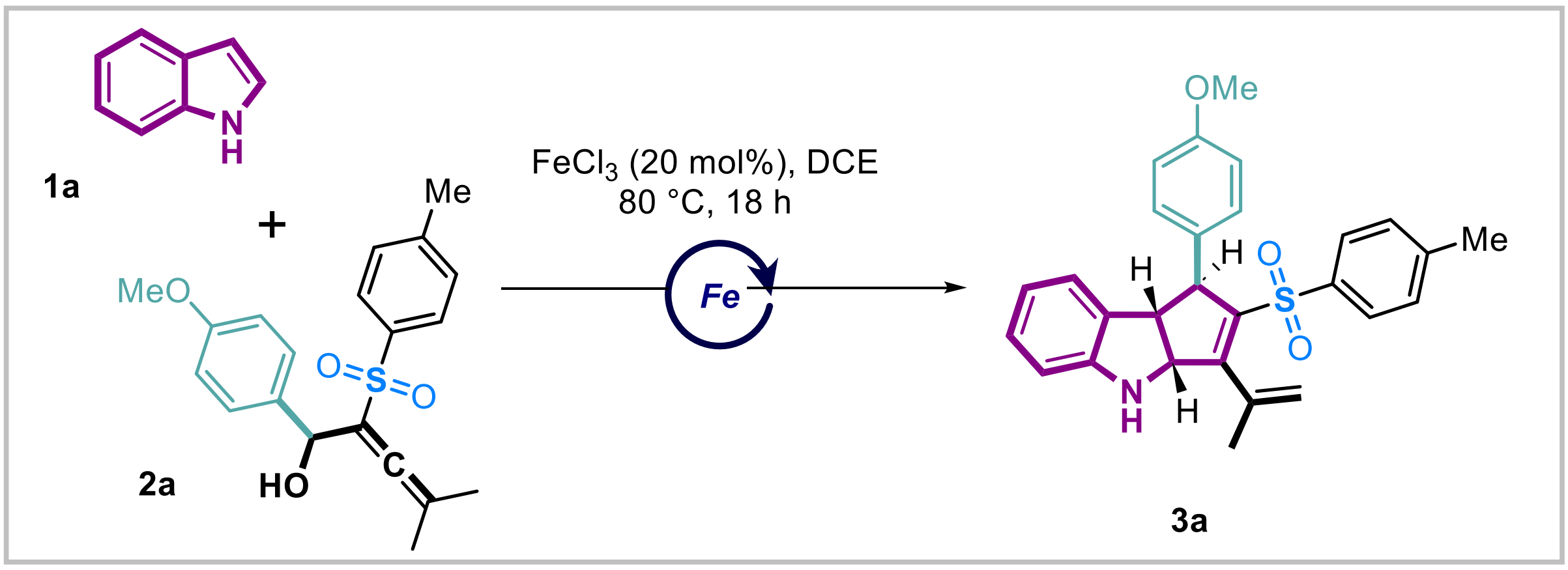

Entry No.	Catalyst	Solvent	Temperature	Time	Yield of 3a[a]
		Screening of Catalyst			
**1.**	p-TsOH.H_2O	DCE	80	18	72%
**2.**	$AlCl_3$	DCE	80	18	n.d.
**3.**	$ZnCl_2$	DCE	80	18	50%
**4.**	TfOH	DCE	80	18	<5%
**5.**	$Sc(OTf)_3$	DCE	80	18	58%
**6.**	$Fe(acac)_3$	DCE	80	18	65%
**7.**	$Fe(OTf)_3$	DCE	80	18	45%
**8.**	$FeCl_3$	DCE	80	18	88%
		Screening of mol% of $FeCl_3$			
**9.**	5 mol% $FeCl_3$	DCE	80	18	72%
**10.**	10 mol% $FeCl_3$	DCE	80	18	73%
**11.**	15 mol% $FeCl_3$	DCE	80	18	76%
**12.**	**20 mol% $FeCl_3$**	**DCE**	**80**	**18**	**88%**
**13.**	50 mol% $FeCl_3$	DCE	80	18	80%

		**Screening of Solvents**			
**14.**	$FeCl_3$	MeCN	80	18	50%
**15.**	$FeCl_3$	DMSO	80	18	traces
**16.**	$FeCl_3$	DCM	80	18	20%
**17.**	$FeCl_3$	Xylene	80	18	50%
**18.**	$FeCl_3$	Toluene	80	18	70%
**19..**	$FeCl_3$	THF	80	18	<5%
		**Screening of Temperature**			
**20.**	$FeCl_3$	DCE	25	18	n.d.
**21.**	$FeCl_3$	DCE	40	18	20%
**22.**	$FeCl_3$	DCE	50	18	55%
**23.**	$FeCl_3$	DCE	60	18	65%
		**Screening of Time**			
**24.**	$FeCl_3$	DCE	80	12	60%
**25.**	$FeCl_3$	DCE	80	24	88%
		**Screening of Additives**			
**26.**	$FeCl_3$ + AcOH	DCE	80	18	55%
**27.**	$FeCl_3$ + K_2CO_3	DCE	80	18	20%
**28.**	$FeCl_3$ + Na_2CO_3	DCE	80	18	24%
**29.**	$FeCl_3$ + NaOAc	DCE	80	18	32%

2.4 General procedure for Dearomative Formal [3+2] Cycloaddition Reactions (GP3)

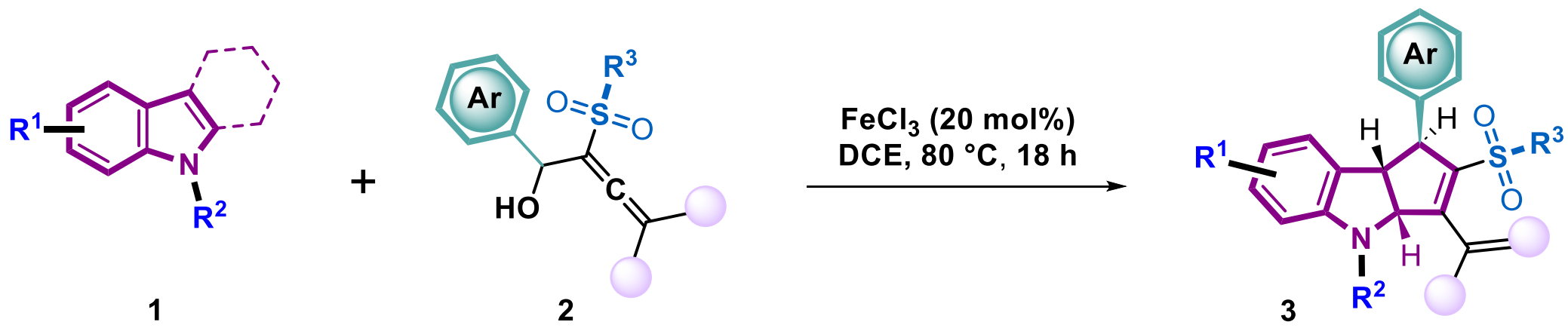

1 2 3

Substrate **2a-2ax** was synthesized in accordance with **GP3**. In the following step, a clear glass screw vial with a Teflon cap, containing a magnetic stir bar, was charged with the indole substrates **1a-1ae** (0.39 mmol, 1.3 equiv), the catalyst $FeCl_3$ (20 mol %), and appropriate allenol substrates **2a-2ay** (0.3 mmol, 1.0 equiv). Subsequently, 3 ml of dry DCE was added to the reaction mixture, following which the vial was sealed. The reaction was stirred for 18 h at 80 °C with the help of an aluminium heating block. The reaction mixture was filtered through celite, extracted with dichloromethane, and dried over anhydrous sodium sulfate. Removal of the solvent under vacuum afforded the crude product, which was purified by column chromatography to afford **3a-3ay**.

Note: Most of the reaction forms a single diastereomer in the reaction. In the case of fused indole **1t**, **1u**, and **1v** give a mixture of diastereomers.

a) 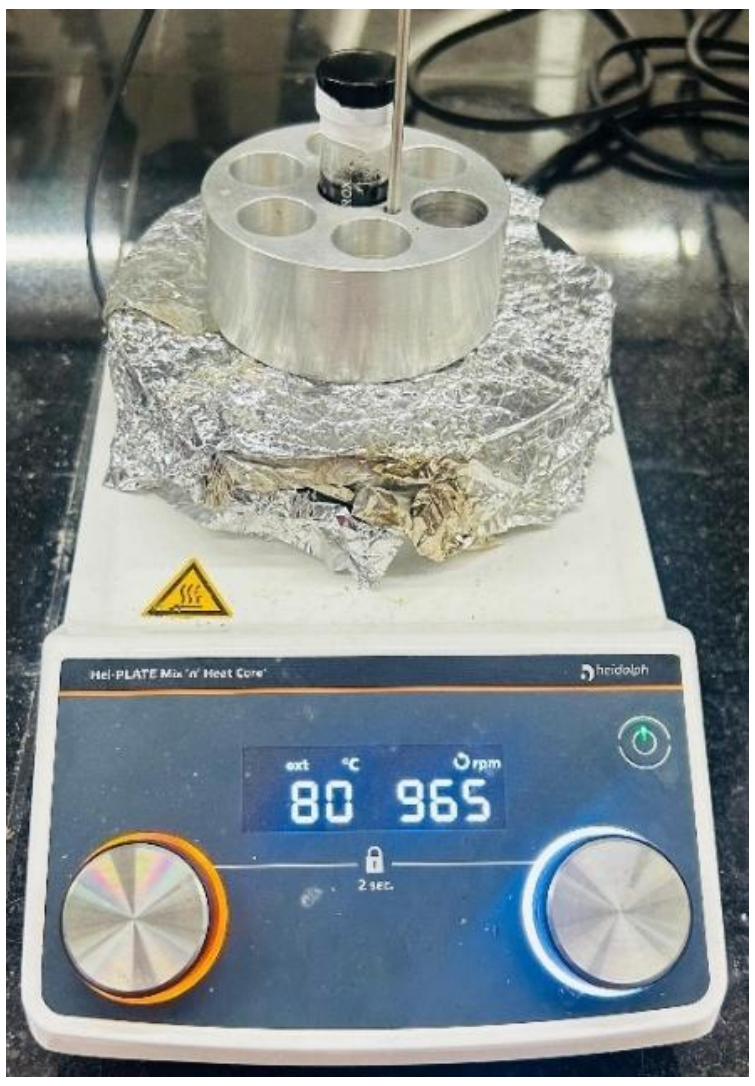

b) 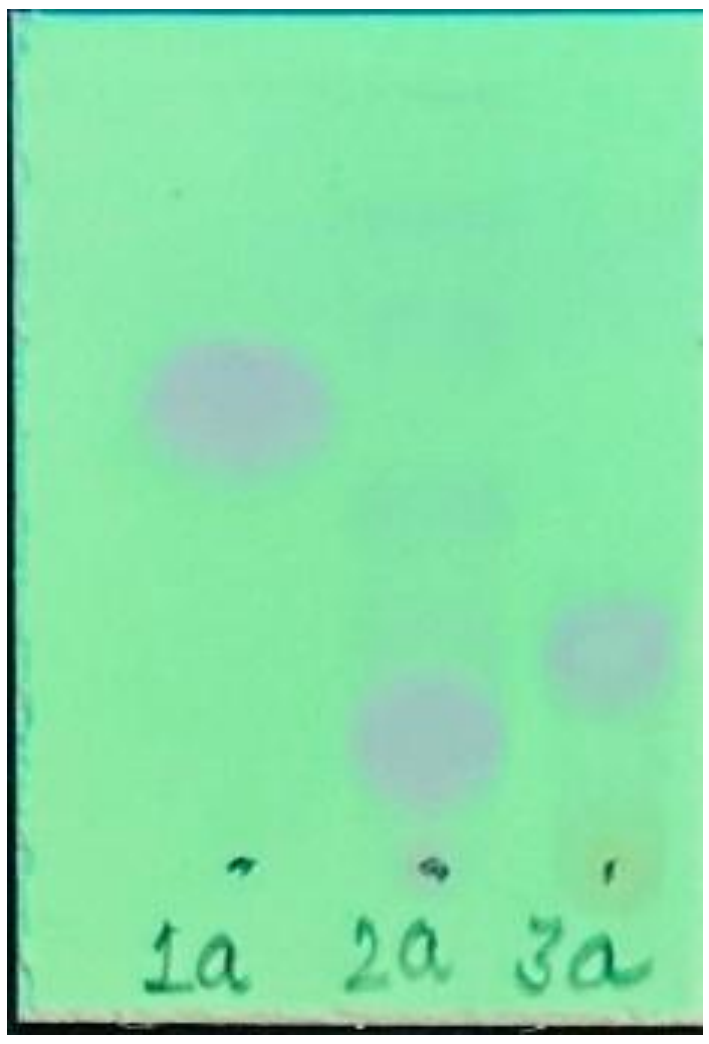

Figure S1: *a) Reaction setup carried out at 80 °C in the aluminium heating block with glass screw vial. b) Thin layer chromatography spot of substrate **1a**, **2a** and product **3a** in 30 % EtOAc/Hexanes.*

Spectral data for final products

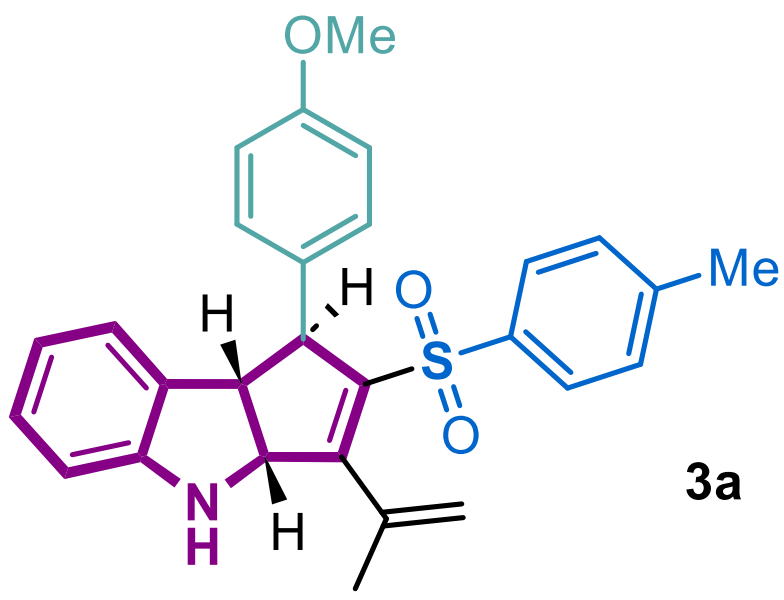

3a

(1S)-1-(4-methoxyphenyl)-3-(prop-1-en-2-yl)-2-tosyl-1,3a,4,8b-tetrahydrocyclopenta[b]indole (**3a**): Prepared according to GP-3 on 0.3 mmol (110 mg).
Off-white solid (119 mg, 85% yield)
Rf = 0.41 (30% EA/hexane)
Melting point =265-267 °C
NMR Spectroscopy:
^{1}H NMR (400 MHz, CHLOROFORM-D) δ 7.19 (d, *J* = 8.3 Hz, 3H), 7.11 – 6.96 (m, 5H), 6.80 (d, *J* = 8.7 Hz, 2H), 6.64 (d, *J* = 7.8 Hz, 1H), 5.27 (t, *J* = 1.6 Hz, 1H), 4.99 (dd, *J* = 7.7, 1.8 Hz, 1H), 4.83 (s, 1H), 4.44 (s, 1H), 4.14 (s, 1H), 3.82 (s, 3H), 3.77 (d, *J* = 7.6 Hz, 1H), 2.33 (s, 3H), 1.88 (s, 3H) ppm.
^{13}C NMR (101 MHz, CHLOROFORM-D) δ 158.7, 154.5, 148.4, 143.8, 141.8, 138.2, 137.3, 134.0, 130.6, 129.0, 128.7, 128.6, 127.9, 124.6, 119.8, 118.0, 114.2, 110.4, 70.5, 59.4, 55.5, 53.9, 22.5, 21.6ppm.

HRMS (ESI) m/z: [M+H]+ Calcd for $C_{28}H_{27}NO_3S^+$ 458.1784; Found 458.1797.

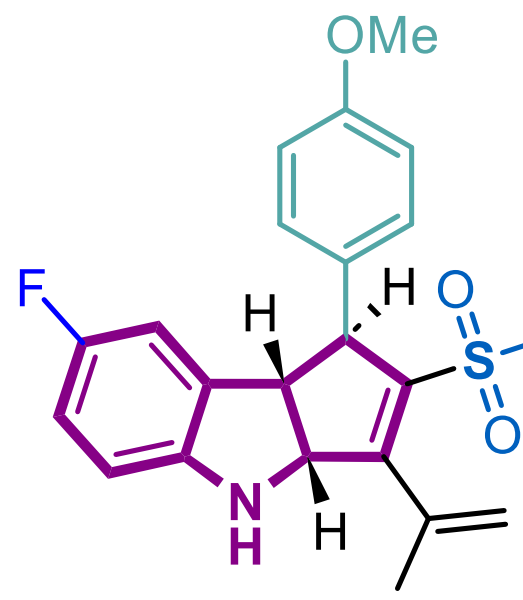

3b

(1S)-7-fluoro-1-(4-methoxyphenyl)-3-(prop-1-en-2-yl)-2-tosyl-1,3a,4,8b-tetrahydrocyclopenta[b]indole (**3b**): Prepared according to GP-3 on 0.3 mmol (110 mg)

Brown solid (100 mg, 71% yield)

Rf = 0.38 (30% EA/hexane)

Melting point = 149-151°C

NMR Spectroscopy:

^{1}H NMR (400 MHz, CHLOROFORM-D) δ 7.21 (d, *J* = 8.3 Hz, 2H), 7.01 (d, *J* = 8.8 Hz, 4H), 6.88 (ddd, *J* = 8.3, 2.5, 1.0 Hz, 1H), 6.80 (d, *J* = 8.7 Hz, 2H), 6.78 – 6.74 (m, 1H), 5.28 (t, *J* = 1.6 Hz, 1H), 4.99 (dd, *J* = 7.6, 1.8 Hz, 1H), 4.87 – 4.79 (m, 1H), 4.35 (s, 1H), 4.02 (s, 1H), 3.81 (s, 3H), 3.74 (d, *J* = 7.6 Hz, 1H), 2.34 (s, 3H), 1.89 (s, 3H) ppm.

^{13}C NMR (101 MHz, CHLOROFORM-D) δ 158.8, 158.8-156.4 (d, *J* = 237.1 Hz), 154.3, 144.4, 144.0, 141.8, 137.9, 137.3, 133.5, 132.3-132.3 (d, *J* = 7.7 Hz), 129.0, 128.6, 127.9, 118.0, 115.1-114.8 (d, *J* = 23.6 Hz), 114.3, 112.0-111.7 (d, *J* = 24.0 Hz), 110.9-110.8 (d, *J* = 8.3 Hz), 71.0, 59.1, 55.4, 54.2, 22.6, 21.6. ppm.

^{19}F NMR (376 MHz, CHLOROFORM-D) δ = -124.77 ppm.

HRMS (ESI) m/z: [M+H]+ Calcd for $C_{28}H_{26}FNO_3S^+$ 476.1695; Found 476.1690.

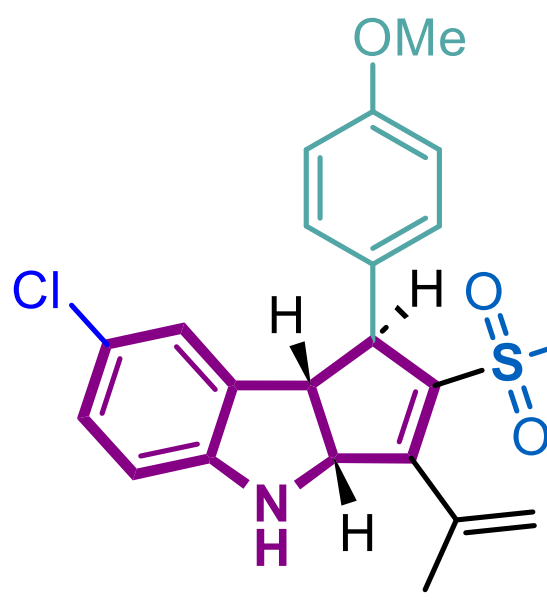

3c

(1S)-7-chloro-1-(4-methoxyphenyl)-3-(prop-1-en-2-yl)-2-tosyl-1,3a,4,8b-tetrahydrocyclopenta[b]indole (**3c**): Prepared according to GP-3 on 0.3 mmol (110 mg)

Brown solid (127 mg, 87% yield)

Rf = 0.39 (30% EA/hexane)

Melting point = 201-203°C

NMR Spectroscopy:

^{1}H NMR (400 MHz, CHLOROFORM-D) δ 7.21 (d, *J* = 8.4 Hz, 2H), 7.10 (d, *J* = 1.2 Hz, 1H), 7.04 – 6.96 (m, 5H), 6.79 (d, *J* = 8.7 Hz, 2H), 6.54 (d, *J* = 8.3 Hz, 1H), 5.28 – 5.26 (m, 1H), 4.98 (dd, *J* =

7.7, 1.8 Hz, 1H), 4.81 (s, 1H), 4.34 (s, 1H), 4.16 (s, 1H), 3.81 (s, 3H), 3.76 – 3.70 (m, 1H), 2.34 (s, 3H), 1.89 (s, 3H) ppm.

^{13}C NMR (101 MHz, CHLOROFORM-D) δ 158.8, 154.1, 147.1, 144.1, 141.7, 137.8, 137.2, 133.4, 132.5, 129.1, 128.6, 128.5, 127.9, 124.7, 124.2, 118.0, 114.3, 111.1, 70.8, 59.0, 55.5, 55.4, 53.8, 22.6, 21.6 ppm.

HRMS (ESI) m/z: [M+H]+ Calcd for $C_{28}H_{27}ClNO_3S^+$ 492.1400; Found 492.1411.

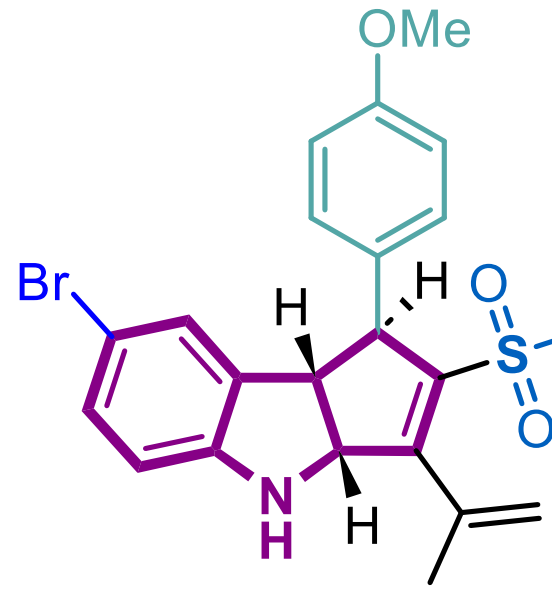

3d

(1S)-7-bromo-1-(4-methoxyphenyl)-3-(prop-1-en-2-yl)-2-tosyl-1,3a,4,8b-tetrahydrocyclopenta[b]indole (**3d**): Prepared according to GP-3 on 0.3 mmol (110 mg)

Brown solid (116 mg, 70% yield)

Rf = 0.37 (30% EA/hexane)

Melting point = 198-200°C

NMR Spectroscopy:

^{1}H NMR (400 MHz, CHLOROFORM-D) δ 7.25 – 7.19 (m, 3H), 7.17 – 7.13 (m, 1H), 7.06 – 6.97 (m, 4H), 6.83 – 6.77 (m, 2H), 6.50 (d, *J* = 8.3 Hz, 1H), 5.28 (t, *J* = 1.4 Hz, 1H), 4.97 (dd, *J* = 7.6, 1.7 Hz, 1H), 4.81 (s, 1H), 4.33 (s, 1H), 4.15 (s, 1H), 3.81 (s, 3H), 3.73 (d, *J* = 7.8 Hz, 1H), 2.34 (d, *J* = 8.3 Hz, 3H), 1.89 (d, *J* = 6.8 Hz, 3H) ppm.

^{13}C NMR (101 MHz, CHLOROFORM-D) δ 158.8, 154.0, 147.6, 144.1, 141.8, 137.8, 137.2, 133.4, 132.9, 131.3, 129.1, 128.6, 127.9, 127.6, 118.0, 114.3, 114.2, 111.6, 111.2, 70.7, 59.0, 55.4, 55.4, 53.8, 22.6, 21.7ppm.

HRMS (ESI) m/z: [M+H]+ Calcd for $C_{28}H_{27}BrNO_3S^+$ 536.0895; Found 536.0912.

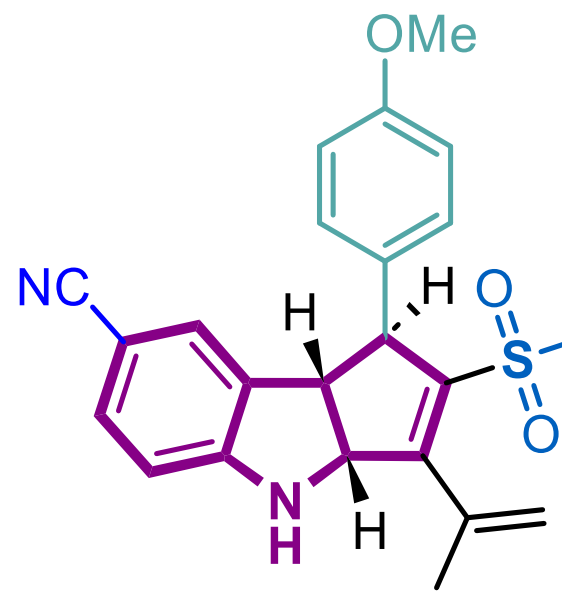

3e

(1S)-1-(4-methoxyphenyl)-3-(prop-1-en-2-yl)-2-tosyl-1,3a,4,8b-tetrahydrocyclopenta[b]indole-7-carbonitrile (**3e**): Prepared according to GP-3 on 0.3 mmol (110 mg)

Brown solid (60 mg, 42% yield)

Rf = 0.35 (30% EA/hexane)

Melting point = 199-201°C

NMR Spectroscopy:

^{1}H NMR (400 MHz, CHLOROFORM-D) δ 7.42 – 7.33 (m, 2H), 7.25 – 7.16 (m, 2H), 7.03 (d, *J* = 8.0 Hz, 2H), 7.02 – 6.94 (m, 2H), 6.85 – 6.75 (m, 2H), 6.60 (d, *J* = 8.4 Hz, 1H), 5.30 (t, *J* = 1.4 Hz, 1H), 5.08 (dd, *J* = 8.0, 1.6 Hz, 1H), 4.86 (s, 1H), 4.61 (s, 1H), 4.33 (s, 1H), 3.82 (s, 3H), 3.76 (d, *J* = 8.0 Hz, 1H), 2.35 (s, 3H), 1.92 (s, 3H) ppm.

^{13}C NMR (101 MHz, CHLOROFORM-D) δ 158.9, 153.2, 152.2, 144.3, 142.3, 137.6, 137.0, 134.1, 133.1, 131.2, 129.1, 128.6, 128.4, 127.9, 120.2, 118.2, 114.4, 109.4, 101.2, 77.2, 70.7, 59.4, 55.5, 53.0, 22.7, 21.7.

HRMS (ESI) m/z: [M+H]+ Calcd for $C_{29}H_{27}N_2O_3S^+$ 483.1737; Found 483.1748.

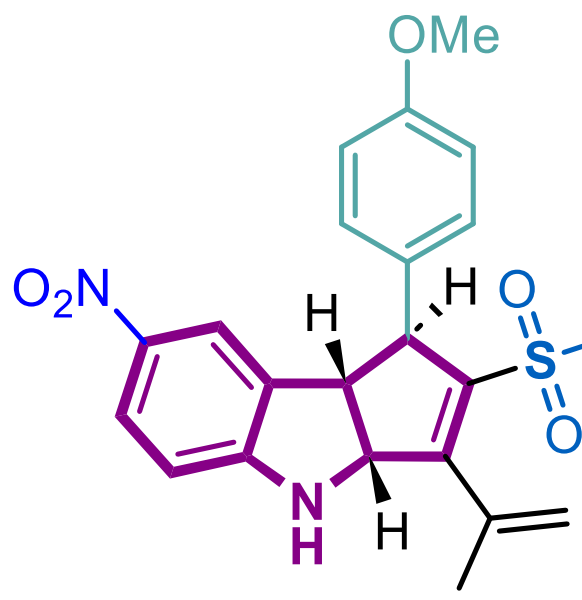

3f

(1S)-1-(4-methoxyphenyl)-7-nitro-3-(prop-1-en-2-yl)-2-tosyl-1,3a,4,8b-tetrahydrocyclopenta[b]indole (**3f**): Prepared according to GP-3 on 0.3 mmol (110 mg)

Yellow solid (90 mg, 59% yield)

Rf = 0.34 (30% EA/hexane)

Melting point = 173-176°C

NMR Spectroscopy:

^{1}H NMR (400 MHz, CHLOROFORM-D) δ 8.05 (dd, *J* = 11.6, 2.9 Hz, 2H), 7.22 (d, *J* = 8.2 Hz, 2H), 7.07 – 6.95 (m, 4H), 6.80 (d, *J* = 8.7 Hz, 2H), 6.56 (d, *J* = 8.5 Hz, 1H), 5.31 (s, 1H), 5.15 (d, *J* = 6.7 Hz, 1H), 4.87 (d, *J* = 11.1 Hz, 2H), 4.39 (s, 1H), 3.82 (s, 3H), 3.79 (s, 1H), 2.33 (s, 3H), 1.93 (s, 3H) ppm.

^{13}C NMR (101 MHz, CHLOROFORM-D) δ 159.0, 154.2, 152.8, 144.4, 142.7, 140.3, 137.5, 137.0, 133.0, 130.9, 129.2, 128.6, 128.1, 126.9, 121.3, 118.3, 114.4, 107.9, 77.2, 77.2, 71.3, 59.4, 55.5, 52.8, 22.7, 21.7 ppm.

HRMS (ESI) m/z: [M+H]+ Calcd for $C_{28}H_{27}N_2O_5S^+$ 503.1635; Found 503.1618.

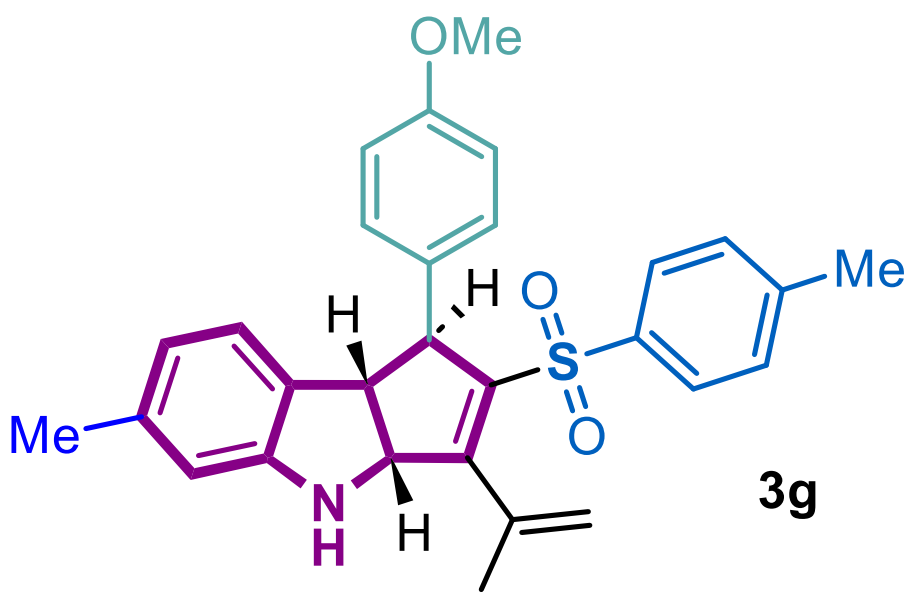

3g

(1S)-1-(4-methoxyphenyl)-6-methyl-3-(prop-1-en-2-yl)-2-tosyl-1,3a,4,8b-tetrahydrocyclopenta[b]indole (**3g**): Prepared according to GP-3 on 0.3 mmol (110 mg)
Brown thick liquid (102 mg, 72% yield)
Rf = 0.39 (30% EA/hexane)
NMR Spectroscopy:
^{1}H NMR (400 MHz, CHLOROFORM-D) δ 7.21 – 7.16 (m, 2H), 7.07 (d, *J* = 7.6 Hz, 1H), 7.01 (dd, *J* = 11.7, 5.0 Hz, 4H), 6.82 – 6.76 (m, 2H), 6.61 (d, *J* = 7.6 Hz, 1H), 6.47 (s, 1H), 5.27 (t, *J* = 1.5 Hz, 1H), 4.98 (dd, *J* = 7.6, 1.8 Hz, 1H), 4.85 (s, 1H), 4.41 (s, 1H), 4.06 (s, 1H), 3.81 (s, 3H), 3.73 (d, *J* = 7.7 Hz, 1H), 2.33 (s, 3H), 2.27 (s, 3H), 1.86 (s, 3H) ppm.
^{13}C NMR (101 MHz, CHLOROFORM-D) δ 158.7, 154.5, 148.6, 143.7, 141.8, 138.6, 138.2, 137.3, 134.1, 129.0, 128.7, 127.9, 127.8, 124.3, 120.7, 118.1, 114.2, 111.2, 70.7, 59.5, 55.5, 53.6, 22.5, 21.6 ppm.
HRMS (ESI) m/z: [M+H]+ Calcd for $C_{29}H_{30}NO_3S^+$ 472.1946; Found 472.1991.

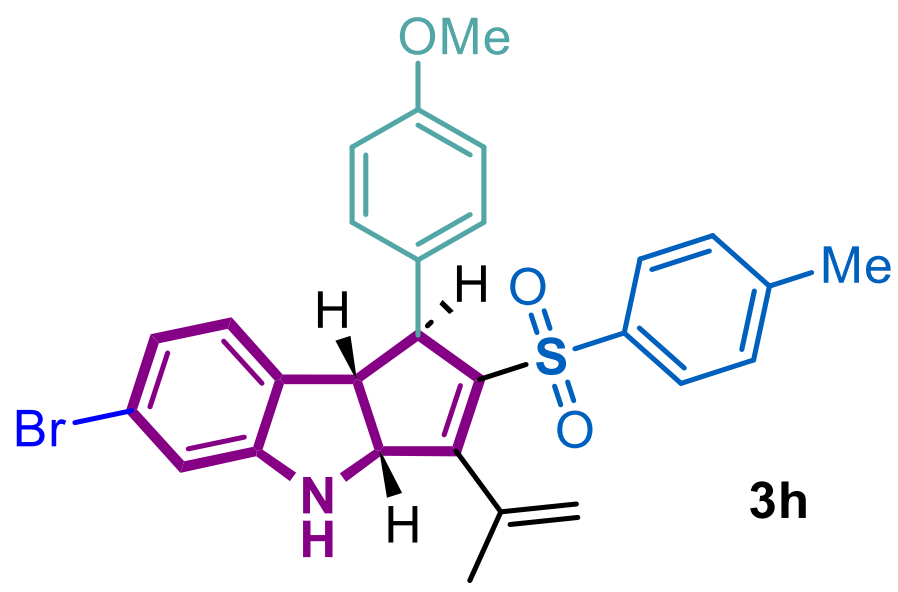

3h

(1S)-6-bromo-1-(4-methoxyphenyl)-3-(prop-1-en-2-yl)-2-tosyl-1,3a,4,8b-tetrahydrocyclopenta[b]indole (**3h**): Prepared according to GP-3 on 0.3 mmol (110 mg)
Brown solid (101 mg, 61% yield)
Rf = 0.37 (30% EA/hexane)
Melting point =191-201 °C
NMR Spectroscopy:
^{1}H NMR (400 MHz, CHLOROFORM-D) δ 7.21 (d, *J* = 8.2 Hz, 2H), 7.08 – 6.98 (m, 5H), 6.88 (dd, *J* = 7.9, 1.6 Hz, 1H), 6.77 (dd, *J* = 16.7, 5.0 Hz, 3H), 5.27 (s, 1H), 4.99 (dd, *J* = 7.7, 1.5 Hz, 1H), 4.82 (s, 1H), 4.37 (s, 1H), 4.19 (s, 1H), 3.81 (s, 3H), 3.69 (d, *J* = 7.7 Hz, 1H), 2.34 (s, 3H), 1.87 (s, 3H) ppm.
^{13}C NMR (101 MHz, CHLOROFORM-D) δ 158.8, 153.9, 149.9, 144.0, 142.1, 138.0, 137.1, 133.7, 129.7, 129.1, 128.7, 127.9, 125.8, 122.5, 122.0, 118.3, 114.3, 113.2, 70.7, 59.3, 55.5, 53.3, 22.5, 21.7 ppm.
HRMS (ESI) m/z: [M+H]+ Calcd for $C_{28}H_{27}BrNO_3S^+$ 536.0890; Found 536.0895.

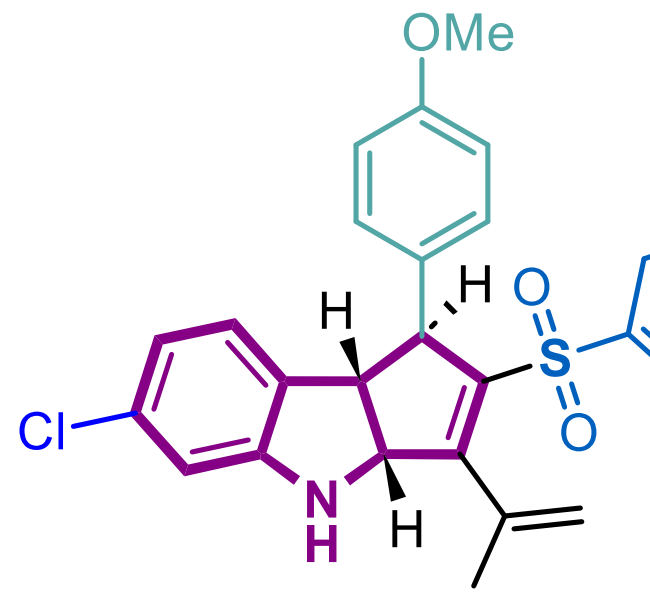

3i

(1S)-6-chloro-1-(4-methoxyphenyl)-3-(prop-1-en-2-yl)-2-tosyl-1,3a,4,8b-tetrahydrocyclopenta[b]indole (**3i**): Prepared according to GP-3 on 0.3 mmol (110 mg)
Brown solid (112 mg, 74% yield)
Rf = 0.38 (30% EA/hexane)
Melting point = 202-204°C
NMR Spectroscopy:
^{1}H NMR (400 MHz, CHLOROFORM-D) δ 7.21 (d, *J* = 8.3 Hz, 2H), 7.08 (d, *J* = 8.0 Hz, 1H), 7.01 (dd, *J* = 8.7, 2.6 Hz, 4H), 6.79 (d, *J* = 8.7 Hz, 2H), 6.74 (dd, *J* = 7.9, 1.8 Hz, 1H), 6.59 (d, *J* = 1.8 Hz, 1H), 5.35 – 5.19 (m, 1H), 5.01 (dd, *J* = 7.7, 1.6 Hz, 1H), 4.82 (s, 1H), 4.37 (s, 1H), 4.19 (s, 1H), 3.81 (s,3H), 3.71 (d, *J* = 7.7 Hz, 1H), 2.34 (s, 3H), 1.87 (s, 3H) ppm.
^{13}C NMR (101 MHz, CHLOROFORM-D) δ 158.8, 153.9, 149.6, 143.9, 142.0, 137.9, 137.1, 134.0, 133.6, 129.2, 129.0, 128.6, 127.9, 125.3, 119.6, 118.2, 114.2, 110.3, 70.8, 59.3, 55.4, 53.2, 22.5, 21.6 ppm.
HRMS (ESI) m/z: [M+H]+ Calcd for $C_{28}H_{27}ClNO_3S^+$ 492.1395; Found 492.1385.

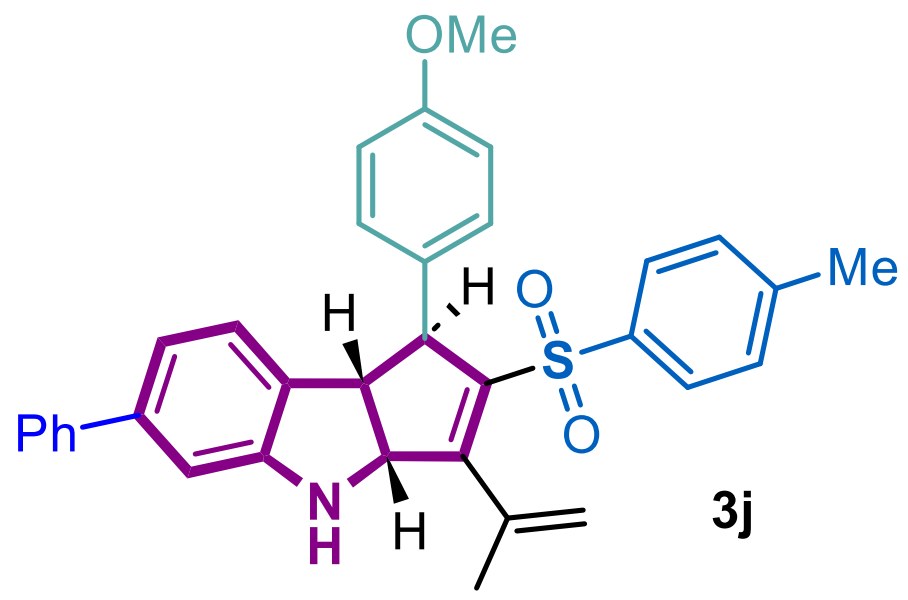

3j

(1S)-1-(4-methoxyphenyl)-6-phenyl-3-(prop-1-en-2-yl)-2-tosyl-1,3a,4,8b-tetrahydrocyclopenta[b]indole (**3j**): Prepared according to GP-3 on 0.3 mmol (110 mg)
Dark green solid (81mg, 49% yield)
Rf = 0.38 (30% EA/hexane)
Melting point = 148-150°C
NMR Spectroscopy:
^{1}H NMR (400 MHz, CHLOROFORM-D) δ 7.55 (d, *J* = 7.9 Hz, 2H), 7.43 (t, *J* = 7.5 Hz, 2H), 7.34 (ddd, *J* = 7.3, 3.7, 1.2 Hz, 1H), 7.23 (dd, *J* = 11.1, 4.4 Hz, 3H), 7.05 (d, *J* = 8.6 Hz, 2H), 7.03 – 6.97 (m, 3H), 6.85 (d, *J* = 1.4 Hz, 1H), 6.81 (d, *J* = 8.7 Hz, 2H), 5.34 – 5.20 (m, 1H), 5.04 (dd, *J* = 7.7, 1.7 Hz, 1H), 4.85 (s, 1H), 4.48 (s, 1H), 4.22 (s, 1H), 3.82 (s, 3H), 3.81 (d, *J* = 4.3 Hz, 1H), 2.32 (s, 3H), 1.89 (s, 3H) ppm.

^{13}C NMR (101 MHz, CHLOROFORM-D) δ 158.8, 154.5, 149.0, 143.8, 142.2, 141.9, 141.6, 138.1, 137.3, 133.9, 129.9, 129.0, 128.7, 128.7, 127.9, 127.2, 124.8, 119.2, 118.1, 114.2, 109.1, 70.7, 59.4, 55.4, 53.6, 22.5, 21.6 ppm.
HRMS (ESI) m/z: [M+H]+ Calcd for $C_{34}H_{32}NO_3S^+$ 534.2097; Found 534.2108.

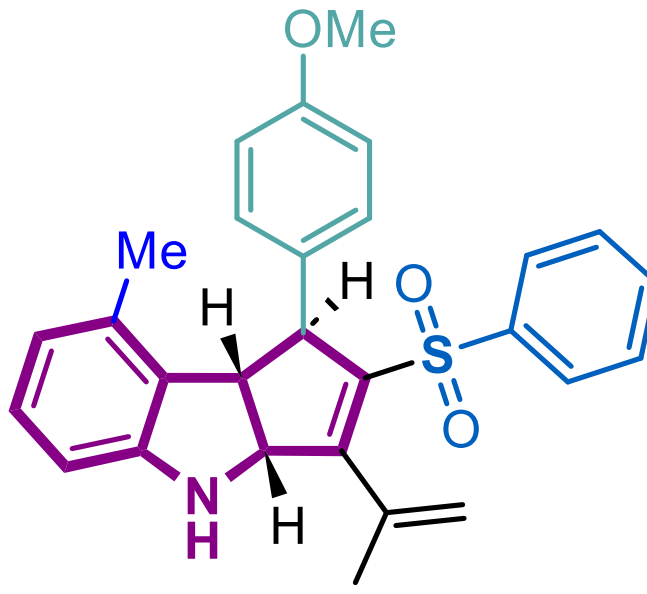

3k

(1S)-1-(4-methoxyphenyl)-8-methyl-3-(prop-1-en-2-yl)-2-tosyl-1,3a,4,8b-tetrahydrocyclopenta[b]indole (**3k**): Prepared according to GP-3 on 0.3 mmol (110 mg)
Brown solid (130 mg, 93% yield)
Rf = 0.39 (30% EA/hexane)
Melting point = 160-162°C
NMR Spectroscopy:
^{1}H NMR (400 MHz, CHLOROFORM-D) δ 7.15 (d, *J* = 8.3 Hz, 2H), 7.08 – 6.92 (m, 5H), 6.85 – 6.72 (m, 2H), 6.59 (d, *J* = 7.5 Hz, 1H), 6.49 (d, *J* = 7.7 Hz, 1H), 5.30 (t, *J* = 1.4 Hz, 1H), 5.07 (dd, *J* = 8.5, 1.9 Hz, 1H), 4.98 (s, 1H), 4.50 (s, 1H), 4.11 (s, 1H), 3.85 (d, *J* = 8.7 Hz, 1H), 3.82 (s, 3H), 2.33 (s, 3H), 2.27 (s, 3H), 1.88 (s, 3H) ppm.
^{13}C NMR (101 MHz, CHLOROFORM-D) δ 158.7, 154.5, 148.5, 143.7, 141.9, 138.3, 137.5, 135.1, 134.2, 129.0, 128.6, 127.9, 121.4, 118.3, 114.2, 107.9, 70., 58.5, 55.5, 54.1, 22.5, 21.6, 19.6 ppm.
HRMS (ESI) m/z: [M+H]+ Calcd for $C_{29}H_{30}NO_3S^+$ 472.1941; Found 472.1960.

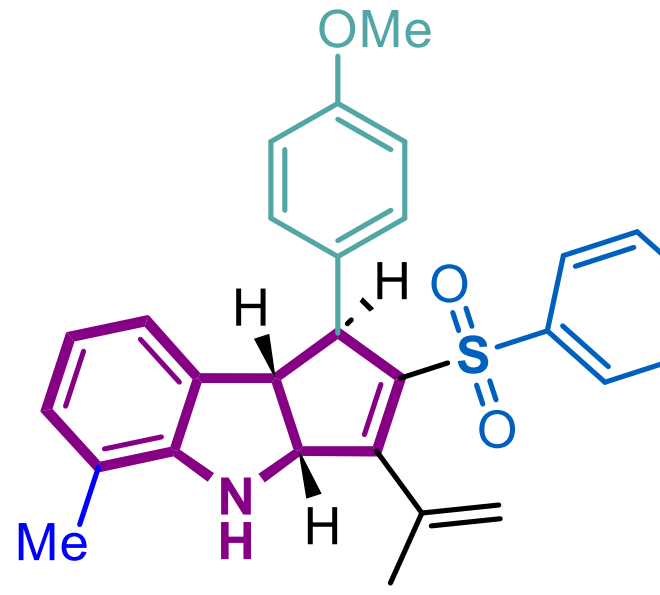

3l

8,8,9-trimethyl-6-tosyl-8,9-dihydro-7H-pyrido[2,3-b]indol-7-one (**3l**): Prepared according to GP-3 on 0.3 mmol (110 mg)
Off-white solid (127 mg, 91% yield)
Rf = 0.38 (30% EA/hexane)
Melting point = 117-119°C

NMR Spectroscopy:

^{1}H NMR (400 MHz, CHLOROFORM-D) δ 7.19 (d, *J* = 8.3 Hz, 2H), 7.02 (ddd, *J* = 13.7, 11.3, 8.0 Hz, 5H), 6.92 (d, *J* = 7.4 Hz, 1H), 6.82 – 6.77 (m, 2H), 6.74 (t, *J* = 7.4 Hz, 1H), 5.31 – 5.22 (m, 1H), 5.00 (dd, *J* = 7.8, 1.8 Hz, 1H), 4.78 (s, 1H), 4.43 (s, 1H), 3.96 (s, 1H), 3.81 (s, 3H), 3.80 (d, *J* = 7.9 Hz, 1H), 2.33 (s, 3H), 2.11 (s, 3H), 1.89 (s, 3H) ppm.

^{13}C NMR (101 MHz, CHLOROFORM-D) δ 158.7, 154.6, 146.9, 143.8, 141.8, 138.2, 137.5, 134.1, 130.1, 129.5, 129.0, 128.7, 127.9, 122.1, 120.1, 119.8, 117.7, 114.2, 70.4, 59.6, 55.5, 54.4, 22.6, 21.6, 17.0 ppm.

HRMS (ESI) m/z: [M+H]+ Calcd for $C_{29}H_{30}NO_3S^+$ 472.1941; Found 472.1917.

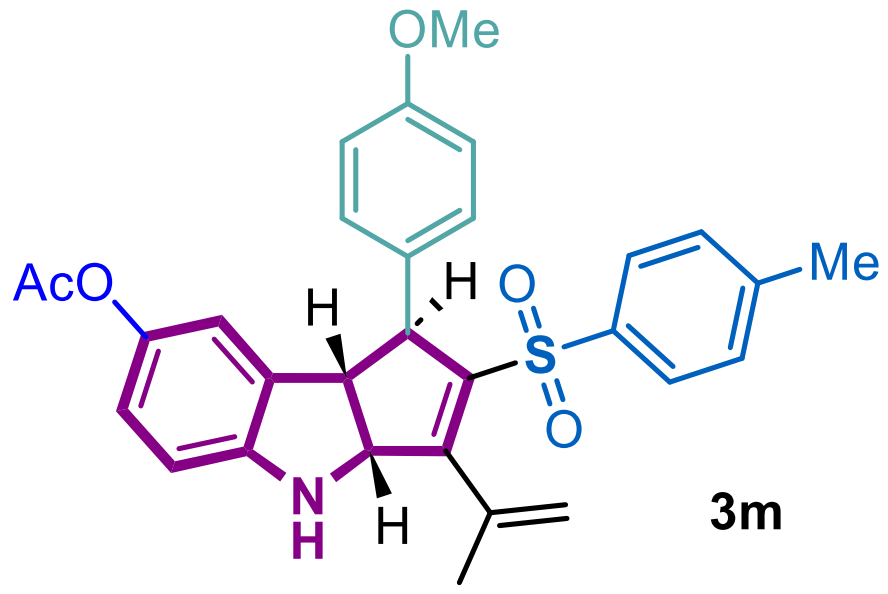

3m

(1S)-1-(4-methoxyphenyl)-3-(prop-1-en-2-yl)-2-tosyl-1,3a,4,8b-tetrahydrocyclopenta[b]indol-7-yl acetate (**3m**): Prepared according to GP-3 on 0.3 mmol (110mg)

Brown thick liquid (125 mg, 79% yield)

Rf = 0.23 (35% EA/hexane)

NMR Spectroscopy:

^{1}H NMR (400 MHz, CHLOROFORM-D) δ 7.19 (d, *J* = 8.3 Hz, 2H), 7.04 – 6.97 (m, 4H), 6.92 (dd, *J* = 2.2, 1.0 Hz, 1H), 6.77 (d, *J* = 8.7 Hz, 3H), 6.58 (d, *J* = 8.4 Hz, 1H), 5.23 (s, 1H), 4.99 (dd, *J* = 7.7, 1.8 Hz, 1H), 4.77 (s, 1H), 4.37 (s, 1H), 4.10 (s, 1H), 3.79 (s, 3H), 3.75 (dd, *J* = 7.7, 0.8 Hz, 1H), 2.31 (s, 3H), 2.27 (s, 3H), 1.88 (s, 3H) ppm.

^{13}C NMR (101 MHz, CHLOROFORM-D) δ 170.2, 158.8, 154.4, 146.2, 144.0, 143.9, 141.8, 138.1, 137.3, 133.6, 131.8, 129.0, 128.6, 127.8, 121.5, 118.1, 117.9, 114.2, 110.4, 70.9, 59.2, 55.4, 54.0, 22.5, 21.6, 21.2 ppm.

HRMS (ESI) m/z: [M+H]+ Calcd for $C_{30}H_{30}NO_5S^+$ 516.1839; Found 516.1872.

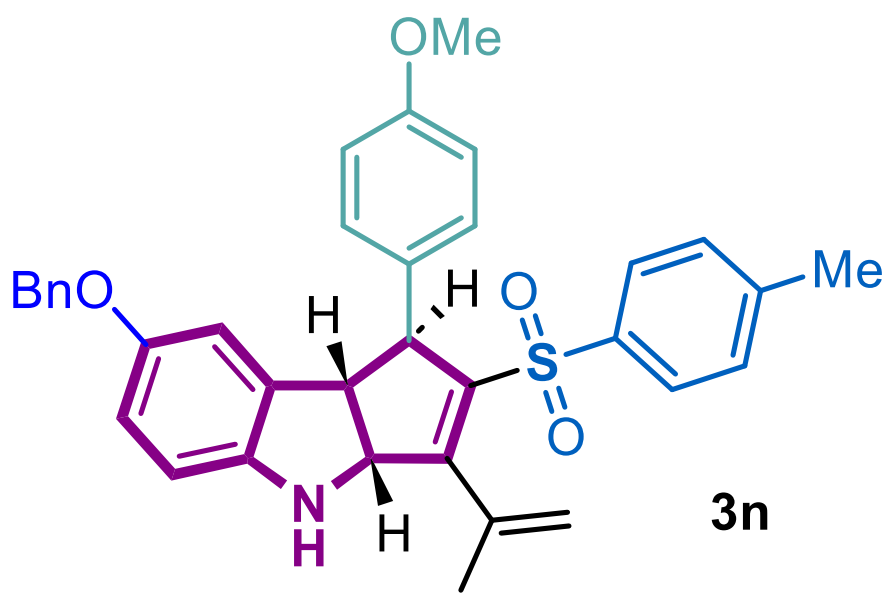

3n

(1S)-7-(benzyloxy)-1-(4-methoxyphenyl)-3-(prop-1-en-2-yl)-2-tosyl-1,3a,4,8b-tetrahydrocyclopenta[b]indole (**3n**): Prepared according to GP-3 on 0.3 mmol (110 mg)
Brown thick liquid (70 mg, 72% yield)
Rf = 0.28 (35% EA/hexane)
NMR Spectroscopy:
^{1}H NMR (400 MHz, CHLOROFORM-D) δ 7.42 (ddt, *J* = 20.4, 14.4, 1.5 Hz, 5H), 7.20 (d, *J* = 8.3 Hz, 2H), 7.05 – 6.97 (m, 4H), 6.88 (dd, *J* = 2.5, 0.6 Hz, 1H), 6.83 – 6.77 (m, 2H), 6.77 – 6.69 (m, 1H), 6.58 (d, *J* = 8.4 Hz, 1H), 5.28 (t, *J* = 1.5 Hz, 1H), 5.05 – 4.94 (m, 3H), 4.85 (s, 1H), 4.40 (s, 1H), 3.96 (s, 1H), 3.82 (s, 3H), 3.75 (d, *J* = 8.4 Hz, 1H), 2.32 (s, 3H), 1.88 (s, 3H) ppm.
^{13}C NMR (101 MHz, CHLOROFORM-D) δ 158.8, 154.7, 153.6, 143.8, 142.4, 141.7, 138.1, 137.4, 133.9, 132.3, 129.0, 128.7, 127.9, 127.7, 118.1, 115.0, 114.2, 112.4, 111.2, 71.0, 59.2, 54.4, 22.5, 21.6 ppm.
HRMS (ESI) m/z: [M+H]+ Calcd for $C_{35}H_{34}NO_4S^+$ 564.2209; Found 564.2198.

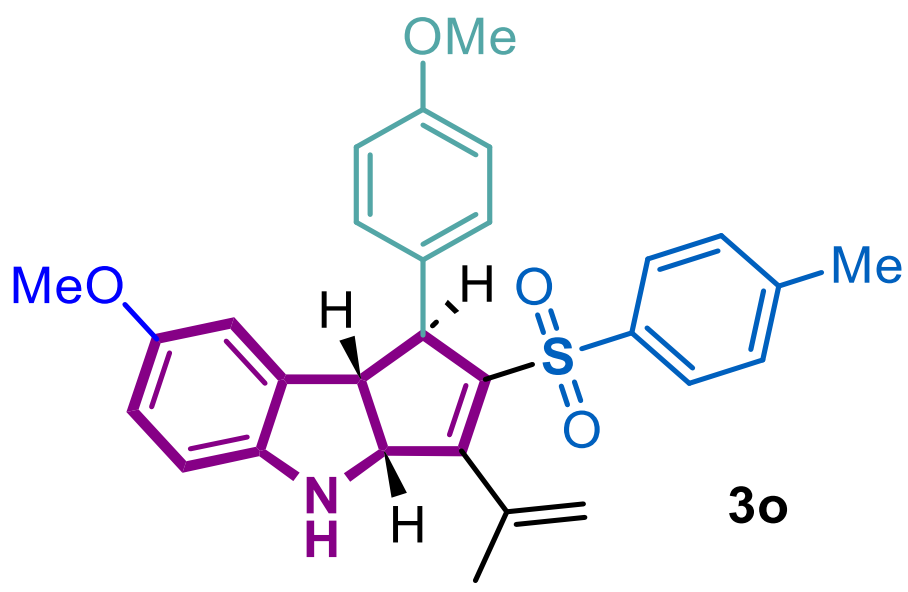

3o

(1S)-7-methoxy-1-(4-methoxyphenyl)-3-(prop-1-en-2-yl)-2-tosyl-1,3a,4,8b-tetrahydrocyclopenta[b]indole (**3o**): Prepared according to GP-3 on 0.3 mmol (110 mg)
Brown solid (118mg, 81% yield)
Rf = 0.31 (30% EA/hexane)
Melting point = 78-80°C
NMR Spectroscopy:
^{1}H NMR (400 MHz, CHLOROFORM-D) δ 7.20 (d, *J* = 8.2 Hz, 2H), 7.07 – 6.97 (m, 4H), 6.83 – 6.75 (m, 3H), 6.66 (dd, *J* = 8.4, 2.5 Hz, 1H), 6.58 (d, *J* = 8.4 Hz, 1H), 5.27 (d, *J* = 1.3 Hz, 1H), 4.97 (dd, *J* = 7.6, 1.7 Hz, 1H), 4.83 (s, 1H), 4.40 (s, 1H), 3.92 (s, 1H), 3.82 (s, 3H), 3.77 (s, 3H), 3.74 (d, *J* = 7.5 Hz, 1H), 2.33 (s, 3H), 1.88 (s, 3H) ppm.
^{13}C NMR (101 MHz, CHLOROFORM-D) δ 195.6, 153.9, 144.9, 143.8, 141.2, 138.2, 136.8, 130.3, 129.9, 129.4, 128.4, 125.7, 124.1, 123.4, 118.1, 111.6, 106.7, 49.7, 25.6, 21.7 ppm.
HRMS (ESI) m/z: [M+H]+ Calcd for $C_{29}H_{30}NO_4S^+$ 488.1890; Found 488.1896.

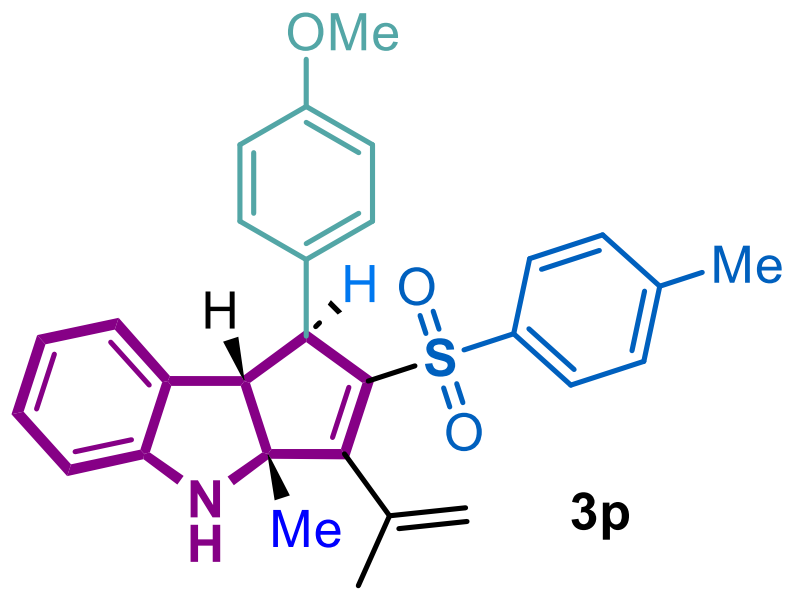

3p

(1S)-1-(4-methoxyphenyl)-3a-methyl-3-(prop-1-en-2-yl)-2-tosyl-1,3a,4,8b-tetrahydrocyclopenta[b]indole (**3p**): Prepared according to GP-3 on 0.3 mmol (110 mg)
Light brown solid (132 mg, 91% yield)
Rf = 0.37 (30% EA/hexane)
Melting point = 126-128°C
NMR Spectroscopy:
^{1}H NMR (400 MHz, CHLOROFORM-D) δ 7.29 – 7.22 (m, 2H), 7.13 – 6.94 (m, 6H), 6.83 – 6.76 (m, 2H), 6.71 (td, *J* = 7.4, 0.8 Hz, 1H), 6.53 (d, *J* = 7.7 Hz, 1H), 5.20 (t, *J* = 1.6 Hz, 1H), 4.55 (d, *J* = 0.9 Hz, 1H), 4.28 (d, *J* = 1.9 Hz, 1H), 4.13 (s, 1H), 3.80 (s, 3H), 3.43 (s, 1H), 2.32 (s, 3H), 2.12 (s, 3H), 1.45 (s, 3H) ppm.
^{13}C NMR (101 MHz, CHLOROFORM-D) δ 158.8, 158.6, 148.0, 143.7, 139.5, 138.6, 138.2, 133.8, 131.4, 129.0, 128.9, 128.5, 127.9, 124.5, 119.4, 116.2, 114.1, 109.6, 77.8, 61.5, 58.6, 55.4, 55.4, 25.5, 24.9, 21.6 ppm.
HRMS (ESI) m/z: [M+H]+ Calcd for $C_{29}H_{30}NO_3S^+$ 472.1941; Found 472.1937.

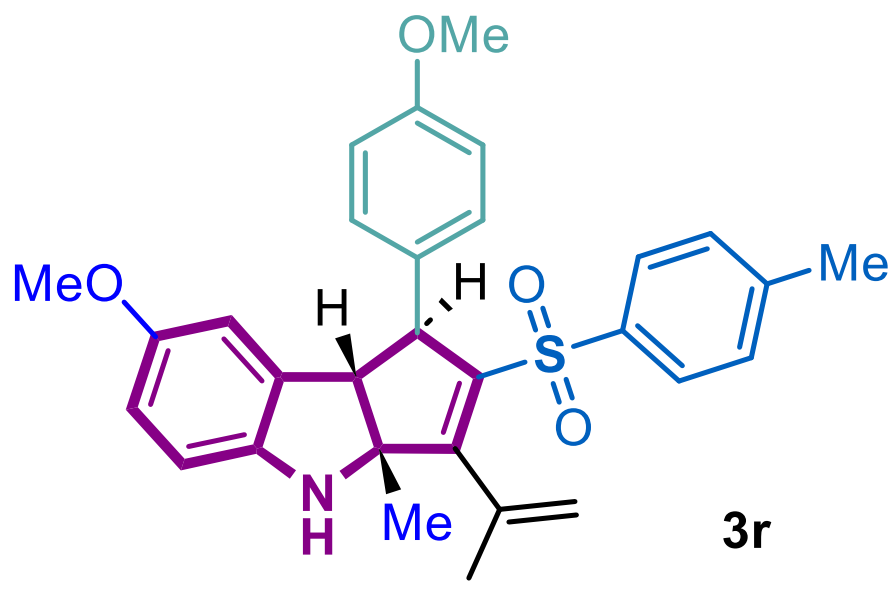

3r

(1S)-7-methoxy-1-(4-methoxyphenyl)-3a-methyl-3-(prop-1-en-2-yl)-2-tosyl-1,3a,4,8b-tetrahydrocyclopenta[b]indole (3r): Prepared according to GP-3 on 0.3 mmol (110 mg)
Brown solid (111 mg, 72% yield)
Rf = 0.38 (25% EA/hexane)
Melting point = 145-147°C
NMR Spectroscopy:
^{1}H NMR (400 MHz, CHLOROFORM-D) δ 7.28 (d, *J* = 8.3 Hz, 2H), 7.11 (d, *J* = 8.6 Hz, 2H), 7.00 (d, *J* = 8.1 Hz, 2H), 6.81 (d, *J* = 8.6 Hz, 2H), 6.69 – 6.56 (m, 2H), 6.49 (d, *J* = 8.3 Hz, 1H), 5.23 (t, *J* = 1.5 Hz, 1H), 4.57 (s, 1H), 4.23 (d, *J* = 1.7 Hz, 1H), 3.93 (s, 1H), 3.81 (s, 3H), 3.73 (s, 3H), 3.41 (s, 1H), 2.33 (s, 3H), 2.13 (s, 3H), 1.44 (s, 3H) ppm.

^{13}C NMR (101 MHz, CHLOROFORM-D) δ 159.0, 158.6, 153.9, 143.7, 141.9, 139.1, 138.7, 138.0, 133.7, 133.0, 129.0, 128.9, 127.9, 116.1, 114.1, 113.7, 110.8, 110.5, 78.3, 61.9, 58.1, 56.0, 55.4, 55.4 ppm.
HRMS (ESI) m/z: [M+H]+ Calcd for $C_{30}H_{32}NO_4S^+$ 502.2052; Found 502.2062.

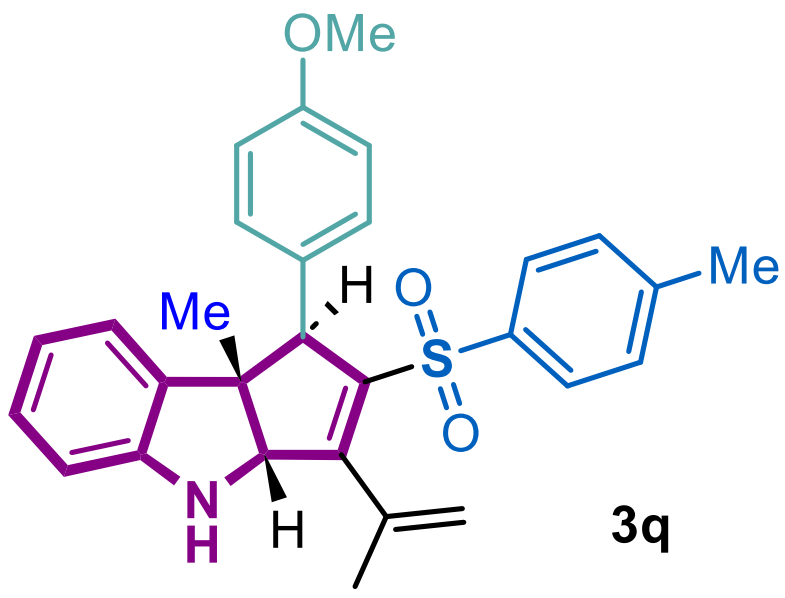

3q

(1R)-1-(4-methoxyphenyl)-8b-methyl-3-(prop-1-en-2-yl)-2-tosyl-1,3a,4,8b-tetrahydrocyclopenta[b]indole (**3q**): Prepared according to GP-3 on 0.3 mmol (110 mg)
Brown solid (124 mg, 85% yield)
Rf = 0.36 (30% EA/hexane)
Melting point = 128-130°C
NMR Spectroscopy:
^{1}H NMR (400 MHz, CHLOROFORM-D) δ 7.29 – 7.22 (m, 2H), 7.13 – 6.94 (m, 6H), 6.83 – 6.76 (m, 2H), 6.71 (td, *J* = 7.4, 0.8 Hz, 1H), 6.53 (d, *J* = 7.7 Hz, 1H), 5.20 (t, *J* = 1.6 Hz, 1H), 4.55 (d, *J* = 0.9 Hz, 1H), 4.28 (d, *J* = 1.9 Hz, 1H), 4.13 (s, 1H), 3.80 (s, 3H), 3.43 (s, 1H), 2.32 (s, 3H), 2.12 (s, 3H), 1.45 (s, 3H) ppm.
^{13}C NMR (101 MHz, CHLOROFORM-D) δ 158.8, 158.6, 148.0, 143.7, 139.5, 138.6, 138.2, 133.8, 131.4, 129.0, 128.9, 128.5, 127.9, 124.5, 119.4, 116.2, 114.1, 109.6, 77.8, 61.5, 58.6, 55.4, 55.4, 25.5, 24.9, 21.6 ppm.
HRMS (ESI) m/z: [M+H]+ Calcd for $C_{30}H_{30}NO_3S^+$ 484.1946; Found 484.1976.

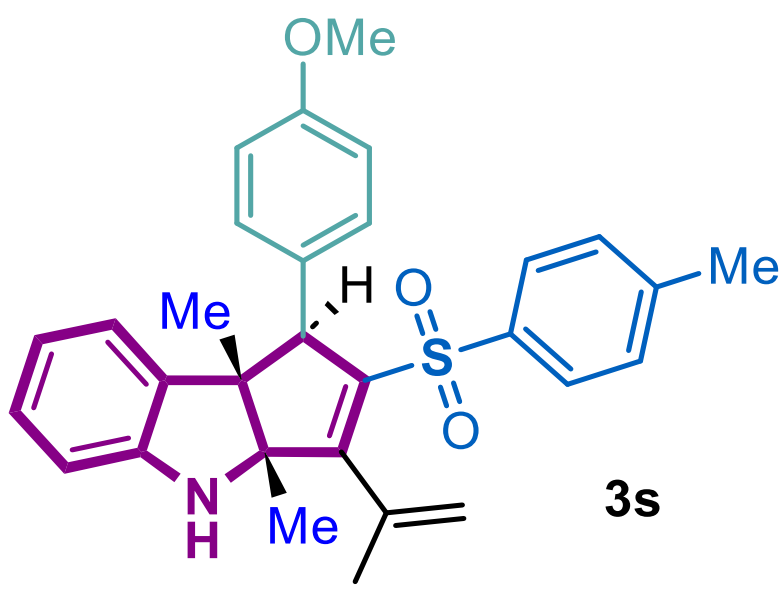

3s

(1R)-1-(4-methoxyphenyl)-3a,8b-dimethyl-3-(prop-1-en-2-yl)-2-tosyl-1,3a,4,8b-tetrahydrocyclopenta[b]indole (**3s**): Prepared according to GP-3 on 0.3 mmol (110 mg)
Yellow thick liquid (74 mg, 49% yield)
Rf = 0.36 (30% EA/hexane)
NMR Spectroscopy:

^{1}H NMR (400 MHz, CHLOROFORM-D) δ 7.23 (d, *J* = 8.3 Hz, 2H), 7.08 (d, *J* = 7.8 Hz, 2H), 7.05 – 6.99 (m, 2H), 6.97 (dt, *J* = 6.7, 1.3 Hz, 2H), 6.83 (d, *J* = 8.6 Hz, 2H), 6.70 (td, *J* = 7.5, 1.0 Hz, 1H), 6.50 (dd, *J* = 8.1, 0.9 Hz, 1H), 5.19 (t, *J* = 1.6 Hz, 1H), 4.31 (d, *J* = 10.1 Hz, 2H), 4.22 (s, 1H), 3.81 (s, 3H), 2.33 (s, 3H), 2.28 – 2.08 (m, 3H), 1.29 (s, 3H), 0.74 (s, 3H) ppm.
^{13}C NMR (101 MHz, CHLOROFORM-D) δ 158.8, 158.6, 146.5, 143.6, 139.5, 138.6, 138.5, 138.1, 130.2, 129.0, 128.1, 127.5, 122.9, 119.6, 115.8, 113.8, 109.2, 78.9, 60.8, 56.7, 55.4, 25.7, 21.6, 20.6, 19.9 ppm.
HRMS (ESI) m/z: [M+H]+ Calcd for $C_{30}H_{32}NO_3S^+$ 486.2097; Found 486.2093.

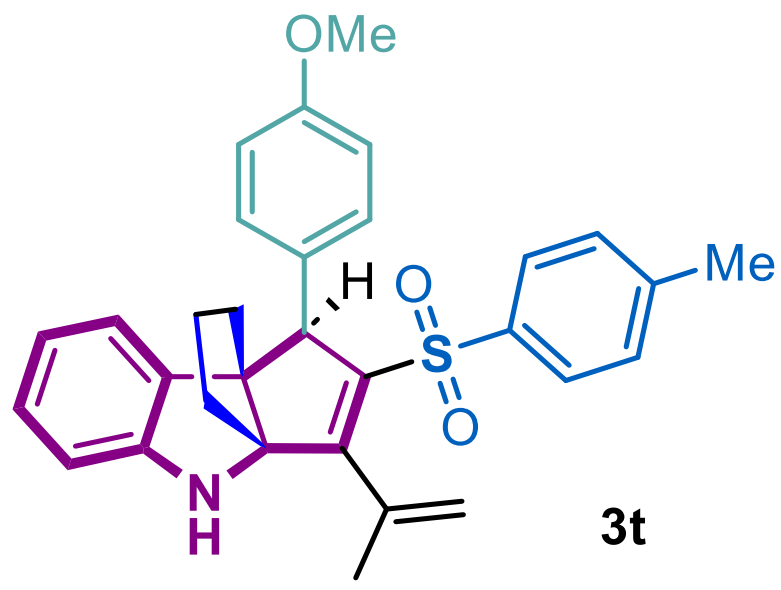

3t

(1R,3aR,8bS)-1-(4-methoxyphenyl)-3-(prop-1-en-2-yl)-2-tosyl-1H,4H-3a,8b-propanocyclopenta[b]indole (**3t**): Prepared according to GP-3 on 0.3 mmol (110 mg). The diastereomeric ratio is 1.25:1. Spectral data is given for the diastereomeric mixture.
Brown thick liquid (51 mg, 33% yield)
Rf = 0.33 (30% EA/hexane)
NMR Spectroscopy:
^{1}H NMR (400 MHz, CHLOROFORM-D) δ7.31 (d, *J* = 8.3 Hz, 2H), 7.11 – 7.07 (m, 2H), 7.05 – 7.00 (m, 3H), 6.80 – 6.73 (m, 2H), 6.55 (d, *J* = 7.7 Hz, 1H), 6.24 (td, *J* = 7.5, 1.0 Hz, 1H), 6.01 – 5.93 (m, 1H), 5.22 – 5.16 (m, 1H), 4.86 (dd, *J* = 1.5, 0.9 Hz, 1H), 4.45 (s, 1H), 4.05 (s, 1H), 3.82 (s, 3H), 2.34 (s, 3H), 2.13 – 2.02 (m, 2H), 1.99 (s, 3H), 1.88 – 1.75 (m, 3H), 1.44 – 1.36 (m, 1H) ppm.
^{13}C NMR (101 MHz, CHLOROFORM-D) δ 158.7, 158.3, 157.4, 156.5, 150.1, 149.2, 143.6, 143.3, 140.1, 139.6, 139.3, 138.7, 138.4, 138.3, 137.7, 131.8, 131.3, 131.2, 131.1, 129.9, 128.9, 128.9, 128.7, 128.4, 128.3, 127.4, 126.1, 123.8, 119.8, 117.3, 112.4, 109.5, 108.5, 89.8, 88.3, 68.2, 67.3, 63.2, 62.4, 55.4, 55.3, 55.2, 44.2, 41.7, 39.5, 37.4, 27.2, 26.1, 23.9, 23.6, 21.6 ppm.
HRMS (ESI) m/z: [M+H]+ Calcd for $C_{31}H_{32}NO_3S^+$ 498.2097; Found 498.2082.

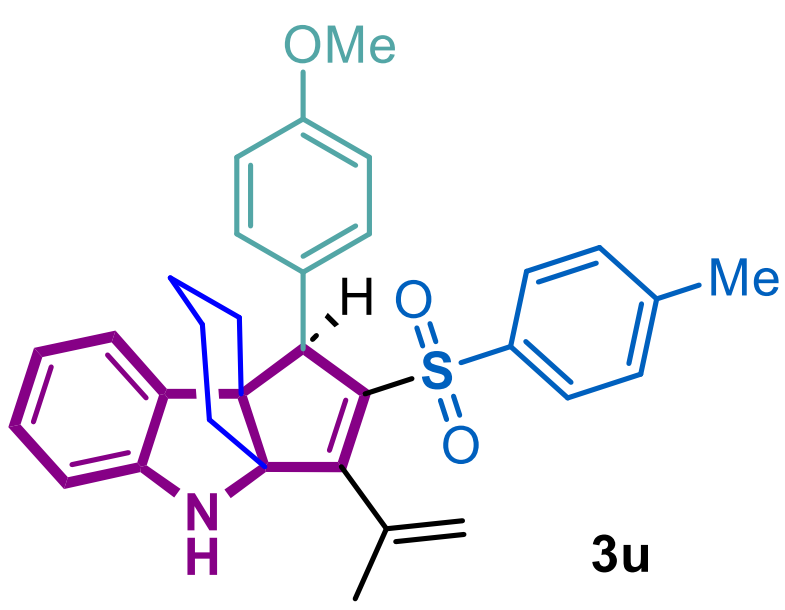

3u

(4bR,8aS,12R)-12-(4-methoxyphenyl)-10-(prop-1-en-2-yl)-11-tosyl-5,6,7,8-tetrahydro-9H-8a,4b-prop[1]enocarbazole (3u): Prepared according to GP-3 on 0.3 mmol (110 mg).
The diastereomeric ratio is 3.33:1. Spectral data are given for the diastereomeric mixture.
Yellow thick liquid (81 mg, 51% yield)
Rf = 0.34 (30% EA/hexane)
NMR Spectroscopy:
^{1}H NMR (400 MHz, CHLOROFORM-D) δ 7.25 – 7.16 (m, 4H), 7.04 – 6.93 (m, 5H), 6.85 – 6.75 (m, 1H), 6.65 (td, *J* = 7.4, 0.9 Hz, 1H), 6.58 – 6.49 (m, 1H), 5.12 (t, *J* = 1.6 Hz, 1H), 4.22 (d, *J* = 2.0 Hz, 2H), 4.18 (s, 1H), 3.82 (s, 3H), 2.32 (s, 3H), 2.13 (s, 3H), 2.00 – 1.80 (m, 3H), 1.42 – 1.14 (m, 4H), 1.01 – 0.91 (m, 1H) ppm.
^{13}C NMR (101 MHz, CHLOROFORM-D) δ 159.6, 158.9, 158.4, 149.3, 146.6, 143.6, 143.4, 141.1, 139.8, 139.7, 139.4, 138.4, 131.4, 130.3, 129.9, 129.3, 129.0, 128.8, 128.3, 127.8, 127.6, 127.4, 125.7, 122.1, 119.4, 118.5, 116.3, 115.4, 114.2, 113.4, 109.5, 109.2, 78.8, 77.9, 63.6, 59.4, 58.5, 55.3, 33.4, 32.4, 32.1, 31.1, 25.3, 24.2, 21.6, 19.8, 19.5, 19.1 ppm.
^{19}F NMR (376 MHz, CHLOROFORM-D) δ = -62.96 ppm.
HRMS (ESI) m/z: [M+H]+ Calcd for $C_{32}H_{34}NO_3S^+$ 512.2259; Found 512.2273.

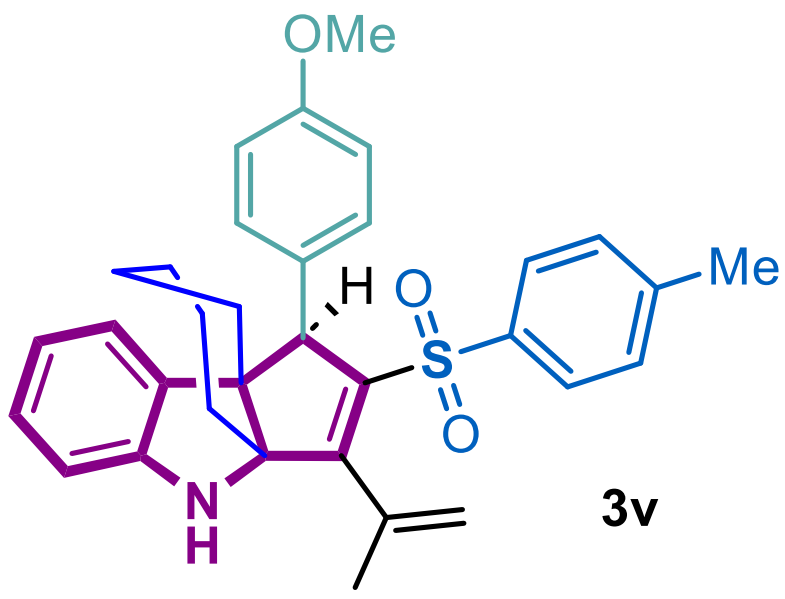

3v

(5aS,10aR,11R)-11-(4-methoxyphenyl)-13-(prop-1-en-2-yl)-12-tosyl-7,8,9,10-tetrahydro-5H,6H-5a,10a-prop[1]enocyclohepta[b]indole (3v): Prepared according to GP-3 on 0.3 mmol (110 mg).
The diastereomeric ratio is 25:1. Spectral data are given for the diastereomeric mixture.
Yellow solid (70 mg, 43% yield)
Rf = 0.32 (30% EA/hexane)
Melting point = 53-55°C
NMR Spectroscopy:
^{1}H NMR (400 MHz, CHLOROFORM-D) δ 7.18 (d, *J* = 8.3 Hz, 2H), 7.12 (dd, *J* = 8.3, 2.1 Hz, 1H), 7.05 (dd, *J* = 12.1, 4.3 Hz, 2H), 6.99 (dd, *J* = 8.5, 2.0 Hz, 1H), 6.94 (d, *J* = 8.1 Hz, 2H), 6.88 (dd, *J* = 8.3, 2.6 Hz, 1H), 6.79 (dd, *J* = 8.5, 2.6 Hz, 1H), 6.73 (dd, *J* = 10.9, 4.1 Hz, 1H), 6.52 (d, *J* = 7.7 Hz, 1H), 5.17 (s, 1H), 4.35 (s, 1H), 4.17 (d, *J* = 10.1 Hz, 2H), 3.83 (s, 3H), 2.32 (s, 3H), 2.16 (s, 3H), 1.96 (dd, *J* = 14.3, 5.6 Hz, 1H), 1.89 – 1.77 (m, 1H), 1.68 (t, *J* = 13.4 Hz, 1H), 1.54 (dd, *J* = 14.4, 8.7 Hz, 3H), 1.25 (d, *J* = 4.3 Hz, 1H), 1.02 – 0.85 (m, 2H), 0.75 (dd, *J* = 25.0, 11.9 Hz, 1H) ppm.

^{13}C NMR (101 MHz, CHLOROFORM-D) δ 158.8, 158.1, 147.3, 143.4, 139.3, 138.7, 138.4, 137.0, 131.8, 131.4, 128.9, 128.4, 128.2, 127.5, 123.4, 119.1, 116.4, 113.9, 108.6, 82.7, 63.7, 61.7, 55.4, 38.3, 31.9, 31.4, 26.1, 23.8, 23.2, 21.6 ppm.
HRMS (ESI) m/z: [M+H]+ Calcd for $C_{33}H_{36}NO_3S^+$ 526.2410; Found 526.2404.

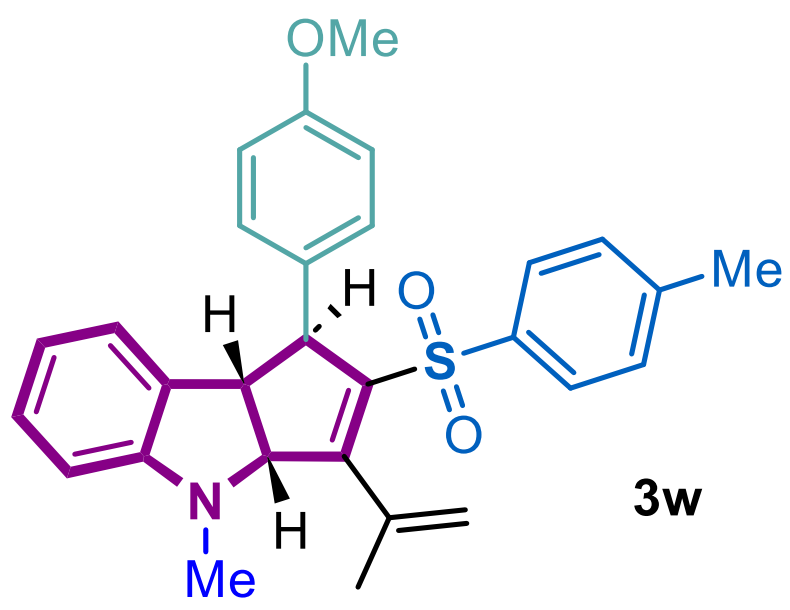

3w

(1S)-1-(4-methoxyphenyl)-4-methyl-3-(prop-1-en-2-yl)-2-tosyl-1,3a,4,8b-tetrahydrocyclopenta[b]indole (**3w**): Prepared according to GP-3 on 0.3 mmol (110 mg)
Brown thick liquid (73 mg, 50% yield)
Rf = 0.30 (30% EA/hexane)
NMR Spectroscopy:
^{1}H NMR (400 MHz, CHLOROFORM-D) δ 7.20 – 7.15 (m, 2H), 7.14 – 7.07 (m, 2H), 7.00 – 6.93 (m, 4H), 6.77 – 6.71 (m, 2H), 6.68 (td, *J* = 7.3, 0.8 Hz, 1H), 6.42 (d, *J* = 7.8 Hz, 1H), 5.23 (t, *J* = 1.6 Hz, 1H), 4.99 – 4.95 (m, 1H), 4.85 (dd, *J* = 9.1, 1.9 Hz, 1H), 4.45 (t, *J* = 2.0 Hz, 1H), 3.94 – 3.87 (m, 1H), 3.81 (s, 3H), 2.96 (s, 3H), 2.33 (s, 3H), 2.02 (d, *J* = 1.0 Hz, 3H) ppm.
^{13}C NMR (101 MHz, CHLOROFORM-D) δ 158.7, 155.0, 150.5, 143.6, 141.8, 139.7, 138.4, 134.5, 130.9, 128.9, 128.8, 128.0, 124.3, 117.9, 116.3, 114.1, 107.3, 79.8, 59.4, 55.5, 53.9, 35.9, 23.2, 21.6 ppm.
HRMS (ESI) m/z: [M+H]+ Calcd for $C_{29}H_{30}NO_3S^+$ 472.1941; Found 472.1952.

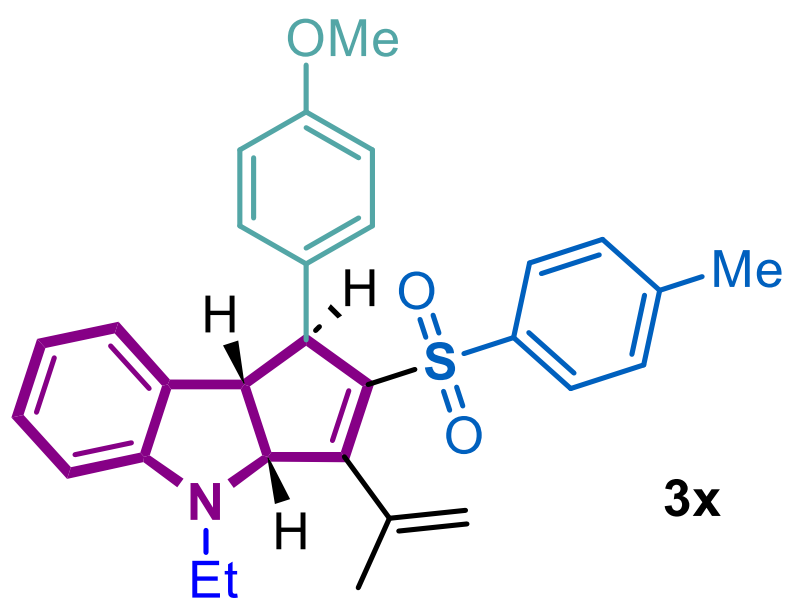

3x

(1S)-4-ethyl-1-(4-methoxyphenyl)-3-(prop-1-en-2-yl)-2-tosyl-1,3a,4,8b-tetrahydrocyclopenta[b]indole (**3x**): Prepared according to GP-3 on 0.3 mmol (110 g)
Dark green solid (105 mg, 70% yield)
Rf = 0.31 (30% EA/hexane)
Melting point = 103-105°C
NMR Spectroscopy:

^{1}H NMR (400 MHz, CHLOROFORM-D) δ 7.22 – 7.06 (m, 4H), 6.97 (d, *J* = 8.1 Hz, 4H), 6.71 (dd, *J* = 19.0, 7.8 Hz, 3H), 6.48 (d, *J* = 8.0 Hz, 1H), 5.21 (s, 1H), 5.04 – 4.85 (m, 2H), 4.47 (s, 1H), 3.87 (d, *J* = 8.8 Hz, 1H), 3.81 (s, 3H), 3.43 (dt, *J* = 14.0, 7.0 Hz, 1H), 3.25 (td, *J* = 14.2, 7.0 Hz, 1H), 2.32 (s, 3H), 2.01 (s, 3H), 1.08 (t, *J* = 7.0 Hz, 3H) ppm.
^{13}C NMR (101 MHz, CHLOROFORM-D) δ 158.3, 155.3, 148.9, 143.2, 141.1, 139.5, 138.1, 134.2, 131.5, 128.5, 128.5, 128.3, 127.7, 124.1, 117.8, 115.4, 113.8, 108.2, 58.8, 55.1, 53.9, 42.9, 29.5, 23.2, 21.3, 11.8 ppm.
HRMS (ESI) m/z: [M+H]+ Calcd for $C_{30}H_{32}NO_3S^+$486.2097; Found 486.2104.

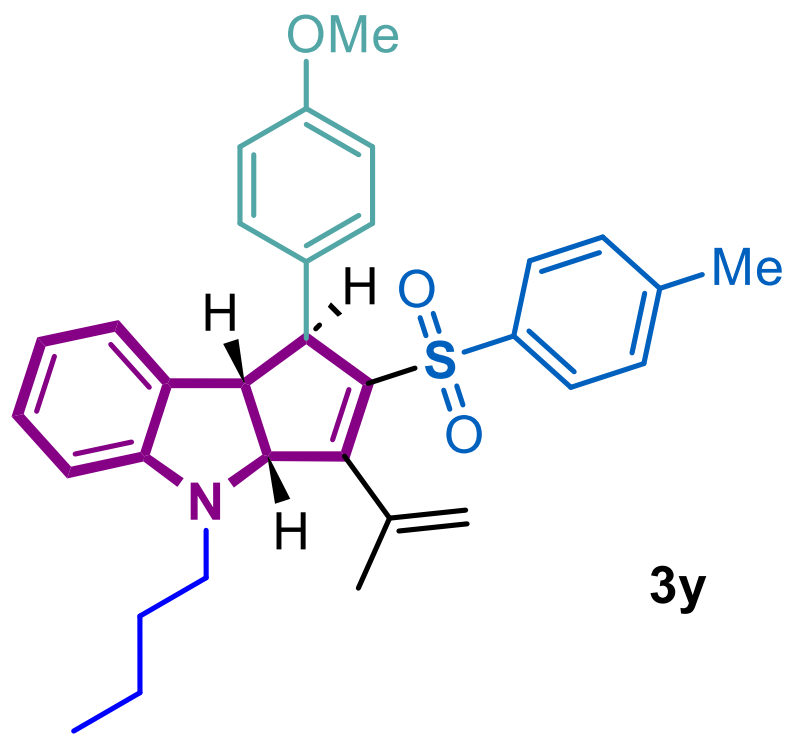

3y

(1S)-4-butyl-1-(4-methoxyphenyl)-3-(prop-1-en-2-yl)-2-tosyl-1,3a,4,8b-tetrahydrocyclopenta[b]indole (**3y**): Prepared according to GP-3 on 0.3 mmol (110 mg)
Brown solid (86 mg, 54% yield)
Rf = 0.33 (30% EA/hexane)
Melting point = 93-95°C
NMR Spectroscopy:
^{1}H NMR (400 MHz, CHLOROFORM-D) δ 7.17 (d, *J* = 8.3 Hz, 2H), 7.10 (dd, *J* = 10.1, 7.7 Hz, 2H), 6.97 (d, *J* = 8.5 Hz, 4H), 6.74 (d, *J* = 8.6 Hz, 2H), 6.68 (t, *J* = 7.3 Hz, 1H), 6.45 (d, *J* = 7.8 Hz, 1H), 5.23 – 5.18 (m, 1H), 4.96 (dd, *J* = 8.6, 1.8 Hz, 1H), 4.88 (s, 1H), 4.47 (s, 1H), 3.85 (d, *J* = 8.5 Hz, 1H), 3.81 (s, 3H), 3.38 (ddd, *J* = 14.5, 8.7, 5.7 Hz, 1H), 3.25 – 3.14 (m, 1H), 2.32 (s, 3H), 1.99 (s, 3H), 1.58 – 1.39 (m, 2H), 1.34 – 1.16 (m, 3H), 0.88 (t, *J* = 7.3 Hz, 2H) ppm.
^{13}C NMR (101 MHz, CHLOROFORM-D) δ 172.9, 158.7, 155.4, 149.3, 143.6, 141.5, 139.7, 138.4, 134.4, 131.4, 128.9, 128.8, 128.7, 128.0, 124.4, 117.9, 116.0, 114.1, 108.1, 58.8, 55.5, 54.1, 48.3, 29.3, 23.4, 21.6, 20.4, 14.1 ppm.
HRMS (ESI) m/z: [M+H]$^+$ Calcd for $C_{32}H_{36}NO_3S^+$ 514.2416; Found 514.2440

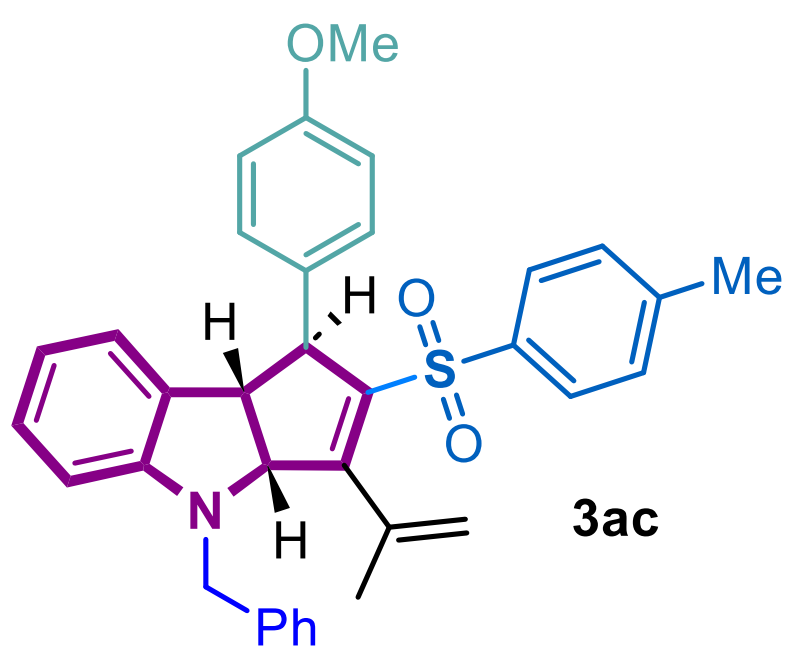

3ac

(1S)-4-benzyl-1-(4-methoxyphenyl)-3-(prop-1-en-2-yl)-2-tosyl-1,3a,4,8b-tetrahydrocyclopenta[b]indole (**3ac**): Prepared according to GP-3 on 0.3 mmol (110 mg)
Orange solid (101 mg, 60 % yield)
Rf = 0.35 (30% EA/hexane)
Melting point = 115-117°C
NMR Spectroscopy:
^{1}H NMR (400 MHz, CHLOROFORM-D) δ 7.23 (dd, *J* = 12.5, 5.1 Hz, 3H), 7.18 – 7.12 (m, 5H), 7.08 (t, *J* = 7.6 Hz, 1H), 6.95 (dd, *J* = 12.5, 8.4 Hz, 4H), 6.72 (dd, *J* = 7.6, 4.6 Hz, 3H), 6.47 (d, *J* = 7.9 Hz, 1H), 5.22 (t, *J* = 1.8 Hz, 1H), 4.99 (dd, *J* = 8.7, 1.8 Hz, 1H), 4.94 (s, 1H), 4.68 (d, *J* = 16.1 Hz, 1H), 4.50 (s, 1H), 4.39 (d, *J* = 16.3 Hz, 1H), 3.85 (d, *J* = 8.7 Hz, 1H), 3.79 (s, 3H), 2.31 (s, 3H), 1.95 (s, 3H) ppm.
^{13}C NMR (101 MHz, CHLOROFORM-D) δ 158.4, 154.7, 149.2, 143.3, 141.5, 138.9, 138.0, 137.9, 133.9, 130.6, 128.6, 128.5, 128.4, 127.7, 127.1, 127.0, 124.2, 117.9, 116.4, 113.8, 107.6, 75.6, 58.7, 55.1, 53.6, 51.1, 22.9, 21.3 ppm.
HRMS (ESI) m/z: [M+H]+ Calcd for $C_{35}H_{34}NO_3S^+$ 548.2259; Found 548.2236.

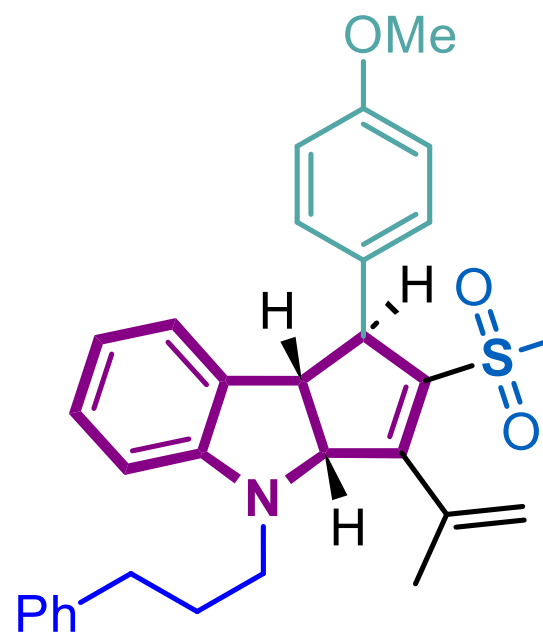

3ad

(1S)-1-(4-methoxyphenyl)-4-(3-phenylpropyl)-3-(prop-1-en-2-yl)-2-tosyl-1,3a,4,8b-tetrahydrocyclopenta[b]indole (**3ad**): Prepared according to GP-3 on 0.3 mmol (110 mg)
Yellow solid (75 mg, 42% yield)
Rf = 0.30 (40% EA/hexane)
Melting point = 105-107°C
NMR Spectroscopy:
^{1}H NMR (400 MHz, CHLOROFORM-D) δ 7.28 (s, 1H), 7.23 – 7.07 (m, 7H), 7.02 – 6.94 (m, 4H), 6.76 (d, *J* = 8.6 Hz, 2H), 6.71 (t, *J* = 7.3 Hz, 1H), 6.42 (d, *J* = 7.8 Hz, 1H), 5.22 – 5.14 (m, 1H), 4.94 (dd, *J* = 8.6, 1.8 Hz, 1H), 4.88 (s, 1H), 4.48 (s, 1H), 3.83 (s, 3H), 3.50 – 3.36 (m, 1H), 3.23 (dt, *J* = 14.9, 7.6 Hz, 1H), 2.61 (t, *J* = 7.6 Hz, 2H), 2.34 (s, 3H), 1.92 (s, 3H), 1.91 – 1.85 (m, 2H) ppm.
^{13}C NMR (101 MHz, CHLOROFORM-D) δ 158.7, 155.2, 149.1, 143.6, 141.7, 141.6, 139.5, 138.4, 134.4, 131.4, 128.9, 128.8, 128.7, 128.5, 128.4, 128.0, 126.1, 124.4, 118.1, 116.3, 114.1, 108.1, 58.8, 55.5, 54.0, 47.9, 33.3, 28.6, 23.3, 21.6 ppm.
HRMS (ESI) m/z: [M+H]+ Calcd for $C_{37}H_{38}NO_3S^+$ 576.2572; Found 576.2554.

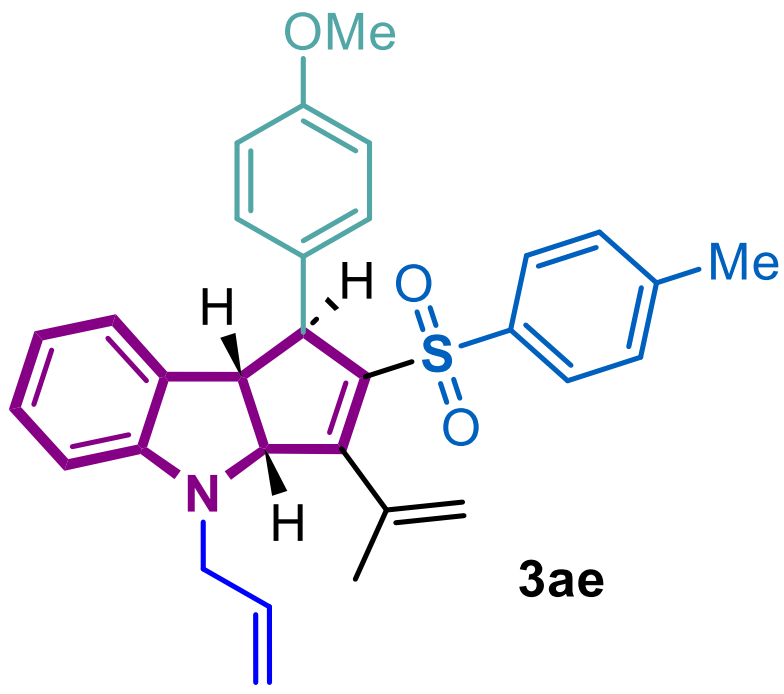

3ae

(1S)-4-allyl-1-(4-methoxyphenyl)-3-(prop-1-en-2-yl)-2-tosyl-1,3a,4,8b-tetrahydrocyclopenta[b]indole (**3ae**): Prepared according to GP-3 on 0.3 mmol (110 mg)
Light yellow crystal (110 mg, 72% yield)
Rf = 0.33 (40% EA/hexane)
Melting point = 187-189°C
NMR Spectroscopy:
^{1}H NMR (400 MHz, CHLOROFORM-D) 8.98 (s, 1H), 7.94 (d, *J* = 8.3 Hz, 2H), 7.86 – 7.82 (m, 1H), 7.45 – 7.40 (m, 1H), 7.38 (dd, *J* = 6.1, 3.2 Hz, 2H), 7.28 (d, *J* = 8.0 Hz, 2H), 3.91 (s, 3H), 2.39 (s, 3H), 2.27 – 2.16 (m, 2H), 2.12 – 2.01 (m, 2H), 0.39 (t, *J* = 7.5 Hz, 6H) ppm.
^{13}C NMR (101 MHz, CHLOROFORM-D) δ 196.1, 151.3, 145.3, 143.6, 139.3, 138.3, 129.3, 128.3, 126.2, 124.7, 123.9, 123.2, 118.3, 110.1, 109.8, 59.6, 33.5, 32.3, 21.7, 9.2 ppm.
HRMS (ESI) m/z: [M+H]+ Calcd for $C_{24}H_{26}NO_3S^+$ 498.2103; Found 498.2112.

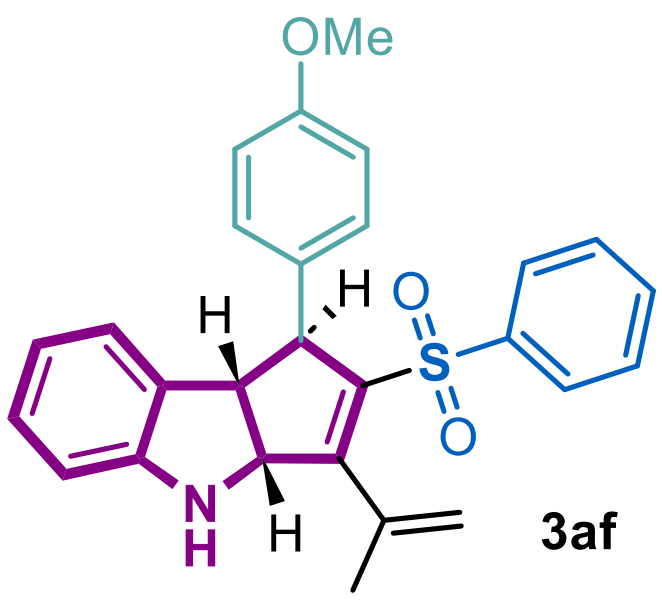

3af

(1S)-1-(4-methoxyphenyl)-2-(phenylsulfonyl)-3-(prop-1-en-2-yl)-1,3a,4,8b-tetrahydrocyclopenta[b]indole (**3af**): Prepared according to GP-3 on 0.3 mmol (103 mg)
Brown solid (125 mg, 95% yield)
Rf = 0.36 (40% EA/hexane)
Melting point = 263-265°C
NMR Spectroscopy:
^{1}H NMR (400 MHz, CHLOROFORM-D) δ 7.41 (s, 1H), 7.32 (dd, *J* = 8.3, 1.4 Hz, 2H), 7.21 (dd, *J* = 8.3, 7.3 Hz, 3H), 7.12 – 7.01 (m, 3H), 6.84 – 6.76 (m, 3H), 6.64 (d, *J* = 7.8 Hz, 1H), 5.27 (t, *J* = 1.8 Hz, 1H), 5.00 (dd, *J* = 7.8, 1.8 Hz, 1H), 4.81 (t, *J* = 1.3 Hz, 1H), 4.46 (t, *J* = 1.6 Hz, 1H), 4.14 (s, 1H), 3.82 (d, *J* = 0.9 Hz, 3H), 3.79 (d, *J* = 7.7 Hz, 1H), 1.88 (s, 3H) ppm.
^{13}C NMR (101 MHz, CHLOROFORM-D) δ 158.5, 154.7, 148.1, 141.3, 140.8, 136.9, 133.5, 132.6, 130.3, 128.4, 128.1, 127.5, 124.3, 119.6, 117.8, 113.9, 110.1, 70.2, 59.1, 55.2, 53.6, 22.2 ppm.

HRMS (ESI) m/z: [M+H]+ Calcd for $C_{27}H_{26}NO_3S^+$ 444.1628; Found 444.1626.

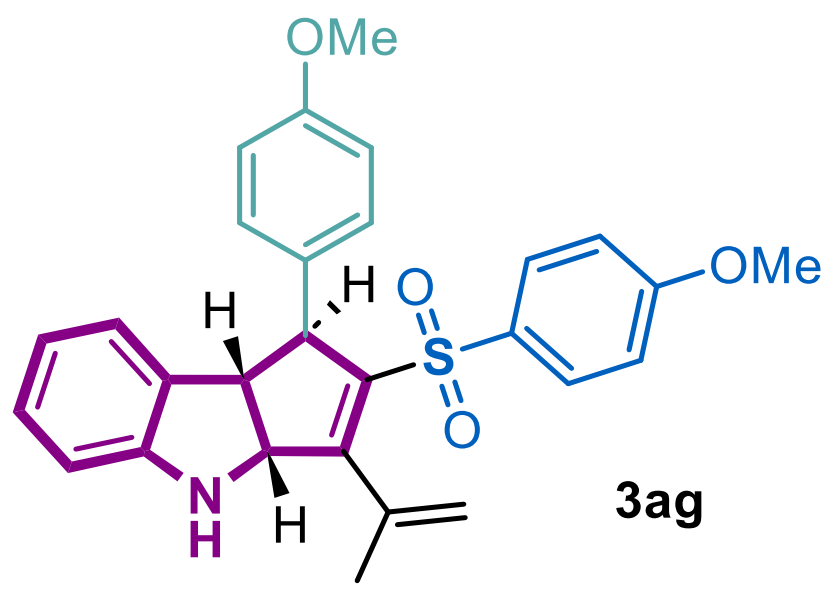

3ag

(1S)-1-(4-methoxyphenyl)-2-((4-methoxyphenyl)sulfonyl)-3-(prop-1-en-2-yl)-1,3a,4,8b-tetrahydrocyclopenta[b]indole (**3ag**): Prepared according to GP-3 on 0.3 mmol (112 mg)
Brown solid (100 mg, 72% yield)
Rf = 0.33 (40% EA/hexane)
Melting point = 158-160°C
NMR Spectroscopy:
^{1}H NMR (400 MHz, CHLOROFORM-D) δ 7.24 (d, *J* = 9.0 Hz, 2H), 7.20 (d, *J* = 7.4 Hz, 1H), 7.07 (dd, *J* = 11.0, 4.2 Hz, 1H), 7.02 (d, *J* = 8.6 Hz, 2H), 6.83 – 6.77 (m, 3H), 6.70 – 6.62 (m, 3H), 5.30 – 5.25 (m, 1H), 4.99 (dd, *J* = 7.7, 1.8 Hz, 1H), 4.85 (s, 1H), 4.43 (s, 1H), 3.81 (s, 3H), 3.79 (s, 3H), 3.76 (d, *J* = 7.8 Hz, 1H), 1.90 (s, 3H) ppm.
^{13}C NMR (101 MHz, CHLOROFORM-D) δ 163.1, 158.7, 154.0, 148.4, 142.0, 137.4, 134.0, 132.7, 130.6, 130.1, 128.7, 128.6, 124.6, 119.9, 117.9, 114.2, 113.5, 110.4, 70.5, 59.4, 55.6, 55.6, 55.4, 55.4, 53.9, 22.6ppm.
HRMS (ESI) m/z: [M+H]+ Calcd for $C_{28}H_{28}NO_4S^+$ 474.1739; Found 474.1762.

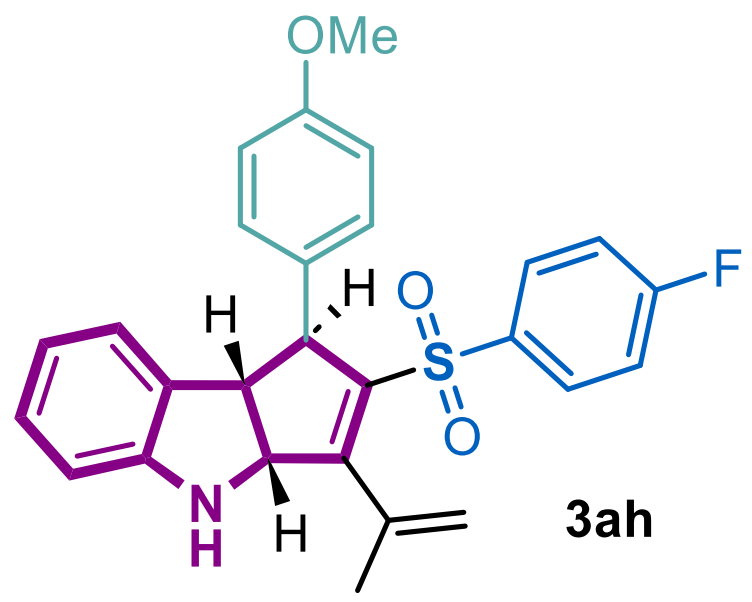

3ah

(1S)-2-((4-fluorophenyl)sulfonyl)-1-(4-methoxyphenyl)-3-(prop-1-en-2-yl)-1,3a,4,8b-tetrahydrocyclopenta[b]indole (**3ah**): Prepared according to GP-3 on 0.3 mmol (108 mg)
Brown solid (40 mg, 29% yield)
Rf = 0.34 (30% EA/hexane)
Melting point = 263-265°C
NMR Spectroscopy:
^{1}H NMR (400 MHz, CHLOROFORM-D) δ 7.30 (dd, *J* = 9.0, 5.1 Hz, 2H), 7.19 (d, *J* = 7.4 Hz, 1H), 7.07 (dd, *J* = 11.9, 4.3 Hz, 1H), 7.01 (d, *J* = 8.7 Hz, 2H), 6.86 (t, *J* = 8.6 Hz, 2H), 6.82 – 6.76 (m,

3H), 6.64 (d, *J* = 7.8 Hz, 1H), 5.30 – 5.24 (m, 1H), 5.00 (dd, *J* = 7.7, 1.8 Hz, 1H), 4.82 (s, 1H), 4.42 (s, 1H), 4.13 (s, 1H), 3.80 (s, 3H), 3.78 (dd, *J* = 7.8, 0.6 Hz, 1H), 1.91 (s, 3H) ppm.

^{13}C NMR (101 MHz, CHLOROFORM-D) δ 166.5-164.0 (d, *J* = 255.5 Hz), 158.8, 155.2, 148.3, 141.5, 137.4, 137.2, 133.7, 130.7-130.6 (d, *J* = 9.6 Hz), 130.5, 128.7, 128.7, 124.6, 119.9, 118.0, 115.7-115.5 (d, J = 22.4 Hz), 114.3, 110.4, 70.7, 59.4, 55.5, 53.9, 22.6 ppm.

^{19}F NMR (376 MHz, CHLOROFORM-D) δ -104.69 ppm.

HRMS (ESI) m/z: [M+H]+ Calcd for $C_{27}H_{25}FNO_3S^+$ 462.1534; Found 462.1526.

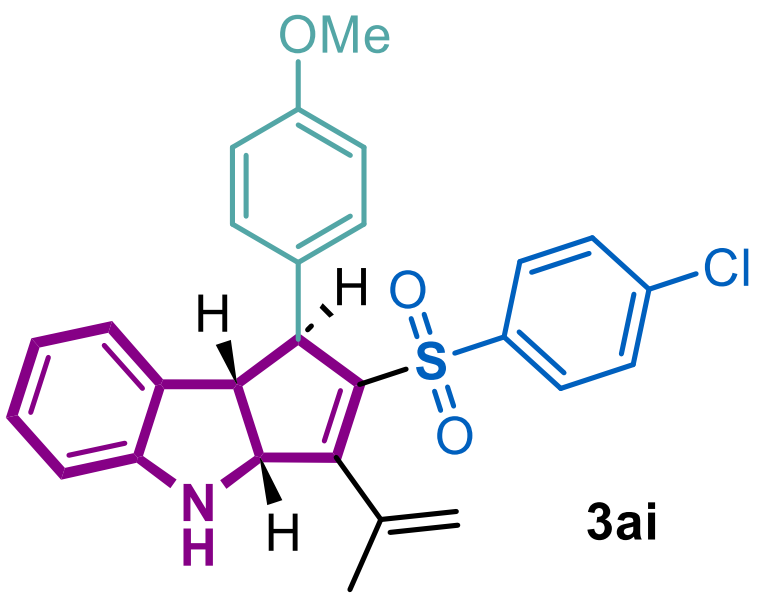

3ai

(1S)-2-((4-chlorophenyl)sulfonyl)-1-(4-methoxyphenyl)-3-(prop-1-en-2-yl)-1,3a,4,8b-tetrahydrocyclopenta[b]indole (**3ai**): Prepared according to GP-3 on 0.3 mmol (115 mg)

Brown solid (90 mg, 65% yield)

Rf = 0.32 (30% EA/hexane)

Melting point = 263-265°C

NMR Spectroscopy:

^{1}H NMR (400 MHz, CHLOROFORM-D) δ 7.25 – 7.14 (m, 5H), 7.09 (t, *J* = 7.6 Hz, 1H), 7.02 – 6.97 (m, 2H), 6.84 – 6.77 (m, 3H), 6.65 (d, *J* = 7.8 Hz, 1H), 5.33 – 5.27 (m, 1H), 5.02 (dd, *J* = 7.8, 1.9 Hz, 1H), 4.84 (t, *J* = 1.4 Hz, 1H), 4.43 (t, *J* = 1.6 Hz, 1H), 4.15 (s, 1H), 3.82 (s, 3H), 3.79 (d, *J* = 7.8 Hz, 1H), 1.93 (t, *J* = 1.4 Hz, 3H) ppm.

^{13}C NMR (101 MHz, CHLOROFORM-D) δ 158.9, 155.6, 148.3, 141.3, 139.6, 139.5, 137.4, 133.6, 130.5, 129.3, 128.8, 128.7, 128.6, 124.6, 120.0, 118.1, 114.3, 110.4, 70.8, 59.4, 55.5, 53.9, 22.7 ppm.

HRMS (ESI) m/z: [M+H]+ Calcd for $C_{27}H_{25}ClNO_3S^+$ 478.1238; Found 478.1232.

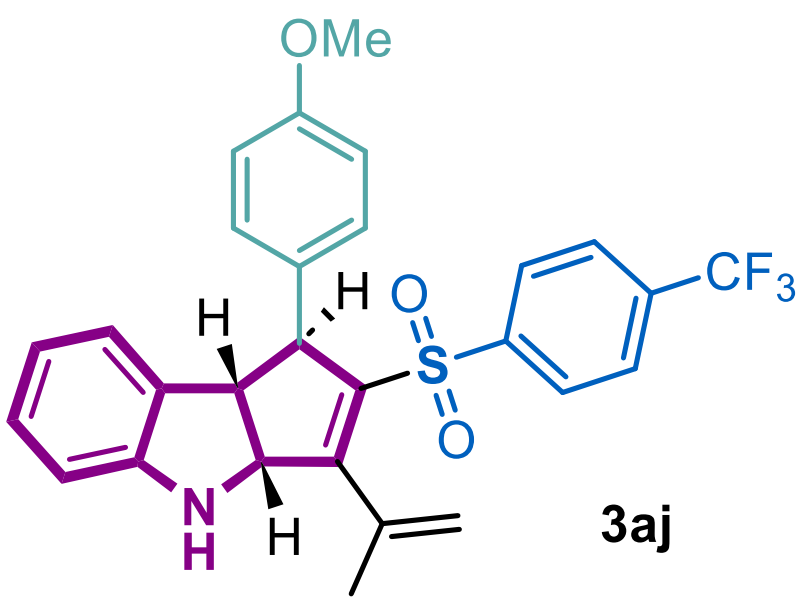

3aj

(1S)-1-(4-methoxyphenyl)-3-(prop-1-en-2-yl)-2-((4-(trifluoromethyl)phenyl)sulfonyl)-1,3a,4,8b-tetrahydrocyclopenta[b]indole (**3aj**): Prepared according to GP-3 on 0.3 mmol (123 mg)

Brown solid (90 mg, 57% yield)

Rf = 0.28 (30% EA/hexane)

Melting point = 263-265°C

NMR Spectroscopy:

^{1}H NMR (400 MHz, CHLOROFORM-D) δ 7.44 (s, 4H), 7.19 (d, *J* = 7.4 Hz, 1H), 7.10 (t, *J* = 7.6 Hz, 1H), 6.97 (d, *J* = 8.7 Hz, 2H), 6.86 – 6.78 (m, 1H), 6.75 (d, *J* = 8.7 Hz, 2H), 6.66 (d, *J* = 7.8 Hz, 1H), 5.32 (t, *J* = 1.5 Hz, 1H), 5.03 (dd, *J* = 7.8, 1.8 Hz, 1H), 4.85 (s, 1H), 4.43 (s, 1H), 4.16 (s, 1H), 3.81 (s, 1H), 3.79 (s, 3H), 1.97 (s, 3H) ppm.

^{13}C NMR (101 MHz, CHLOROFORM-D) δ 158.9, 156.5, 148.3, 144.6, 140.7, 137.5, 134.5, 134.2, 133.3, 130.3, 128.8, 128.7, 128.3, 125.4, 125.4, 124.6, 121.8, 120.0, 117.9, 114.3, 110.4, 70.9, 59.4, 55.3, 53.9, 22.9 ppm.

^{19}F NMR (376 MHz, CHLOROFORM-D) δ -63.15 ppm.

HRMS (ESI) m/z: [M+H]+ Calcd for $C_{28}H_{25}F_3NO_3S^+$ 512.1502; Found 512.1508.

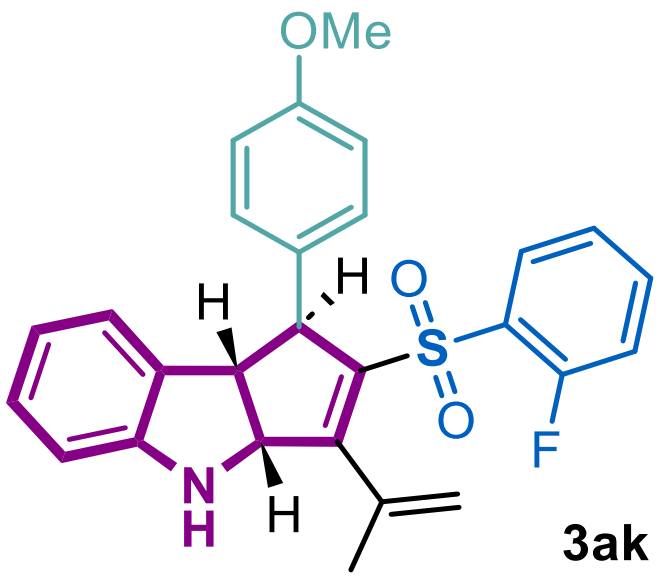

3ak

(1S)-2-((2-fluorophenyl)sulfonyl)-1-(4-methoxyphenyl)-3-(prop-1-en-2-yl)-1,3a,4,8b-tetrahydrocyclopenta[b]indole (**3ak**): Prepared according to GP-3 on 0.3 mmol (110 mg) Brown solid (110 mg, 78 % yield)

Rf = 0.31 (30% EA/hexane)

Melting point = 263-265°C

NMR Spectroscopy:

^{1}H NMR (400 MHz, CHLOROFORM-D) δ 7.60 – 7.51 (m, 1H), 7.38 (tdd, *J* = 7.4, 4.9, 1.7 Hz, 1H), 7.20 (d, *J* = 7.4 Hz, 1H), 7.11 – 6.98 (m, 4H), 6.88 – 6.76 (m, 2H), 6.77 – 6.70 (m, 2H), 6.63 (d, *J* = 7.8 Hz, 1H), 5.22 – 5.11 (m, 1H), 5.04 (dd, *J* = 7.6, 1.8 Hz, 1H), 4.56 (s, 1H), 4.44 (s, 1H), 3.80 (d, *J* = 7.7 Hz, 1H), 3.76 (s, 3H), 2.04 (s, 3H) ppm.

^{13}C NMR (101 MHz, CHLOROFORM-D) δ 160.7-158.2 (d, J = 255 Hz), 158.6, 156.5, 148.4, 139.7, 137.0, 135.4-135.3 (d, *J* = 8.4 Hz), 133.7, 130.5, 130.3, 129.3-129.2 (d, *J* = 13.6 Hz), 128.6, 128.3, 124.6, 123.9-123.9 (d, *J* = 3.8 Hz), 119.9, 117.6, 116.7-116.5 (d, *J* = 20.6 Hz), 114.3, 110.3, 69.9, 59.1, 55.4, 54.4, 22.5 ppm.

^{19}F NMR (376 MHz, CHLOROFORM-D) δ -107.50 ppm.

HRMS (ESI) m/z: [M+H]+ Calcd for $C_{27}H_{25}FNO_3S^+$ 462.1539; Found 462.1547.

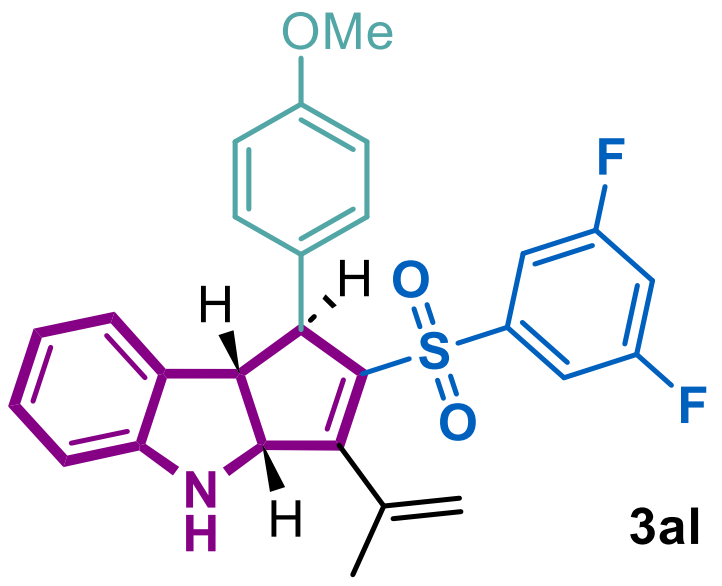

3al

(1S)-2-((3,5-difluorophenyl)sulfonyl)-1-(4-methoxyphenyl)-3-(prop-1-en-2-yl)-1,3a,4,8b-tetrahydrocyclopenta[b]indole (**3al**): Prepared according to GP-3 on 0.3 mmol (115 mg)
Brown solid (105 mg, 76% yield)
Rf = 0.30 (30% EA/hexane)
Melting point = 263-265°C
NMR Spectroscopy:
^{1}H NMR (400 MHz, CHLOROFORM-D) δ 7.21 (d, *J* = 7.4 Hz, 1H), 7.10 (t, *J* = 7.6 Hz, 1H), 7.04 – 6.99 (m, 2H), 6.88 – 6.76 (m, 6H), 6.67 (d, *J* = 7.8 Hz, 1H), 5.34 (t, *J* = 1.5 Hz, 1H), 5.06 (dd, *J* = 7.9, 1.9 Hz, 1H), 4.89 (s, 1H), 4.48 – 4.36 (m, 1H), 4.17 (s, 1H), 3.85 (d, *J* = 7.6 Hz, 1H), 3.82 (s, 3H), 1.98 (d, *J* = 1.0 Hz, 3H) ppm.
^{13}C NMR (101 MHz, CHLOROFORM-D) δ 163.5, 163.4, 161.0, 160.9, 159.1, 156.8, 148.2, 144.6, 140.8, 137.4, 133.2, 130.4, 128.9, 124.6, 120.1, 118.2, 114.4, 111.4, 111.1, 110.5, 108.5, 108.3, 71.3, 71.2, 59.5, 55.5, 55.4, 53.8, 22.8 ppm.
^{19}F NMR (376 MHz, CHLOROFORM-D) δ -106.42 ppm.
HRMS (ESI) m/z: [M+H]+ Calcd for $C_{27}H_{24}F_2NO_3S^+$ 480.1439; Found 480.1425.

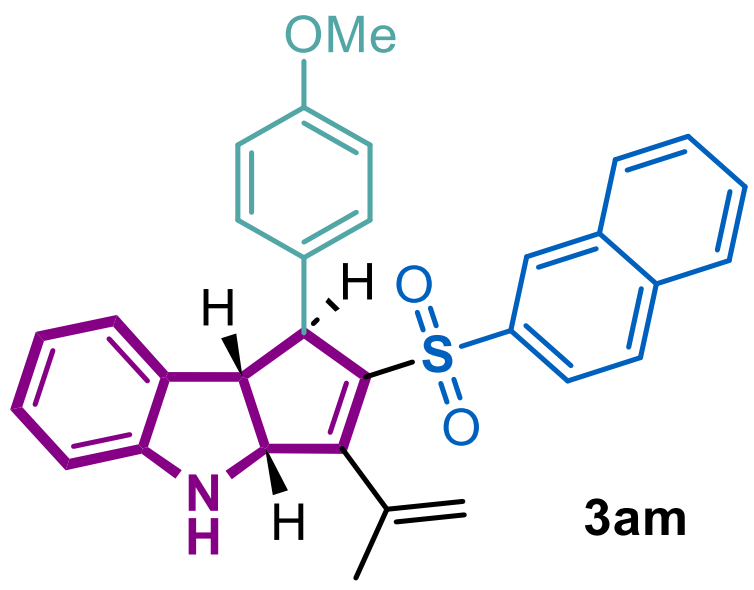

3am

(1S)-1-(4-methoxyphenyl)-2-(naphthalen-2-ylsulfonyl)-3-(prop-1-en-2-yl)-1,3a,4,8b-tetrahydrocyclopenta[b]indole (**3am**): Prepared according to GP-3 on 0.3 mmol (120 mg)
Brown solid (125 mg, 83% yield)
Rf = 0.33 (30% EA/hexane)
Melting point = 138-140°C
NMR Spectroscopy:
^{1}H NMR (400 MHz, CHLOROFORM-D) δ 7.81 (d, *J* = 7.6 Hz, 1H), 7.72 (d, *J* = 1.7 Hz, 1H), 7.70 – 7.63 (m, 2H), 7.60 (ddd, *J* = 8.2, 6.9, 1.4 Hz, 1H), 7.52 (ddd, *J* = 8.1, 6.9, 1.2 Hz, 1H), 7.39 (dd, *J* = 8.7, 1.9 Hz, 1H), 7.21 (d, *J* = 7.4 Hz, 1H), 7.12 – 7.05 (m, 1H), 7.01 – 6.95 (m, 2H), 6.80 (td, *J* = 7.4, 0.9 Hz, 1H), 6.71 – 6.57 (m, 3H), 5.32 (t, *J* = 1.6 Hz, 1H), 5.01 (dd, *J* = 7.8, 1.7 Hz, 1H), 4.95 – 4.81 (m, 1H), 4.51 (s, 1H), 3.79 (d, *J* = 7.2 Hz, 1H), 3.65 (s, 3H), 1.91 (s, 3H) ppm.

^{13}C NMR (101 MHz, CHLOROFORM-D) δ 158.6, 155.2, 148.4, 141.6, 138.0, 137.4, 134.9, 133.7, 131.8, 130.6, 129.9, 129.5, 128.9, 128.8, 128.6, 127.8, 127.2, 124.7, 122.5, 120.0, 118.2, 114.1, 110.5, 70.9, 70.8, 59.7, 55.3, 55.2, 53.9, 22.7 ppm.

HRMS (ESI) m/z: [M+H]+ Calcd for $C_{31}H_{28}NO_3S^+$ 494.1789; Found 494.1792.

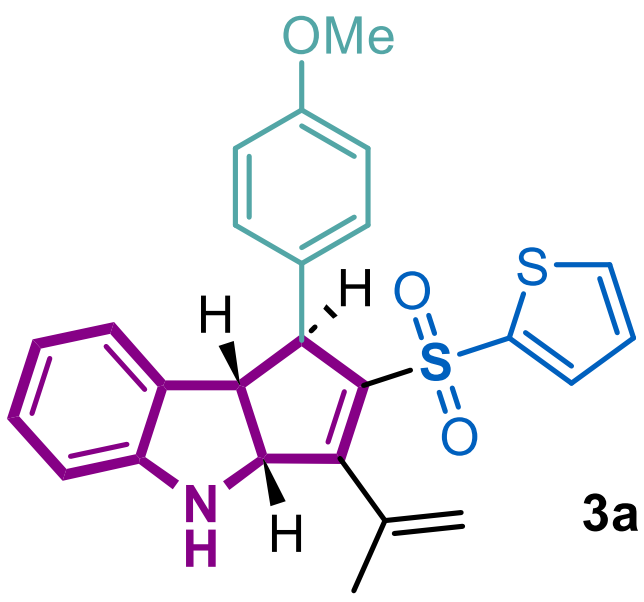

3an

(1S)-1-(4-methoxyphenyl)-3-(prop-1-en-2-yl)-2-(thiophen-2-ylsulfonyl)-1,3a,4,8b-tetrahydrocyclopenta[b]indole (**3an**): Prepared according to GP-3 on 0.3 mmol (110 mg)

Brown Thick Liquid (75 mg, 82% yield)

Rf = 0.28 (30% EA/hexane)

NMR Spectroscopy:

^{1}H NMR (400 MHz, CHLOROFORM-D) δ 7.45 (dd, *J* = 5.0, 1.3 Hz, 1H), 7.20 (d, *J* = 7.5 Hz, 1H), 7.11 – 7.04 (m, 3H), 7.00 (dd, *J* = 3.7, 1.3 Hz, 1H), 6.86 – 6.75 (m, 4H), 6.65 (d, *J* = 7.8 Hz, 1H), 5.31 (dd, *J* = 3.7, 2.2 Hz, 1H), 5.02 (dd, *J* = 7.7, 1.8 Hz, 1H), 4.88 (s, 1H), 4.47 (s, 1H), 4.17 (s, 1H), 3.81 (s, 3H), 3.80 (d, *J* = 8.0 Hz, 1H), 1.98 (s, 3H) ppm.

^{13}C NMR (101 MHz, CHLOROFORM-D) δ 158.8, 155.3, 148.4, 141.3, 137.1, 133.9, 133.6, 130.5, 128.7, 128.6, 127.1, 124.6, 119.9, 118.0, 114.3, 110.4, 70.5, 59.3, 55.5, 54.0, 22.7 ppm.

HRMS (ESI) m/z: [M+H]+ Calcd for $C_{25}H_{24}NO_3S_2^+$ 450.1192; Found 450.1189.

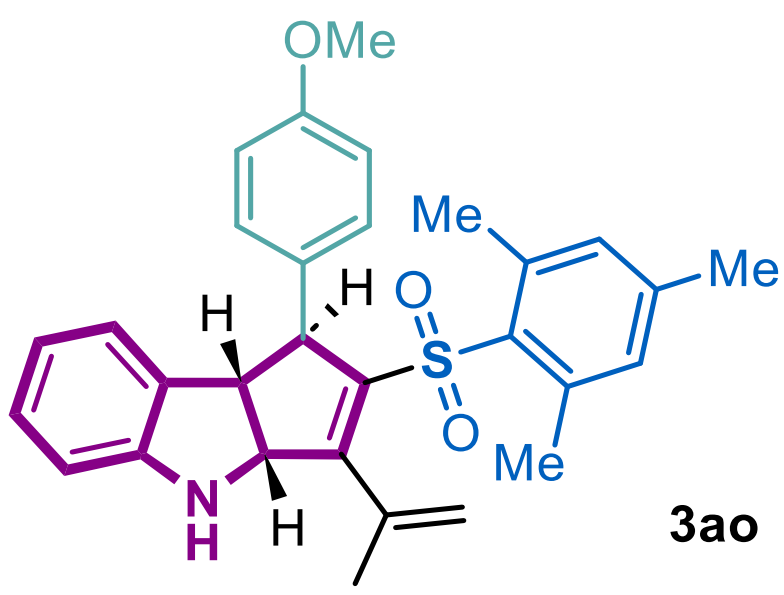

3ao

(1S)-2-(mesitylsulfonyl)-1-(4-methoxyphenyl)-3-(prop-1-en-2-yl)-1,3a,4,8b-tetrahydrocyclopenta[b]indole (**3ao**): Prepared according to GP-3 on 0.3 mmol (118 mg)

Brown solid (130 mg, 88% yield)

Rf = 0.31 (30% EA/hexane)

Melting point = 263-265°C

NMR Spectroscopy:

¹H NMR (400 MHz, CHLOROFORM-D) δ 7.19 – 7.05 (m, 4H), 6.90 – 6.85 (m, 2H), 6.79 (t, *J* = 7.3 Hz, 1H), 6.73 (s, 2H), 6.64 (d, *J* = 7.8 Hz, 1H), 5.06 – 4.92 (m, 2H), 4.35 (s, 1H), 4.19 (d, *J* = 3.2 Hz, 2H), 3.81 (s, 3H), 3.77 (d, *J* = 7.3 Hz, 1H), 2.24 (s, 3H), 2.13 (s, 6H), 1.97 (s, 3H) ppm.
¹³C NMR (101 MHz, CHLOROFORM-D) δ 158.5, 151.5, 148.3, 142.6, 142.5, 139.9, 137.0, 134.6, 133.2, 131.3, 130.5, 128.4, 128.3, 124.2, 119.5, 116.3, 113.9, 110.1, 70.0, 59.2, 55.1, 53.9, 22.2, 21.5, 20.8 ppm.
HRMS (ESI) m/z: [M+H]+ Calcd for $C_{30}H_{32}NO_3S^+$ 486.6450; Found 486.6460.

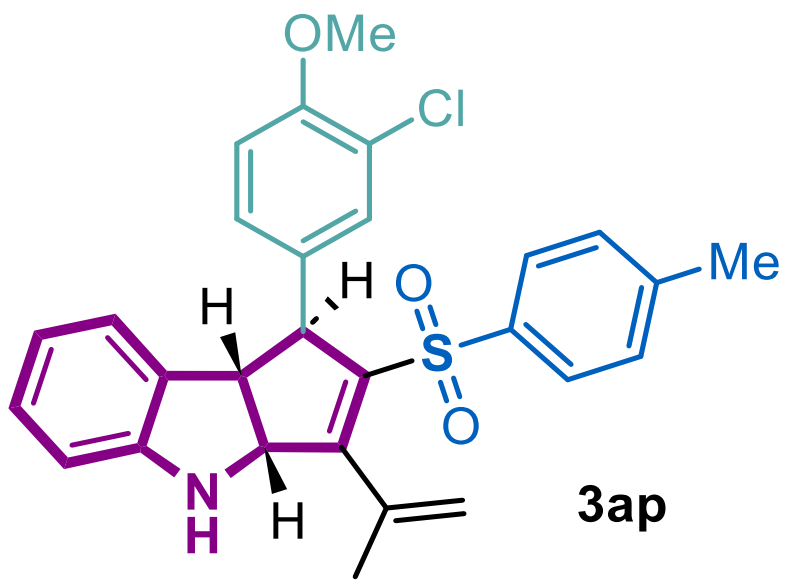

3ap

(1S)-1-(3-chloro-4-methoxyphenyl)-3-(prop-1-en-2-yl)-2-tosyl-1,3a,4,8b-tetrahydrocyclopenta[b]indole (**3ap**): Prepared according to GP-3 on 0.3 mmol (120 mg)
Brown solid (96 mg, 42% yield)
Rf = 0.32 (30% EA/hexane)
Melting point = 263-265°C
NMR Spectroscopy:
¹H NMR (400 MHz, CHLOROFORM-D) δ 7.24 (d, *J* = 8.2 Hz, 2H), 7.18 (d, *J* = 7.4 Hz, 1H), 7.11 – 6.99 (m, 4H), 6.88 (d, *J* = 2.2 Hz, 1H), 6.85 – 6.76 (m, 2H), 6.64 (d, *J* = 7.8 Hz, 1H), 5.29 (d, *J* = 1.3 Hz, 1H), 4.98 (dd, *J* = 7.8, 1.8 Hz, 1H), 4.86 (s, 1H), 4.38 (s, 1H), 4.20 (s, 1H), 3.90 (s, 3H), 3.74 (d, *J* = 7.8 Hz, 1H), 2.34 (s, 3H), 1.93 (s, 3H) ppm.
¹³C NMR (101 MHz, CHLOROFORM-D) δ 158.7, 155.2, 149.1, 143.6, 141.7, 141.6, 139.5, 138.4, 134.4, 131.4, 128.9, 128.8, 128.7, 128.5, 128.4, 128.0, 126.1, 124.4, 118.1, 116.3, 114.1, 108.1, 58.8, 55.5, 54.0, 47.9, 33.3, 28.6, 23.3, 21.6 ppm.
HRMS (ESI) m/z: [M+H]+ Calcd for $C_{28}H_{27}ClNO_3S^+$ 492.1400; Found 492.1408.

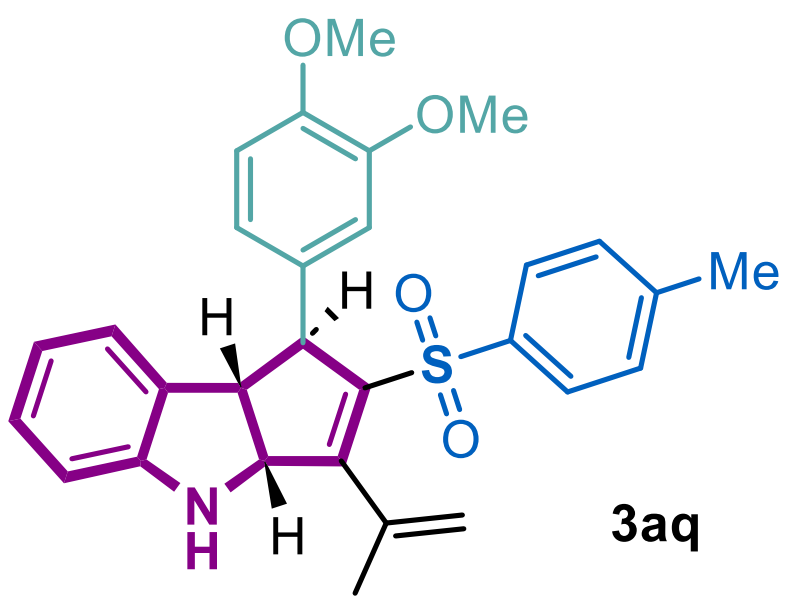

3aq

(1S)-1-(3,4-dimethoxyphenyl)-3-(prop-1-en-2-yl)-2-tosyl-1,3a,4,8b-tetrahydrocyclopenta[b]indole (**3aq**): Prepared according to GP-3 on 0.3 mmol (118mg)
Brown solid (125 mg, 84 % yield)
Rf = 0.30 (30% EA/hexane)

Melting point = 263-265°C

NMR Spectroscopy:

^{1}H NMR (400 MHz, CHLOROFORM-D) δ 7.22 (dd, *J* = 7.6, 5.6 Hz, 3H), 7.08 (t, *J* = 7.6 Hz, 1H), 7.00 (d, *J* = 8.0 Hz, 2H), 6.84 – 6.74 (m, 2H), 6.69 (dd, *J* = 8.2, 2.0 Hz, 1H), 6.65 (d, *J* = 7.7 Hz, 1H), 6.52 (d, *J* = 2.0 Hz, 1H), 5.36 – 5.20 (m, 1H), 4.98 (dd, *J* = 7.7, 1.8 Hz, 1H), 4.84 (s, 1H), 4.44 (s, 1H), 4.14 (s, 1H), 3.89 (s, 3H), 3.78 (d, *J* = 7.5 Hz, 1H), 3.75 (s, *J* = 5.6 Hz, 3H), 2.33 (s, 3H), 1.91 (s, 3H) ppm.

^{13}C NMR (101 MHz, CHLOROFORM-D) δ 154.6, 149.1, 148.4, 148.2, 143.9, 141.8, 138.2, 137.3, 134.3, 130.5, 128.9, 128.7, 128.0, 124.7, 119.9, 119.8, 118.1, 111.5, 110.6, 110.5, 70.6, 59.8, 56.1, 55.7, 53.9, 29.8, 22.5, 21.6 ppm.

HRMS (ESI) m/z: [M+H]+ Calcd for $C_{29}H_{30}NO_4S^+$ 488.1890; Found 488.1894.

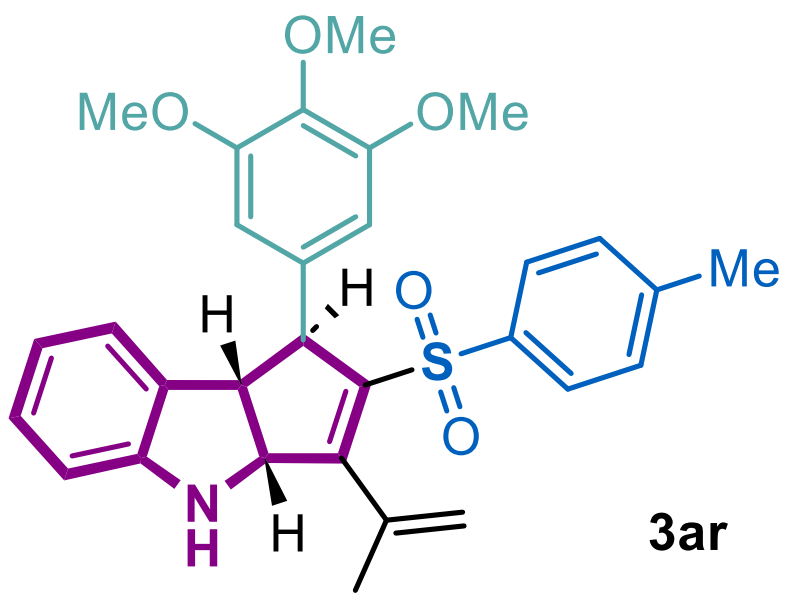

3ar

(1S)-3-(prop-1-en-2-yl)-2-tosyl-1-(3,4,5-trimethoxyphenyl)-1,3a,4,8b-tetrahydrocyclopenta[b]indole (**3ar**): Prepared according to GP-3 on 0.3 mmol (126 mg)

Off-white solid (136 mg, 87 % yield)

Rf = 0.28 (30% EA/hexane)

Melting point = 194-196°C

NMR Spectroscopy:

^{1}H NMR (400 MHz, CHLOROFORM-D) δ 7.23 (dd, *J* = 4.5, 2.7 Hz, 3H), 7.11 – 7.05 (m, 1H), 7.01 (d, *J* = 8.0 Hz, 2H), 6.80 (td, *J* = 7.4, 0.9 Hz, 1H), 6.64 (d, *J* = 7.7 Hz, 1H), 6.24 (s, 2H), 5.30 (t, *J* = 1.5 Hz, 1H), 4.95 (dd, *J* = 7.7, 1.8 Hz, 1H), 4.87 – 4.78 (m, 1H), 4.46 – 4.35 (m, 1H), 4.13 (s, 1H), 3.83 (s, 3H), 3.75 (d, *J* = 7.7 Hz, 1H), 3.73 (s, 6H), 2.31 (s, 3H), 1.93 (s, 3H) ppm.

^{13}C NMR (101 MHz, CHLOROFORM-D) δ 154.9, 153.4, 148.4, 144.1, 141.4, 138.2, 137.2, 136.9, 130.3, 128.9, 128.8, 128.0, 124.6, 119.9, 118.0, 110.5, 104.3, 70.7, 61.0, 60.4, 55.9, 53.8, 22.6, 21.6 ppm.

HRMS (ESI) m/z: [M+H]+ Calcd for $C_{30}H_{32}NO_5S^+$ 518.2001; Found 518.2014.

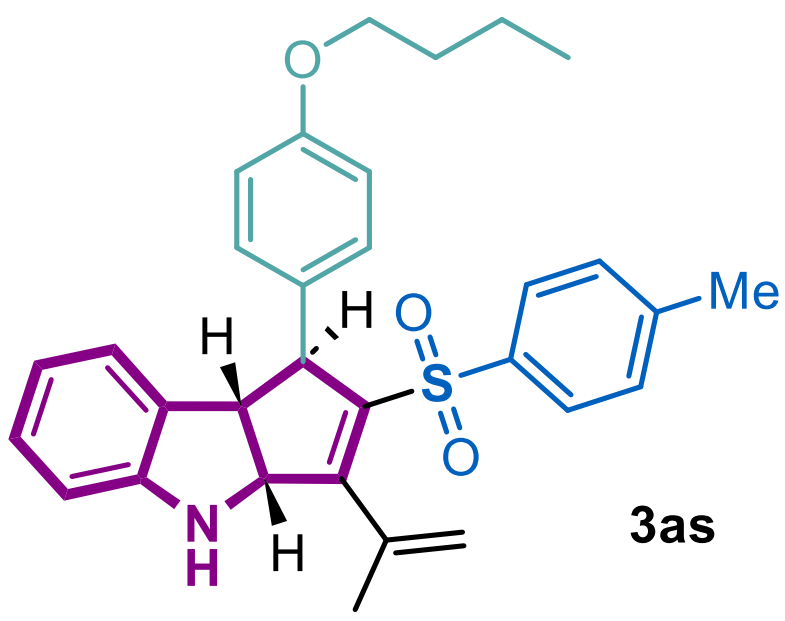

3as

(1S)-1-(4-butoxyphenyl)-3-(prop-1-en-2-yl)-2-tosyl-1,3a,4,8b-tetrahydrocyclopenta[b]indole (3as): Prepared according to GP-3 on 0.3 mmol (120 mg)
Brown solid (138 mg, 92% yield)
Rf = 0.30 (40% EA/hexane)
Melting point = 87-90°C
NMR Spectroscopy:
^{1}H NMR (400 MHz, CHLOROFORM-D) δ 7.20 (d, *J* = 8.2 Hz, 3H), 7.08 (t, *J* = 7.6 Hz, 1H), 7.00 (dd, *J* = 8.2, 6.1 Hz, 4H), 6.79 (t, *J* = 8.2 Hz, 3H), 6.64 (d, *J* = 7.8 Hz, 1H), 5.27 (s, 1H), 4.99 (d, *J* = 7.4 Hz, 1H), 4.83 (s, 1H), 4.43 (s, 1H), 4.14 (s, 1H), 4.03 – 3.88 (m, 2H), 3.77 (d, *J* = 7.6 Hz, 1H), 2.32 (s, 3H), 1.88 (s, 3H), 1.79 (dt, *J* = 14.5, 6.6 Hz, 2H), 1.52 (dq, *J* = 14.7, 7.4 Hz, 2H), 1.00 (t, *J* = 7.4 Hz, 3H) ppm.
^{13}C NMR (101 MHz, CHLOROFORM-D) δ 158.3, 154.5, 148.4, 143.7, 141.9, 138.2, 137.3, 133.7, 130.7, 129.0, 128.7, 127.9, 124.6, 119.9, 118.0, 114.8, 110.4, 70.5, 67.8, 59.5, 53.9, 31.4, 22.5, 21.6, 19.4, 14.0 ppm.
HRMS (ESI) m/z: [M+H]+ Calcd for $C_{31}H_{34}NO_3S^+$ 500.2254; Found 500.2259.

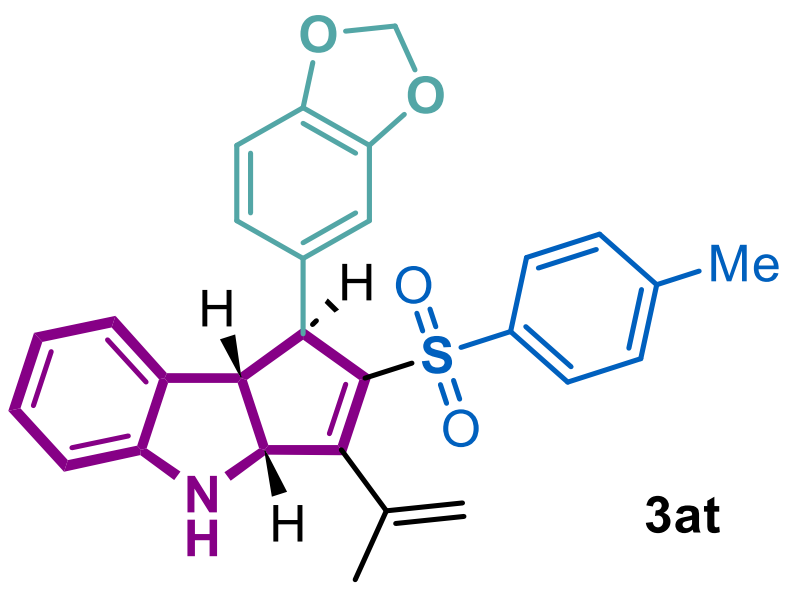

3at

(1S)-1-(benzo[d][1,3]dioxol-5-yl)-3-(prop-1-en-2-yl)-2-tosyl-1,3a,4,8b-tetrahydrocyclopenta[b]indole (3at): Prepared according to GP-3 on 0.3 mmol (112 mg)
Brown thick liquid (100 mg, 72% yield)
Rf = 0.30 (40% EA/hexane)
NMR Spectroscopy:
^{1}H NMR (400 MHz, CHLOROFORM-D) δ 7.33 – 7.23 (m, 2H), 7.17 (d, *J* = 7.2 Hz, 1H), 7.05 (dd, *J* = 19.3, 7.7 Hz, 3H), 6.78 (t, *J* = 7.3 Hz, 1H), 6.72 (d, *J* = 7.8 Hz, 1H), 6.68 – 6.59 (m, 2H), 6.44 (s, 1H), 5.93 (d, *J* = 8.8 Hz, 2H), 5.26 (s, 1H), 4.96 (d, *J* = 7.8 Hz, 1H), 4.81 (s, 1H), 4.38 (s, 1H), 4.14 (s, 1H), 3.74 (d, *J* = 7.8 Hz, 1H), 2.34 (s, 3H), 1.92 (s, 3H) ppm.
^{13}C NMR (101 MHz, CHLOROFORM-D) δ 154.6, 148.1, 147.7, 146.3, 143.6, 141.0, 137.8, 137.1, 135.4, 130.1, 128.7, 128.4, 127.6, 124.2, 120.7, 119.5, 117.4, 110.0, 108.2, 107.4, 100.8, 70.1, 59.6, 53.7, 22.4, 21.3 ppm.
HRMS (ESI) m/z: [M+H]+ Calcd for $C_{28}H_{26}NO_4S^+$ 472.1577; Found 472.1567.

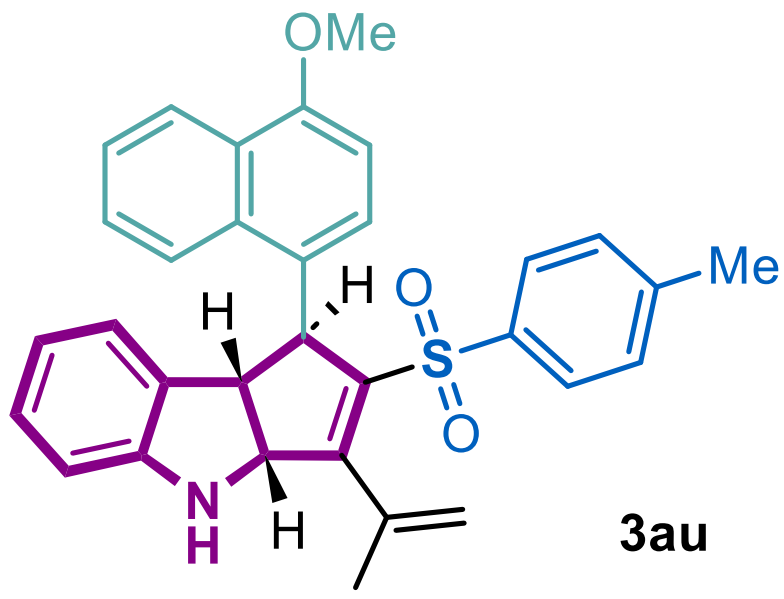

3au

(1S)-1-(4-methoxynaphthalen-1-yl)-3-(prop-1-en-2-yl)-2-tosyl-1,3a,4,8b-tetrahydrocyclopenta[b]indole (3au): Prepared according to GP-3 on 0.3 mmol (130 mg)
Brown thick liquid (60 mg, 44% yield)
Rf = 0.30 (40% EA/hexane)
NMR Spectroscopy:
^{1}H NMR (400 MHz, CHLOROFORM-D) δ 8.33 (d, *J* = 8.3 Hz, 1H), 8.17 (d, *J* = 8.4 Hz, 1H), 7.65 – 7.59 (m, 1H), 7.53 (t, *J* = 7.6 Hz, 1H), 7.37 (d, *J* = 7.4 Hz, 1H), 7.21 (d, *J* = 8.2 Hz, 2H), 7.14 – 7.01 (m, 2H), 6.84 (dd, *J* = 14.3, 7.6 Hz, 3H), 6.65 (t, *J* = 7.4 Hz, 2H), 5.27 (s, 1H), 5.08 (s, 1H), 4.93 – 4.81 (m, 1H), 4.69 (s, 1H), 4.23 (s, 1H), 3.98 (s, 3H), 3.76 (d, *J* = 7.2 Hz, 1H), 2.23 (s, 3H), 2.09 (s, 3H) ppm.
^{13}C NMR (101 MHz, CHLOROFORM-D) δ 155.5, 154.9, 148.9, 143.8, 140.8, 137.7, 131.9, 130.5, 129.2, 128.9, 128.7, 128.3, 127.8, 127.0, 126.1, 125.2, 124.4, 123.8, 122.9, 122.6, 119.8, 117.2, 110.4, 103.4, 69.8, 55.6, 54.1, 53.7, 23.0, 21.5 ppm.
HRMS (ESI) m/z: [M+H]+ Calcd for $C_{32}H_{30}NO_3S^+$ 508.1941; Found 508.1942.

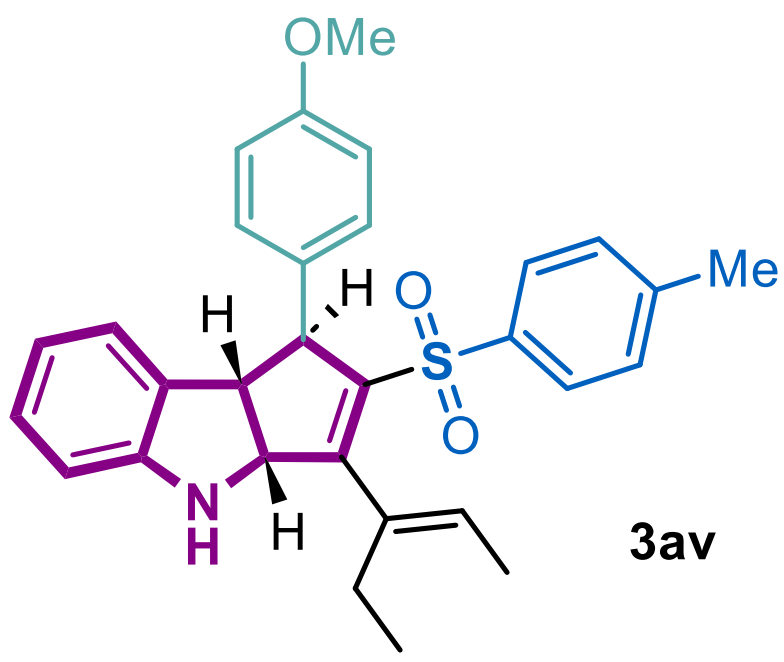

3av

(1S)-1-(4-methoxyphenyl)-3-((E)-pent-2-en-3-yl)-2-tosyl-1,3a,4,8b-tetrahydrocyclopenta[b]indole (3av): Prepared according to GP-3 on 0.3 mmol (130 mg)
Off-white solid (114 mg, 78% yield)
Rf = 0.35 (30% EA/hexane)
Melting point = 105-107°C
NMR Spectroscopy:
^{1}H NMR (400 MHz, CHLOROFORM-D) δ 7.23 (dd, *J* = 4.5, 2.7 Hz, 3H), 7.11 – 7.05 (m, 1H), 7.01 (d, *J* = 8.0 Hz, 2H), 6.80 (td, *J* = 7.4, 0.9 Hz, 1H), 6.64 (d, *J* = 7.7 Hz, 1H), 6.24 (s, 2H), 5.30 (t, *J* = 1.5 Hz, 1H), 4.95 (dd, *J* = 7.7, 1.8 Hz, 1H), 4.87 – 4.78 (m, 1H), 4.46 – 4.35 (m, 1H), 4.13 (s, 1H), 3.83 (s, 3H), 3.75 (d, *J* = 7.7 Hz, 1H), 3.73 (s, 6H), 2.31 (s, 3H), 1.93 (s, 3H) ppm.

^{13}C NMR (101 MHz, CHLOROFORM-D) δ 155.5, 154.9, 148.9, 143.8, 140.8, 137.7, 131.9, 130.5, 129.2, 128.9, 128.7, 128.3, 127.8, 127.0, 126.1, 125.2, 124.4, 123.8, 122.9, 122.6, 119.8, 117.2, 110.4, 103.4, 69.8, 55.6, 54.1, 53.7, 23.0, 21.5 ppm.
HRMS (ESI) m/z: [M+H]+ Calcd for $C_{30}H_{32}NO_3S^+$ 486.2103; Found 486.2137.

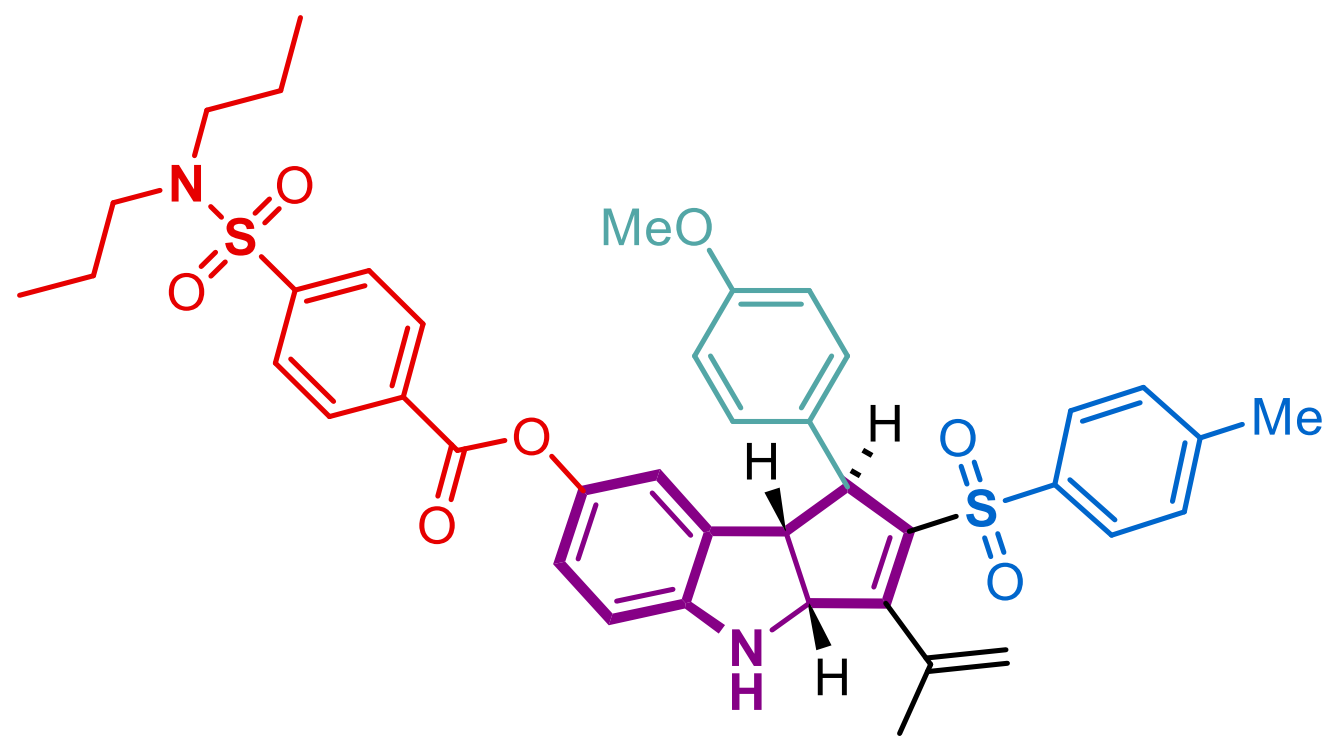

7a

(1S,8bR)-1-(4-methoxyphenyl)-3-(prop-1-en-2-yl)-2-tosyl-1,3a,4,8b-tetrahydrocyclopenta[b]indol-7-yl 4-(N,N-dipropylsulfamoyl)benzoate (**7a**): Prepared according to GP-3 on 0.3 mmol (110 mg)
Yellow solid (96 mg, 42% yield)
Rf = 0.30 (40% EA/hexane)
Melting point = 92-95°C
NMR Spectroscopy:
^{1}H NMR (400 MHz, CHLOROFORM-D) δ 8.36 – 8.28 (m, 2H), 7.95 (d, *J* = 8.5 Hz, 2H), 7.21 (d, *J* = 8.3 Hz, 2H), 7.08 (d, *J* = 1.2 Hz, 1H), 7.00 (t, *J* = 8.5 Hz, 4H), 6.93 (dd, *J* = 8.6, 2.3 Hz, 1H), 6.77 (d, *J* = 8.6 Hz, 2H), 6.66 (d, *J* = 8.4 Hz, 1H), 5.27 (s, 1H), 5.04 (dd, *J* = 7.7, 1.5 Hz, 1H), 4.83 (s, 1H), 4.42 (s, 1H), 4.17 (s, 1H), 3.81 (s, 1H), 3.80 (s, 3H), 3.31 – 2.89 (m, 4H), 2.32 (s, 3H), 1.89 (s, 3H), 1.56 (dt, *J* = 14.8, 7.4 Hz, 4H), 0.89 (t, *J* = 7.4 Hz, 6H) ppm.
^{13}C NMR (101 MHz, CHLOROFORM-D) δ 164.5, 158.8, 154.3, 146.6, 144.8, 143.9, 141.9, 138.1, 137.2, 133.6, 133.2, 132.0, 130.8, 129.1, 128.7, 127.9, 127.3, 121.5, 118.1, 114.3, 110.5, 71.1, 59.4, 55.5, 54.0, 50.0, 29.8, 22.5, 22.0, 21.6, 11.3 ppm.
HRMS (ESI) m/z: [M+H]+ Calcd for $C_{41}H_{45}N_2O_7S_2^+$ 741.2663; Found 741.2650.

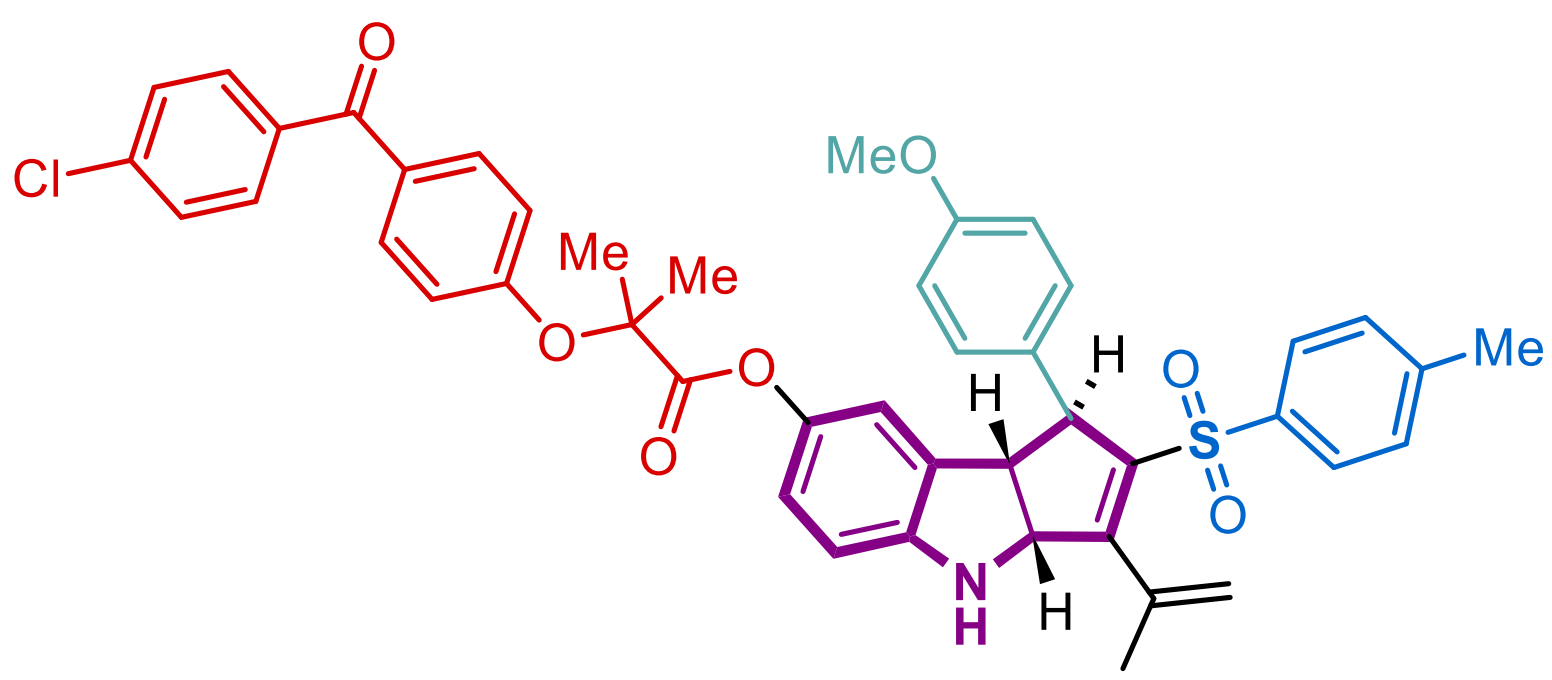

7b

(1S,3aS,8bR)-1-(4-methoxyphenyl)-3-(prop-1-en-2-yl)-2-tosyl-1,3a,4,8b-tetrahydrocyclopenta[b]indol-7-yl 2-(4-(4-chlorobenzoyl)phenoxy)-2-methylpropanoate (**7b**): Prepared according to GP-3 on 0.3 mmol (110 mg)

Orange solid (187 mg, 78% yield)

Rf = 0.30 (40% EA/hexane)

Melting point = 103-105°C

NMR Spectroscopy:

^{1}H NMR (400 MHz, CHLOROFORM-D) δ 7.84 – 7.79 (m, 2H), 7.77 – 7.68 (m, 2H), 7.48 – 7.41 (m, 2H), 7.21 – 7.13 (m, 2H), 7.04 – 6.93 (m, 6H), 6.86 – 6.81 (m, 1H), 6.80 – 6.72 (m, 2H), 6.67 (dd, *J* = 8.7, 2.2 Hz, 1H), 6.57 (d, *J* = 8.4 Hz, 1H), 5.28 – 5.22 (m, 1H), 5.01 (dd, *J* = 7.7, 1.7 Hz, 1H), 4.79 (s, 1H), 4.36 (s, 1H), 3.80 (s, 3H), 3.75 (d, *J* = 7.8 Hz, 1H), 2.32 (s, 3H), 1.85 (d, *J* = 12.2 Hz, 9H) ppm.

^{13}C NMR (101 MHz, CHLOROFORM-D) δ 194.4, 172.9, 159.7, 158.8, 154.2, 146.5, 143.9, 143.7, 141.9, 138.5, 138.1, 137.2, 136.4, 133.5, 132.3, 131.9, 131.3, 130.7, 129.1, 128.7, 128.6, 127.9, 121.1, 118.2, 117.7, 117.5, 114.3, 110.4, 79.6, 71.0, 59.2, 55.5, 53.9, 25.7, 25.5, 22.5, 21.6 ppm.

HRMS (ESI) m/z: [M+H]+ Calcd for $C_{45}H_{41}ClNO_7S^+$ 774.2287; Found 774.2299.

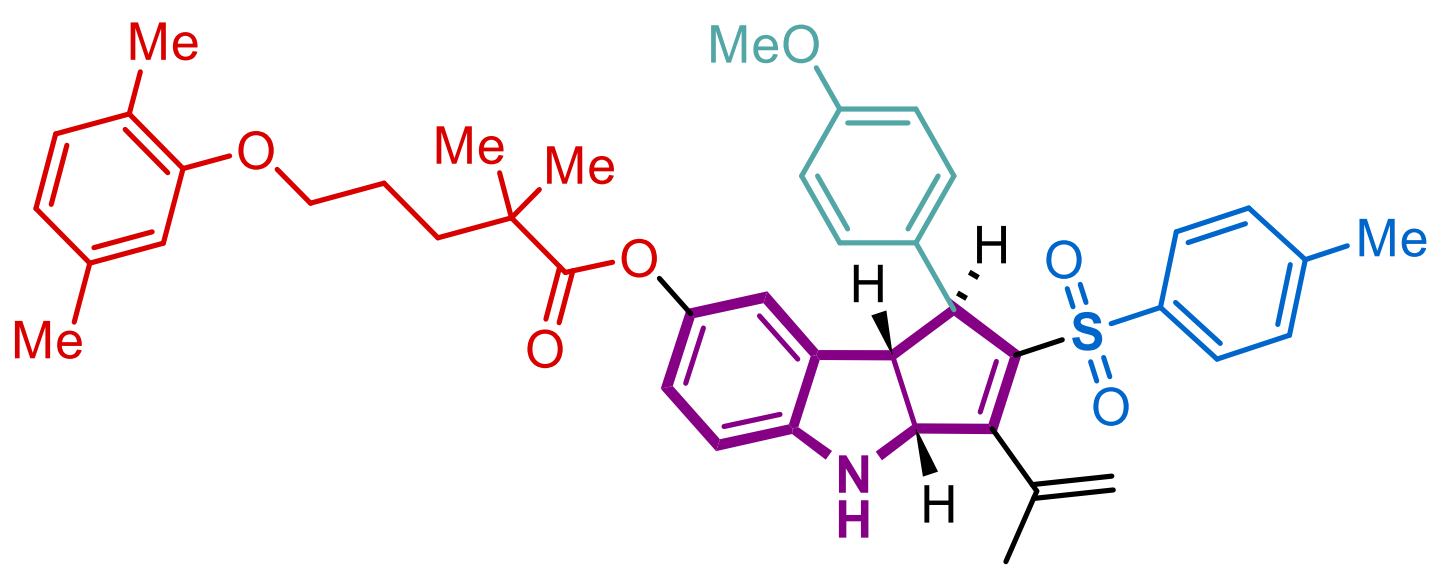

7c

(1S,3aS,8bR)-1-(4-methoxyphenyl)-3-(prop-1-en-2-yl)-2-tosyl-1,3a,4,8b-tetrahydrocyclopenta[b]indol-7-yl 5-(2,5-dimethylphenoxy)-2,2-dimethylpentanoate (**7c**): Prepared according to GP-3 on 0.3 mmol (110 mg)

Brown thick liquid (179 mg, 82% yield)

Rf = 0.30 (40% EA/hexane)

NMR Spectroscopy:

^{1}H NMR (400 MHz, CHLOROFORM-D) δ 7.21 (d, *J* = 8.2 Hz, 2H), 7.05 – 6.93 (m, 5H), 6.91 (d, *J* = 1.8 Hz, 1H), 6.76 (ddd, *J* = 10.7, 7.6, 2.1 Hz, 3H), 6.67 (d, *J* = 10.0 Hz, 2H), 6.59 (d, *J* = 8.3 Hz, 1H), 5.25 (d, *J* = 1.4 Hz, 1H), 5.00 (dd, *J* = 7.7, 1.7 Hz, 1H), 4.79 (s, 1H), 4.41 (s, 1H), 4.19 – 4.05 (m, 1H), 4.01 (s, 2H), 3.81 (s, 3H), 3.77 (d, *J* = 7.8 Hz, 1H), 2.32 (d, *J* = 5.3 Hz, 6H), 2.20 (s, 3H), 1.93 – 1.83 (m, 7H), 1.38 (d, *J* = 3.3 Hz, 6H) ppm.

^{13}C NMR (101 MHz, CHLOROFORM-D) δ 176.9, 158.8, 157.0, 154.4, 146.0, 144.4, 143.8, 141.9, 138.2, 137.3, 136.6, 133.7, 131.7, 130.4, 129.1, 128.7, 127.9, 123.7, 121.5, 120.8, 118.1,

114.2, 112.1, 110.5, 71.0, 67.9, 59.1, 55.5, 54.1, 42.5, 37.3, 25.4, 25.2, 22.5, 21.6, 21.5, 16.0 ppm.

HRMS (ESI) m/z: [M+H]+ Calcd for $C_{43}H_{48}NO_6S^+$ 706.3197; Found 706.3208.

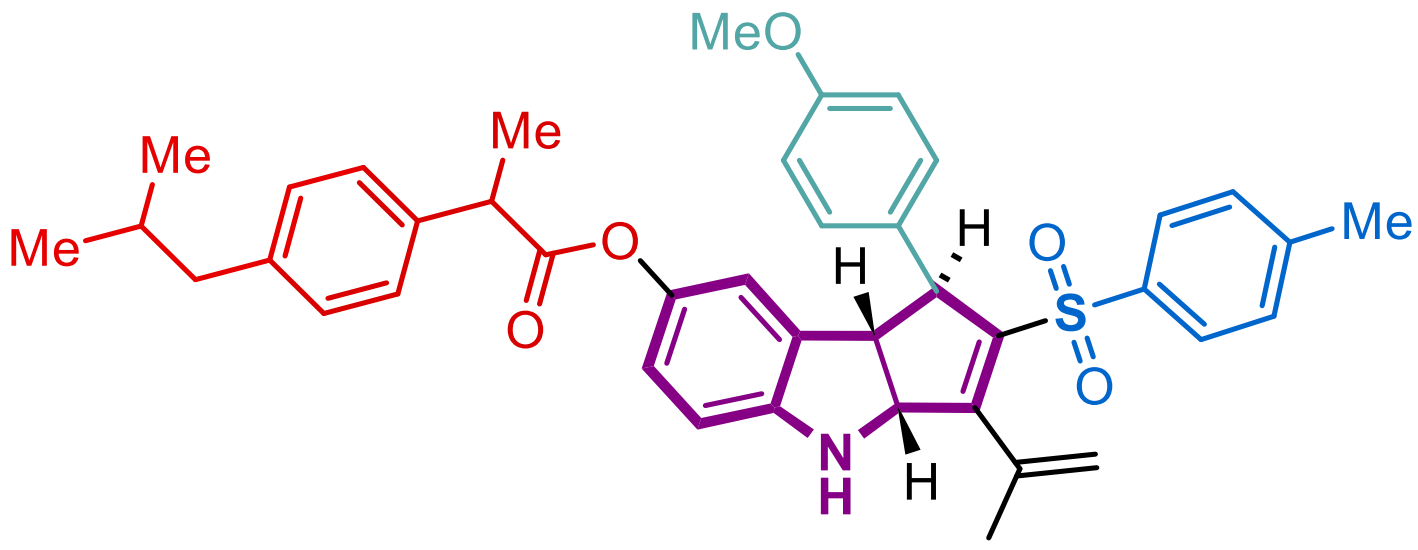

7d

(1S,3aS,8bR)-1-(4-methoxyphenyl)-3-(prop-1-en-2-yl)-2-tosyl-1,3a,4,8b-tetrahydrocyclopenta[b]indol-7-yl 2-(4-isobutylphenyl)propanoate (**7d**): Prepared according to GP-3 on 0.3 mmol (110 mg)

Brown thick liquid (82 mg, 40% yield)

Rf = 0.30 (40% EA/hexane)

NMR Spectroscopy:

^{1}H NMR (400 MHz, CHLOROFORM-D) δ 7.32 (dd, *J* = 8.1, 3.3 Hz, 2H), 7.19 (ddd, *J* = 15.0, 8.2, 2.8 Hz, 4H), 7.05 – 6.97 (m, 4H), 6.90 – 6.81 (m, 1H), 6.79 (d, *J* = 8.4 Hz, 2H), 6.70 (ddd, *J* = 18.8, 8.5, 2.3 Hz, 1H), 6.56 (dd, *J* = 8.4, 3.3 Hz, 1H), 5.24 (s, 1H), 4.98 (dt, *J* = 7.6, 1.6 Hz, 1H), 4.75 (s, 1H), 4.35 (d, *J* = 6.9 Hz, 1H), 3.93 (q, *J* = 7.1 Hz, 1H), 3.81 (s, 3H), 3.74 (d, *J* = 7.3 Hz, 1H), 2.49 (dd, *J* = 7.2, 2.8 Hz,2H), 2.31 (s, 3H), 1.88 (s, 3H), 1.62 (d, *J* = 7.2 Hz, 3H), 1.36 – 1.22 (m, 2H), 0.90 (ddd, *J* = 17.3, 8.5, 2.7 Hz, 8H) ppm.

^{13}C NMR (101 MHz, CHLOROFORM-D) δ 173.8, 158.8, 154.3, 145.9, 144.3, 143.9, 141.9, 140.8, 138.1, 137.5, 137.2, 133.6, 131.7, 129.6, 129.0, 128.6, 127.8, 127.3, 121.4, 117.9, 114.2, 110.5, 70.9, 59.1, 59.1, 55.4, 54.0, 45.3, 45.2, 34.2, 30.3, 22.5, 22.5, 21.6, 18.8, 14.2 ppm.

HRMS (ESI) m/z: [M+H]+ Calcd for $C_{41}H_{44}NO_5S^+$ 662.2935; Found 662.2933.

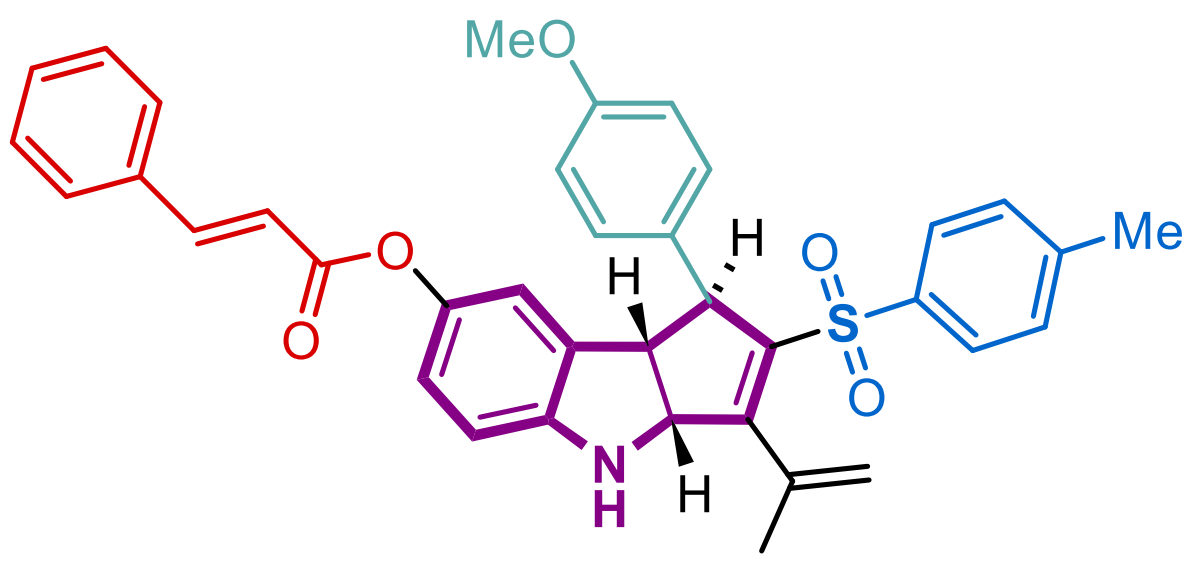

7e

(1S,3aS,8bR)-1-(4-methoxyphenyl)-3-(prop-1-en-2-yl)-2-tosyl-1,3a,4,8b-tetrahydrocyclopenta[b]indol-7-yl cinnamate (**7e**): Prepared according to GP-3 on 0.3 mmol (110 mg).

Yellow thick liquid (170 mg, 91% yield)

Rf = 0.30 (40% EA/hexane)

NMR Spectroscopy:

^{1}H NMR (400 MHz, CHLOROFORM-D) δ 7.87 (d, *J* = 16.0 Hz, 1H), 7.67 – 7.56 (m, 2H), 7.49 – 7.35 (m, 3H), 7.22 (d, *J* = 8.3 Hz, 2H), 7.07 – 6.95 (m, 5H), 6.88 (dd, *J* = 8.4, 2.3 Hz, 1H), 6.81 – 6.76 (m, 2H), 6.69 – 6.55 (m, 2H), 5.30 – 5.23 (m, 1H), 5.02 (dd, *J* = 7.7, 1.4 Hz, 1H), 4.81 (s, 1H), 4.41 (s, 1H), 4.19 – 4.07 (m, 1H), 3.80 (s, 3H), 3.78 (s, 1H), 2.33 (s, 3H), 1.90 (s, 3H) ppm.

^{13}C NMR (101 MHz, CHLOROFORM-D) δ 166.1, 158.7, 154.4, 146.4, 146.2, 144.1, 143.8, 141.8, 138.1, 137.3, 134.3, 133.6, 131.8, 130.7, 129.1, 129.0, 128.6, 128.3, 127.8, 121.6, 118.1, 117.9, 117.5, 114.2, 110.5, 70.9, 59.3, 55.4, 54.1, 34.0, 25.7, 25.0, 22.6, 21.6, 14.3 ppm.

HRMS (ESI) m/z: [M+H]+ Calcd for $C_{37}H_{34}NO_5S^+$ 604.2152; Found 604.2168.

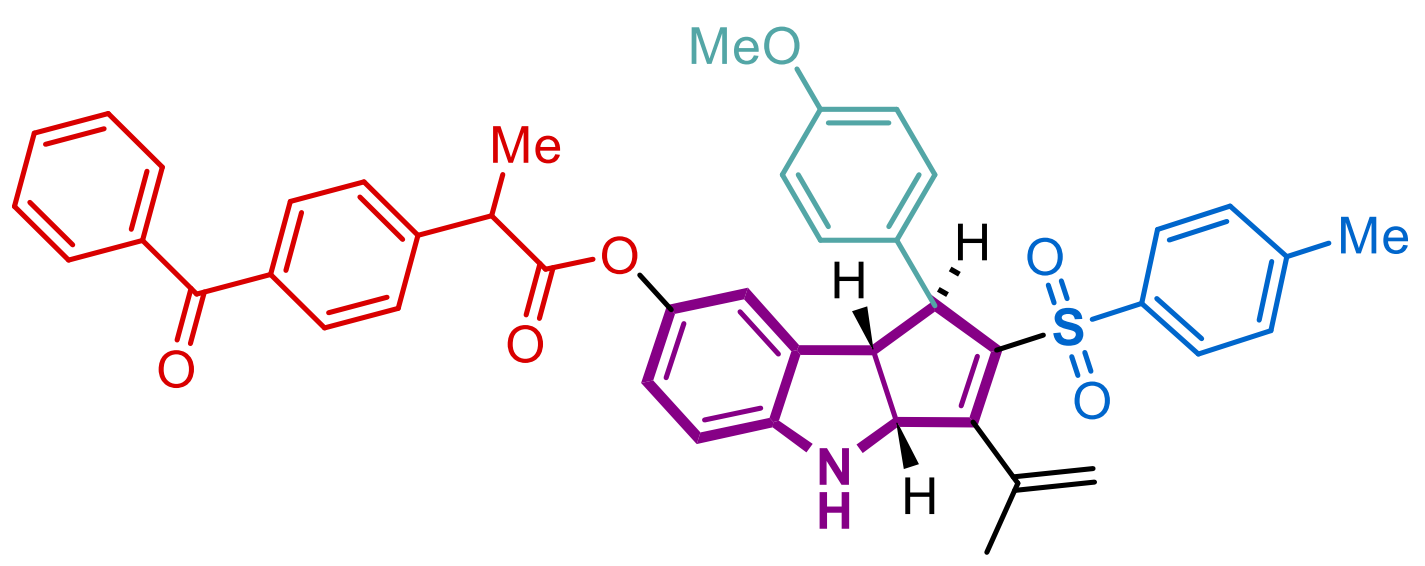

7f

ethyl-4-((1R,3aS,8bR)-1-(4-methoxyphenyl)-3-(prop-1-en-2-yl)-2-tosyl-3a,4-dihydrocyclopenta[b]indol-8b(1H)-yl)butanoate (**7f**): Prepared according to GP-3 on 0.3 mmol (110 mg).

Brown thick liquid (90 mg, 42% yield)

Rf = 0.30 (40% EA/hexane)

NMR Spectroscopy:

^{1}H NMR (400 MHz, CHLOROFORM-D) δ 7.85 (ddd, *J* = 11.1, 5.6, 3.7 Hz, 3H), 7.77 – 7.72 (m, 1H), 7.67 (dd, *J* = 7.8, 1.3 Hz, 1H), 7.60 (td, *J* = 7.3, 1.3 Hz, 1H), 7.56 – 7.44 (m, 3H), 7.20 (dd, *J* = 8.3, 2.4 Hz, 2H), 7.00 (dt, *J* = 12.2, 4.0 Hz, 4H), 6.93 – 6.84 (m, 1H), 6.78 (dd, *J* = 8.7, 2.6 Hz, 2H), 6.71 (ddd, *J* = 15.7, 8.3, 2.3 Hz, 1H), 6.56 (dd, *J* = 8.4, 2.5 Hz, 1H), 5.24 (s, 1H), 4.99 (dt, *J* = 7.7, 1.7 Hz, 1H), 4.77 (s, 1H), 4.38 (d, *J* = 4.2 Hz, 1H), 4.04 (q, *J* = 7.2 Hz, 1H), 3.81 (d, *J* = 1.8 Hz, 3H), 3.75 (d, *J* = 7.2 Hz, 1H), 2.31 (s, 3H), 1.87 (s, 3H), 1.67 (d, *J* = 7.2 Hz, 3H) ppm.

^{13}C NMR (101 MHz, CHLOROFORM-D) δ 196.6, 173.2, 158.8, 154.3, 146.2, 144.1, 143.9, 141.9, 138.1, 137.5, 137.2, 133.6, 132.7, 131.7, 130.2, 129.5, 129.4, 129.4, 129.0, 128.9, 128.7, 128.5, 127.9, 121.4, 117.9, 114.2, 110.4, 70.9, 59.1, 55.4, 54.0, 45.6, 22.5, 21.6, 18.8 ppm.

HRMS (ESI) m/z: [M+H]+ Calcd for $C_{44}H_{40}NO_6S^+$ 710.2576; Found 710.2546.

2.5 General procedure for the gram-scale reaction

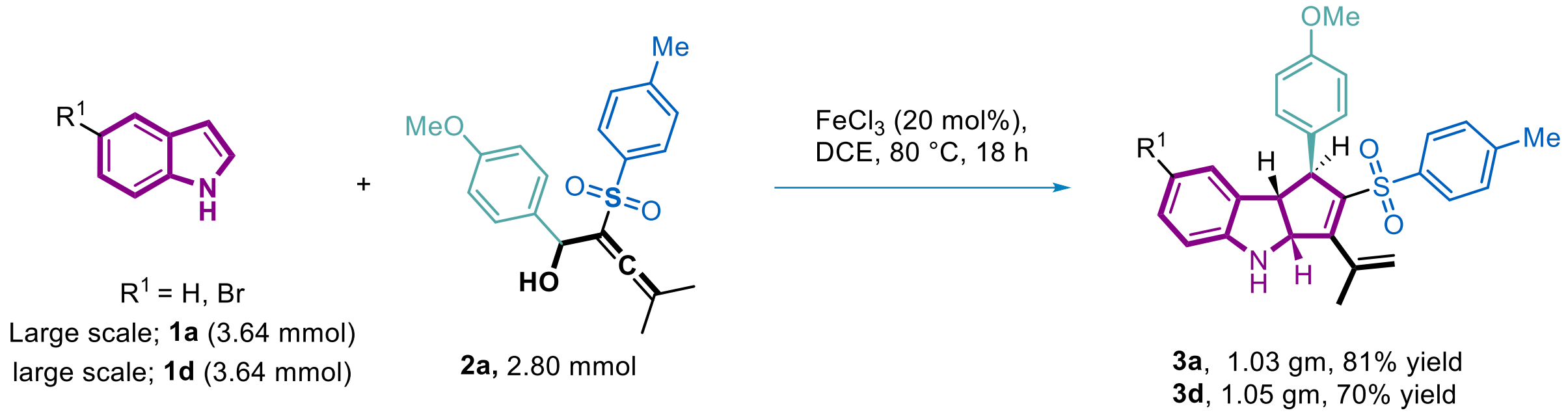

A clear glass sealed tube, containing a magnetic stir bar, was charged with the indole substrates **1a/1d** (0.39 mmol, 1.3 equiv), the catalyst $FeCl_3$ (20 mol %), and substrates **2a** (0.3 mmol, 1.0 equiv) in 1,2-dichloroethane. Subsequently, 3 ml of dry DCE was added to the reaction mixture, following which the vial was sealed. The reaction was stirred for 18 h at 80 °C with the help of an aluminium heating block. The reaction mixture was filtered through celite, extracted with dichloromethane, and dried over anhydrous sodium sulfate. Removal of the solvent under vacuum afforded the crude product, which was purified by column chromatography to afford **3a** and **3d.**

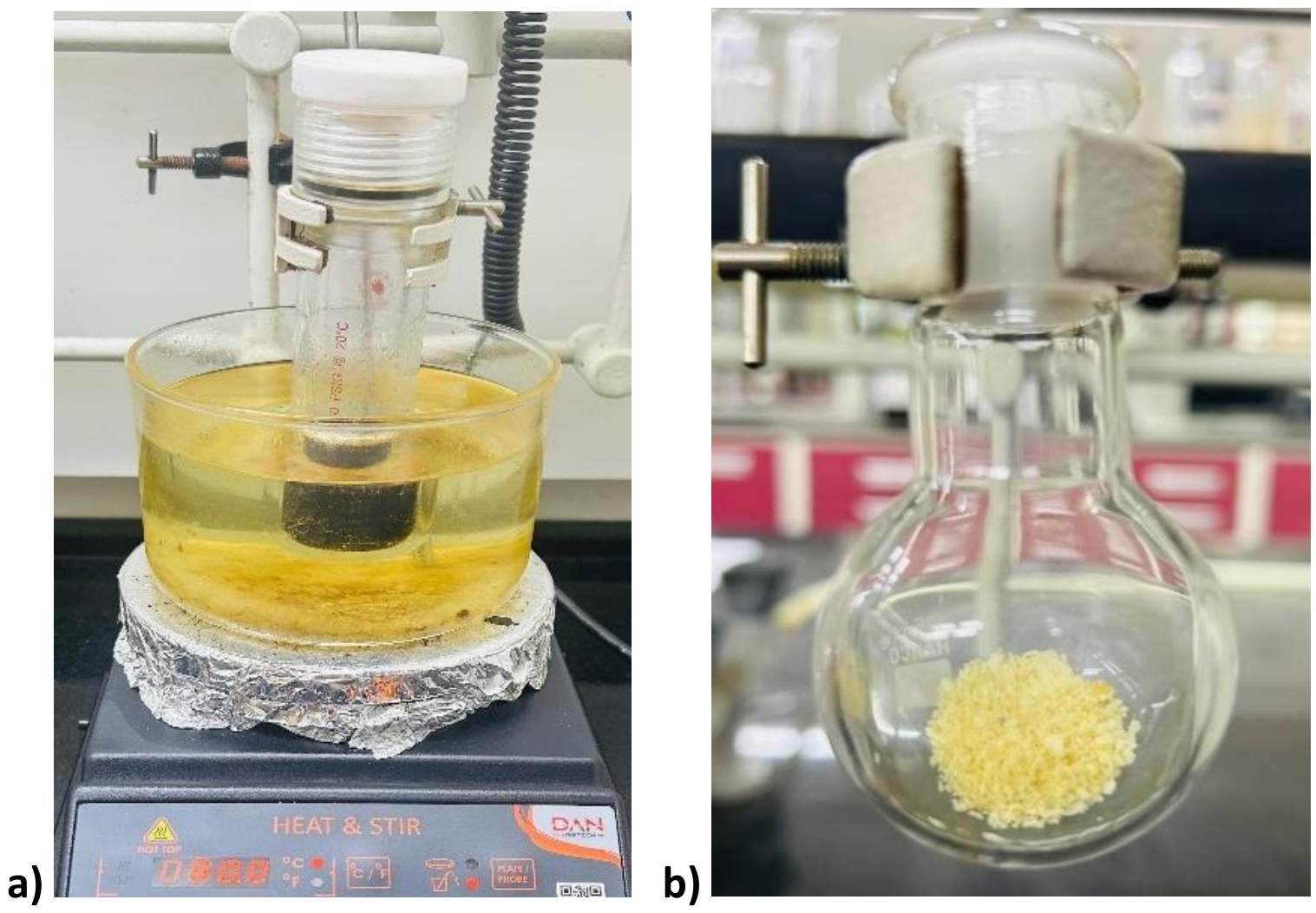

Figure S2 : *a) Gram scale reaction setup. b) Isolated dearomative formal[3+2] cyclopenta(b)indoles from scale-up reaction.*

2.6 Double dearomative formal [3+2] cycloaddition reaction

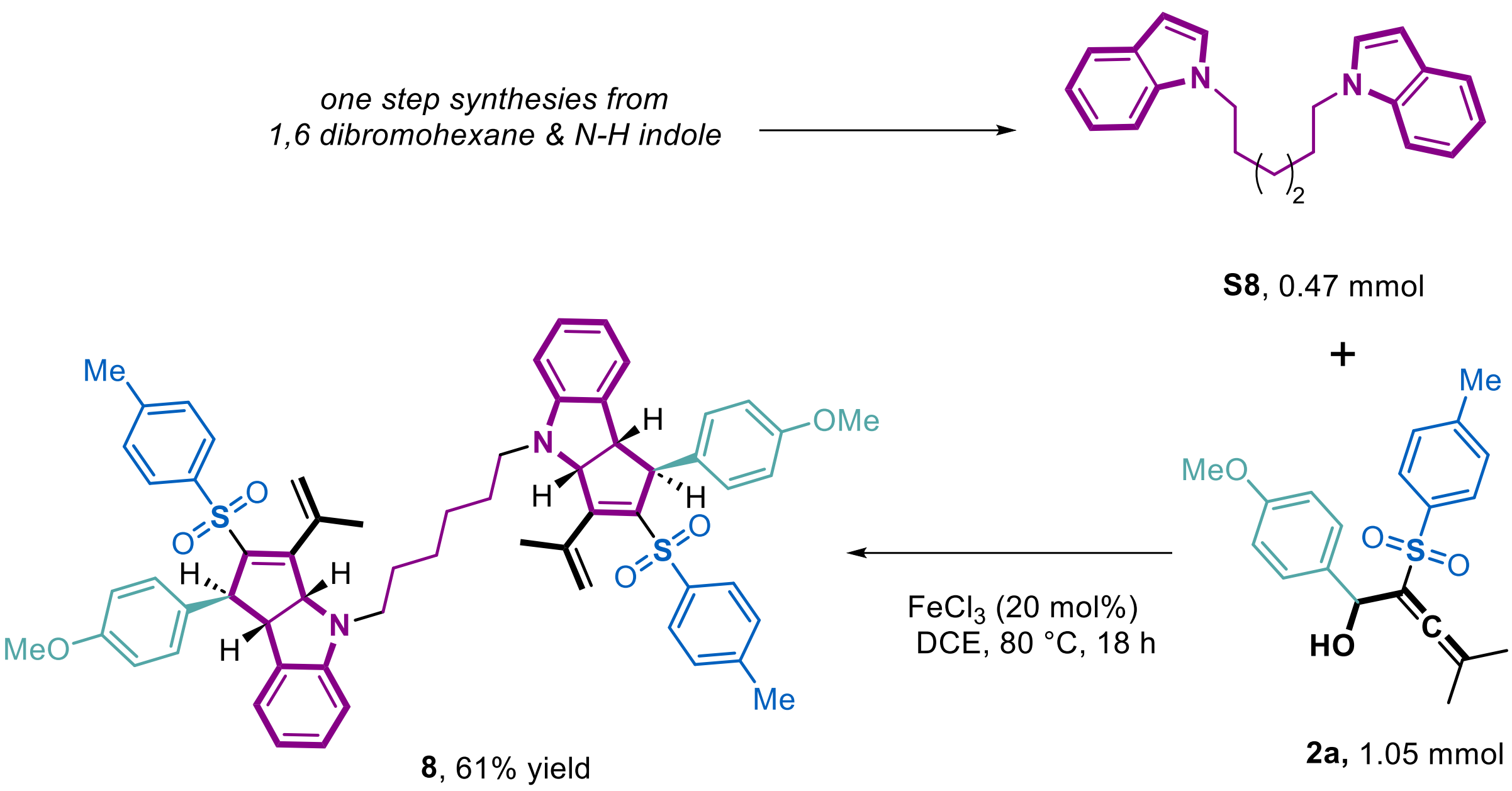

The bis-indole substrate is prepared according to the literature protocol.[11]

Following the GP3, a clear glass screw vial with a Teflon cap, containing a magnetic stir bar, was charged with the indole substrate **S8** (0.47 mmol, 1.0 equiv), the catalyst $FeCl_3$ (20 mol %), and allenol substrate **2a** (1.05 mmol, 2.2 equiv) in 1,2-dichloroethane. The reaction was stirred for 18 h at 80 °C with the help of an aluminium heating block. The reaction mixture was filtered through celite, extracted with dichloromethane, and dried over anhydrous sodium sulfate. Removal of the solvent under vacuum afforded the crude product, which was purified by column chromatography to afford **8** as off yellow solid with 61% isolated yield. Further, compound **8** was confirmed with ^{1}H-NMR, ^{13}C-NMR, and HRMS analysis.

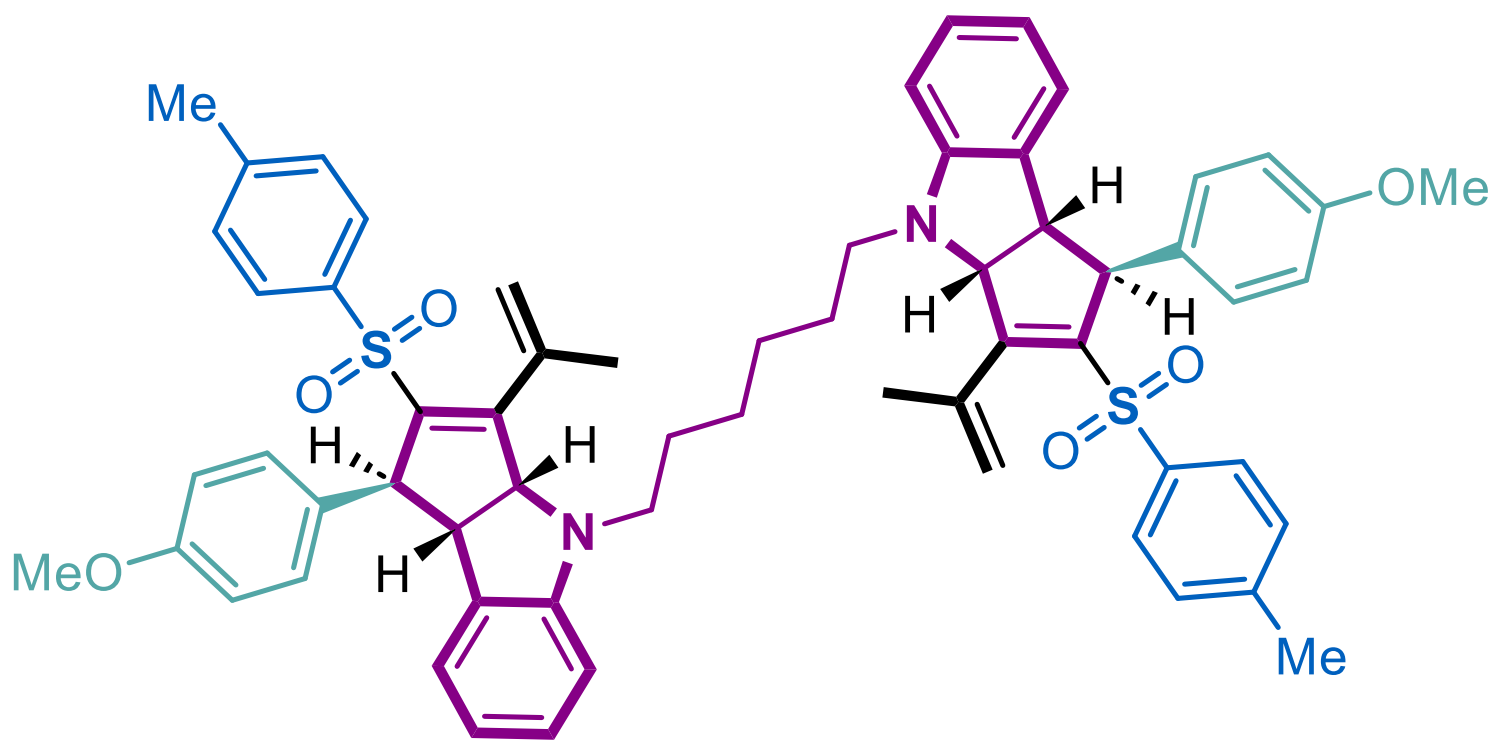

8

1,6-bis((1S,3aS,8bR)-1-(4-methoxyphenyl)-3-(prop-1-en-2-yl)-2-tosyl-3a,8b-dihydrocyclopenta[b]indol-4(1H)-yl)hexane (**8**):
Orange thick liquid (248 mg, 99% yield)

---

[11] Y. Hong et al. / Synthesis of double DeA branched organic dyes employing indole and phenoxazine as donors for efficient DSSCs. Tetrahedron 70 (2014) 6296e6302

Rf = 0.28 (30% EA/hexane)

NMR Spectroscopy:

^{1}H NMR (400 MHz, CHLOROFORM-D) δ 7.16 (d, J = 8.3 Hz, 4H), 7.07 (dd, J = 16.4, 7.8 Hz, 4H), 6.99 – 6.93 (m, 8H), 6.77 – 6.70 (m, 4H), 6.67 (t, J = 7.3 Hz, 2H), 6.39 (d, J = 7.8 Hz, 2H), 5.21 – 5.13 (m, 2H), 4.91 (dt, J = 8.6, 1.6 Hz, 2H), 4.85 (s, 2H), 4.45 (d, J = 1.5 Hz, 2H), 3.84 – 3.81 (m, 2H), 3.80 (d, J = 1.9 Hz, 6H), 3.34 (dt, J = 13.0, 6.4 Hz, 2H), 3.12 (dt, J = 14.6, 7.2 Hz, 2H), 2.32 (s, 6H), 1.96 (s, 6H), 1.46 (s, 4H), 1.23 (d, J = 11.3 Hz, 4H) ppm.

^{13}C NMR (101 MHz, CHLOROFORM-D) δ 158.7, 155.3, 149.2, 143.6, 141.5, 139.7, 138.4, 134.4, 131.4, 128.9, 128.8, 128.6, 128.0, 124.4, 117.9, 115.9, 114.1, 107.9, 58.9, 55.5, 55.4, 54.1, 48.4, 27.2, 27.1, 23.4, 21.6 ppm.

HRMS (ESI) m/z: [M+H]+ Calcd for $C_{62}H_{65}N_2O_6S_2^+$ 997.4300; Found 997.4284.

2.7 Product diversification

1) **Suzuki-cross coupling reaction of 3d:**

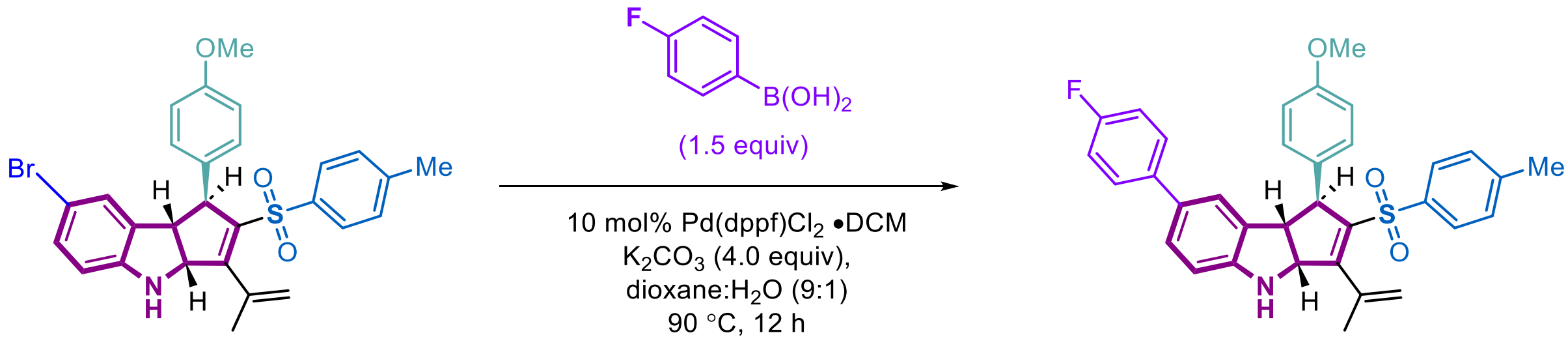

3d

9, 65% yield

To a solution of **3d** (50 mg, 0.09 mmol), (4-fluorophenyl) boronic acid (38 mg, 0.3 mmol), K_2CO_3 (50 mg, 0.40 mmol) in dioxane/H_2O (5 mL) under nitrogen, a catalytic amount of Pd(dppf)Cl_2.DCM (7.4 mg) was added. The solution was heated at 90 °C for 12 h. The solution was extracted with water and ethyl acetate. The organic phase was dried by magnesium sulfate, and the solvent was evaporated. The crude product was purified by column chromatography (silica gel, Hexane/EtOAc) to give a yellow powder of **9** in 65% yield (34 mg).

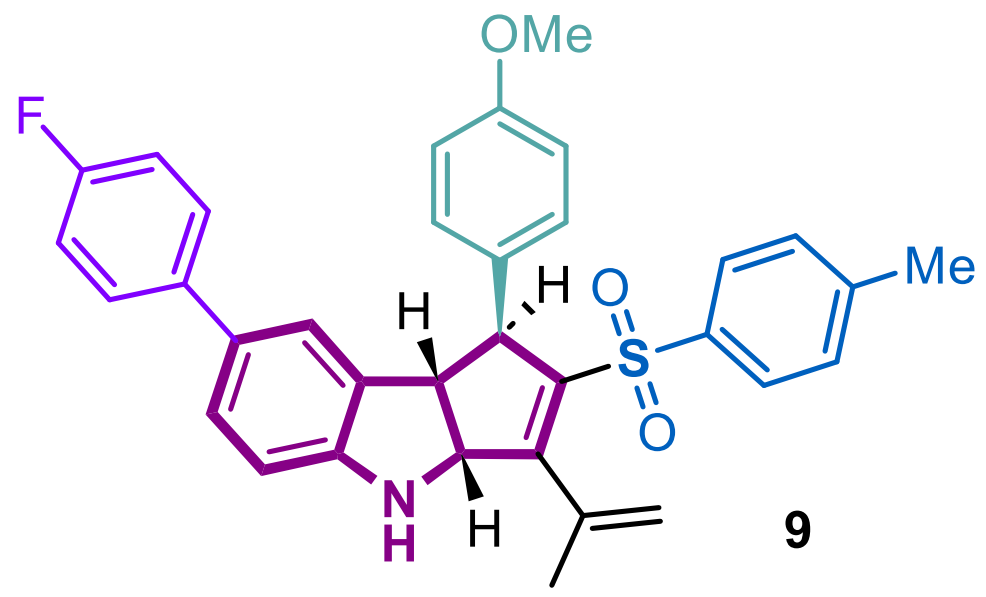

9

(1S)-7-(4-fluorophenyl)-1-(4-methoxyphenyl)-3-(prop-1-en-2-yl)-2-tosyl-1,3a,4,8b-tetrahydrocyclopenta[b]indole (**9**):

Brown solid (34 mg, 65% yield)

Rf = 0.42 (25% EA/hexane)

Melting point = 210-212°C

NMR Spectroscopy:

^{1}H NMR (400 MHz, CHLOROFORM-D) δ 7.45 (dd, *J* = 8.7, 5.4 Hz, 2H), 7.31 (s, 1H), 7.28 – 7.22 (m, 1H), 7.20 (d, *J* = 8.2 Hz, 2H), 7.10 (t, *J* = 8.7 Hz, 2H), 7.05 (d, *J* = 8.6 Hz, 2H), 6.98 (d, *J* = 8.1 Hz, 2H), 6.80 (d, *J* = 8.6 Hz, 2H), 6.69 (d, *J* = 8.0 Hz, 1H), 5.37 – 5.25 (m, 1H), 5.03 (dd, *J* = 7.6, 1.6 Hz, 1H), 4.87 (s, 1H), 4.44 (s, 1H), 4.21 (s, 1H), 3.81 (s, 3H), 3.79 (s, 1H), 2.28 (s, 3H), 1.90 (s, 3H) ppm.

^{13}C NMR (101 MHz, CHLOROFORM-D) δ 163.2-160.8 (d, *J* = 245.1 Hz), 158.8, 154.3, 147.9, 143.9, 141.8, 137.9, 137.7, 137.7, 137.3, 133.8, 132.3, 131.4, 129.0, 128.7, 128.2-128.2 (d, *J* = 7.8 Hz), 127.9, 127.7, 123.3, 118.0, 115.6-115.4 (d, *J* = 21.2 Hz), 114.2, 110.4, 70.7, 59.2, 55.4, 55.4, 53.9, 22.6, 21.6 ppm.

^{19}F NMR (376 MHz, CHLOROFORM-D) δ -117.05.

HRMS (ESI) m/z: [M+H]+ Calcd for $C_{34}H_{31}FNO_3S^+$ 552.2009; Found 552.2002.

2) Hydrogenation of the prenyl olefin of 3a:

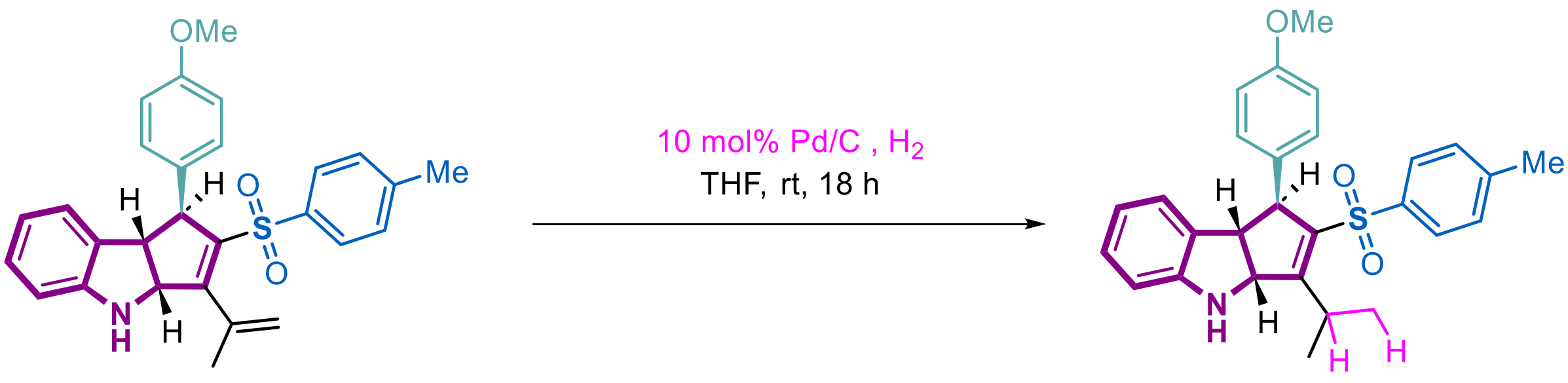

3a **10**, 73% yield

In an Argon-flushed round-bottom flask, 50 mg (1 equiv.) of compound **3a** and 1.15 mg of Pd/C (10% palladium on carbon) were dissolved in 5 ml of anhydrous THF. The reaction was stirred for 18 h under the H_2 balloon pressure at room temperature. After complete consumption of **3a**, the mixture was subsequently filtered through Celite. The solvent was then removed under reduced pressure, resulting in the desired hydrogenated product purified by column chromatography (silica gel, Hexane/EtOAc) to give a yellow powder of **9** in 73% yield (36 mg).

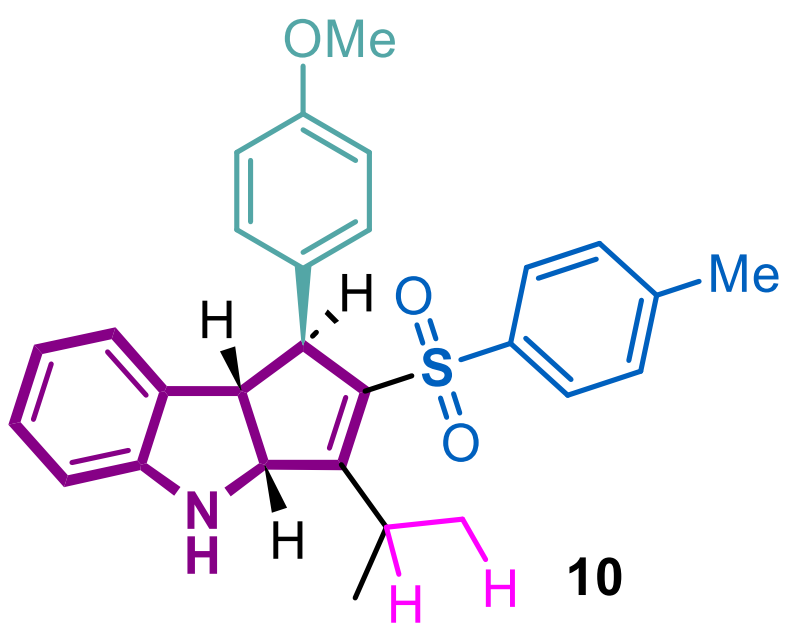

10

(1S)-3-isopropyl-1-(4-methoxyphenyl)-2-tosyl-1,3a,4,8b-tetrahydrocyclopenta[b]indole (10)

Brown Thick Liquid (36 mg, 73% yield)

Rf = 0.50 (20% EA/hexane)

NMR Spectroscopy:

^{1}H NMR (400 MHz, CHLOROFORM-D) δ 7.17 – 7.04 (m, 4H), 6.99 (d, *J* = 8.3 Hz, 2H), 6.93 – 6.87 (m, 2H), 6.77 (dt, *J* = 7.3, 4.4 Hz, 1H), 6.73 – 6.68 (m, 2H), 6.64 (d, *J* = 7.8 Hz, 1H), 5.23 (dd, *J* = 8.5, 1.7 Hz, 1H), 4.32 (t, *J* = 1.9 Hz, 1H), 3.83 (d, *J* = 6.4 Hz, 1H), 3.80 (s, 3H), 3.76 (dd, *J* = 8.5, 2.5 Hz, 1H), 2.38 – 2.29 (m, 3H), 1.27 (dd, *J* = 13.5, 7.0 Hz, 7H) ppm.

^{13}C NMR (101 MHz, CHLOROFORM-D) δ 159.5, 158.7, 148.9, 143.5, 140.5, 138.6, 134.4, 131.2, 129.1, 128.9, 128.5, 127.5, 124.5, 119.7, 114.1, 110.2, 69.5, 59.4, 55.4, 54.5, 27.9, 22.7, 21.6, 20.8 ppm

HRMS (ESI) m/z: [M+H]+ Calcd for $C_{28}H_{30}NO_3S^+$ 460.1946; Found 460.1956.

3) Cyclopropanation of prenyl olefin of 3a:

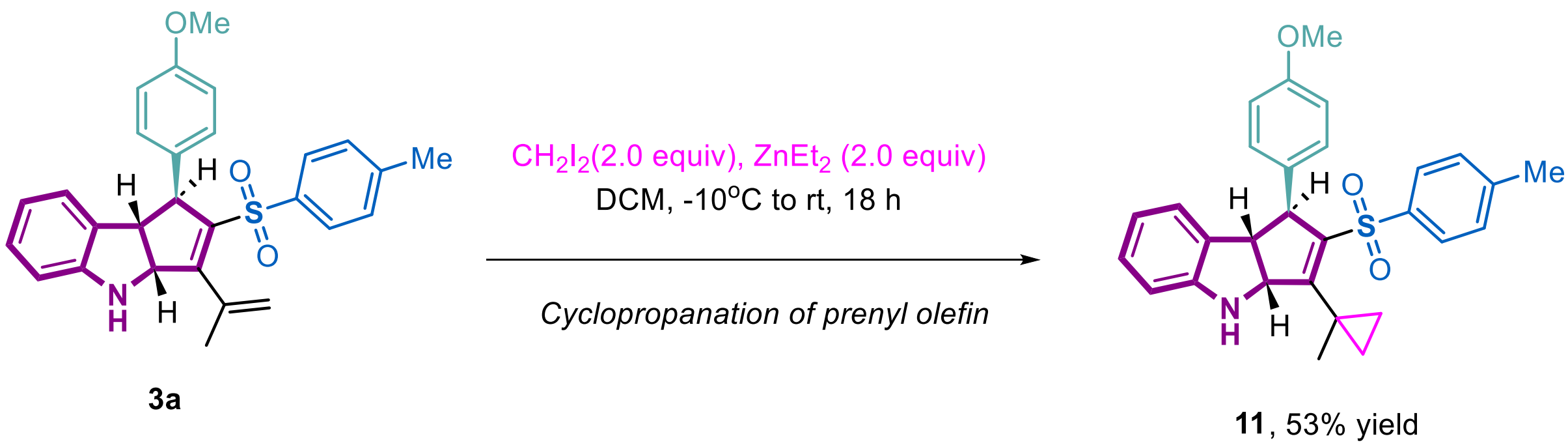

To a solution of $ZnEt_2$ (0.21 mL, 2.0 equiv) in CH_2Cl_2 (3 mL) at −10 °C was added a solution of CH_2I_2 (20 μL, 2.0 equiv) in CH_2Cl_2 (2 mL). This solution was stirred at −10 °C for 20 min, after which **3a** in 1 ml CH_2Cl_2 (50 mg, 1.0 equiv) was added. This solution was stirred for an additional 30 min at −10 °C and then 18 h at room temperature. After complete consumption of **3a**, the reaction mixture was quenched with aq. NH4Cl was extracted with EtOAC. The crude reaction mixture was evaporated and purified by column chromatography (silica gel, Hexane/EtOAc) to give a yellow powder of **11** in 53% yield (27 mg).

The reaction forms the mixture of diastereomers with a ratio of 3:1. Spectral data is given for the diastereomeric mixture.

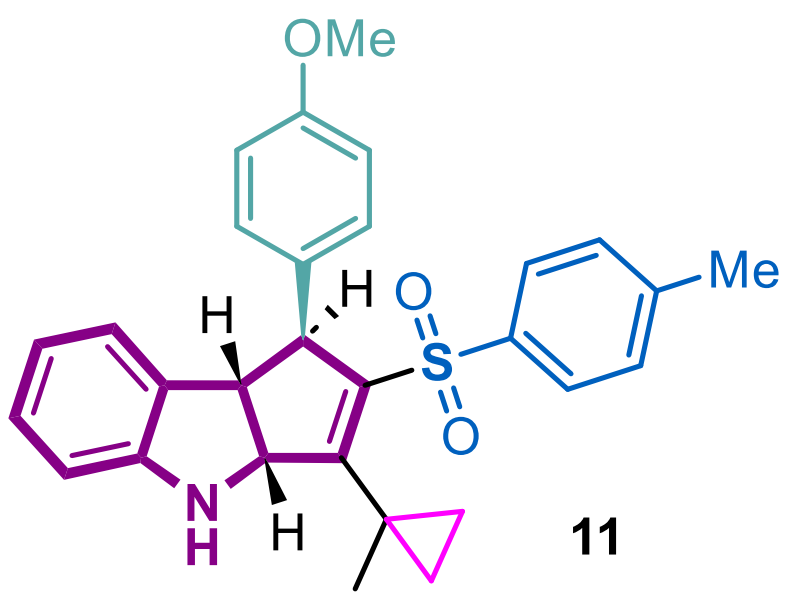

11

(1S)-1-(4-methoxyphenyl)-3-(1-methylcyclopropyl)-2-tosyl-1,3a,4,8b-tetrahydrocyclopenta[b]indole (**11**):

Green solid (27 mg, 53% yield)

Rf = 0.50 (30% EA/hexane)

Melting point = 154-156°C

NMR Spectroscopy:

^{1}H NMR (400 MHz, CHLOROFORM-D) δ 7.23 (td, *J* = 4.8, 2.4 Hz, 3H), 7.11 (dd, *J* = 12.1, 5.5 Hz, 3H), 6.94 – 6.84 (m, 4H), 6.76 (t, *J* = 7.4 Hz, 1H), 6.43 (d, *J* = 7.9 Hz, 1H), 5.33 (s, 1H), 5.11 (d, *J* = 1.5 Hz, 1H), 4.71 (dd, *J* = 6.4, 1.2 Hz, 1H), 4.51 (s, 1H), 3.79 (s, 3H), 3.64 (d, *J* = 6.0 Hz, 1H), 3.22 (ddd, *J* = 14.2, 12.9, 3.1 Hz, 1H), 2.28 (s, 3H), 2.08 – 1.92 (m, 1H), 1.80 – 1.63 (m, 1H), 1.22 (s, 3H) ppm.

^{13}C NMR (101 MHz, CHLOROFORM-D) δ 158.8, 151.3, 148.9, 143.5, 140.0, 138.3, 138.2, 133.6, 132.3, 128.7, 128.6, 127.6, 124.9, 119.1, 116.0, 114.4, 108.1, 75.1, 59.6, 55.45, 53.0, 45.2, 32.9, 31.7, 21.6 ppm.

HRMS (ESI) m/z: [M+H]+ Calcd for $C_{29}H_{30}NO_3S^+$ 472.6184; Found 472.6196.

3) Demethylative esterification of 3a:

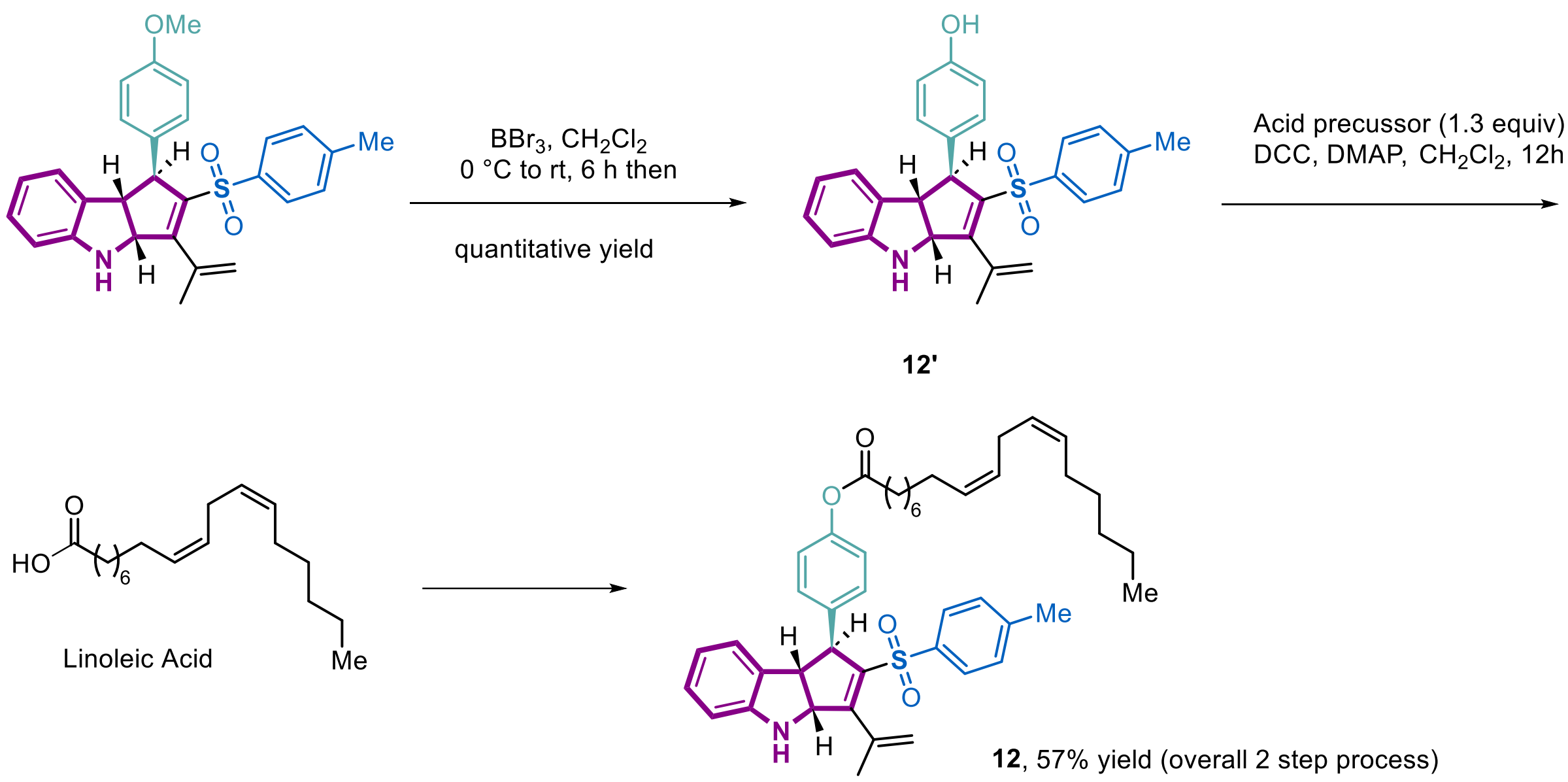

To a flame-dried round-bottom flask equipped with a magnetic stir bar under a nitrogen atmosphere was added the substrate **3a** (80 mg, 0.22 mmol, 1.0 equiv) dissolved in dichloromethane (0.05–0.1 M). The reaction mixture was cooled to 0 °C, and boron tribromide (1.0 M BBr_3, 0.33 ml, 1.5 equiv.) was added dropwise. The reaction was allowed to warm to room temperature and stirred for 3 h. Upon completion (monitored by TLC), the reaction was carefully quenched by the slow addition of H_2O at 0 °C. The mixture was diluted with DCM, and the organic layer was separated. The aqueous phase was extracted with DCM (3 ×10ml). The combined organic layer was dried over anhydrous Na_2SO_4, filtered, and concentrated under reduced pressure to afford the demethylated product, which was used in the next step after further purification by column chromatography in 1:4 (EtOAc: Hexane). The demethylated product **12’** was confirmed through ^{1}H and ^{13}C NMR Spectroscopy.

The 12’ product was further subjected to esterification with Linoleic Acid using **GP1**, yielding the final esterification product **12** with 88% yield.

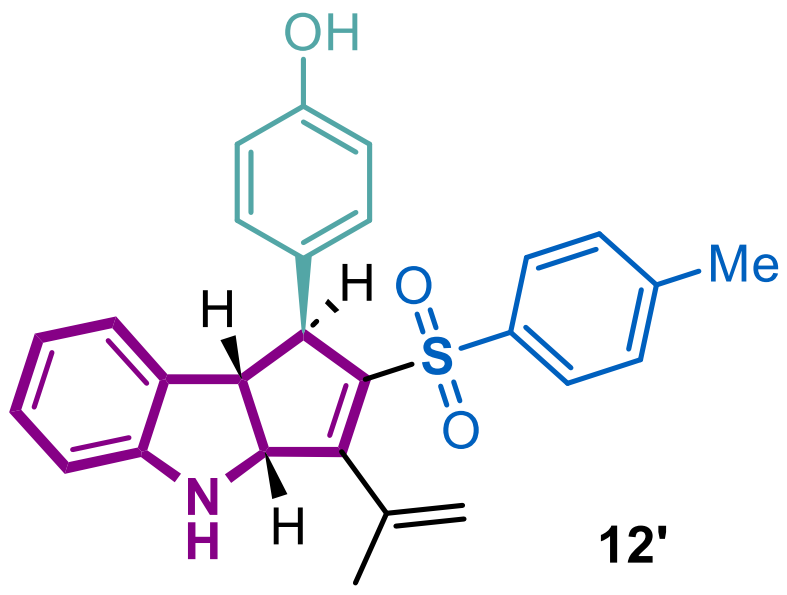

12'

4-((1S)-3-(prop-1-en-2-yl)-2-tosyl-1,3a,4,8b-tetrahydrocyclopenta[b]indol-1-yl)phenol (**12'**):
Brown thick liquid (60 mg, 78% yield)
Rf = 0.40 (30% EA/hexane)
NMR Spectroscopy:
^{1}H NMR (400 MHz, Chloroform-*d*) δ 7.20 (t, *J* = 7.9 Hz, 3H), 7.11 – 7.04 (m, 1H), 7.05 – 6.99 (m, 2H), 6.98 (d, *J* = 8.5 Hz, 2H), 6.80 (td, *J* = 7.4, 1.0 Hz, 1H), 6.70 (d, *J* = 8.5 Hz, 2H), 6.65 (d, *J* = 7.8 Hz, 1H), 5.61 (s, 1H), 5.25 (s, 1H), 4.98 (dd, *J* = 7.6, 1.8 Hz, 3H), 4.84 – 4.66 (m, 3H), 4.42 (t, *J* = 1.4 Hz, 3H), 3.83 – 3.51 (m, 3H), 2.33 (s, 3H), 1.87 (dd, *J* = 1.6, 0.9 Hz, 3H).

^{13}C NMR (101 MHz, Chloroform-*d*) δ 171.6 155.1, 154.7, 148.3, 144.0, 141.8, 137.2, 133.7, 130.6, 129.1, 128.8, 128.7, 127.8, 124.6, 120.0, 118.1, 115.7, 110.5, 70.3, 59.4, 53.9, 52.7, 22.4, 21.6.

HRMS (ESI) m/z: [M+H]+ Calcd for $C_{27}H_{27}NO_3S^+$ 444.1633; Found 444.1637.

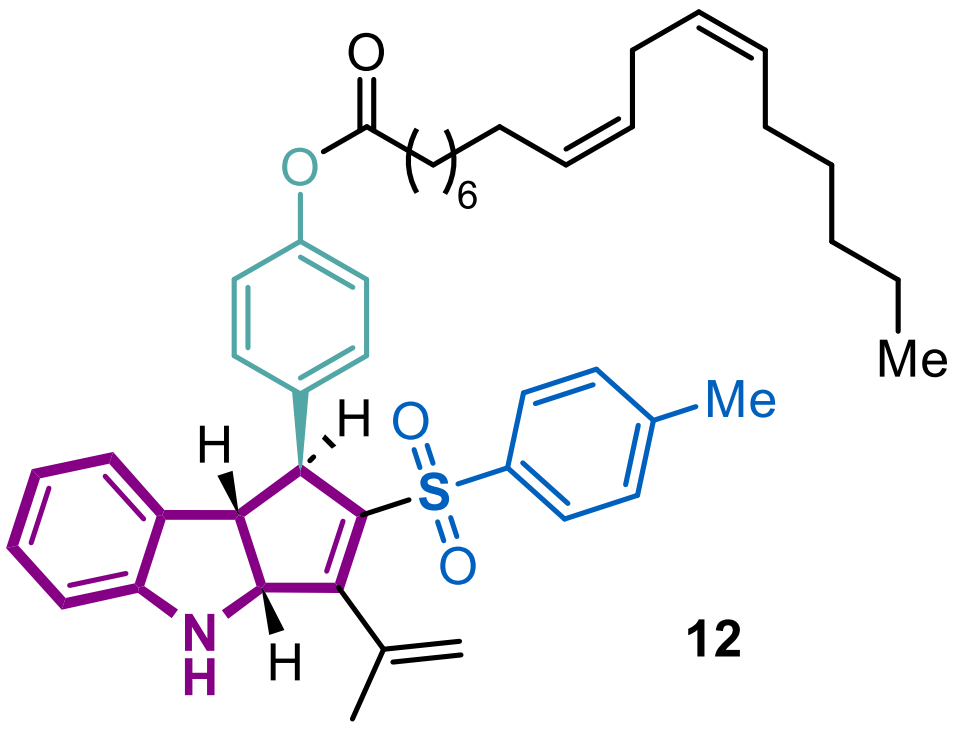

12

4-(3-(prop-1-en-2-yl)-2-(p-tolyl)-1,3a,4,8b-tetrahydrocyclopenta[b]indol-1-yl)phenyl (9Z,12Z)-octadeca-9,12-dienoate (**12**):
Brown thick liquid (70 mg, 88% yield)
Rf = 0.42 (20% EA/hexane)
NMR Spectroscopy:
^{1}H NMR (400 MHz, Chloroform-*d*) δ 7.24 – 7.14 (m, 3H), 7.14 – 7.06 (m, 3H), 7.08 – 7.01 (m, 2H), 6.96 (d, *J* = 8.5 Hz, 2H), 6.80 (td, *J* = 7.4, 1.0 Hz, 1H), 6.65 (d, *J* = 7.7 Hz, 1H), 5.48 – 5.19 (m, 6H), 5.11 – 4.93 (m, 1H), 4.86 (t, *J* = 1.4 Hz, 1H), 4.49 (s, 1H), 3.80 (d, *J* = 7.6 Hz, 1H), 2.78 (q, *J* = 6.5, 6.0 Hz, 3H), 2.57 (t, *J* = 7.5 Hz, 2H), 2.32 (s, 3H), 2.18 – 1.96 (m, 6H), 1.88 (d, *J* = 1.3 Hz, 3H), 1.76 (q, *J* = 7.4 Hz, 2H), 1.45 – 1.19 (m, 14H), 0.99 – 0.76 (m, 3H).

13C NMR (101 MHz, CHLOROFORM-D) δ 172.3, 154.9, 149.8, 148.3, 141.7, 139.3, 137.9, 137.1, 130.3, 130.3, 130.1, 130.1, 129.2, 128.8, 128.7, 128.2, 128.1, 128.0, 127.9, 124.6, 122.0, 120.0, 118.3, 110.5, 77.3, 70.5, 59.6, 53.7, 34.5, 31.6, 29.7, 29.7, 29.3, 29.2, 29.2, 29.23, 29.2, 29.1, 27.3, 27.3, 25.7, 25.7, 25.1, 24.8, 22.7, 22.4, 21.6, 14.2, 14.2 ppm.

HRMS (ESI) m/z: [M+H]+ Calcd for $C_{45}H_{56}NO_4S^+$ 706.3930; Found 706.3937.

2.8 Control experiments

1. Reaction with $CDCl_3$ solvent: A clear glass screw vial with a Teflon cap, containing a magnetic stir bar, was charged with the indole substrates **1a** (0.39 mmol, 1.3 equiv), the catalyst $FeCl_3$ (20 mol %), and substrate **2a** (0.3 mmol, 1.0 equiv) in $CDCl_3$. Subsequently, 3 ml of dry DCE was added to the reaction mixture, following which the vial was sealed. The reaction was stirred for 18 h at 80 °C with the help of an aluminium heating block. The reaction mixture was filtered through Celite, extracted with dichloromethane, and dried over anhydrous sodium sulfate. Removal of the solvent under vacuum afforded the crude product, which was purified by column chromatography to afford **3a-N(D)**.

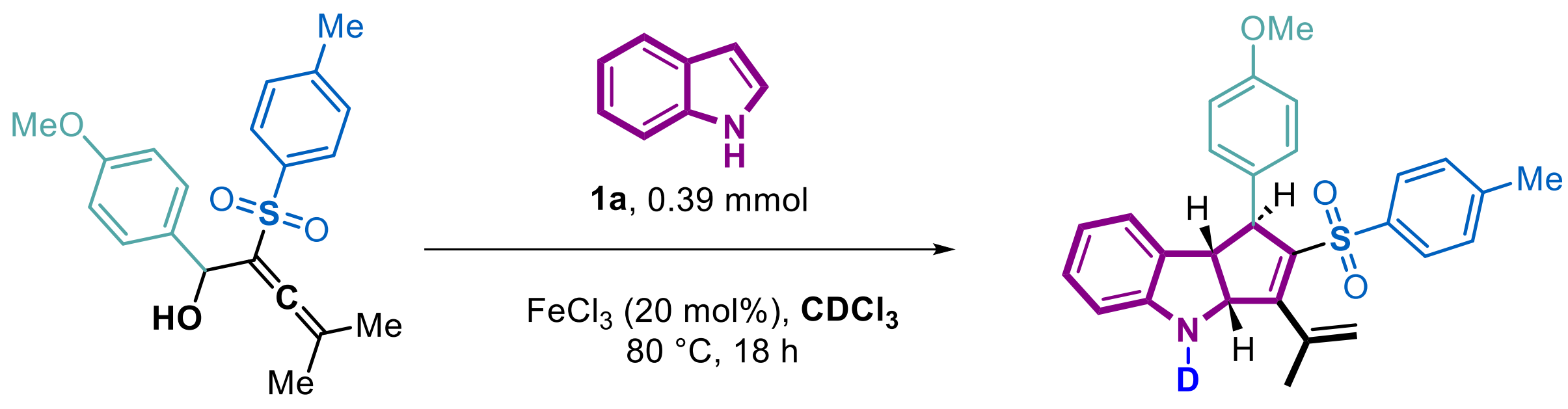

2a, 0.30 mmol **3a-N(D)**, 100%

(1S)-1-(4-methoxyphenyl)-3-(prop-1-en-2-yl)-2-tosyl-1,3a,4,8b-tetrahydrocyclopenta[b]indole-4-d (3a-N(D)):

Brown solid (106 mg, 75% yield)

Rf = 0.38 (30% EA/hexane)

NMR Spectroscopy:

^{1}H NMR (400 MHz, CHLOROFORM-D) δ 7.22 – 7.16 (m, 3H), 7.08 (t, J = 7.6 Hz, 1H), 7.05 – 6.98 (m, 4H), 6.84 – 6.78 (m, 3H), 6.66 (d, J = 7.7 Hz, 1H), 5.28 – 5.24 (m, 1H), 5.00 (dd, J = 7.6, 1.8 Hz, 1H), 4.83 (s, 1H), 4.44 (s, 1H), 3.82 (s, 3H), 3.78 (d, J = 7.2 Hz, 1H), 2.33 (s, 3H), 1.88 (s, 3H).

HRMS (ESI) m/z: [M+H]+ Calcd for $C_{28}H_{27}DNO_3S^+$ 459.1798; Found 459.1814.

2. Reaction with N-D indole: A clear glass screw vial with a Teflon cap, containing a magnetic stir bar, was charged with the indole substrates **1a-N(D)** (0.39 mmol, 1.3 equiv), the catalyst $FeCl_3$ (20 mol %), and substrate **2a** (0.3 mmol, 1.0 equiv) in 1,2-DCE. Subsequently, 3 ml of dry DCE was added to the reaction mixture, following which the vial was sealed. The reaction was stirred for 18 h at 80 °C with the help of an aluminium heating block. The reaction mixture

was filtered through celite, extracted with dichloromethane, and dried over anhydrous sodium sulfate. Removal of the solvent under vacuum afforded the crude product, which was purified by column chromatography to afford **3a-N(D)**.

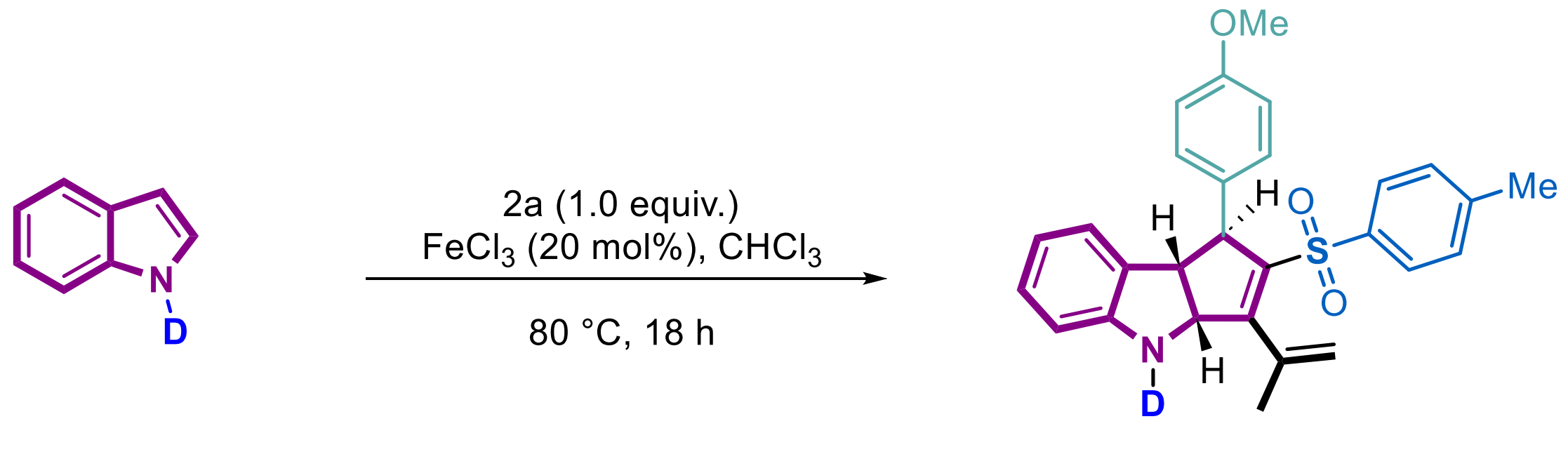

1a-N(D) (1.3 equiv) **3a-N(D)**, 85% yield

3. **Reaction in the absence of indole substrate 1a**: A clear glass screw vial with a Teflon cap, containing a magnetic stir bar, was charged with the substrate **2a** (0.3 mmol, 1.0 equiv) and the catalyst $FeCl_3$ (20 mol %) in $CHCl_3$. Subsequently, 3 ml of dry DCE was added to the reaction mixture, following which the vial was sealed. The reaction was stirred for 18 h at 80 °C with the help of an aluminium heating block. The reaction mixture was filtered through celite, extracted with dichloromethane, and dried over anhydrous sodium sulfate. Removal of the solvent under vacuum afforded the crude product, which was purified by column chromatography to afford two products.

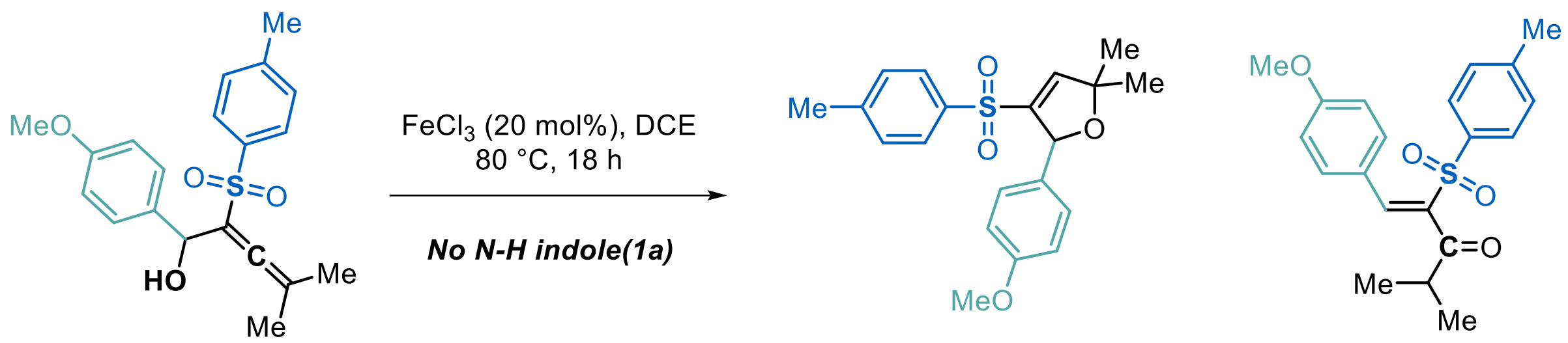

2a 12, 65% yield *through 5 endo cyclization* 13, 15% yield

NMR Spectroscopy:

5-(4-methoxyphenyl)-2,2-dimethyl-4-tosyl-2,5-dihydrofuran:

^{1}H NMR (400 MHz, CHLOROFORM-D) δ 7.92 (s, 1H), 7.78 (d, *J* = 8.4 Hz, 2H), 7.35 – 7.30 (m, 2H), 7.25 – 7.21 (m, 2H), 6.91 – 6.85 (m, 2H), 3.83 (s, 3H), 2.74 (hept, *J* = 6.9 Hz, 1H), 2.43 (s, 3H), 1.61 (dd, *J* = 4.1, 3.1 Hz, 2H), 1.08 (s, 3H), 1.06 (s, 3H)ppm.

^{13}C NMR (101 MHz, CHLOROFORM-D) δ 206.4, 162.2, 144.5, 141.8, 139.5, 137.8, 132.0, 129.8, 128.5, 124.5, 114.5, 55.5, 41.8, 21.8, 18.2 ppm.

(Z)-1-(4-methoxyphenyl)-4-methyl-2-tosylpent-1-en-3-one:

¹H NMR (400 MHz, CHLOROFORM-D) δ 7.22 (d, *J* = 8.3 Hz, 2H), 7.00 (dd, *J* = 8.5, 0.6 Hz, 2H), 6.92 (dd, *J* = 5.4, 3.3 Hz, 3H), 6.58 (d, *J* = 8.8 Hz, 2H), 5.82 (d, *J* = 2.1 Hz, 1H), 3.74 (s, 3H), 2.33 (s, 3H), 1.49 (s, 3H), 1.43 (s, 3H) ppm.

¹³C NMR (101 MHz, CHLOROFORM-D) δ 159.9, 146.6, 144.0, 143.0, 136.4, 129.7, 129.3, 129.3, 127.9, 113.6, 86.9, 84.8, 55.3, 28.0, 26.9, 21.6 ppm.

3. X-ray Crystallographic Data for 3a

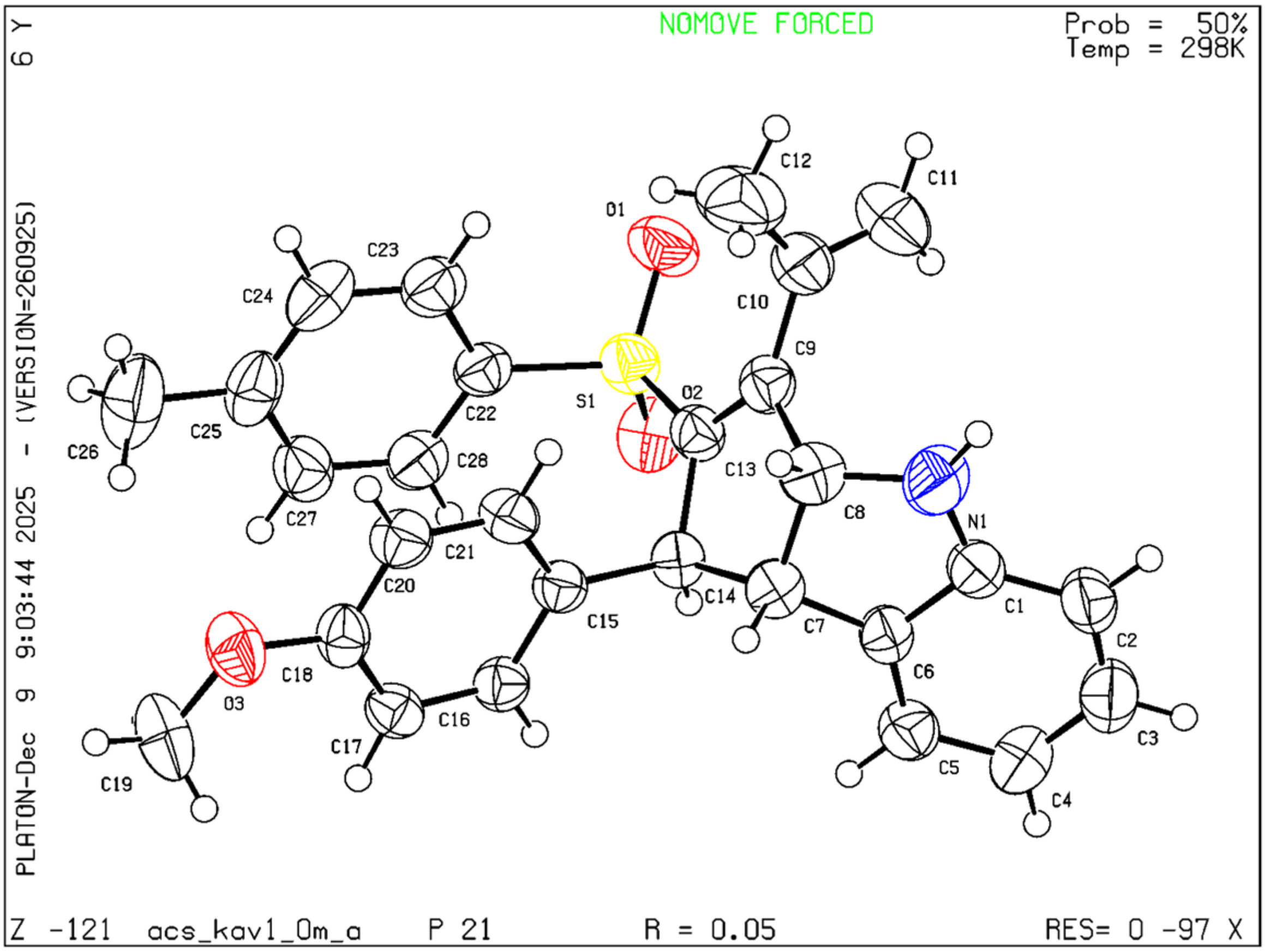

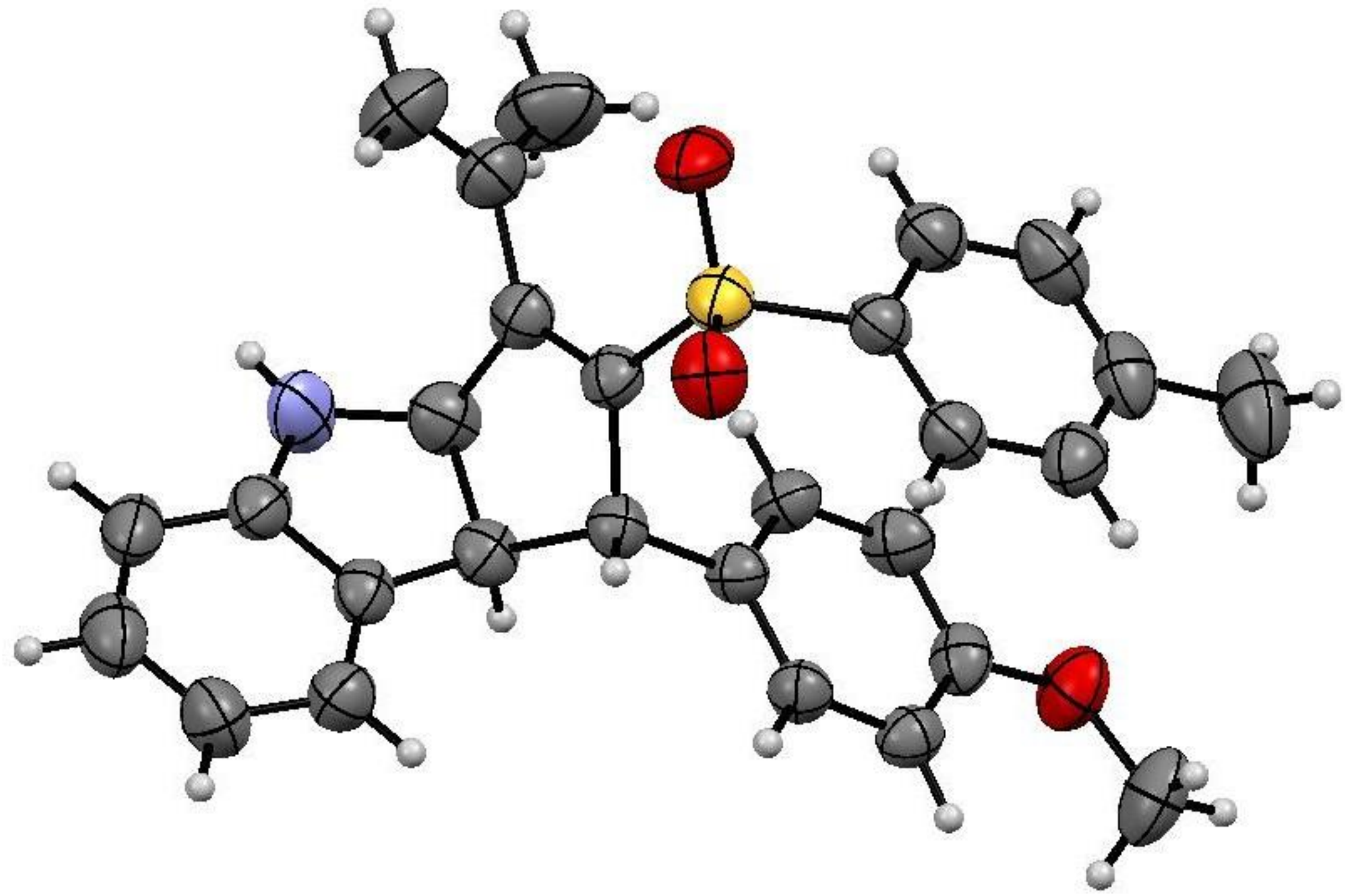

Figure S3: Molecular structure of **(1S)-1-(4-methoxyphenyl)-3-(prop-1-en-2-yl)-2-tosyl-1,3a,4,8b-tetrahydrocyclopenta[b]indole (3a)**. Gray, red, yellow, and purple spheres correspond to C, O, S, and N atoms, respectively. Hydrogen atoms are omitted for clarity. X-ray crystallographic coordinates have been deposited at the Cambridge Crystallographic Data Centre (CCDC) with the accession code **2514415**. X-ray derived ORTEP of **3a** with thermal ellipsoids shown at the 30% probability level.

Colorless crystal of **3a** were grown by slow evaporation using EtOAc and Hexanes at ambient temperature.

Table S1. Crystal data and structure refinement for **3a.**

**Table 1 Crystal data and structure refinement for ACS_KAV1_0m_a.**	
CCDC no.	2514415
Empirical formula	$C_{28}H_{27}NO_3S$
Formula weight	457.56
Temperature/K	298(2)
Crystal system	monoclinic
Space group	$P2_1$
a/Å	11.190(2)
b/Å	7.9615(14)
c/Å	13.430(2)
α/°	90
β/°	99.689(6)

$\gamma/°$	90
Volume/Å^3	1179.4(4)
Z	2
ρ_{calc}g/cm^3	1.288
μ/mm^{-1}	0.168
F(000)	484.0
Crystal size/mm^3	0.193 × 0.145 × 0.122
Radiation	MoKα (λ = 0.71073)
2Θ range for data collection/°	5.19 to 57.06
Index ranges	$-14 \leq h \leq 15$, $-10 \leq k \leq 10$, $-18 \leq l \leq 17$
Reflections collected	34314
Independent reflections	5957 [R_{int} = 0.0990, R_{sigma} = 0.0874]
Data/restraints/parameters	5957/1/302
Goodness-of-fit on F^2	0.954
Final R indexes [I>=2σ (I)]	R_1 = 0.0540, wR_2 = 0.1127
Final R indexes [all data]	R_1 = 0.1178, wR_2 = 0.1369
Largest diff. peak/hole / e Å^{-3}	0.17/-0.23

4. Computational studies on the mechanism

All calculations were conducted using a DFT method as implemented in the Gaussian 16 suite of programs.[12] For geometry optimization and frequency analysis, the BP86 functional[1311] level of theory in combination with Grimme's D3 dispersion corrections with a Becke-Johnson

[12] M. J. Frisch, G. W. Trucks, H. B. Schlegel, G. E. Scuseria, M. A. Robb, J. R. Cheeseman, G. Scalmani, V. Barone, G. A. Petersson, H. Nakatsuji, X. Li, M. Caricato, A. V. Marenich, J. Bloino, B. G. Janesko, R. Gomperts, B. Mennucci, H. P. Hratchian, J. V. Ortiz, A. F. Izmaylov, J. L. Sonnenberg, D. W. Young, F. Ding, F. Lipparini, F. Egidi, J. Goings, B. Peng, A. Petrone, T. Henderson, D. Ranasinghe, V. G. Zakrzewski, J. Gao, N. Rega, G. Zheng, W. Liang, M. Hada, M. Ehara, K. Toyota, R. Fukuda, J. Hasegawa, M. Ishida, T. Nakajima, Y. Honda, O. Kitao, H. Nakai, T. Vreven, K. Throssell, Jr. J. A.Montgomery, J. E.; Peralta, F. Ogliaro, M. J. Bearpark, J. J. Heyd, E. N. Brothers, K. N. Kudin, V. N. Staroverov, T. A. Keith, R. Kobayashi, J. Normand, K. Raghavachari, A. P. Rendell, J. C. Burant, S. S. Iyengar, J. Tomasi, M. Cossi, J. M. Millam, M. Klene, C. Adamo, R. Cammi, J. W. Ochterski, R. L. Martin, K. Morokuma, O. Farkas, J. B. Foresman, D. J. Fox, Gaussian 16, Revision C.02, Gaussian, Inc., Wallingford CT, 2019.

[13] J. P. Perdew, Phys. Rev. B, 1986, 33, 8822; (b) J. P. Perdew, Phys. Rev. B, 1986, 34, 7406; (c) A. Becke, Phys. Rev. A, 1988, 38, 3098.

damping scheme (D3BJ)[14], [15] in the gas phase. Geometry optimizations utilized the def2-SVP[16] split-valence plus single polarization basis set for non-metals and the Stuttgart/Dresden small core relativistic effective core potential (RECP) with a valence double-ζ basis set (SDD)[17] for Fe. No symmetry constraints were imposed during geometry optimizations. Transition states were verified by examining the normal mode corresponding to the imaginary frequency and performing intrinsic reaction coordinate (IRC) analysis16 to ensure proper connection between reactants and products on the potential energy surface. Single-point energy refinements were carried out using the hybrid-meta-GGA M06 functional[18] with the def2-TZVP[14, 19] basis set for nonmetals and SDD for Fe. Solvation energies were calculated implicitly for all intermediates and transition states using a self-consistent reaction field (SCRF) approach in dichloroethane solvent (ε = 10.125) and the SMD continuum solvation model.[20] Free energies (ΔG) reported throughout the article, unless stated otherwise, were determined at the M06(SMD)/SDD/def2-TZVP//BP86-D3(BJ)/SDD/def2-SVP level. The ΔG values were obtained by adding the in-solvent electronic energy (ΔE) at M06(SMD)/SDD/def2-TZVP to the free energy corrections computed at BP86-D3(BJ)/SDD/def2-SVP in the gas phase.

Table S2. Cartesian coordinates (Å) of the optimized structures of all intermediate and transition states at BP86-D3(BJ)/def2-SVP level of theory. E_e^S represents the absolute electronic energy in Hartree at the M06(SMD)/def2-TZVP level of theory in dichloroethane solvent.

Indole
E_e^S = -363.679369

C	19.343093000	17.533900000	5.218106000
C	19.343093000	16.866960000	6.466314000
C	19.343093000	17.653895000	7.669220000
C	19.343093000	19.061532000	7.647347000
C	19.343093000	19.688885000	6.395057000
C	19.343093000	18.932917000	5.193755000
C	19.343093000	15.482448000	6.872335000
C	19.343093000	15.466190000	8.257176000

[14] Grimme, S.; Antony, J.; Ehrlich, S.; Krieg, H. A consistent and accurate ab initio parametrization of density functional dispersion correction (DFT-D) for the 94 elements H-Pu. J. Chem. Phys. 2010, 132, 154104.
[15] Weigend, F. Accurate Coulomb-fitting basis sets for H to Rn. Phys. Chem. Chem. Phys. 2006, 8, 1057-1065.
[16] Weigend, F.; Ahlrichs, R. Balanced Basis Sets of Split Valence, Triple Zeta Valence and Quadruple Zeta Valence Quality for H To Rn: Design and Assessment of Accuracy. Phys.Chem. Chem. Phys. 2005, 7, 3297−3305.
[17] Häussermann, U.; Dolg, M.; Stoll, H.; Preuss, H.; Schwerdtfeger, P.; Pitzer, R. M. Accuracy of energy-adjusted quasirelativistic ab initio pseudopotentials. Mol. Phys. 1993, 78, 1211−1224.
[18] Zhao, Y.; Truhlar, D. G. The M06 Suite of Density Functionals for Main Group Thermochemistry, Thermochemical Kinetics, Non-Covalent Interactions, Excited States, and Transition Elements: Two New Functionals and Systematic Testing of Four M06-Class Functionals and 12 Other Functionals. Theor. Chem. Acc. 2008, 120, 215−241.
[19] a). Fukui, K. The path of chemical reactions-the IRC approach Acc. Chem. Res. 1981, 14, 363–368; b). Maeda, S.; Harabuchi, Y.; Ono, Y.; Taketsugu, T.; Morokuma, K. Intrinsic reaction coordinate: Calculation, bifurcation,and automated search. Int. J. Quantum Chem. 2015, 115, 258–269; c) Gonzalez, C.; Schlegel, H. B. Reaction path following in mass-weighted internal coordinates J. Phys. Chem. 1990, 94, 5523–5527.
[20] Marenich, A. V.; Cramer, C. J.; Truhlar, D. G. Universal Solvation Model Based on Solute Electron Density and on a Continuum Model of the Solvent Defined by the Bulk Dielectric Constant and Atomic Surface Tensions. J. Phys. Chem. B 2009, 113, 6378−6396

N	19.343093000	16.769510000	8.734682000
H	19.343093000	16.956185000	4.280285000
H	19.343093000	19.649184000	8.578884000
H	19.343093000	20.788885000	6.342228000
H	19.343093000	19.460982000	4.227330000
H	19.343093000	14.603267000	6.217942000
H	19.343093000	14.618865000	8.953529000
H	19.343093000	17.035260000	9.718054000

$FeCl_3$

E_e^S = -1504.558938

Fe	8.586933000	14.620340000	3.095559000
Cl	7.229172000	13.373146000	4.124575000
Cl	7.917258000	15.487082000	1.282247000
Cl	10.614920000	14.546413000	3.705068000

A

E_e^S = -1474.050935

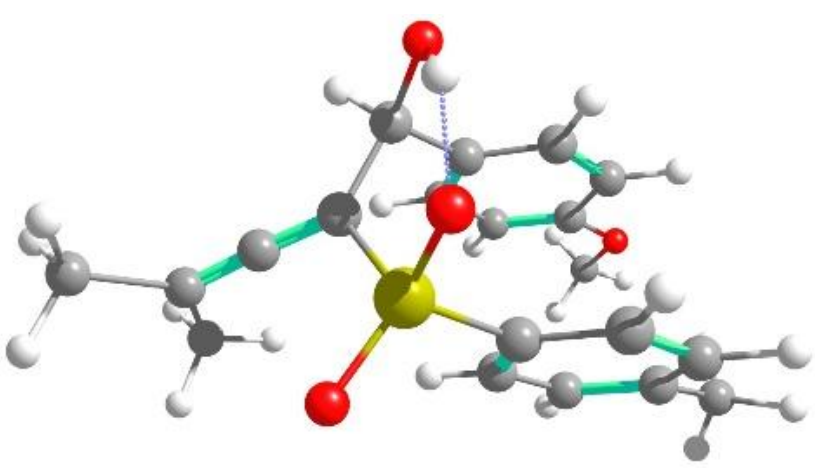

S	5.932730000	18.610777000	7.075461000
O	5.591075000	20.036682000	7.271812000
O	7.361449000	18.198182000	6.847645000
C	5.008401000	17.961148000	5.634807000
C	5.345336000	17.661012000	8.487193000
C	4.166771000	18.811098000	5.088326000
C	3.967928000	17.565560000	8.738089000
H	3.246620000	18.069178000	8.077867000
C	6.287682000	17.034879000	9.313172000
H	7.357996000	17.128341000	9.077481000
C	3.322059000	19.700174000	4.590380000
C	3.533938000	16.782843000	9.812600000
H	2.453608000	16.669557000	9.996321000
C	4.454257000	16.113335000	10.652023000

IN1

E_e^S = -2978.635225

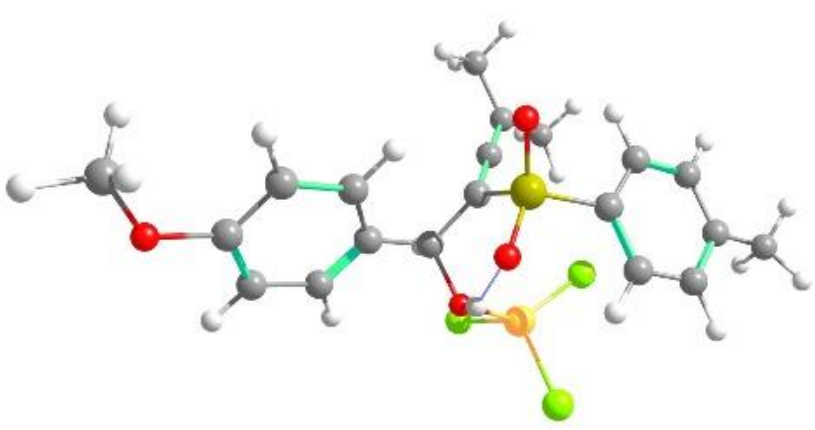

S	6.044558000	17.357053000	6.015080000

C	5.832665000	16.274267000	10.400188000
H	6.564116000	15.767254000	11.049752000
C	1.877473000	19.739798000	5.040918000
H	1.193744000	19.627138000	4.171476000
H	1.651655000	20.723070000	5.508132000
H	1.649061000	18.942767000	5.774223000
C	3.768882000	20.729459000	3.576069000
H	4.836316000	20.612575000	3.311146000
H	3.619697000	21.751920000	3.986265000
H	3.158986000	20.655529000	2.649488000
C	3.958189000	15.192811000	11.738736000
H	3.593618000	14.246078000	11.283656000
H	3.109415000	15.638464000	12.297314000
H	4.757522000	14.939337000	12.462493000
C	5.299535000	16.518450000	5.196401000
H	4.819954000	16.415396000	4.196130000
C	4.584099000	15.560823000	6.157878000
C	3.181305000	15.502151000	6.193059000
C	5.315972000	14.778362000	7.069779000
C	2.506329000	14.710241000	7.137157000
C	4.662681000	13.991712000	8.022407000
C	3.252938000	13.966351000	8.075370000
H	2.593878000	16.105415000	5.480511000
H	6.414274000	14.809696000	7.022137000
H	1.407768000	14.693484000	7.142943000
H	5.226017000	13.394189000	8.754264000
O	2.702499000	13.208039000	9.075401000
C	1.289454000	13.148867000	9.166229000
H	0.842196000	14.153784000	9.345763000
H	0.833134000	12.716339000	8.247004000
H	1.056934000	12.492713000	10.026092000
O	6.673774000	16.252646000	5.050971000
H	7.163282000	16.862268000	5.660426000

$FeCl_3(OH)$

E_e^S = -1474.050935

O	7.970220000	16.248482000	3.611146000
H	7.594302000	16.682542000	2.810599000
Fe	8.544134000	14.656279000	3.049464000
Cl	7.563346000	12.692624000	3.353413000
Cl	8.088867000	15.135589000	0.906077000
Cl	10.554220000	14.311370000	3.913959000

O	6.536824000	16.008246000	5.547077000
O	6.044455000	17.691774000	7.458698000
C	4.324587000	17.492895000	5.357874000
C	6.966056000	18.593439000	5.108383000
C	3.700767000	18.636198000	5.539582000
C	7.823449000	18.171641000	4.081265000
H	7.965134000	17.100007000	3.885407000
C	6.773366000	19.954090000	5.401262000
H	6.129074000	20.255630000	6.239338000
C	3.126812000	19.812572000	5.734772000
C	8.469962000	19.140840000	3.304433000
H	9.126082000	18.815854000	2.482280000

C	8.258261000	20.516889000	3.530847000
C	7.416212000	20.904579000	4.599910000
H	7.262418000	21.976148000	4.804587000
C	2.333871000	20.092499000	6.992269000
H	1.289235000	20.367732000	6.731584000
H	2.773930000	20.959501000	7.530893000
H	2.315027000	19.224000000	7.676925000
C	3.251237000	20.915250000	4.707349000
H	3.727919000	21.807969000	5.167596000
H	2.241852000	21.225969000	4.361803000
H	3.847623000	20.598228000	3.831190000
C	8.878847000	21.548052000	2.623275000
H	9.880860000	21.232730000	2.269769000
H	8.973699000	22.533769000	3.120203000
H	8.243278000	21.687922000	1.721446000
C	3.796834000	16.268500000	4.638465000
H	2.916927000	16.573240000	4.037191000
C	3.457625000	15.077410000	5.501124000
C	2.839982000	13.962185000	4.886675000
C	3.734344000	15.027310000	6.878049000
C	2.513651000	12.828969000	5.630066000
C	3.404733000	13.894918000	7.640343000
C	2.794589000	12.784756000	7.017529000
H	2.624069000	13.991523000	3.806849000
H	4.215606000	15.879454000	7.381894000
H	2.034802000	11.954306000	5.165675000
H	3.634720000	13.889407000	8.714279000
O	2.439562000	11.639724000	7.659691000
C	2.708368000	11.533908000	9.049974000
H	3.797576000	11.612861000	9.266371000
H	2.168525000	12.312534000	9.634998000
H	2.349423000	10.535131000	9.361140000
O	4.793845000	15.871964000	3.621644000
H	5.631304000	15.671010000	4.157024000
Fe	5.029683000	17.171134000	2.127786000
Cl	6.774924000	16.183109000	1.313651000
Cl	3.013206000	16.892102000	1.369060000
Cl	5.259812000	19.314368000	2.010456000

IN2

E_e^S = -1398.027428

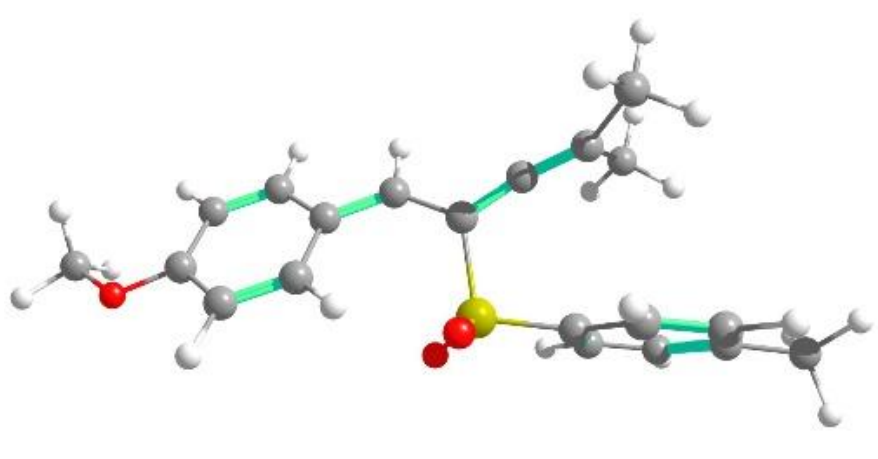

S	5.544753000	16.018961000	8.537709000
O	6.903031000	15.621213000	8.094545000
O	4.678656000	15.091490000	9.312875000
C	4.622709000	16.471486000	6.957695000
C	5.619484000	17.592432000	9.382103000
C	3.973406000	17.645260000	6.971251000
C	6.692014000	18.456567000	9.107429000
H	7.487669000	18.143670000	8.414931000
C	4.593229000	17.936919000	10.276699000
H	3.779651000	17.225238000	10.481068000
C	3.384033000	18.801421000	7.196680000
C	6.725279000	19.703240000	9.746662000
H	7.564336000	20.388893000	9.548854000
C	5.707339000	20.092700000	10.648801000
C	4.645673000	19.190224000	10.900536000
H	3.852459000	19.473240000	11.610740000
C	1.955511000	18.866969000	7.693859000
H	1.336385000	19.464048000	6.991982000
H	1.945617000	19.387142000	8.675532000
H	1.500167000	17.866803000	7.814883000
C	4.135989000	20.102271000	7.008117000
H	5.156442000	19.949808000	6.612141000
H	4.213085000	20.608552000	7.993784000
H	3.573467000	20.770903000	6.323424000
C	5.774164000	21.420376000	11.358829000
H	6.296172000	22.185977000	10.751648000
H	6.339880000	21.318319000	12.310813000
H	4.766376000	21.799584000	11.619246000
C	4.699076000	15.649483000	5.787847000
H	4.402498000	16.178076000	4.863863000
C	5.095698000	14.311043000	5.619487000
C	5.273127000	13.847405000	4.265521000
C	5.329257000	13.373029000	6.692573000
C	5.720392000	12.570118000	3.985257000
C	5.755739000	12.092390000	6.418240000
C	5.978777000	11.673721000	5.068661000
H	5.072114000	14.543722000	3.436126000
H	5.118933000	13.658703000	7.732865000
H	5.868912000	12.255310000	2.943881000
H	5.924872000	11.359236000	7.220096000
O	6.406884000	10.428386000	4.919468000
C	6.657264000	9.882856000	3.610082000
H	5.725064000	9.867244000	3.009053000
H	7.444566000	10.464973000	3.088788000
H	7.008000000	8.850538000	3.781468000

IN3

E_e^S = -1761.735862

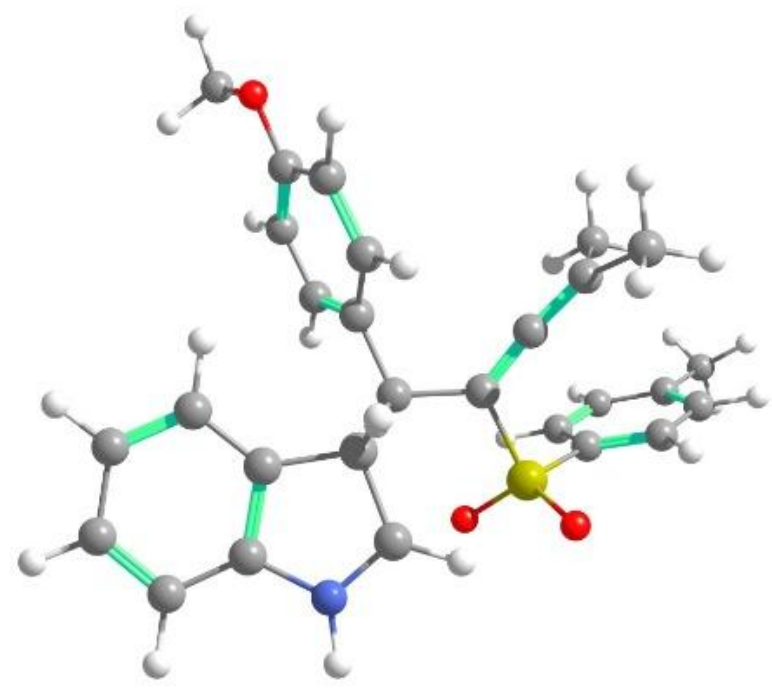

S	6.864178000	4.702783000	11.997642000
O	5.990701000	3.520301000	12.293274000
O	7.098743000	5.705029000	13.082382000
O	2.630592000	6.656707000	5.240308000

N	3.081393000	4.944066000	13.519511000
H	3.140963000	4.582659000	14.479953000
C	3.627603000	4.589004000	8.148456000
H	3.653374000	3.536742000	8.474320000
C	3.536509000	7.263997000	7.320552000
H	3.487383000	8.306371000	6.972971000
C	4.081892000	5.594190000	9.020613000
C	8.507677000	2.984098000	10.610264000
H	7.616314000	2.348661000	10.501138000
C	9.555366000	5.008740000	11.528713000
H	9.463631000	5.926600000	12.127315000
C	9.731148000	2.630071000	10.028068000
H	9.803051000	1.695752000	9.449015000
C	3.136330000	4.895684000	6.868831000
H	2.793977000	4.082852000	6.214340000
C	6.060160000	5.552847000	10.573967000
C	8.440214000	4.174901000	11.352231000
C	4.028388000	6.938642000	8.584819000
H	4.375264000	7.750415000	9.244450000
C	3.086964000	6.243186000	6.444499000
C	3.672224000	5.910820000	11.514341000
H	3.783881000	7.010396000	11.375546000
C	7.809531000	6.908721000	9.149128000
C	2.235053000	5.437870000	11.460309000
C	1.930838000	4.817562000	12.693862000
C	10.877703000	3.447215000	10.173767000
C	4.591103000	5.229914000	10.393525000
H	4.481404000	4.137514000	10.552210000
C	1.256250000	5.478896000	10.460704000
H	1.453355000	5.961478000	9.493704000
C	8.573714000	6.171868000	8.071494000
H	9.651187000	6.139001000	8.342344000
H	8.490242000	6.706923000	7.101723000
H	8.214583000	5.134063000	7.942294000
C	4.070990000	5.562423000	12.911380000
H	5.034172000	5.779936000	13.405886000
C	0.004023000	4.890218000	10.732989000
H	-0.780307000	4.917551000	9.961616000
C	-0.270065000	4.270686000	11.969917000
H	-1.258324000	3.820549000	12.146371000
C	6.856600000	6.289377000	9.827406000
C	0.699516000	4.224267000	12.987775000
H	0.493615000	3.749969000	13.958587000
C	2.161357000	5.683079000	4.311694000
H	1.846435000	6.241919000	3.411794000
H	1.290574000	5.120502000	4.715458000
H	2.964019000	4.964861000	4.033618000
C	10.767810000	4.635427000	10.932429000
H	11.653295000	5.277543000	11.063147000
C	12.193685000	3.037493000	9.563131000
H	12.051967000	2.534298000	8.585838000
H	12.719532000	2.316065000	10.226091000
H	12.868509000	3.903821000	9.419659000
C	8.180280000	8.346459000	9.427201000
H	7.587994000	8.781953000	10.253647000
H	9.257789000	8.411405000	9.690925000
H	8.033008000	8.964739000	8.515822000

IN3’

E_e^S = -1761.729024

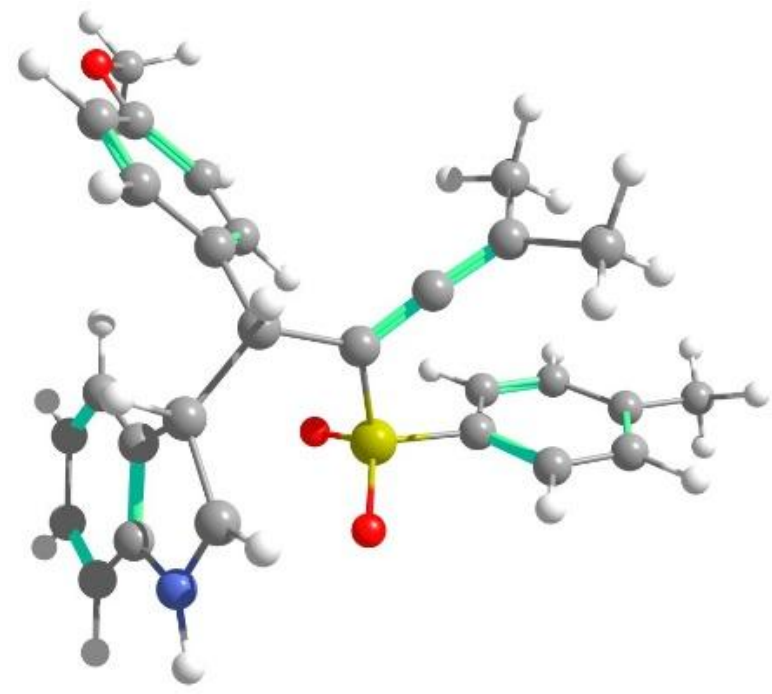

S	6.127046000	4.812805000	11.919560000
O	5.477702000	3.576244000	12.431816000
O	5.951226000	6.101241000	12.689731000
O	1.323473000	1.063545000	7.491577000
N	3.347890000	6.504907000	13.537014000
H	3.601420000	7.080873000	14.347675000
C	3.839374000	2.901976000	9.501227000
H	4.720777000	2.715893000	10.132844000
C	1.532826000	3.353986000	7.971919000
H	0.633107000	3.508633000	7.358550000
C	3.354626000	4.213651000	9.356947000
C	8.333478000	3.225344000	11.523070000
H	7.627445000	2.384131000	11.583457000
C	8.743908000	5.643922000	11.652807000
H	8.352520000	6.659630000	11.808570000
C	9.696973000	3.020334000	11.273447000
H	10.071242000	1.994100000	11.131753000
C	3.190946000	1.815077000	8.892643000
H	3.596277000	0.803454000	9.029597000
C	5.506348000	5.176960000	10.253177000
C	7.876946000	4.540325000	11.703545000
C	2.194023000	4.422937000	8.580378000
H	1.798862000	5.443520000	8.440914000
C	2.025726000	2.034110000	8.121491000
C	3.169754000	5.744850000	11.366915000
H	2.250348000	6.226334000	10.942443000
C	7.372781000	5.085990000	8.392049000
C	2.658088000	4.687723000	12.334803000
C	2.764014000	5.218751000	13.641233000
C	10.601909000	4.105324000	11.204965000
C	4.005920000	5.386733000	10.077539000
H	3.913777000	6.279620000	9.424128000
C	2.049590000	3.438764000	12.163386000
H	1.911923000	3.002273000	11.166708000
C	7.690425000	3.760508000	7.735486000
H	8.721013000	3.449410000	8.011667000
H	7.660114000	3.858797000	6.629601000
H	6.984921000	2.965290000	8.040811000
C	3.698350000	6.795826000	12.299915000
H	4.156384000	7.755813000	12.033287000
C	1.623647000	2.741968000	13.310868000
H	1.162683000	1.749899000	13.191906000
C	1.769536000	3.281730000	14.605101000

H	1.426533000	2.707508000	15.478557000
C	6.397706000	5.171808000	9.282790000
C	2.337924000	4.552349000	14.793961000
H	2.440615000	4.995949000	15.795203000
C	1.770375000	-0.284287000	7.597117000
H	1.058258000	-0.892653000	7.010364000
H	1.766003000	-0.635104000	8.653170000
H	2.792034000	-0.410174000	7.174971000
C	10.102385000	5.414738000	11.401795000
H	10.795404000	6.269989000	11.360069000
C	12.071604000	3.869834000	10.966786000
H	12.603625000	3.741730000	11.934914000
H	12.545687000	4.725333000	10.446346000
H	12.249832000	2.951570000	10.373313000
C	8.229815000	6.276637000	8.031013000
H	7.914663000	7.196966000	8.557292000
H	9.287684000	6.063040000	8.296392000
H	8.197609000	6.461204000	6.936123000

IN4

E_e^S = -1761.725413

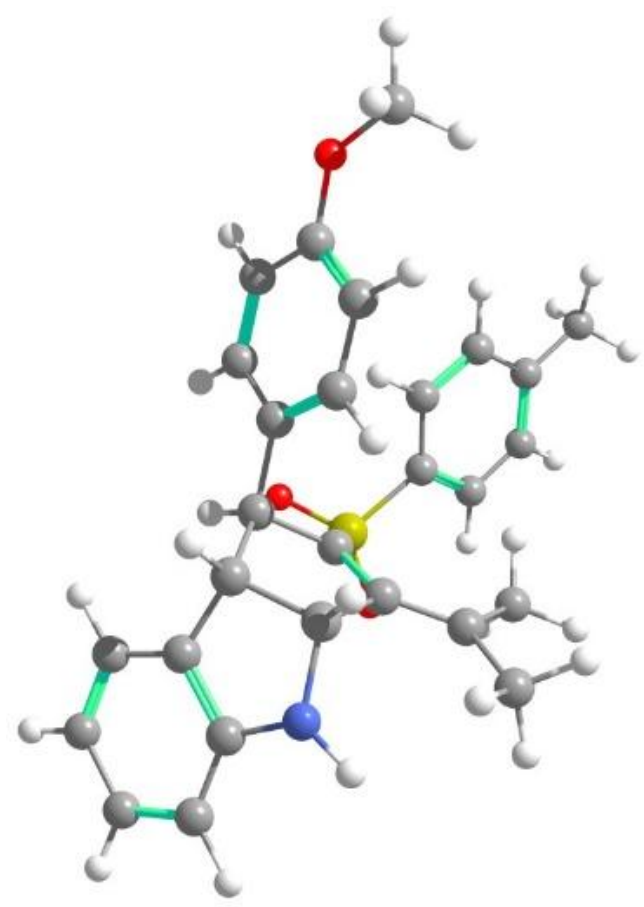

S	5.552075000	3.074281000	11.268641000
O	4.750580000	1.913512000	10.812365000
O	5.333653000	3.673181000	12.614139000
O	7.778698000	1.707584000	5.057902000
N	3.509004000	7.333982000	9.911725000
H	3.814413000	8.185686000	10.382307000
C	6.181281000	4.239563000	7.224680000
H	6.271378000	5.307031000	7.478386000
C	6.018524000	1.510632000	6.595759000
H	5.978845000	0.444027000	6.330498000
C	5.200916000	3.439767000	7.855191000
C	7.823474000	2.270908000	9.869250000
H	7.208689000	2.301157000	8.960213000
C	8.052988000	2.581085000	12.293383000
H	7.600604000	2.869329000	13.254072000
C	9.158620000	1.851394000	9.810639000
H	9.587652000	1.563151000	8.838139000
C	7.058021000	3.707537000	6.272242000
H	7.796235000	4.361838000	5.789652000
C	5.171489000	4.396688000	10.067661000
C	7.289055000	2.647105000	11.113704000
C	5.150172000	2.059263000	7.536257000
H	4.422359000	1.413955000	8.050217000
C	6.983196000	2.329332000	5.949256000
C	3.507966000	5.312271000	8.632367000
H	3.244187000	5.415664000	7.559459000
C	6.393918000	6.510306000	10.812731000
C	2.289426000	5.435780000	9.534075000
C	2.360446000	6.654780000	10.261555000
C	9.958686000	1.774722000	10.974399000
C	4.273574000	4.000661000	8.921883000
H	3.581598000	3.195408000	9.230525000
C	1.207734000	4.574276000	9.716061000
H	1.139733000	3.626417000	9.159625000
C	6.500479000	7.998540000	10.693471000
H	7.561864000	8.277353000	10.513614000
H	6.243494000	8.478147000	11.666062000
H	5.883073000	8.453242000	9.897734000
C	4.415319000	6.478496000	9.150142000
H	4.916159000	7.050855000	8.342710000
C	0.191246000	4.939585000	10.623531000
H	-0.669107000	4.271730000	10.777539000
C	0.268367000	6.156103000	11.337919000
H	-0.533274000	6.420377000	12.044149000
C	5.435635000	5.779770000	10.091494000
C	1.351103000	7.031519000	11.171943000
H	1.411461000	7.975580000	11.733530000
C	8.757671000	2.464235000	4.344692000
H	9.264636000	1.750497000	3.670974000
H	9.504876000	2.912033000	5.035663000
H	8.283584000	3.267558000	3.740286000
C	9.380380000	2.144033000	12.212565000
H	9.984234000	2.085088000	13.131966000
C	11.381031000	1.282320000	10.909045000
H	11.797910000	1.357629000	9.886001000
H	11.432536000	0.213188000	11.210731000
H	12.039785000	1.843486000	11.601808000
C	7.374958000	5.857246000	11.717680000
H	6.821556000	5.331238000	12.529645000
H	7.936242000	5.054230000	11.191626000
H	8.093508000	6.574901000	12.153993000

IN4’

E_e^S = -1761.724018

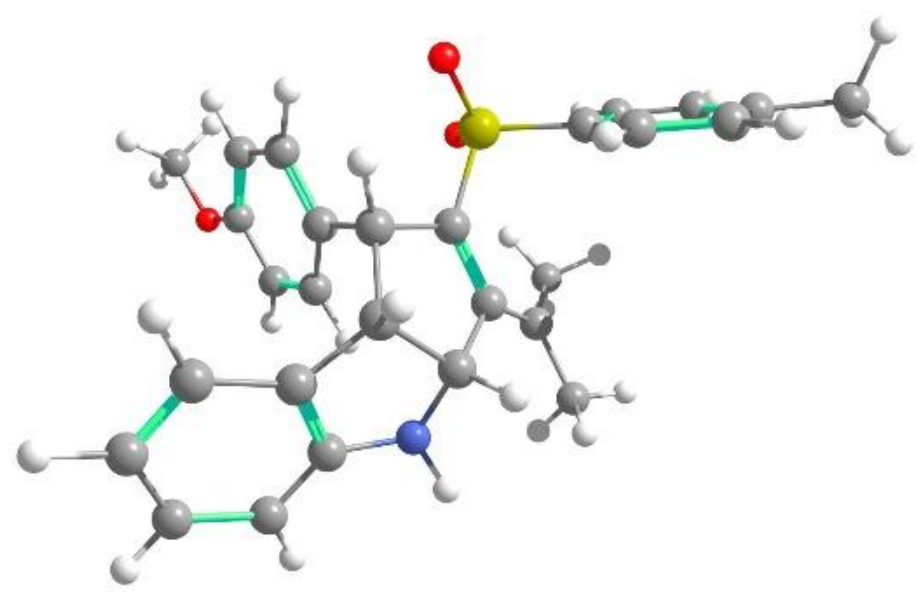

S	6.686770000	2.992883000	9.980746000
O	6.492487000	2.250250000	8.709806000

O	6.541518000	2.303392000	11.288415000
O	1.406113000	0.934023000	12.609446000
N	3.357736000	7.064574000	10.471792000
H	3.446434000	8.016826000	10.834215000
C	3.538042000	1.885558000	9.760173000
H	4.087642000	1.443539000	8.916634000
C	2.194333000	3.037028000	11.952308000
H	1.673878000	3.449574000	12.828338000
C	3.561647000	3.296999000	9.942844000
C	8.562494000	4.669283000	8.830704000
H	7.811657000	4.861998000	8.049182000
C	9.228769000	3.518962000	10.894554000
H	8.973021000	2.831576000	11.714655000
C	9.828122000	5.263050000	8.765641000
H	10.064873000	5.939702000	7.929326000
C	2.814002000	1.064132000	10.617041000
H	2.781439000	-0.017103000	10.428002000
C	5.470976000	4.369378000	9.875807000
C	8.272661000	3.817702000	9.911193000
C	2.917128000	3.849004000	11.091435000
H	3.000100000	4.921824000	11.315485000
C	2.124901000	1.635525000	11.725023000
C	3.793138000	5.578762000	8.607682000
H	4.113000000	5.816974000	7.573233000
C	6.151187000	6.153933000	11.557359000
C	2.315798000	5.817673000	8.804592000
C	2.130914000	6.720856000	9.878703000
C	10.817404000	4.993271000	9.743037000
C	4.332245000	4.159015000	8.940236000
H	4.588412000	3.553223000	8.053883000
C	1.214416000	5.288109000	8.123427000
H	1.357574000	4.585254000	7.287880000
C	6.126852000	7.613966000	11.891795000
H	7.096242000	7.929001000	12.329917000
H	5.361658000	7.799214000	12.680039000
H	5.892622000	8.265029000	11.028511000
C	4.467546000	6.544684000	9.648166000
H	5.007492000	7.373546000	9.137526000
C	-0.080559000	5.675981000	8.516642000
H	-0.958297000	5.276492000	7.987473000
C	-0.255957000	6.582741000	9.581451000
H	-1.273325000	6.883805000	9.875325000
C	5.459218000	5.673434000	10.444517000
C	0.842604000	7.113243000	10.280728000
H	0.695609000	7.812865000	11.117420000
C	1.304668000	-0.490347000	12.482794000
H	0.668808000	-0.822964000	13.322107000
H	2.303513000	-0.968868000	12.561145000
H	0.825282000	-0.771022000	11.521197000
C	10.496615000	4.111132000	10.799684000
H	11.256690000	3.881114000	11.562901000
C	12.190677000	5.603635000	9.631728000
H	12.816838000	5.015851000	8.925324000
H	12.716577000	5.615302000	10.606080000
H	12.147884000	6.639490000	9.239817000
C	6.904266000	5.284888000	12.502397000
H	6.647768000	4.213230000	12.422947000
H	7.997603000	5.396696000	12.316325000
H	6.742451000	5.635314000	13.544191000

IN4”

E_e^S = -1761.688392

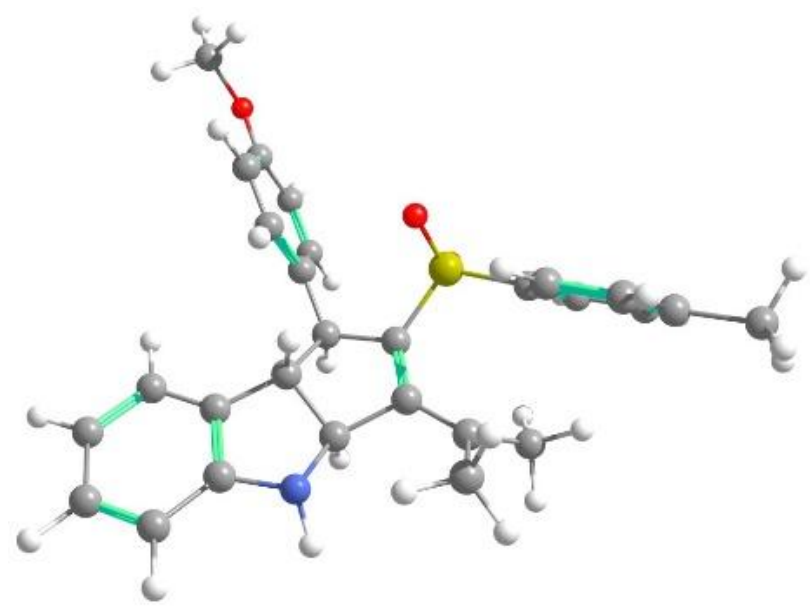

S	7.545008000	3.725068000	10.386915000
O	7.955641000	3.843087000	8.965420000
O	7.334928000	2.417378000	11.054035000
O	5.949500000	-0.268941000	5.981872000
N	3.641925000	7.373778000	9.873657000
H	3.289354000	8.004490000	10.601888000
C	5.528097000	3.151550000	7.324208000
H	5.641907000	4.197868000	7.004341000
C	5.285729000	0.470707000	8.107536000
H	5.203141000	-0.588816000	8.389889000
C	5.117039000	2.849397000	8.639064000
C	9.235636000	5.870437000	10.767080000
H	8.928078000	6.176333000	9.755839000
C	9.138882000	4.221180000	12.585844000
H	8.729504000	3.278799000	12.980170000
C	10.171622000	6.611547000	11.500587000
H	10.590588000	7.535025000	11.070273000
C	5.808808000	2.141177000	6.397013000
H	6.120835000	2.414670000	5.380397000
C	5.946348000	4.563767000	10.465656000
C	8.712153000	4.695060000	11.336042000
C	4.997201000	1.491530000	9.011195000
H	4.688273000	1.232904000	10.035735000
C	5.696506000	0.785816000	6.786585000
C	4.223603000	5.252107000	9.073958000
H	5.017044000	5.632690000	8.386922000
C	6.366594000	6.820118000	11.683011000
C	2.913355000	5.639865000	8.450418000
C	2.677247000	6.974067000	8.898437000
C	10.612511000	6.177454000	12.774217000
C	4.794563000	3.928535000	9.645641000
H	4.065987000	3.526917000	10.387441000
C	2.092234000	5.063963000	7.481035000
H	2.288727000	4.043669000	7.118460000
C	6.378645000	8.244739000	11.255158000
H	7.324053000	8.422500000	10.688922000
H	6.380532000	8.953512000	12.107525000
H	5.567774000	8.469346000	10.526267000
C	4.263601000	6.144121000	10.309490000
H	3.633781000	5.671700000	11.109760000
C	1.004602000	5.815343000	6.984774000
H	0.329644000	5.370287000	6.238535000

C	0.776013000	7.130700000	7.433157000
H	-0.076569000	7.700163000	7.032938000
C	5.697580000	5.877038000	10.854271000
C	1.613313000	7.734116000	8.390946000
H	1.424416000	8.760577000	8.740389000
C	6.379278000	-0.032781000	4.643080000
H	6.528070000	-1.028543000	4.187861000
H	7.338176000	0.529715000	4.619413000
H	5.611043000	0.524619000	4.063335000
C	10.087684000	4.968313000	13.296066000
H	10.438061000	4.606269000	14.275595000
C	11.656600000	6.950819000	13.536188000
H	12.665426000	6.526172000	13.339274000
H	11.491652000	6.894445000	14.630469000
H	11.684817000	8.015900000	13.234449000
C	6.872669000	6.477049000	13.037310000
H	6.806566000	5.401408000	13.269308000
H	7.914957000	6.838045000	13.190865000
H	6.258245000	7.039554000	13.780824000

3a

E_e^S = -1761.326481

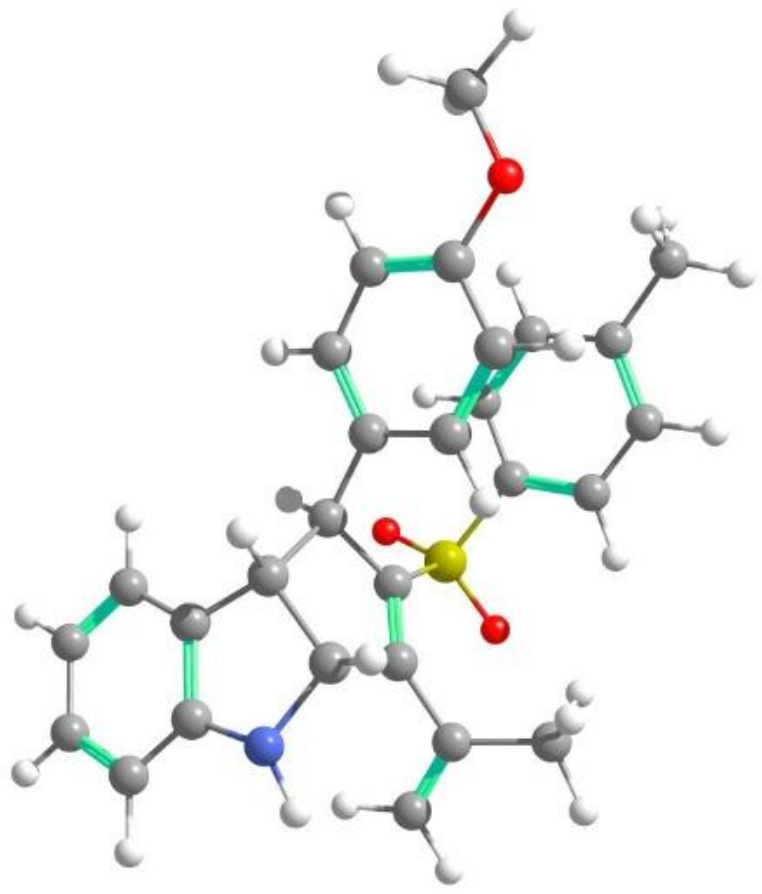

S	6.628616000	2.656122000	10.254136000
O	5.620242000	1.575420000	10.089921000
O	7.589599000	2.661837000	11.386788000
O	6.700090000	5.356139000	3.982664000
N	4.010579000	6.727425000	11.994355000
H	4.519998000	6.887467000	12.870819000
C	4.475766000	4.155162000	6.706139000
H	3.581566000	3.528619000	6.857385000
C	6.767397000	5.708056000	6.313243000
H	7.676346000	6.302395000	6.139397000
C	5.129075000	4.699134000	7.823712000
C	7.035983000	2.200361000	7.554144000
H	6.092610000	1.639230000	7.613509000
C	8.789073000	3.463016000	8.702524000
H	9.203264000	3.870091000	9.636493000
C	7.707394000	2.389540000	6.341202000
H	7.279868000	1.971445000	5.416004000
C	4.950930000	4.363960000	5.398697000
H	4.419692000	3.906526000	4.552866000
C	5.707239000	4.202610000	10.256656000
C	7.575406000	2.761813000	8.721535000
C	6.275636000	5.493418000	7.601185000
H	6.817037000	5.915945000	8.462111000
C	6.115793000	5.131626000	5.200152000
C	3.840068000	5.634013000	9.858997000
H	3.485203000	6.327494000	9.067357000
C	6.756075000	5.334076000	12.328263000
C	2.708465000	5.238901000	10.791630000
C	2.885435000	5.894535000	12.035414000
C	8.908942000	3.131063000	6.277560000
C	4.612671000	4.430037000	9.228703000
H	3.958357000	3.534221000	9.192845000
C	1.620619000	4.388329000	10.588631000
H	1.485995000	3.874568000	9.622832000
C	8.184175000	5.724005000	12.034480000
H	8.699255000	4.854651000	11.575180000
H	8.727424000	6.000997000	12.959171000
H	8.239842000	6.570939000	11.317621000
C	4.832689000	6.319512000	10.852287000
H	5.370102000	7.196612000	10.428337000
C	0.701284000	4.180999000	11.639818000
H	-0.156643000	3.507005000	11.495817000
C	0.888477000	4.828183000	12.874594000
H	0.172277000	4.654846000	13.693457000
C	5.844711000	5.195803000	11.166391000
C	6.270317000	5.167595000	13.580431000
H	6.920049000	5.303849000	14.460269000
H	5.242803000	4.811241000	13.756018000
C	1.979928000	5.692366000	13.091298000
H	2.120694000	6.192314000	14.062075000
C	6.084823000	4.794489000	2.834523000
H	6.703887000	5.095005000	1.968481000
H	5.049686000	5.179006000	2.691540000
H	6.048264000	3.682207000	2.886619000
C	9.449245000	3.641160000	7.477852000
H	10.398160000	4.200501000	7.448306000
C	9.557346000	3.410167000	4.944561000
H	9.611757000	2.495874000	4.318043000
H	10.582311000	3.814224000	5.059486000
H	8.954747000	4.157578000	4.383549000

3a’

E_e^S = -1761.313501

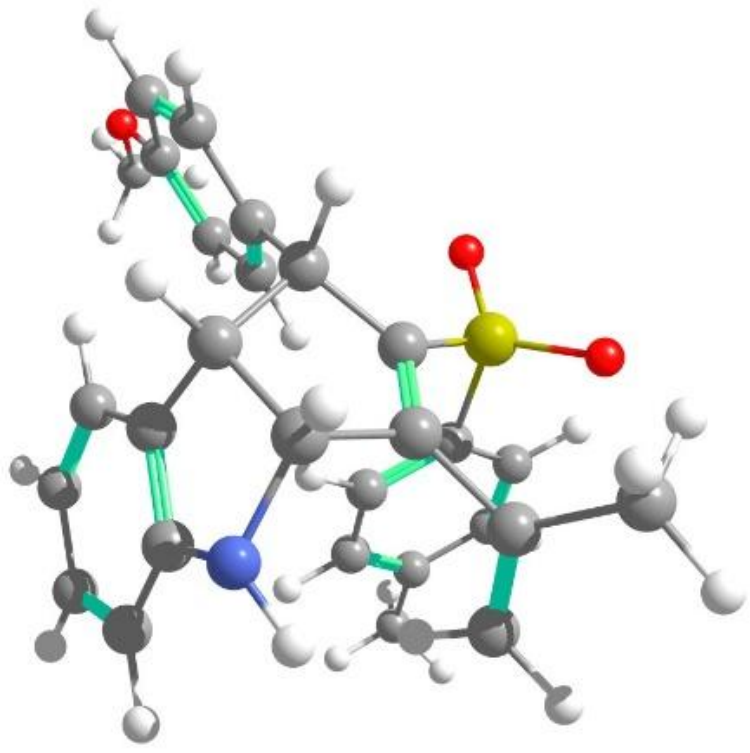

S	7.022363000	2.671436000	10.512780000
O	8.239271000	3.266747000	11.130896000
O	7.186022000	1.732836000	9.376235000
O	2.941497000	-0.242376000	5.698745000
N	3.510119000	6.150932000	11.264325000
H	3.819382000	6.386863000	12.214805000
C	4.314758000	1.637913000	8.594074000
H	4.502339000	1.486042000	9.662532000
C	3.790684000	1.951451000	5.862809000
H	3.583826000	2.054832000	4.787287000
C	4.624584000	2.864448000	7.983378000
C	6.872329000	1.000469000	12.679198000
H	7.958261000	0.914634000	12.522269000
C	4.732010000	1.999144000	12.052460000
H	4.134921000	2.682069000	11.430474000
C	6.233459000	0.298354000	13.708600000
H	6.828899000	-0.360569000	14.361596000
C	3.756761000	0.572992000	7.869068000
H	3.540715000	-0.368375000	8.392065000
C	5.891269000	3.989451000	10.034975000
C	6.112703000	1.845451000	11.851574000
C	4.357327000	2.993655000	6.601818000
H	4.596252000	3.941802000	6.091738000
C	3.484470000	0.724810000	6.493653000
C	3.971881000	5.084593000	9.147496000
H	3.641934000	5.685453000	8.273827000
C	6.341015000	5.390644000	12.147865000
C	2.789055000	4.446745000	9.865605000
C	2.623287000	5.077889000	11.126524000
C	4.842518000	0.427004000	13.932858000
C	5.116686000	4.089961000	8.724441000
H	5.777446000	4.639060000	8.014119000
C	1.914623000	3.425609000	9.475707000
H	2.000773000	2.963402000	8.483070000
C	7.615316000	6.192942000	12.042097000
H	8.387597000	5.563916000	11.553071000
H	7.978806000	6.506344000	13.040451000
H	7.471091000	7.099112000	11.414597000
C	4.591170000	5.956047000	10.289797000
H	5.004759000	6.930990000	9.950869000
C	0.925381000	2.981268000	10.379501000
H	0.246589000	2.166044000	10.086814000
C	0.809183000	3.572880000	11.650213000
H	0.040858000	3.211996000	12.352597000
C	5.692635000	5.047993000	10.857249000
C	5.758283000	5.050036000	13.320448000
H	6.215742000	5.339150000	14.280689000
H	4.853389000	4.424024000	13.356344000
C	1.647842000	4.636088000	12.037826000
H	1.538101000	5.114400000	13.023282000
C	2.632953000	-1.494364000	6.287646000
H	2.214631000	-2.125390000	5.480836000
H	1.876208000	-1.396022000	7.099593000
H	3.538638000	-1.991244000	6.704241000
C	4.109405000	1.289601000	13.092235000
H	3.027084000	1.422812000	13.249241000
C	4.170181000	-0.344270000	15.043340000
H	4.682582000	-0.183536000	16.015110000
H	4.197067000	-1.437132000	14.843073000
H	3.109047000	-0.049359000	15.161427000

3a”

E_e^S = -1761.297799

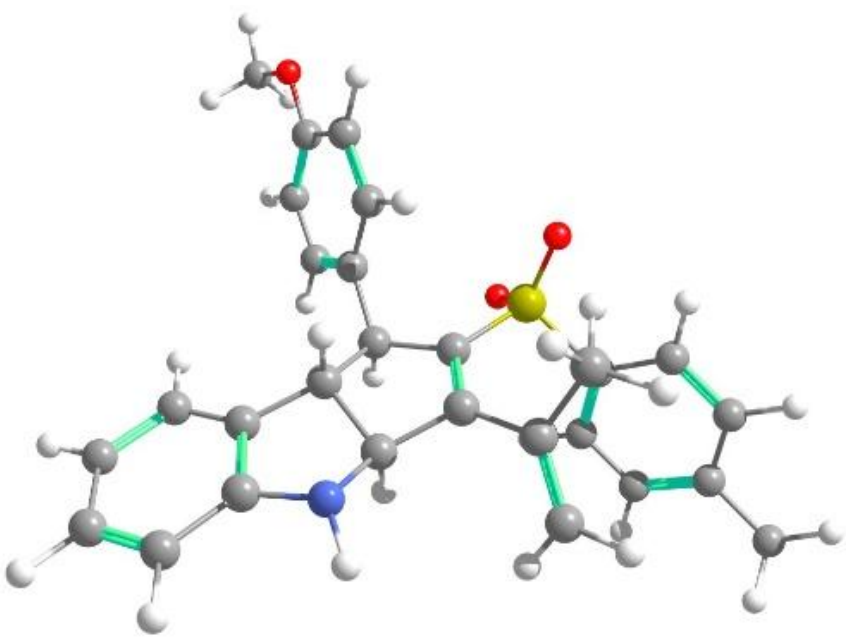

S	6.214424000	2.837849000	10.075796000
O	7.634527000	3.269413000	9.935666000
O	5.543941000	2.057465000	9.004562000
O	6.499980000	5.445627000	4.094953000
N	3.315435000	6.961829000	12.009136000
H	3.016015000	6.946097000	12.988389000
C	4.051644000	4.554469000	6.741177000
H	3.031023000	4.153590000	6.853380000
C	6.654182000	5.536998000	6.445476000
H	7.680203000	5.908634000	6.306638000
C	4.801525000	4.858566000	7.888743000
C	7.211922000	1.680876000	12.397072000
H	8.181981000	2.074123000	12.059233000
C	4.818696000	1.389519000	11.984416000
H	3.945931000	1.547395000	11.332808000
C	7.077440000	0.979214000	13.606085000
H	7.965324000	0.810934000	14.236655000
C	4.576098000	4.737982000	5.450299000
H	3.959686000	4.483484000	4.577192000
C	5.150557000	4.255484000	10.380333000
C	6.075275000	1.893960000	11.610618000
C	6.113053000	5.355354000	7.720145000
H	6.732610000	5.573761000	8.604209000
C	5.888442000	5.233793000	5.297121000
C	3.754066000	6.042517000	9.920025000
H	4.648866000	6.710046000	9.837094000
C	5.654618000	4.710669000	12.874073000

C	2.555092000	6.941986000	9.785382000
C	2.391921000	7.518358000	11.086266000
C	5.824645000	0.490697000	14.030767000
C	4.192847000	4.718591000	9.266089000
H	3.332224000	4.011875000	9.214798000
C	1.769915000	7.369529000	8.714784000
H	1.918829000	6.937472000	7.713003000
C	7.140847000	4.980855000	12.852494000
H	7.627720000	4.483289000	11.990428000
H	7.627047000	4.656512000	13.792938000
H	7.317127000	6.071089000	12.721212000
C	3.673766000	5.679528000	11.404756000
H	2.819626000	4.953638000	11.532770000
C	0.787155000	8.359139000	8.939346000
H	0.146789000	8.695227000	8.109519000
C	0.626214000	8.919182000	10.219207000
H	-0.143100000	9.690760000	10.381718000
C	4.939750000	4.865374000	11.589589000
C	4.969900000	4.409559000	14.003007000
H	5.488833000	4.290993000	14.967135000
H	3.882659000	4.231951000	13.989271000
C	1.425571000	8.511334000	11.307426000
H	1.286781000	8.956966000	12.305056000
C	5.779061000	5.138909000	2.914640000
H	6.449565000	5.376296000	2.067088000
H	4.851189000	5.749329000	2.823217000
H	5.501626000	4.060936000	2.865486000
C	4.700328000	0.701331000	13.196130000
H	3.716444000	0.312741000	13.505789000
C	5.675408000	-0.253279000	15.335969000
H	6.624904000	-0.276079000	15.905663000
H	5.358194000	-1.303954000	15.162661000
H	4.899932000	0.214194000	15.978766000

TS1

E_e^S = -1761.713084

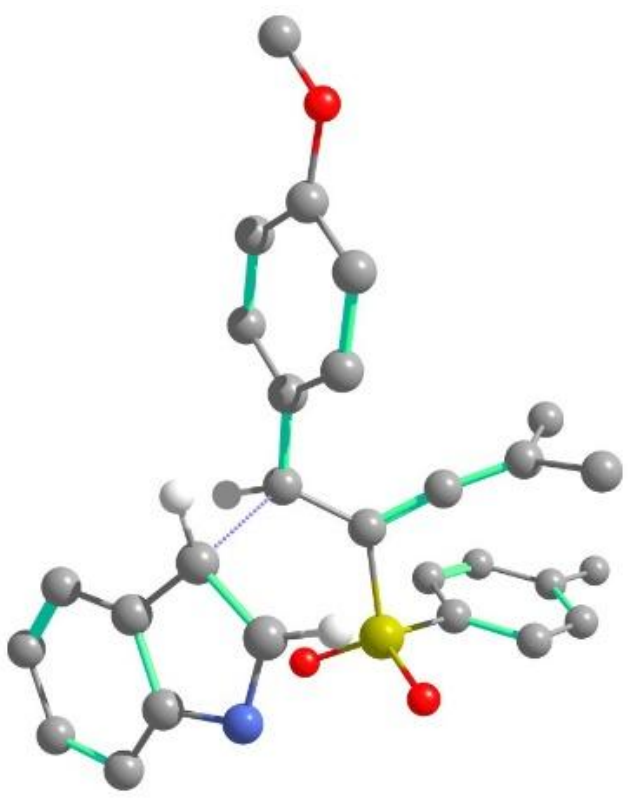

S	6.982560000	4.577080000	11.852346000
O	6.116995000	3.386029000	12.100132000
O	7.197522000	5.579176000	12.936951000
O	2.265850000	6.224117000	5.239167000
N	3.184356000	4.812943000	13.203509000
H	3.288065000	4.196324000	14.015059000
C	3.217137000	4.504375000	8.364338000
H	2.998150000	3.588749000	8.935367000
C	3.757546000	6.854767000	6.933977000
H	3.932321000	7.773604000	6.355279000
C	4.171960000	5.422369000	8.872742000
C	8.661697000	2.924379000	10.421769000
H	7.766375000	2.313769000	10.232091000
C	9.698476000	4.866695000	11.511702000
H	9.591938000	5.748366000	12.160326000
C	9.905635000	2.580752000	9.877847000
H	9.989129000	1.682068000	9.246298000
C	2.567800000	4.720072000	7.144741000
H	1.853167000	3.973734000	6.773387000
C	6.228329000	5.461144000	10.391100000
C	8.578081000	4.070166000	11.230066000
C	4.413502000	6.615195000	8.134715000
H	5.123225000	7.362173000	8.519065000
C	2.834361000	5.903852000	6.415622000
C	3.734396000	6.229597000	11.517997000
H	4.122266000	7.201671000	11.184780000
C	8.099491000	6.879253000	9.196647000
C	2.352481000	5.773525000	11.314775000
C	2.067186000	4.832510000	12.344122000
C	11.059027000	3.361484000	10.131186000
C	4.821442000	5.124068000	10.143835000
H	4.565056000	4.137775000	10.570462000
C	1.394343000	6.011521000	10.316615000
H	1.585257000	6.738793000	9.514185000
C	8.959046000	6.193876000	8.156487000
H	10.003216000	6.132303000	8.531668000
H	8.975421000	6.785115000	7.216409000
H	8.608272000	5.169215000	7.933811000
C	4.136853000	5.654905000	12.774672000
H	5.088817000	5.785583000	13.310270000
C	0.178520000	5.305378000	10.377375000
H	-0.590931000	5.487869000	9.612189000
C	-0.078398000	4.369319000	11.402831000
H	-1.039446000	3.833745000	11.420035000
C	7.073582000	6.240883000	9.735621000
C	0.867727000	4.113981000	12.412015000
H	0.667012000	3.389735000	13.215304000
C	1.325806000	5.324548000	4.648304000
H	1.007777000	5.794262000	3.700412000
H	0.439206000	5.180706000	5.303122000
H	1.792312000	4.339069000	4.432927000
C	10.933162000	4.503211000	10.955762000
H	11.823172000	5.115714000	11.171001000
C	12.396426000	2.959966000	9.563174000
H	12.295941000	2.523851000	8.549207000
H	12.871182000	2.184873000	10.203718000
H	13.096116000	3.816861000	9.511484000
C	8.459453000	8.290350000	9.601755000
H	7.790746000	8.687920000	10.387890000
H	9.503100000	8.309590000	9.982628000
H	8.423660000	8.966437000	8.720802000

TS1’

E_e^S = -1761.699417

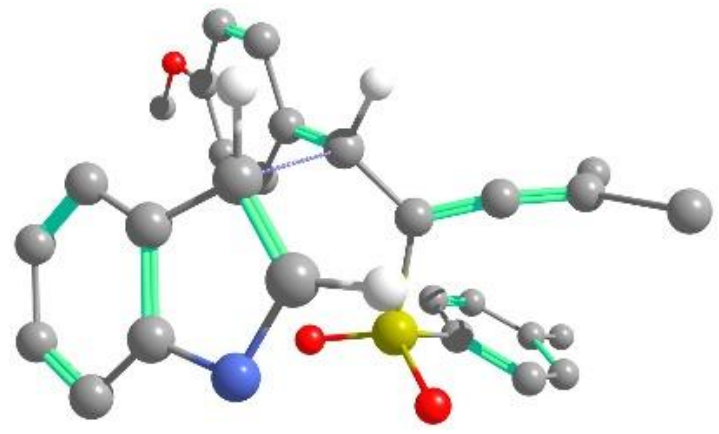

S	6.330245000	4.840069000	11.753483000
O	5.502273000	3.772708000	12.381603000
O	6.533399000	6.123738000	12.491602000
O	1.683416000	0.240576000	8.333525000
N	3.743816000	6.283161000	12.995467000
H	4.475405000	6.511442000	13.678368000
C	3.876261000	2.621105000	10.116948000
H	4.554840000	2.672919000	10.980023000
C	2.031527000	2.532541000	7.991534000
H	1.307833000	2.464580000	7.166276000
C	3.613548000	3.803012000	9.374317000
C	8.036790000	2.908418000	10.759054000
H	7.135273000	2.296072000	10.606751000
C	9.075479000	4.990902000	11.537100000
H	8.960098000	5.986255000	11.990359000
C	9.298884000	2.432711000	10.382126000
H	9.386337000	1.430505000	9.933422000
C	3.261342000	1.410217000	9.792338000
H	3.491458000	0.517527000	10.389257000
C	5.632008000	5.297198000	10.086614000
C	7.944332000	4.188685000	11.329265000
C	2.656257000	3.728104000	8.317523000
H	2.422086000	4.633449000	7.733976000
C	2.332776000	1.350961000	8.723426000
C	2.839272000	6.056153000	10.925723000
H	2.297282000	6.501645000	10.080949000
C	7.609942000	6.401043000	8.734604000
C	2.271626000	5.024759000	11.801628000
C	2.900757000	5.159957000	13.070111000
C	10.464913000	3.212430000	10.572847000
C	4.230788000	5.094496000	9.635570000
H	4.028481000	5.816749000	8.826116000
C	1.378384000	3.963163000	11.588323000
H	0.877788000	3.829952000	10.618224000
C	8.490139000	5.504062000	7.892592000
H	9.505995000	5.470663000	8.342406000
H	8.593427000	5.912776000	6.865253000
H	8.101117000	4.470766000	7.834696000
C	3.699216000	6.833366000	11.772258000
H	4.325295000	7.696809000	11.519399000
C	1.140746000	3.069969000	12.649400000
H	0.439784000	2.234216000	12.504045000
C	1.781851000	3.222476000	13.897894000
H	1.575202000	2.503631000	14.704915000
C	6.543333000	5.908833000	9.344745000
C	2.680286000	4.278002000	14.132010000
H	3.190618000	4.395259000	15.098744000
C	1.930548000	-0.992858000	9.012772000
H	1.297596000	-1.747918000	8.513327000
H	1.647197000	-0.925749000	10.085269000
H	2.997527000	-1.291454000	8.928129000
C	10.329928000	4.493214000	11.155572000
H	11.227246000	5.111763000	11.316198000
C	11.819846000	2.672215000	10.192158000
H	12.217741000	2.020862000	11.000770000
H	12.556353000	3.483606000	10.031951000
H	11.770498000	2.053774000	9.273715000
C	8.010187000	7.850143000	8.886838000
H	7.328276000	8.408702000	9.554545000
H	9.038587000	7.905536000	9.304026000
H	8.034664000	8.351020000	7.895560000

TS2

E_e^S = -1761.718915

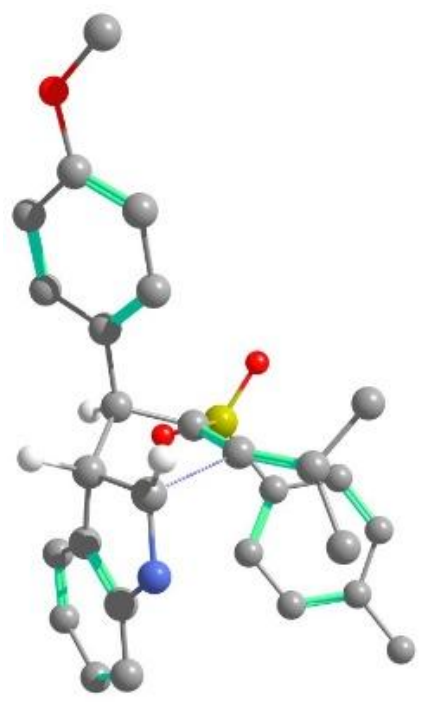

S	7.150489000	2.208326000	10.378878000
O	8.348314000	1.798958000	9.605716000
O	6.080203000	1.246822000	10.747902000
O	5.744715000	2.430933000	3.041841000
N	4.388043000	6.335264000	10.179149000
H	4.576508000	7.326448000	10.352776000
C	6.183023000	3.824371000	6.444758000
H	6.766510000	4.596851000	6.969836000
C	4.776611000	1.815113000	5.090357000
H	4.243417000	1.027289000	4.538415000
C	5.308644000	2.990352000	7.169055000
C	9.047075000	3.332901000	12.051613000
H	9.774820000	2.949865000	11.320278000
C	6.709088000	3.545798000	12.765170000
H	5.643210000	3.332665000	12.593715000
C	9.447857000	4.052631000	13.188781000
H	10.519984000	4.239062000	13.360565000
C	6.358855000	3.677240000	5.060405000
H	7.055962000	4.340599000	4.530983000
C	6.303427000	3.524905000	9.430197000
C	7.680052000	3.096730000	11.854048000
C	4.616512000	1.976536000	6.465757000
H	3.941898000	1.298434000	7.013096000
C	5.650781000	2.666597000	4.369109000
C	4.101771000	4.309525000	9.058491000
H	3.241653000	4.369433000	8.350887000
C	7.740695000	5.611335000	9.895566000
C	3.619848000	4.211789000	10.496935000

C	3.786575000	5.471326000	11.119374000
C	8.501189000	4.532556000	14.123346000
C	5.063544000	3.158815000	8.657285000
H	4.657710000	2.207276000	9.055090000
C	3.115394000	3.138752000	11.236530000
H	2.999466000	2.141029000	10.788756000
C	9.041817000	5.501042000	9.152699000
H	9.886014000	5.583883000	9.869802000
H	9.157245000	6.346880000	8.437306000
H	9.115652000	4.545869000	8.599908000
C	4.808365000	5.662074000	9.078233000
H	5.093046000	6.224407000	8.177068000
C	2.777167000	3.358197000	12.587666000
H	2.383950000	2.524371000	13.188041000
C	2.934183000	4.627347000	13.184101000
H	2.656569000	4.768600000	14.239487000
C	6.704728000	4.790842000	9.551866000
C	3.449090000	5.715549000	12.456226000
H	3.585849000	6.702876000	12.921664000
C	6.623458000	3.233297000	2.258497000
H	6.538891000	2.860629000	1.221594000
H	7.677875000	3.132301000	2.598179000
H	6.329132000	4.305886000	2.286041000
C	7.128933000	4.268543000	13.888664000
H	6.377855000	4.627063000	14.610562000
C	8.942425000	5.268683000	15.363069000
H	9.079723000	4.555682000	16.205237000
H	8.190083000	6.013985000	15.689483000
H	9.910661000	5.785455000	15.213332000
C	7.628746000	6.626625000	10.986558000
H	6.583158000	6.839995000	11.273879000
H	8.111389000	6.189149000	11.892838000
H	8.173956000	7.563123000	10.748591000

TS2'

E_e^S = -1761.719114

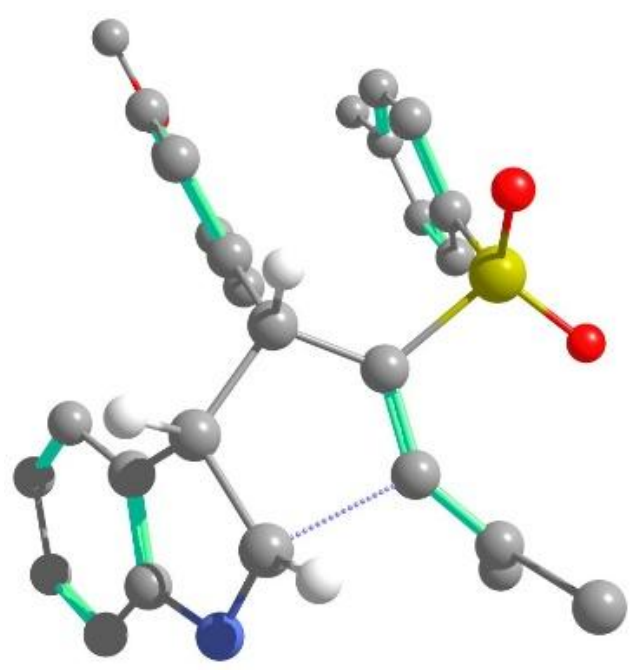

S	2.313807000	-0.362513000	1.601009000
O	3.356477000	-1.404913000	1.386835000
O	2.093350000	0.278409000	2.922510000
O	-0.909176000	3.989868000	-2.122938000
N	-1.858447000	-3.775242000	0.742395000
H	-1.789482000	-4.795642000	0.794606000
C	-1.223753000	1.970190000	0.969388000
H	-1.523533000	2.075555000	2.024686000
C	-0.425435000	1.728757000	-1.707128000
H	-0.107965000	1.659999000	-2.757200000
C	-0.726639000	0.740758000	0.507530000
C	3.016193000	0.550763000	-0.906983000
H	3.331209000	-0.483838000	-1.107231000
C	2.162274000	2.223464000	0.675284000
H	1.807543000	2.464120000	1.687248000
C	3.097950000	1.543390000	-1.893375000
H	3.476326000	1.282870000	-2.894424000
C	-1.325518000	3.083381000	0.117445000
H	-1.705601000	4.033544000	0.515801000
C	0.699176000	-1.197557000	1.238817000
C	2.525564000	0.904951000	0.358766000
C	-0.339468000	0.635824000	-0.849544000
H	0.051053000	-0.320073000	-1.230967000
C	-0.907500000	2.970952000	-1.227127000
C	-1.746807000	-1.517354000	1.311504000
H	-2.516179000	-1.258273000	2.076711000
C	1.658247000	-3.563189000	1.001890000
C	-2.449036000	-1.704851000	-0.023813000
C	-2.491437000	-3.087971000	-0.316813000
C	2.700493000	2.872964000	-1.627383000
C	-0.593978000	-0.448414000	1.434118000
H	-0.589117000	-0.071136000	2.478855000
C	-3.074852000	-0.806181000	-0.894795000
H	-3.091327000	0.271063000	-0.682252000
C	2.077031000	-4.342182000	2.211034000
H	3.144062000	-4.085370000	2.400617000
H	2.031526000	-5.438796000	2.041572000
H	1.508650000	-4.068905000	3.119523000
C	-1.278920000	-2.934223000	1.621327000
H	-1.004768000	-3.299348000	2.621798000
C	-3.693264000	-1.314928000	-2.055888000
H	-4.182725000	-0.618469000	-2.752976000
C	-3.702893000	-2.695447000	-2.337237000
H	-4.194942000	-3.062648000	-3.250311000
C	0.792718000	-2.512916000	1.113472000
C	-3.103330000	-3.616648000	-1.459317000
H	-3.124998000	-4.698040000	-1.660493000
C	-1.384198000	5.267627000	-1.705191000
H	-1.300998000	5.931028000	-2.585051000
H	-0.768291000	5.680075000	-0.875648000
H	-2.447242000	5.220368000	-1.382440000
C	2.255420000	3.197038000	-0.324214000
H	1.964909000	4.234227000	-0.094421000
C	2.704948000	3.923787000	-2.707297000
H	3.189510000	4.859449000	-2.360255000
H	1.659347000	4.180491000	-2.983725000
H	3.227371000	3.579232000	-3.620371000
C	2.167716000	-3.991276000	-0.339348000
H	1.847995000	-3.310489000	-1.148981000
H	3.278648000	-4.018308000	-0.301352000
H	1.839037000	-5.027747000	-0.577496000

TS2"

E_e^S = -1761.678627

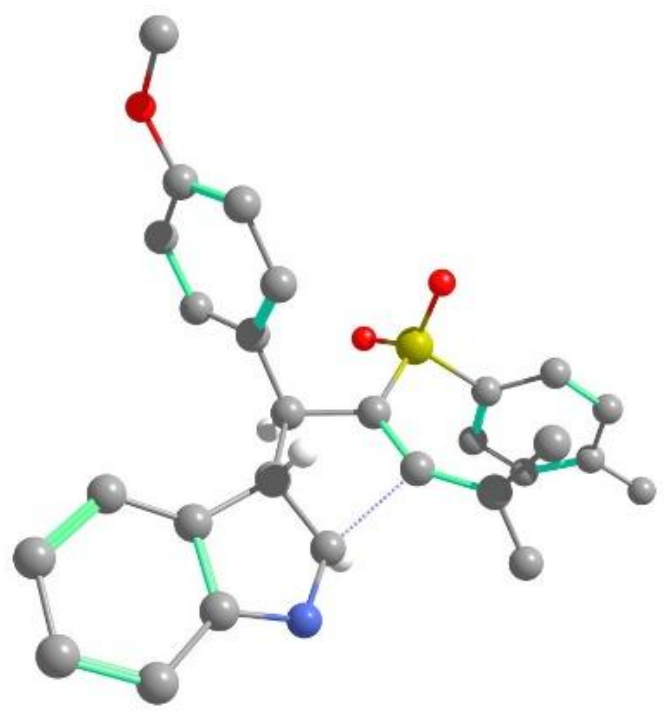

S	0.255418000	-0.570789000	-1.337213000
O	1.211149000	-1.673783000	-1.061972000
O	0.240031000	0.175211000	-2.616668000
O	6.516249000	0.625023000	-2.562140000
N	0.172094000	3.332940000	2.718408000
H	-0.638468000	3.620389000	3.273792000
C	3.558227000	0.545043000	-0.337414000
H	3.183466000	0.009049000	0.549633000
C	4.504331000	1.833648000	-2.638596000
H	4.895175000	2.318222000	-3.545204000
C	2.756672000	1.515872000	-0.967096000
C	-1.571889000	-2.421379000	-0.383052000
H	-0.688464000	-3.046995000	-0.186945000
C	-2.505795000	-0.339095000	-1.280271000
H	-2.349471000	0.630242000	-1.777687000
C	-2.869594000	-2.857864000	-0.073937000
H	-3.012279000	-3.851376000	0.380314000
C	4.827742000	0.216499000	-0.833764000
H	5.423372000	-0.552273000	-0.323924000
C	0.540547000	0.690121000	-0.043160000
C	-1.407529000	-1.158830000	-0.970434000
C	3.242125000	2.145941000	-2.132250000
H	2.616973000	2.888330000	-2.652874000
C	5.312269000	0.864168000	-1.993763000
C	1.460568000	2.544914000	0.986036000
H	1.974180000	1.750754000	1.593457000
C	-0.796525000	-0.216077000	1.974988000
C	2.132321000	3.764733000	1.559210000
C	1.379918000	4.080283000	2.728078000
C	-3.998548000	-2.052717000	-0.350329000
C	1.402462000	1.893641000	-0.393735000
H	0.856692000	2.518468000	-1.135151000
C	3.350069000	4.398165000	1.316916000
H	3.950471000	4.139544000	0.432063000
C	-0.141552000	-1.450226000	2.519844000
H	-0.884567000	-2.266736000	2.627371000
H	0.252791000	-1.244466000	3.542192000
H	0.699791000	-1.780099000	1.881262000
C	0.066743000	2.569554000	1.582865000
H	-0.850059000	2.732479000	0.987340000
C	3.776373000	5.389050000	2.228631000
H	4.713872000	5.932485000	2.037620000
C	3.023671000	5.692848000	3.380606000
H	3.385762000	6.463558000	4.077516000
C	-0.082865000	0.614735000	1.149150000
C	1.813127000	5.029454000	3.661152000
H	1.231200000	5.264005000	4.564964000
C	7.365032000	-0.362489000	-1.985126000
H	8.274809000	-0.391514000	-2.611939000
H	6.884825000	-1.365850000	-1.991035000
H	7.648762000	-0.098009000	-0.942179000
C	-3.792616000	-0.789355000	-0.958919000
H	-4.662963000	-0.158101000	-1.199431000
C	-5.392666000	-2.543858000	-0.053944000
H	-5.399511000	-3.307551000	0.748117000
H	-5.834106000	-3.014849000	-0.959337000
H	-6.067195000	-1.714956000	0.239504000
C	-2.192082000	0.104379000	2.401962000
H	-2.509920000	1.128283000	2.130947000
H	-2.869155000	-0.605549000	1.869934000
H	-2.342724000	-0.070387000	3.488426000

5. NMR Spectra for all compounds

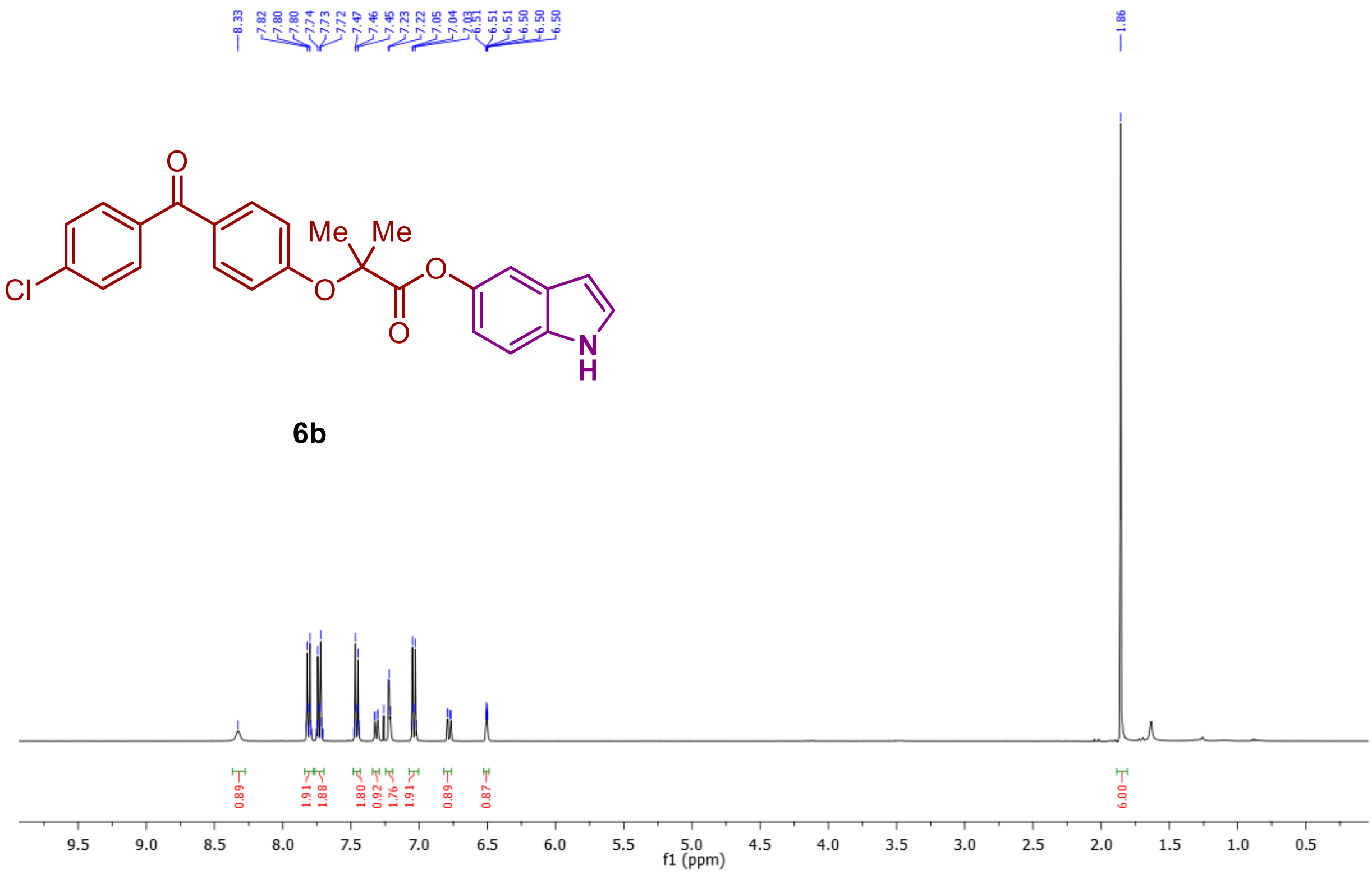

Figure S4: ^{1}H-NMR of ***6b*** in $CDCl_3$ (400 MHz).

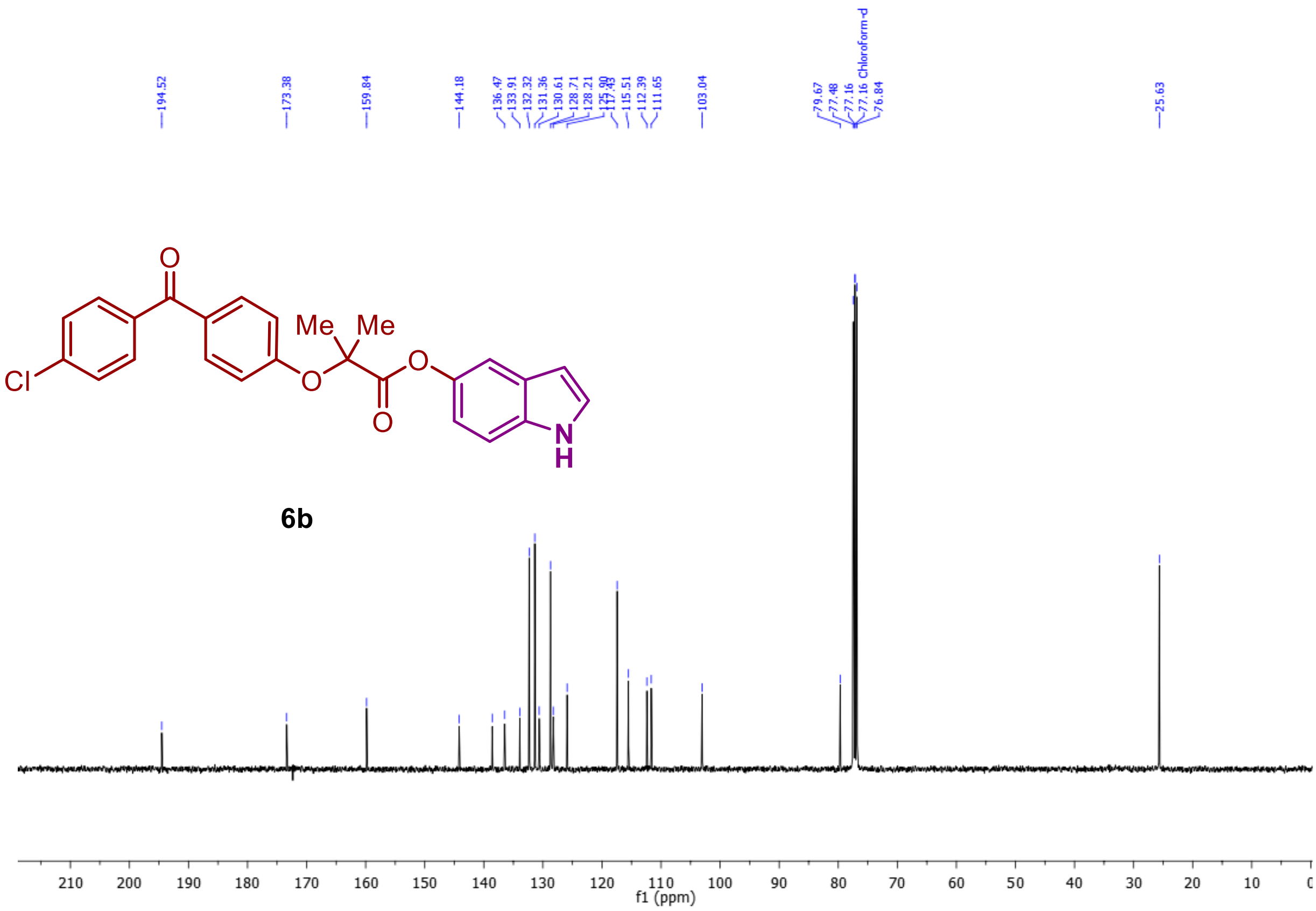

Figure S5: ^{13}C-NMR of ***6b*** in $CDCl_3$ (101 MHz)

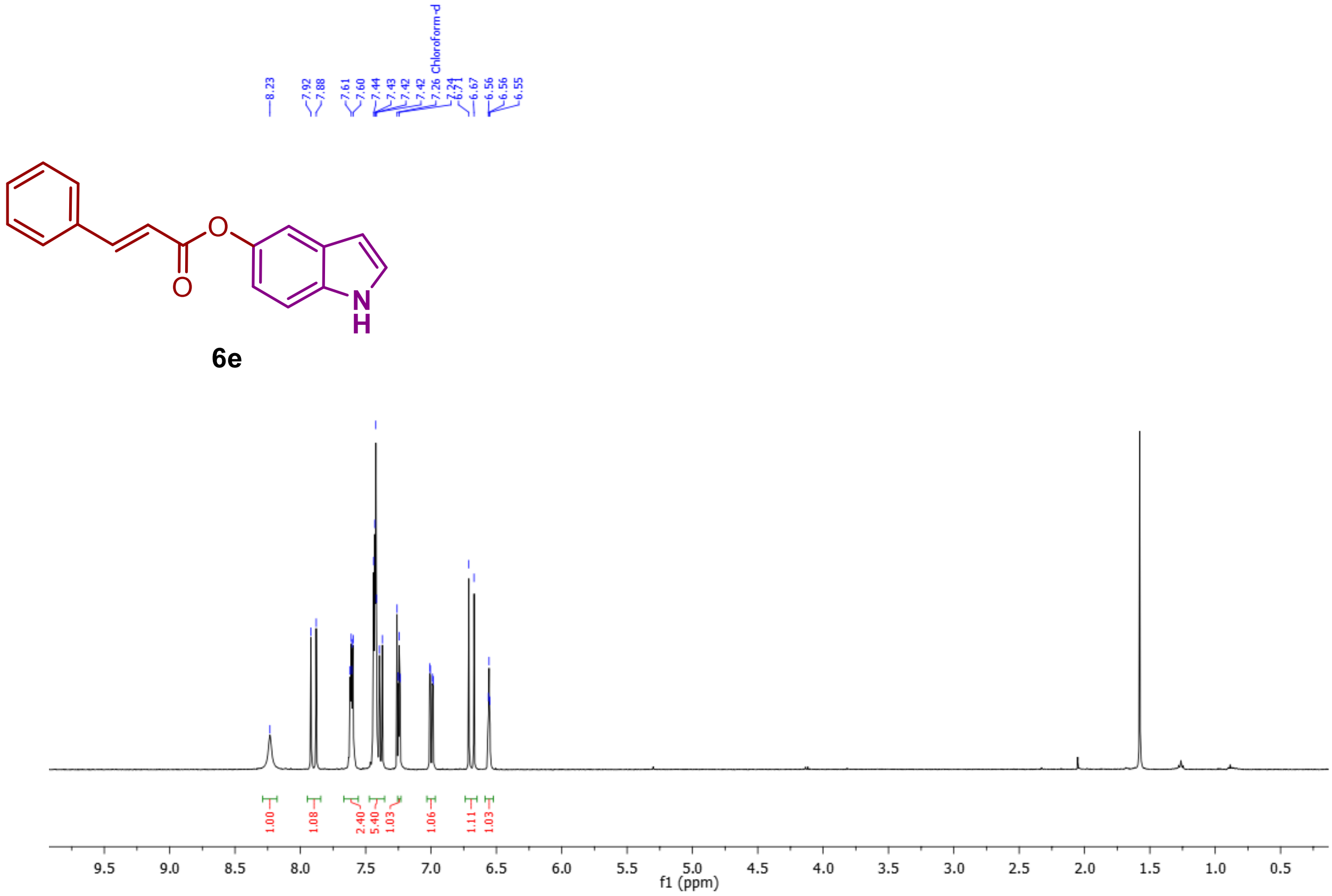

Figure S6: *^{13}C-NMR of **6e** in $CDCl_3$ (101 MHz)*

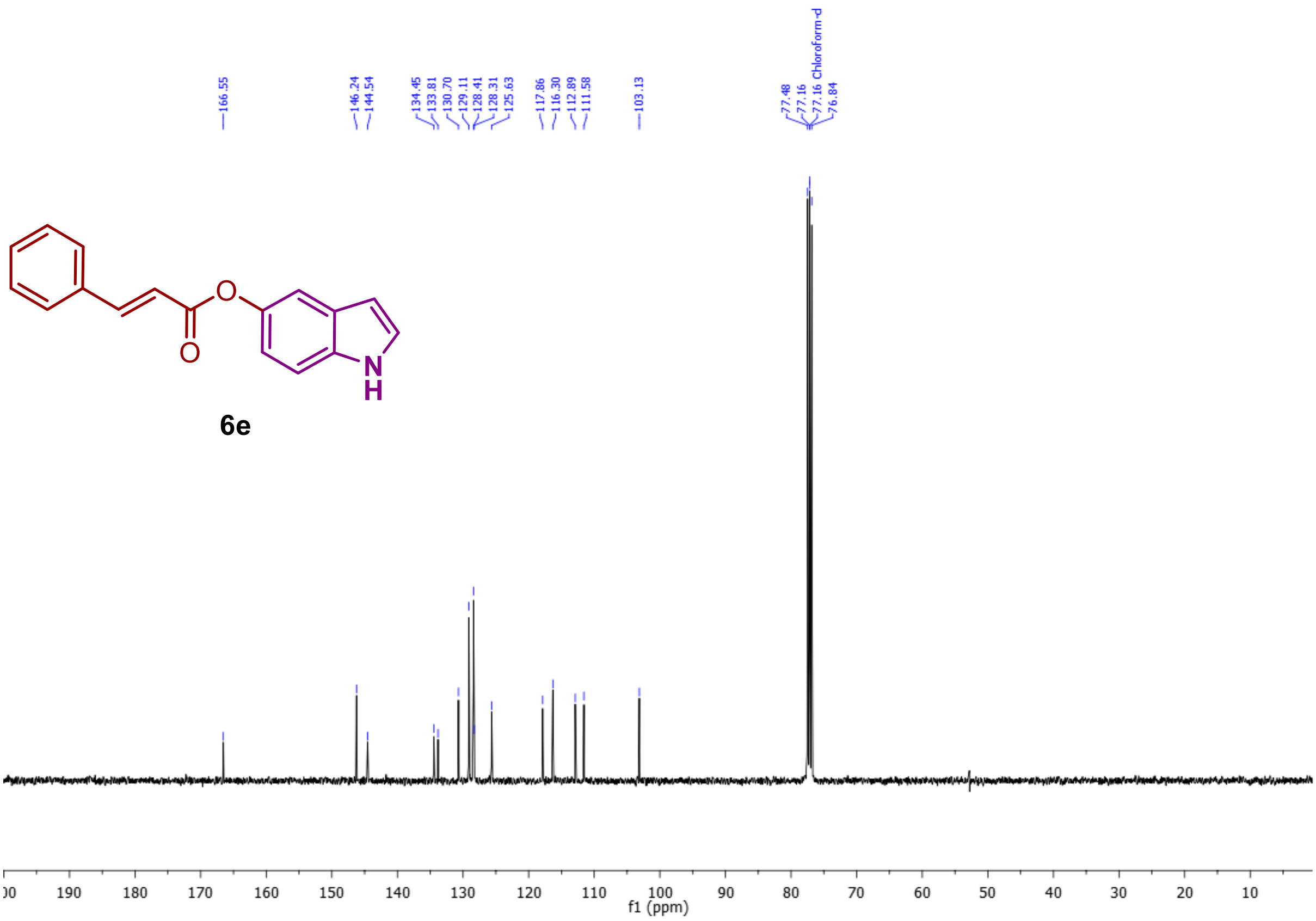

***Figure S7**: ^{13}C-NMR of **6e** in $CDCl_3$ (101 MHz)*

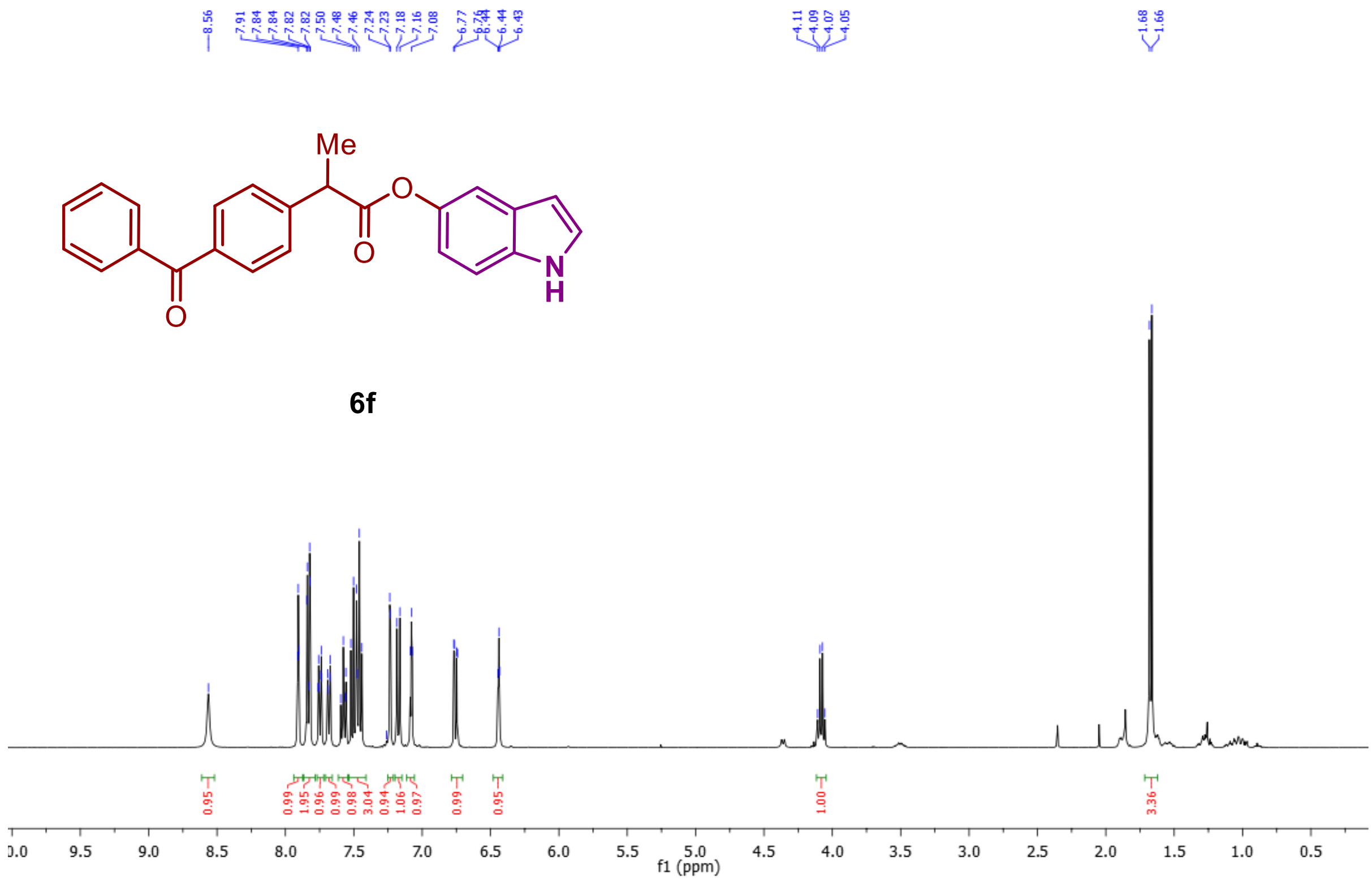

Figure S8: *^{1}H-NMR of **6f** in $CDCl_3$ (400 MHz)*

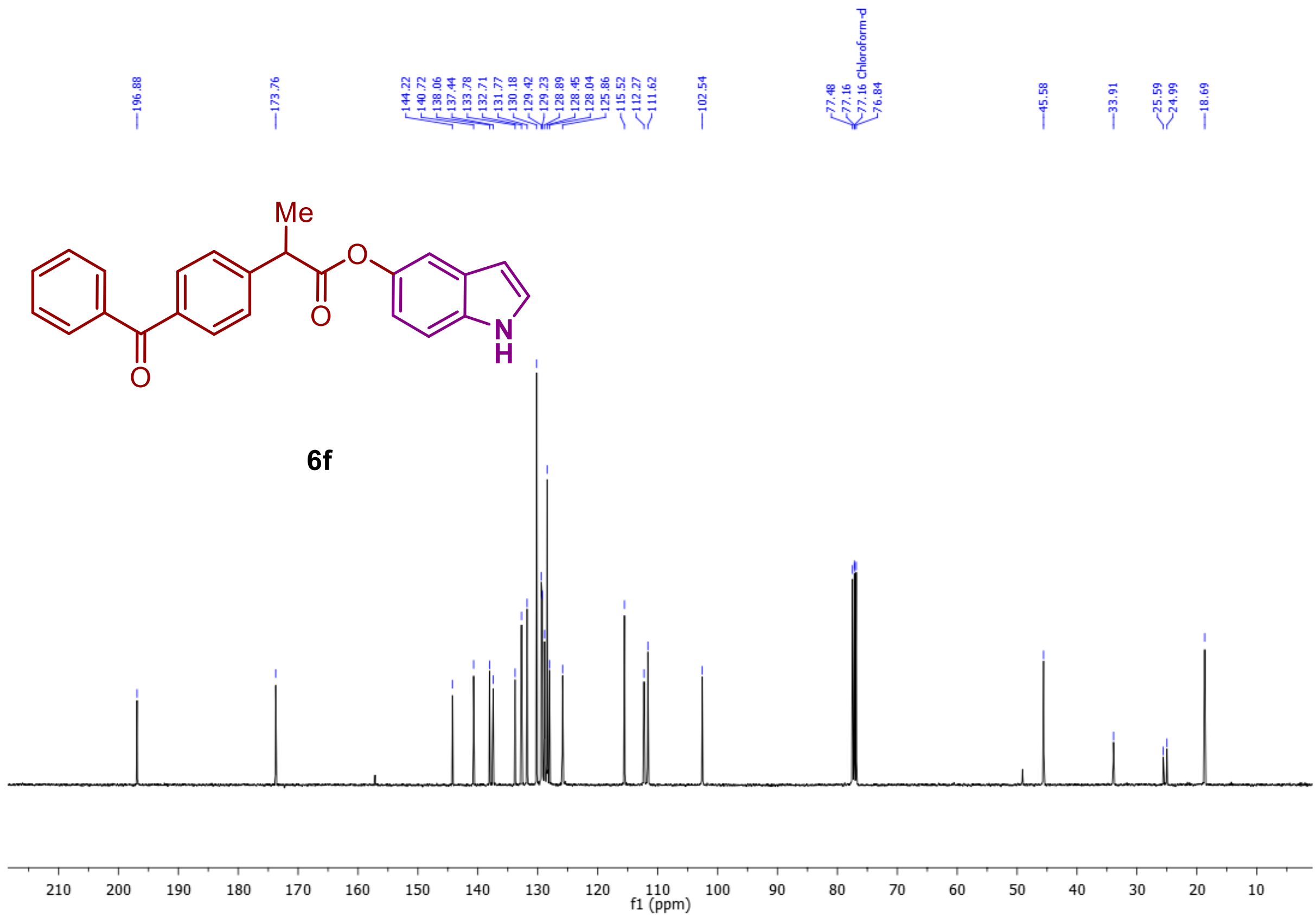

Figure S9: *^{13}C-NMR of **6f** in $CDCl_3$ (101 MHz)*

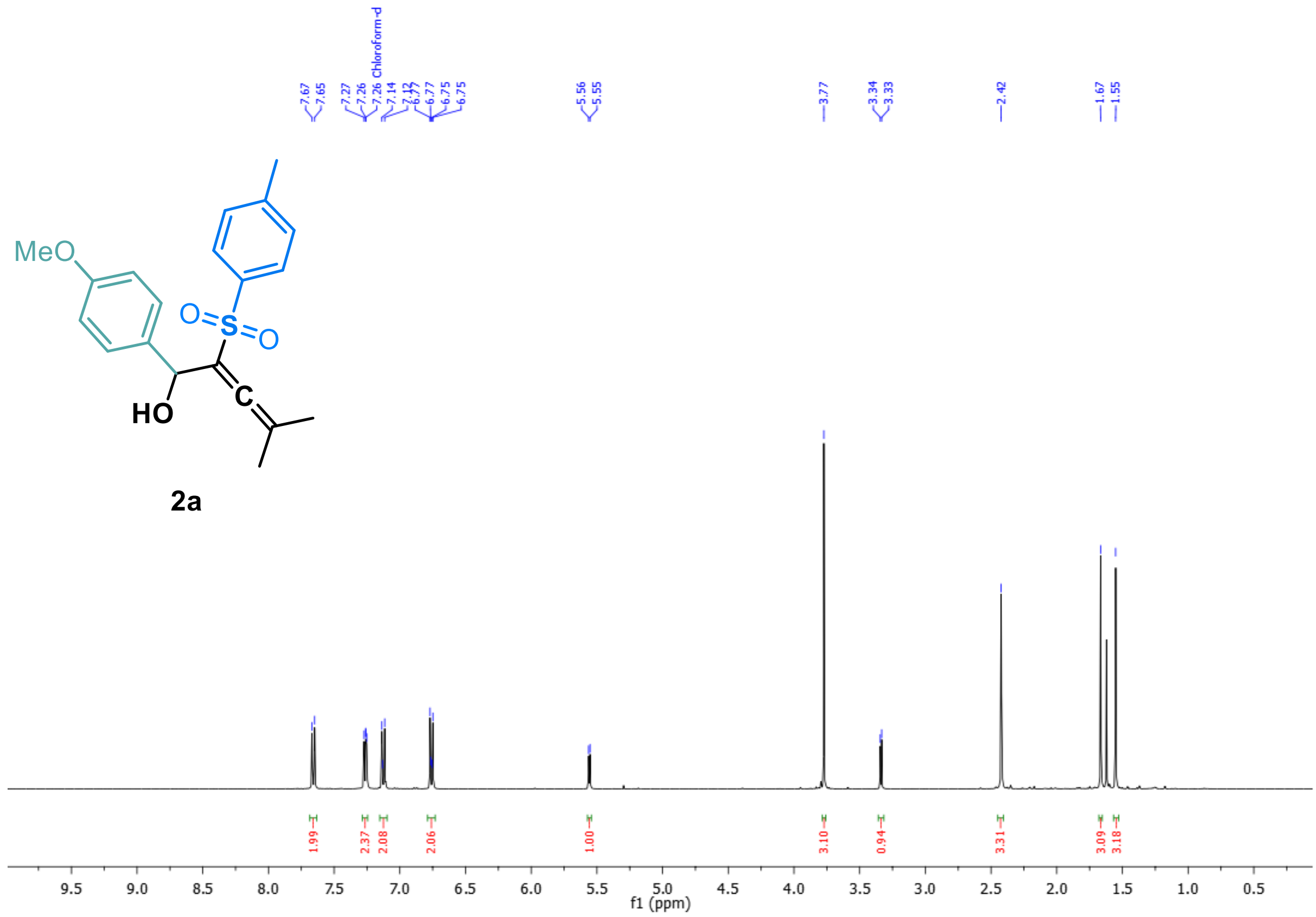

Figure S10: ^{1}H-NMR of **2a** in $CDCl_3$ (400 MHz).

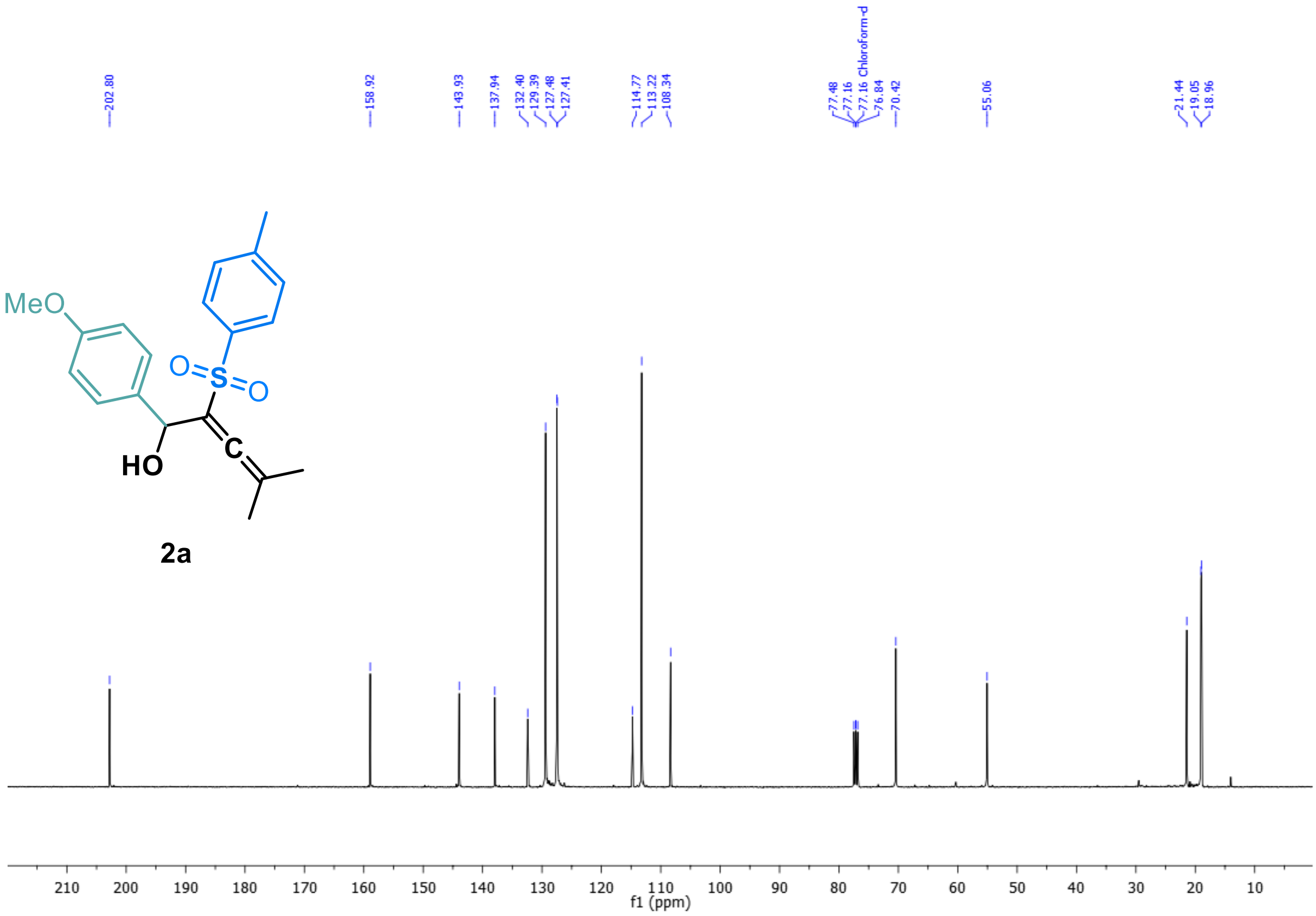

Figure S11: ^{13}C-NMR of 2***a*** in $CDCl_3$ (101 MHz)

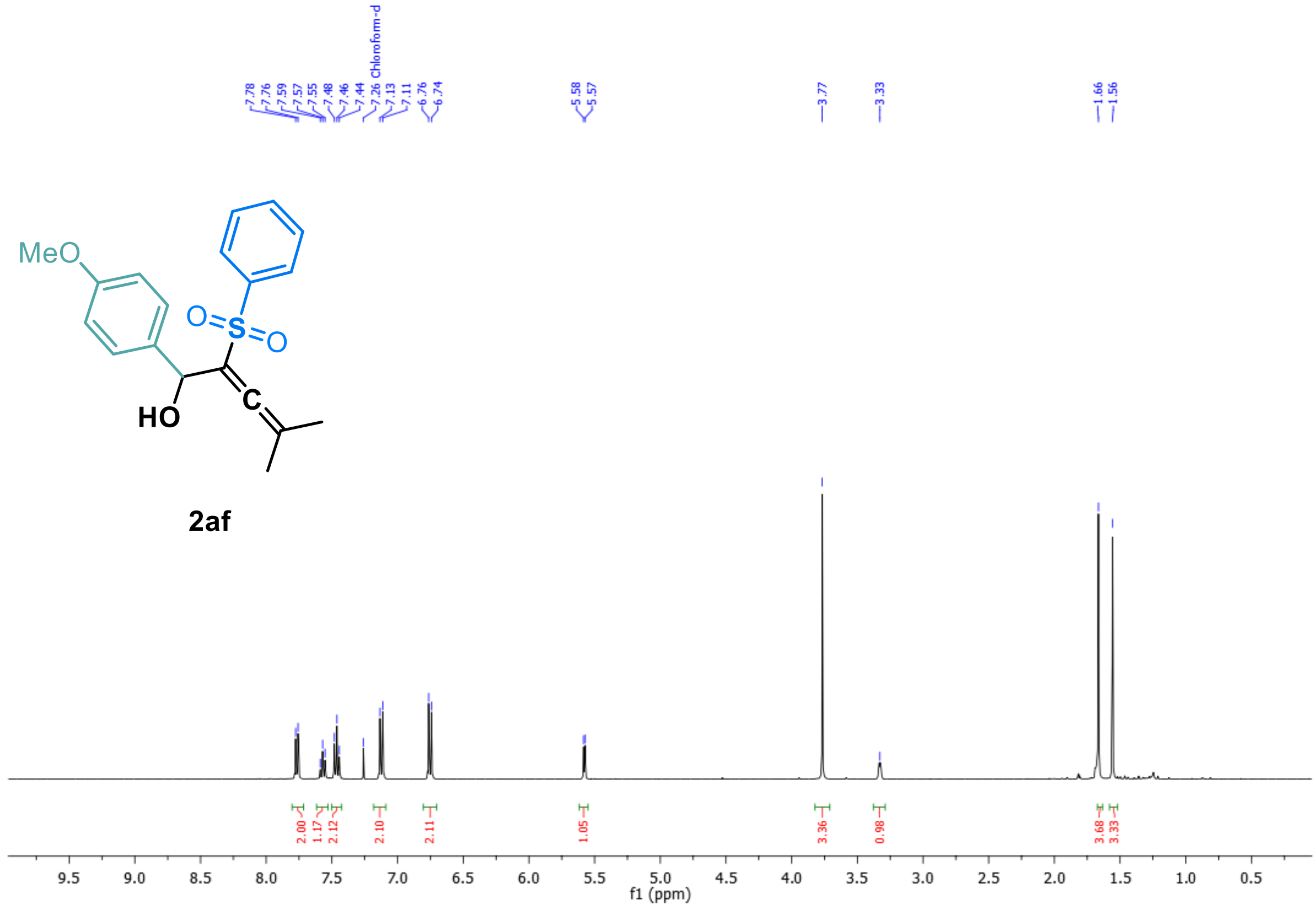

***Figure S12**: ^{1}H-NMR of **2af** in $CDCl_3$ (400 MHz)*

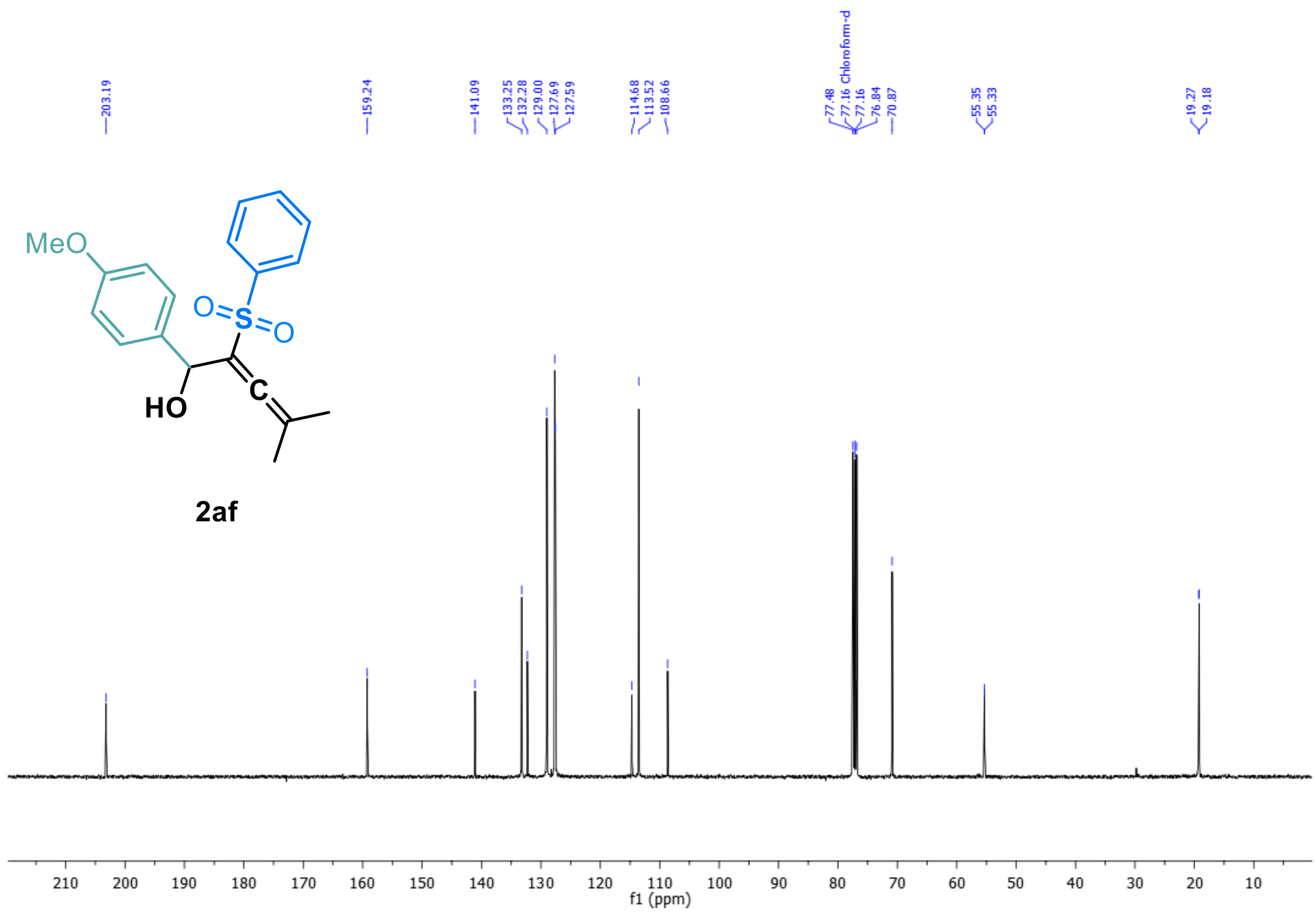

***Figure S13**: ^{13}C-NMR of 2**af** in $CDCl_3$ (101 MHz)*

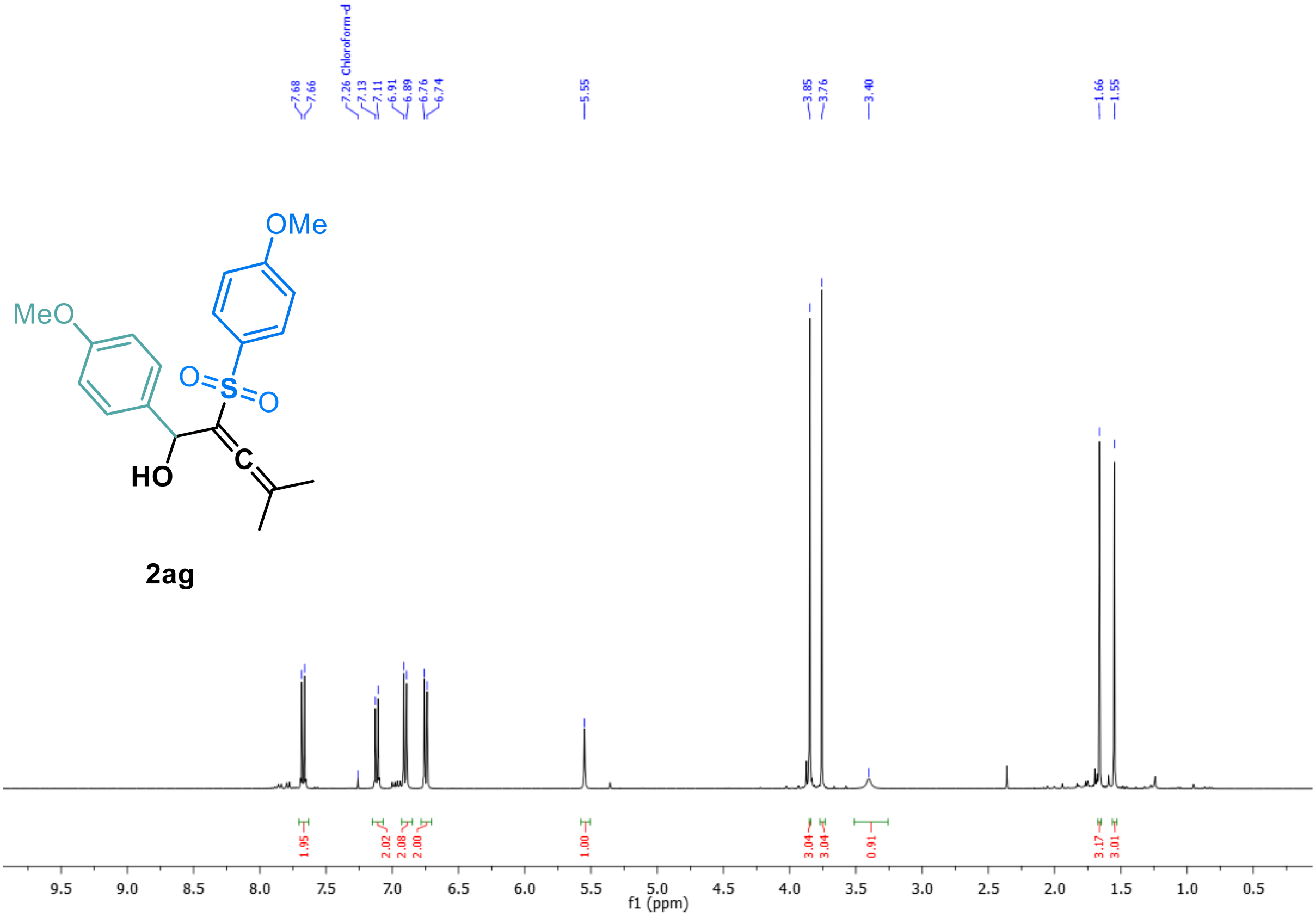

Figure S14: ^{1}H-NMR of **2ag** in $CDCl_3$ (400 MHz)

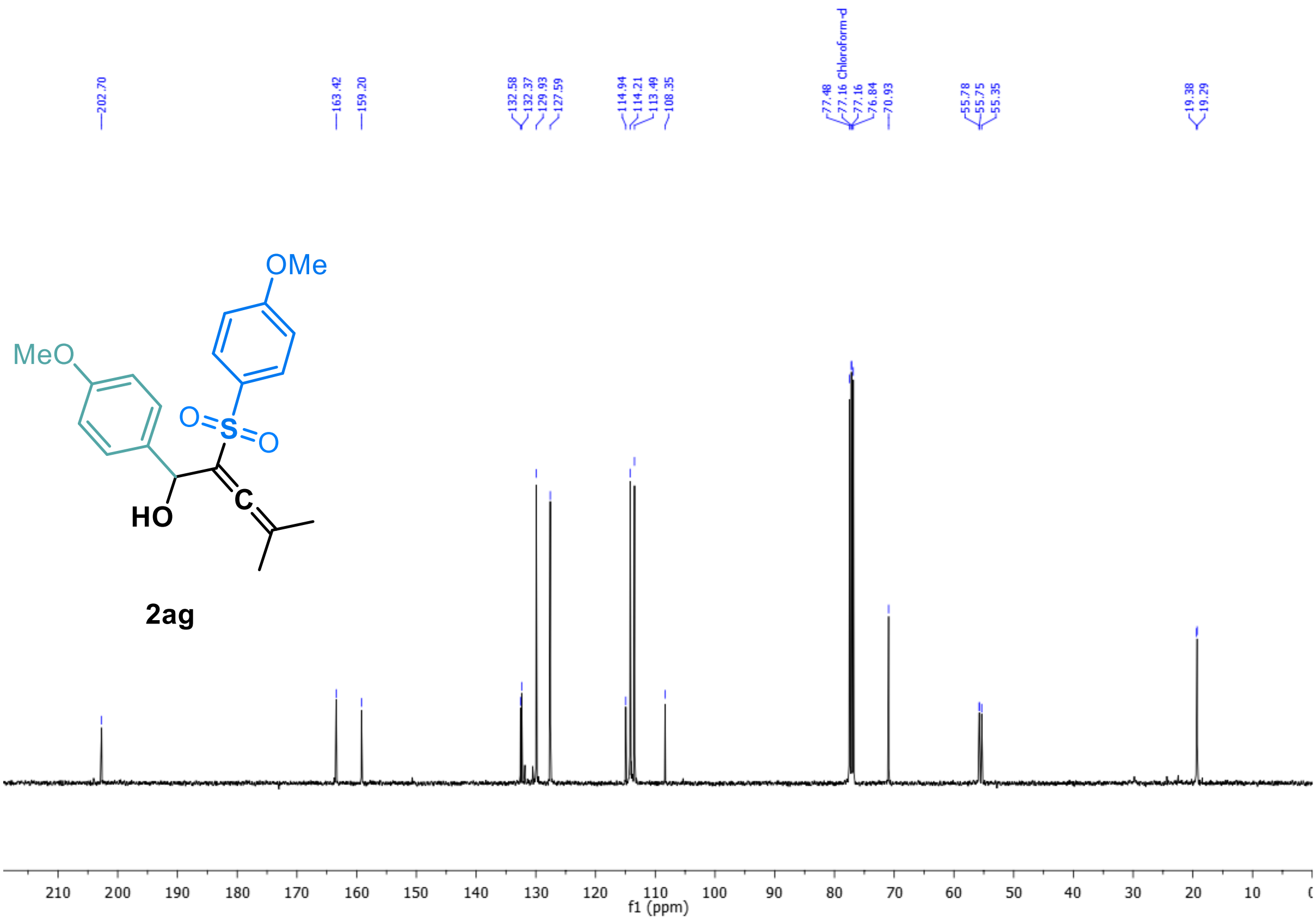

Figure S15: ^{13}C-NMR of 2**ag** in $CDCl_3$ (101 MHz)

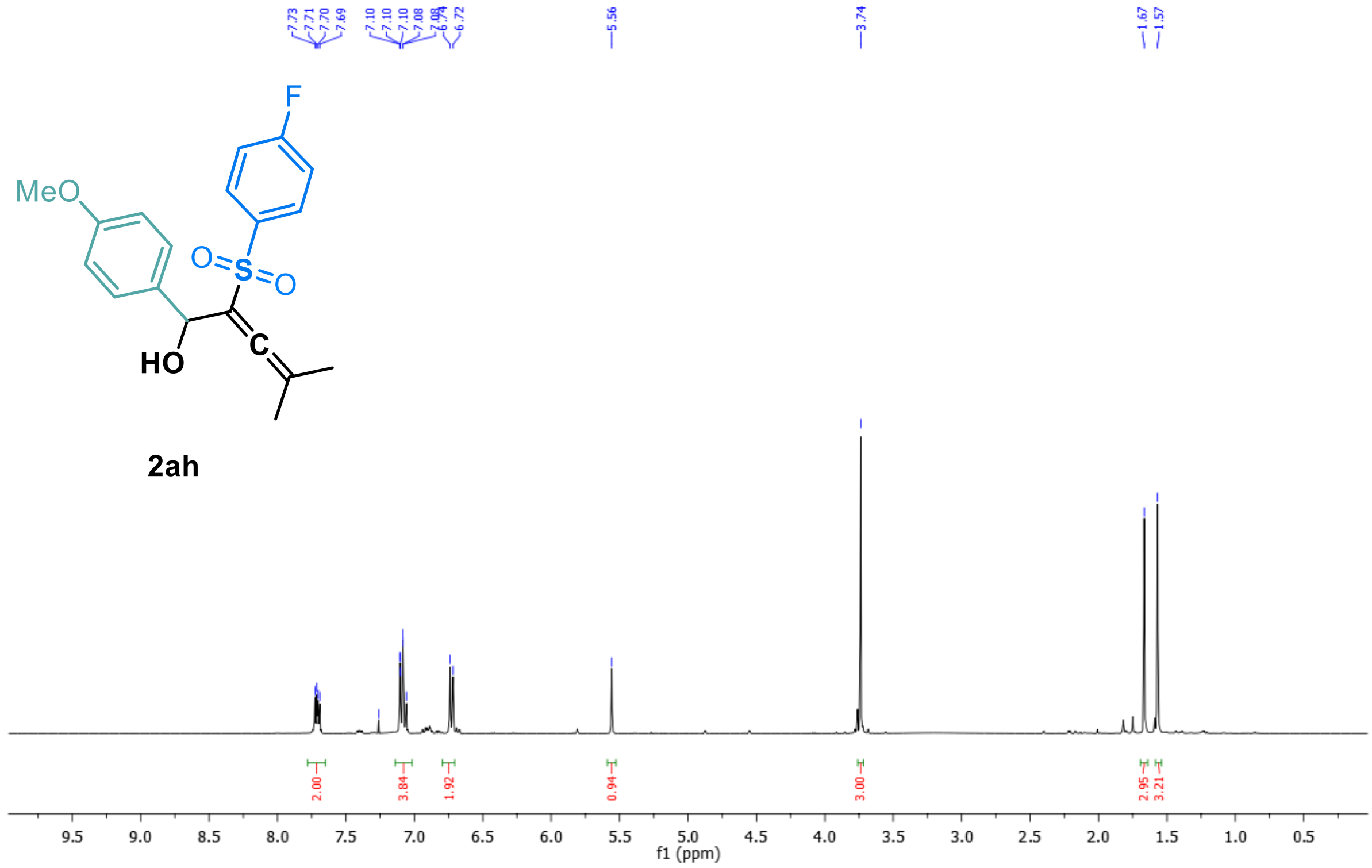

Figure S16: *^{1}H-NMR of* ***2ah*** *in $CDCl_3$ (400 MHz)*

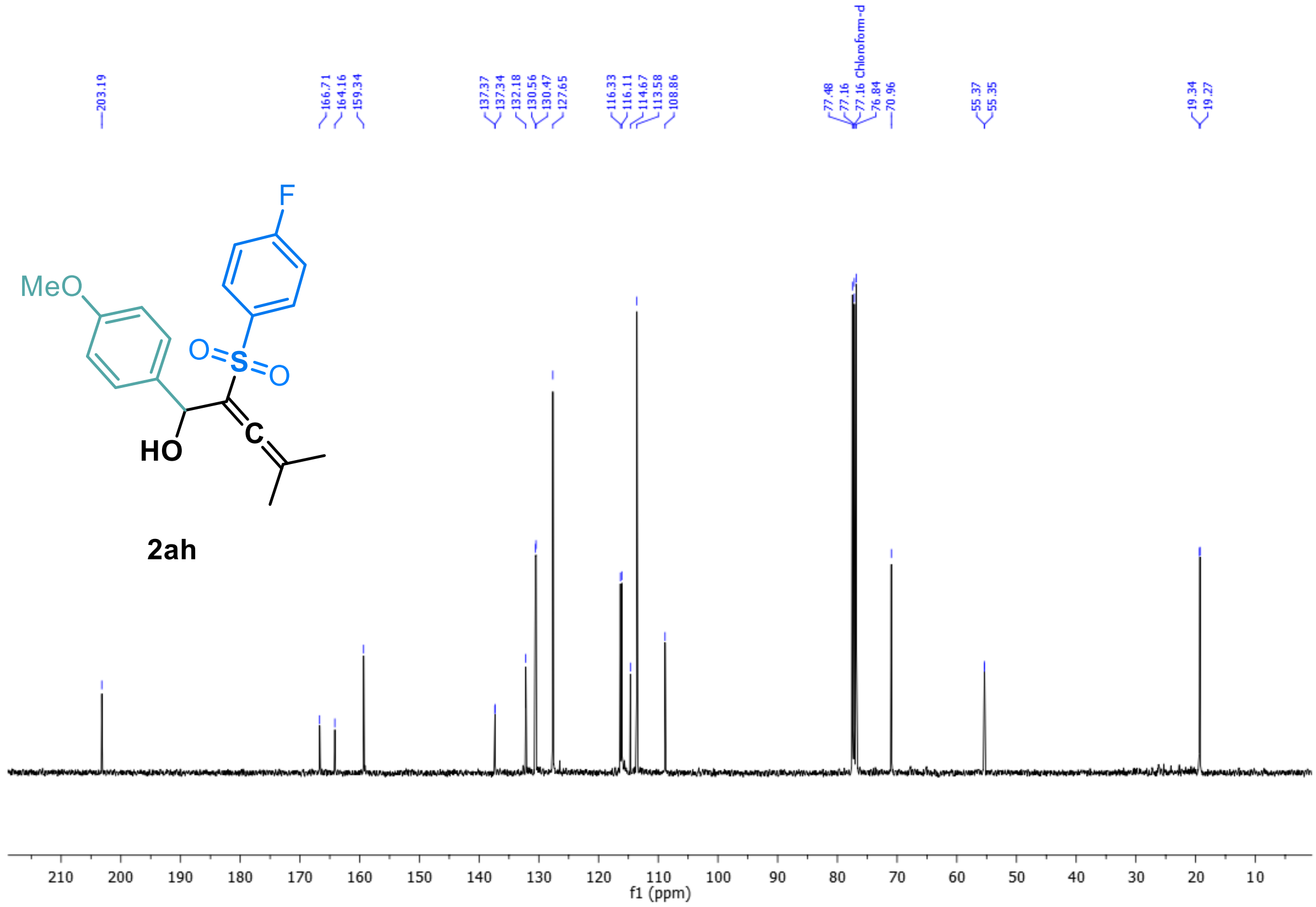

Figure S17: *^{13}C-NMR of 2**ah** in $CDCl_3$ (101 MHz)*

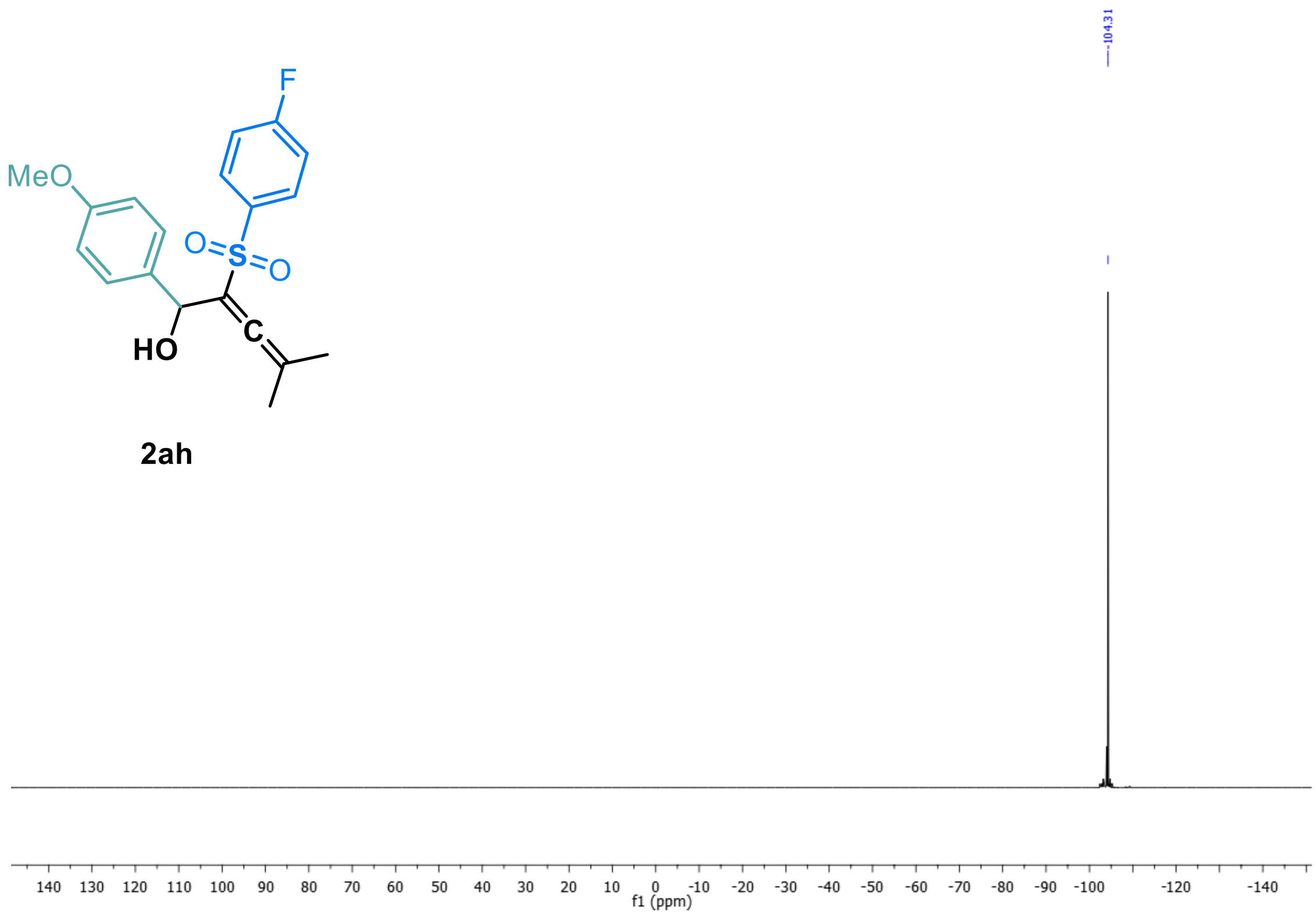

Figure S18: ^{19}F-NMR of **2ah** in $CDCl_3$ (346 MHz)

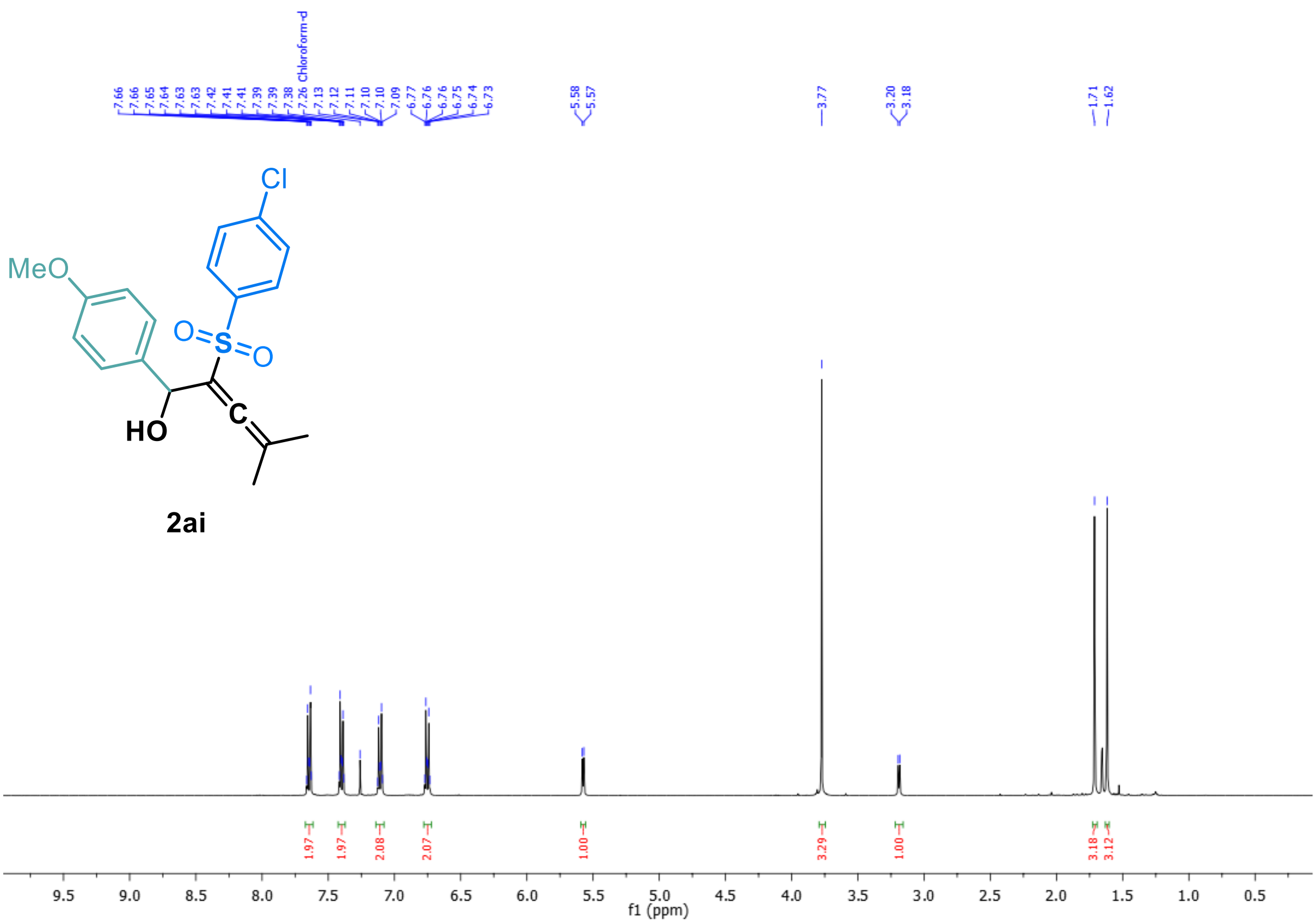

Figure S19: ^{1}H-NMR of ***2ai*** in $CDCl_3$ (400 MHz)

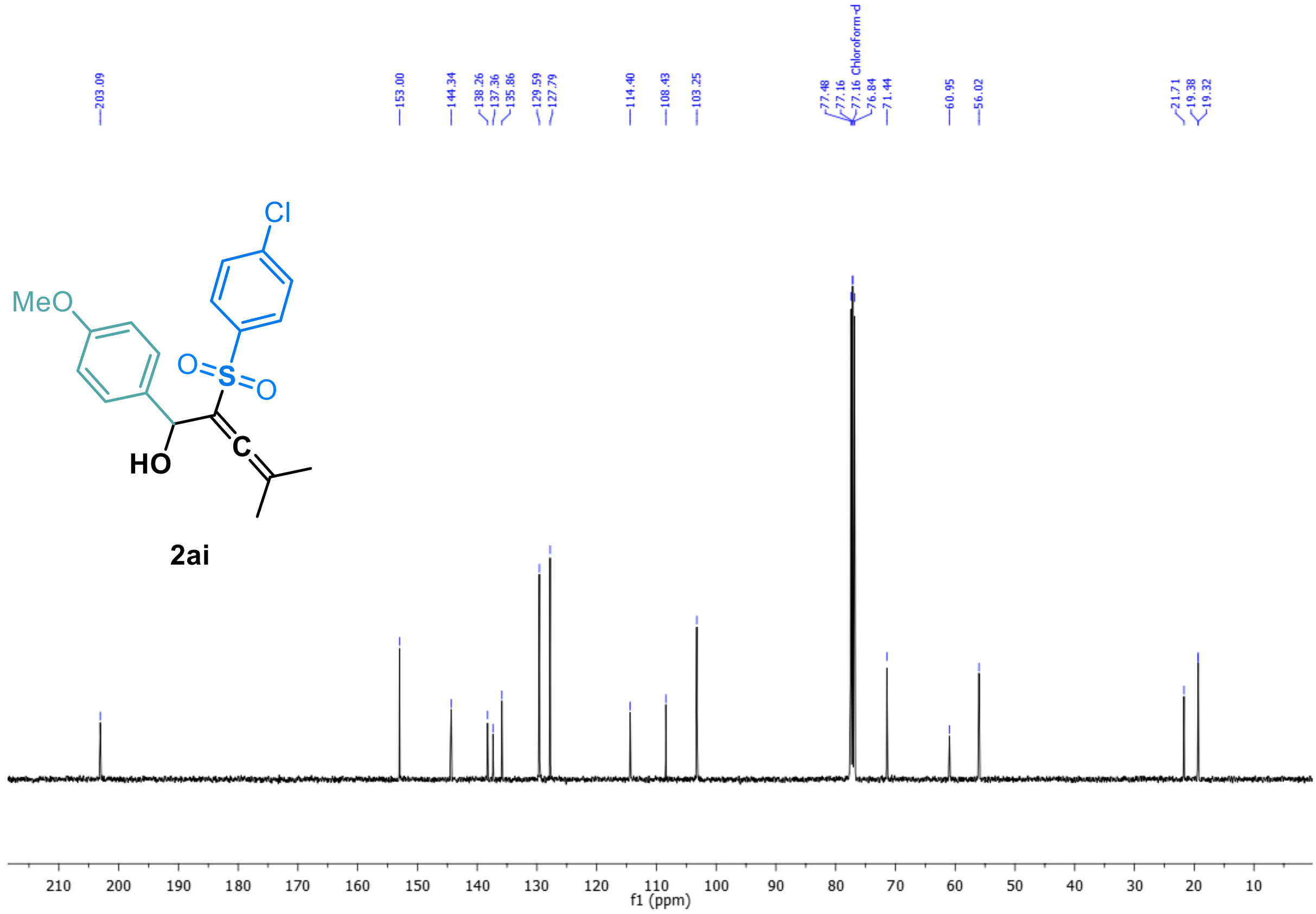

Figure S20: ^{13}C-NMR of 2***ai*** in $CDCl_3$ (101 MHz)

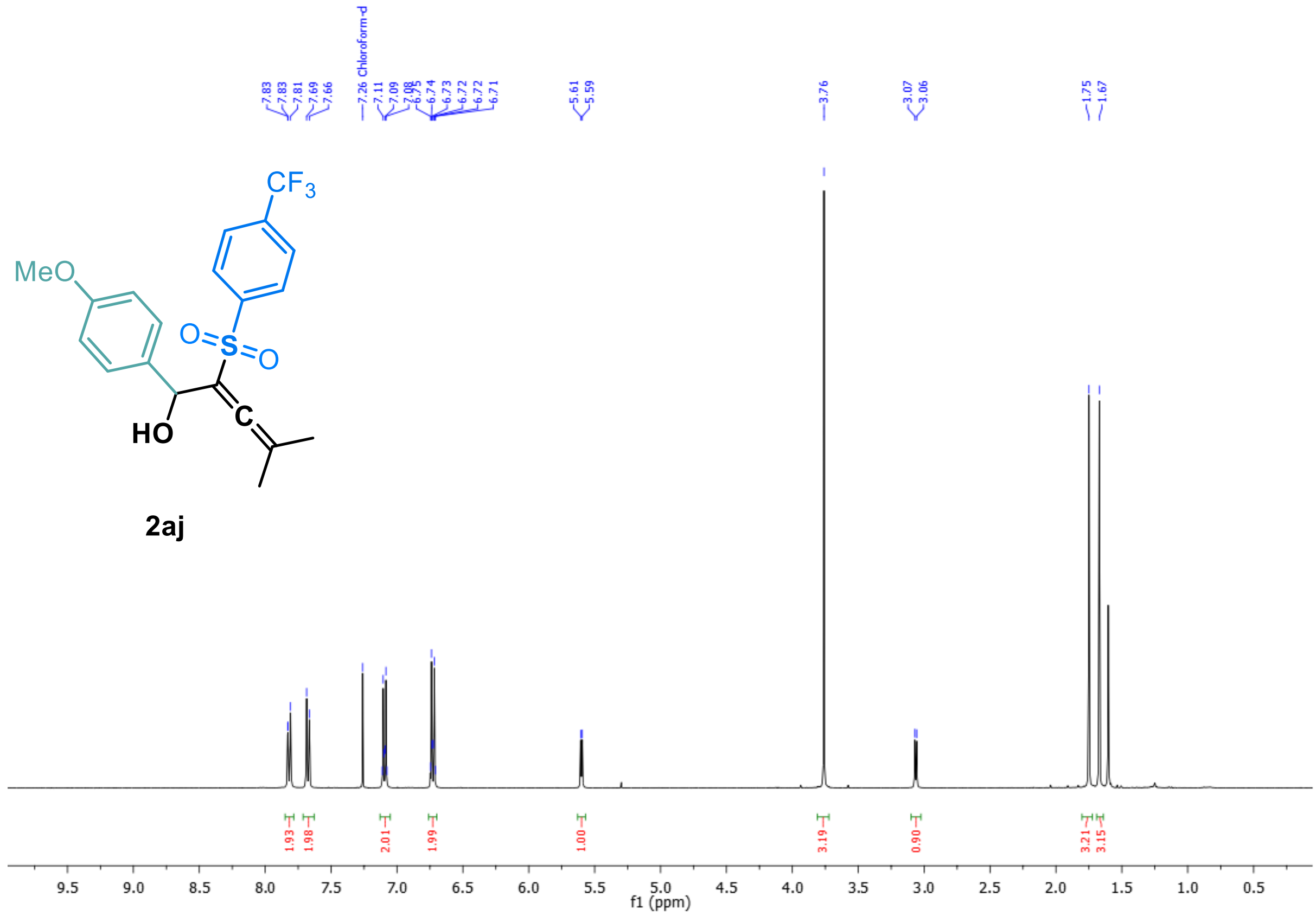

Figure S21: ^{1}H-NMR of ***2aj*** in $CDCl_3$ (400 MHz)

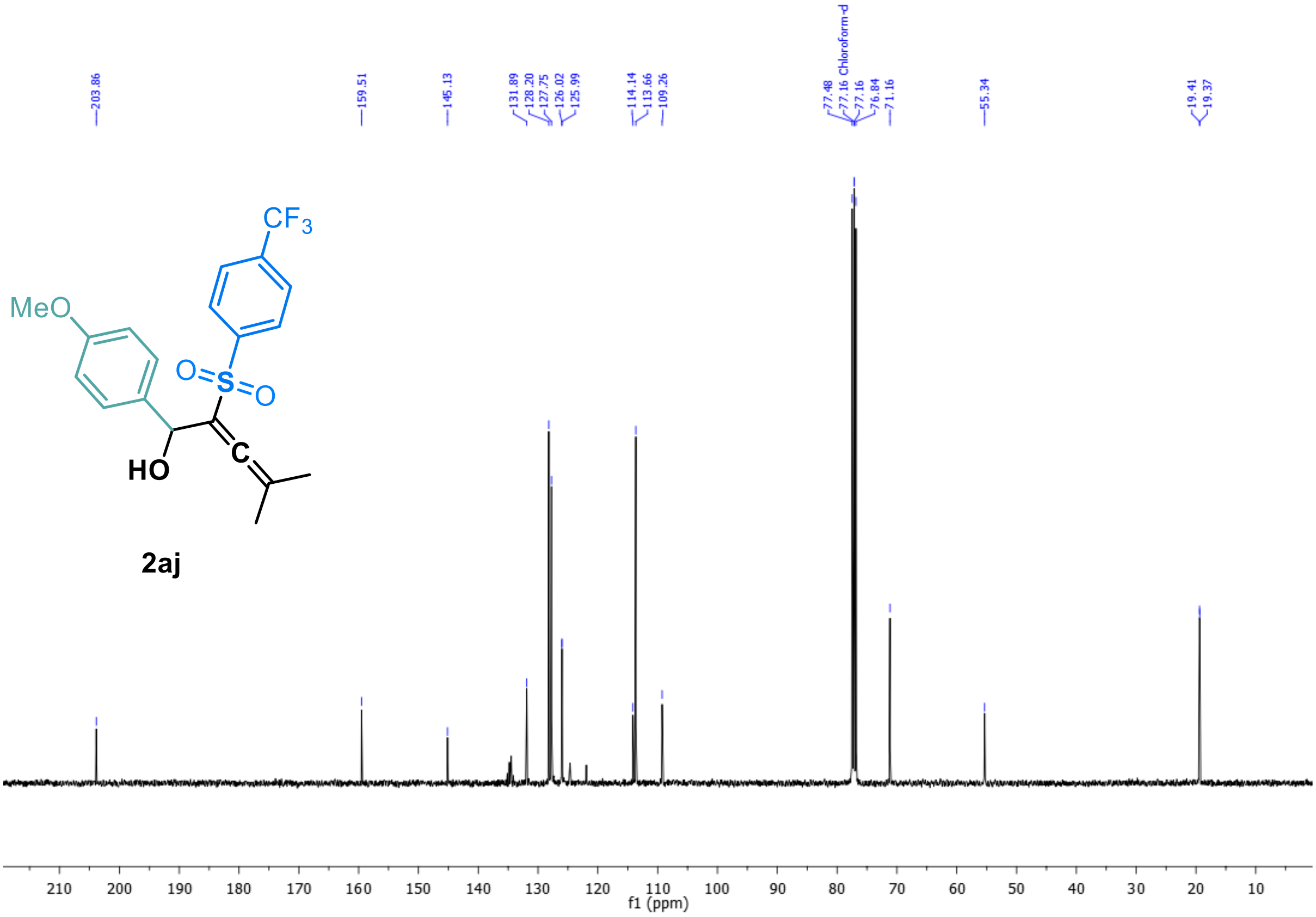

Figure S22: ^{13}C-NMR of 2***aj*** in $CDCl_3$ (101 MHz)

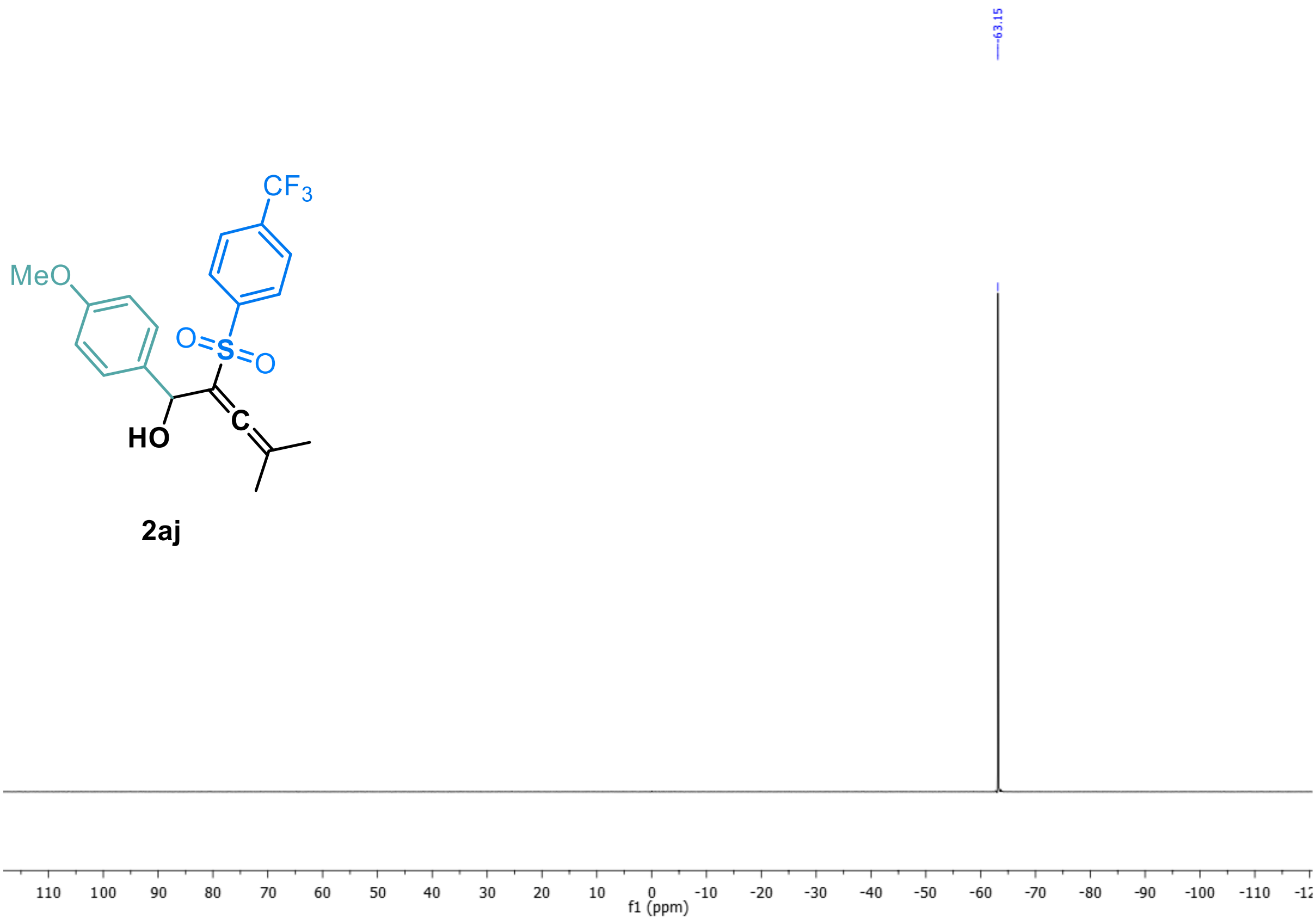

Figure S23: ^{19}F-NMR of **2*aj*** in $CDCl_3$ (376 MHz)

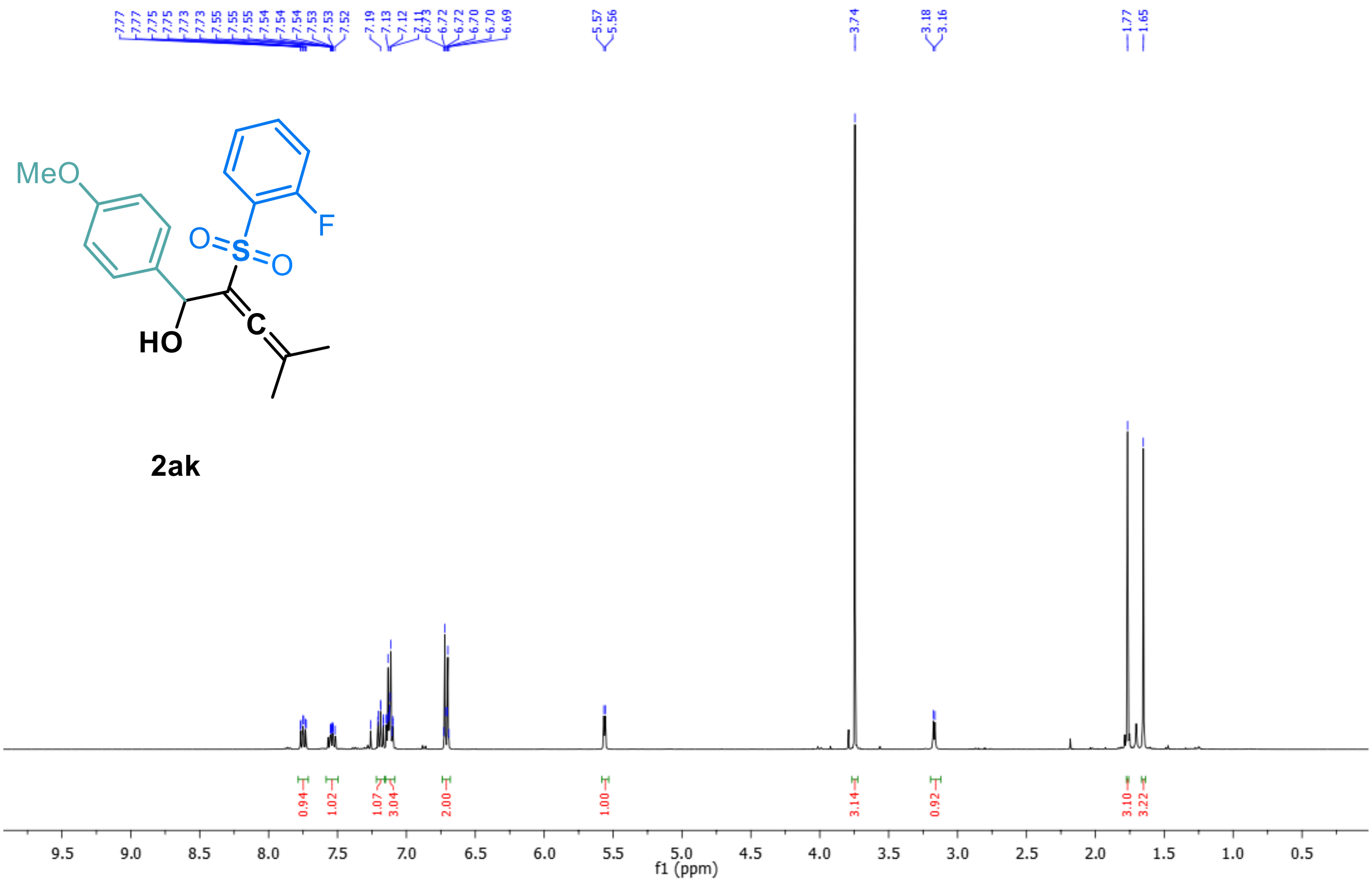

2ak

Figure S24: ^{1}H-NMR of ***2ak*** in $CDCl_3$ (400 MHz)

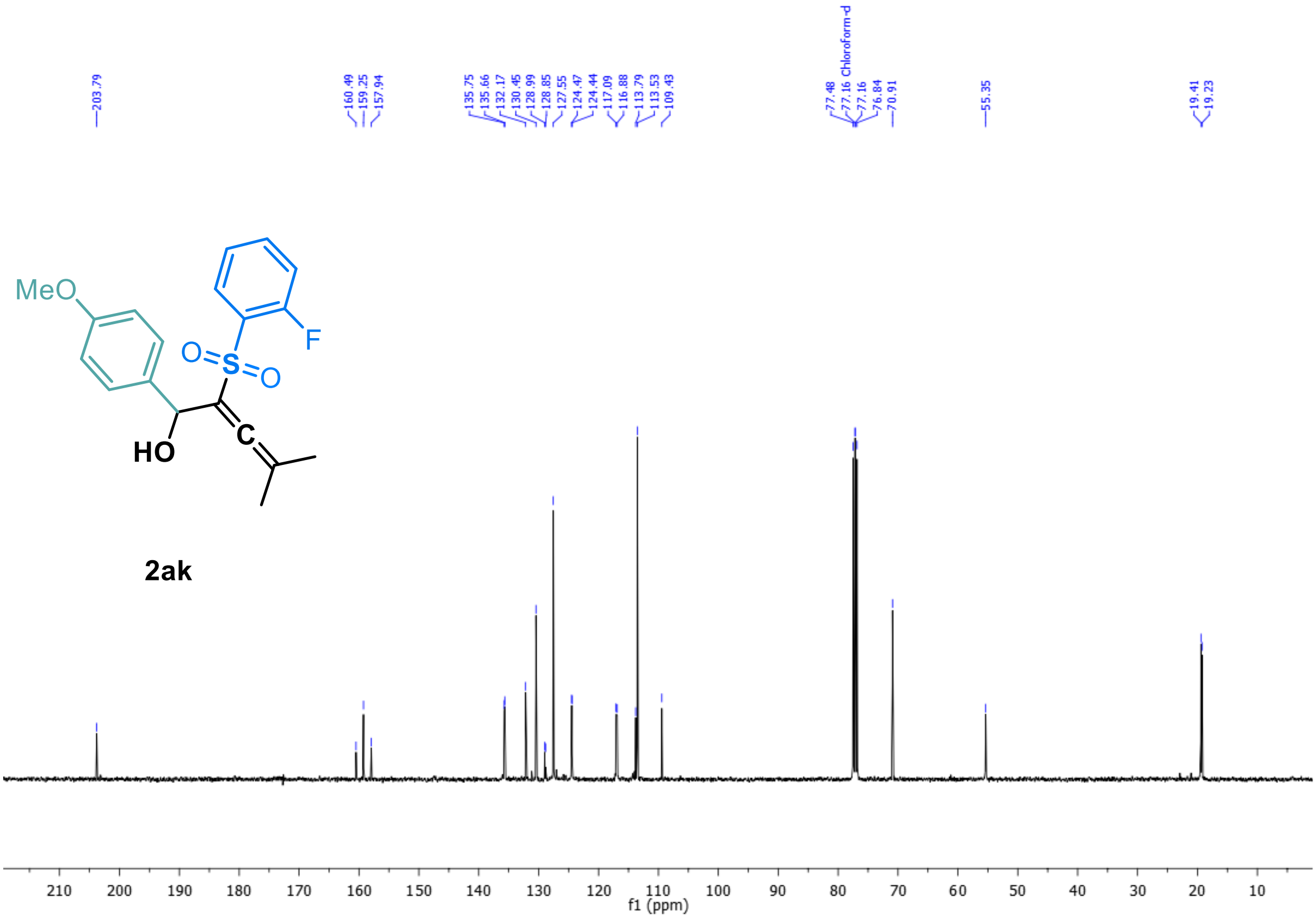

2ak

Figure S25: ^{13}C-NMR of ***2ak*** in $CDCl_3$ (101 MHz)

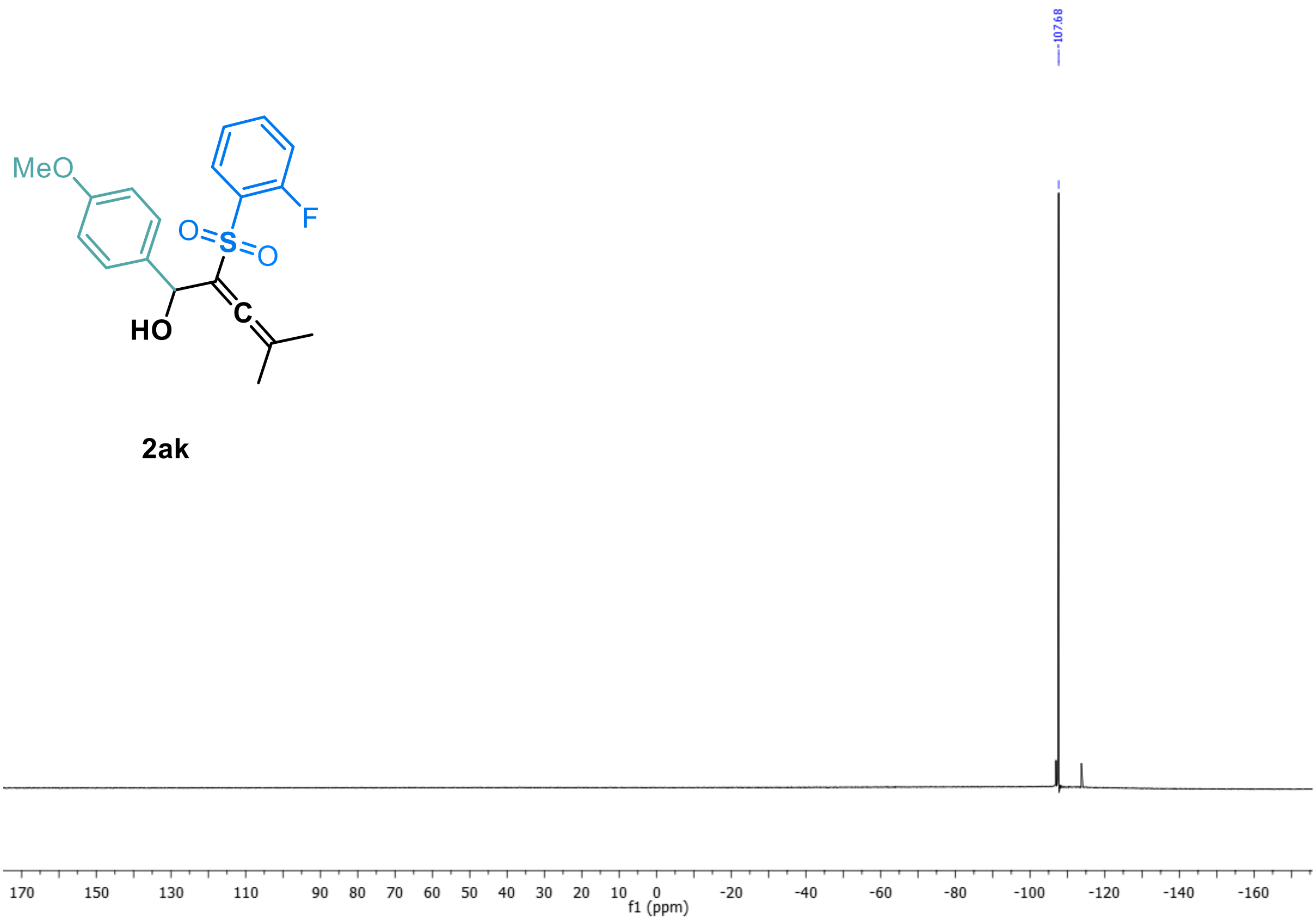

Figure S26: *^{19}F-NMR of **2ak** in CDCl$_3$ (376 MHz)*

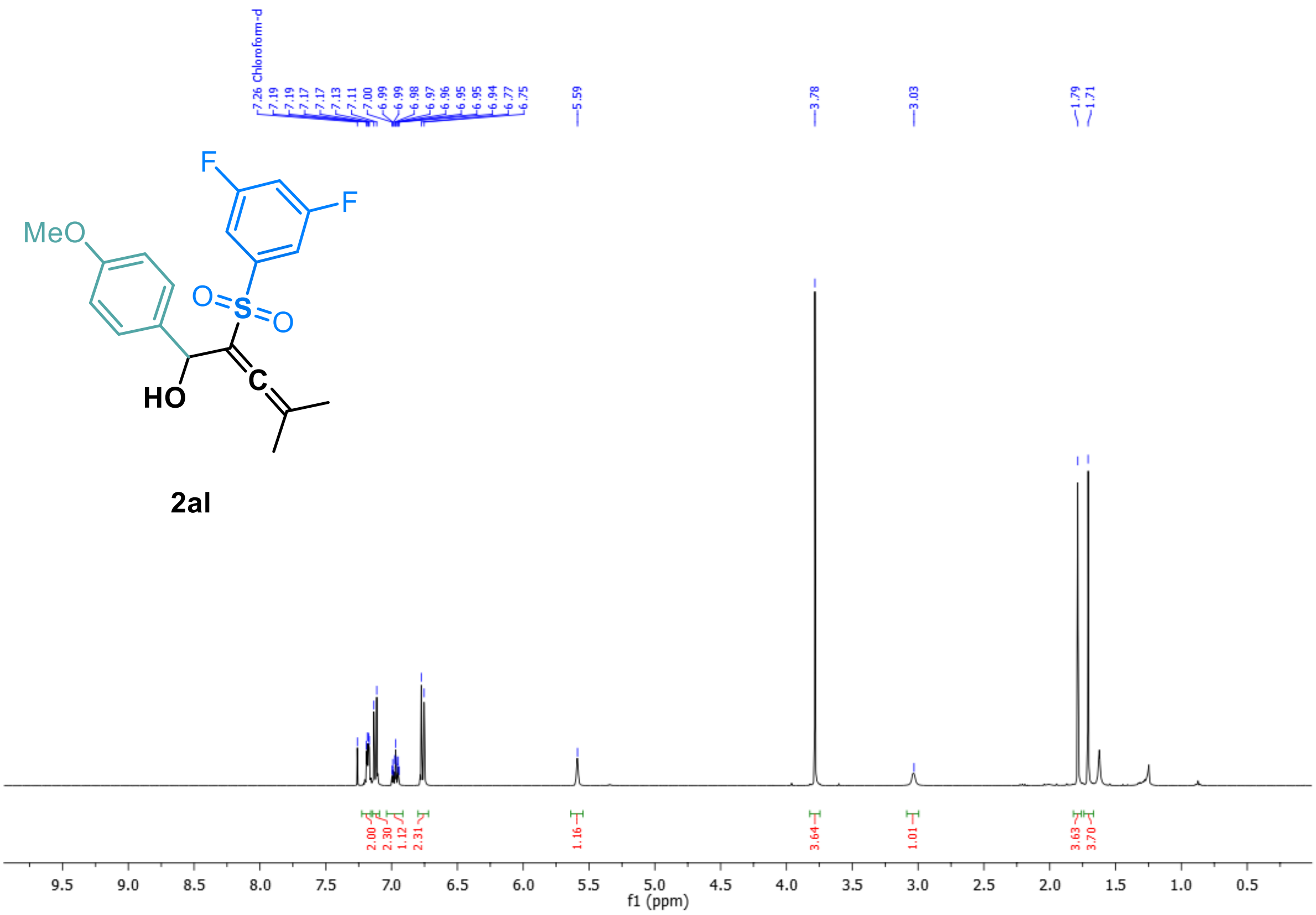

Figure S27: *^{1}H-NMR of **2al** in CDCl$_3$ (400 MHz)*

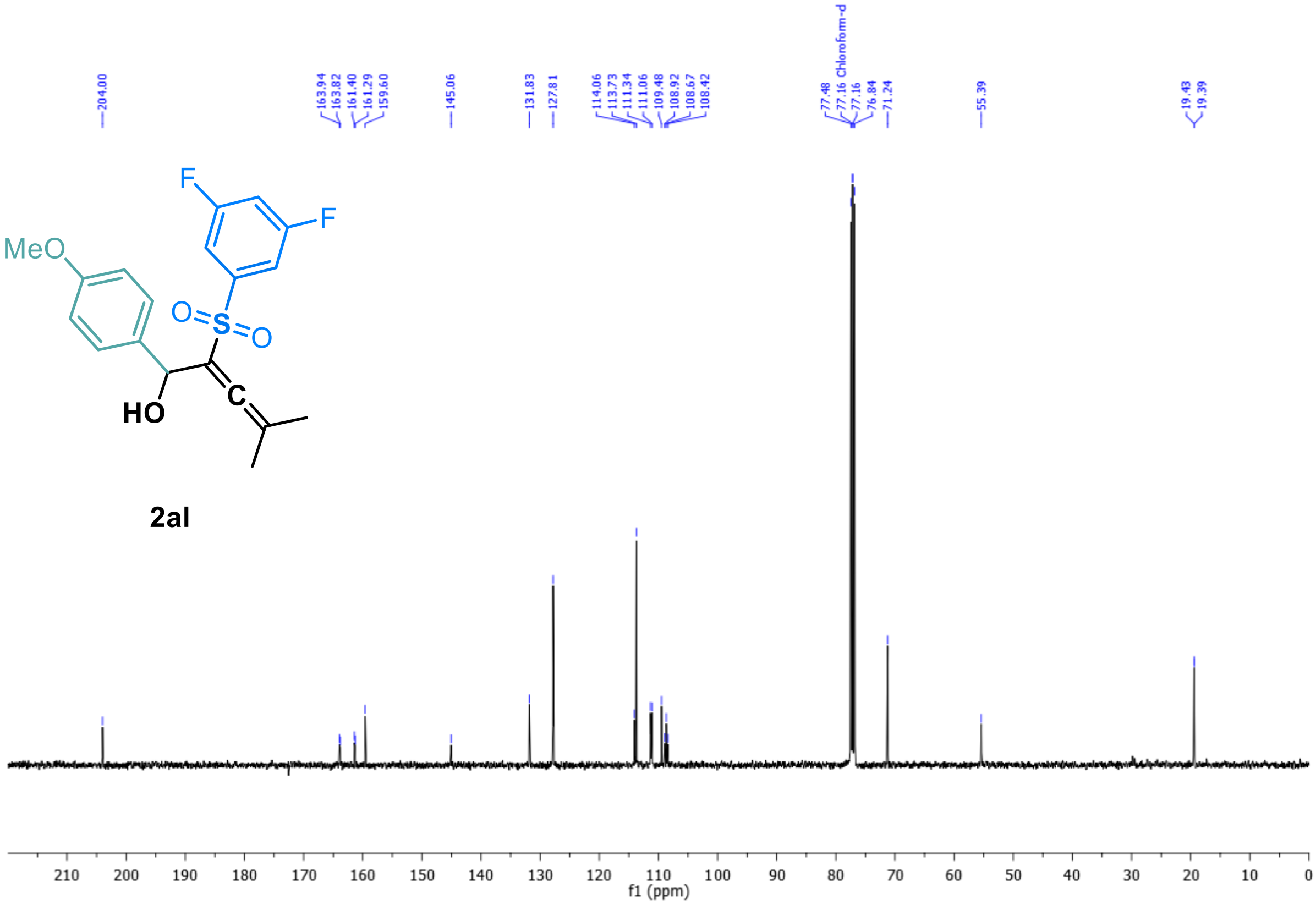

Figure S28: *^{13}C-NMR of 2**al** in $CDCl_3$ (101 MHz)*

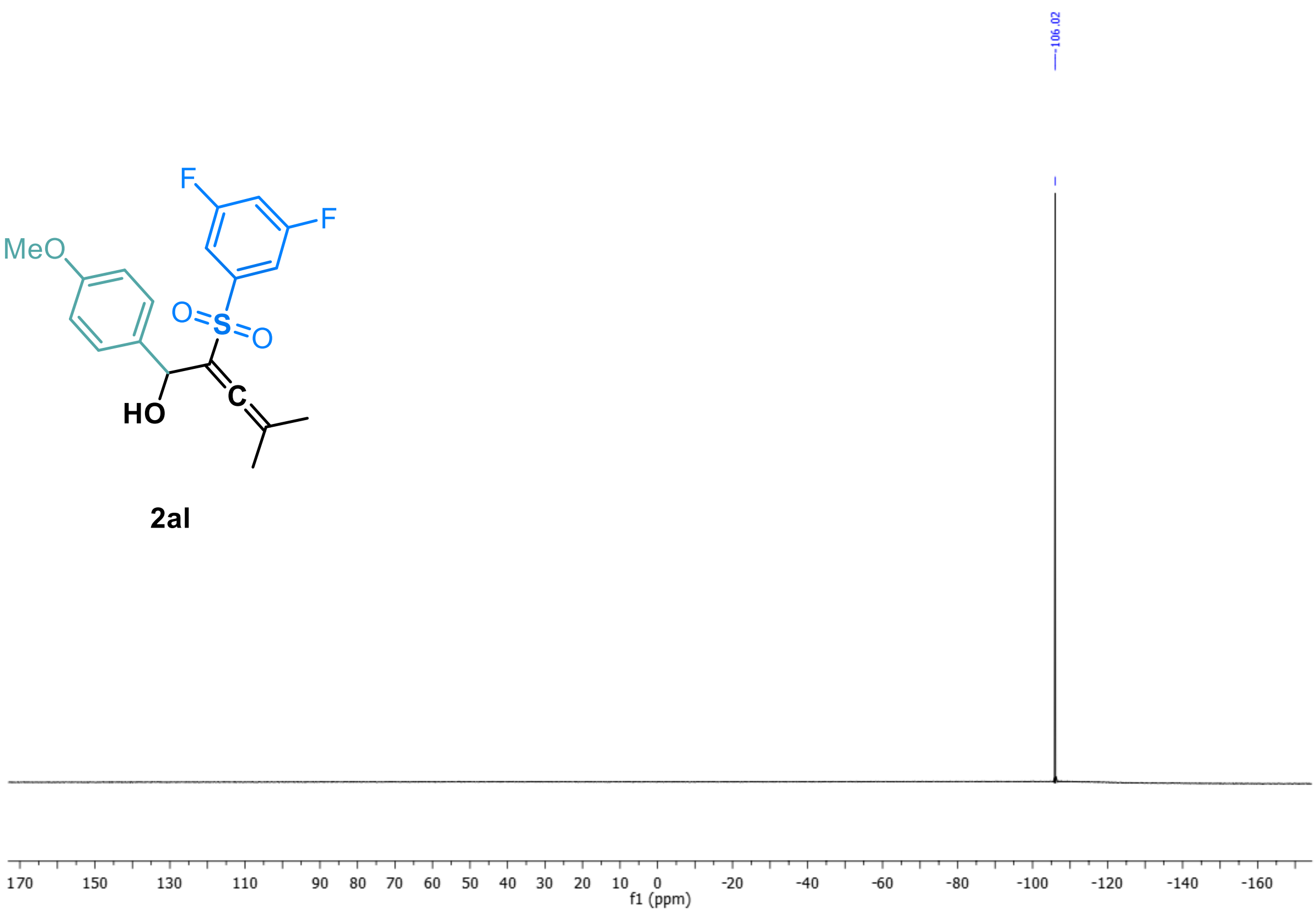

Figure S29:*^{19}F-NMR of **2al** in $CDCl_3$ (376 MHz)*

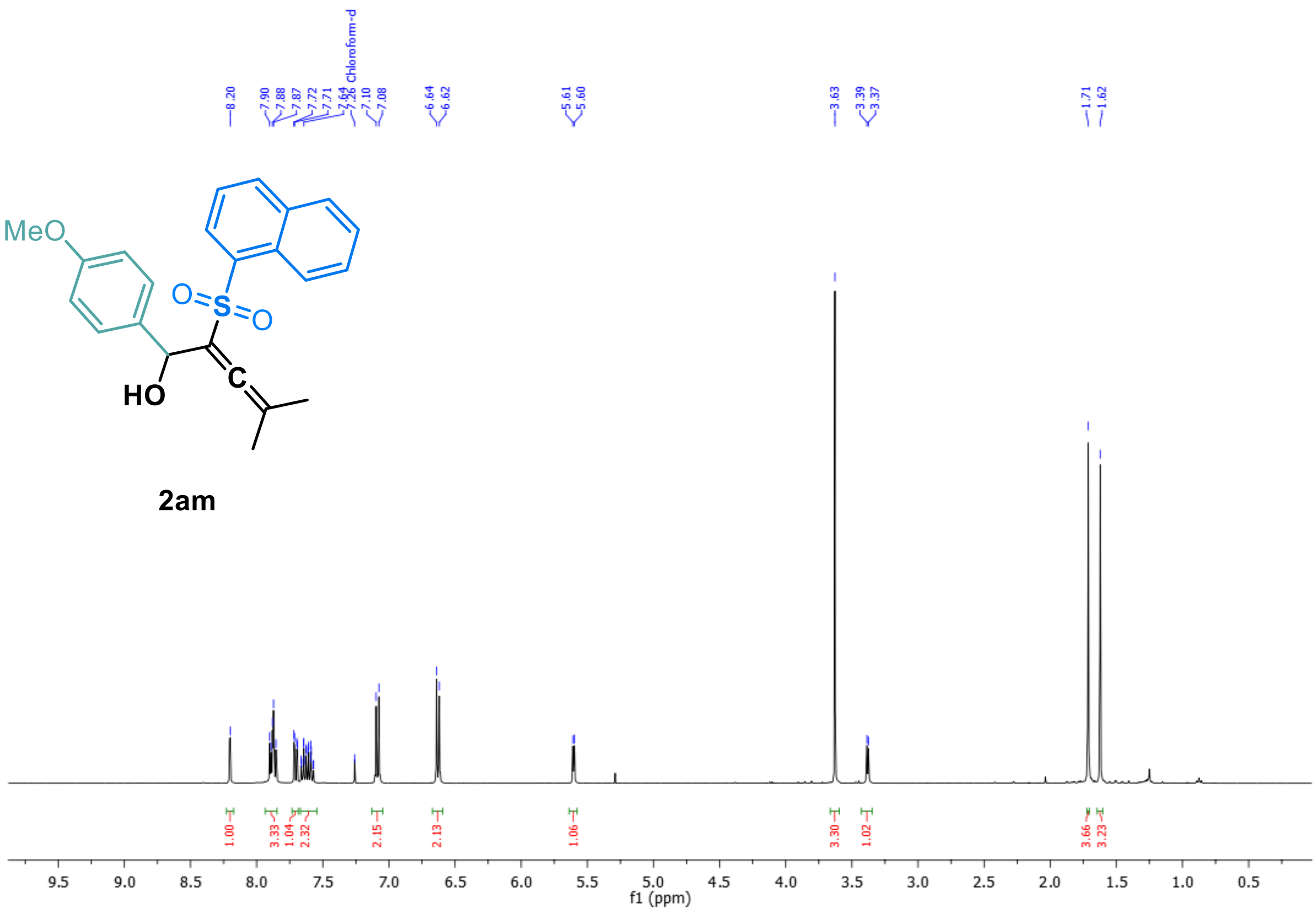

Figure S30: *^{1}H-NMR of **2am** in $CDCl_3$ (400 MHz)*

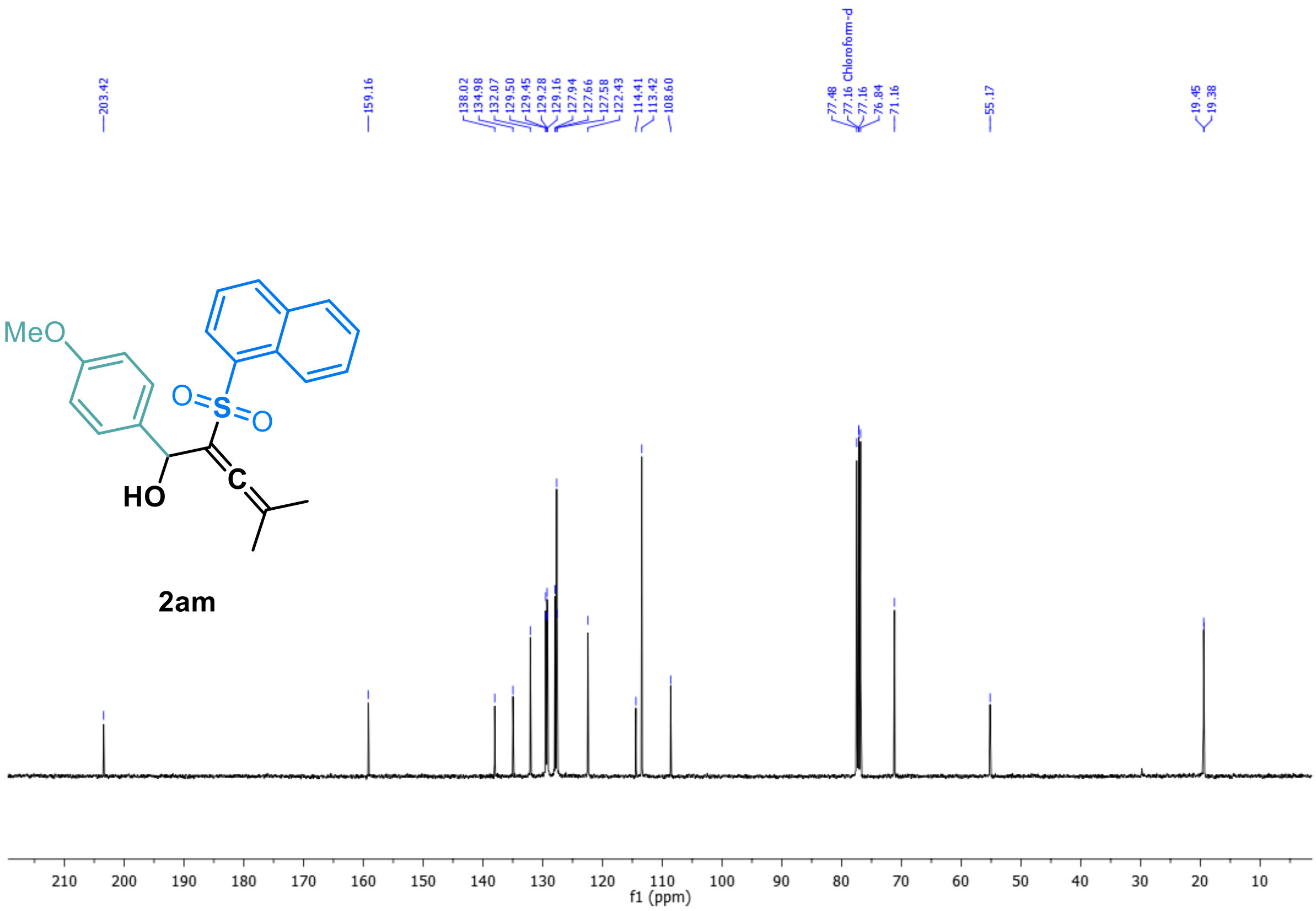

Figure S31: *^{13}C-NMR of 2**am** in $CDCl_3$ (101 MHz)*

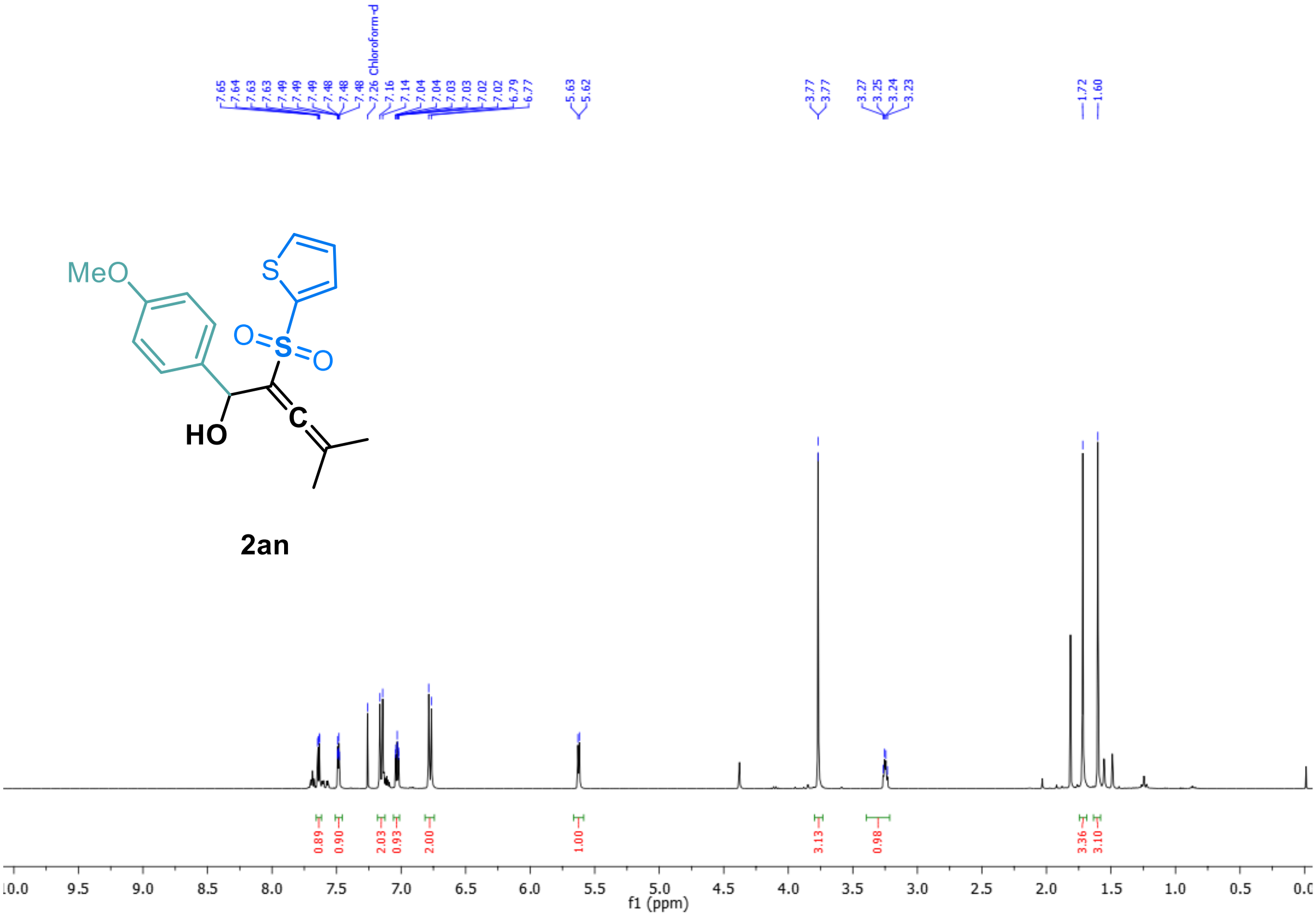

Figure S32: *¹H-NMR of* ***2an*** *in CDCl₃ (400 MHz)*

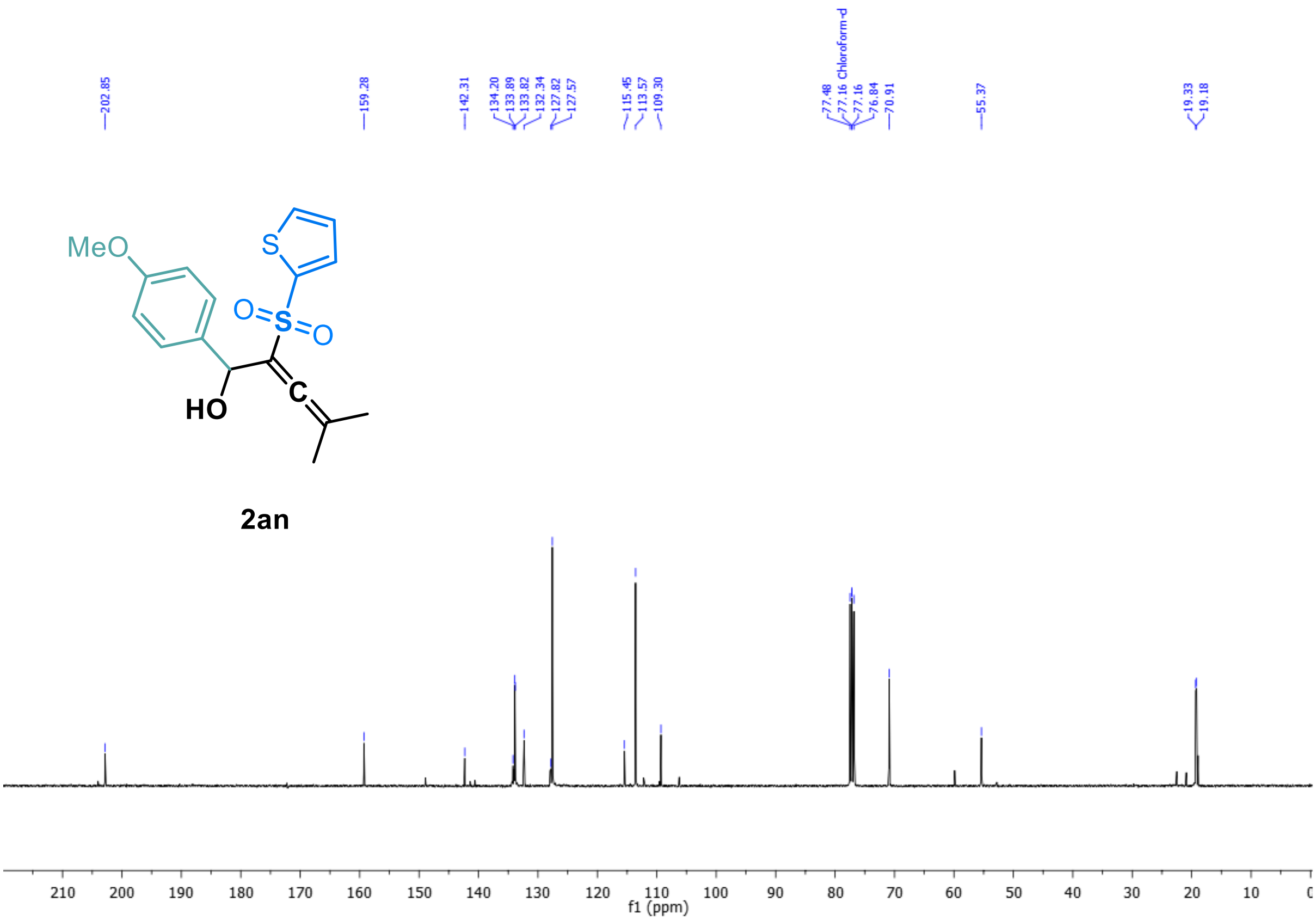

Figure S33 *^{13}C-NMR of* ***2an*** *in $CDCl_3$ (101 MHz)*

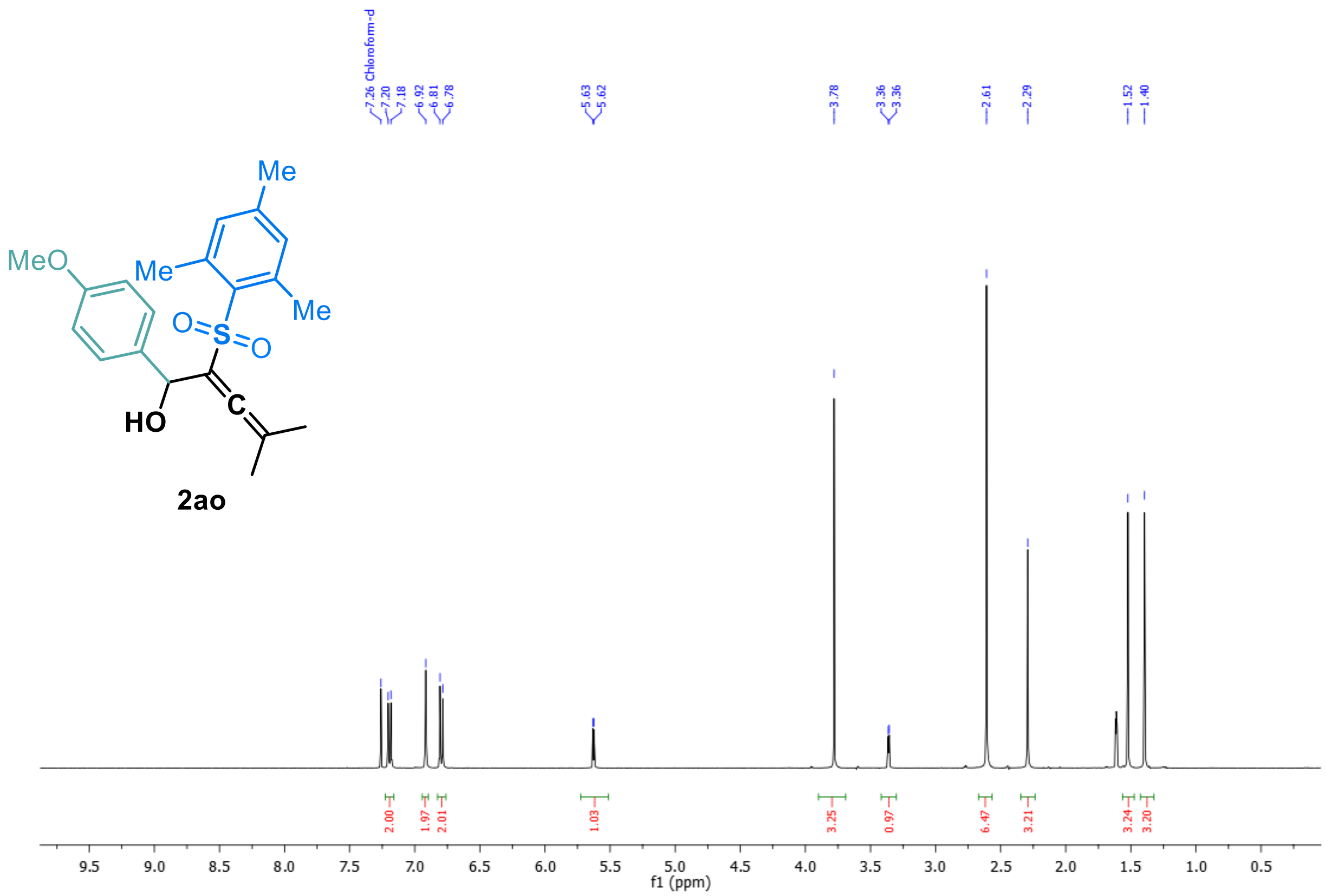

Figure S34: *^{1}H-NMR of **2ao** in $CDCl_3$ (400 MHz)*

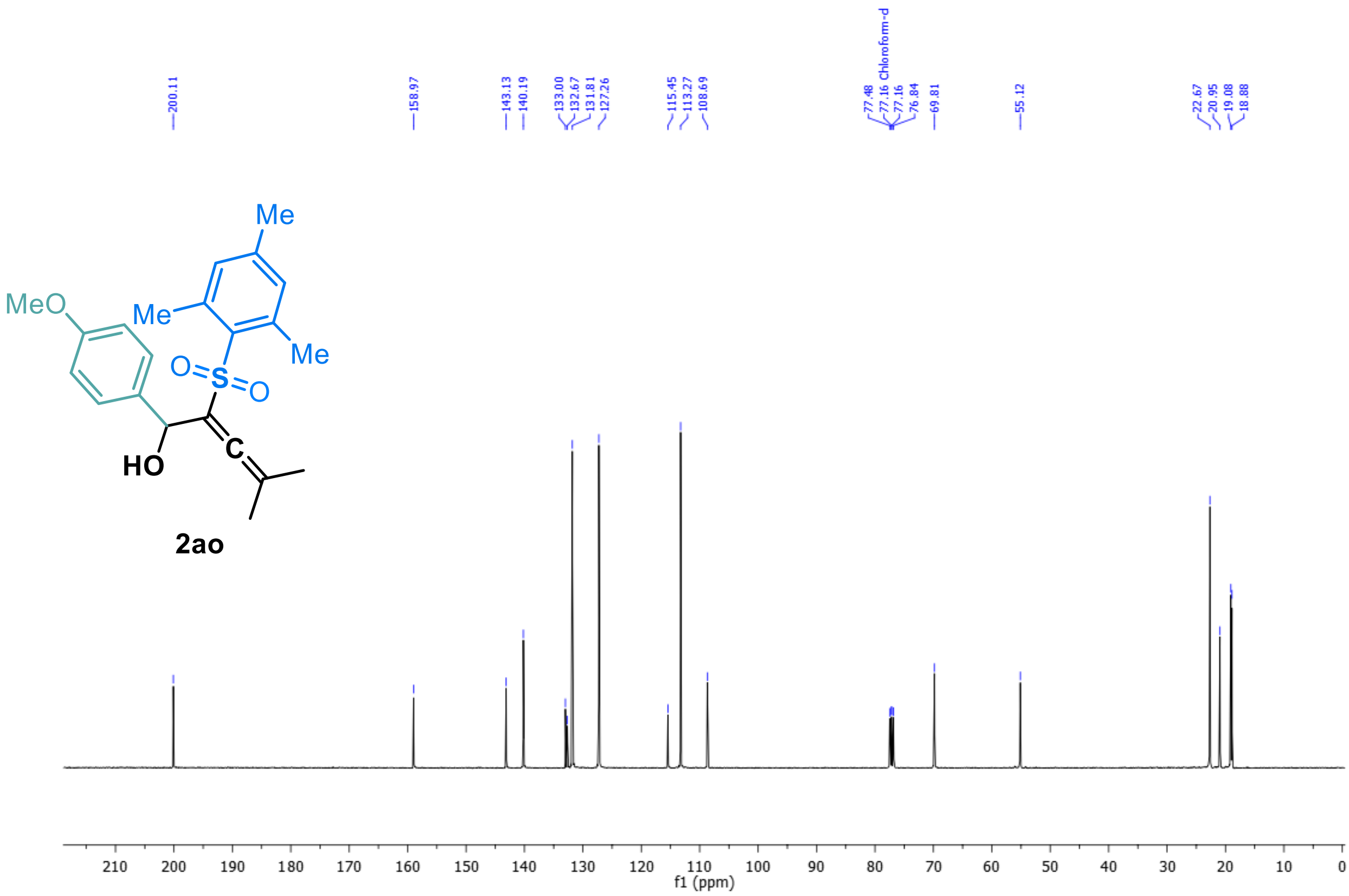

Figure S35: *^{13}C-NMR of 2**ao** in $CDCl_3$ (101 MHz)*

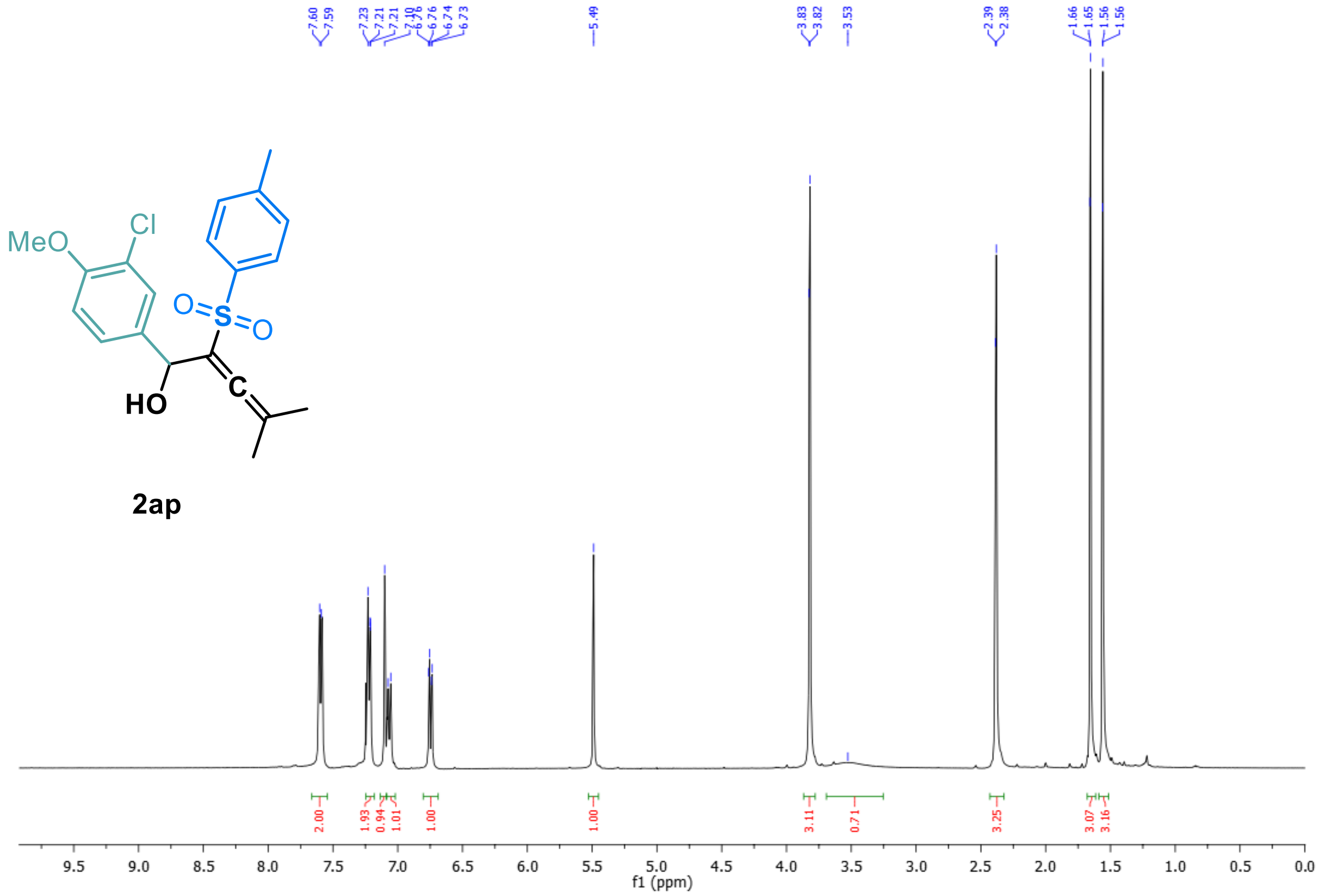

Figure S36: ^{1}H-NMR of **2ap** in $CDCl_3$ (400 MHz)

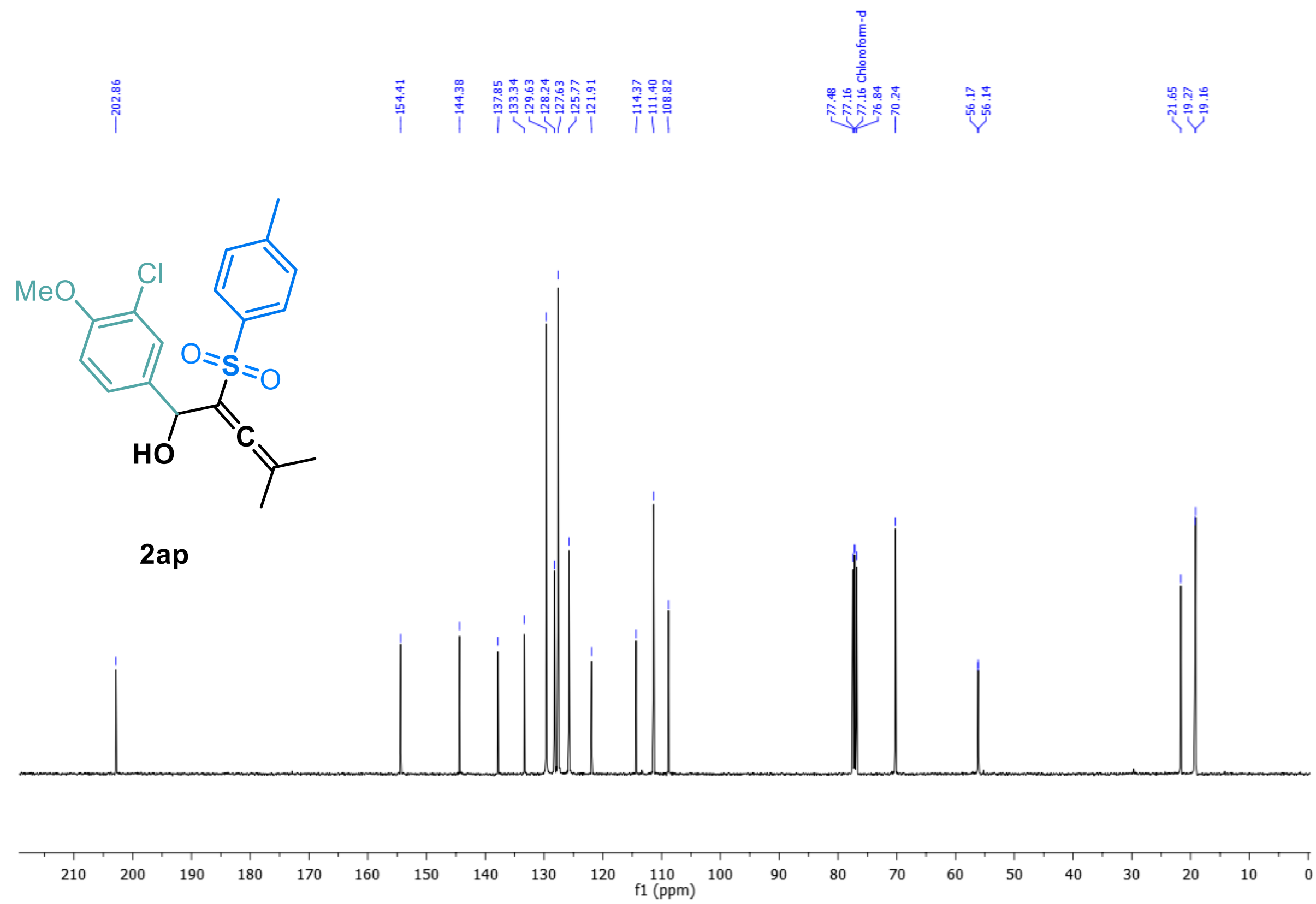

Figure S37: ^{13}C-NMR of 2**ap** in $CDCl_3$ (101 MHz)

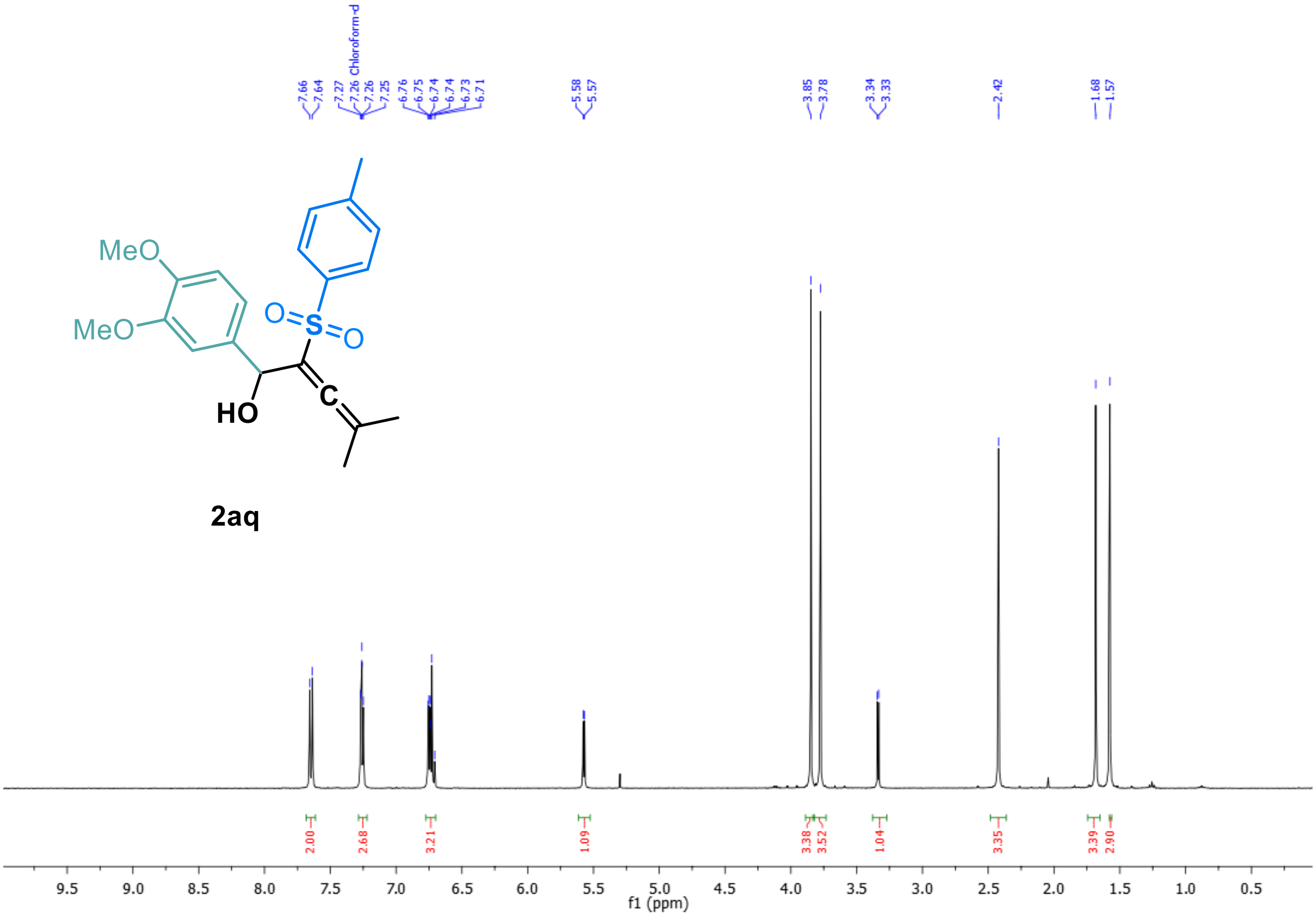

Figure S38: *^{1}H-NMR of* ***2aq*** *in* $CDCl_3$ *(400 MHz)*

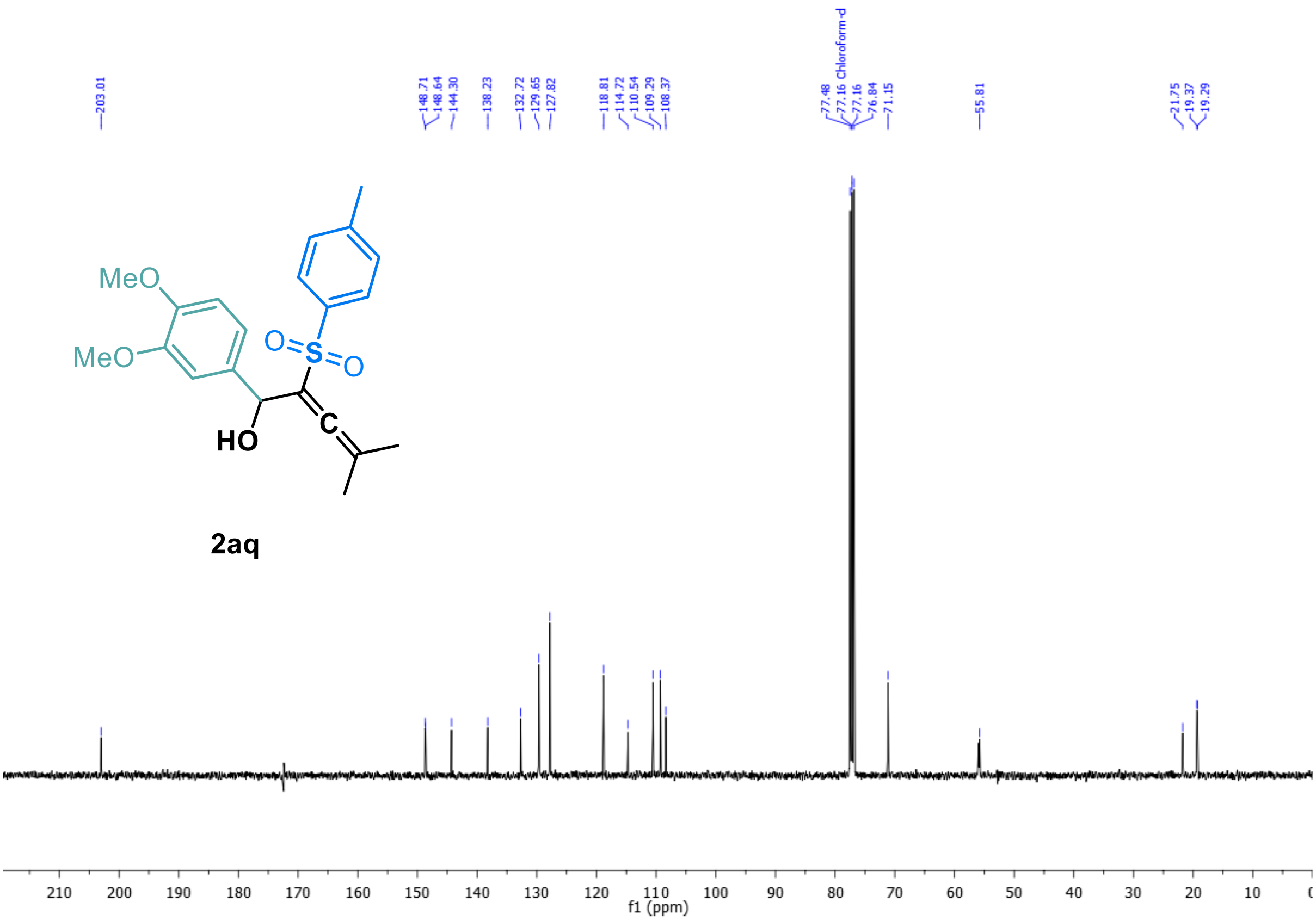

Figure S39: *^{13}C-NMR of 2****aq*** *in* $CDCl_3$ *(101 MHz)*

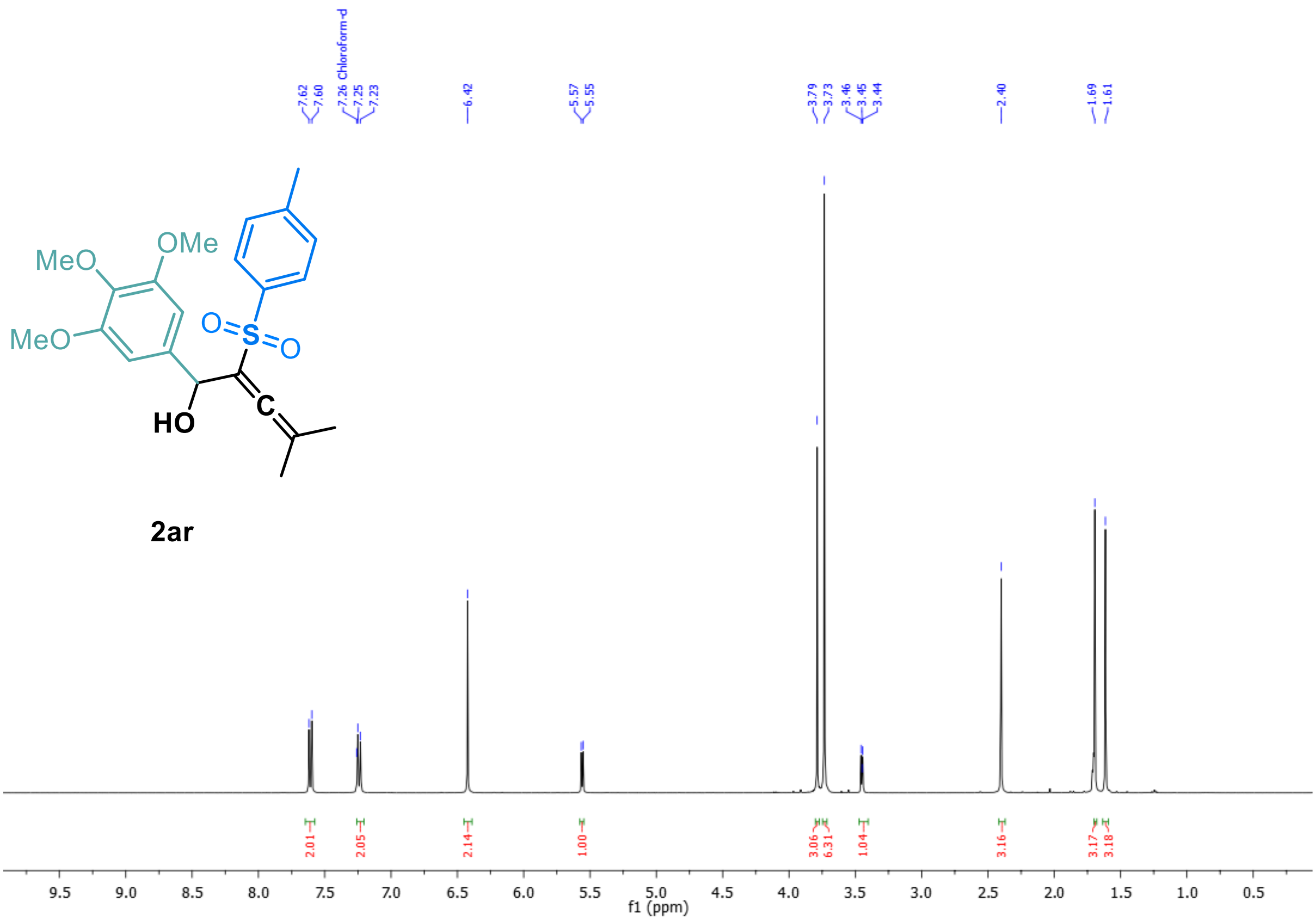

Figure S40: *^{1}H-NMR of **2ar** in $CDCl_3$ (400 MHz)*

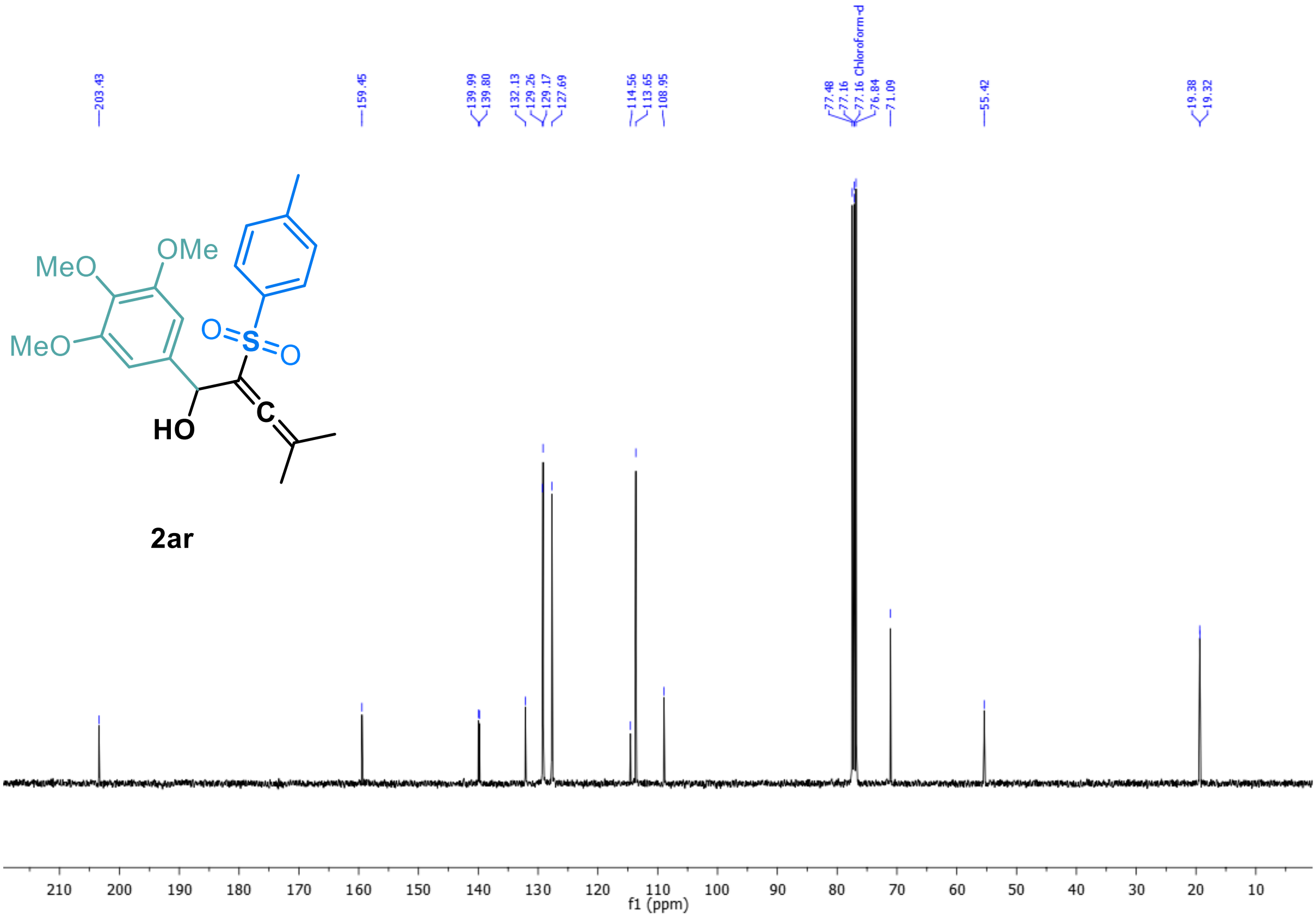

Figure S41: *^{13}C-NMR of **2ar** in $CDCl_3$ (101 MHz)*

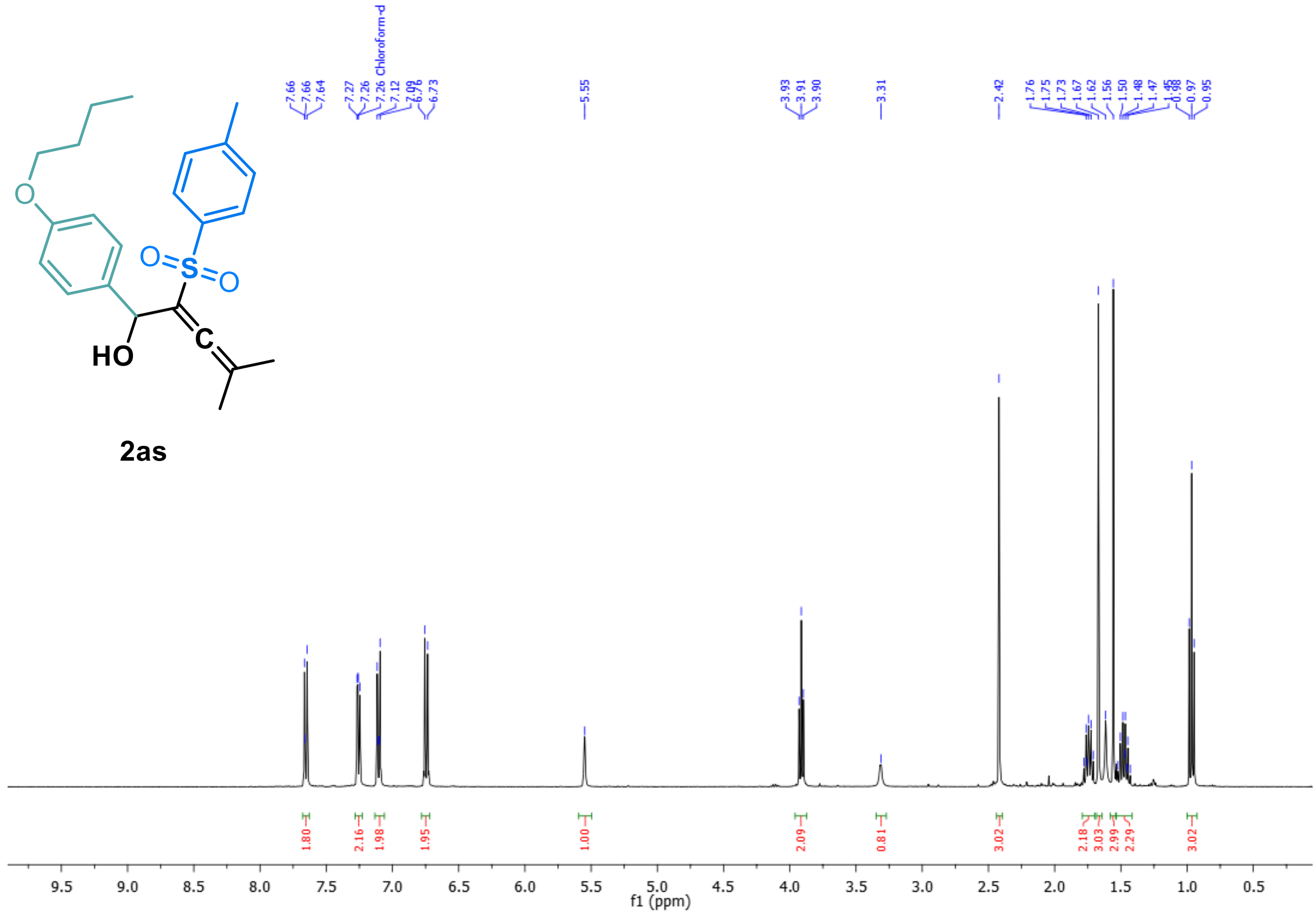

Figure S42: ^{1}H-NMR of **2as** in $CDCl_3$ (400 MHz)

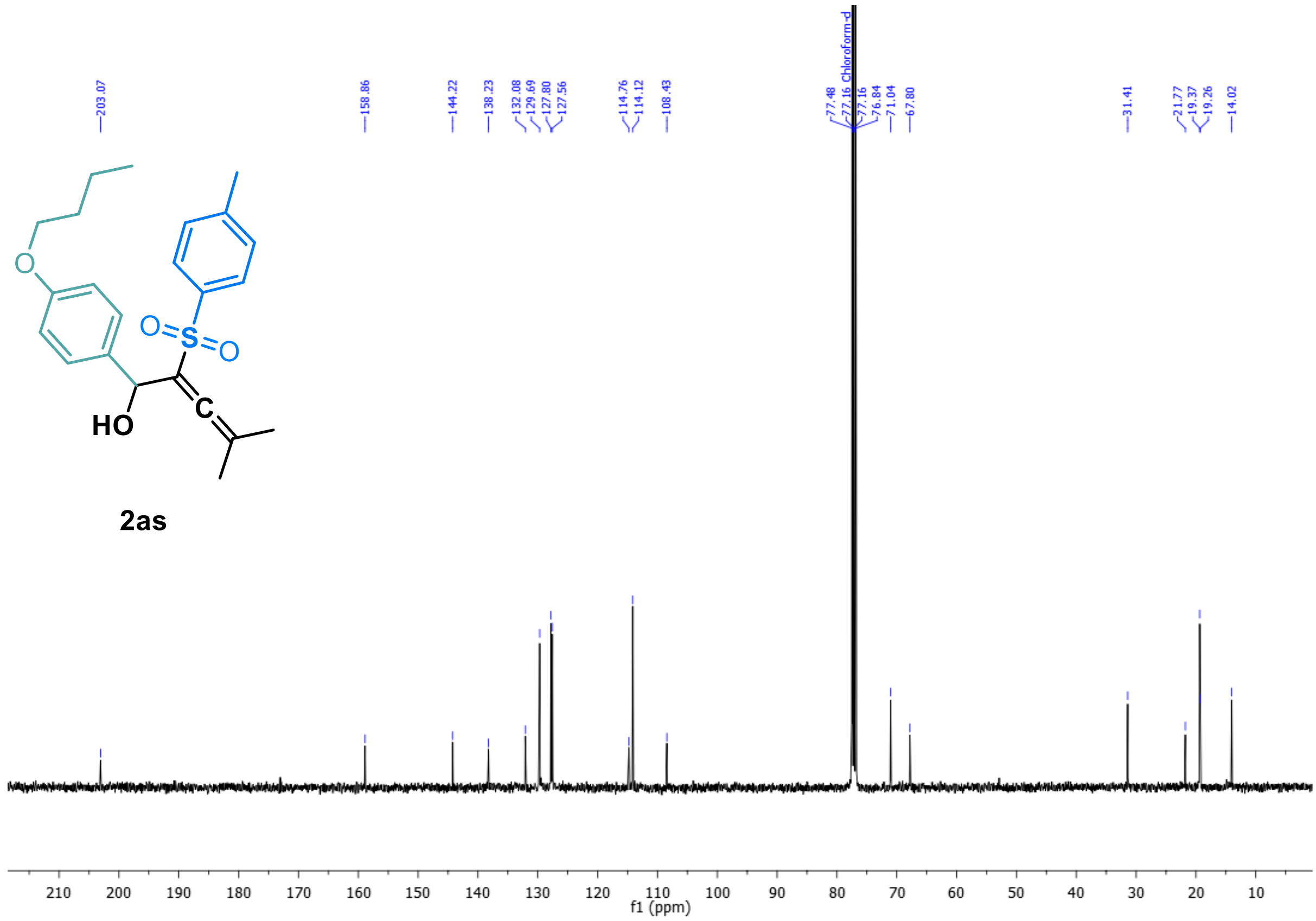

Figure S43: ^{13}C-NMR of 2**as** in $CDCl_3$ (101 MHz)

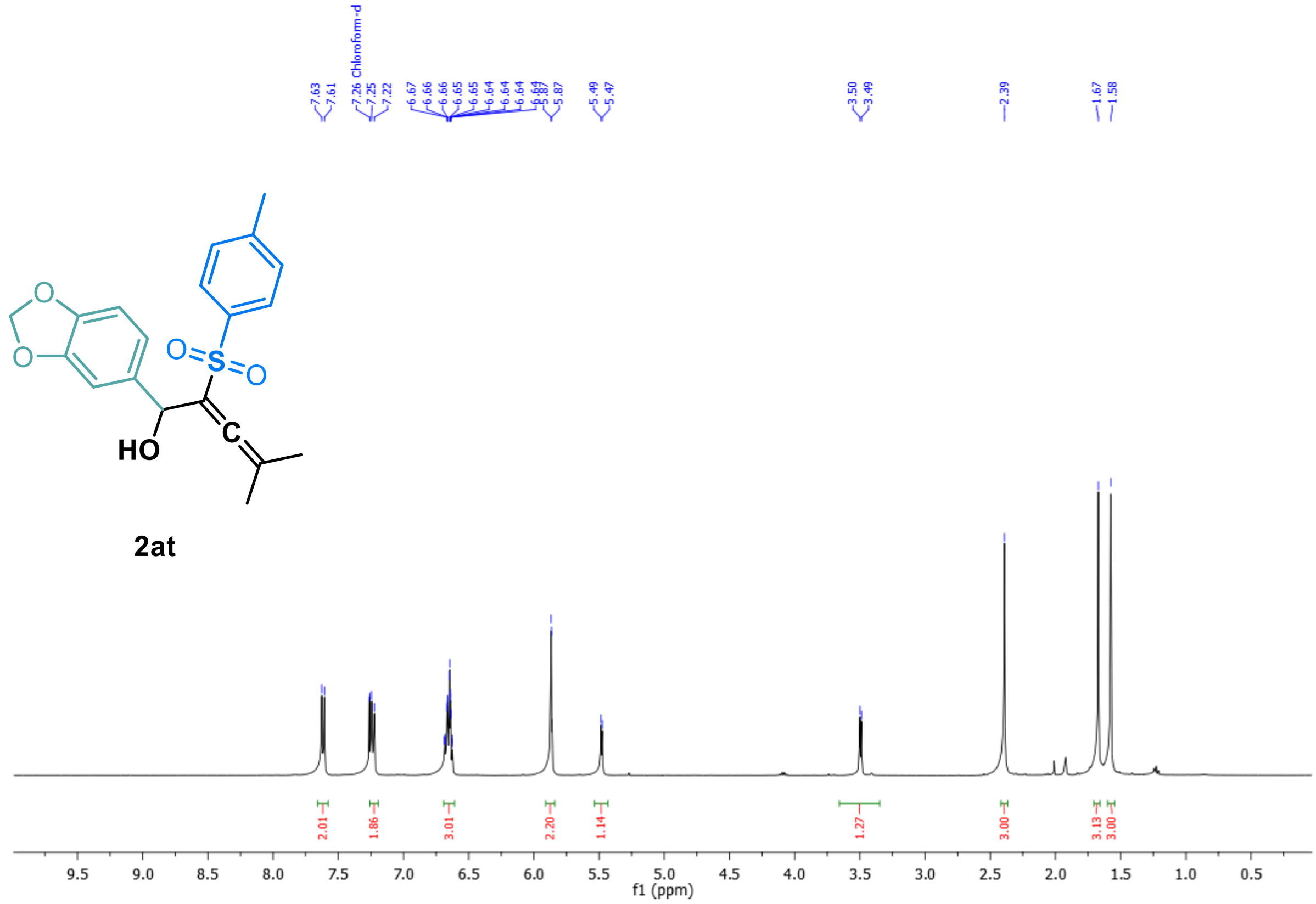

Figure S44: *^{1}H-NMR of* ***2at*** *in $CDCl_3$ (400 MHz)*

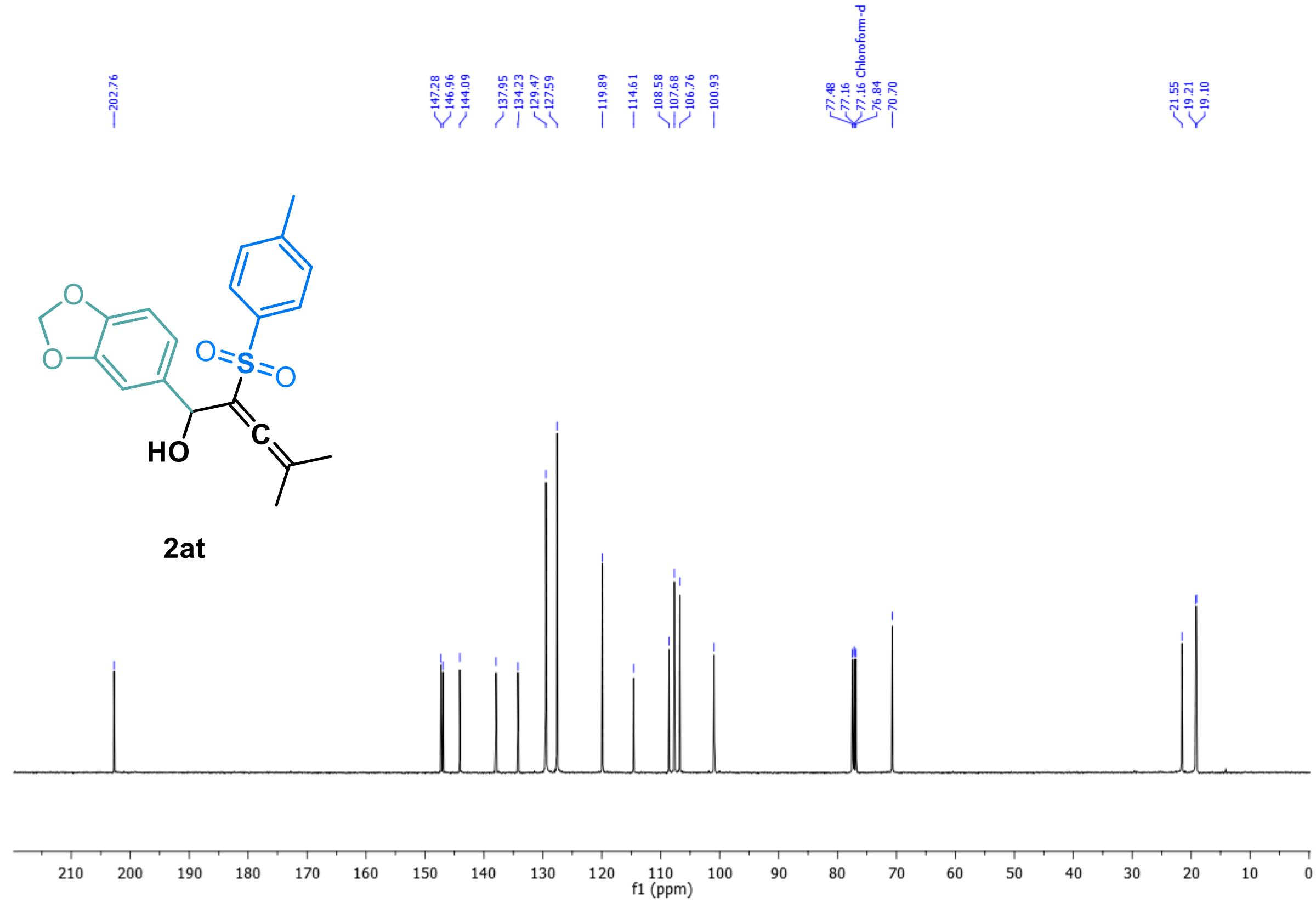

Figure S45: *^{13}C-NMR of 2****at*** *in $CDCl_3$ (101 MHz)*

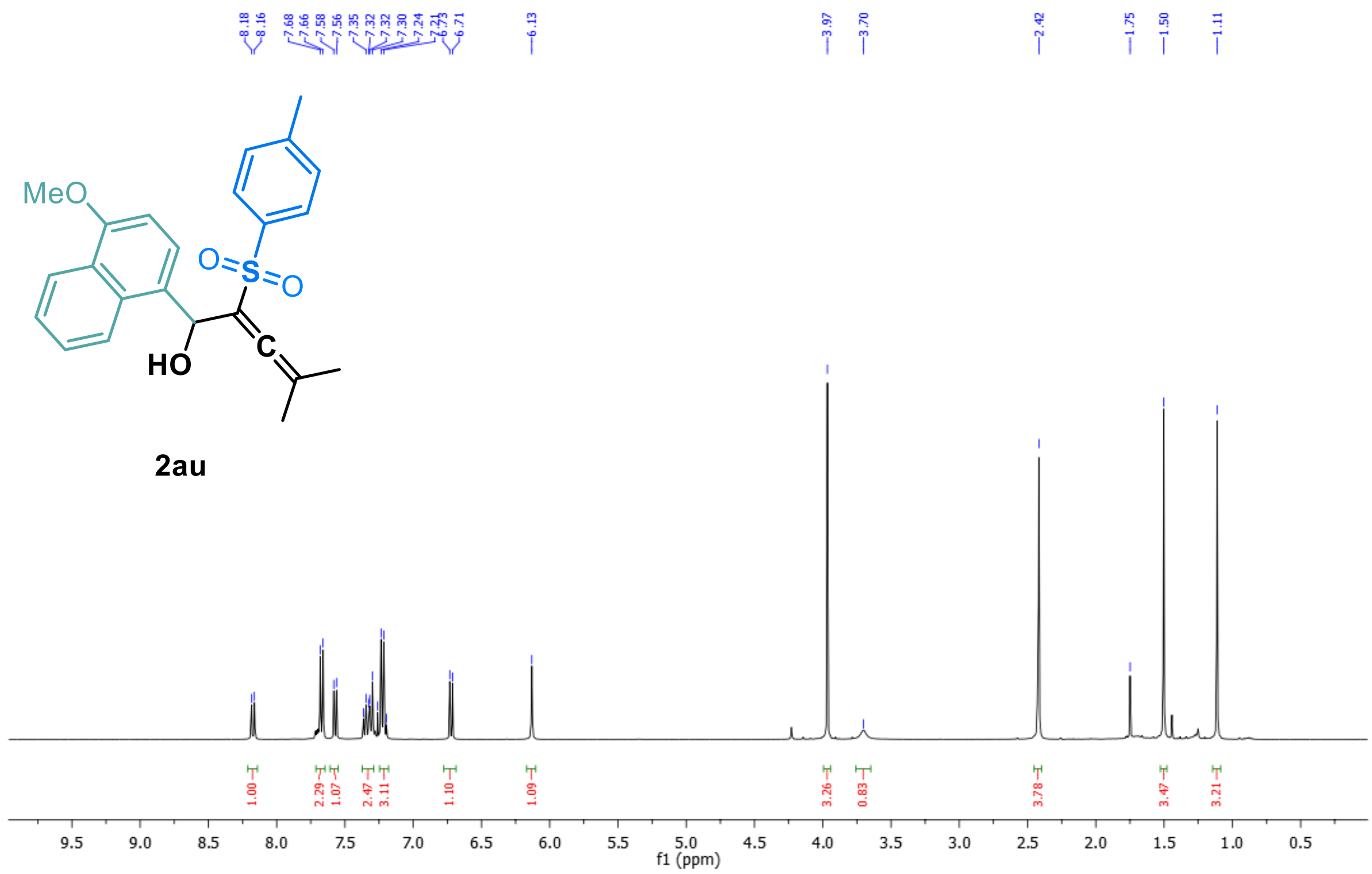

Figure S46: *^{1}H-NMR of **2au** in $CDCl_3$ (400 MHz)*

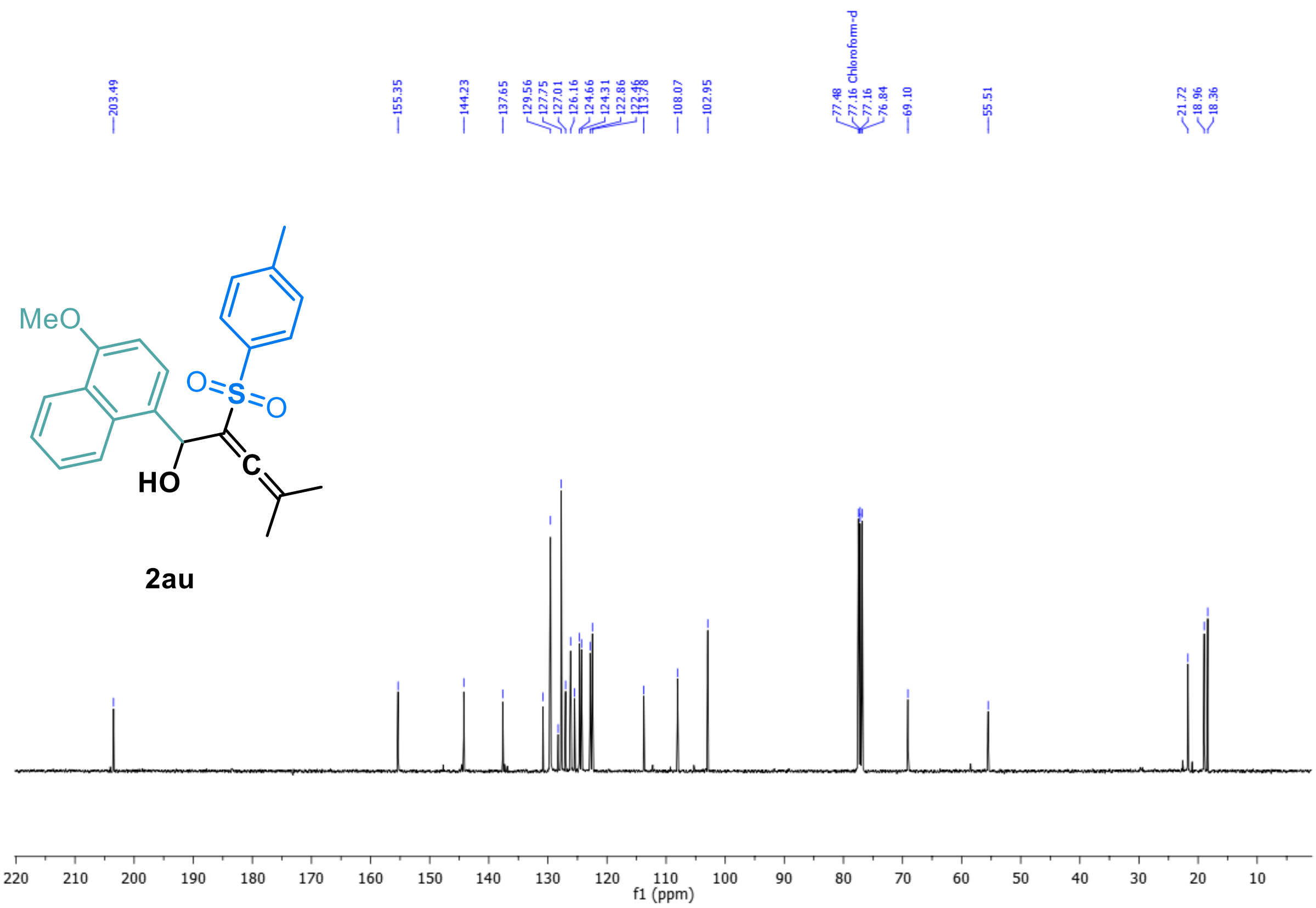

Figure S47: *^{13}C-NMR of 2**au** in $CDCl_3$ (101 MHz)*

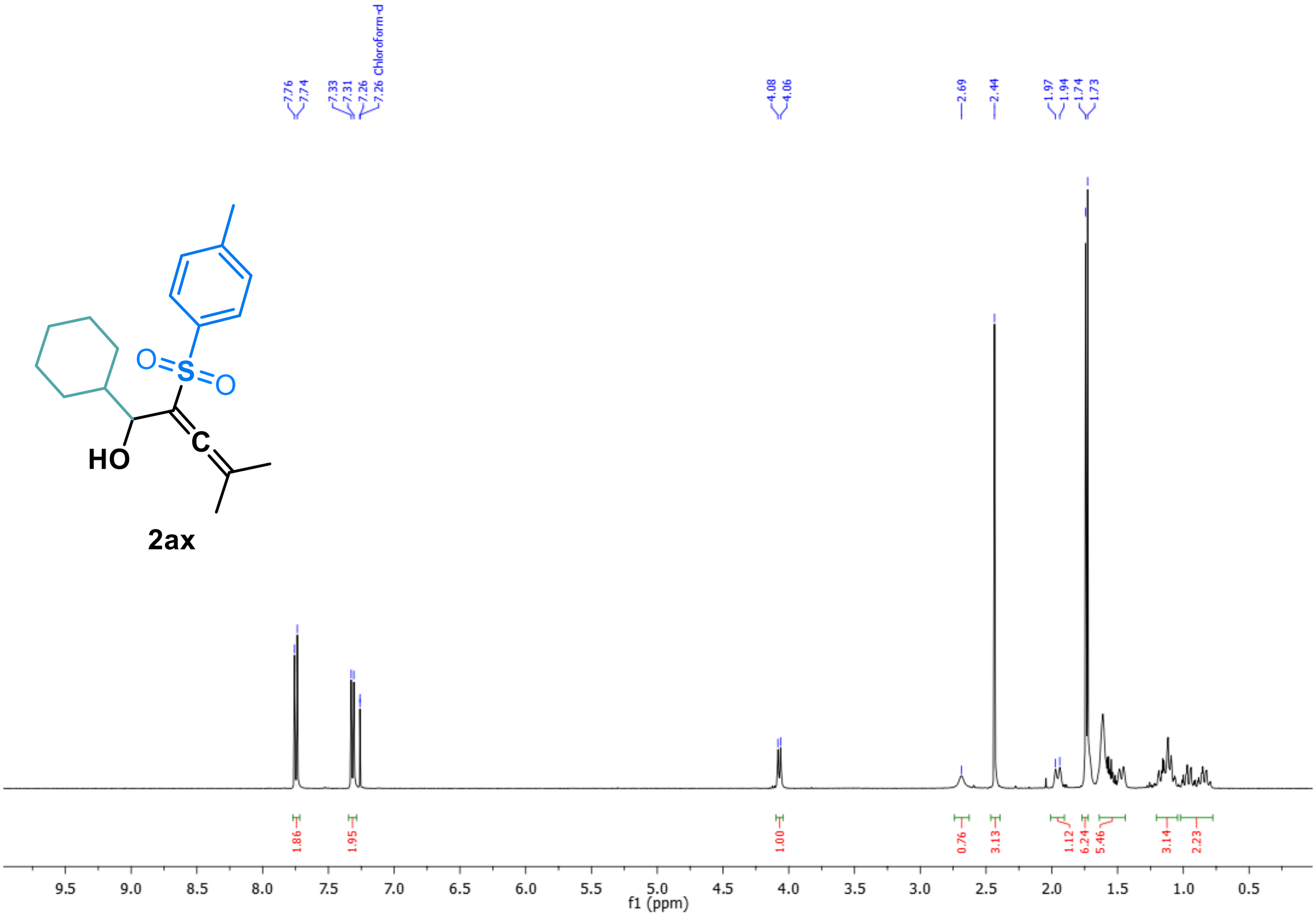

Figure S 48: *^{1}H-NMR of **2ax** in $CDCl_3$ (400 MHz)*

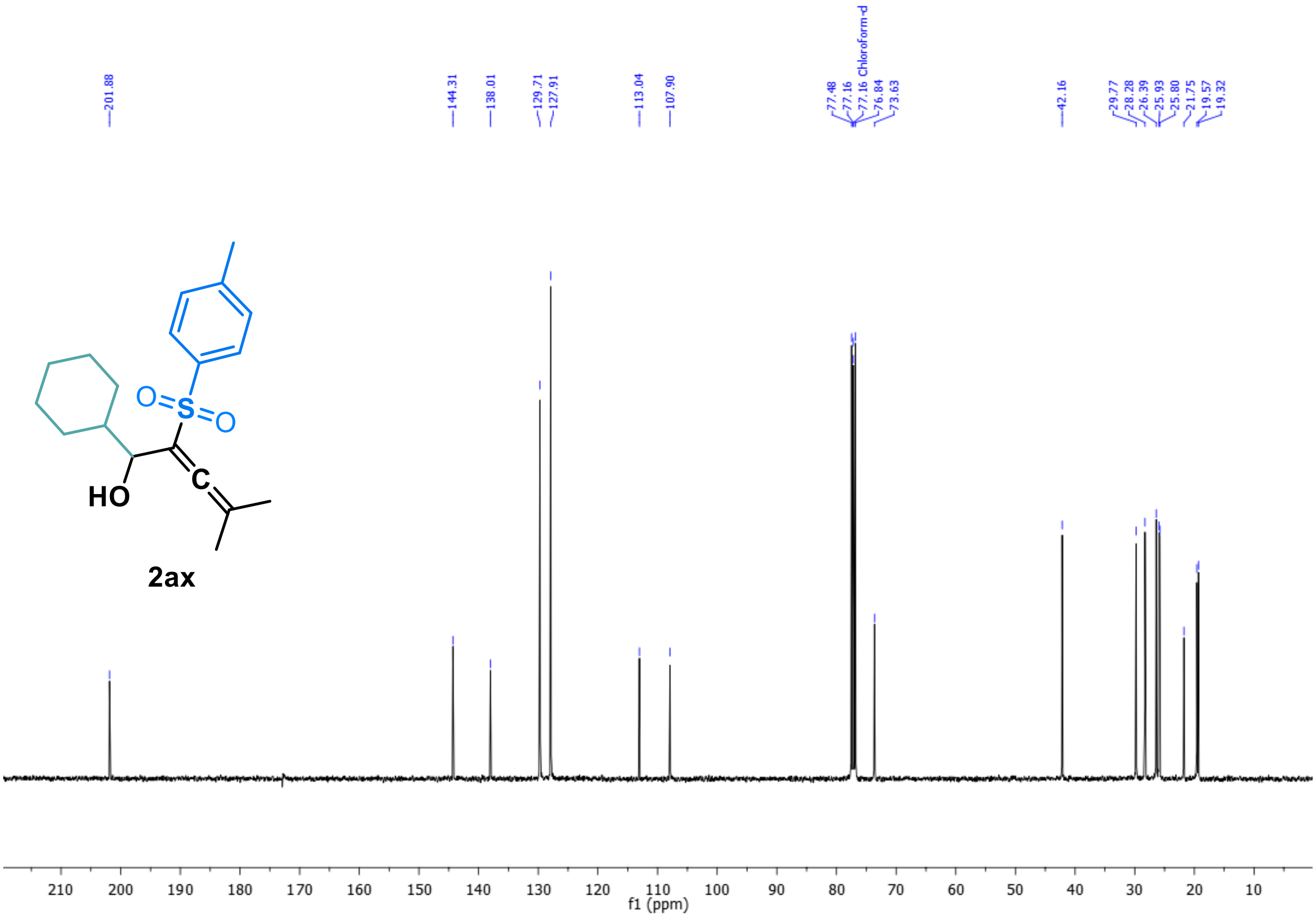

***Figure S49**: ^{13}C-NMR of 2**ax** in $CDCl_3$ (101 MHz)*

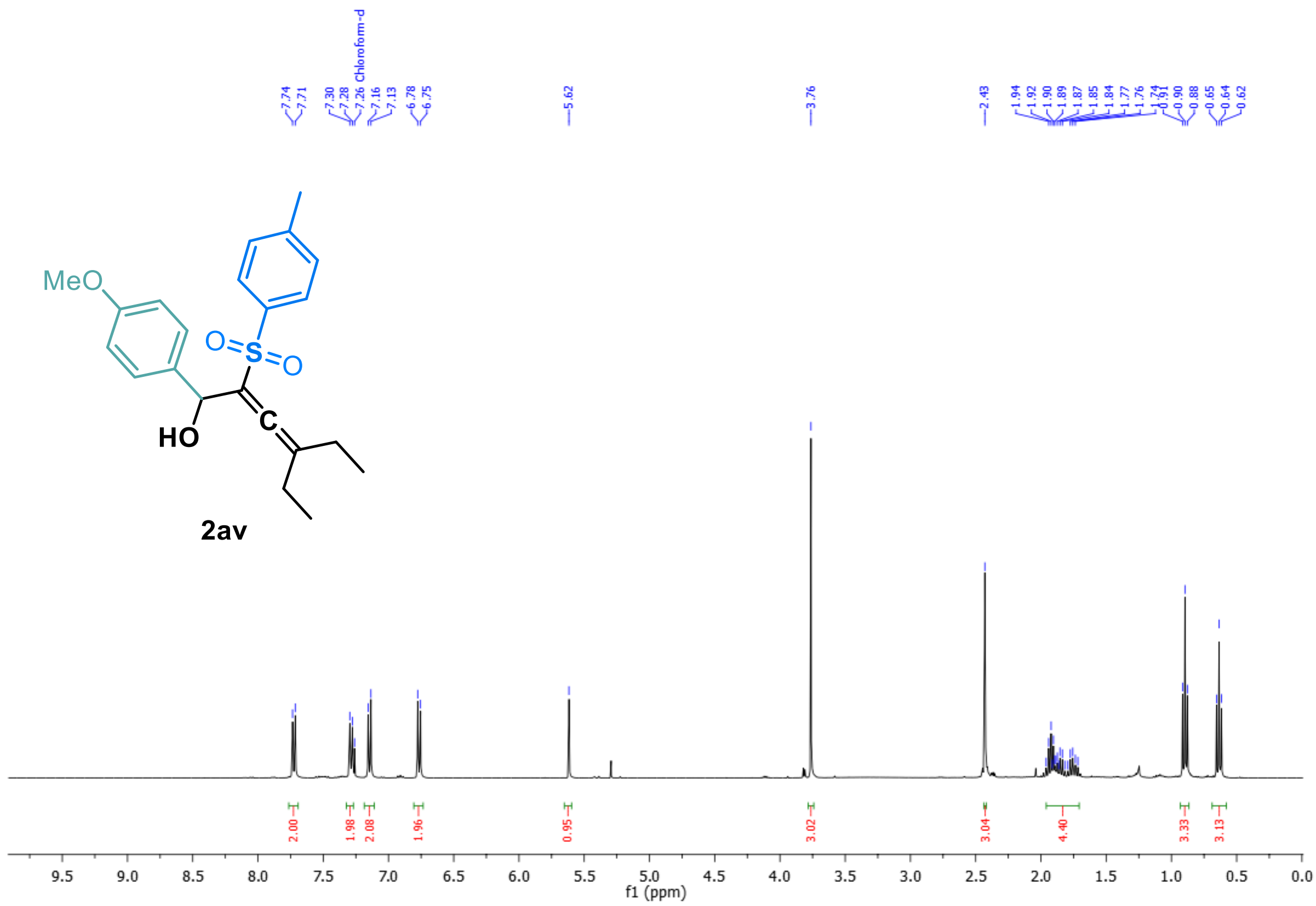

Figure S50: ^{1}H-NMR of **2av** in $CDCl_3$ (400 MHz)

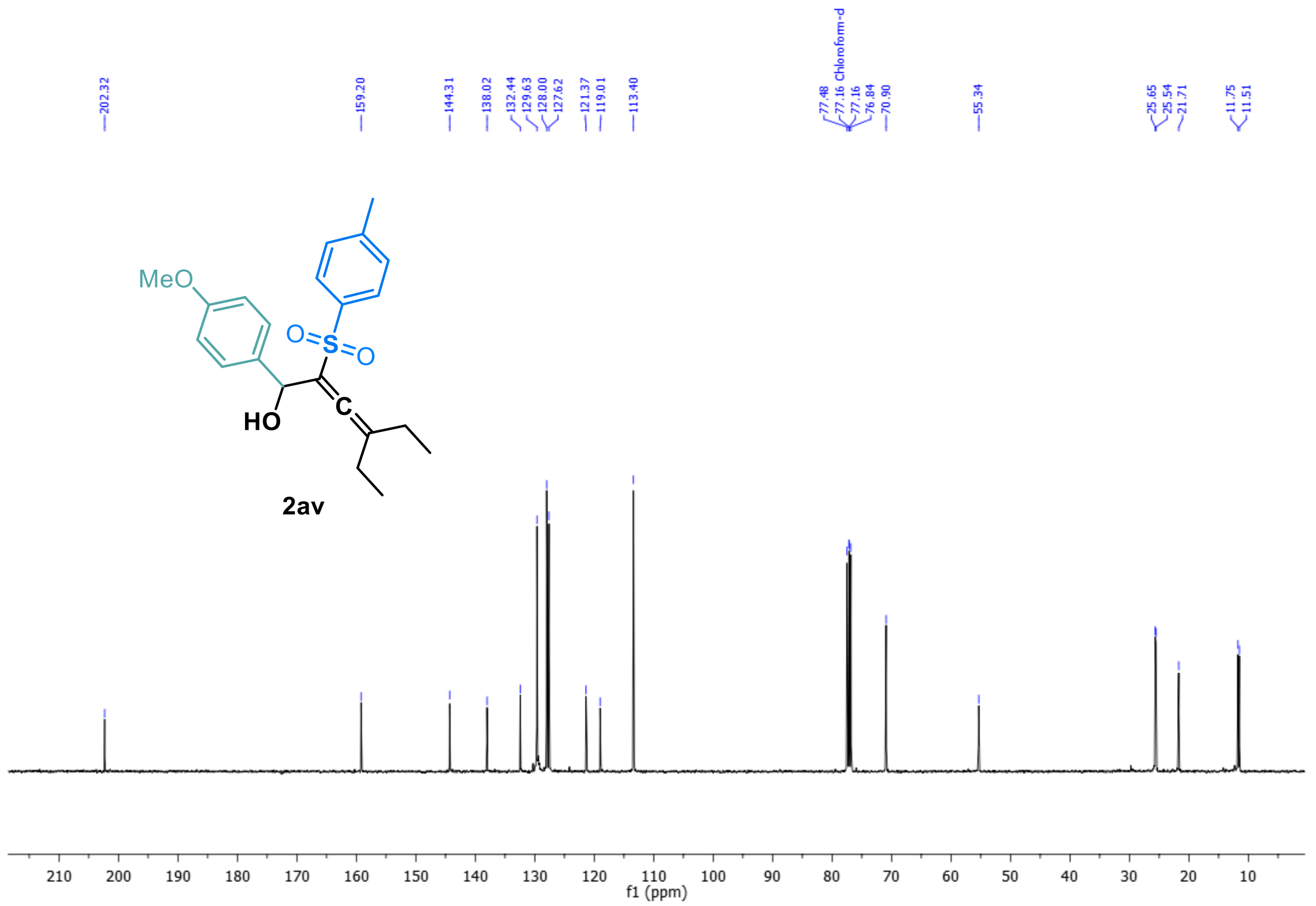

Figure S51: ^{13}C-NMR of **2av** in $CDCl_3$ (101 MHz)

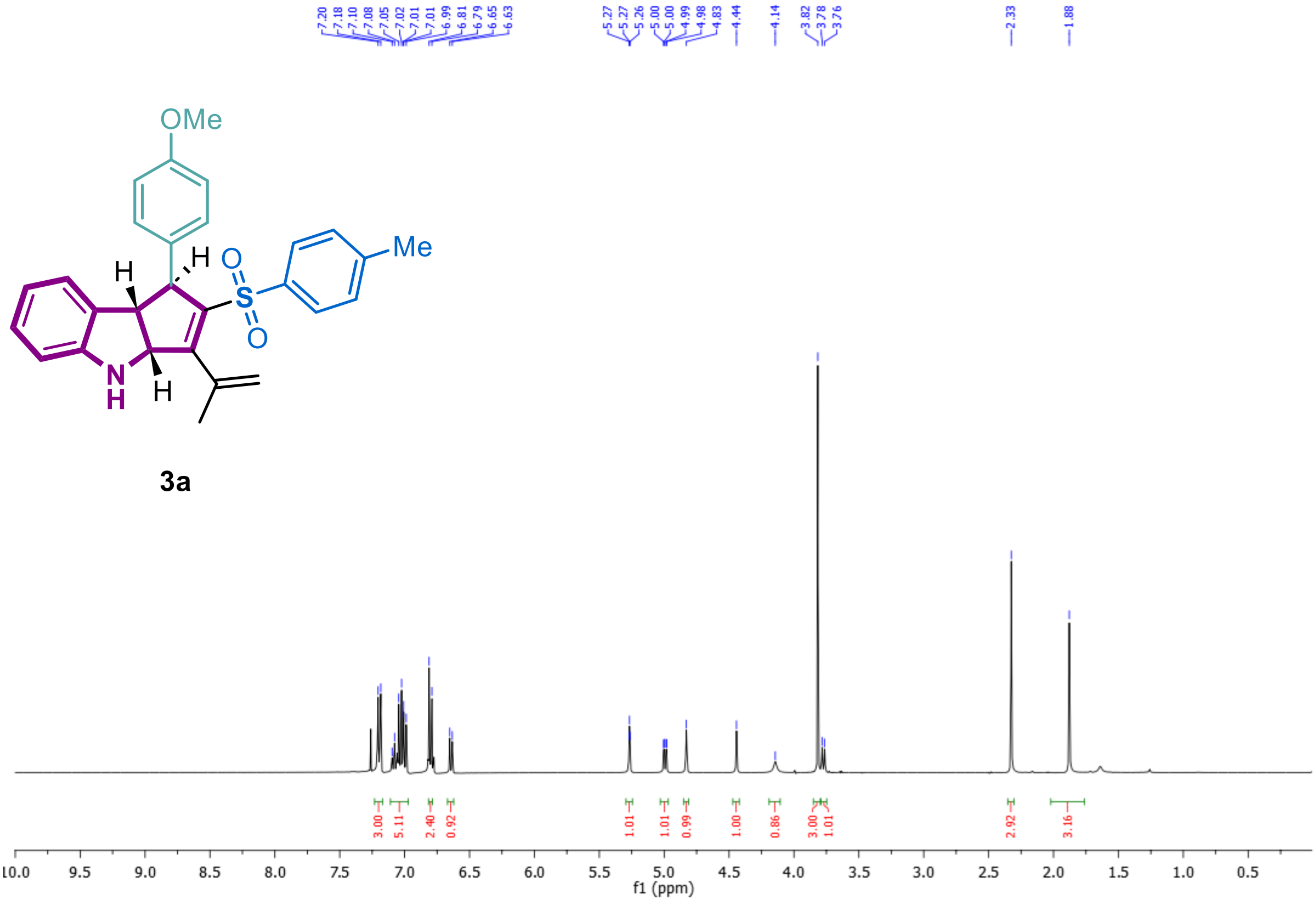

Figure S52: *^{1}H-NMR of **3a** in $CDCl_3$ (400 MHz).*

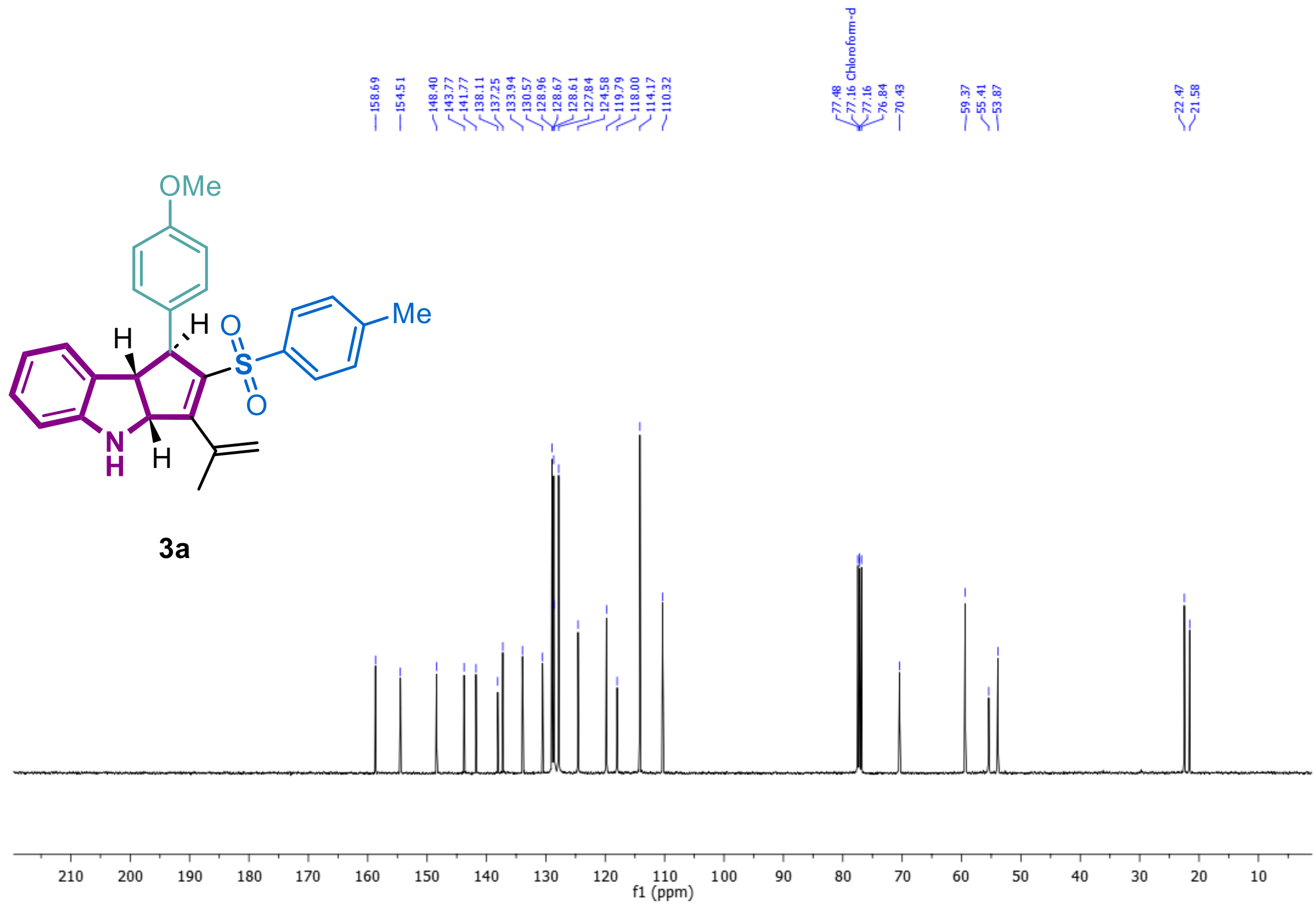

Figure S53: *^{13}C-NMR of **3a** in $CDCl_3$ (101 MHz)*

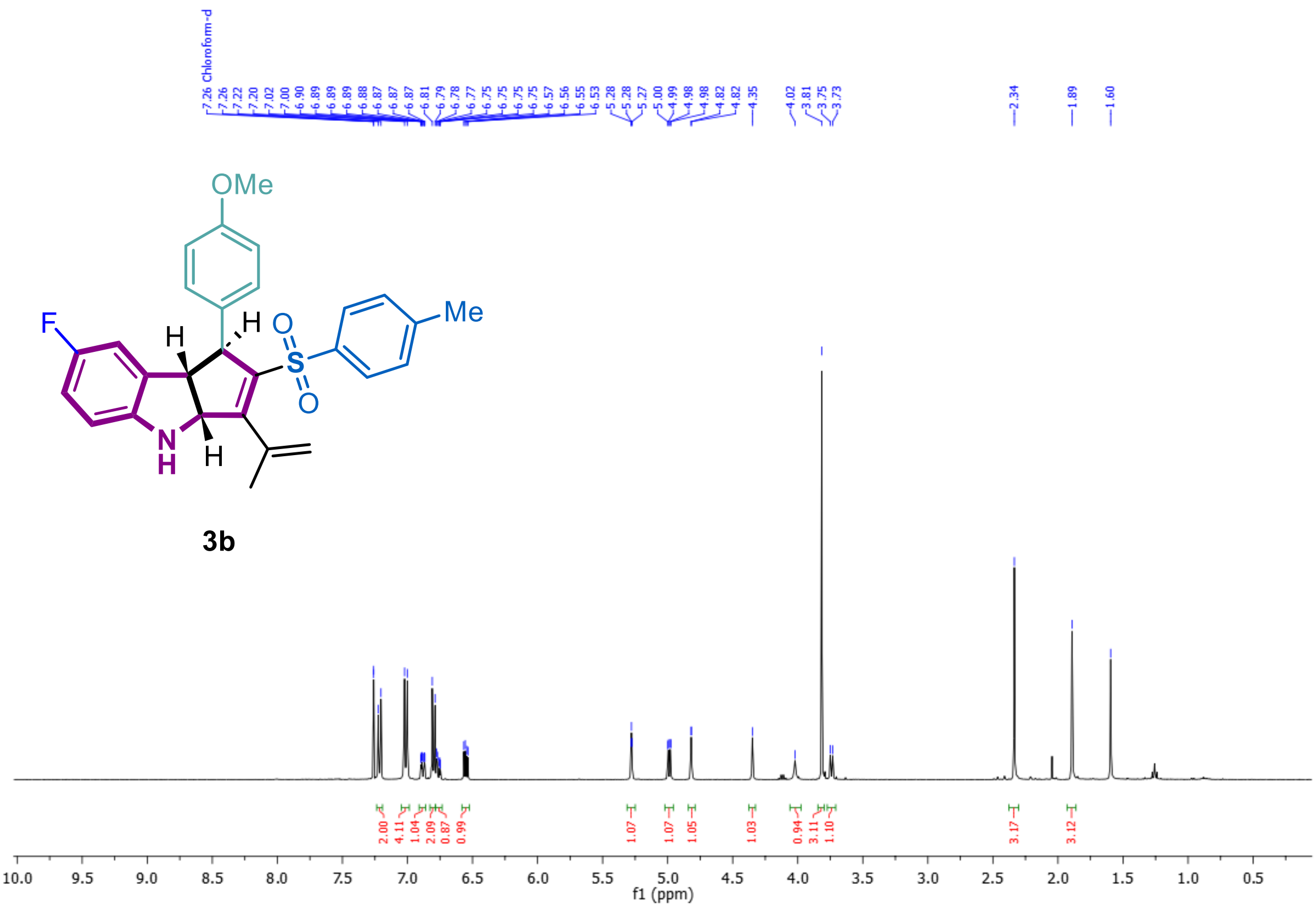

Figure S54: *^{1}H-NMR of **3b** in $CDCl_3$ (400 MHz).*

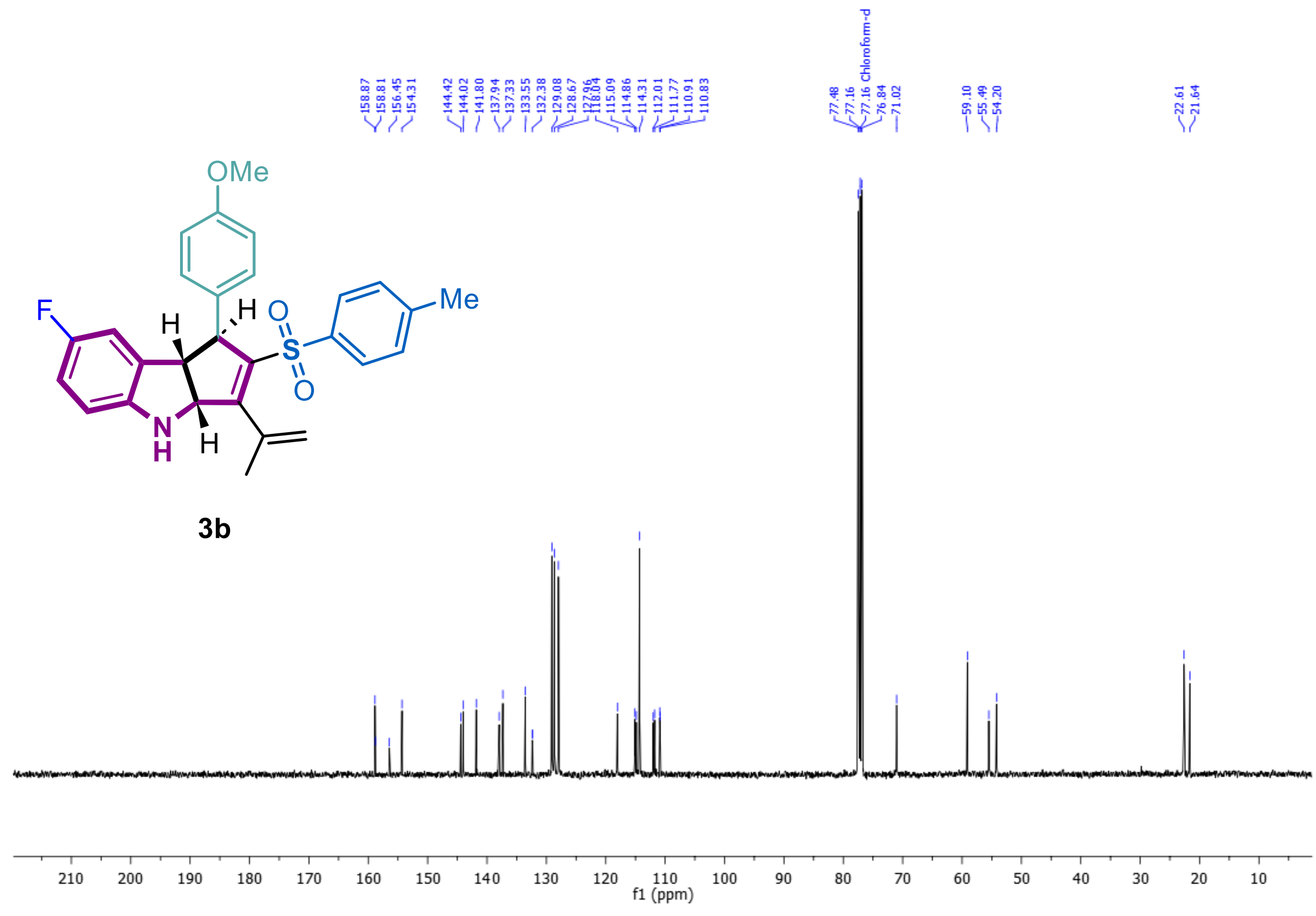

Figure S55: *^{13}C-NMR of **3b** in $CDCl_3$ (101 MHz)*

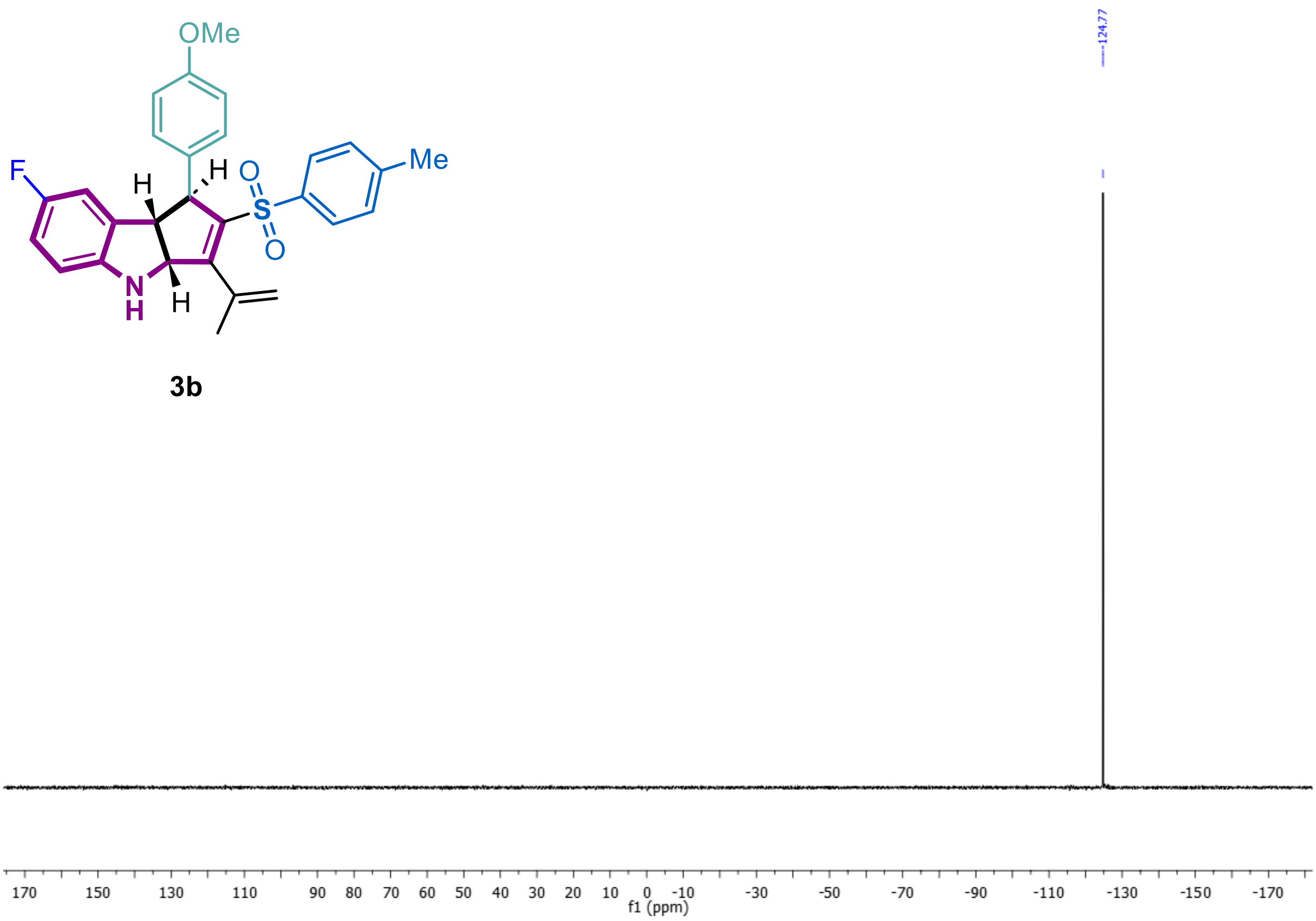

Figure S56: ^{19}F-NMR of **3b** in $CDCl_3$ (376 MHz)

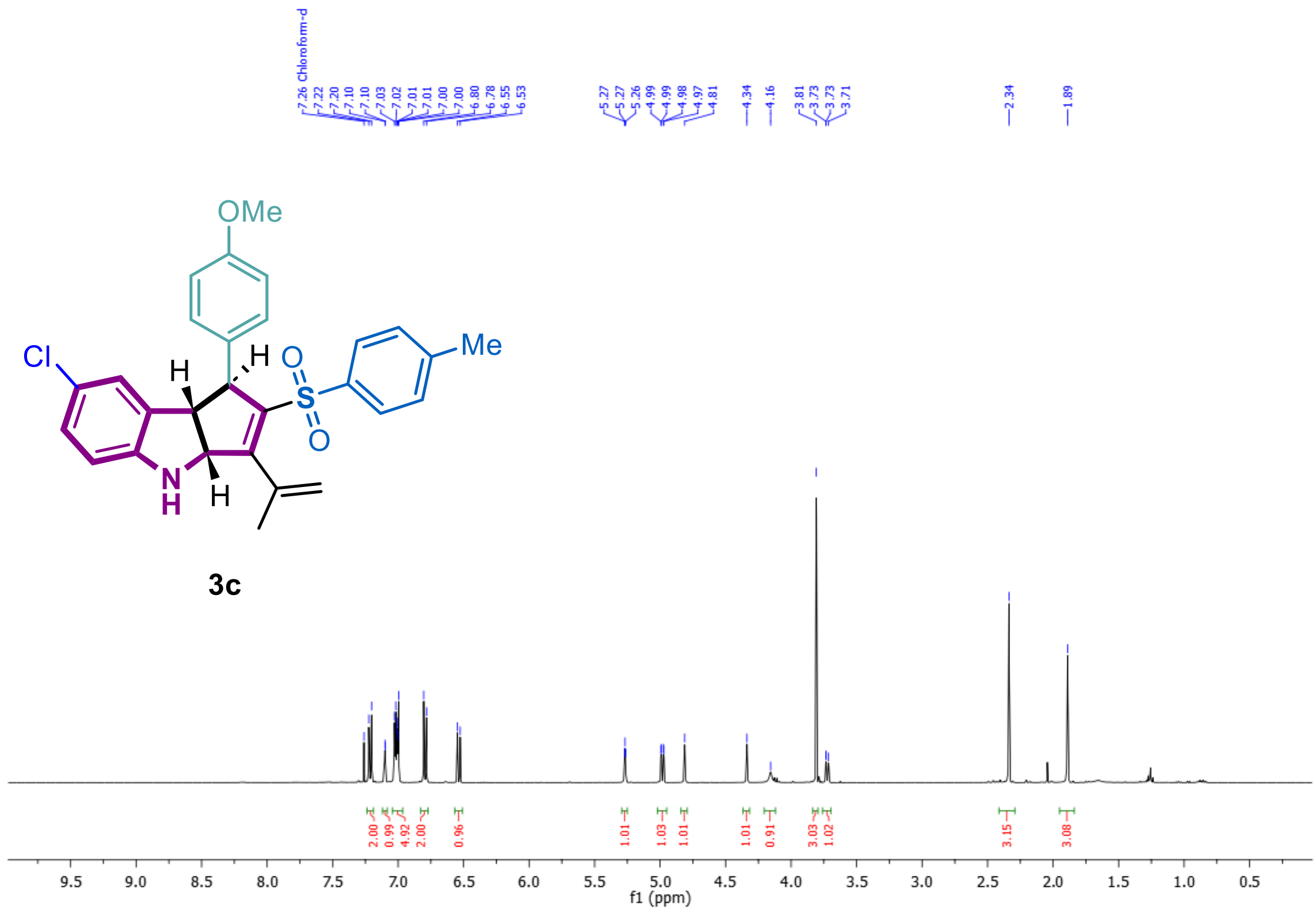

Figure S57: ^{1}H-NMR of **3c** in $CDCl_3$ (400 MHz).

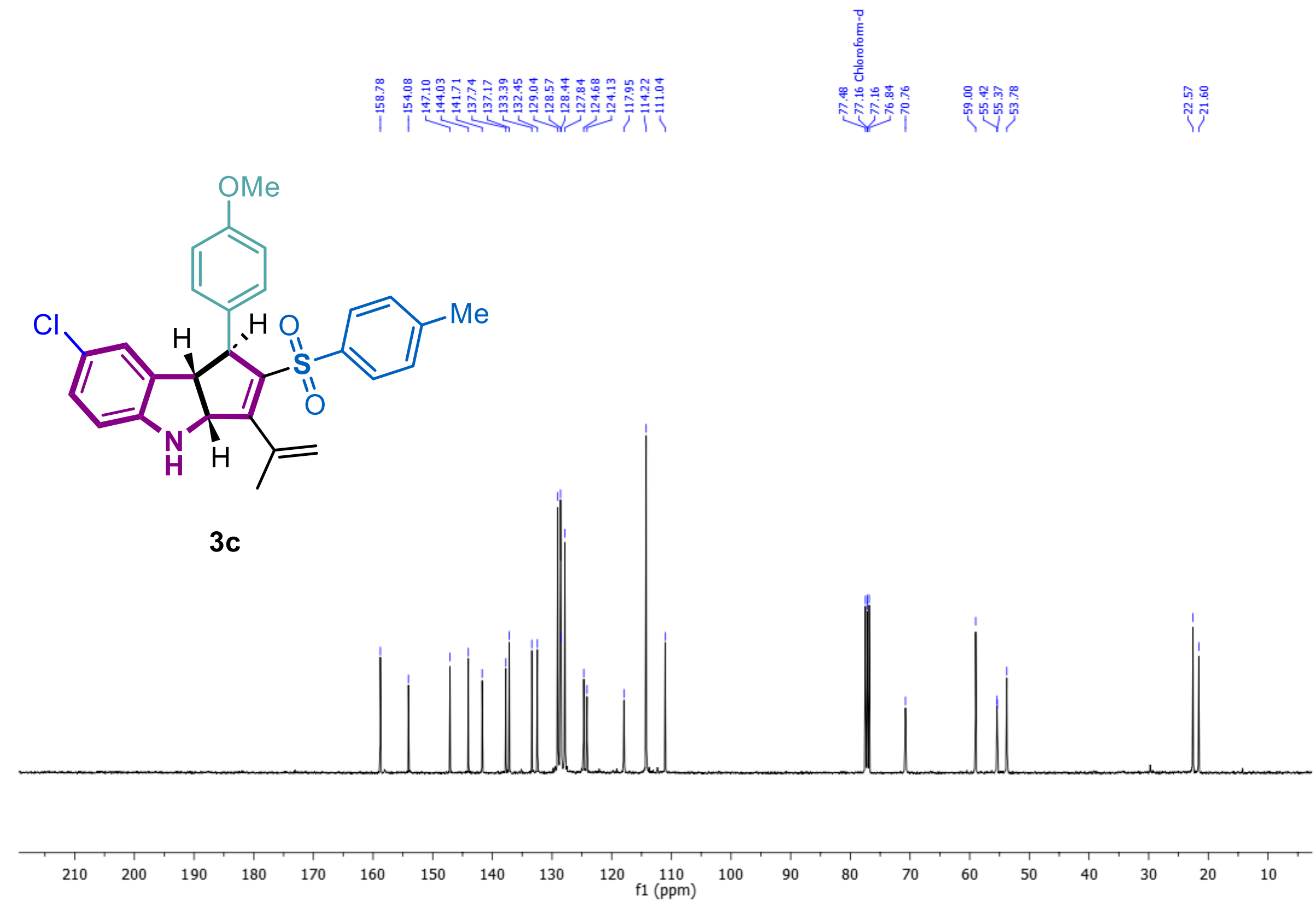

Figure S58: ^{13}C-NMR of **3c** in $CDCl_3$ (101 MHz)

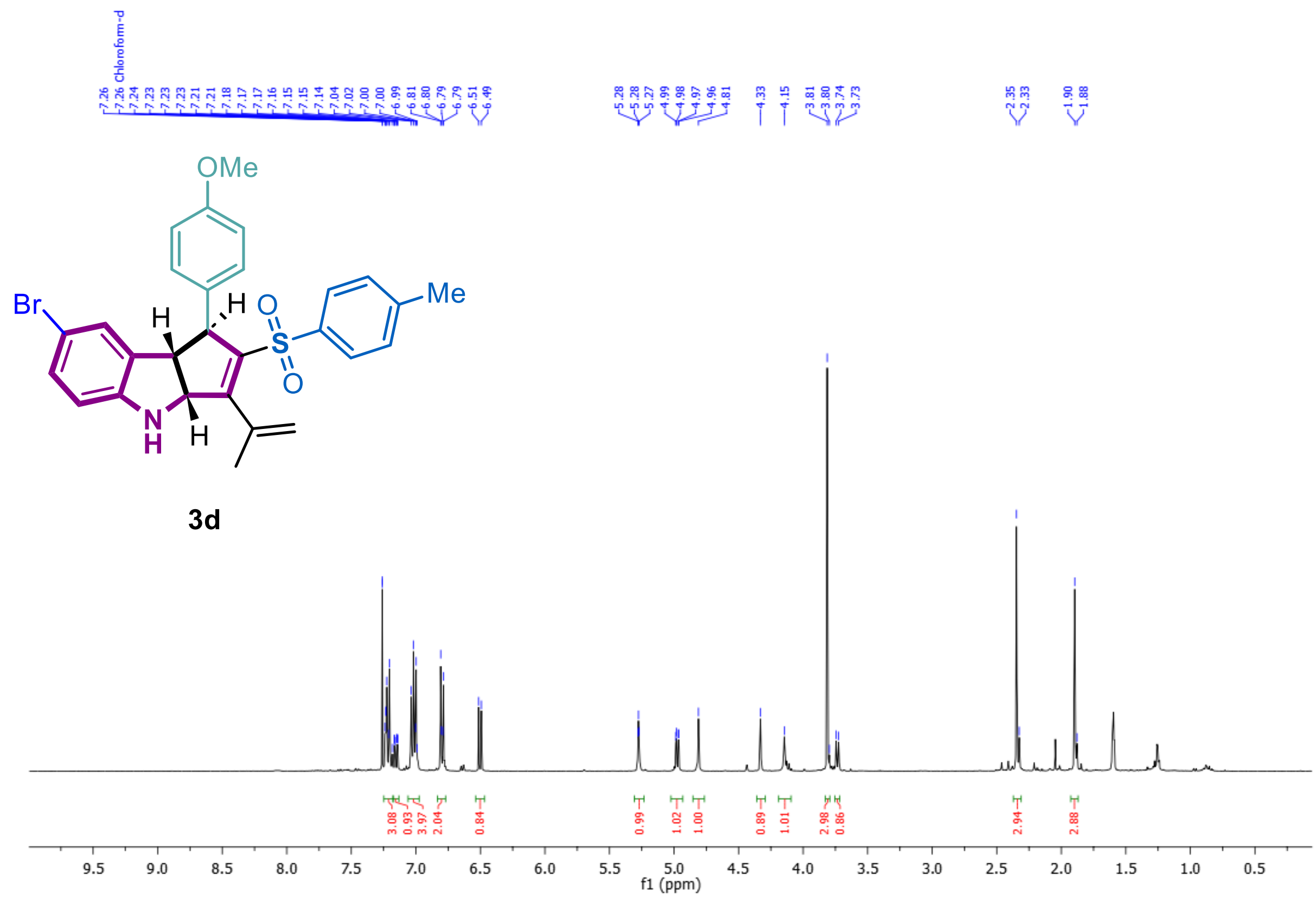

Figure S59: ^{1}H-NMR of **3d** in $CDCl_3$ (400 MHz).

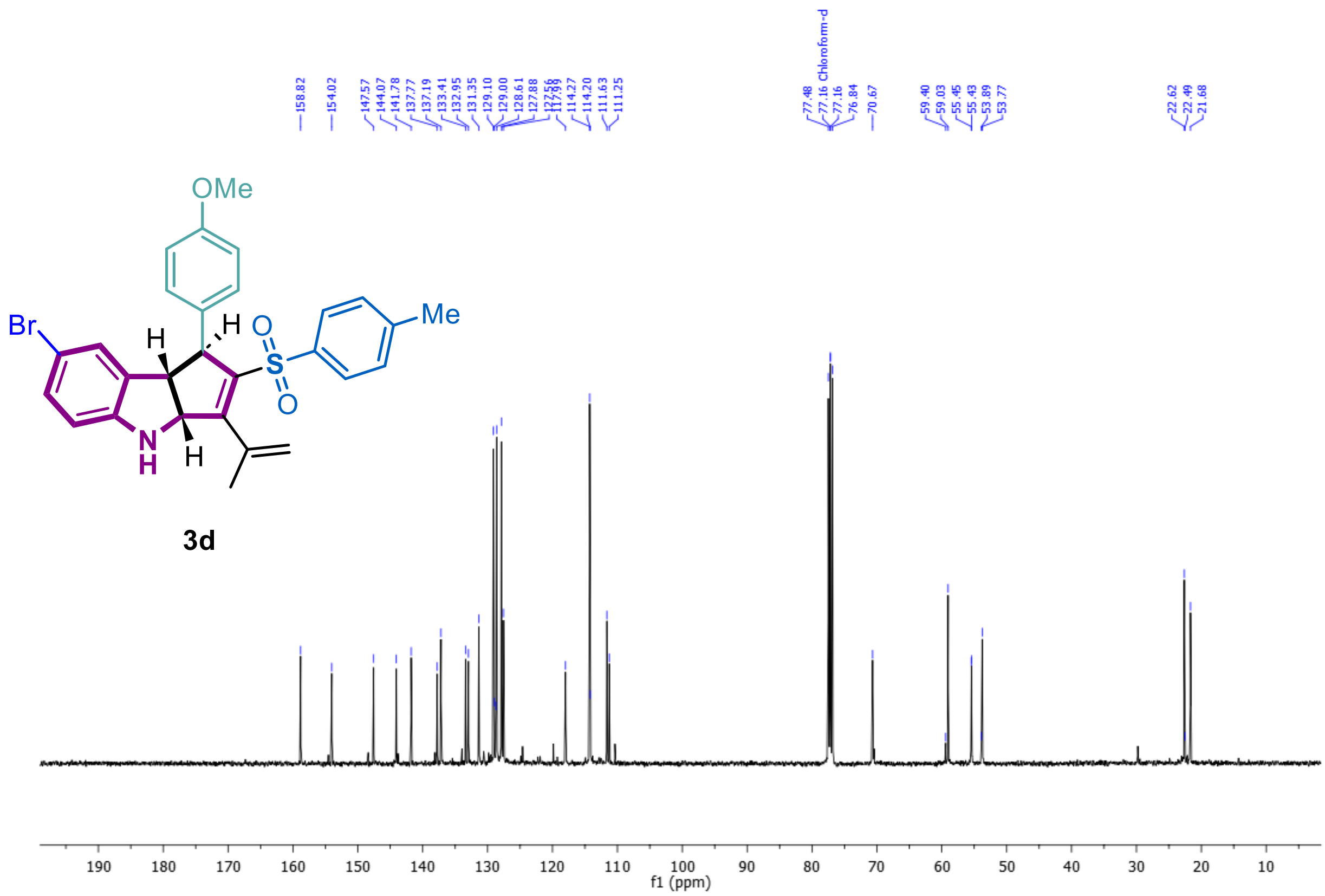

Figure S60: ^{13}C-NMR of **3d** in $CDCl_3$ (101 MHz)

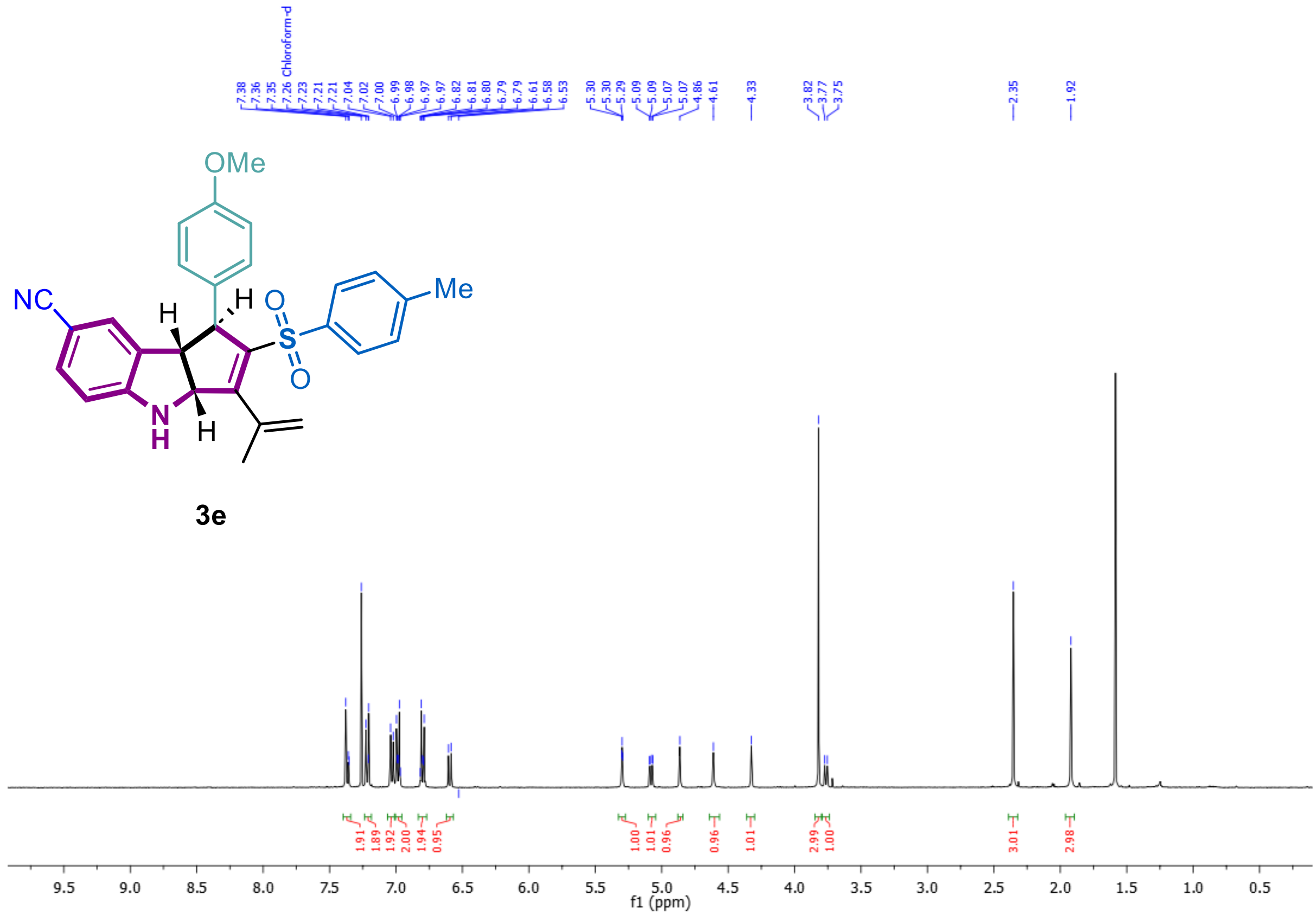

Figure S61: ^{1}H-NMR of **3e** in $CDCl_3$ (400 MHz)

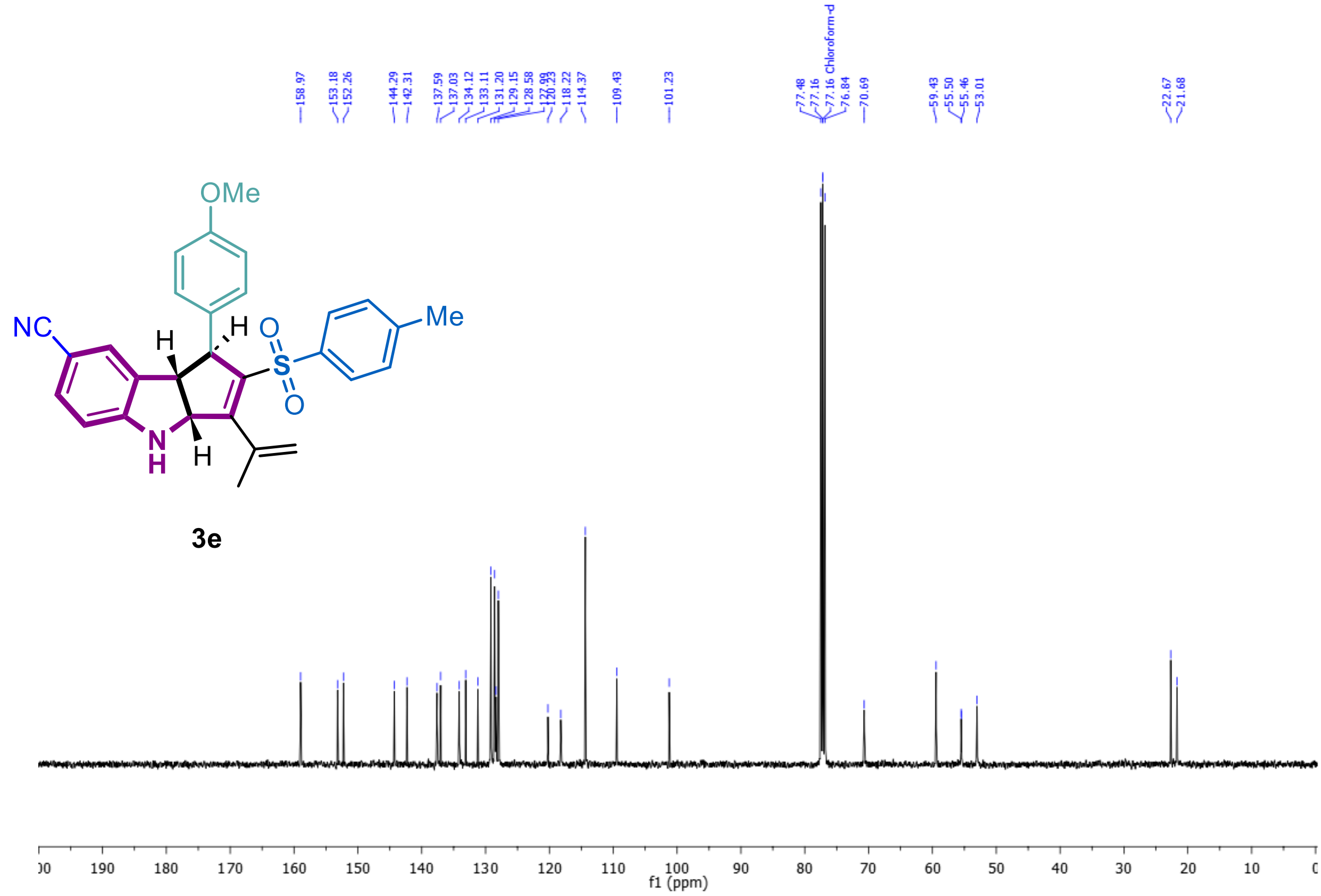

Figure S62: *^{13}C-NMR of **3e** in CDCl₃ (101 MHz)*

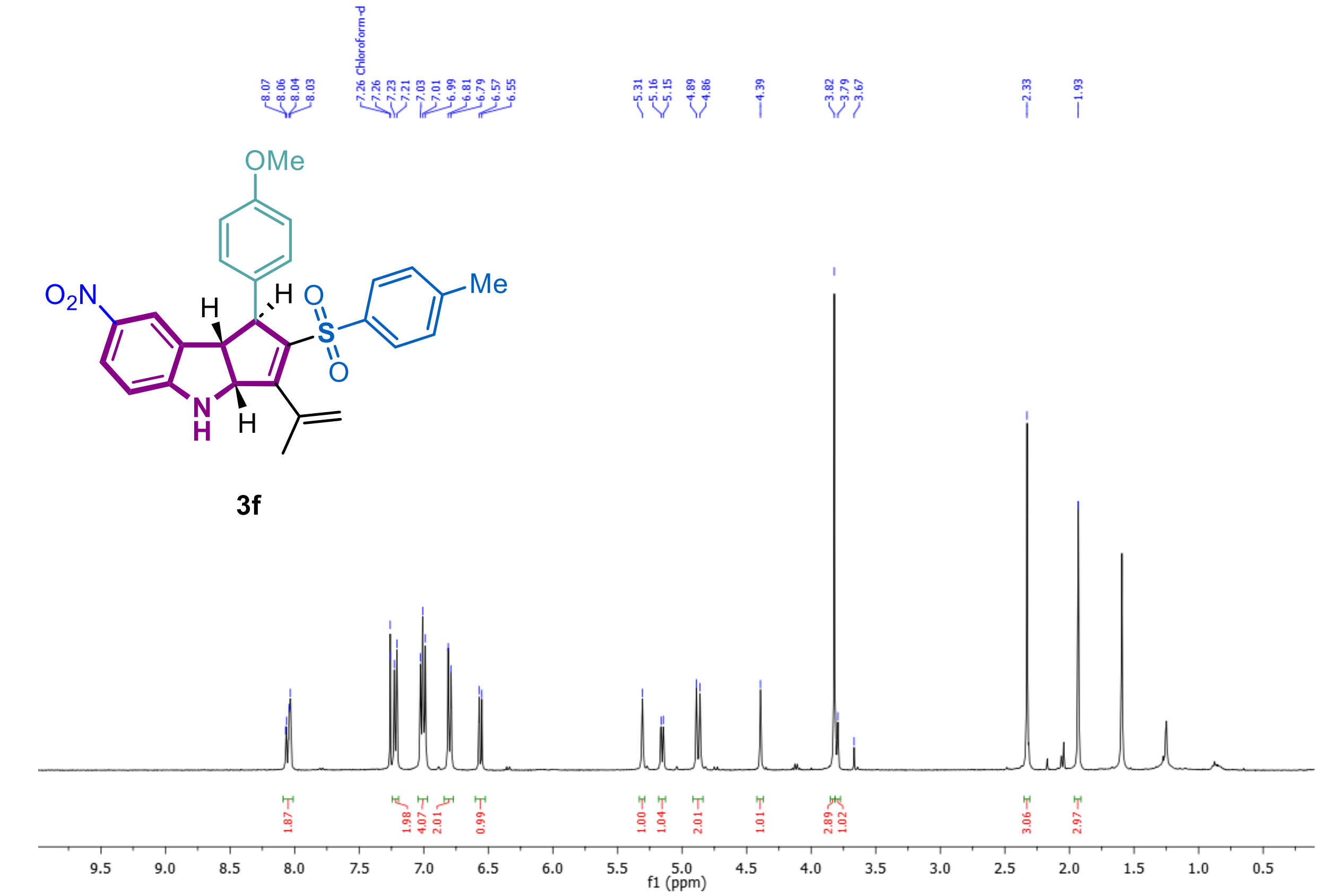

Figure S63: *^{1}H-NMR of **3f** in CDCl₃ (400 MHz).*

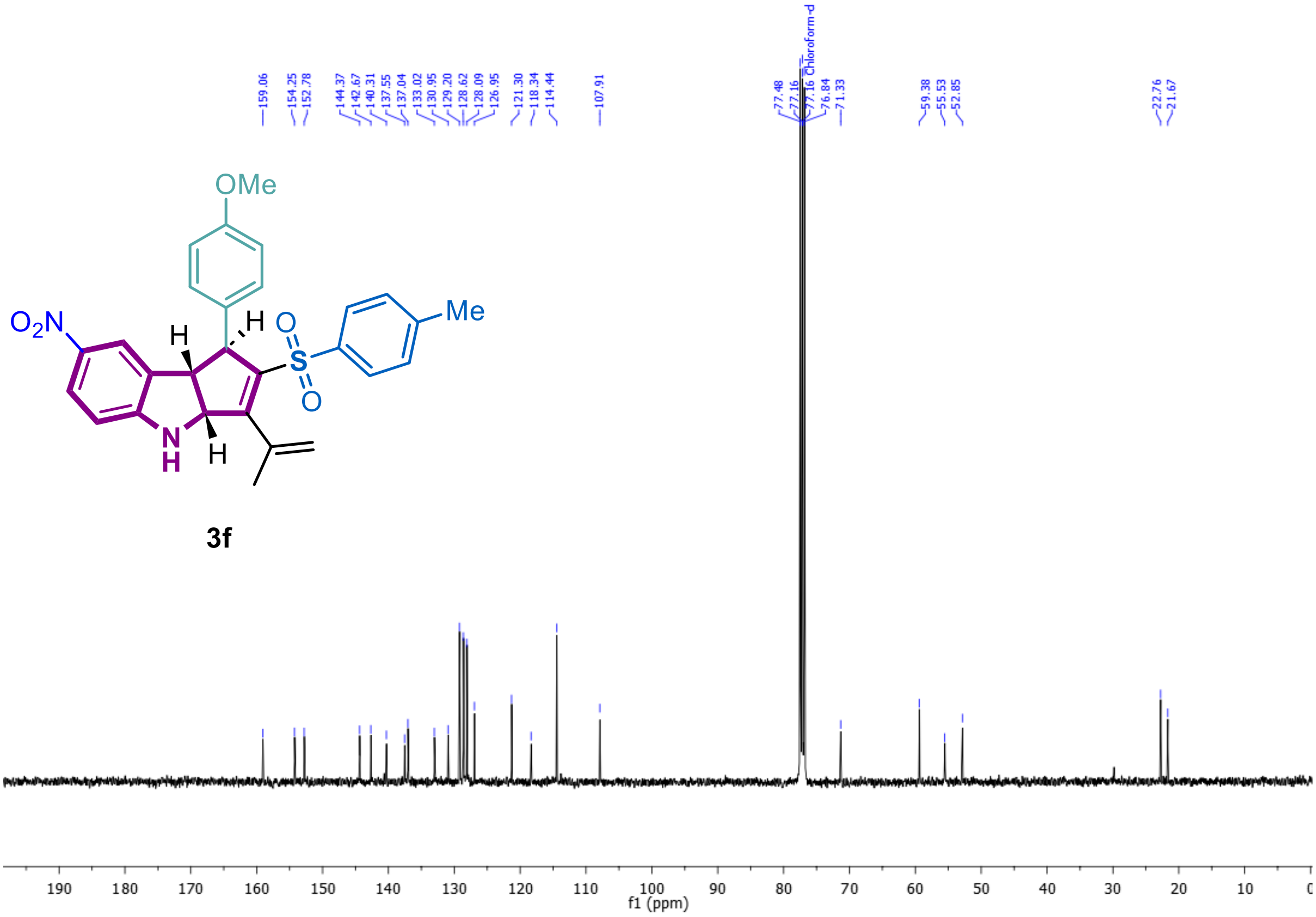

Figure S64: ^{13}C-NMR of ***3f*** in $CDCl_3$ (101 MHz).

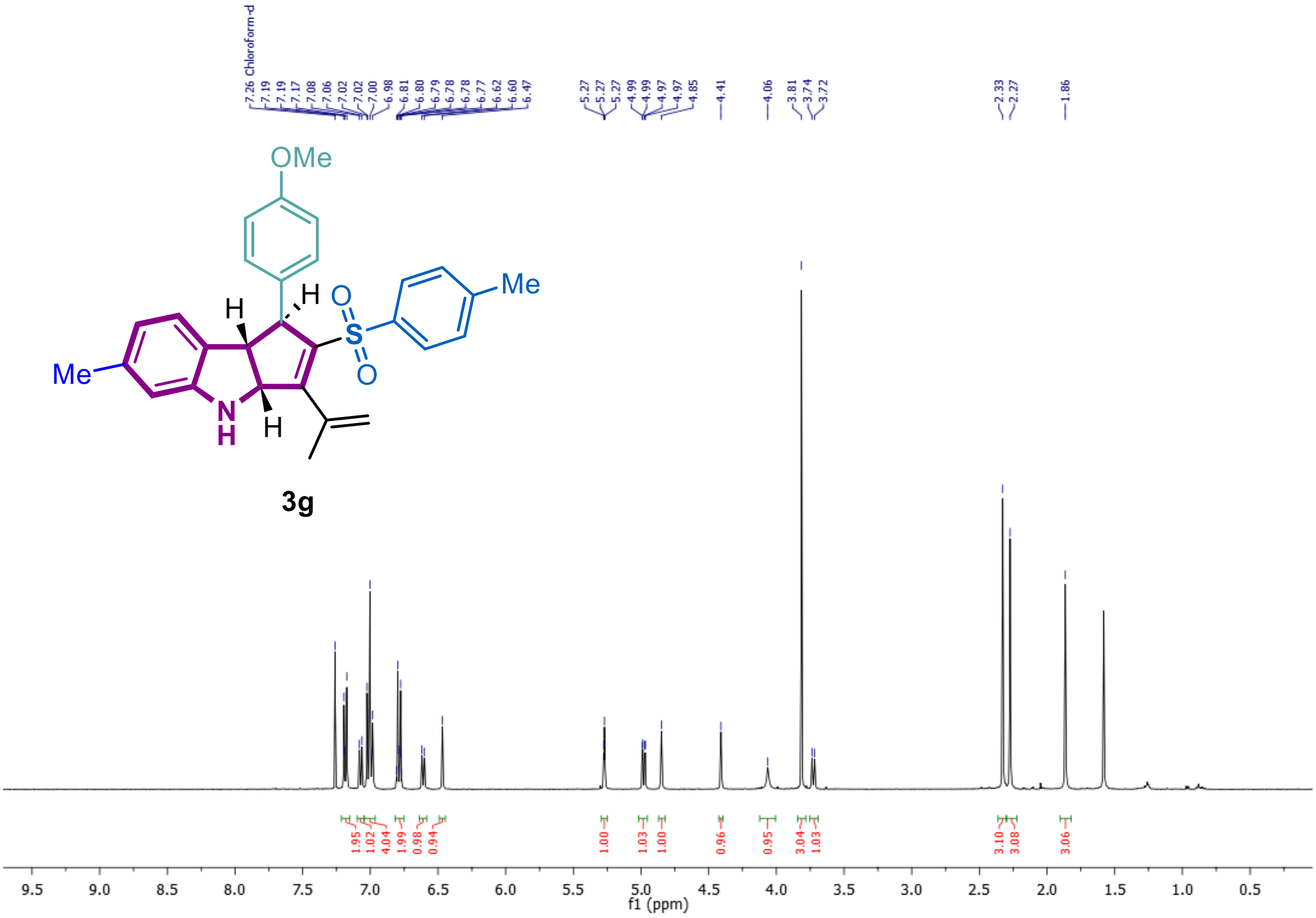

Figure S65: ^{1}H-NMR of ***3g*** in $CDCl_3$ (400 MHz).

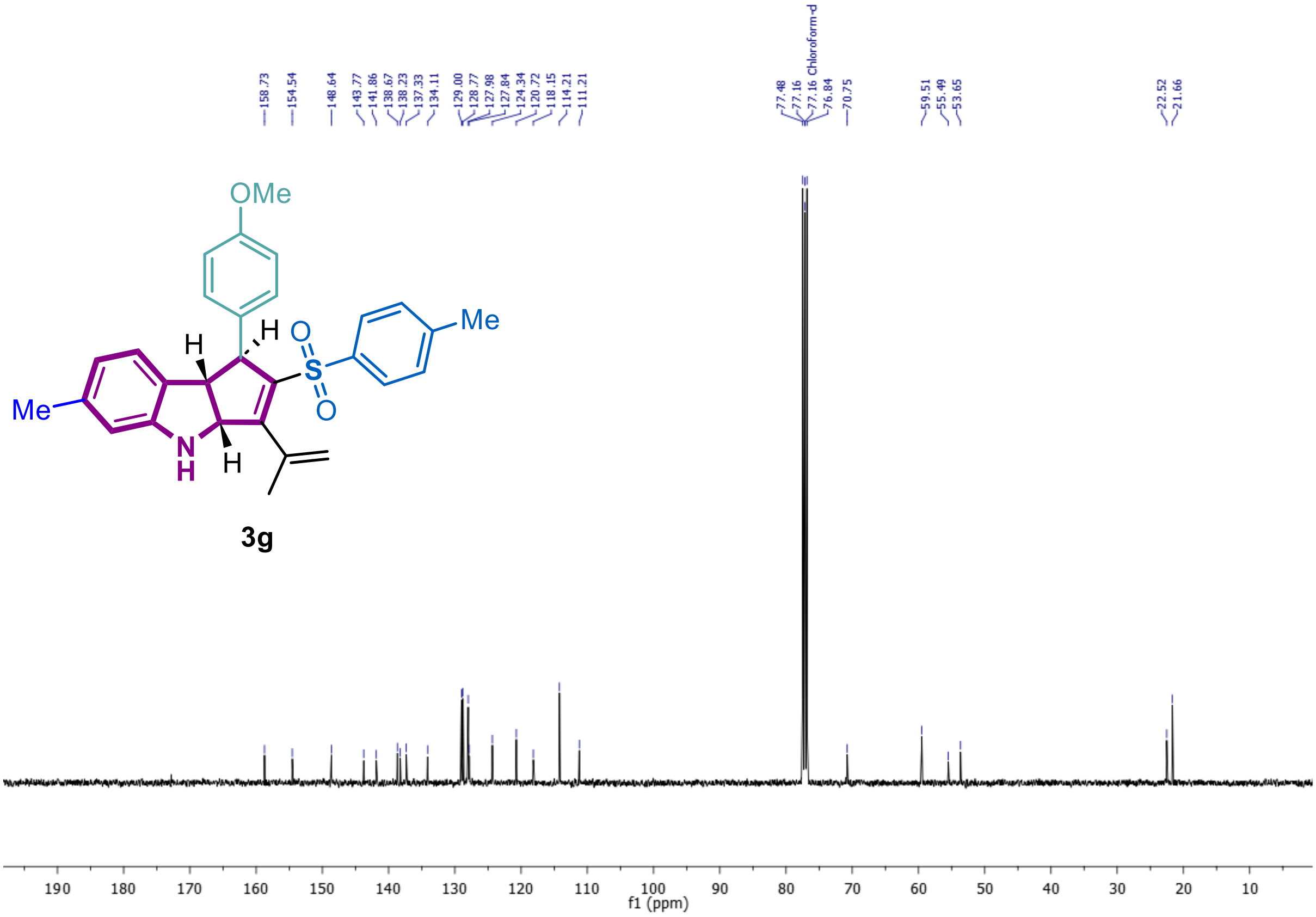

Figure S66: *^{13}C-NMR of **3g** in CDCl₃ (101 MHz)*

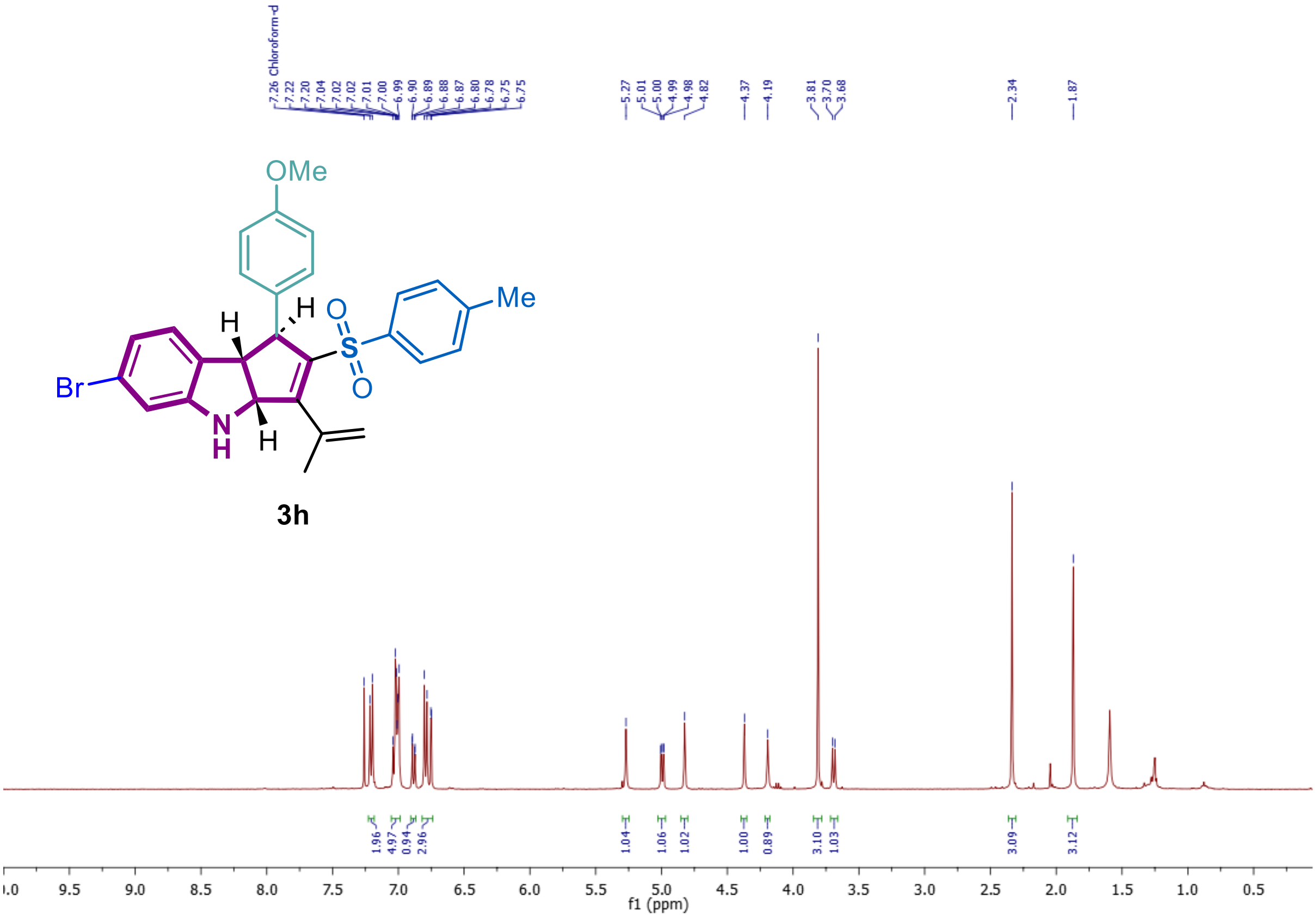

Figure S67: *^{1}H-NMR of **3h** in $CDCl_3$ (400 MHz).*

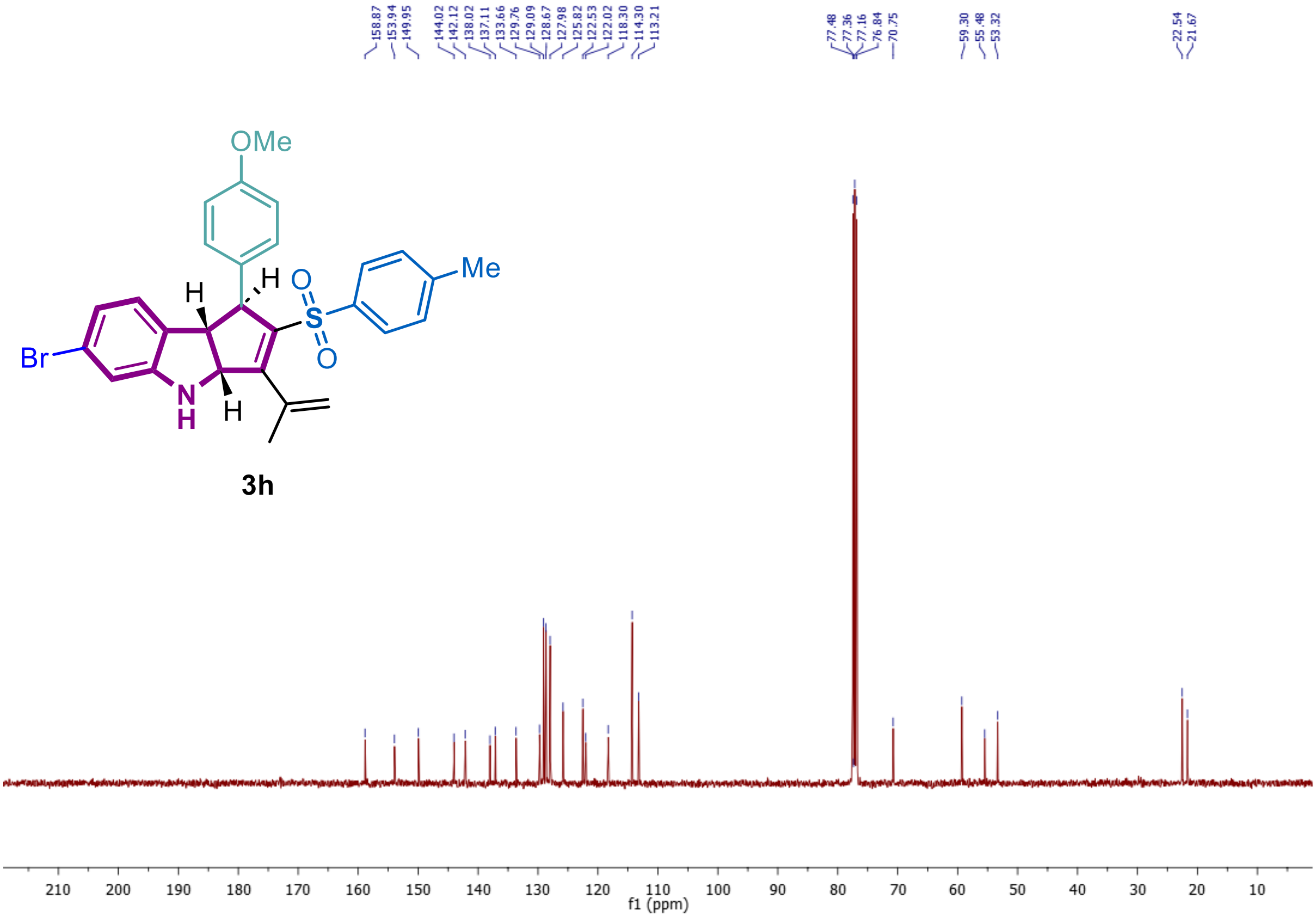

Figure S68: *^{13}C-NMR of **3h** in $CDCl_3$ (101 MHz)*

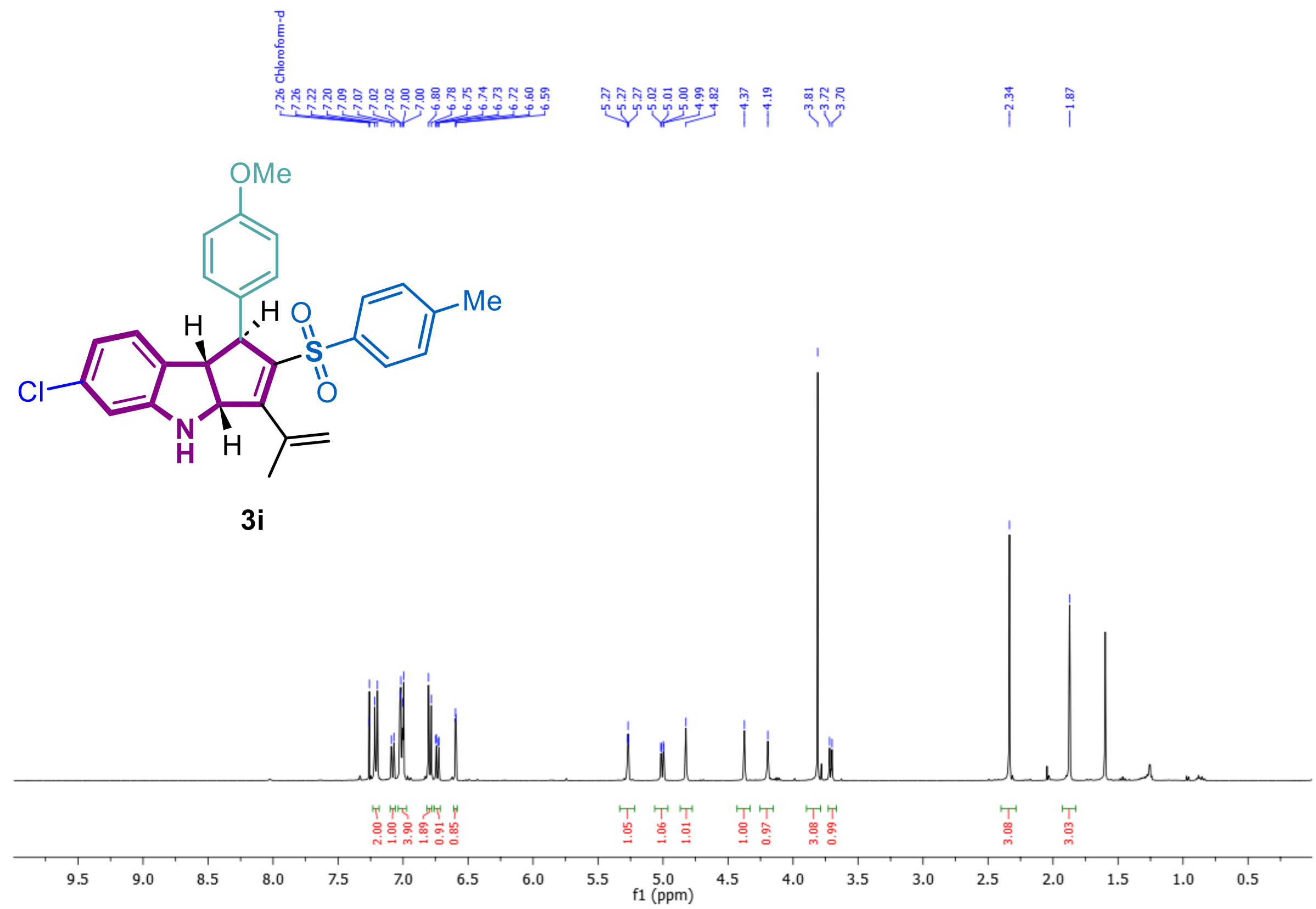

Figure S69: *^{1}H-NMR of **3i** in $CDCl_3$ (400 MHz).*

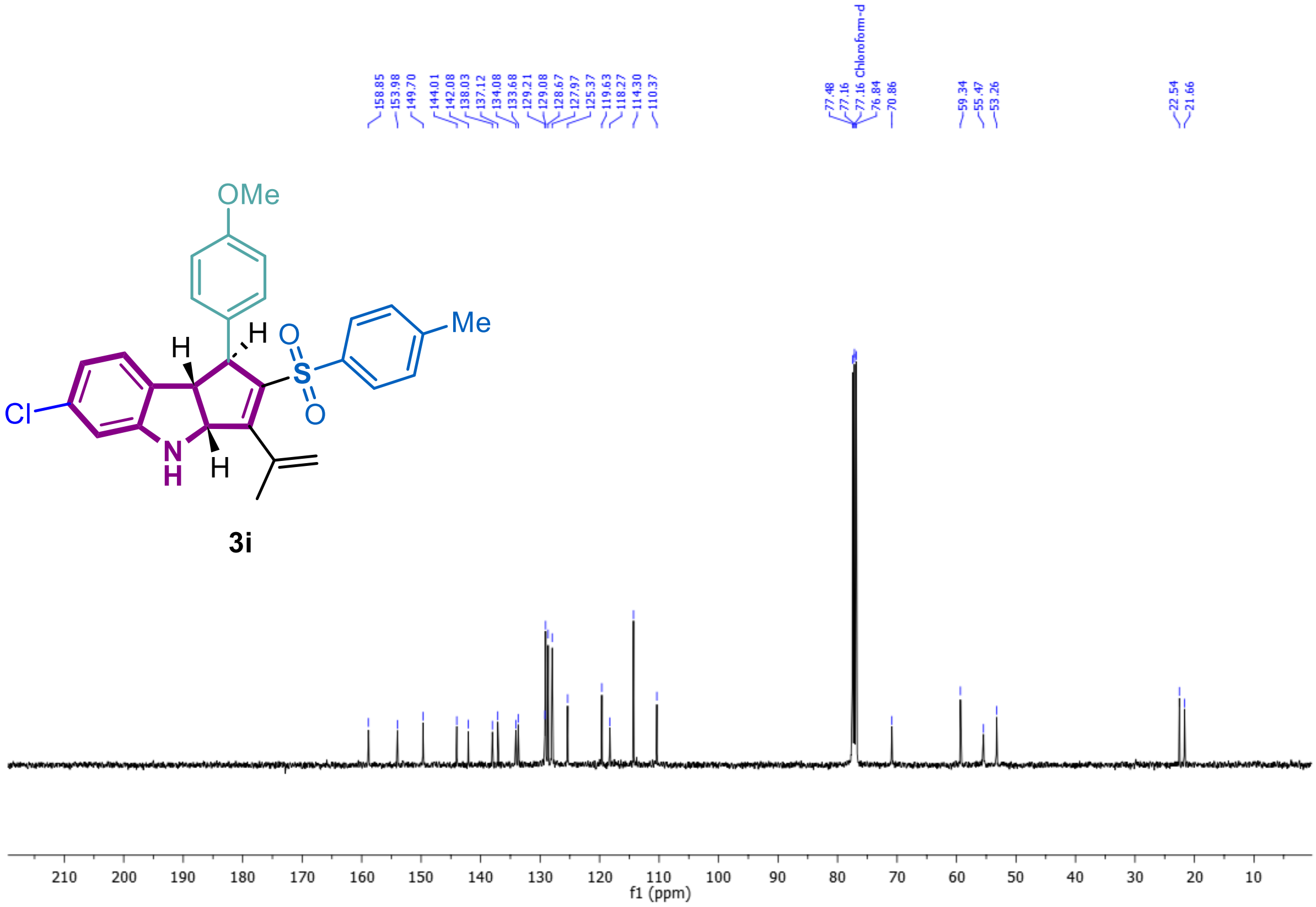

3i

Figure S70: *^{13}C-NMR of **3i** in $CDCl_3$ (101 MHz)*

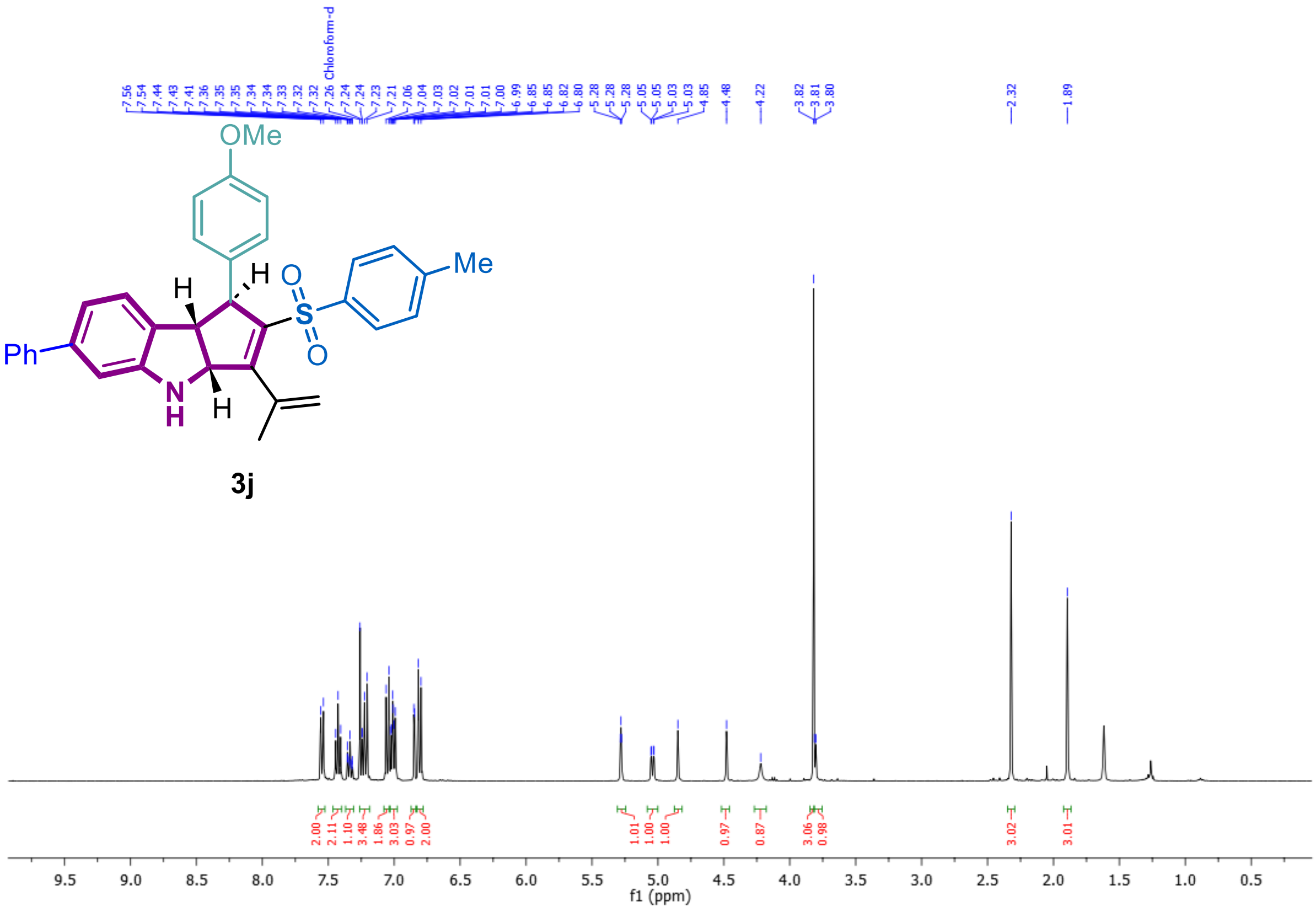

3j

Figure S71: *^{1}H-NMR of **3j** in $CDCl_3$ (400 MHz).*

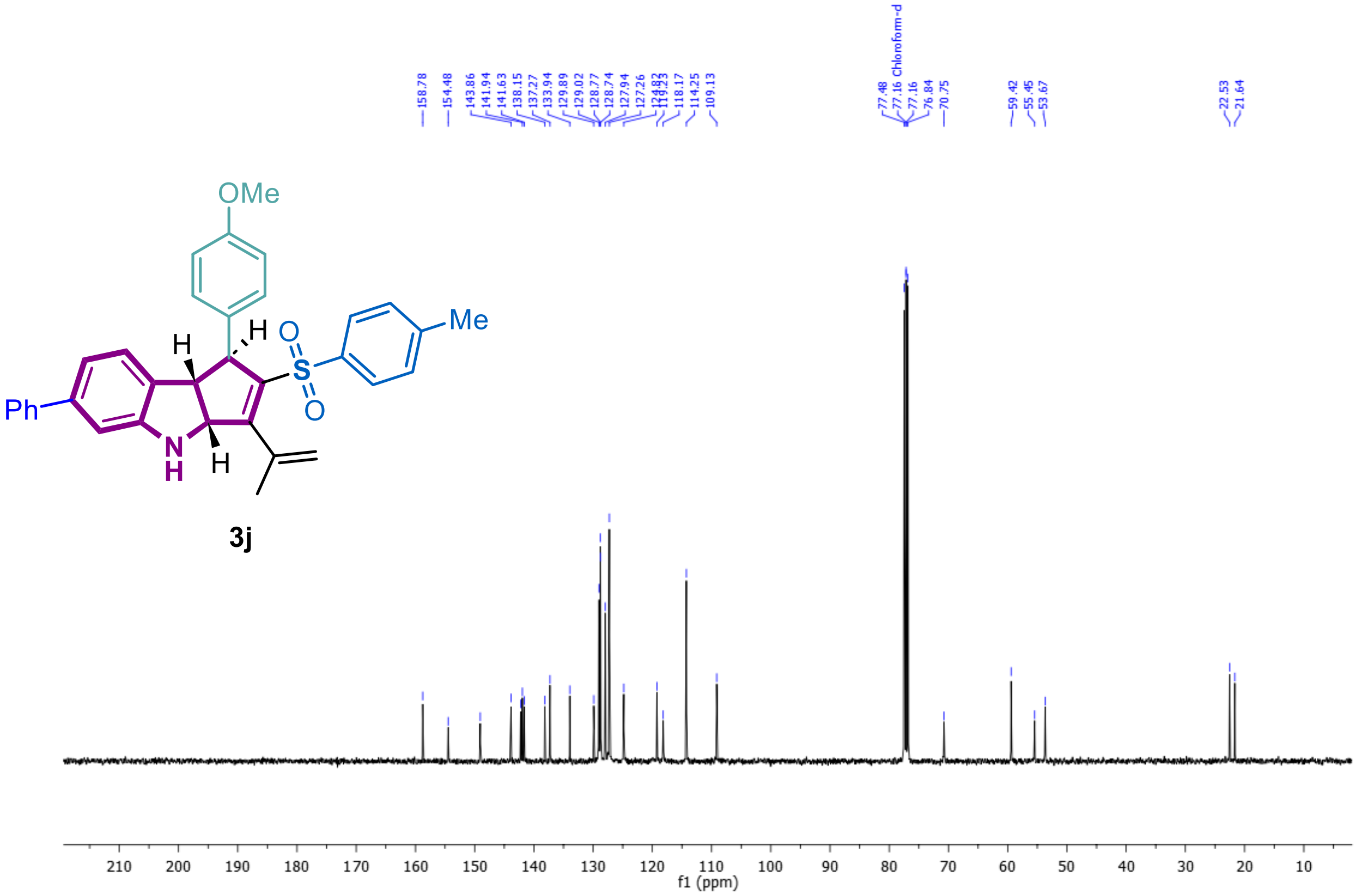

3j

Figure S72: ^{13}C-NMR of **3j** in $CDCl_3$ (101 MHz)

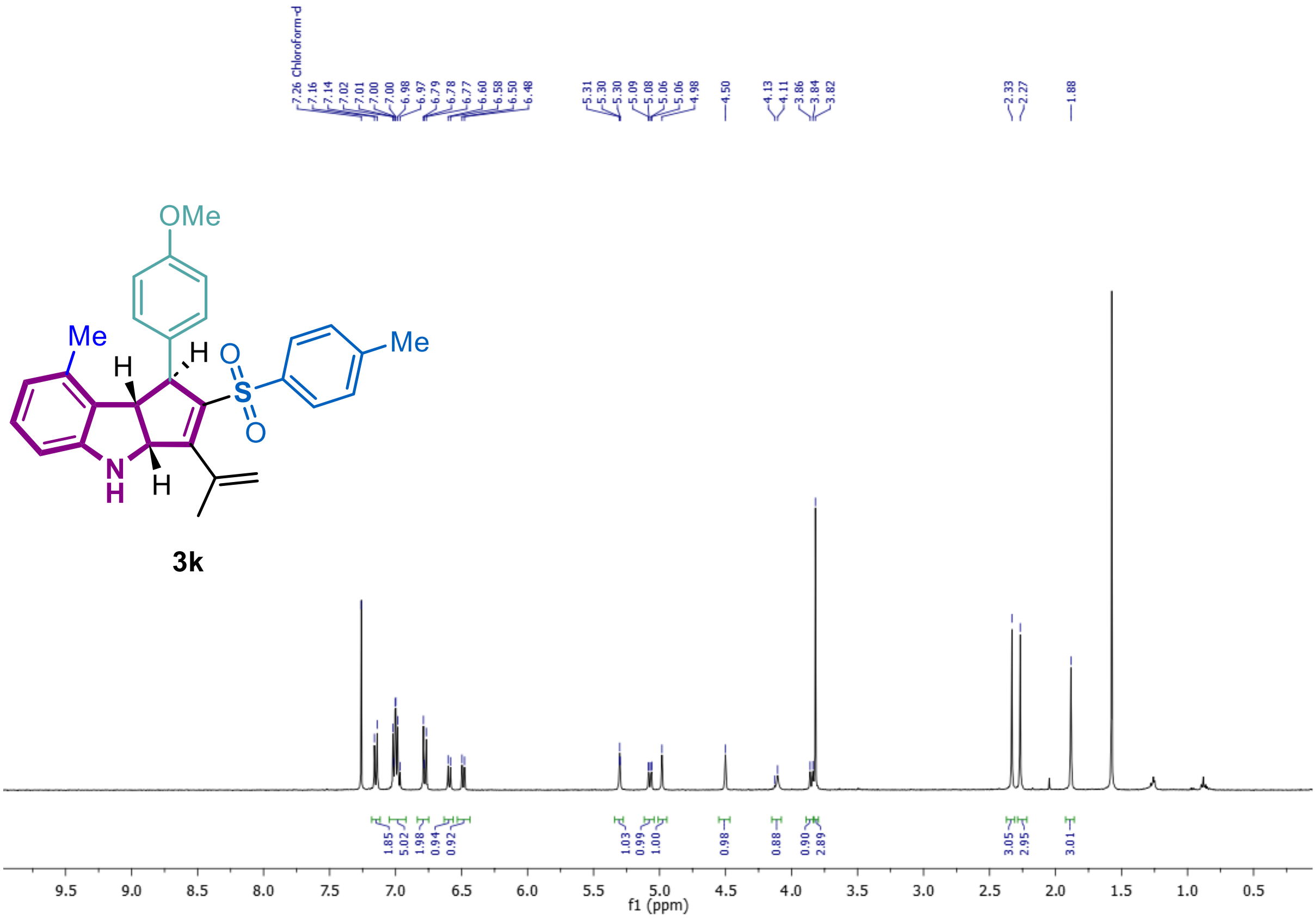

3k

Figure S73: ^{1}H-NMR of **3k** in $CDCl_3$ (400 MHz).

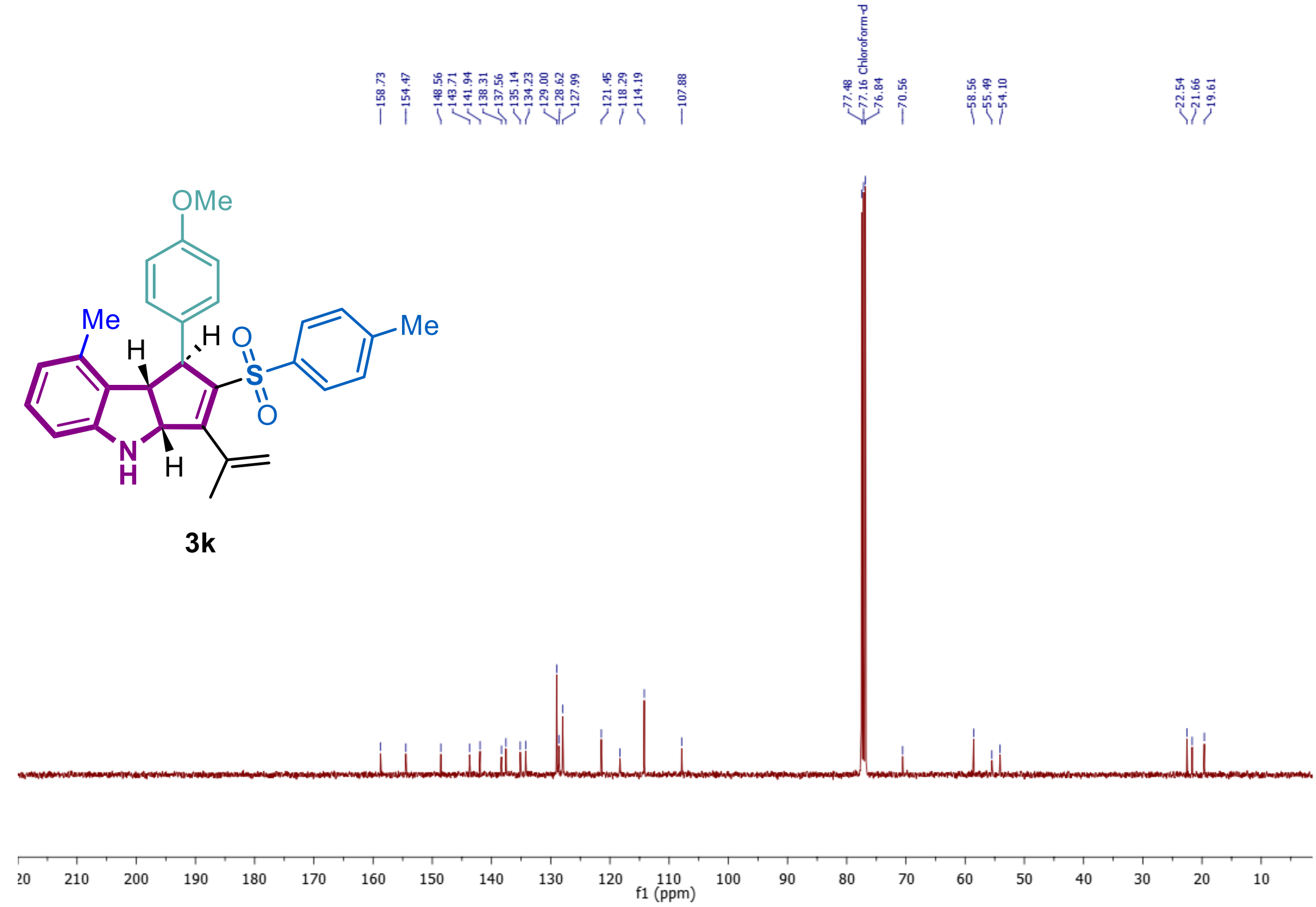

Figure S74: ^{13}C-NMR of **3k** in $CDCl_3$ (101 MHz)

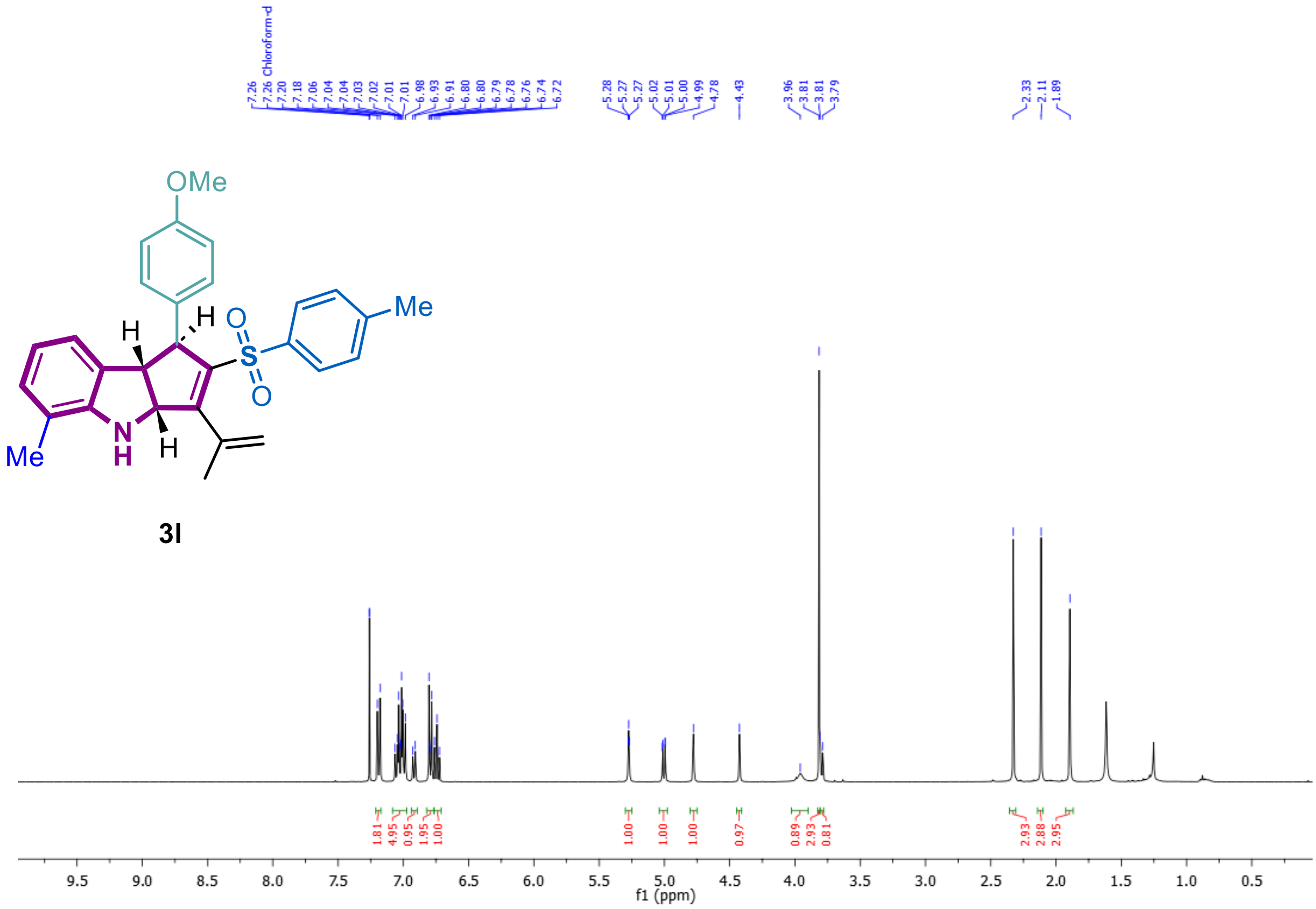

Figure S75: ^{1}H-NMR of **3l** in $CDCl_3$ (400 MHz)

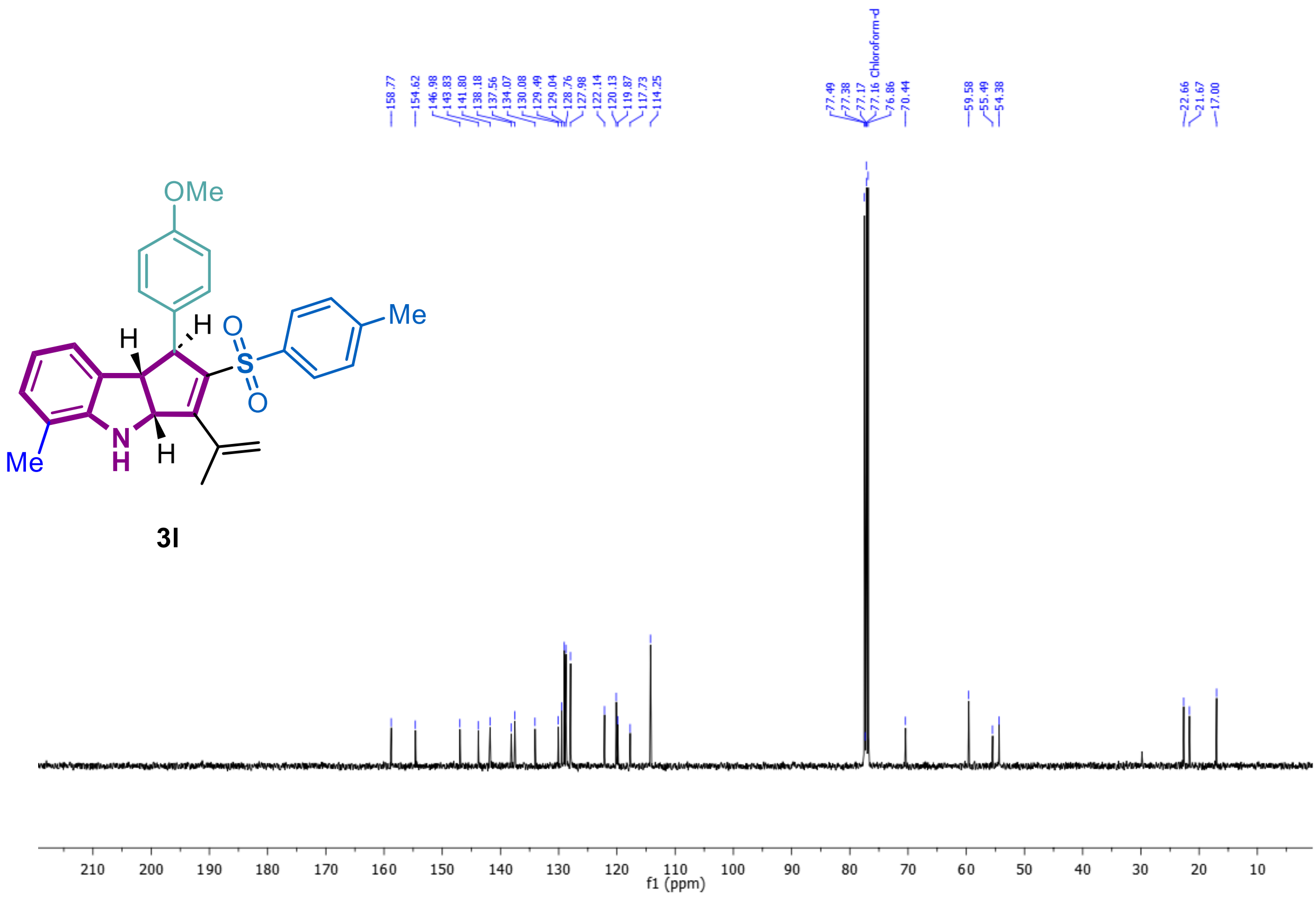

Figure S76: ^{13}C-NMR of **3l** in $CDCl_3$ (101 MHz)

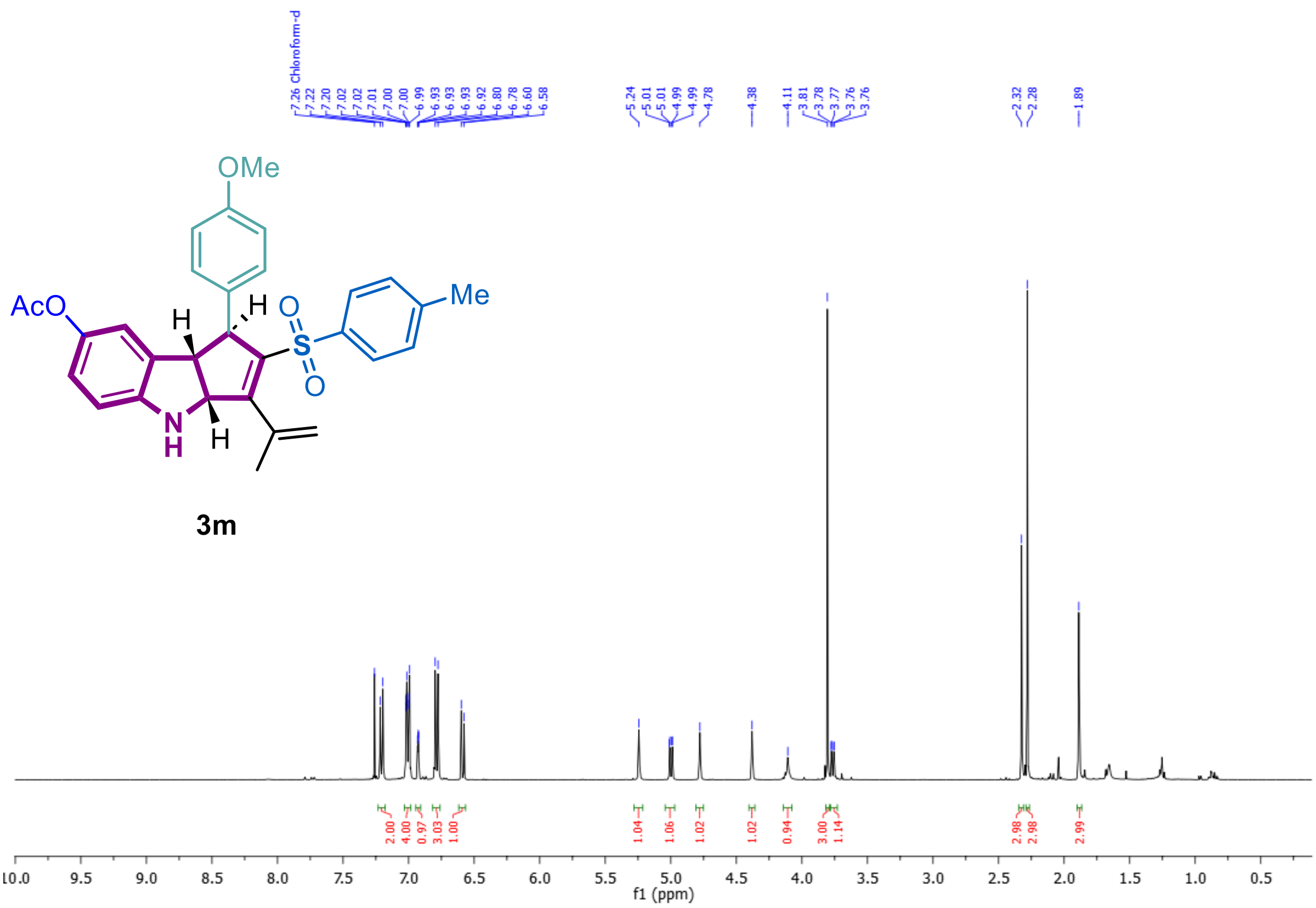

Figure S77: ^{1}H-NMR of **3m** in $CDCl_3$ (400 MHz)

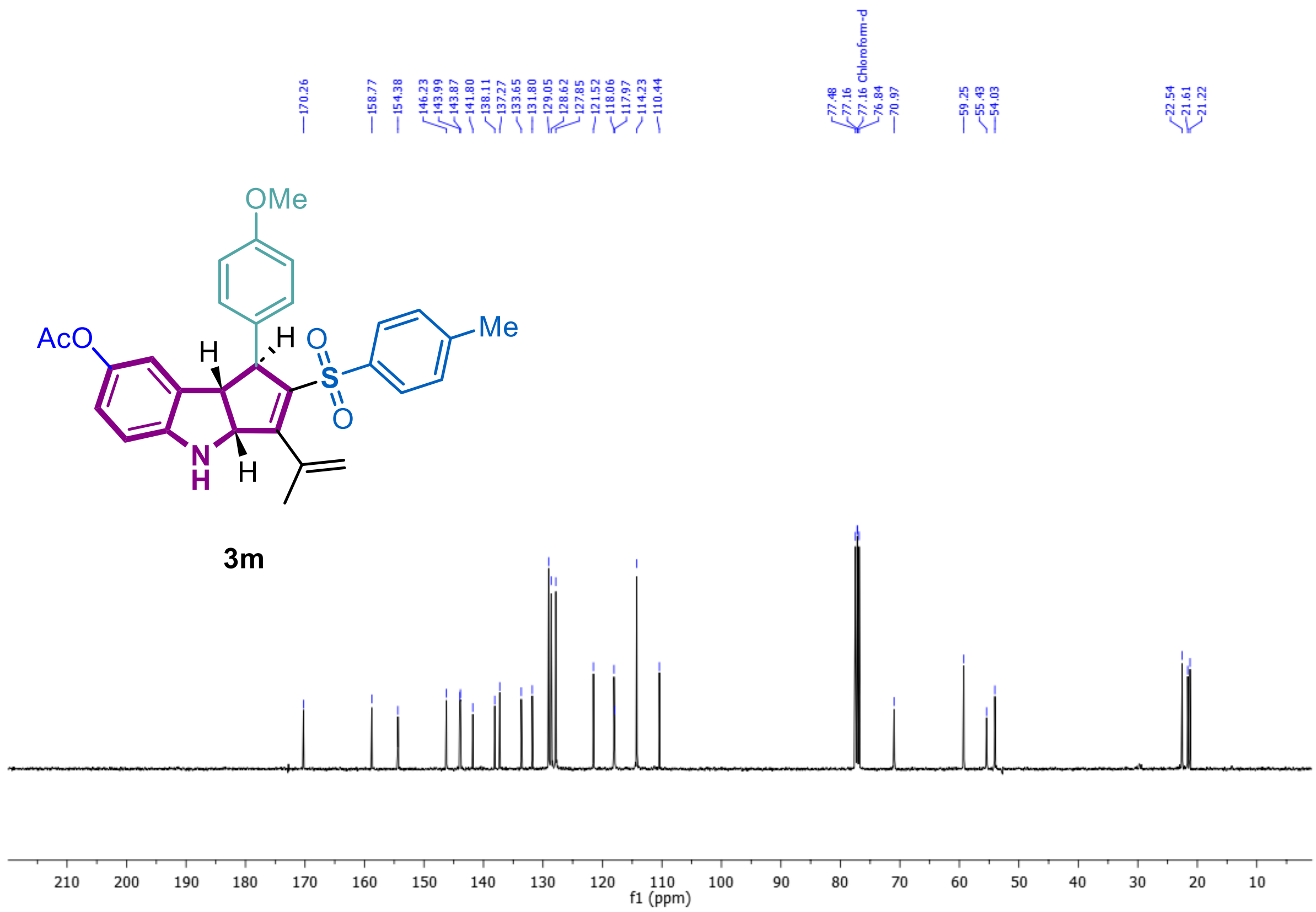

Figure S78: ^{13}C-NMR of **3m** in $CDCl_3$ (101 MHz)

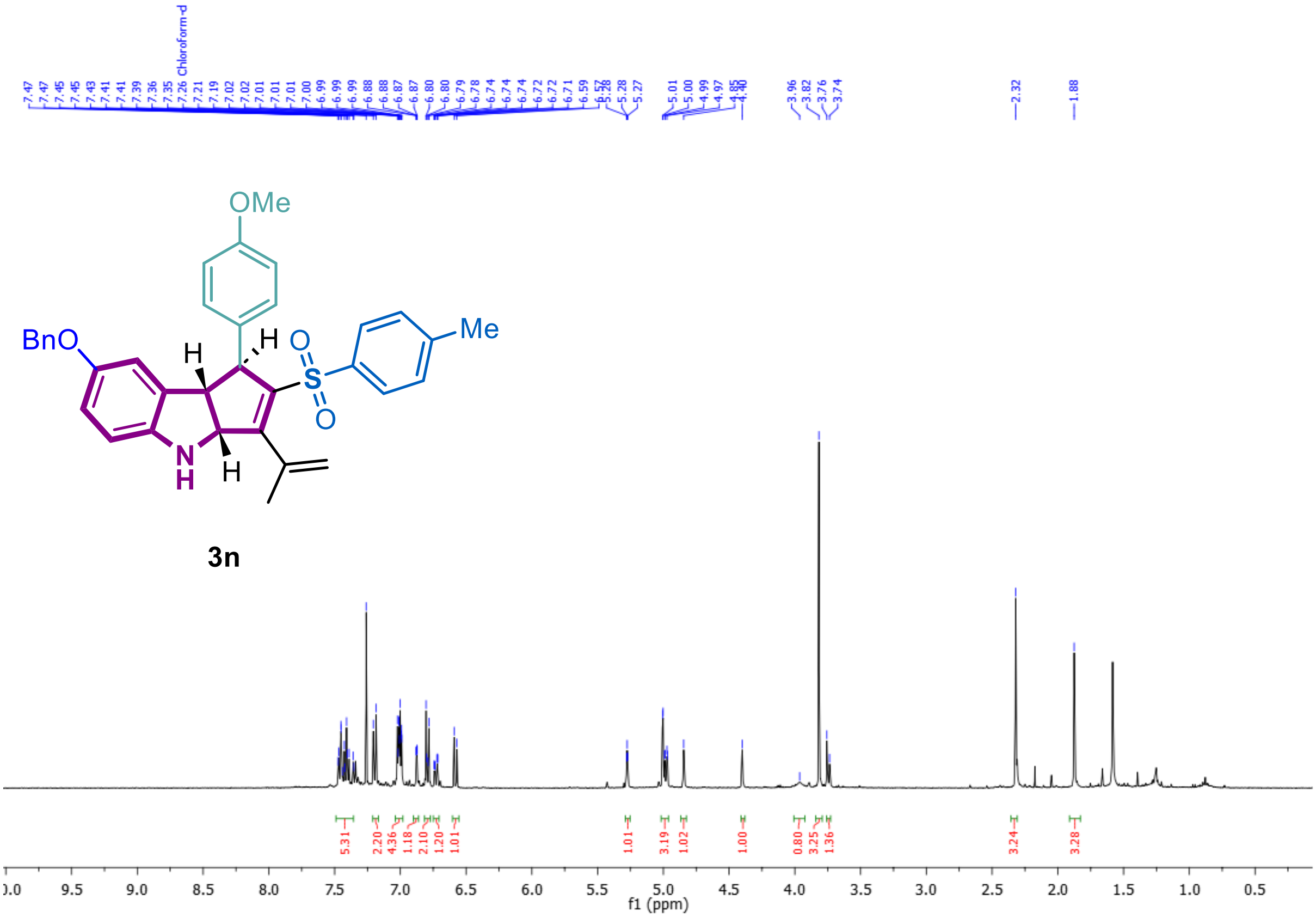

Figure S79: ^{1}H-NMR of **3n** in $CDCl_3$ (400 MHz)

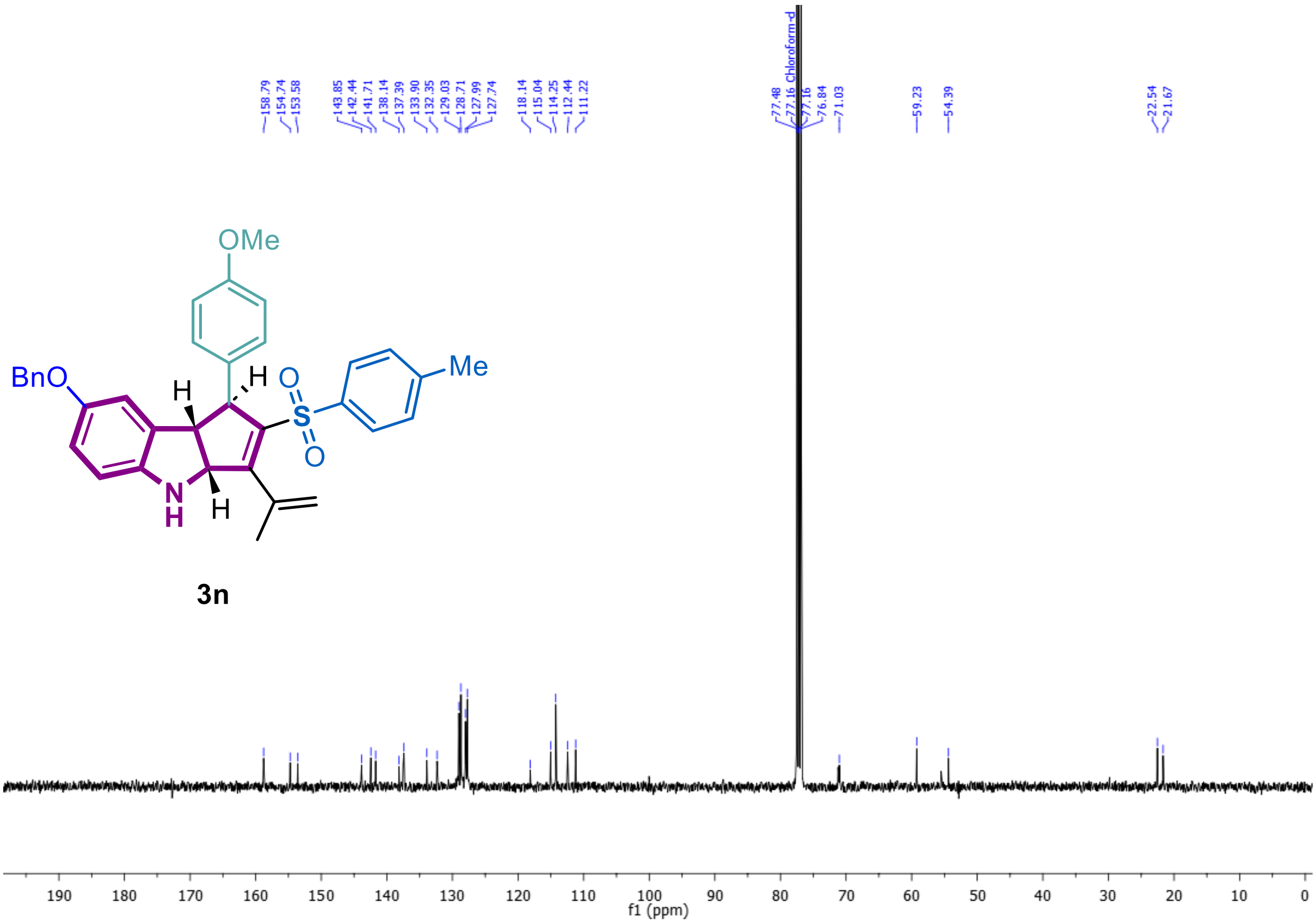

3n

__Figure S80__: ^{13}C-NMR of __3n__ in $CDCl_3$ (101 MHz)

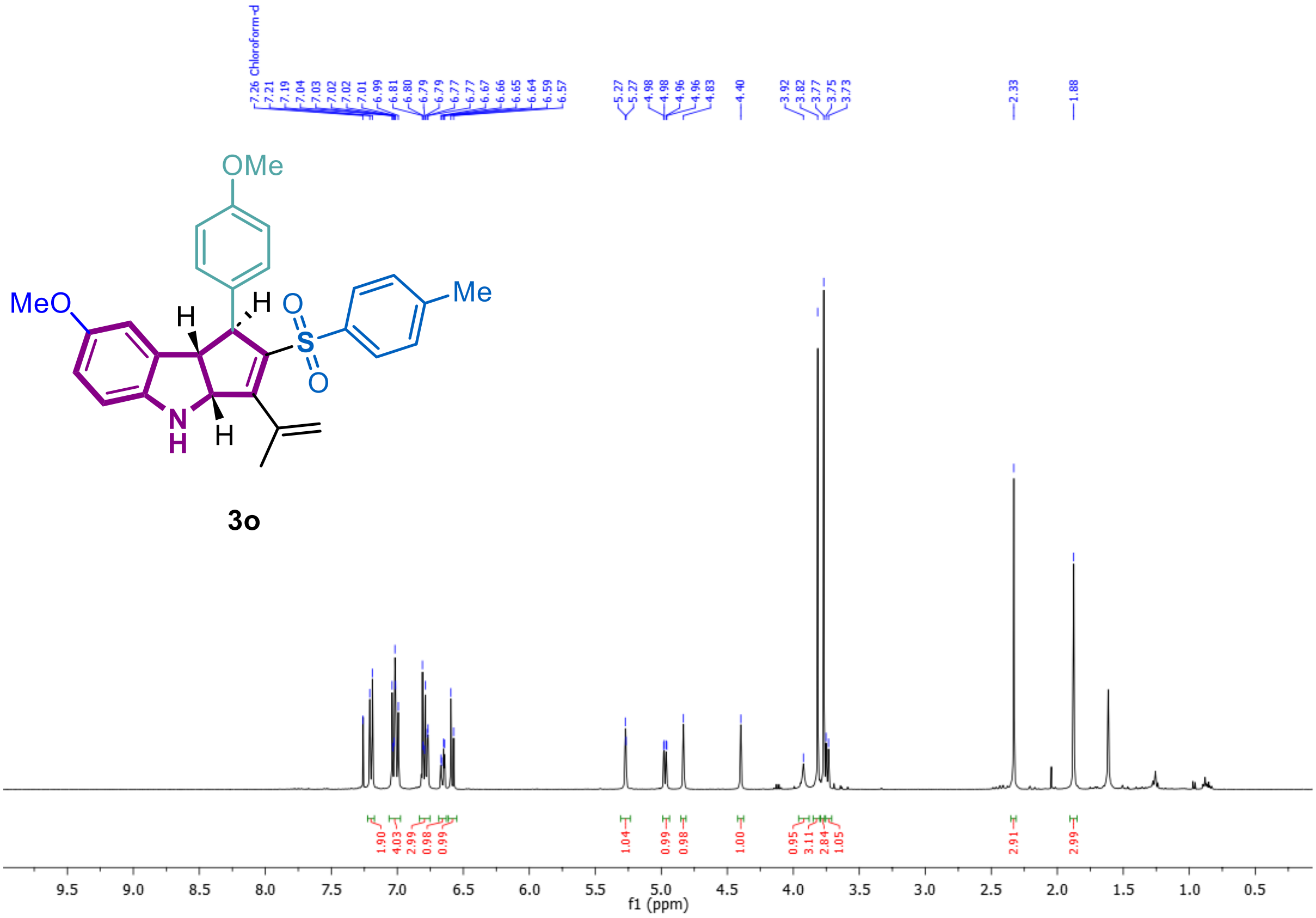

3o

__Figure S81__: ^{1}H-NMR of __3o__ in $CDCl_3$ (400 MHz)

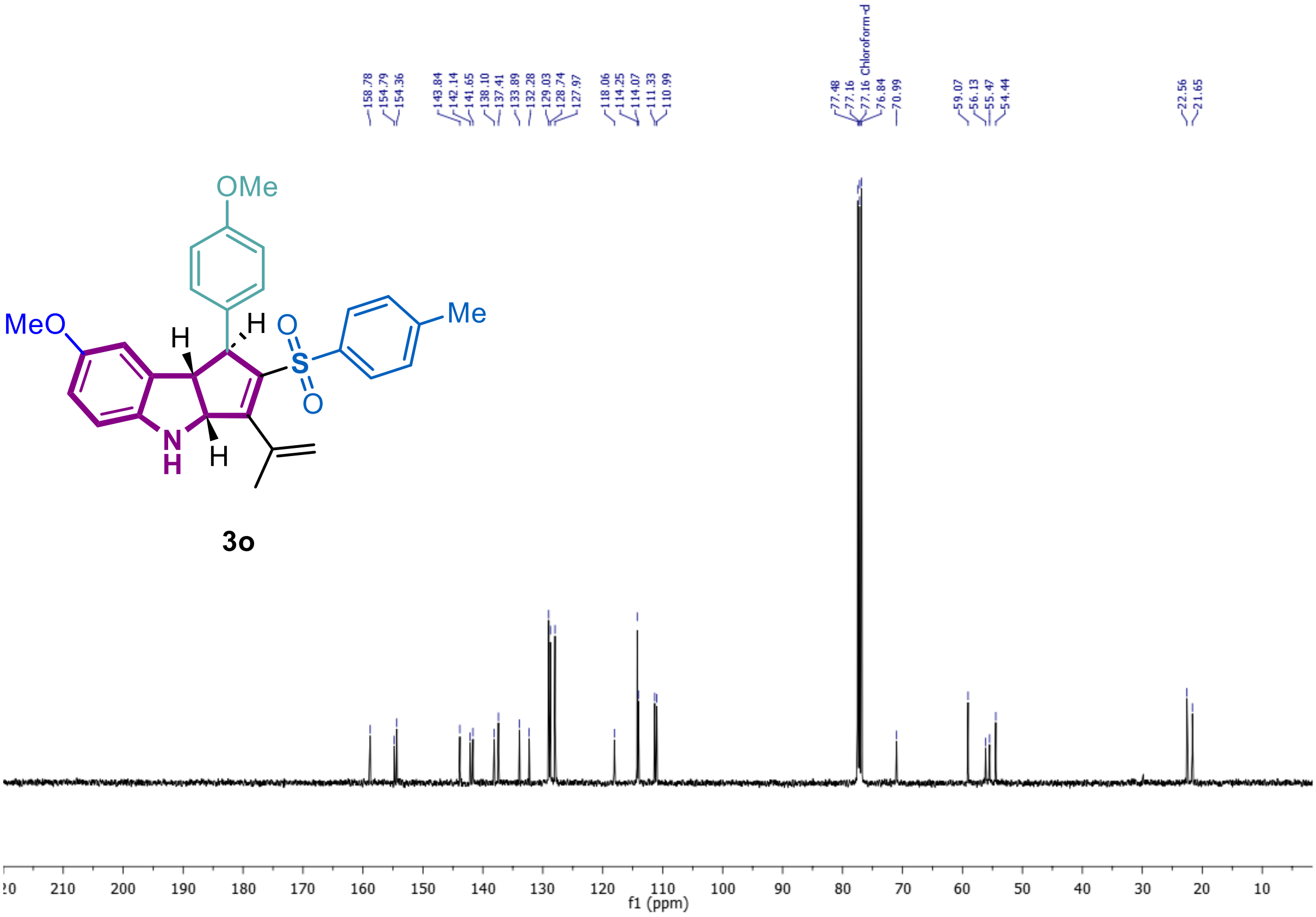

Figure S82: *^{13}C-NMR of **3o** in $CDCl_3$ (101 MHz)*

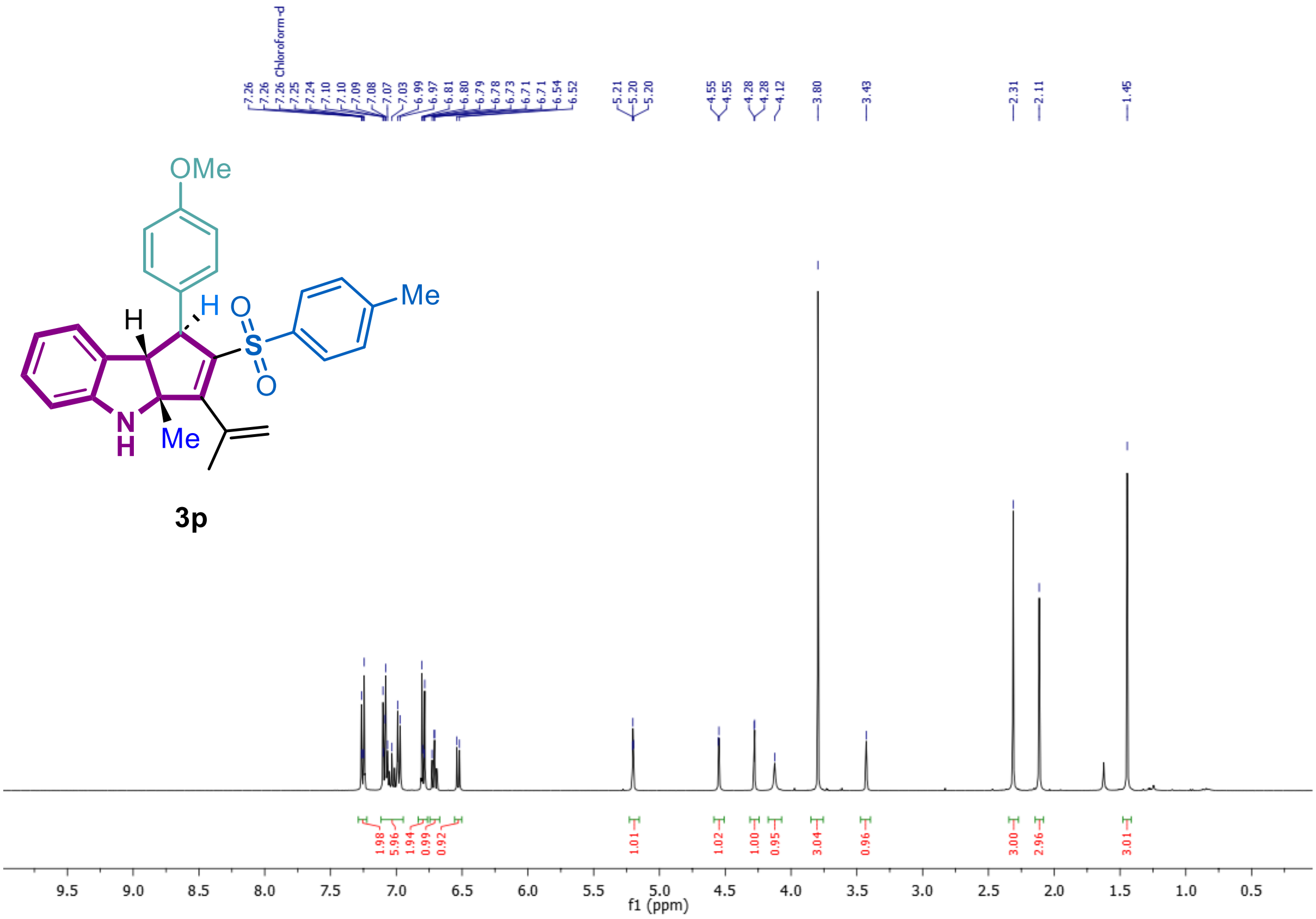

Figure S83: *^{1}H-NMR of **3p** in $CDCl_3$ (400 MHz)*

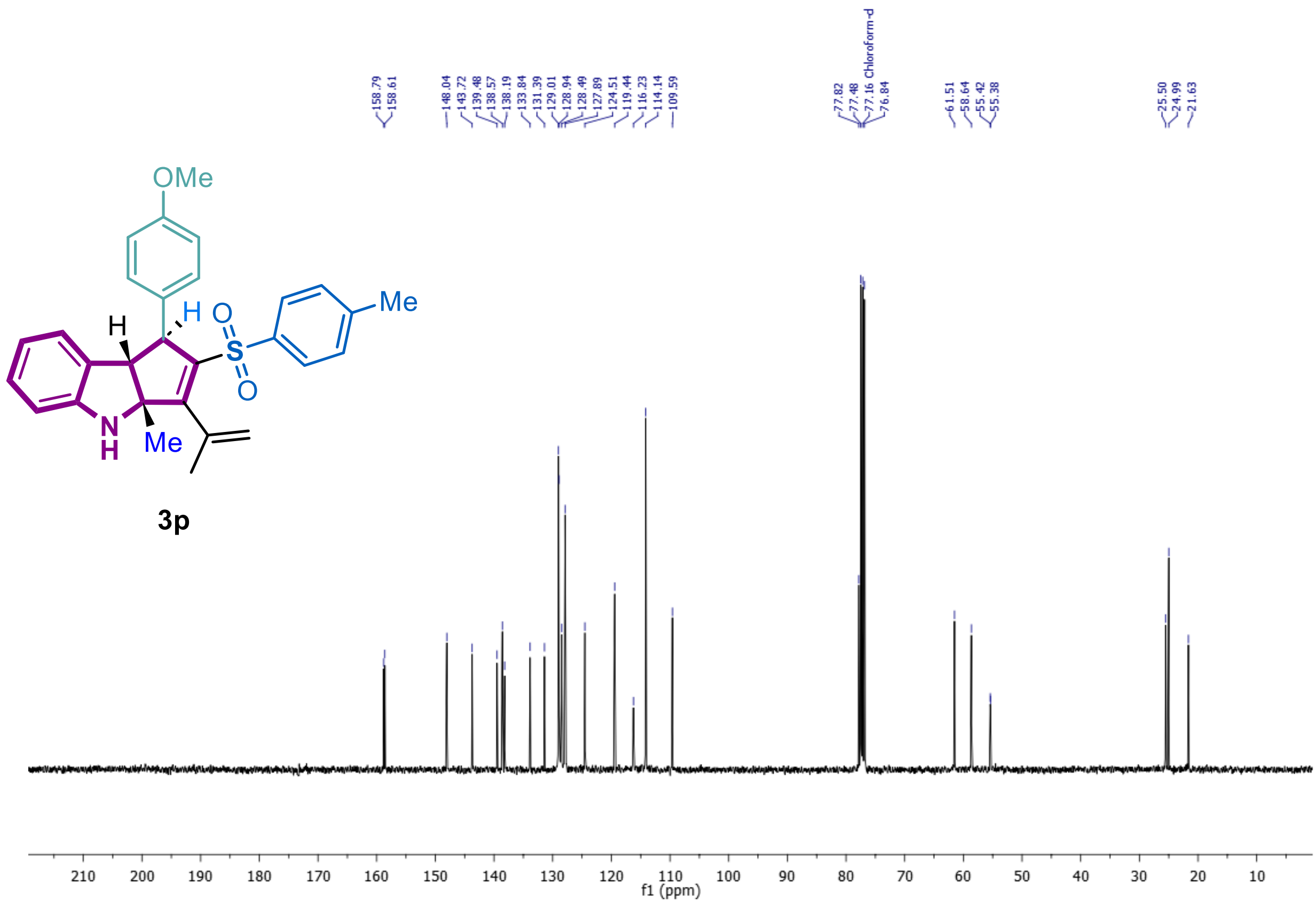

Figure S84: ^{13}C-NMR of **3p** in $CDCl_3$ (101 MHz)

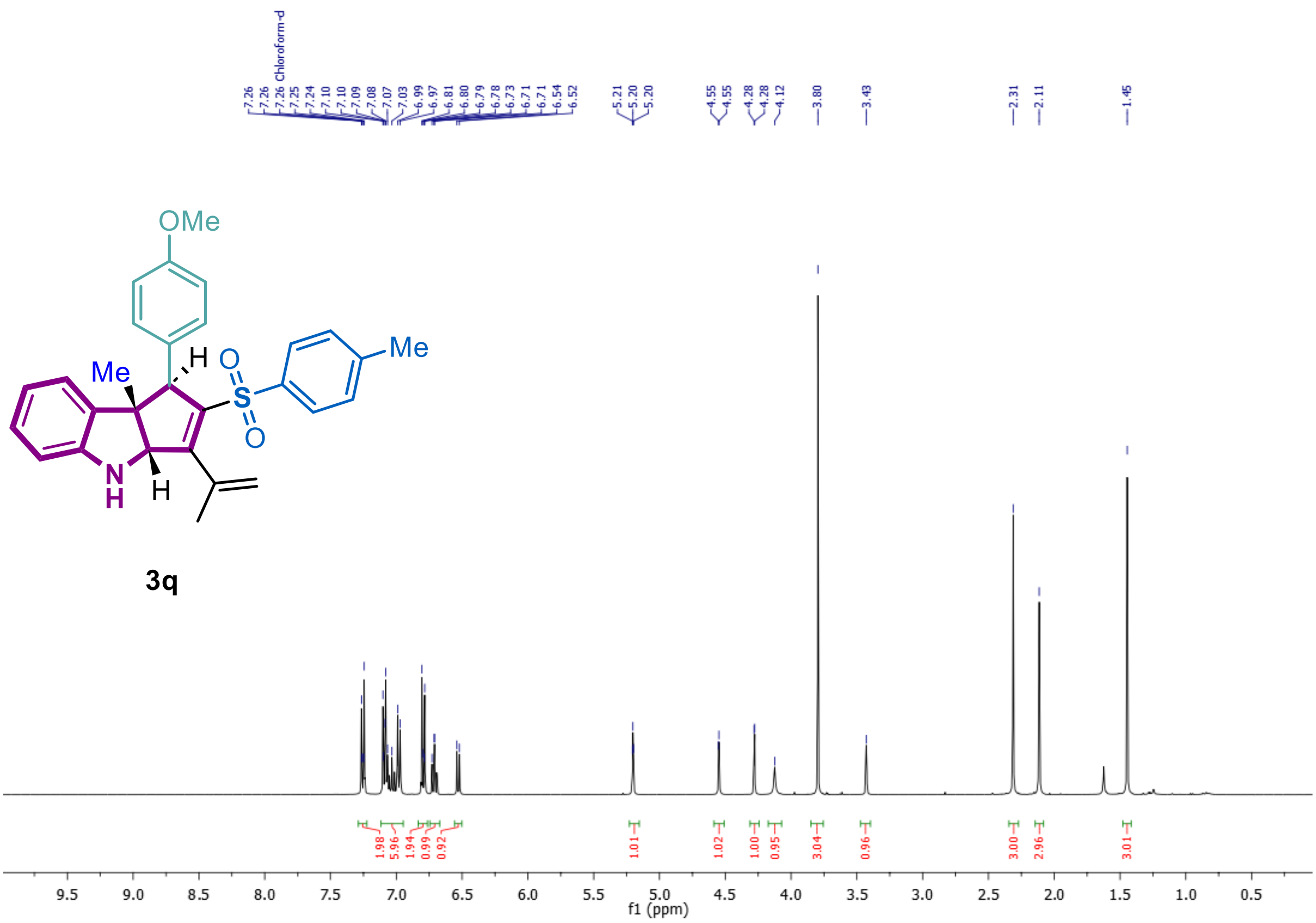

Figure S85: ^{1}H-NMR of **3q** in $CDCl_3$ (400 MHz)

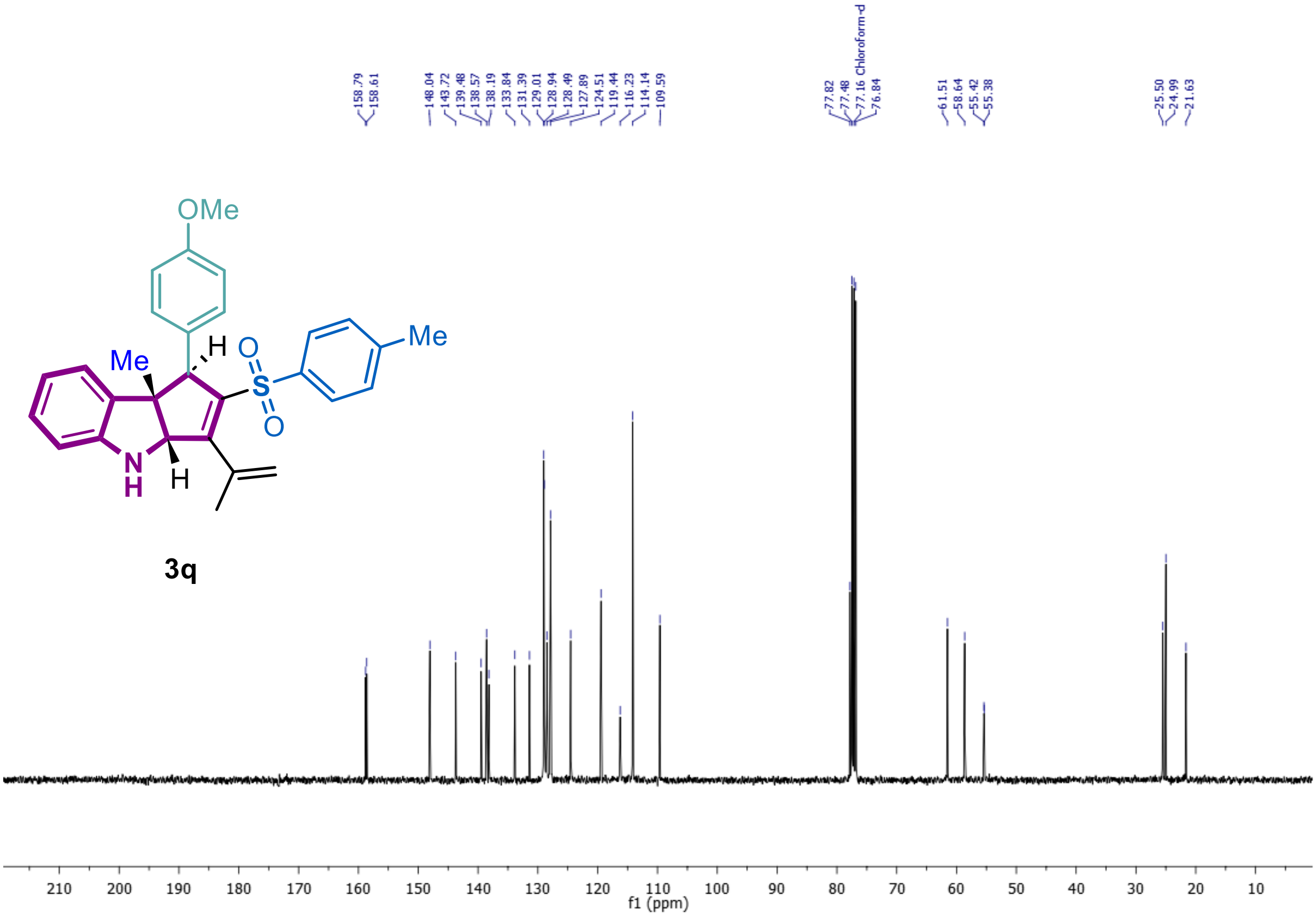

3q

Figure S86: *^{13}C-NMR of **3q** in $CDCl_3$ (101 MHz)*

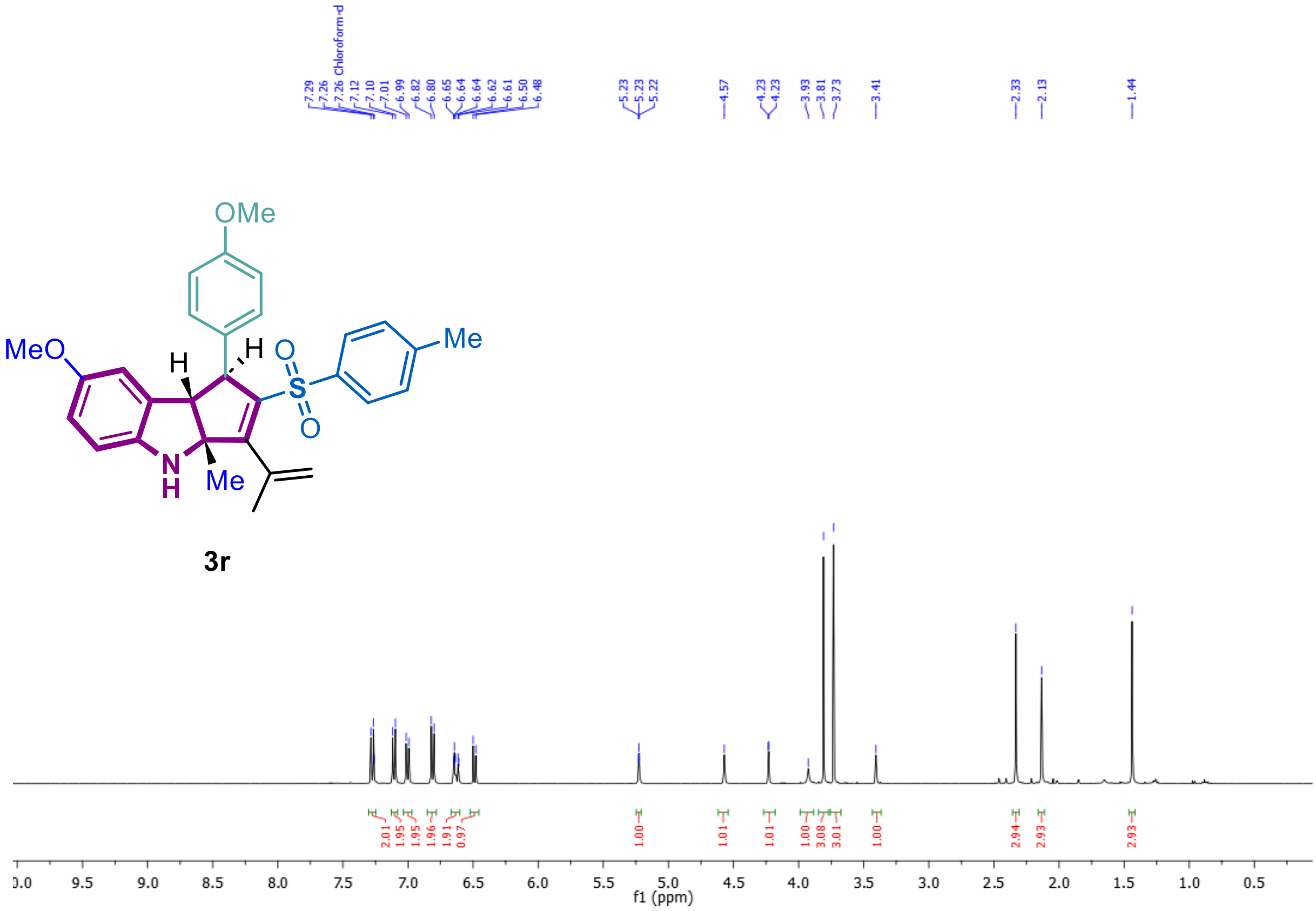

3r

Figure S87: ^{1}H-NMR of **3r** in $CDCl_3$ (400 MHz)

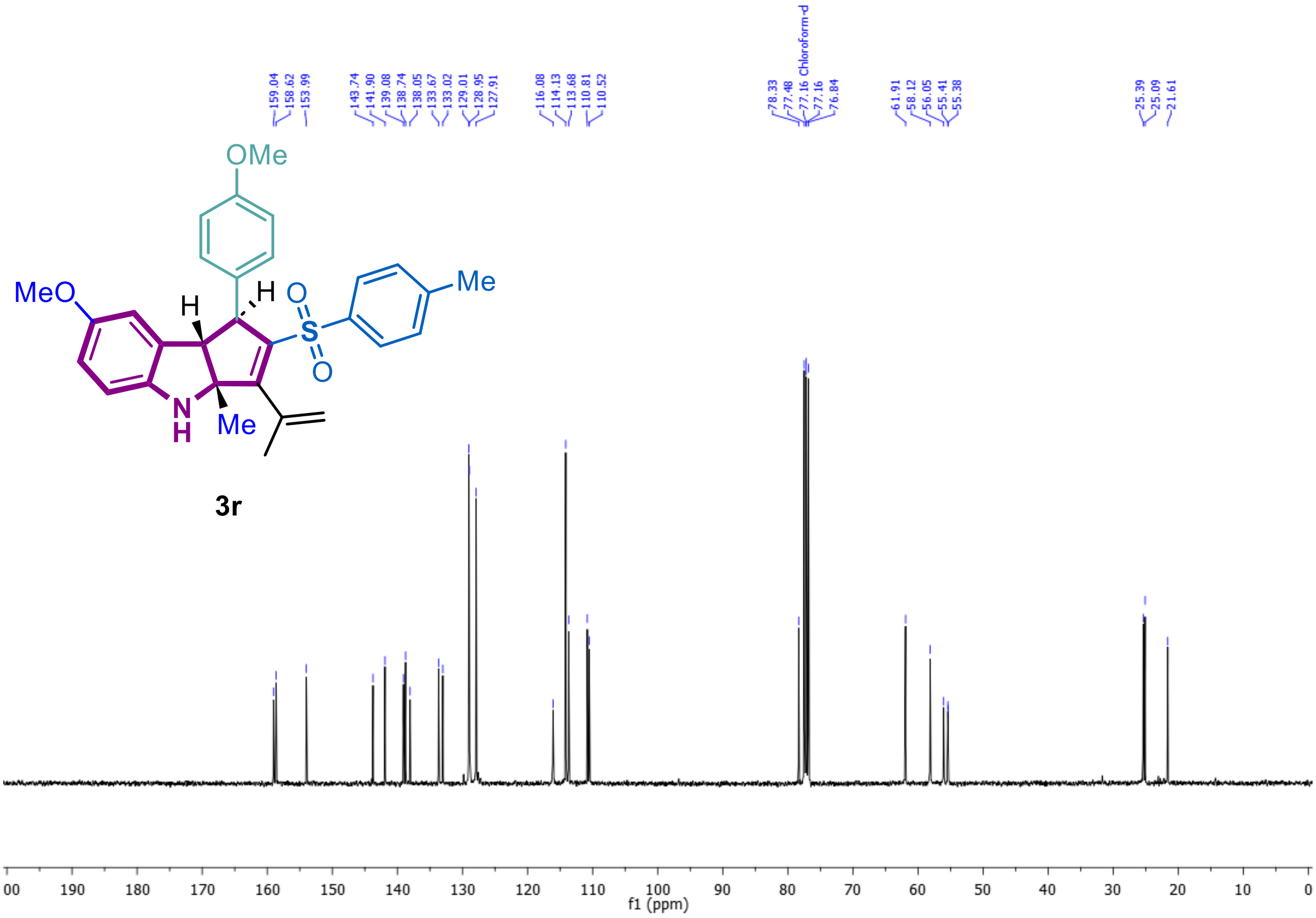

3r

Figure S88: *^{13}C-NMR of **3r** in $CDCl_3$ (101 MHz)*

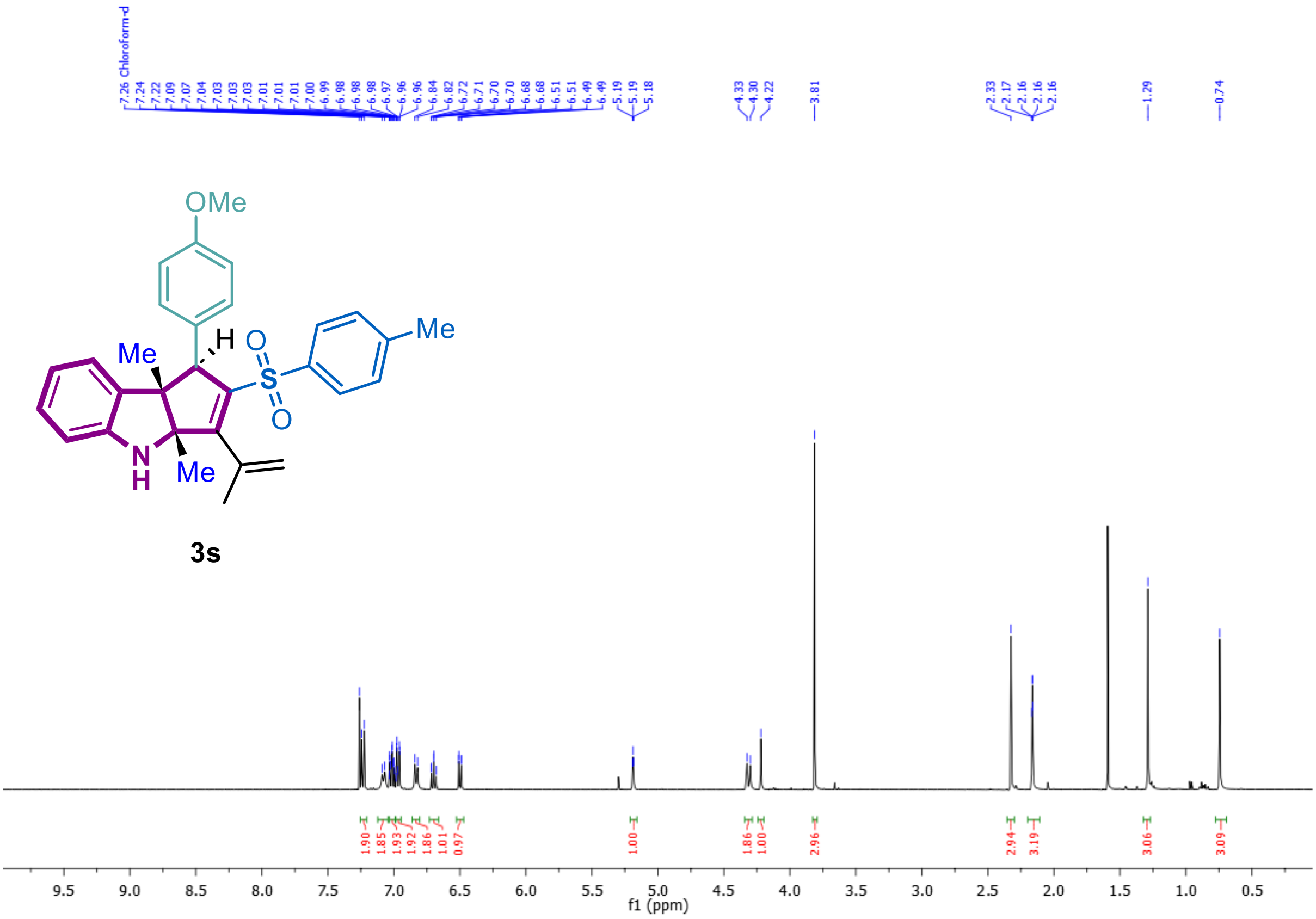

3s

Figure S89: *^{1}H-NMR of **3s** in $CDCl_3$ (400 MHz)*

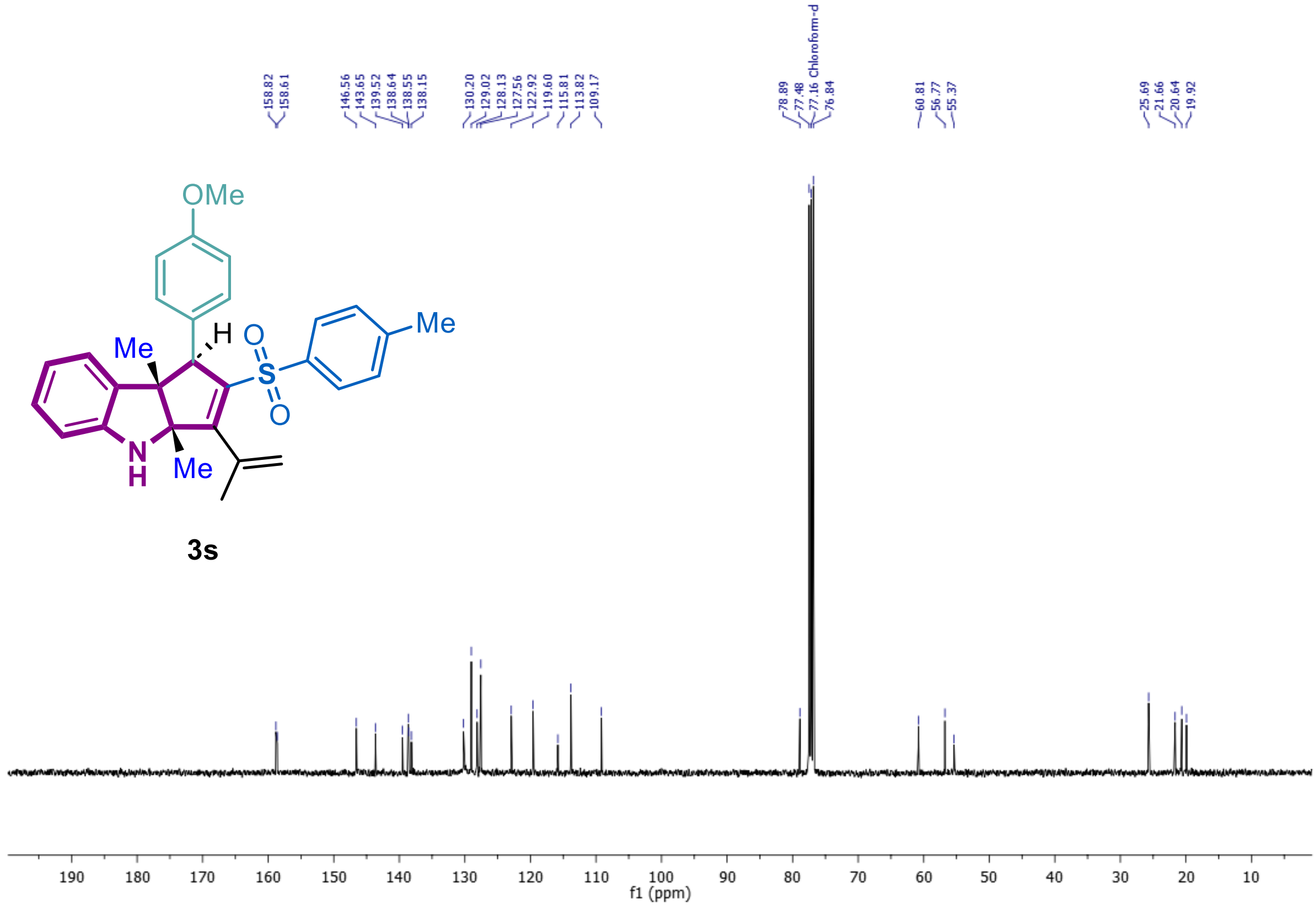

3s

Figure S90: ^{13}C-NMR of **3s** in $CDCl_3$ (101 MHz)

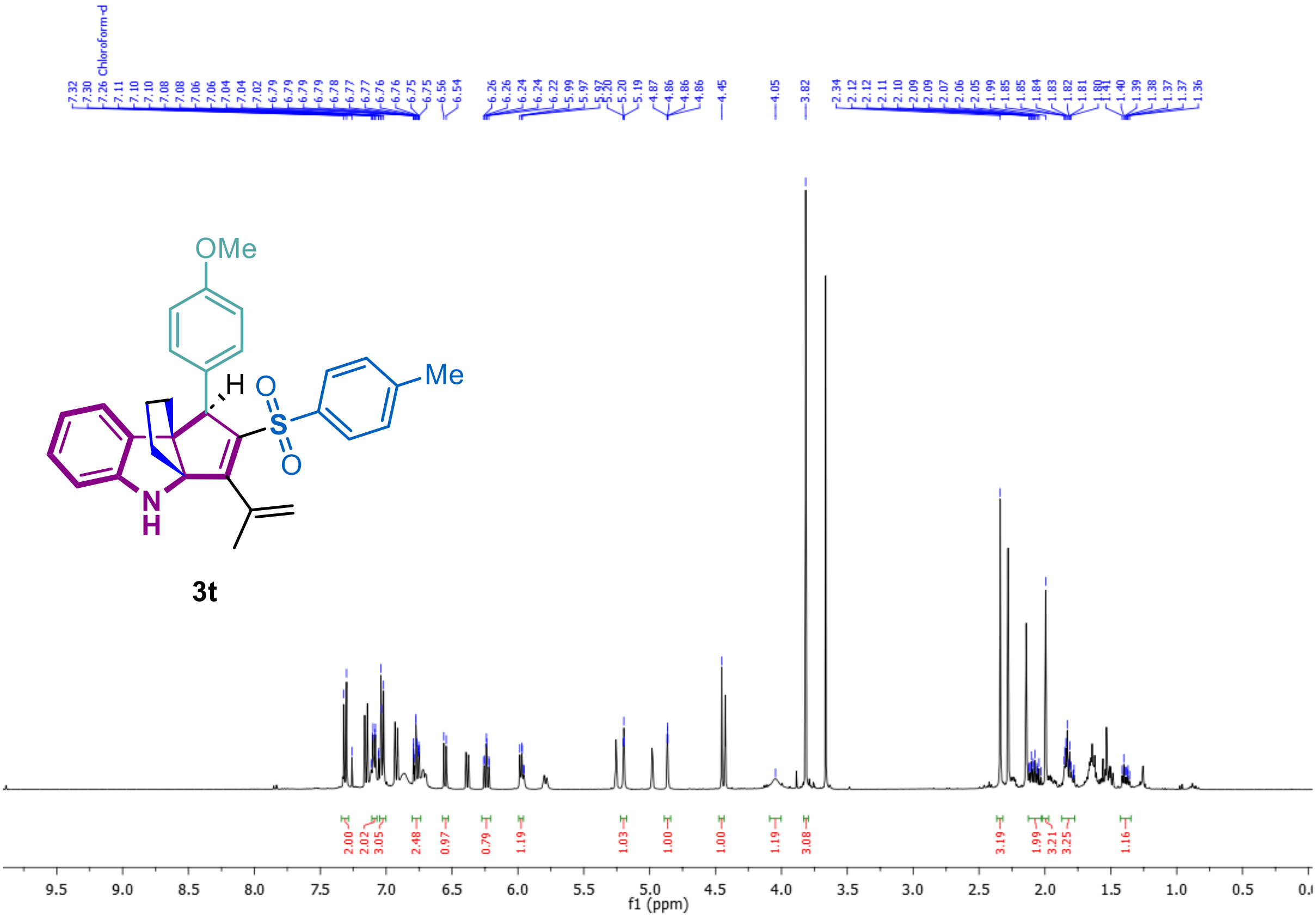

3t

Figure S91: ^{1}H-NMR of **3t** in $CDCl_3$ (400 MHz)

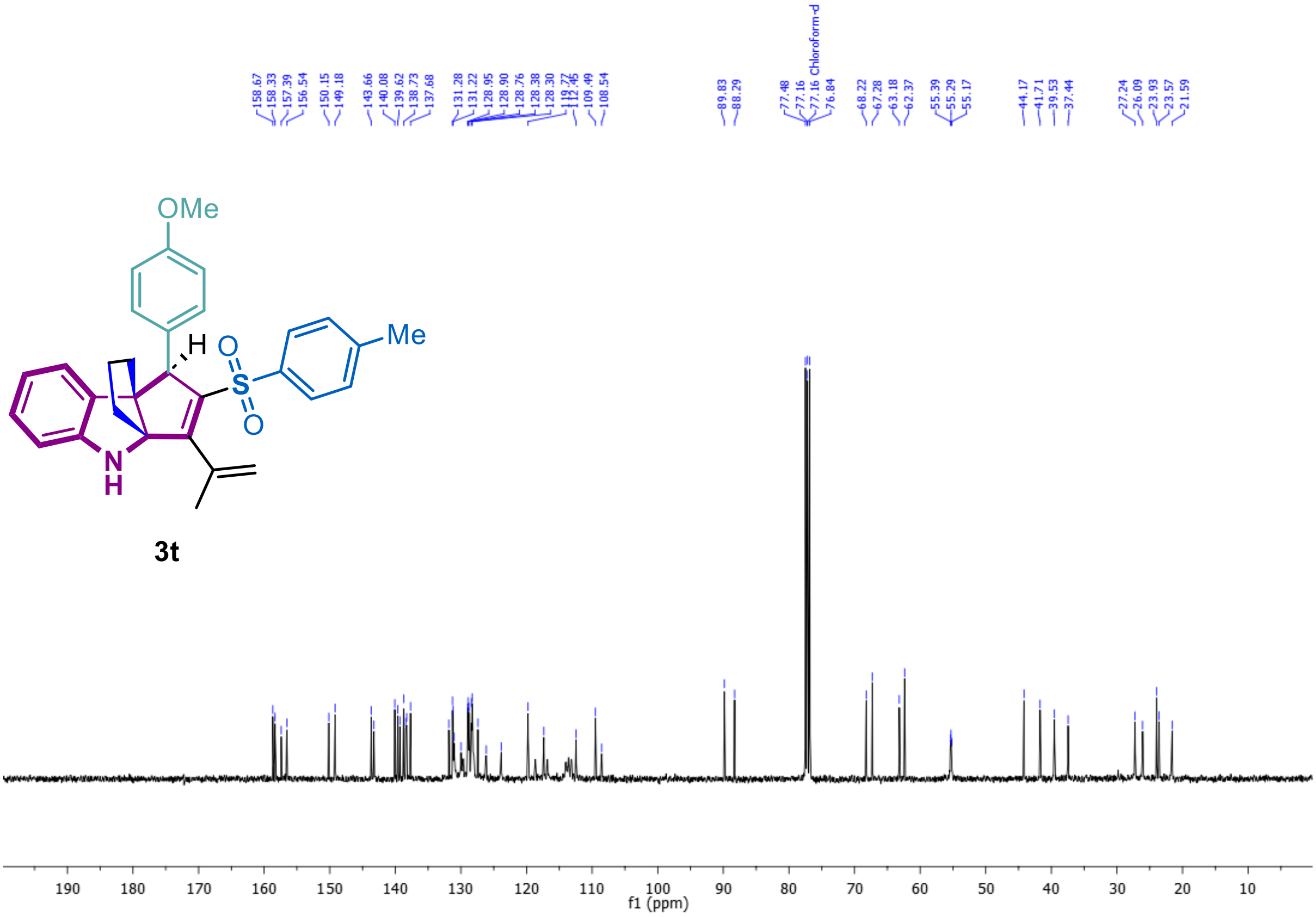

Figure S92: ^{13}C-NMR of ***3t*** *in* $CDCl_3$ *(101 MHz)*

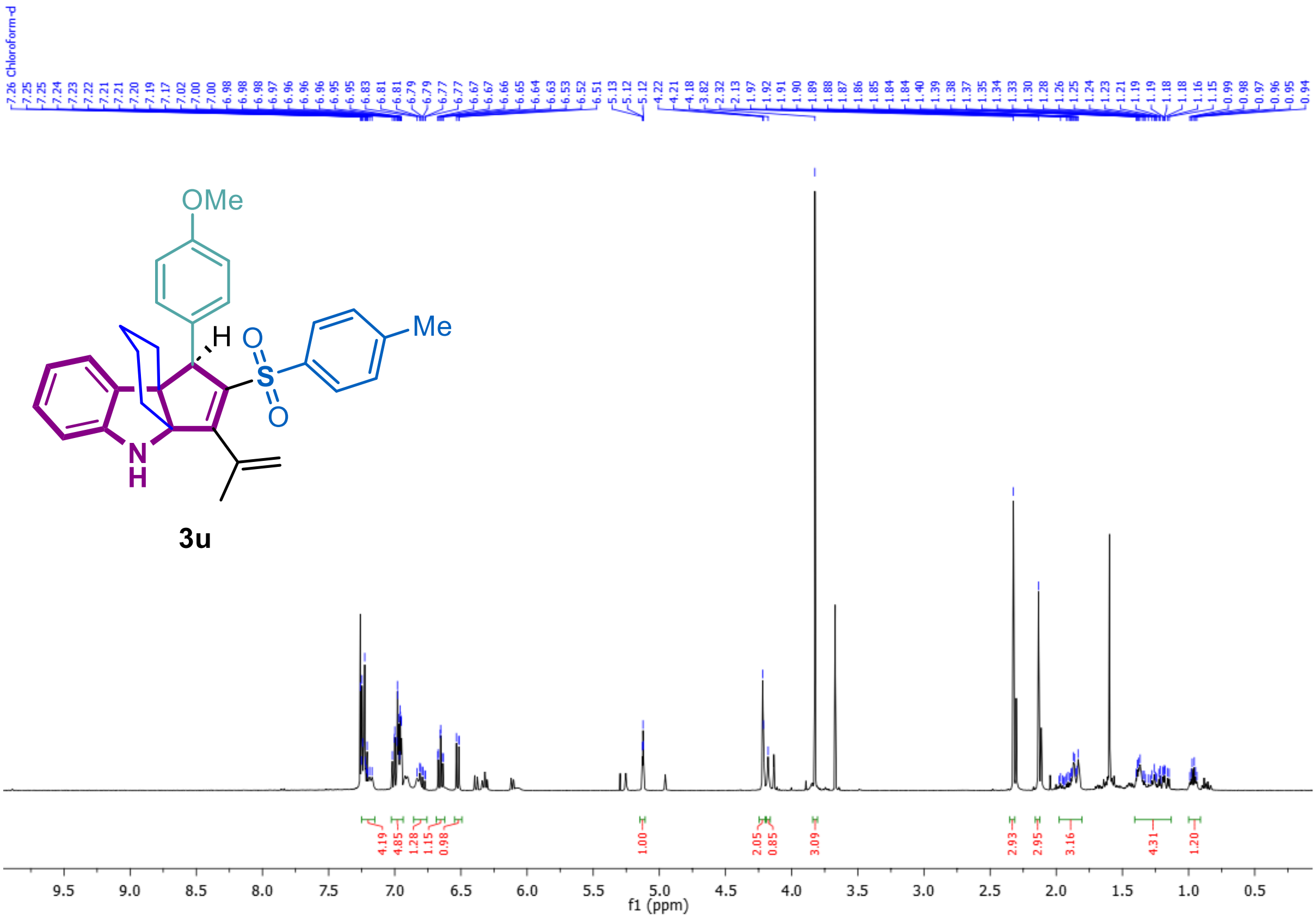

Figure S93: ^{1}H-NMR of ***3u*** *in* $CDCl_3$ *(400 MHz)*

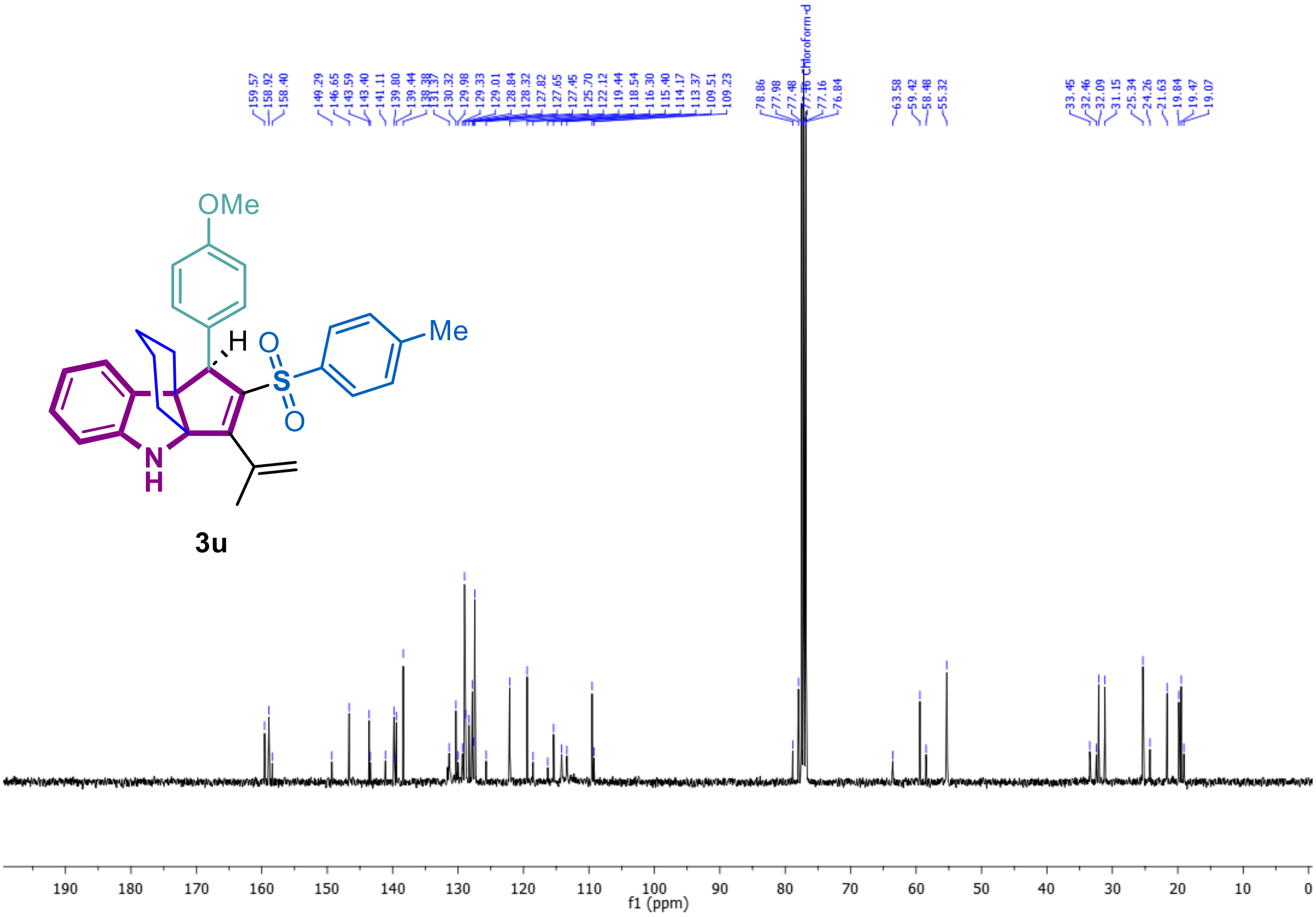

Figure S94: ^{13}C-NMR of **3u** in $CDCl_3$ (101 MHz)

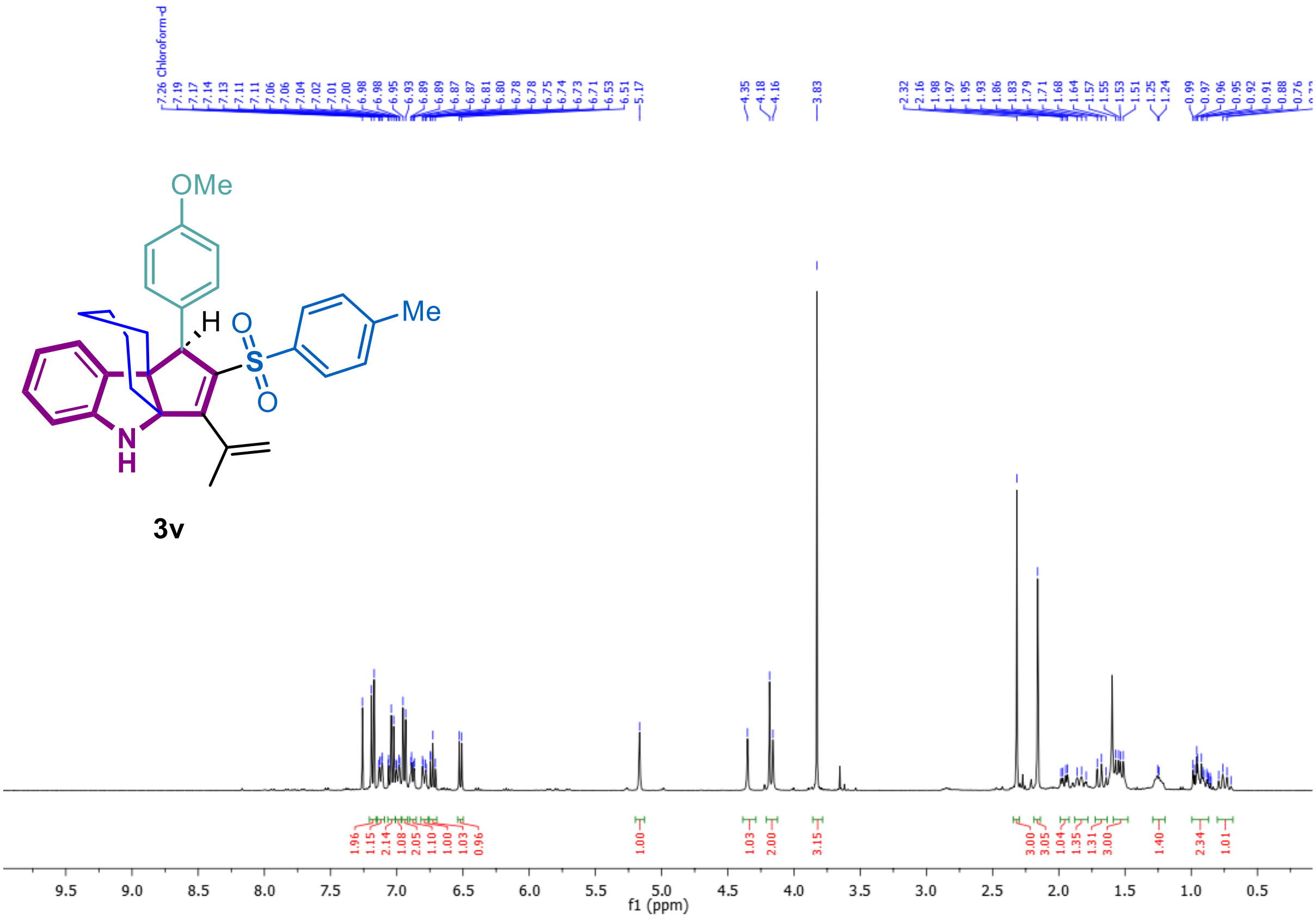

Figure S95: ^{1}H-NMR of **3v** in $CDCl_3$ (400 MHz)

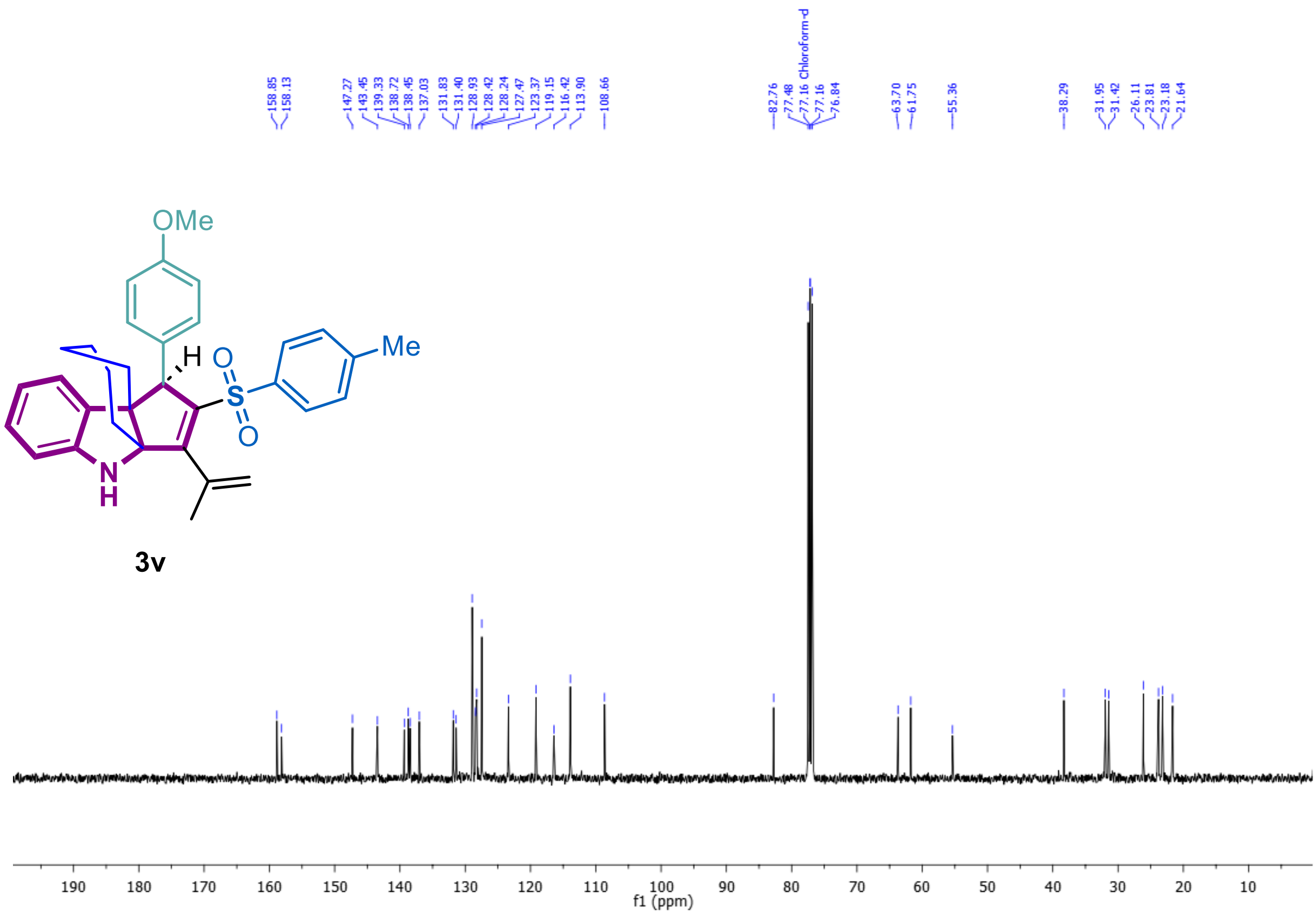

Figure S96: ^{13}C-NMR of **3v** in $CDCl_3$ (101 MHz)

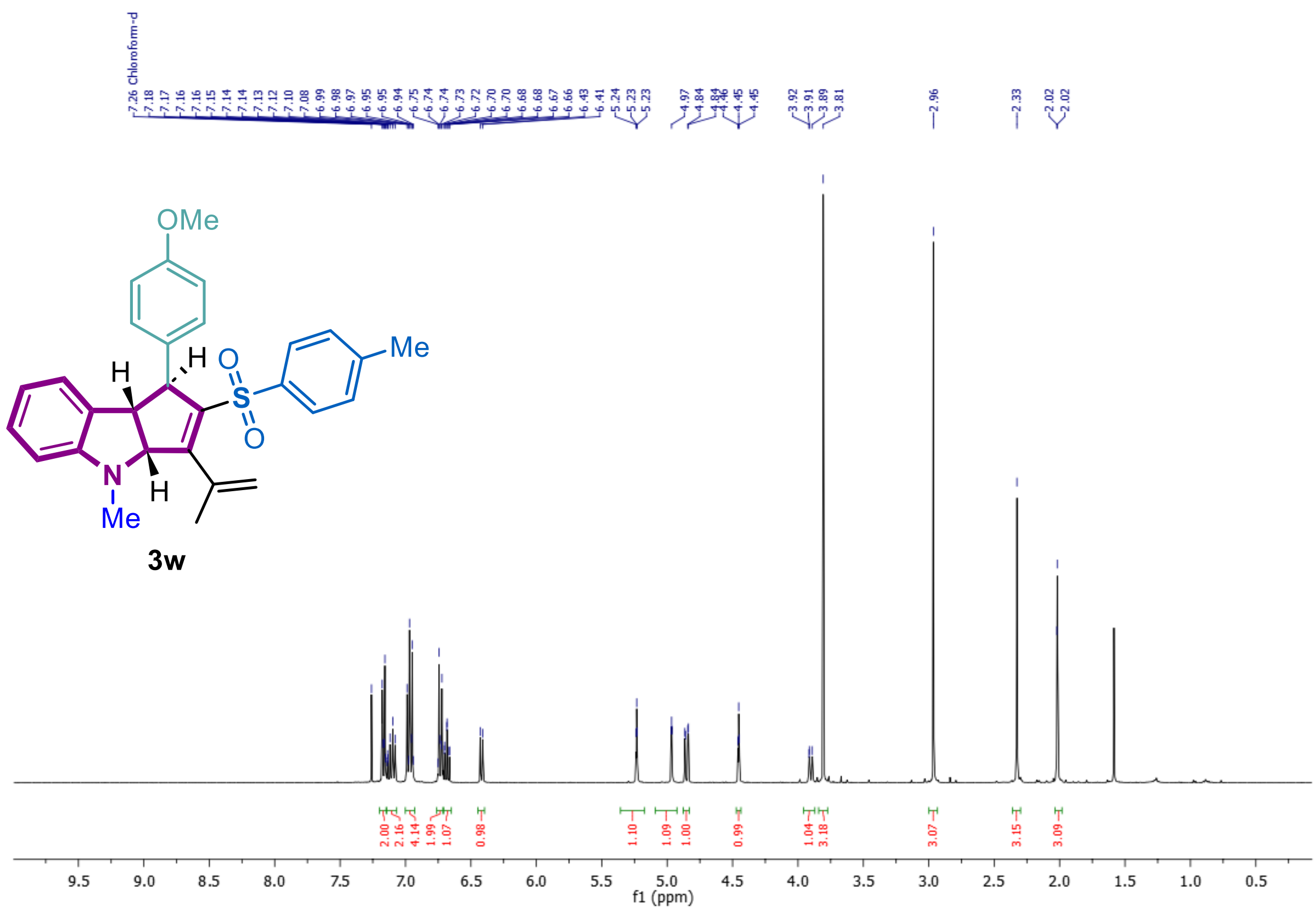

Figure S97: ^{1}H-NMR of **3w** in $CDCl_3$ (400 MHz)

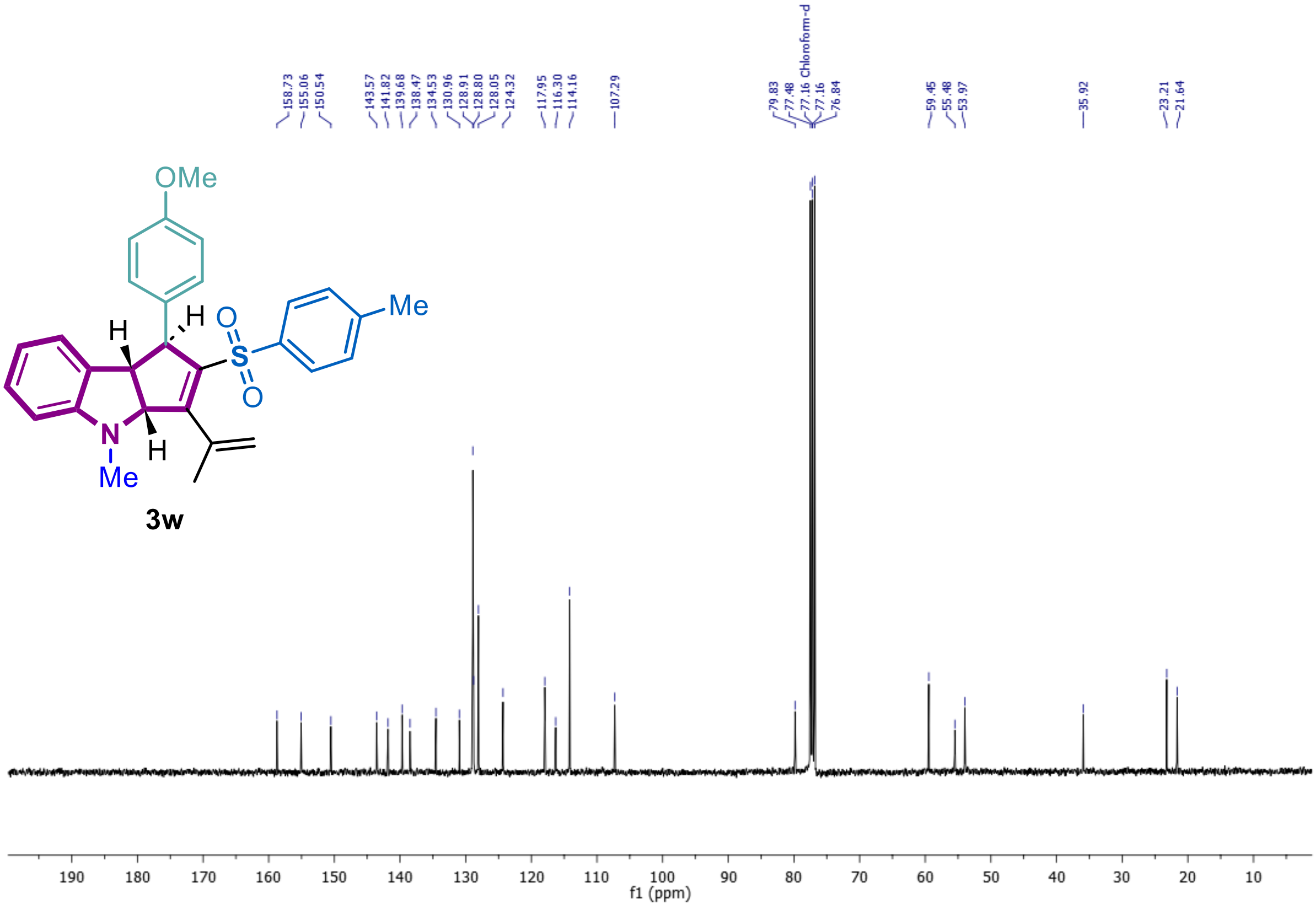

Figure S98: ^{13}C-NMR of **3w** in $CDCl_3$ (101 MHz)

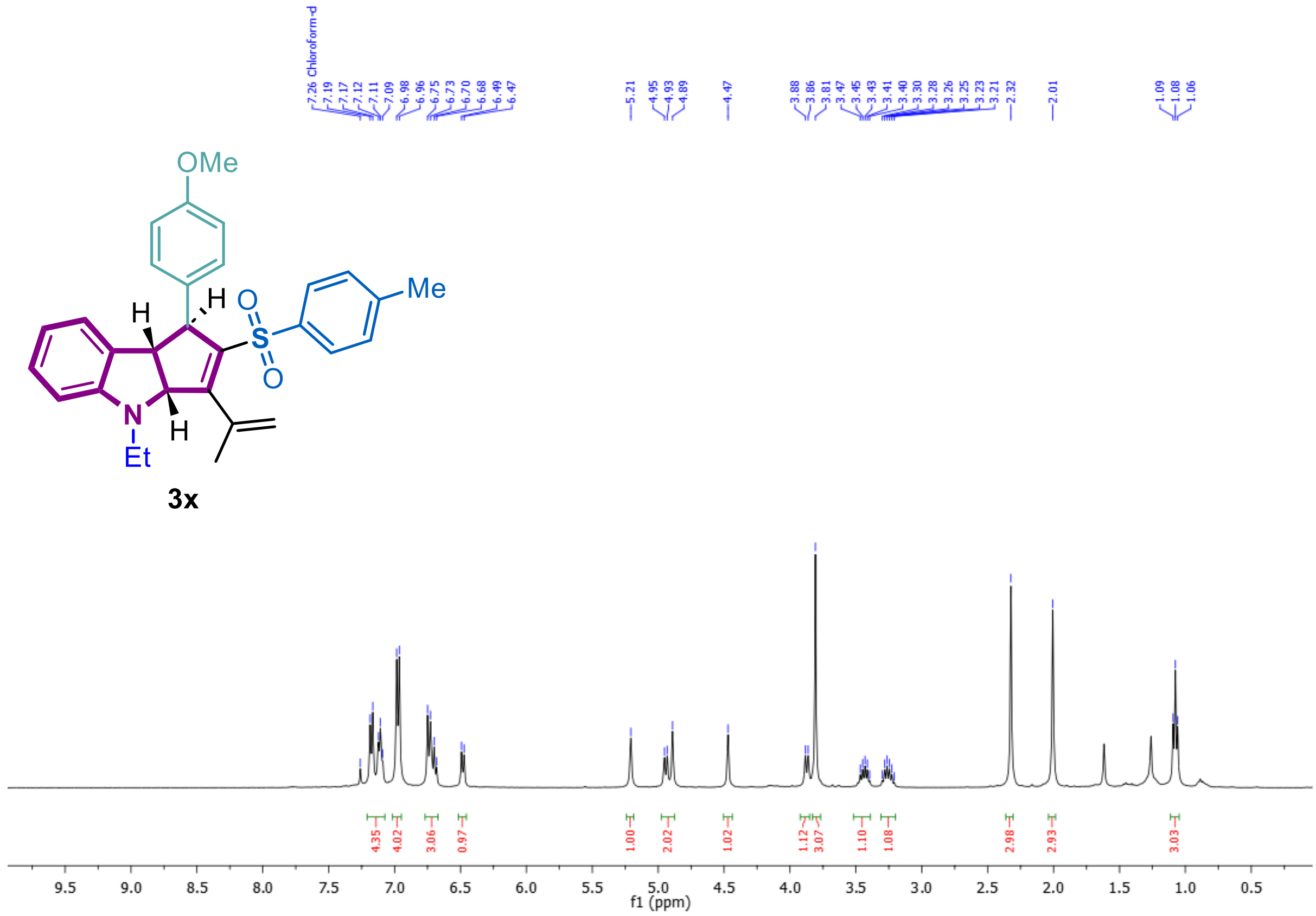

Figure S99: ^{1}H-NMR of **3x** in $CDCl_3$ (400 MHz)

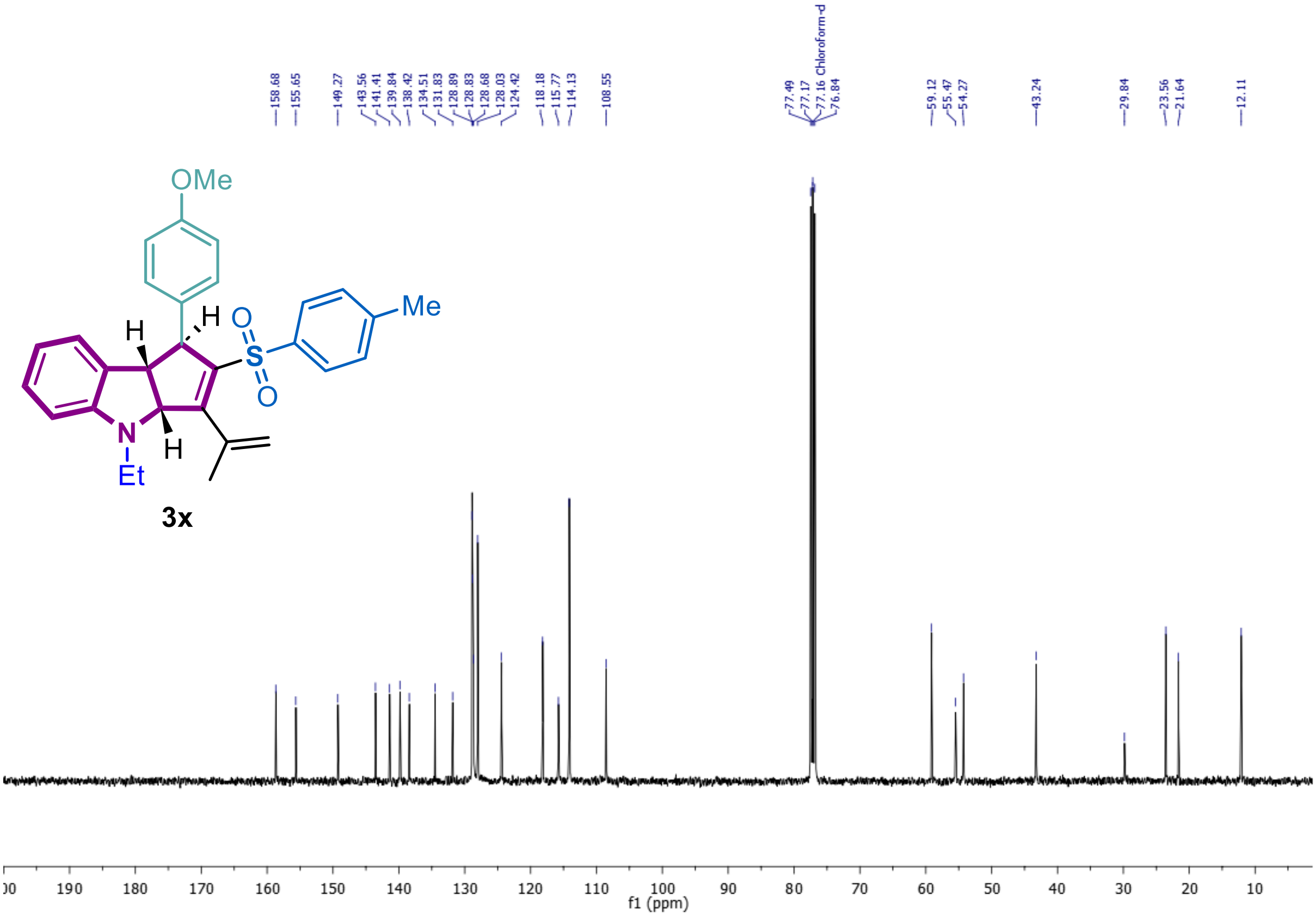

Figure S100: *^{13}C-NMR of* ***3x*** *in $CDCl_3$ (101 MHz)*

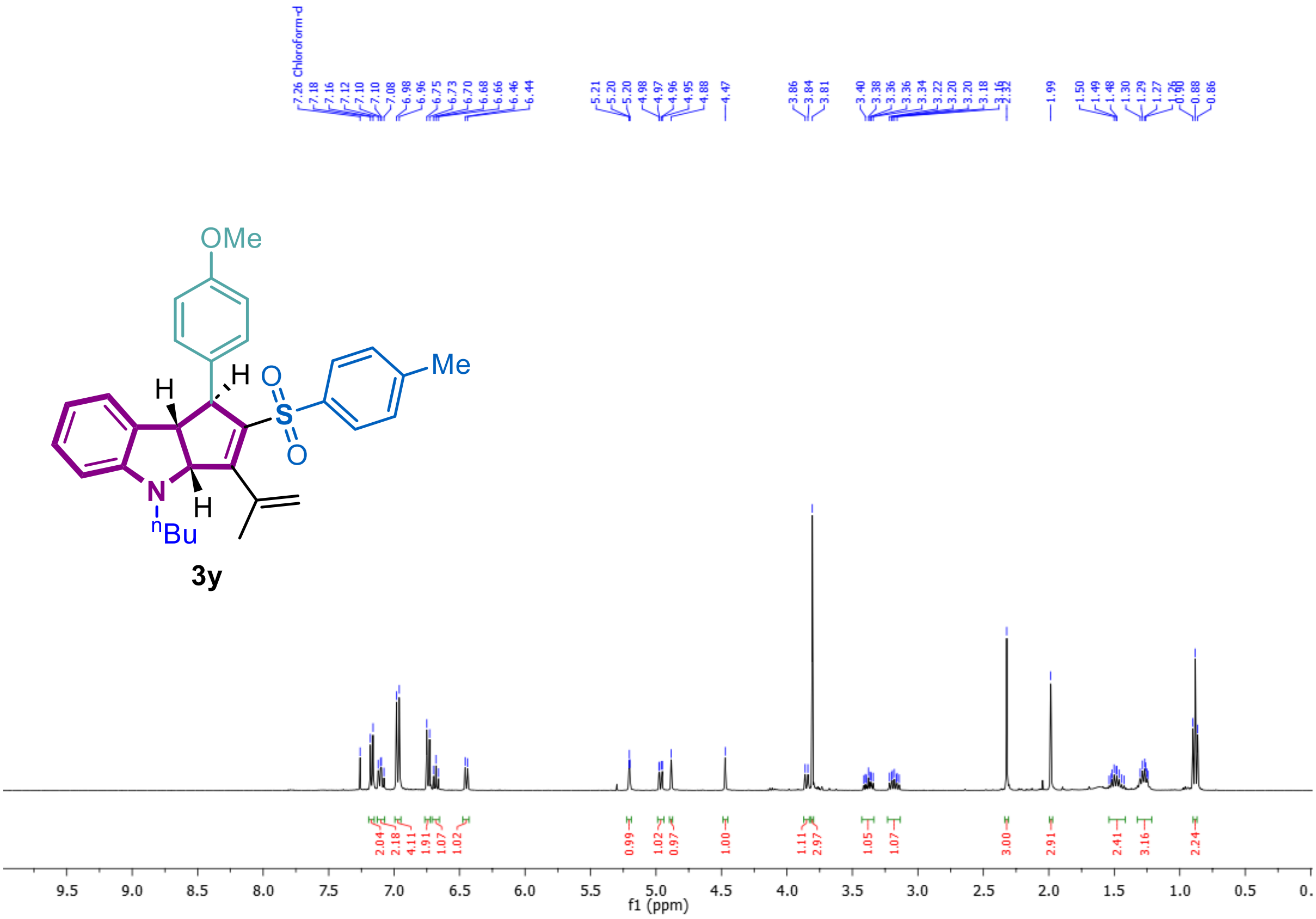

Figure S101: *^{1}H-NMR of* ***3y*** *in $CDCl_3$ (400 MHz)*

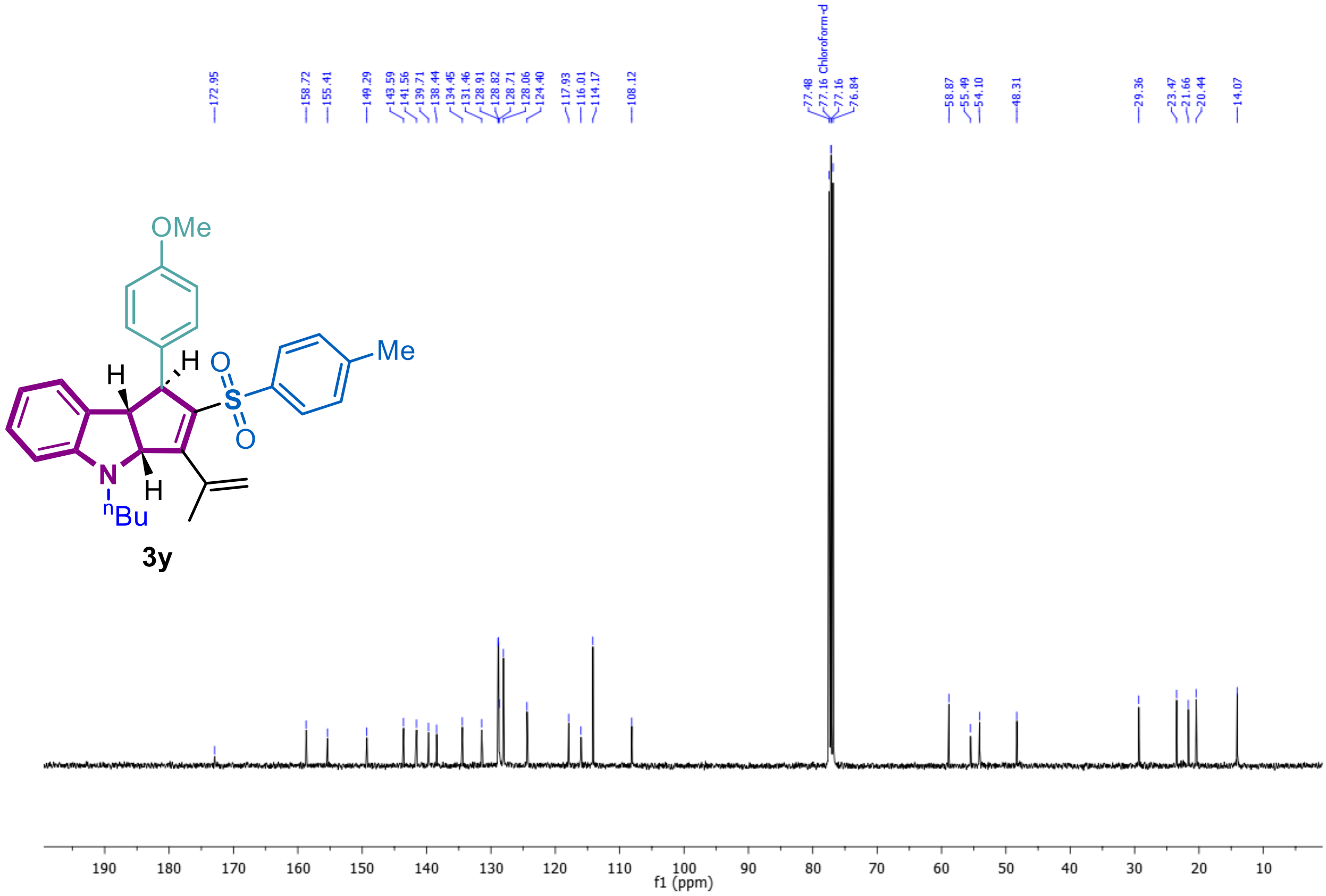

3y

Figure S102*: ^{13}C-NMR of* ***3y*** *in $CDCl_3$ (101 MHz)*

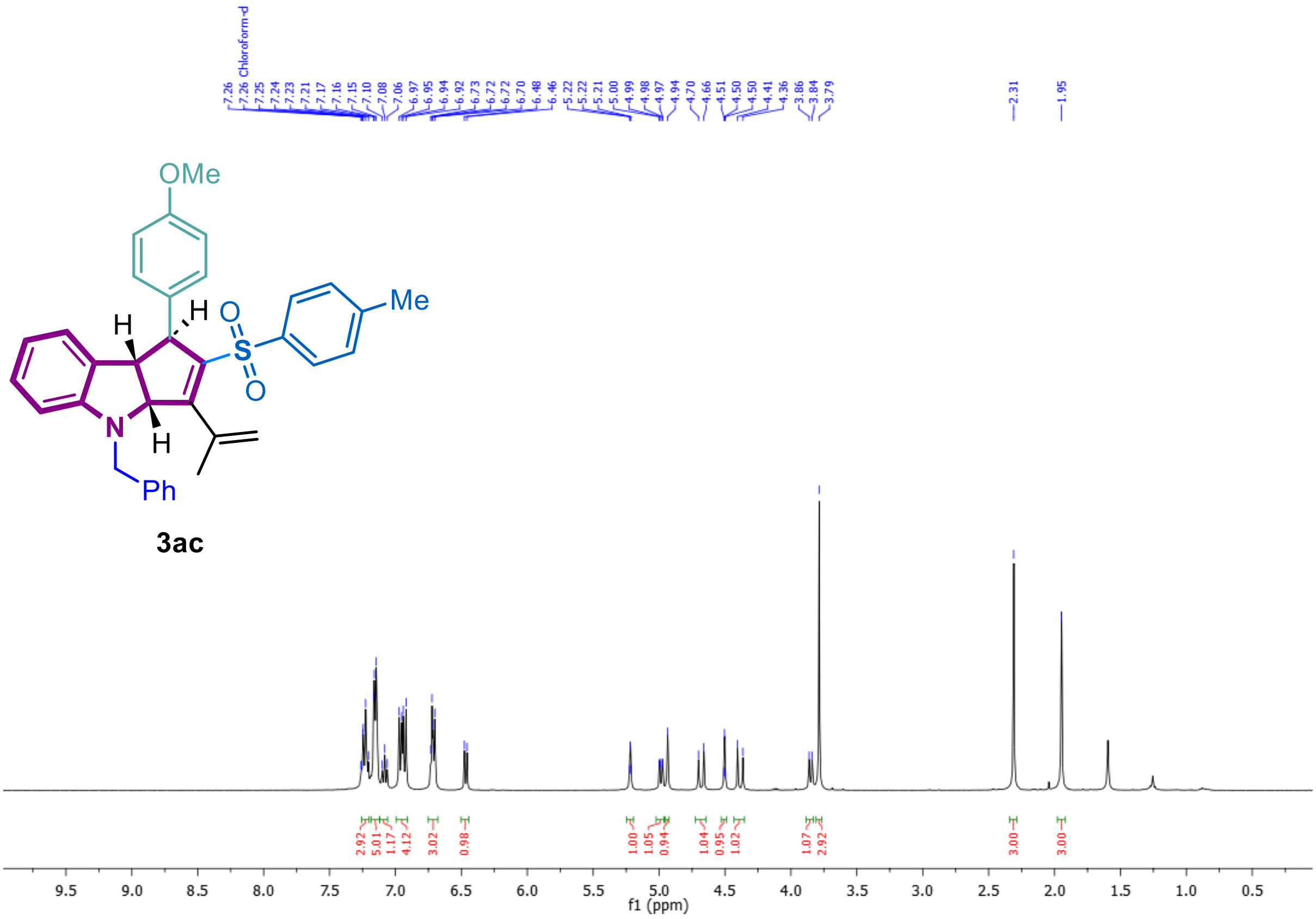

3ac

Figure S103*: ^{1}H-NMR of* ***3ac*** *in $CDCl_3$ (400 MHz)*

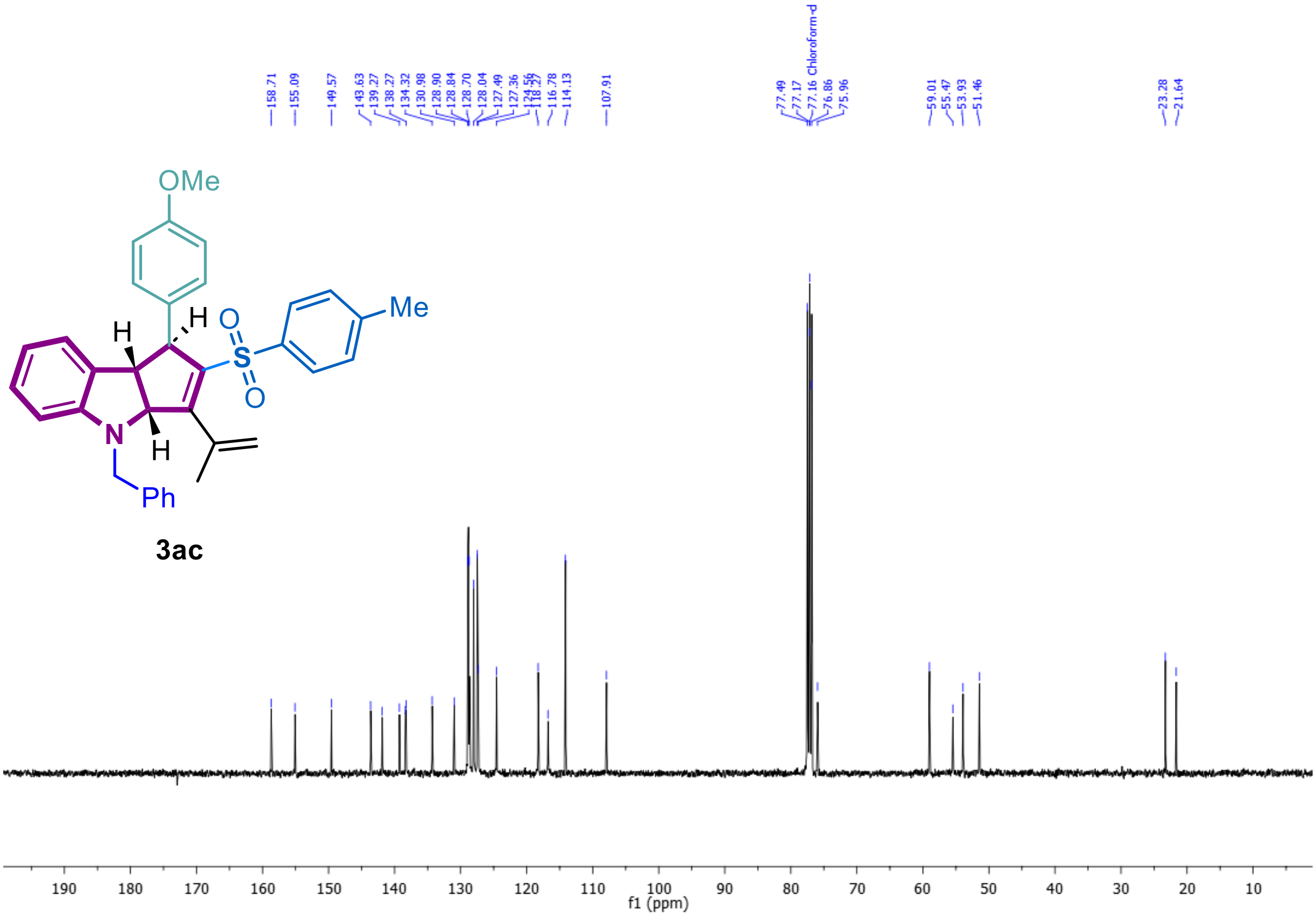

3ac

Figure S104: ^{13}C-NMR of **3ac** in $CDCl_3$ (101 MHz)

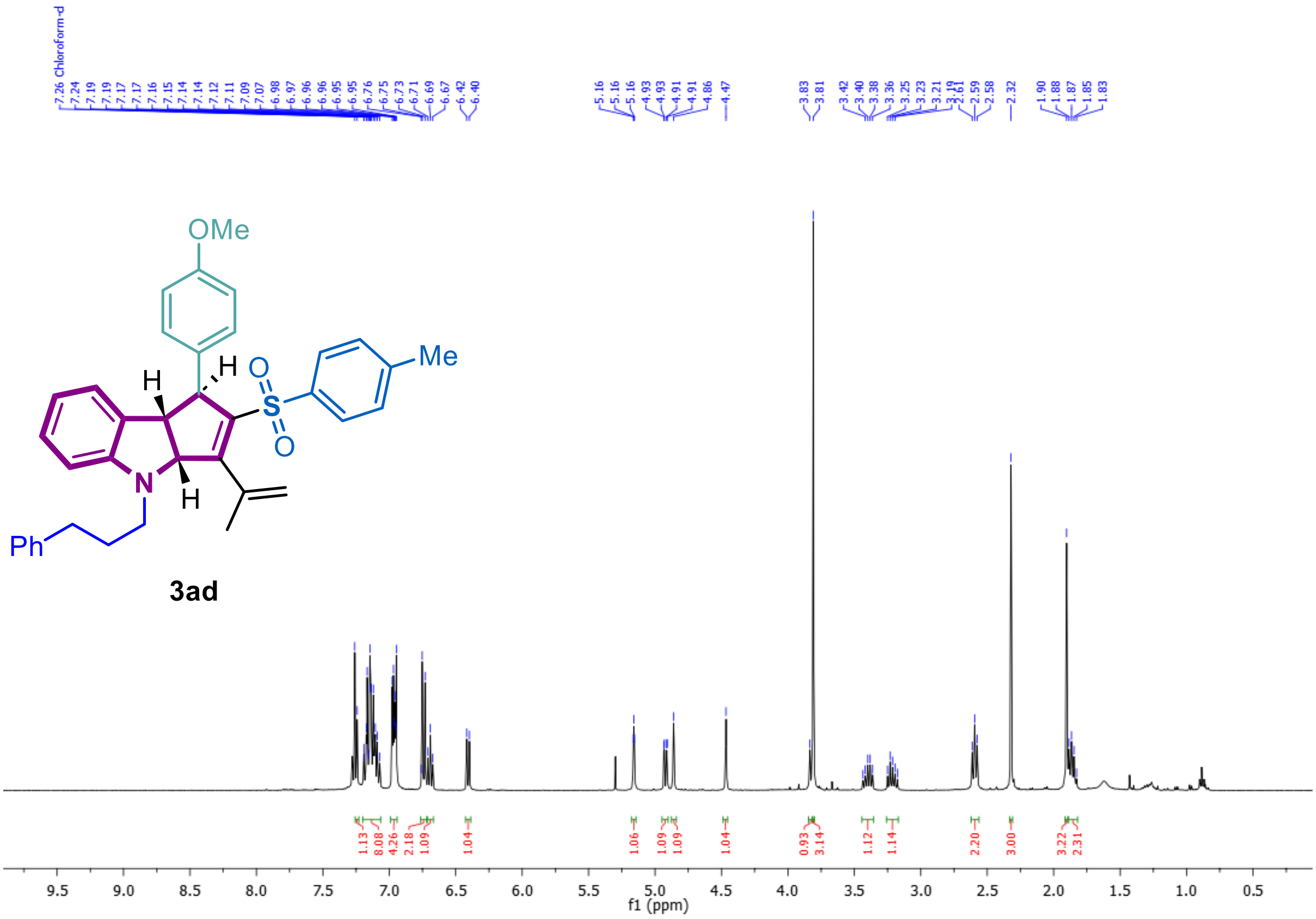

3ad

Figure S105: ^{1}H-NMR of **3ad** in $CDCl_3$ (400 MHz)

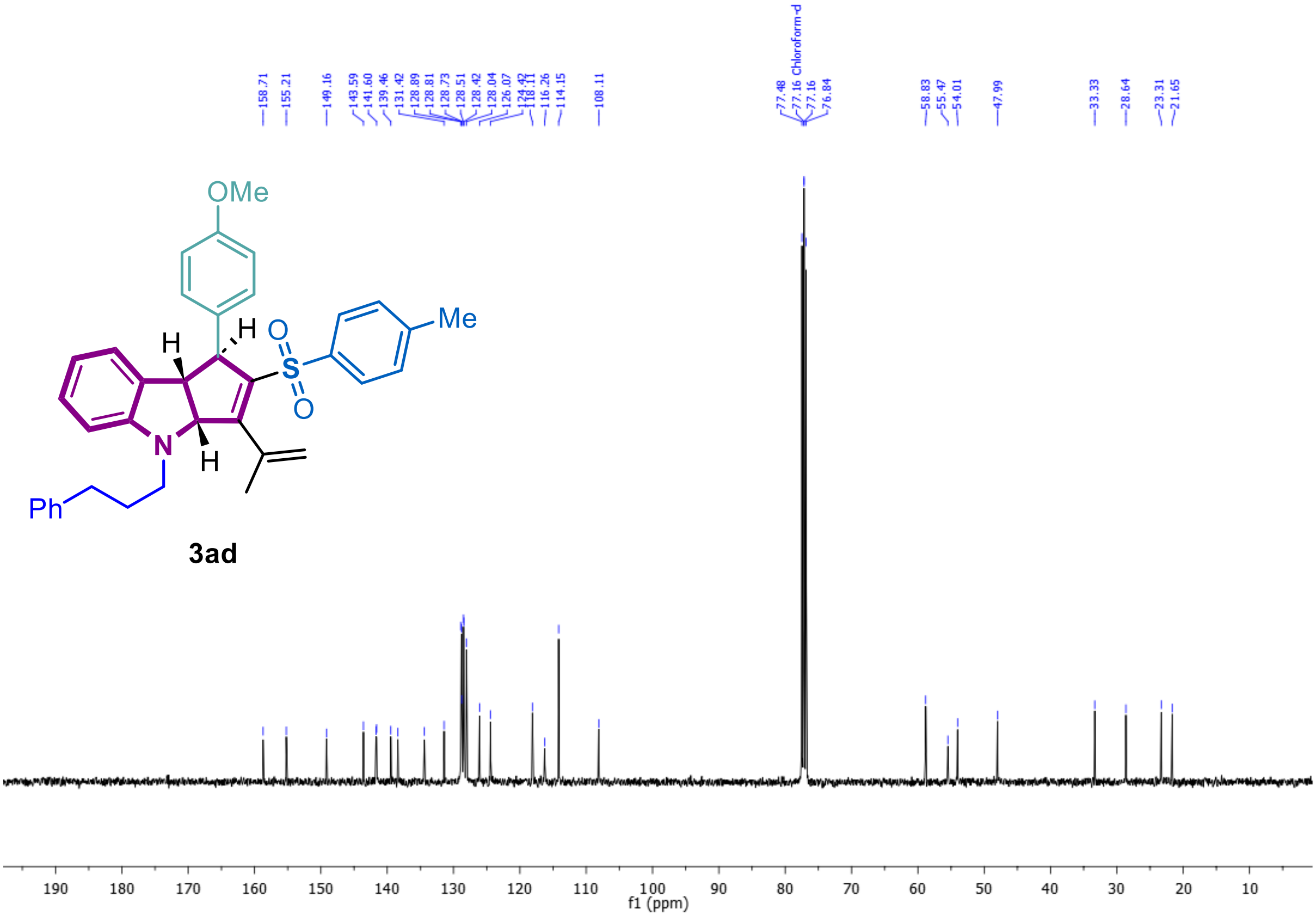

Figure S106: ^{13}C-NMR of **3ad** in $CDCl_3$ (101 MHz)

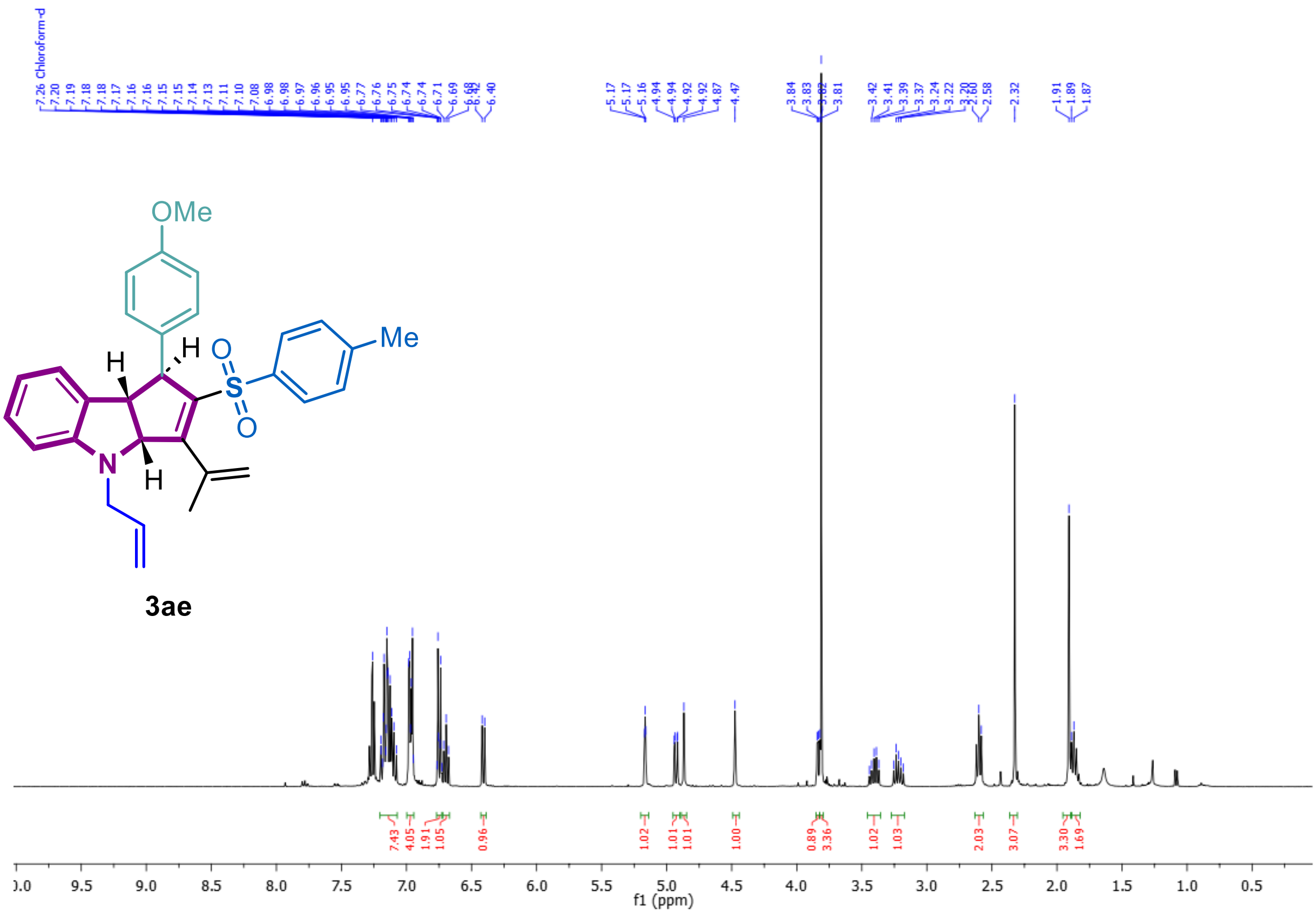

Figure S107: ^{1}H-NMR of **3ae** in $CDCl_3$ (400 MHz)

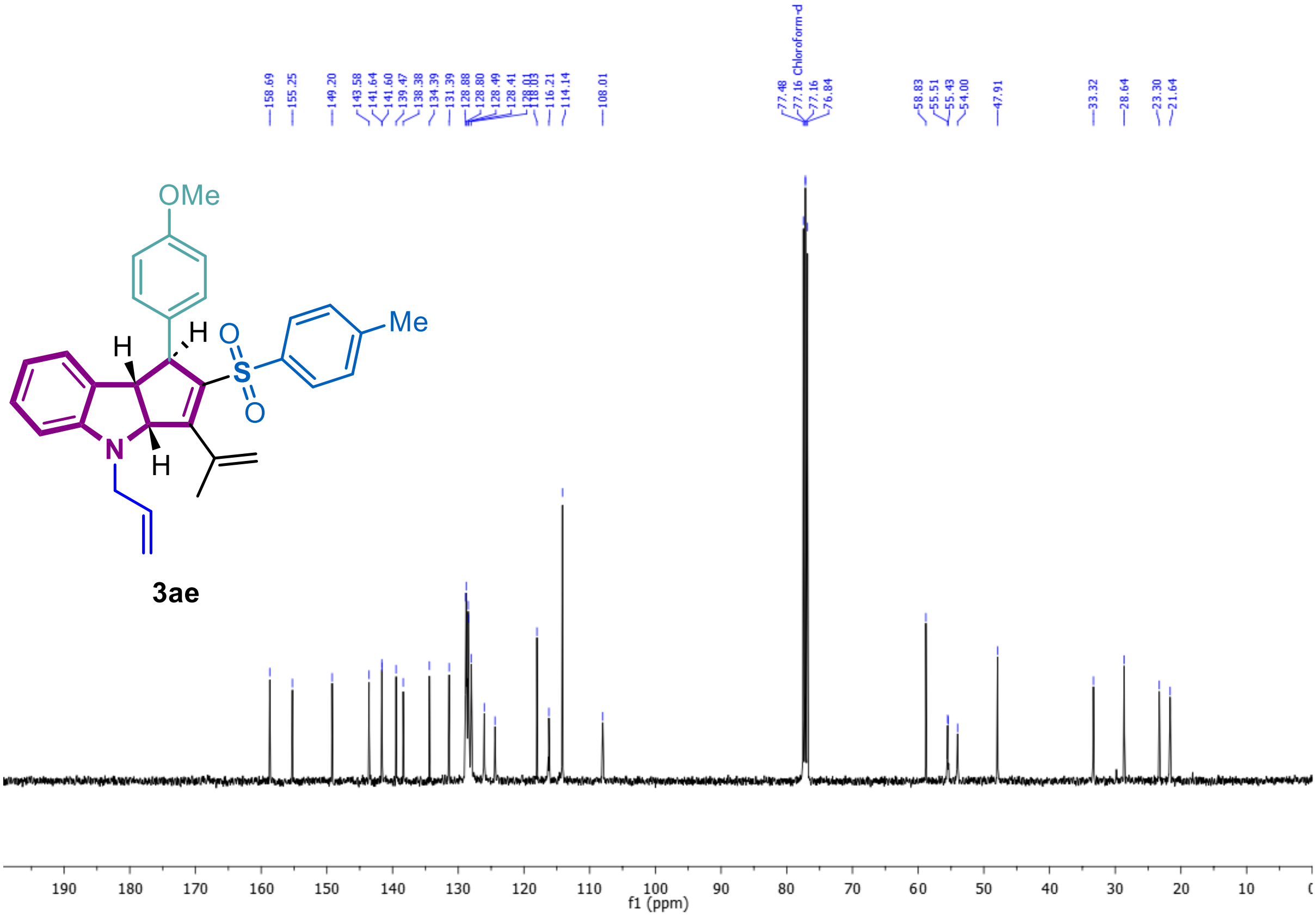

Figure S108: *^{13}C-NMR of **3ae** in $CDCl_3$ (101 MHz)*

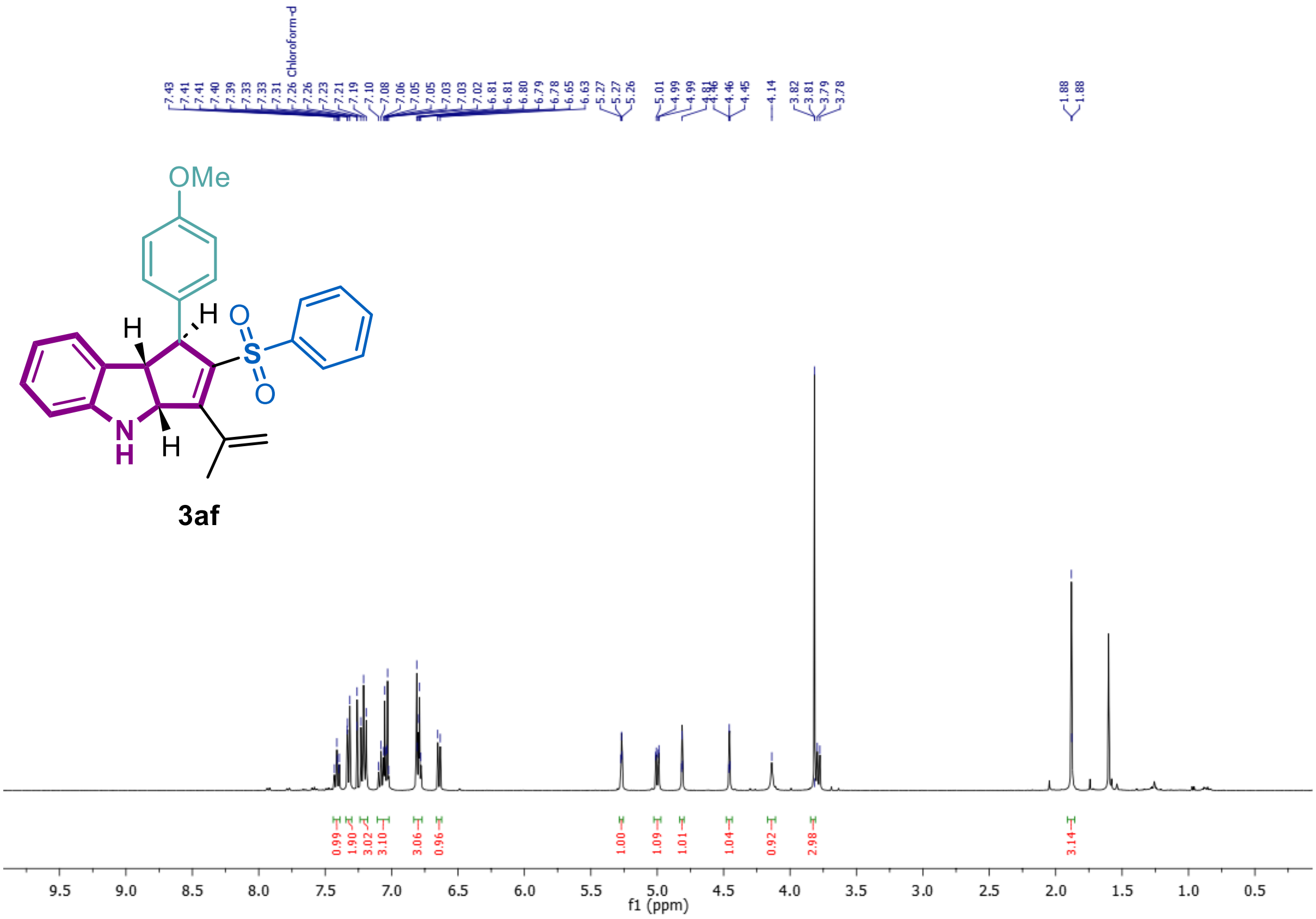

Figure S109: *^{1}H-NMR of **3af** in $CDCl_3$ (400 MHz)*

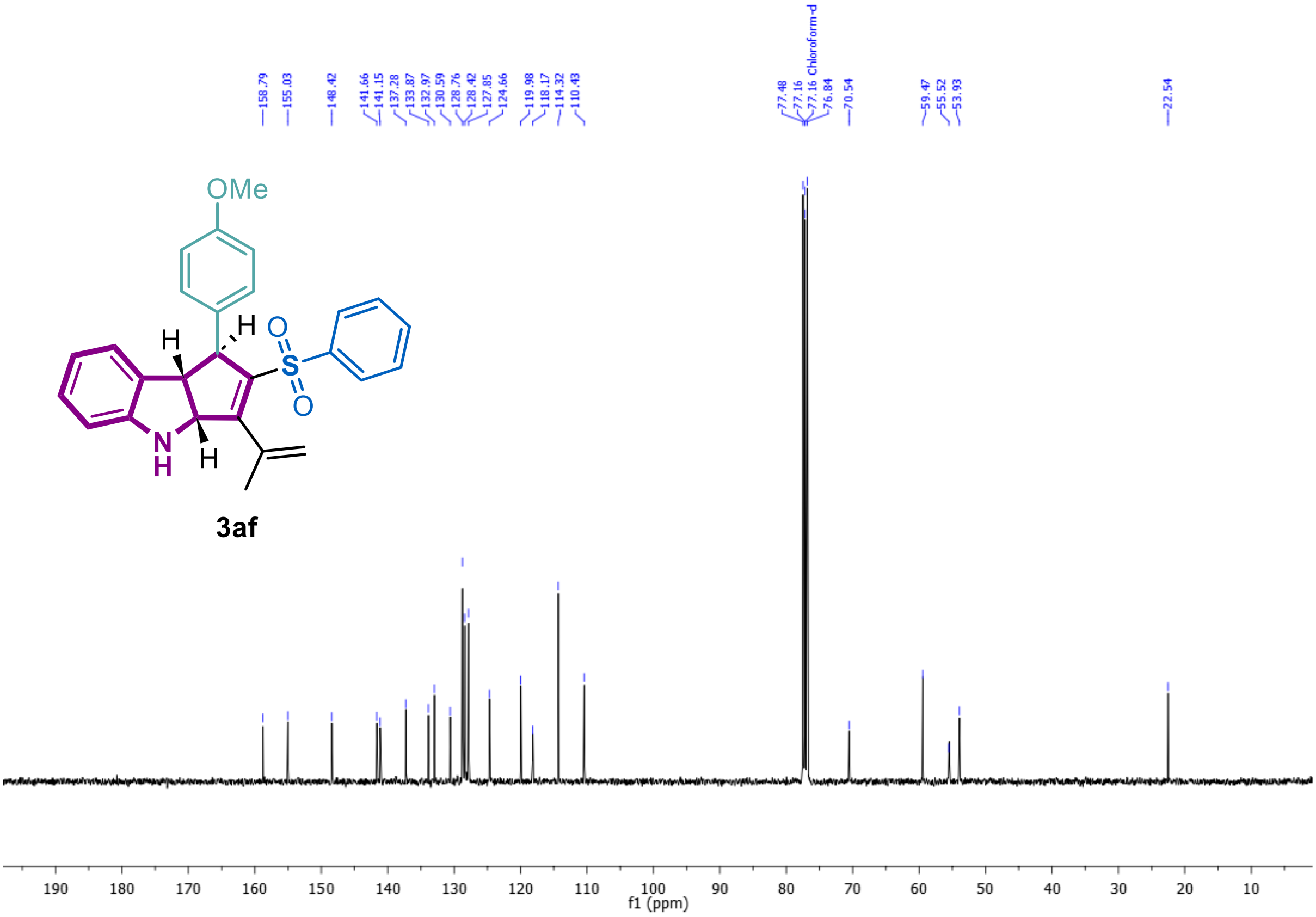

Figure S110: *^{13}C-NMR of **3af** in $CDCl_3$ (101 MHz)*

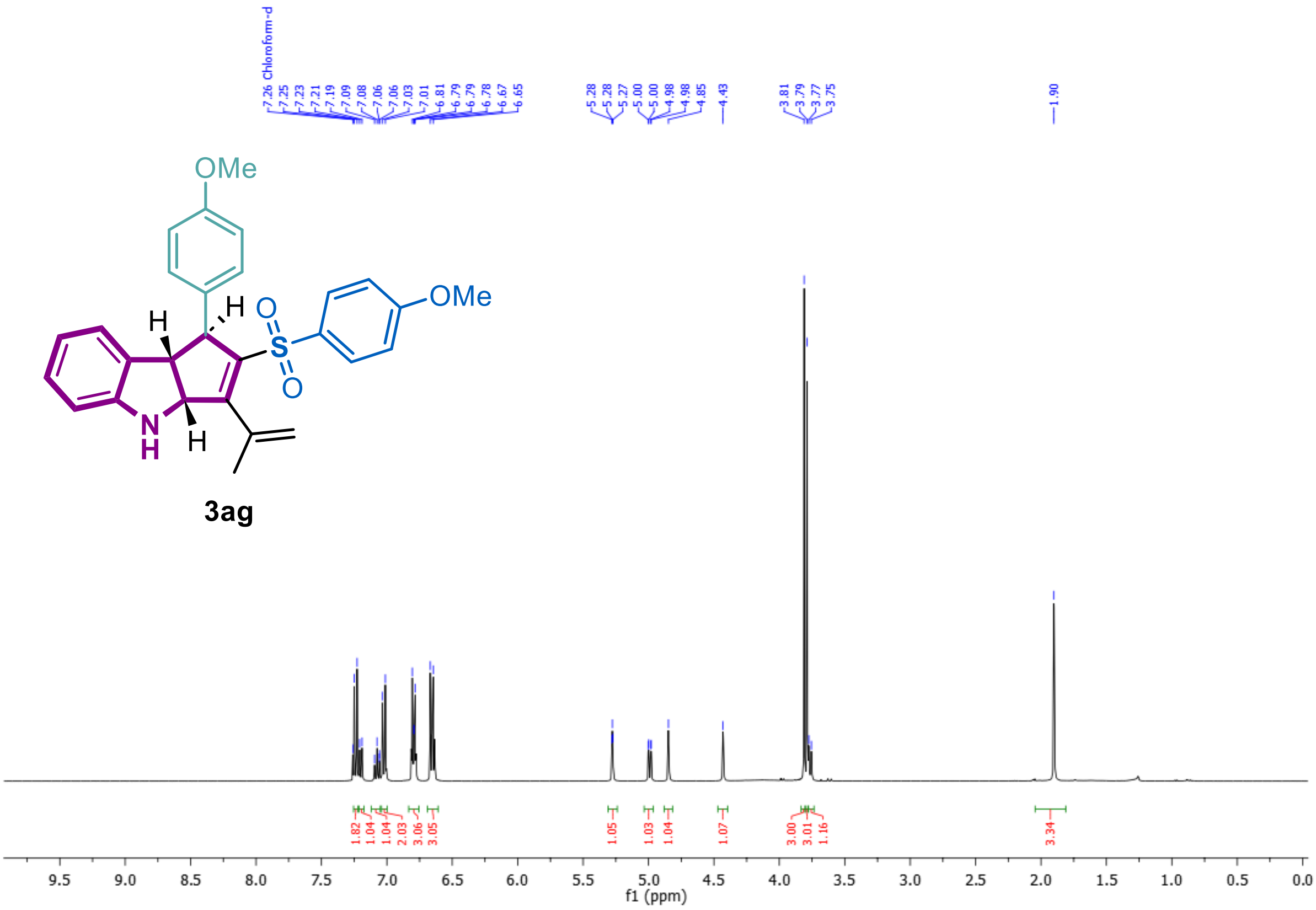

Figure S111: *^{1}H-NMR of **3ag** in $CDCl_3$ (400 MHz)*

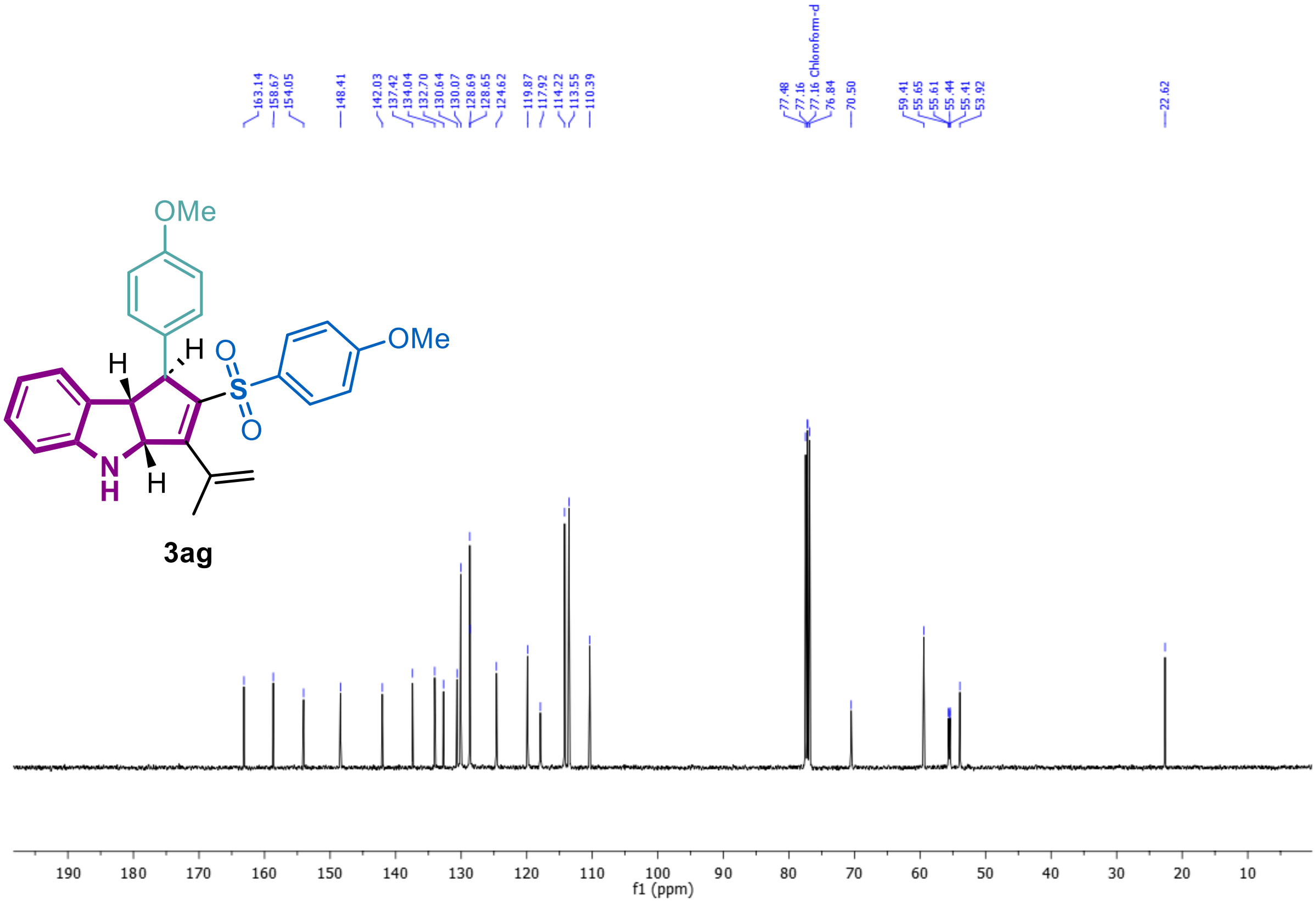

Figure S112: ^{13}C-NMR of ***3ag*** *in* $CDCl_3$ *(101 MHz)*

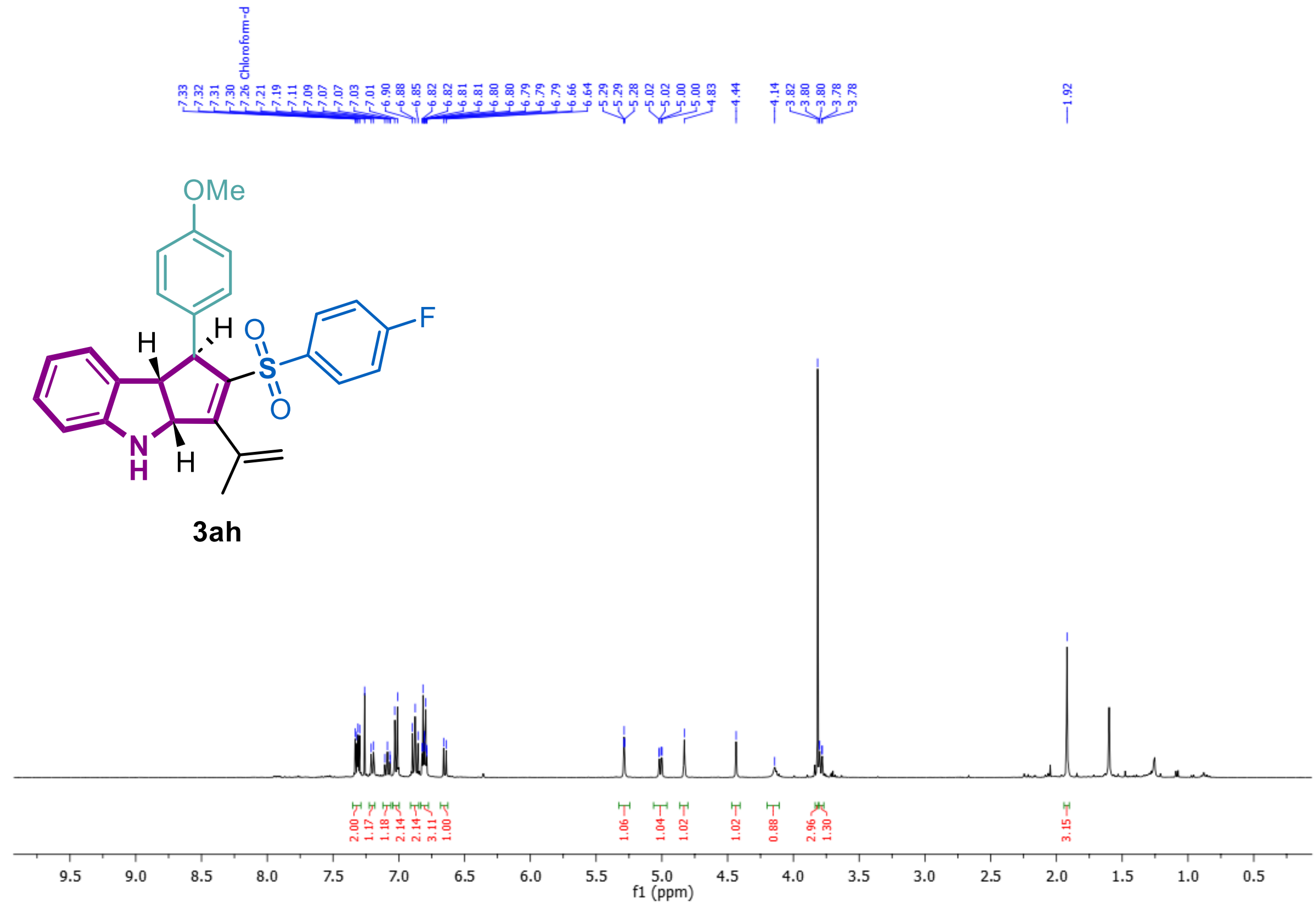

Figure S113: ^{1}H-NMR of ***3ah*** *in* $CDCl_3$ *(400 MHz)*

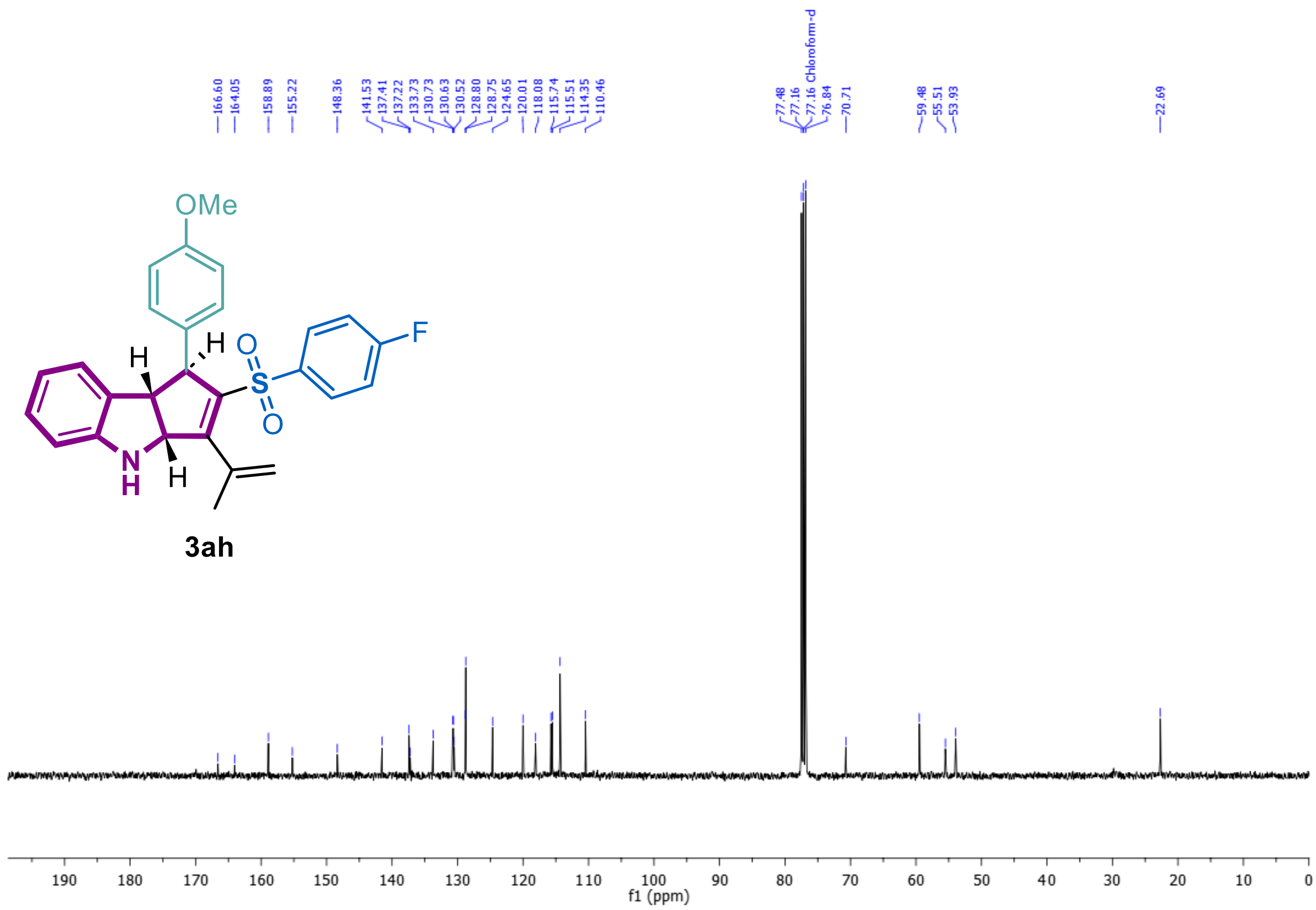

Figure S114: *^{13}C-NMR of **3ah** in $CDCl_3$ (101 MHz)*

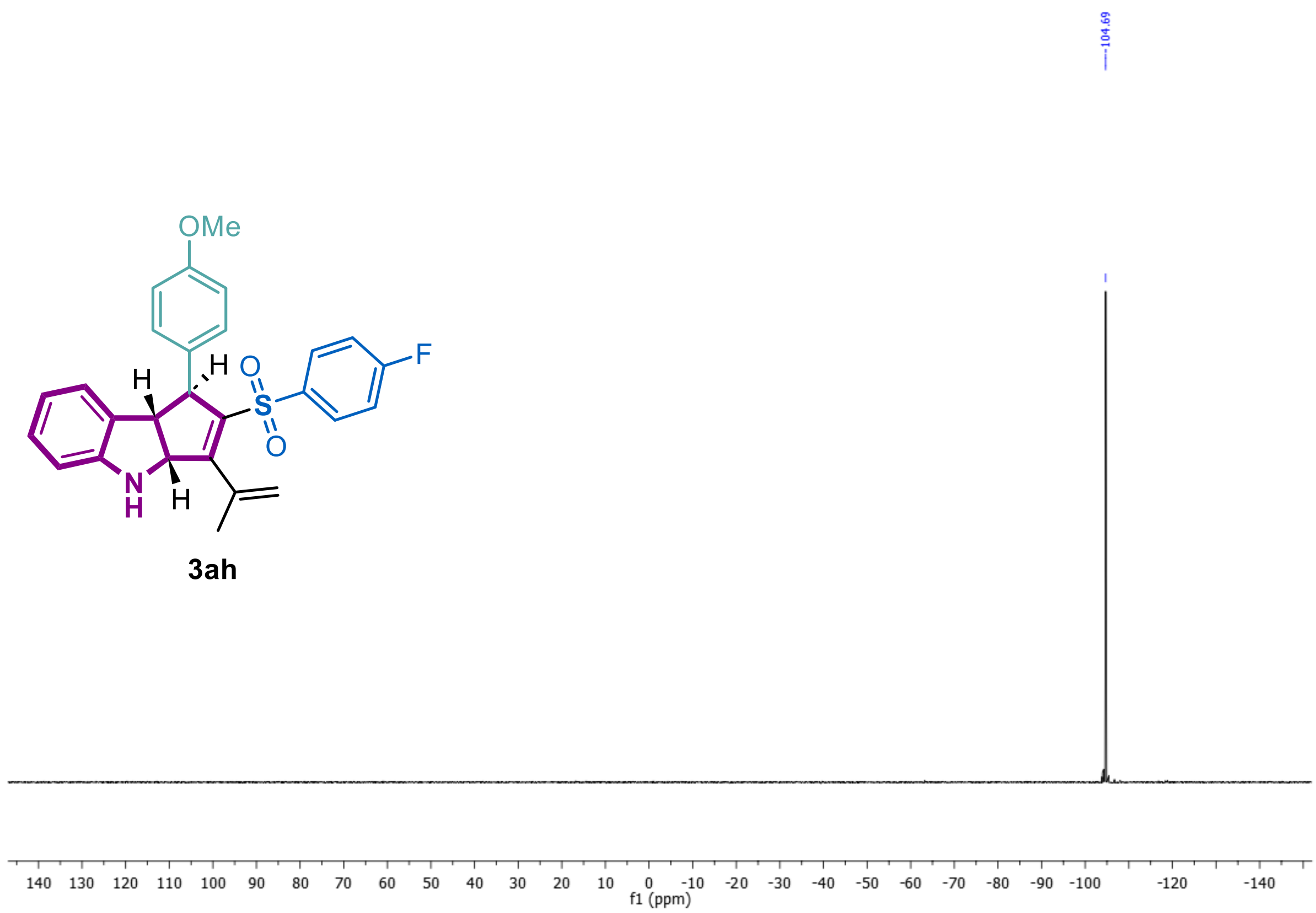

Figure S115: *^{19}F-NMR of **3ah** in $CDCl_3$ (376 MHz)*

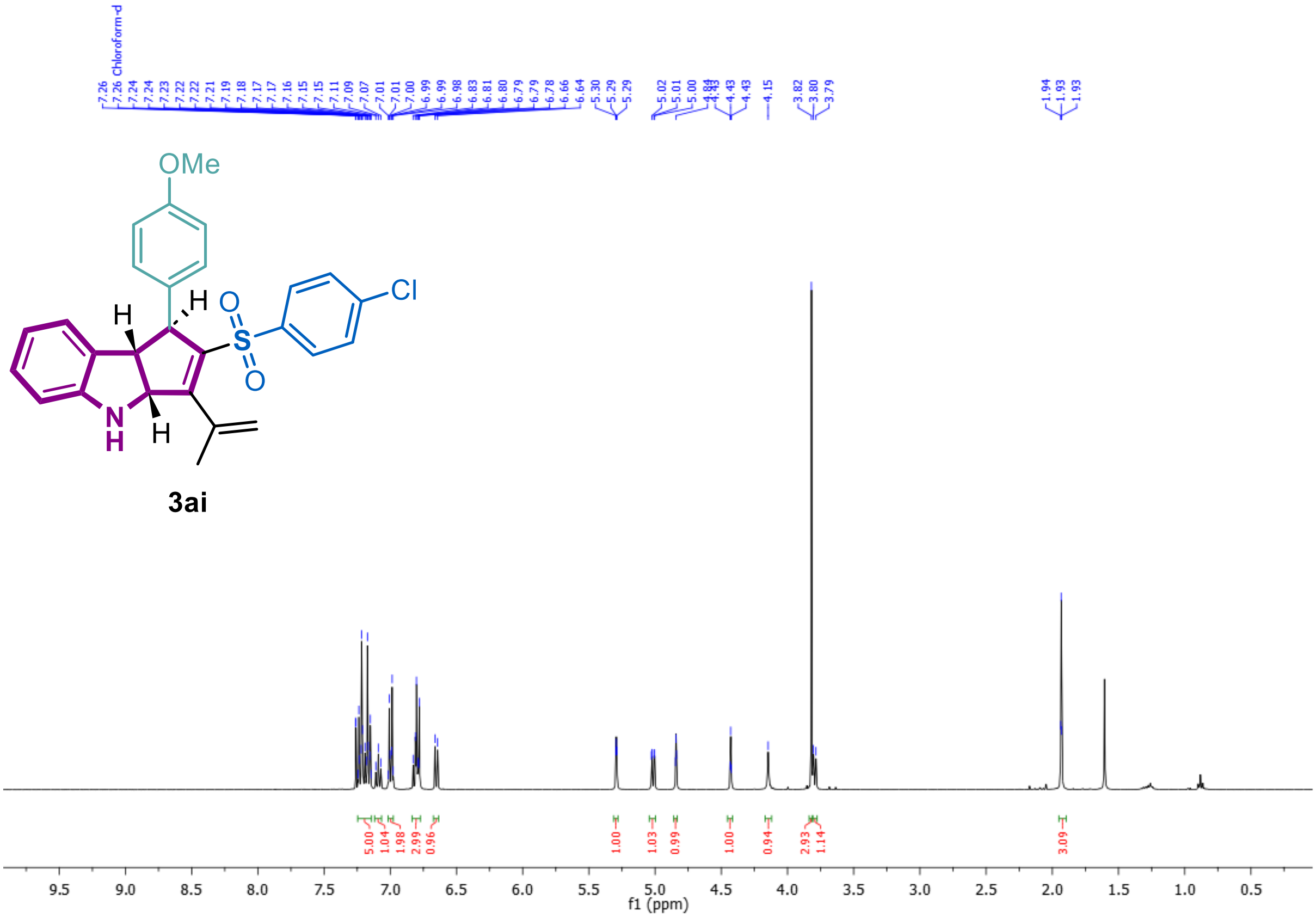

Figure S116: ^{1}H-NMR of **3ai** in $CDCl_3$ (400 MHz)

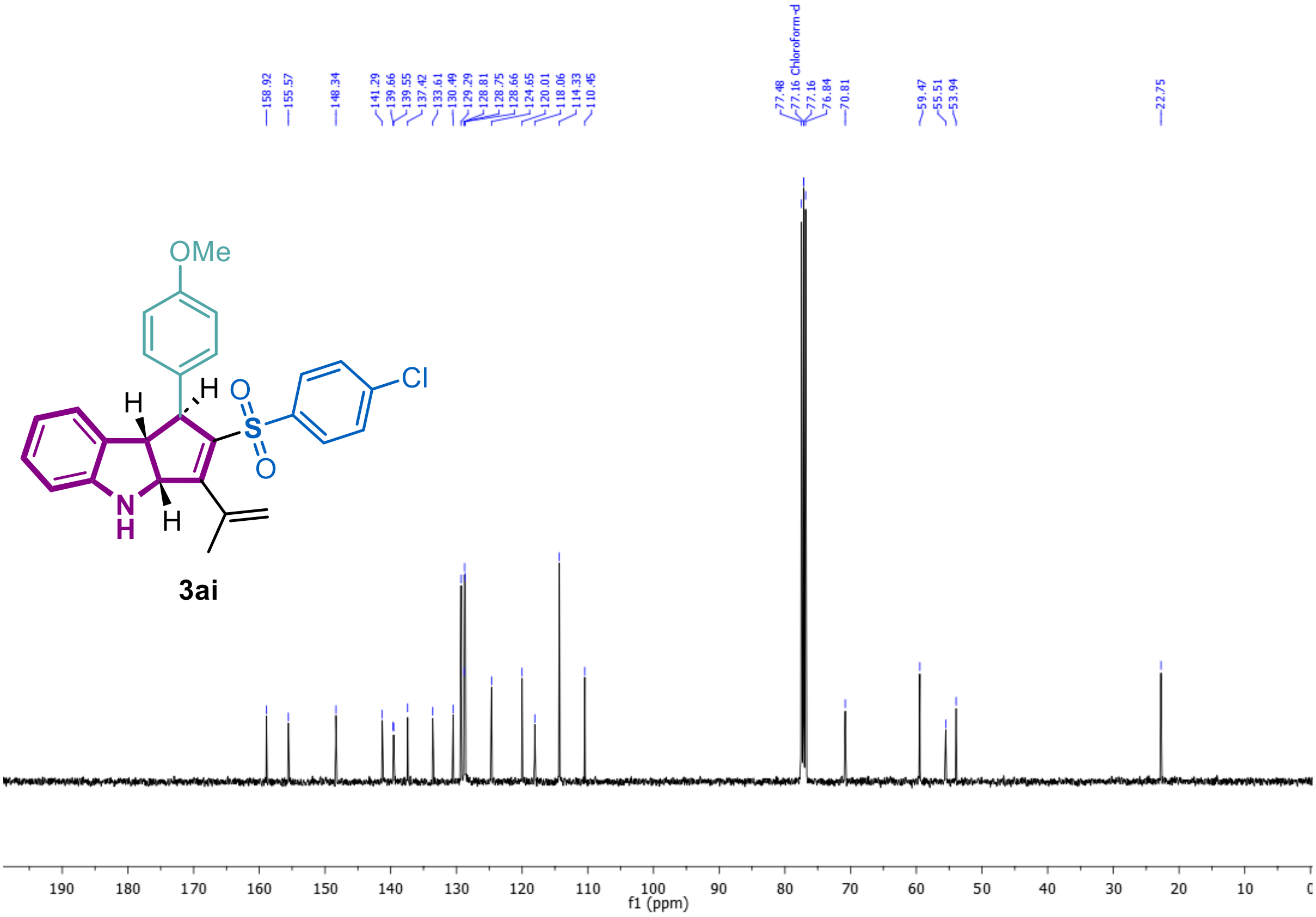

Figure S117: ^{13}C-NMR of **3ai** in $CDCl_3$ (101 MHz)

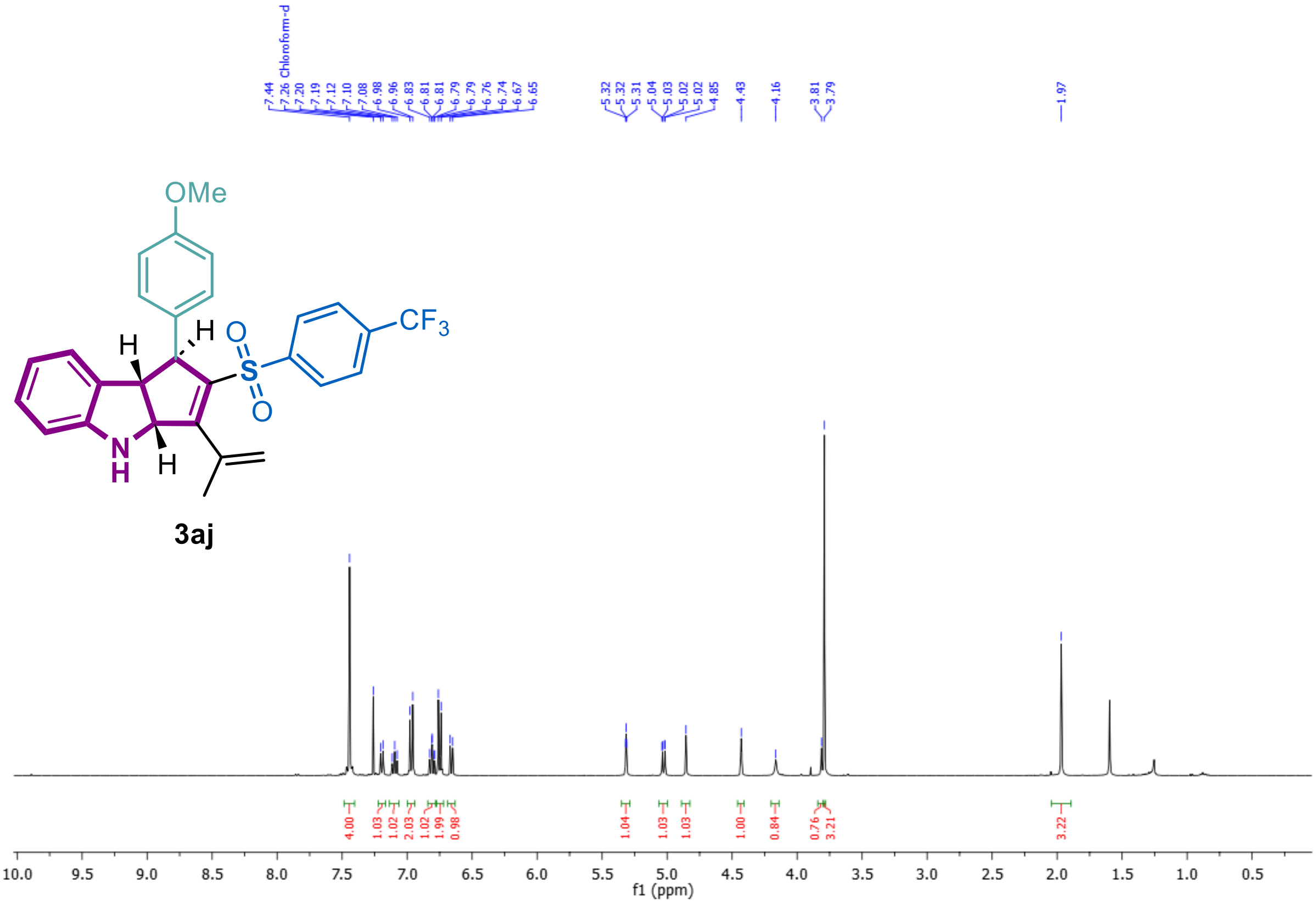

Figure S118: ^{1}H-NMR of **3aj** in $CDCl_3$ (400 MHz)

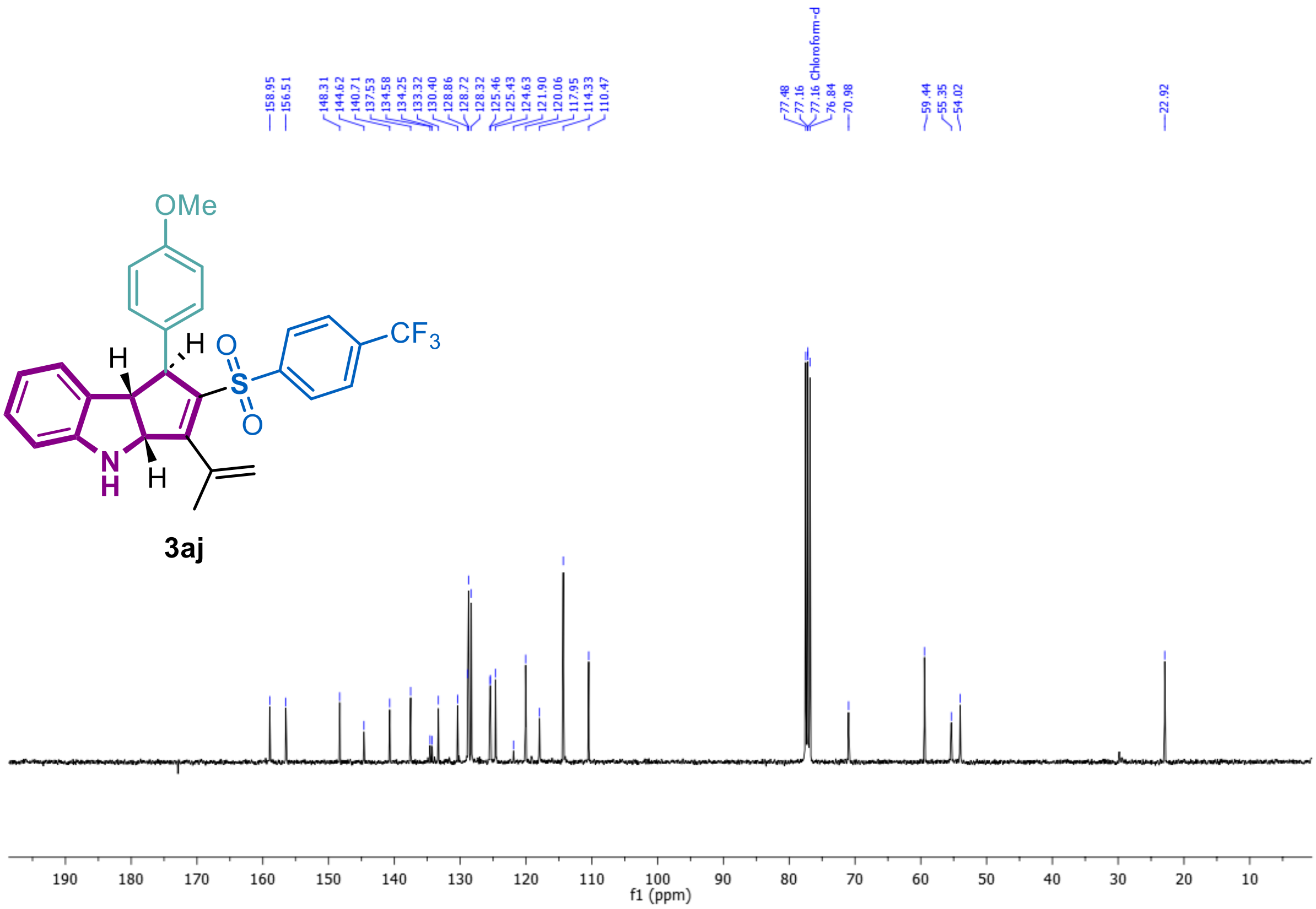

Figure S119: ^{13}C-NMR of **3aj** in $CDCl_3$ (101 MHz)

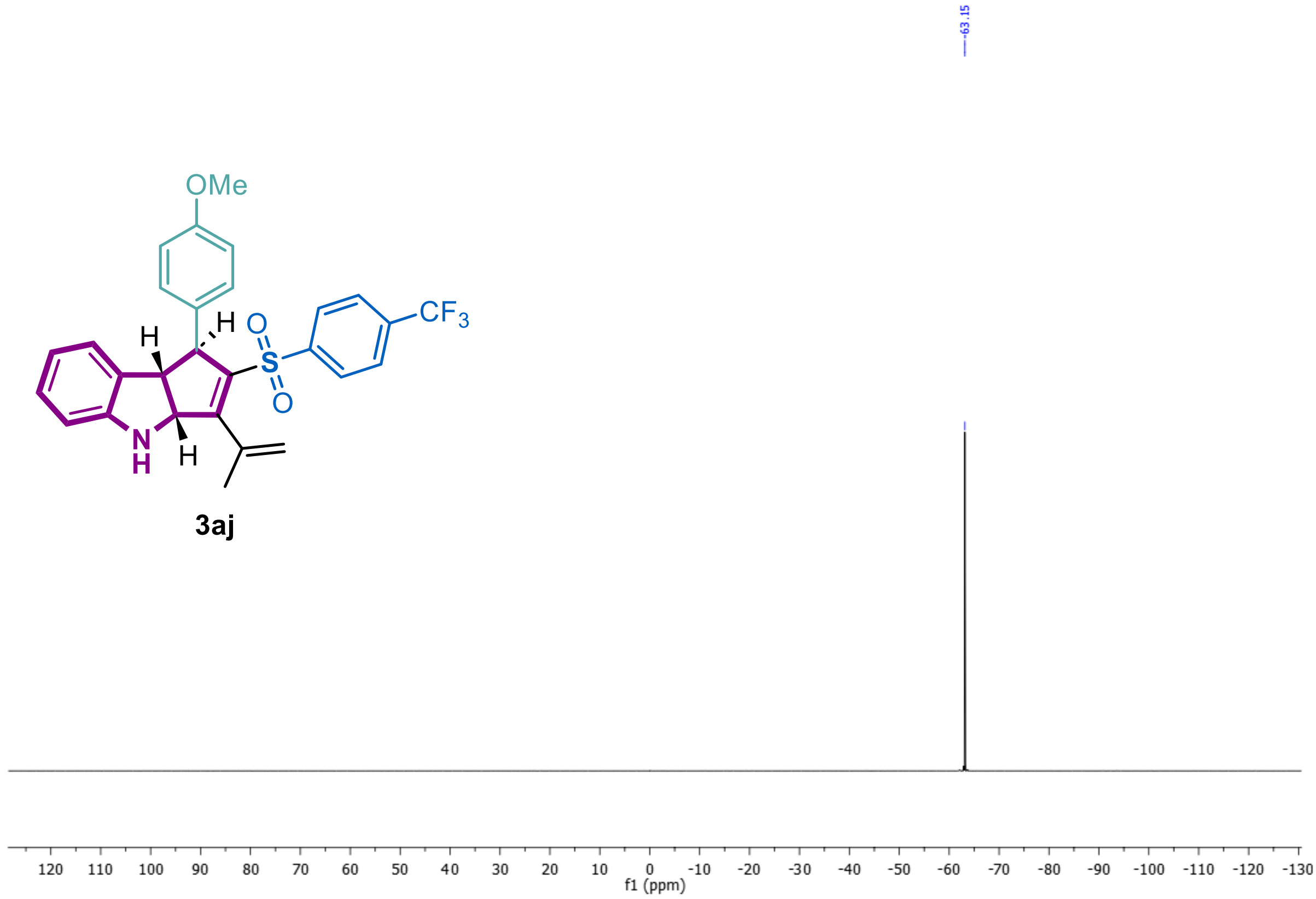

Figure S120: ^{19}F-NMR of ***3aj*** in $CDCl_3$ (376 MHz)

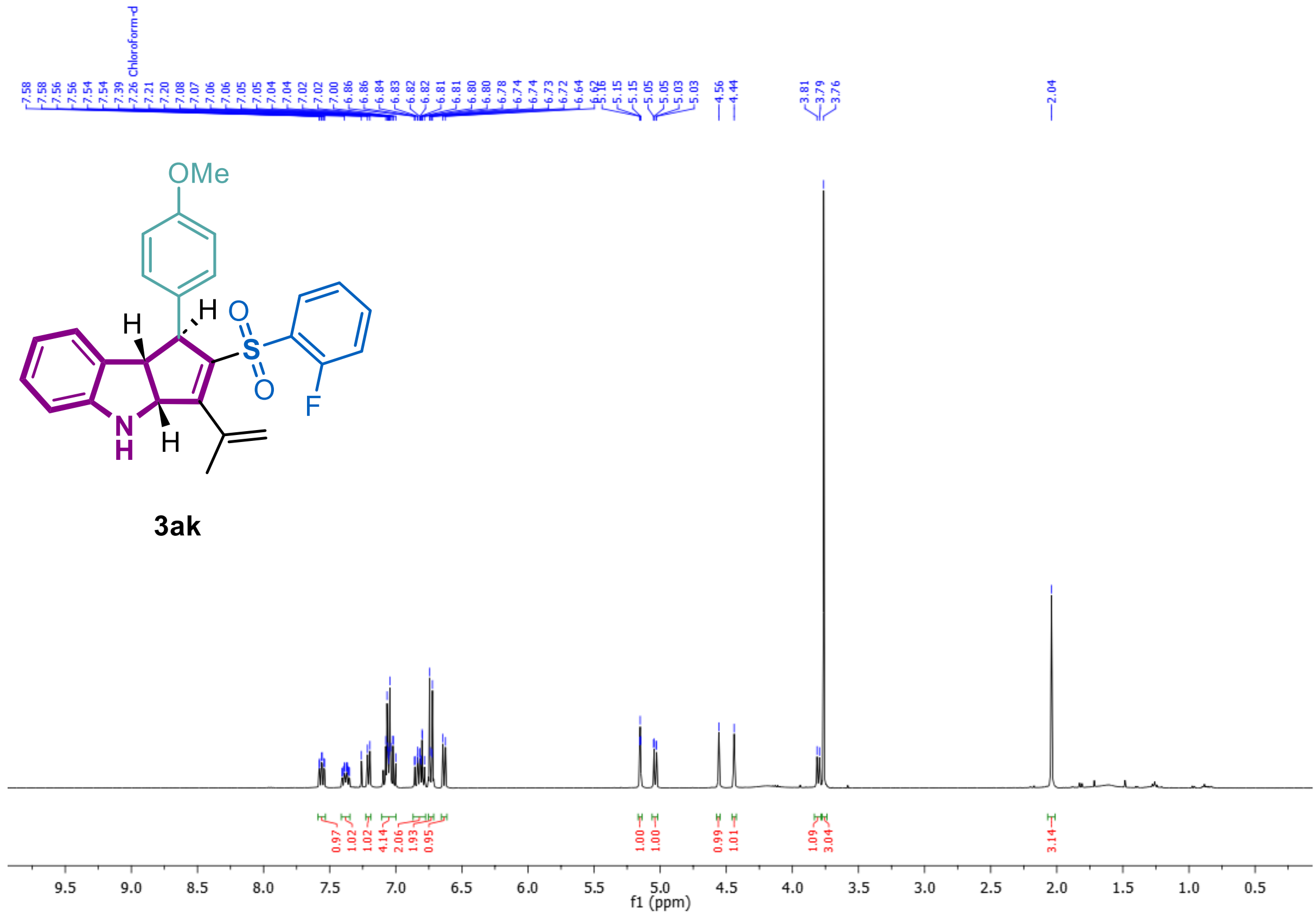

Figure S121: ^{1}H-NMR of ***3ak*** in $CDCl_3$ (400 MHz)

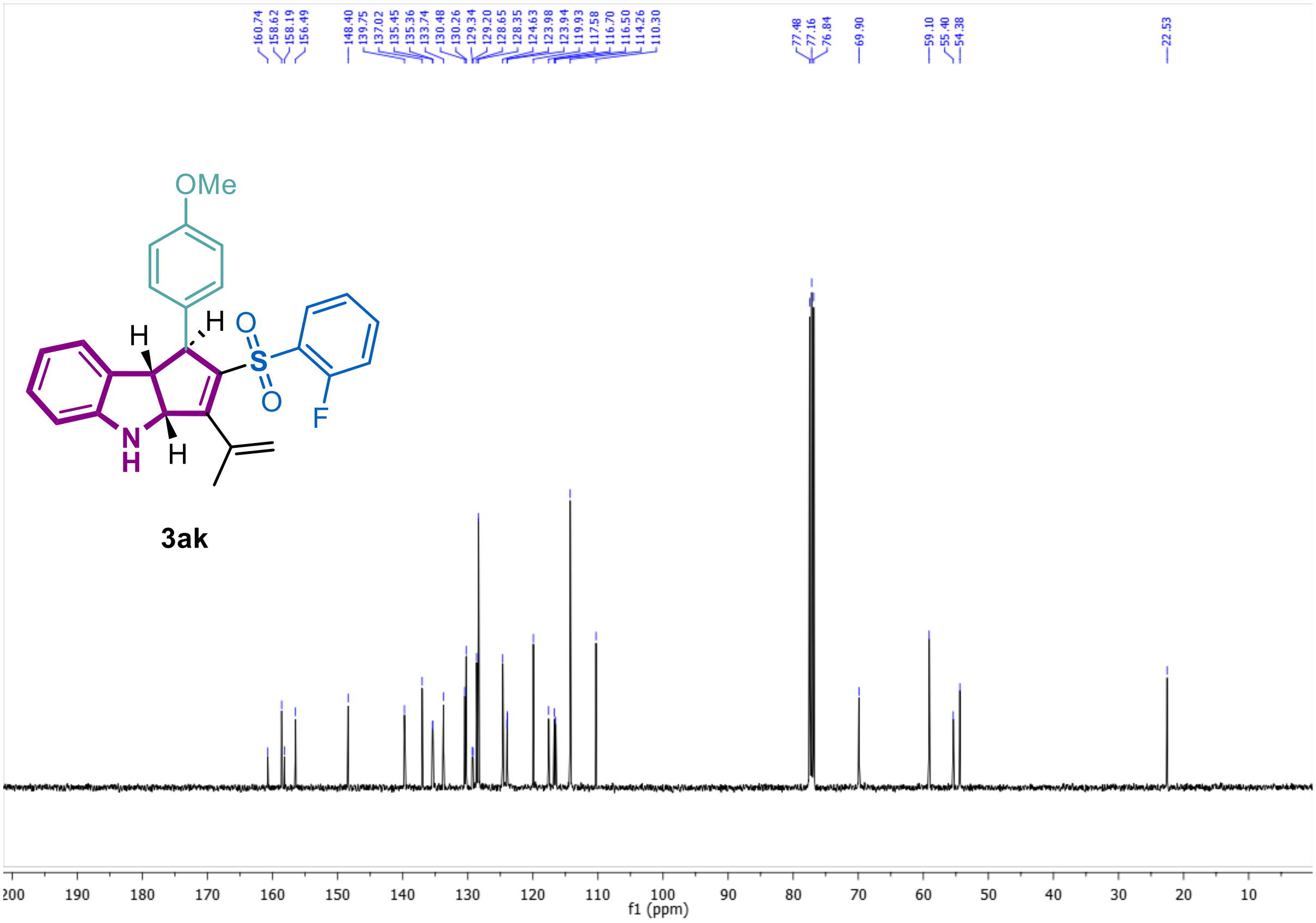

Figure S122: *^{13}C-NMR of* ***3ak*** *in $CDCl_3$ (101 MHz)*

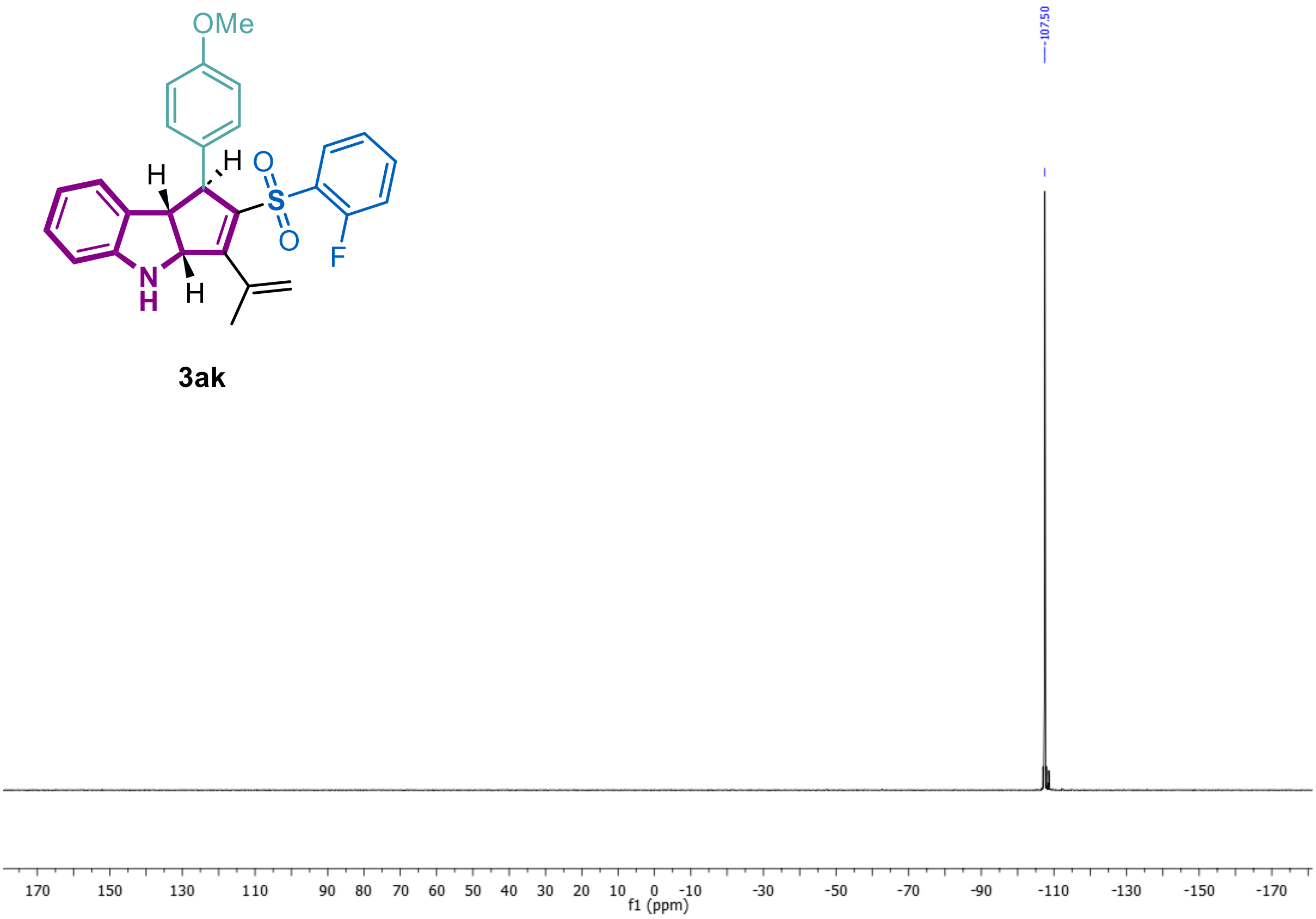

Figure S123: *^{19}F-NMR of* ***3aj*** *in $CDCl_3$ (376 MHz)*

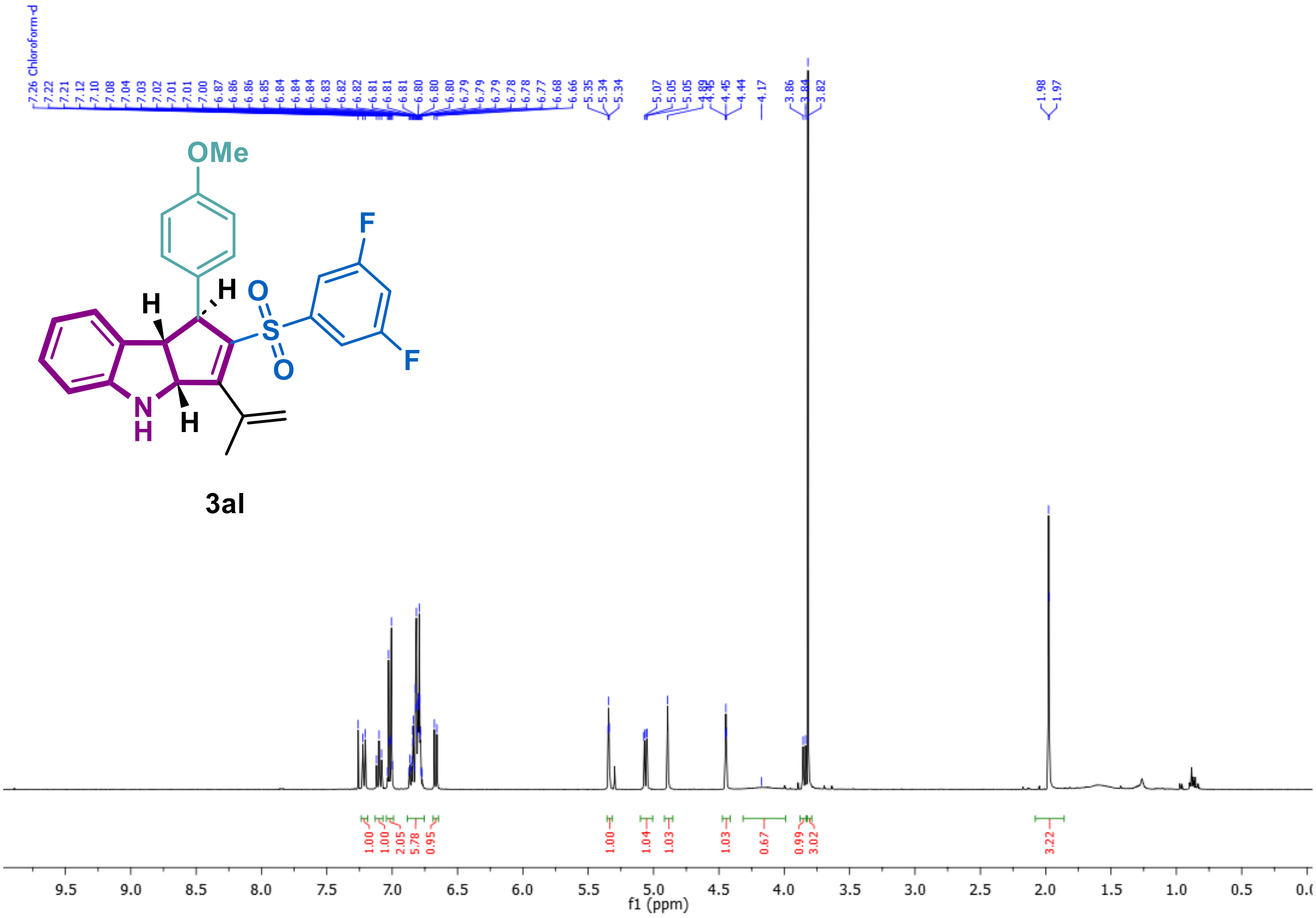

Figure S124: ^{1}H-NMR of **3al** in $CDCl_3$ (400 MHz)

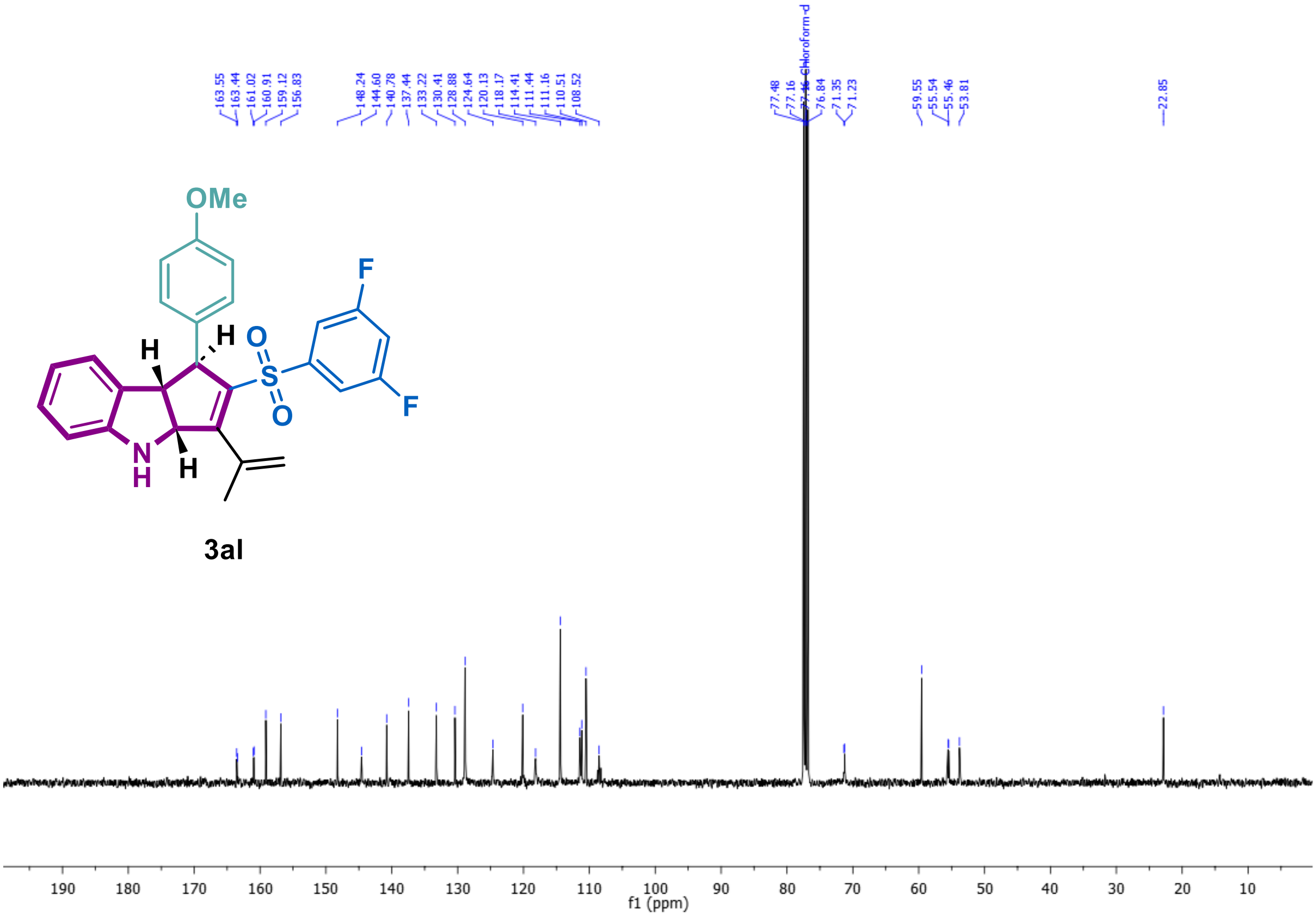

Figure S125: ^{13}C-NMR of **3al** in $CDCl_3$ (101 MHz)

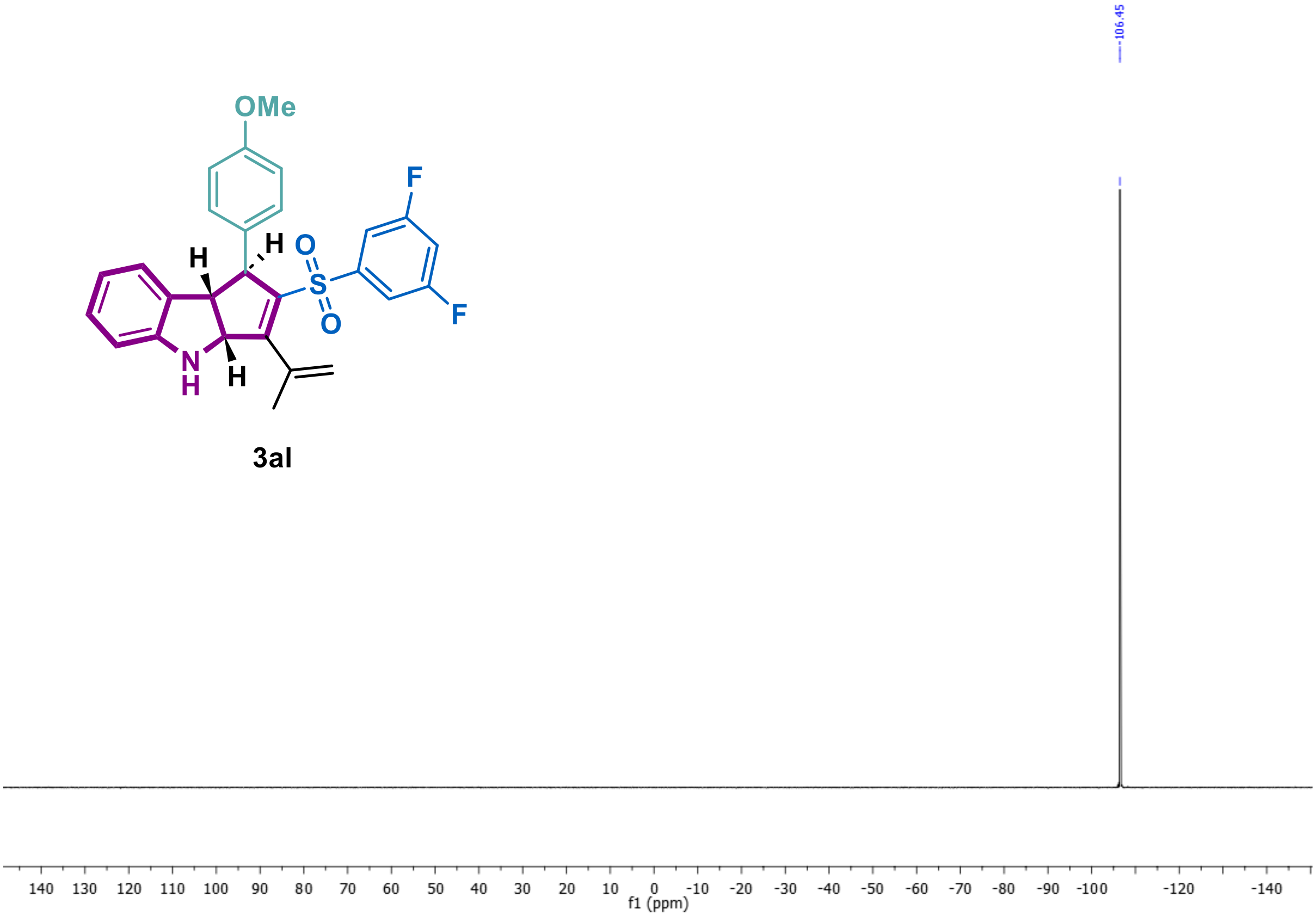

Figure S126: *^{19}F-NMR of* ***3al*** *in $CDCl_3$ (376 MHz)*

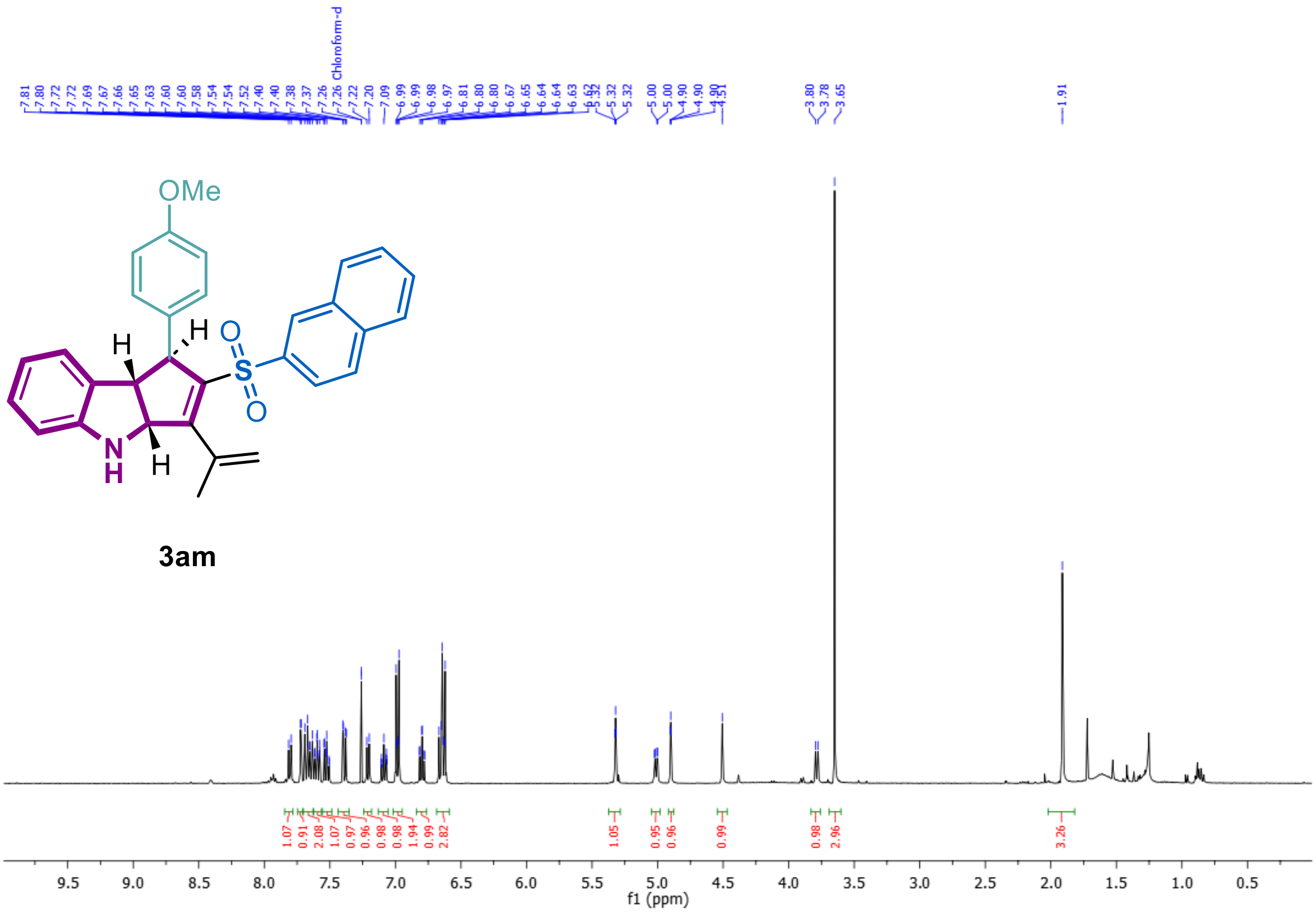

Figure S127: *^{1}H-NMR of* ***3am*** *in $CDCl_3$ (400 MHz)*

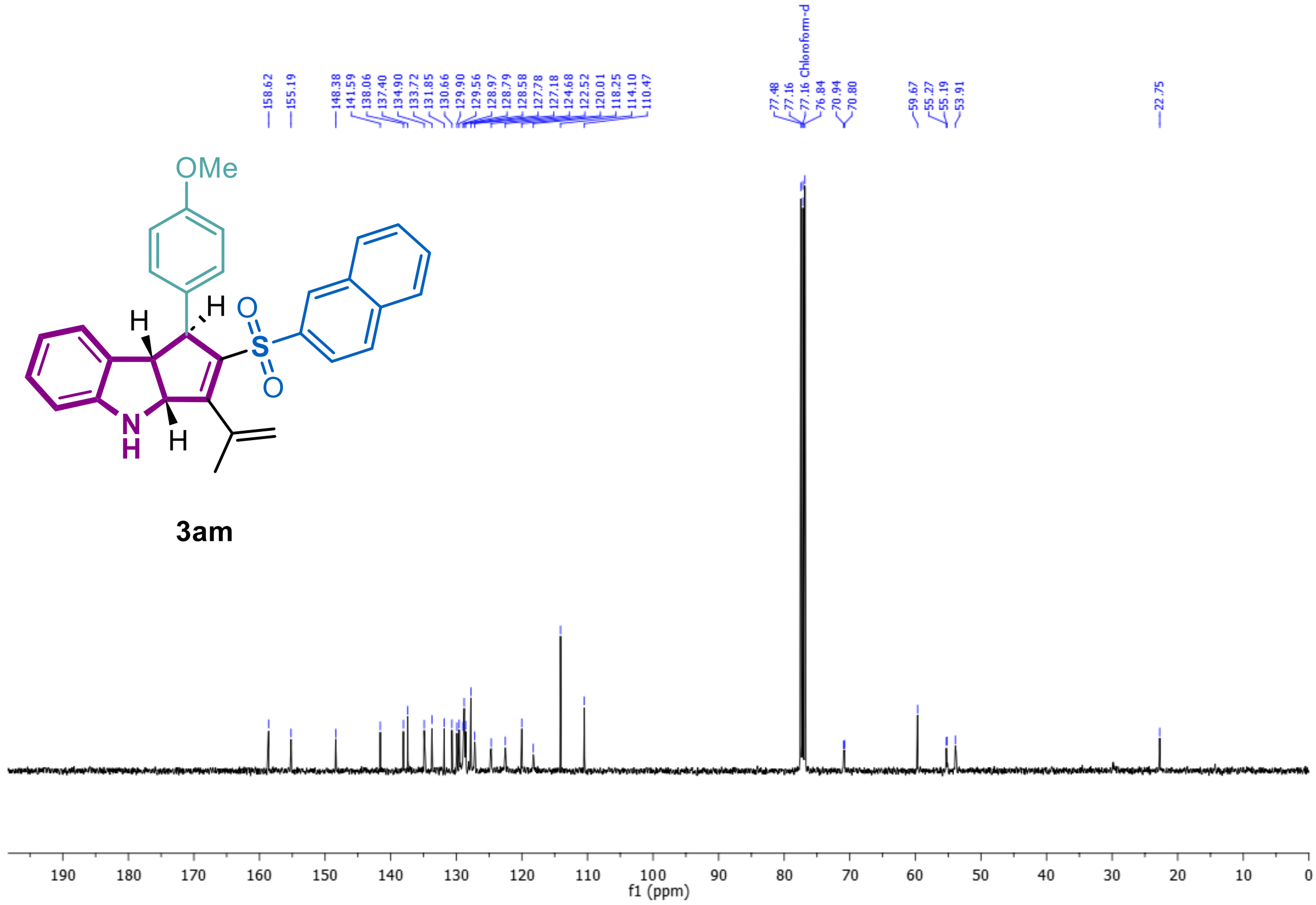

3am

Figure S128: ^{13}C-NMR of ***3am*** in $CDCl_3$ (101 MHz)

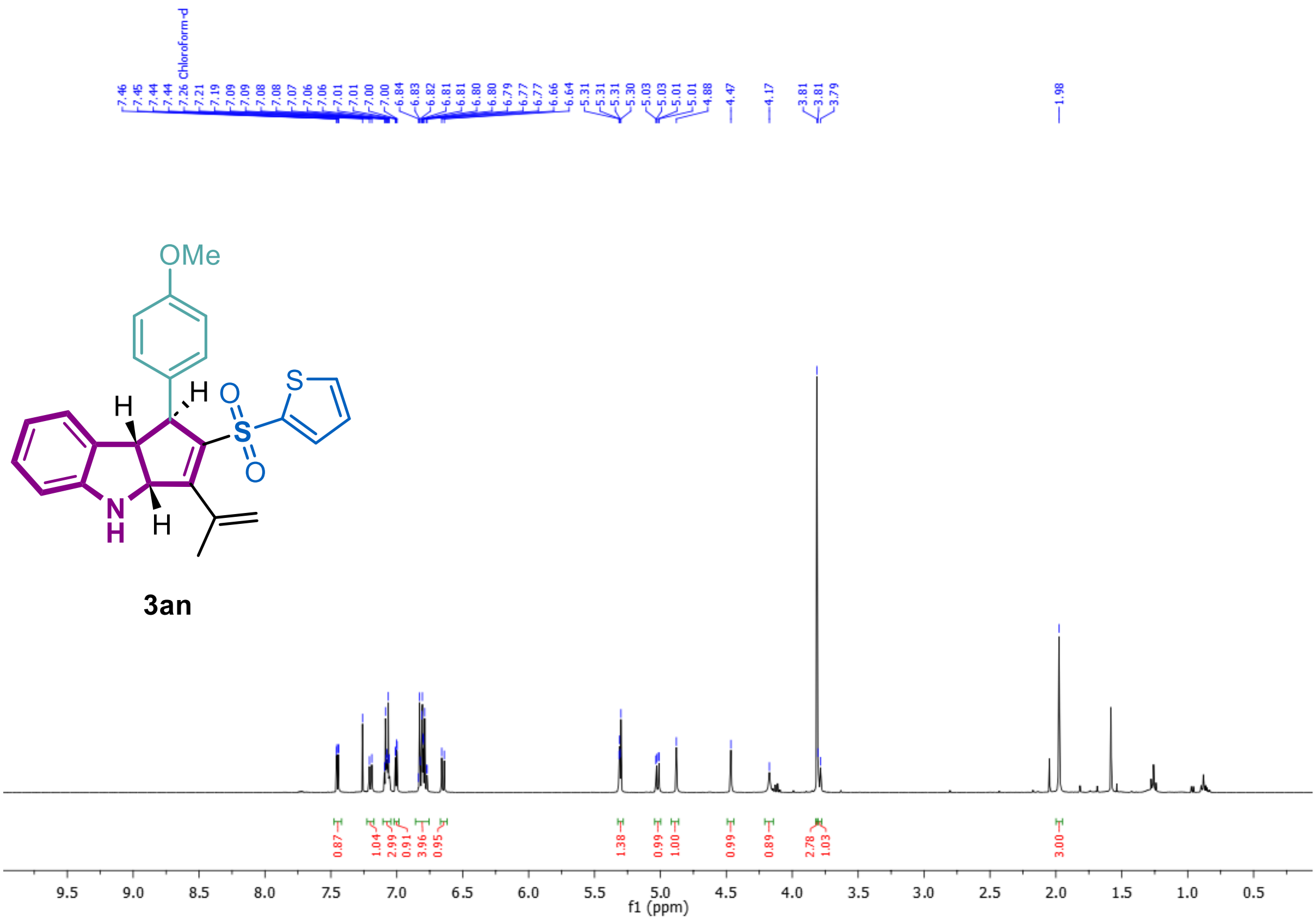

3an

Figure S129: ^{1}H-NMR of ***3an*** in $CDCl_3$ (400 MHz)

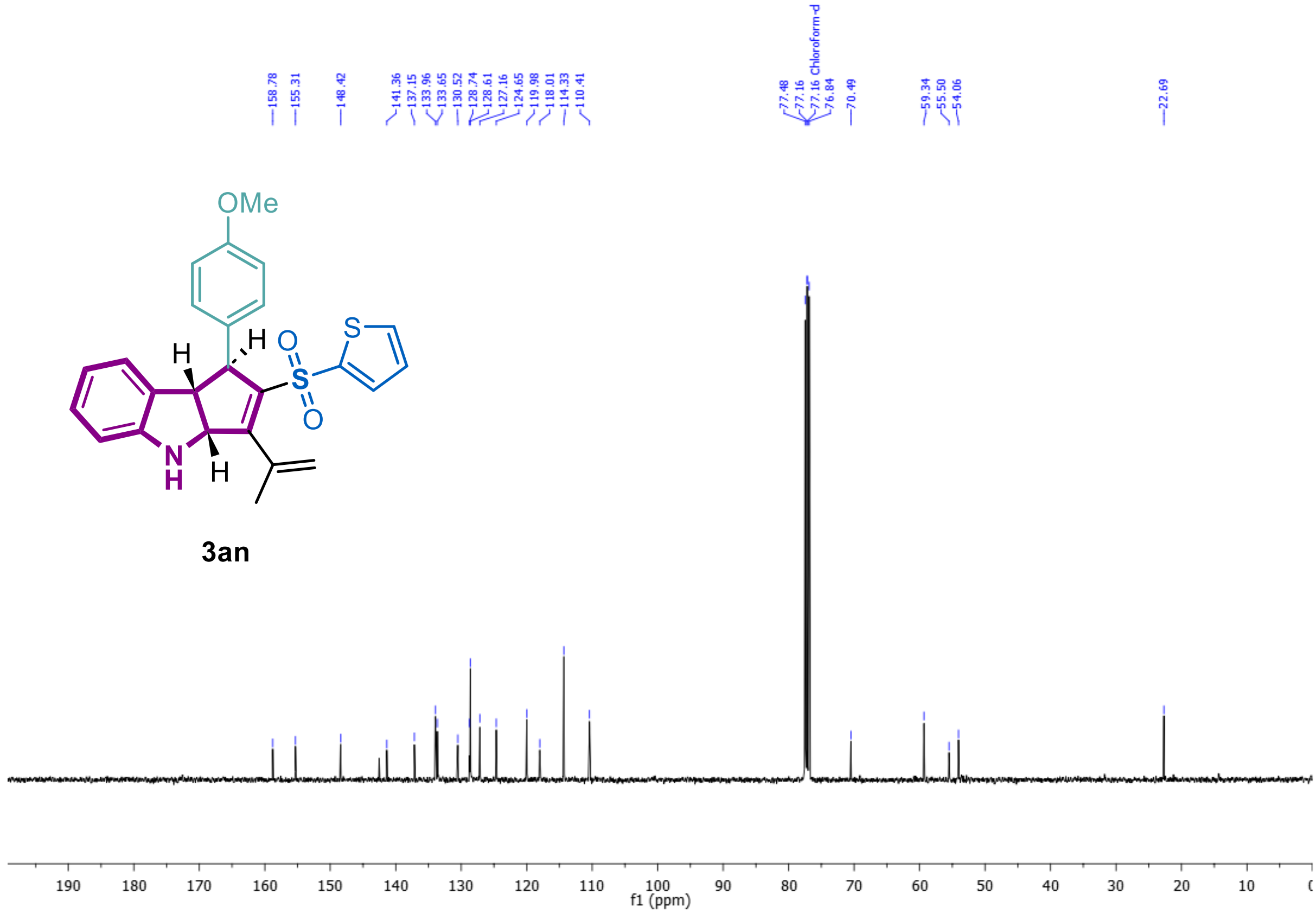

Figure S130: *^{13}C-NMR of **3an** in $CDCl_3$ (101 MHz)*

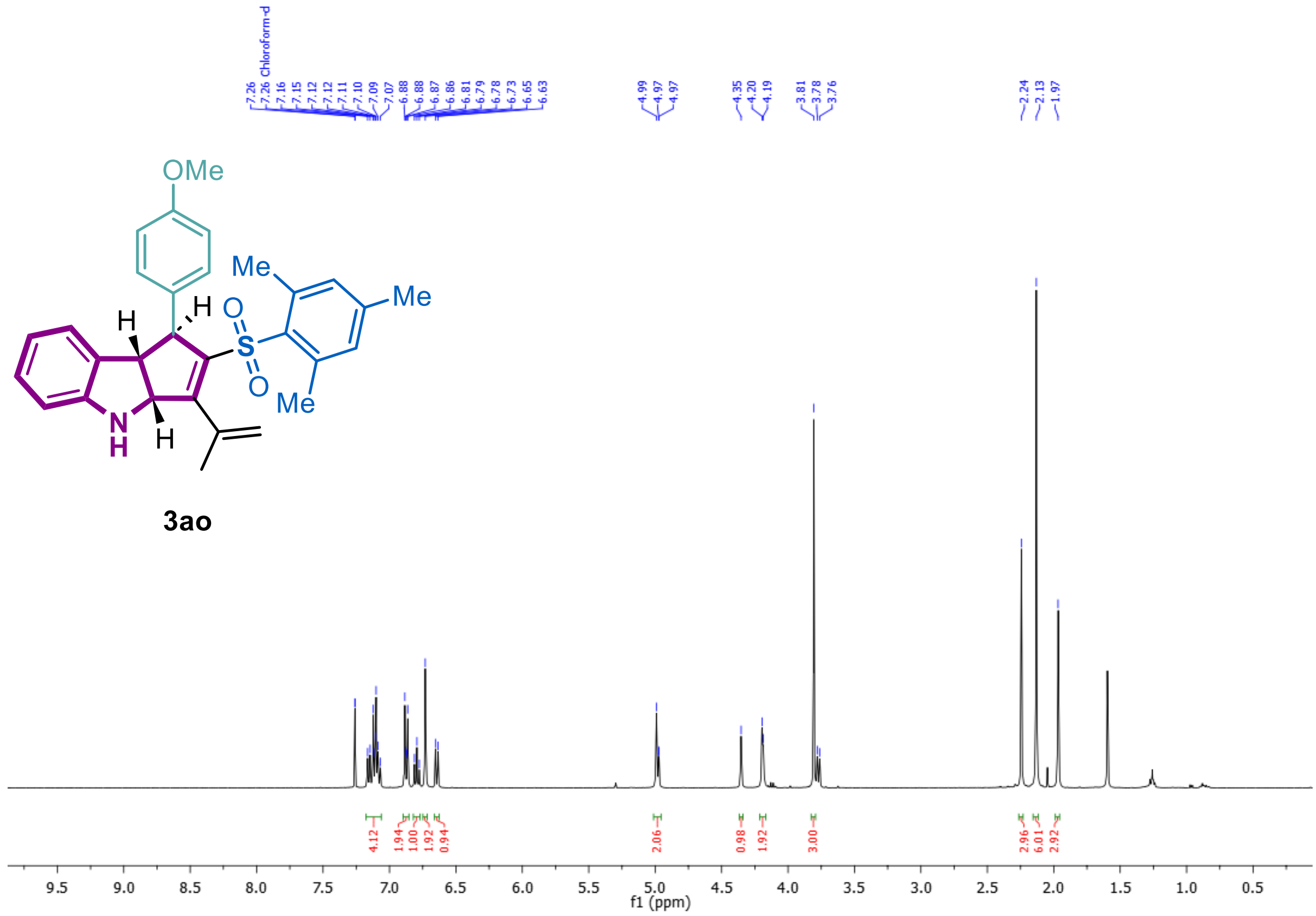

Figure S131: *^{1}H-NMR of **3ao** in $CDCl_3$ (400 MHz)*

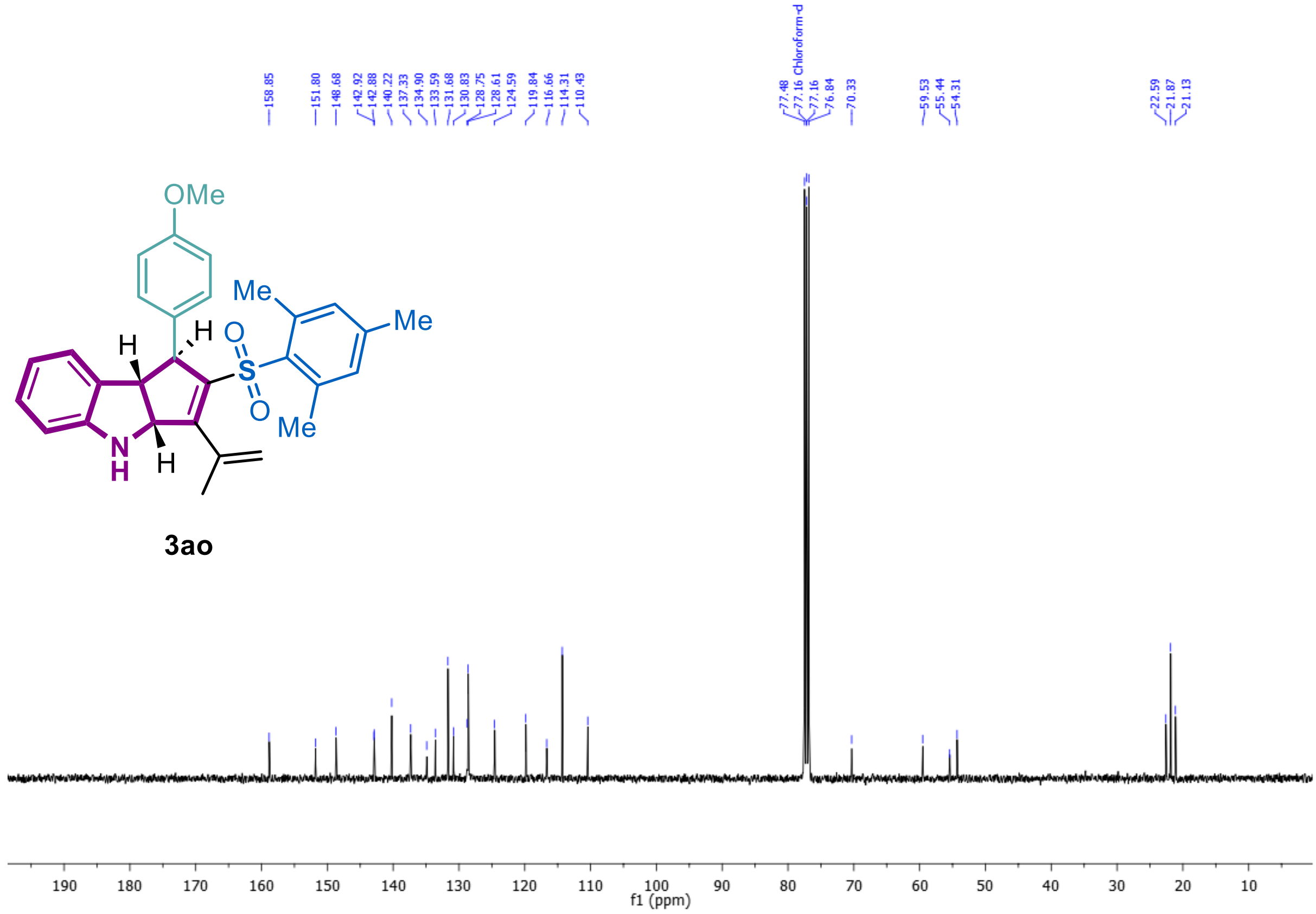

Figure S132: ^{13}C-NMR of **3ao** in $CDCl_3$ (101 MHz)

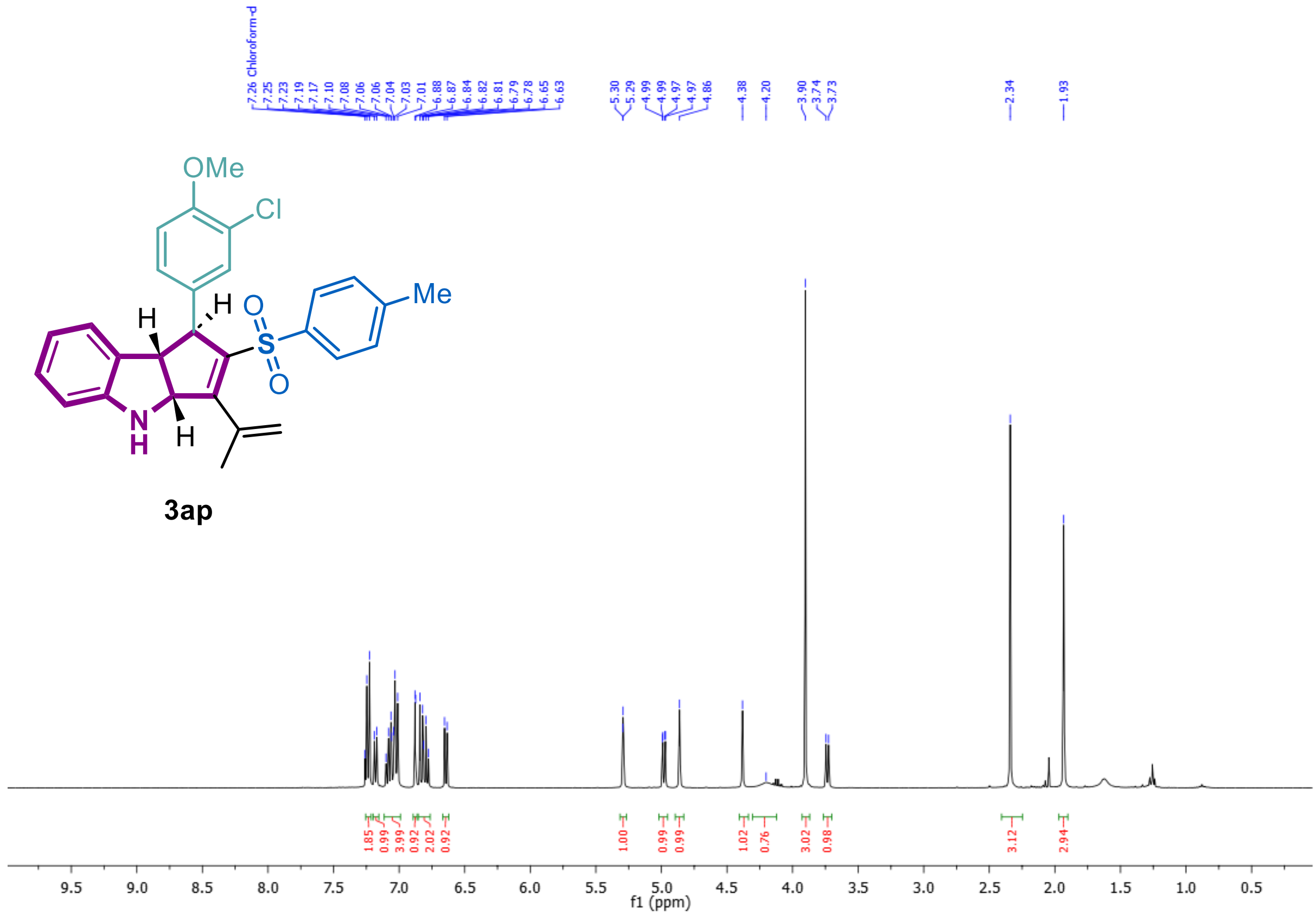

Figure S133: ^{1}H-NMR of **3ap** in $CDCl_3$ (400 MHz)

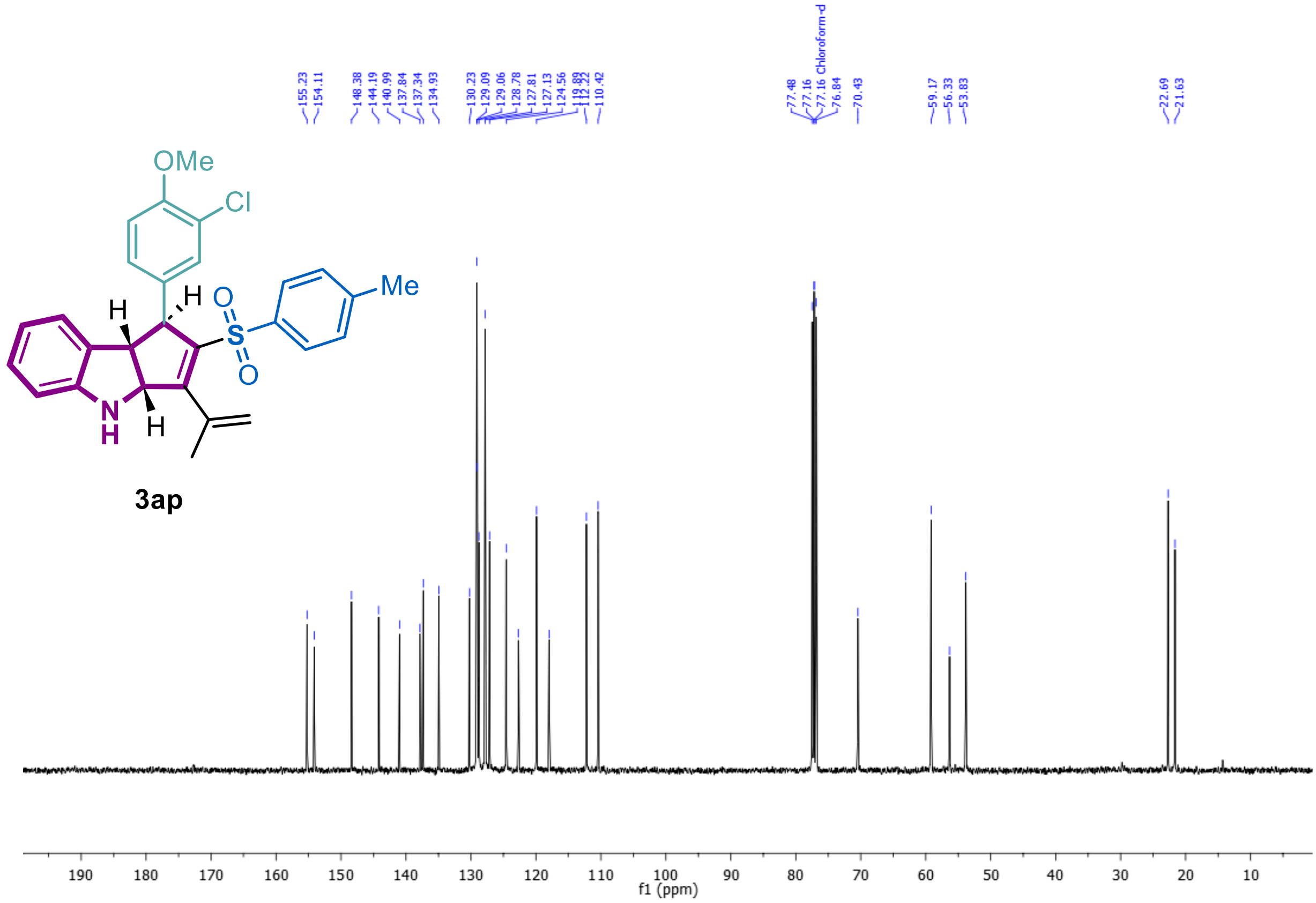

Figure S134: ^{13}C-NMR of **3ap** in $CDCl_3$ (101 MHz)

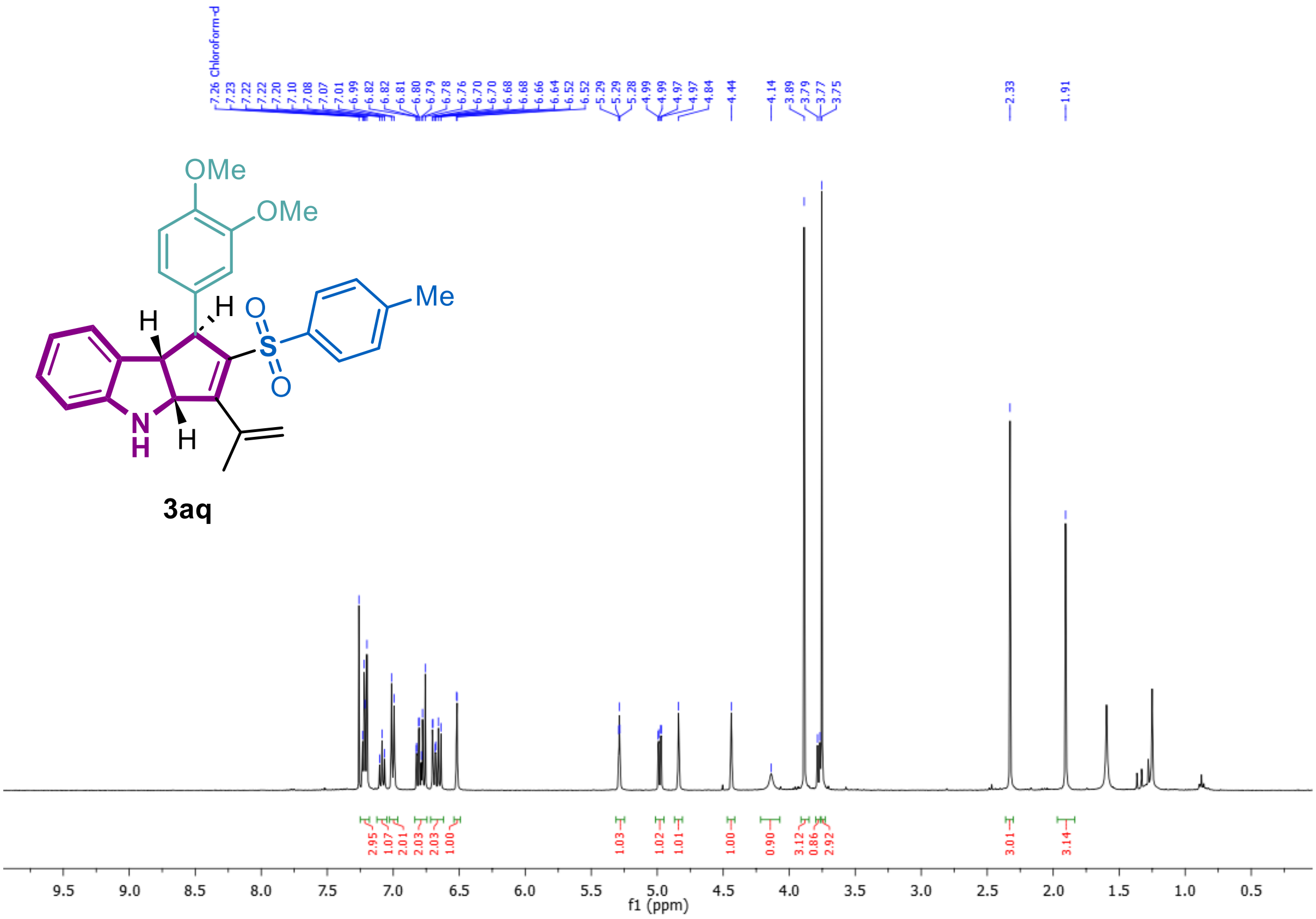

Figure S135: ^{1}H-NMR of **3aq** in $CDCl_3$ (400 MHz)

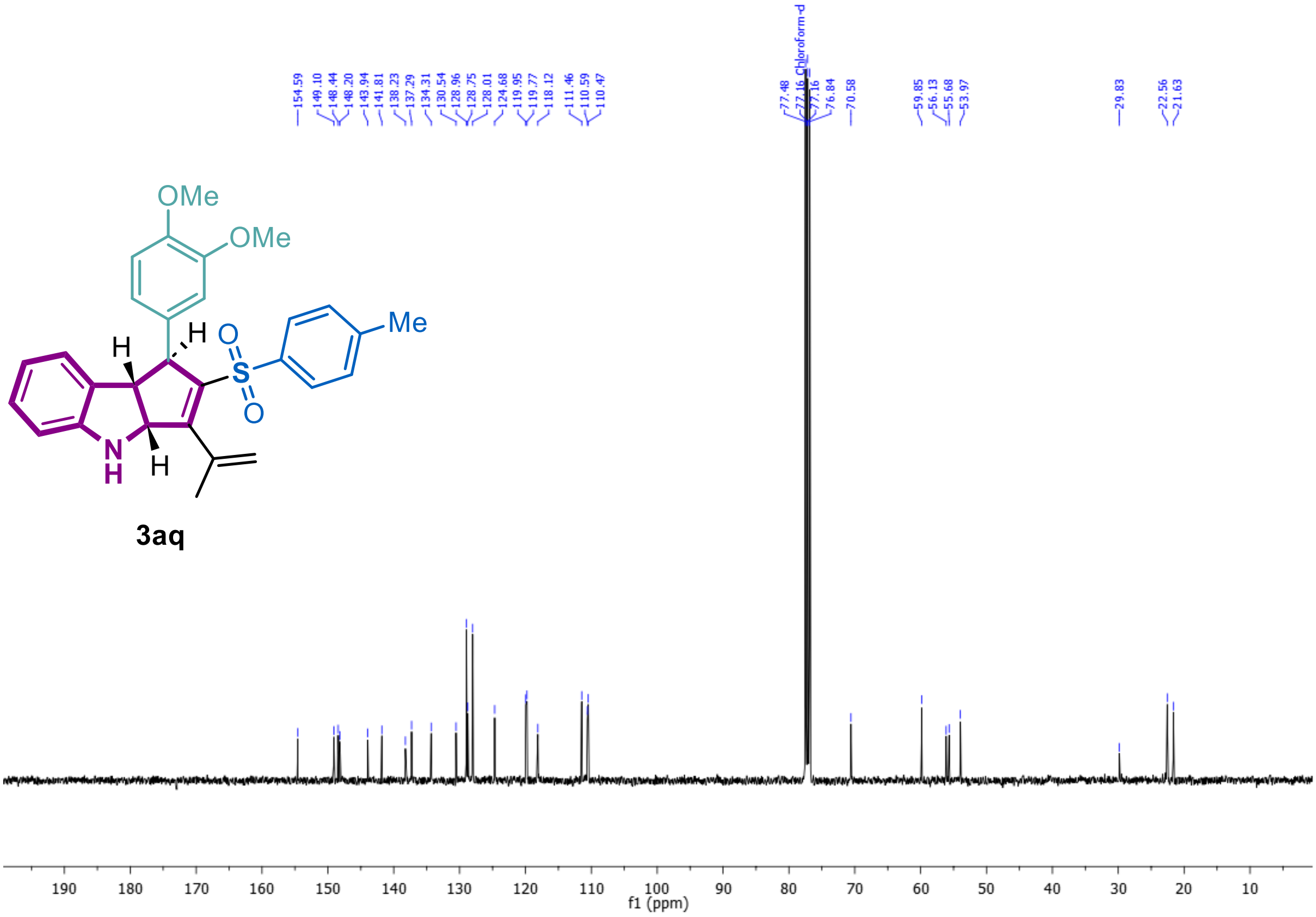

Figure S136: ^{13}C-NMR of ***3aq*** *in CDCl$_3$ (101 MHz)*

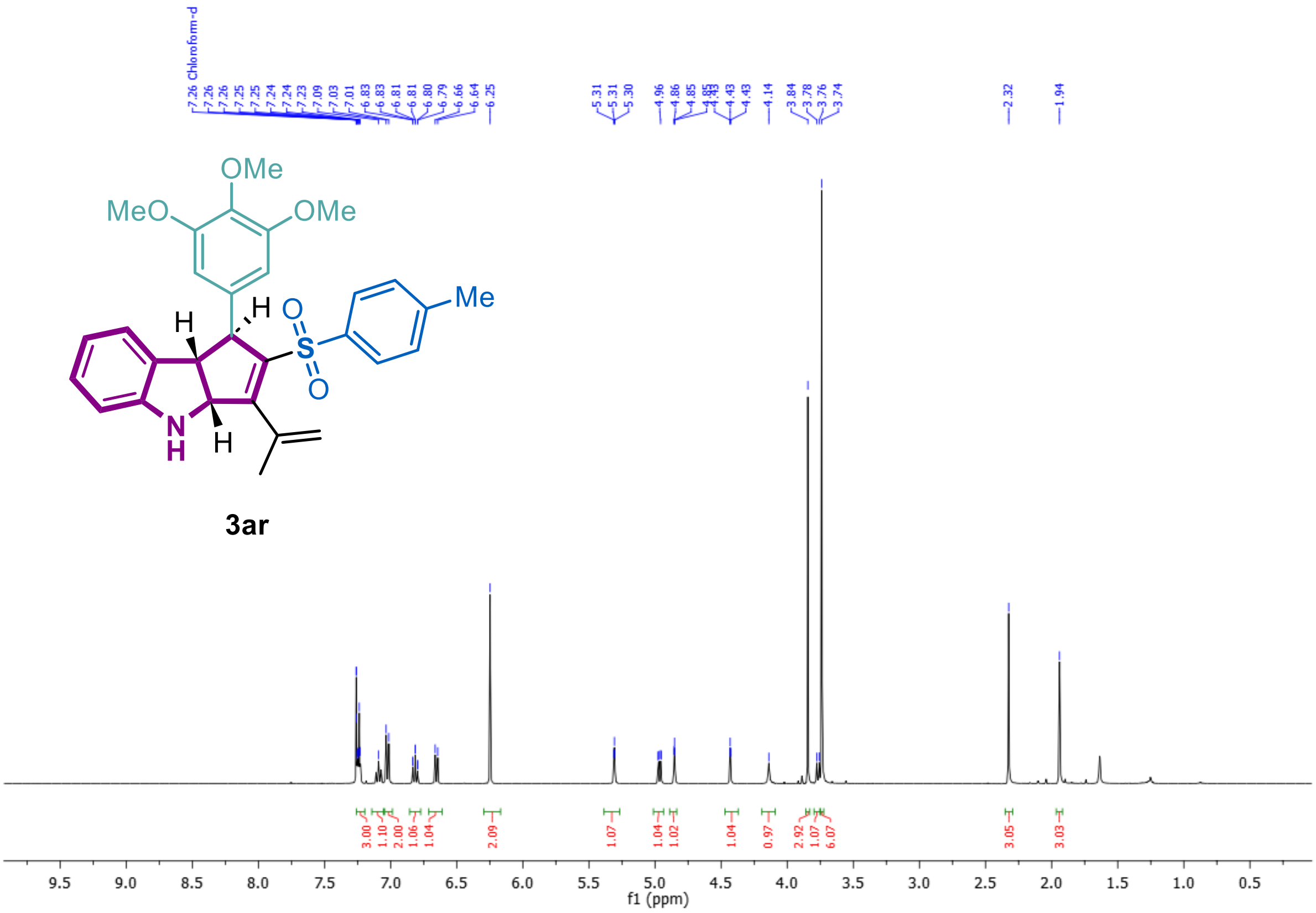

Figure S137: ^{1}H-NMR of ***3ar*** *in CDCl$_3$ (400 MHz)*

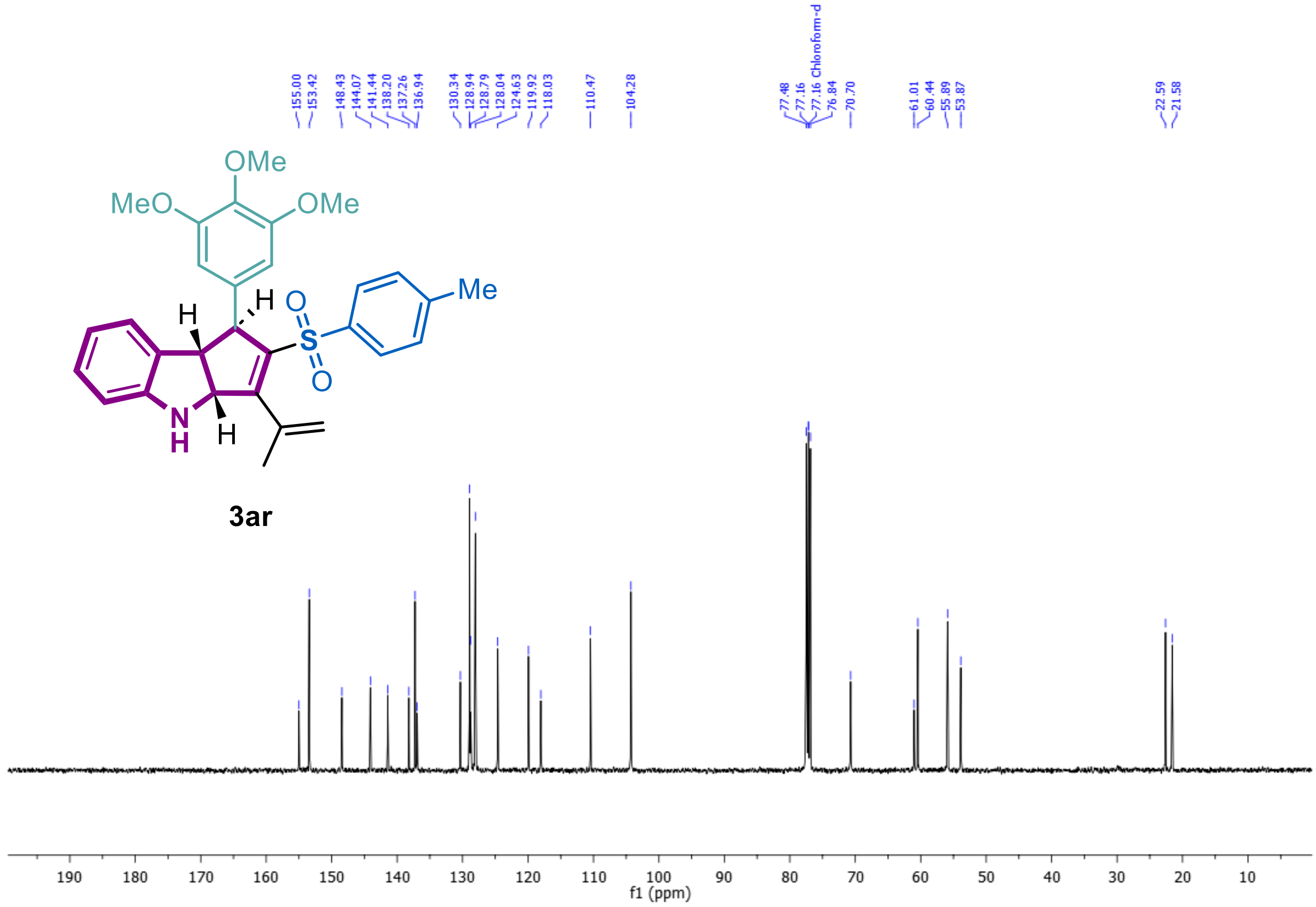

3ar

***Figure S138**: ^{13}C-NMR of **3ar** in $CDCl_3$ (101 MHz)*

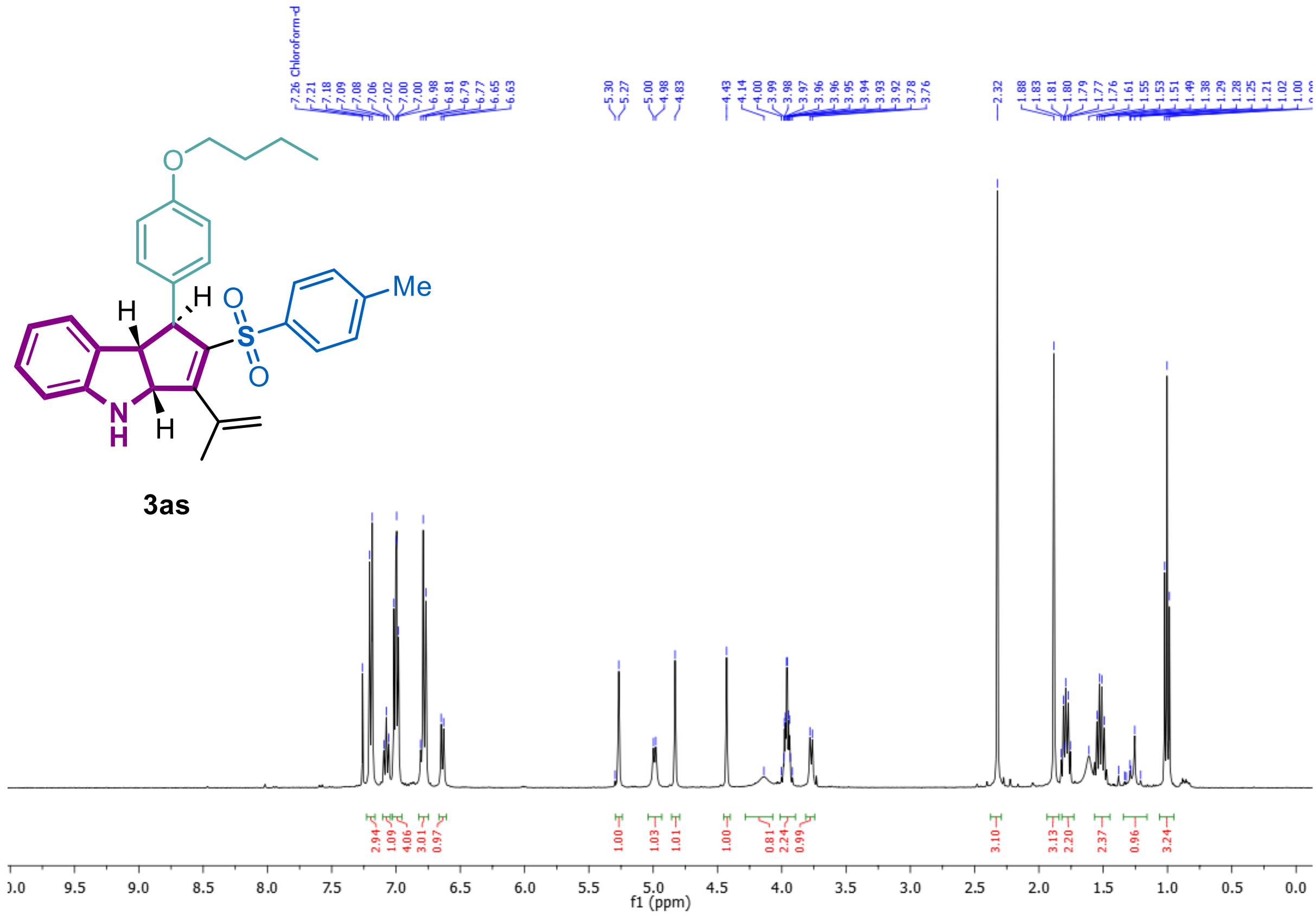

3as

***Figure S139**: ^{1}H-NMR of **3as** in $CDCl_3$ (400 MHz)*

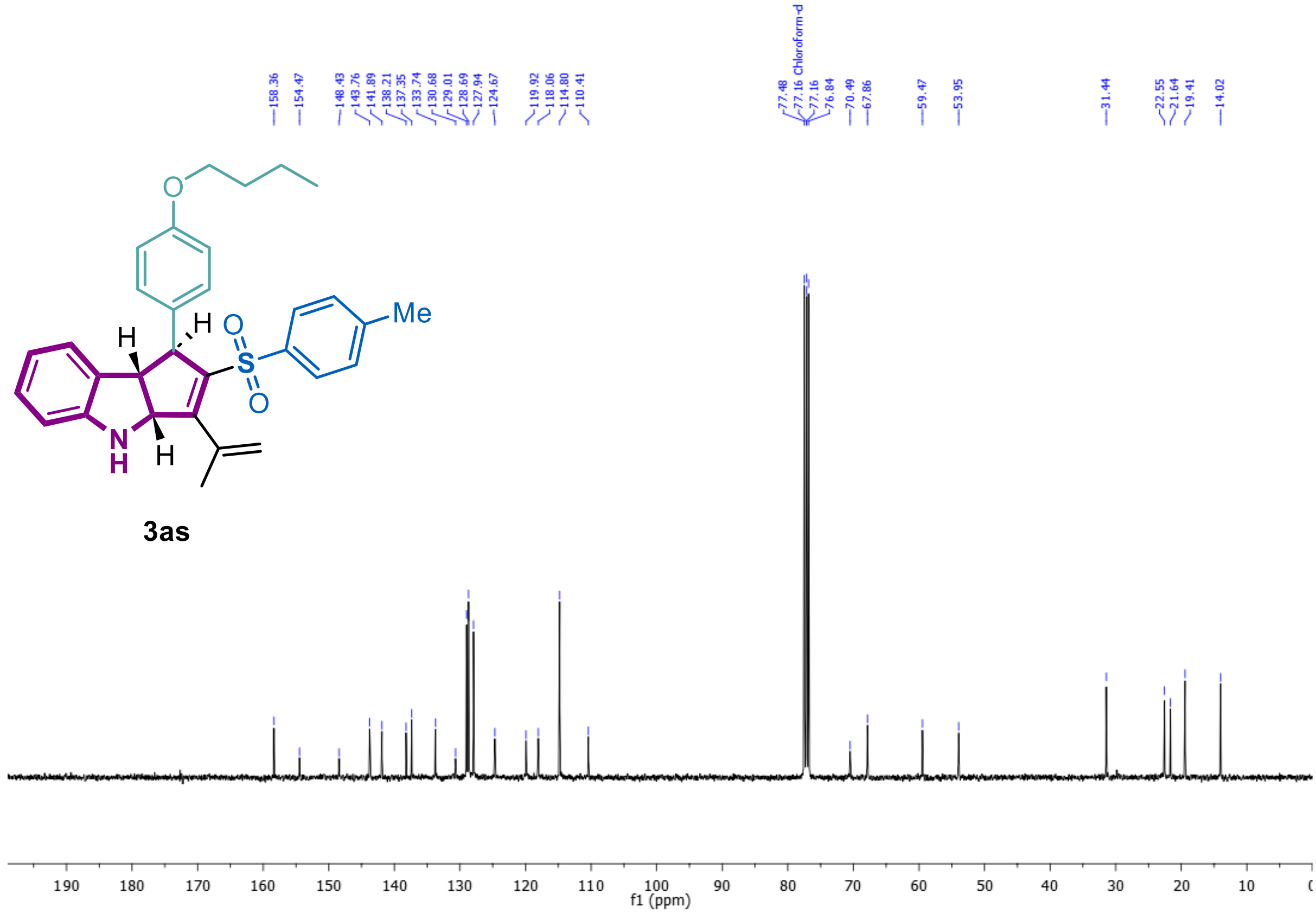

Figure S140: ^{13}C-NMR of **3as** in $CDCl_3$ (101 MHz)

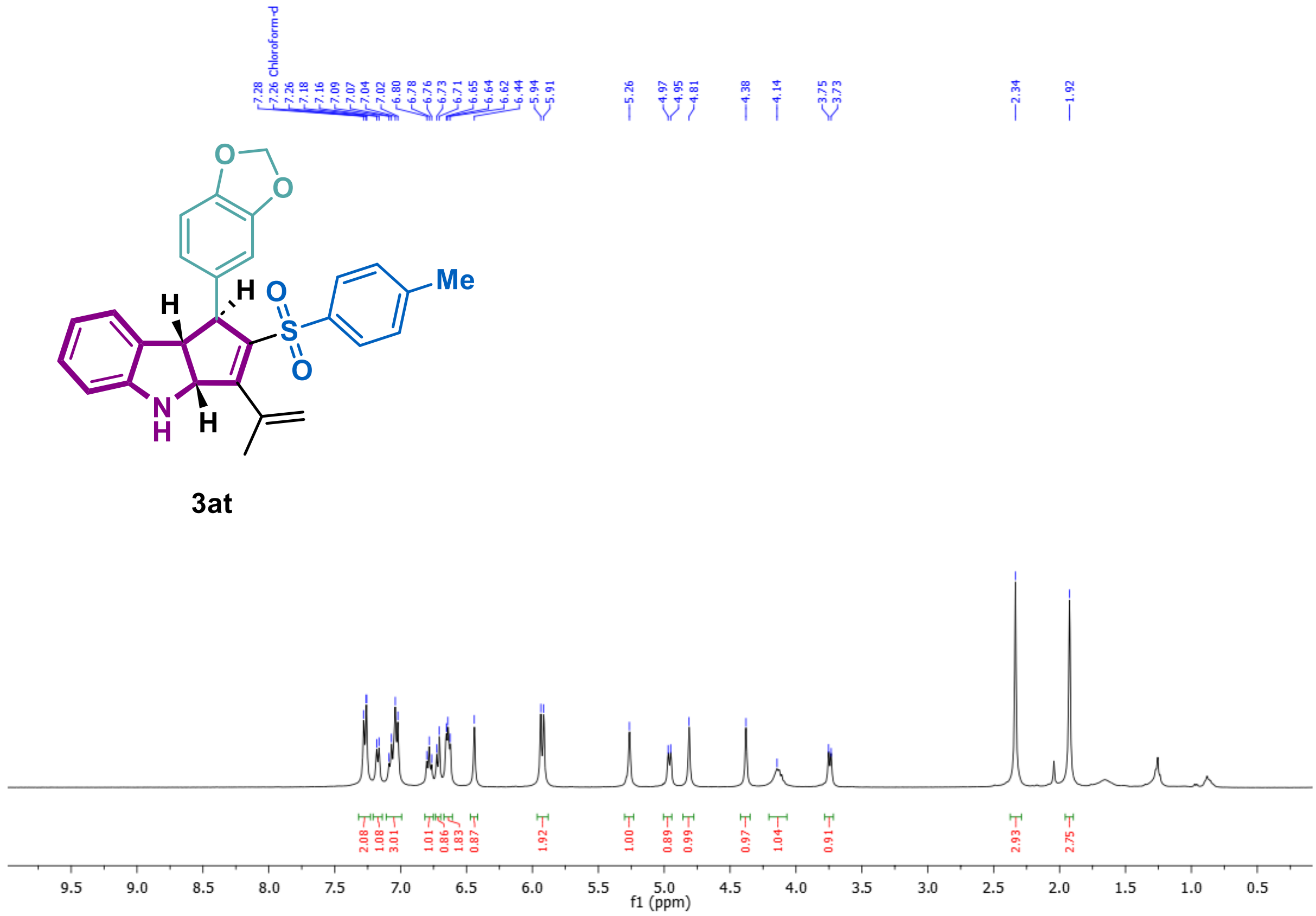

Figure S141: ^{1}H-NMR of **3at** in $CDCl_3$ (400 MHz)

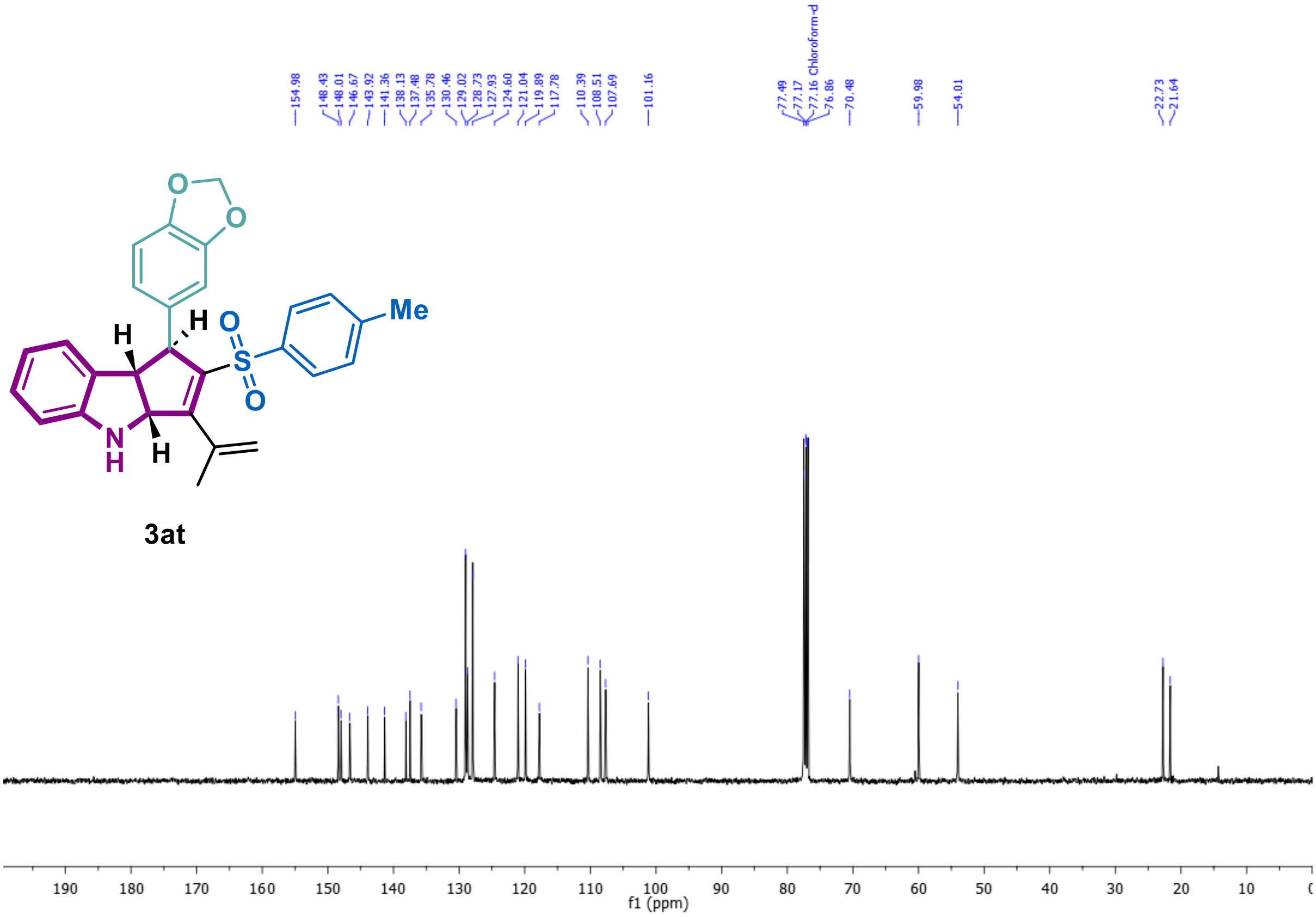

Figure S142: ^{13}C-NMR of **3at** in $CDCl_3$ (101 MHz)

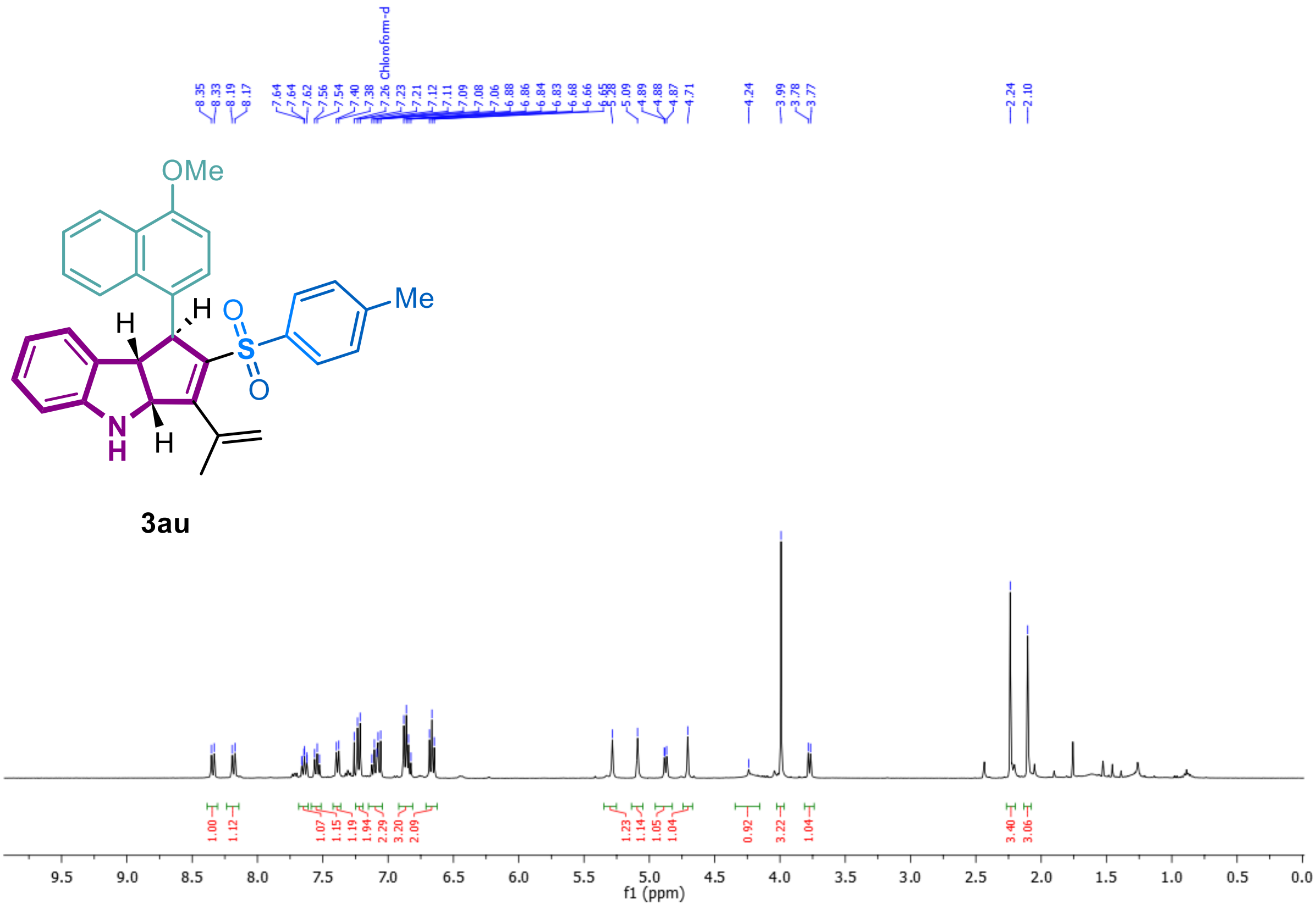

Figure S143: ^{1}H-NMR of **3au** in $CDCl_3$ (400 MHz)

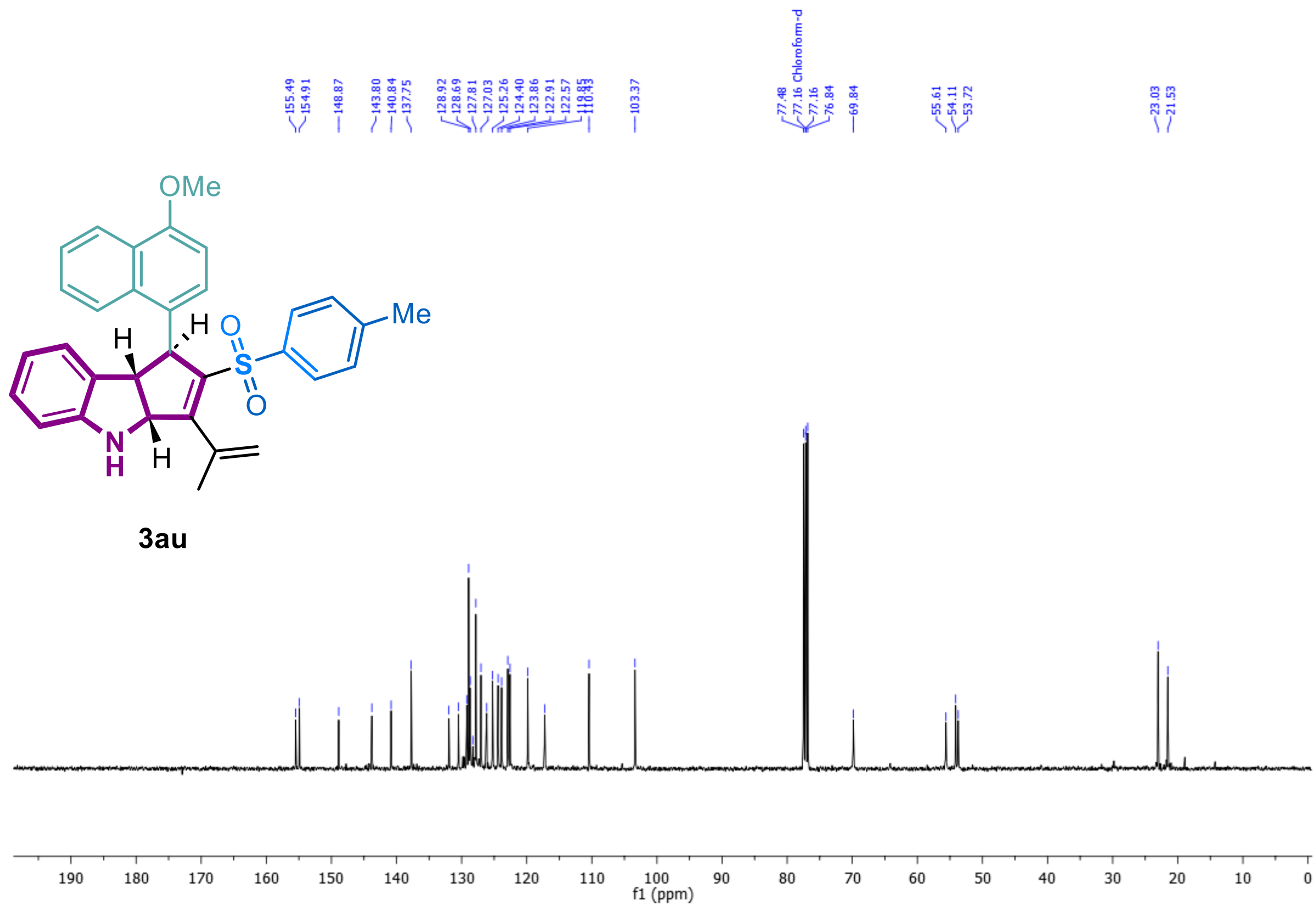

***Figure S144**: ^{13}C-NMR of **3au** in $CDCl_3$ (101 MHz)*

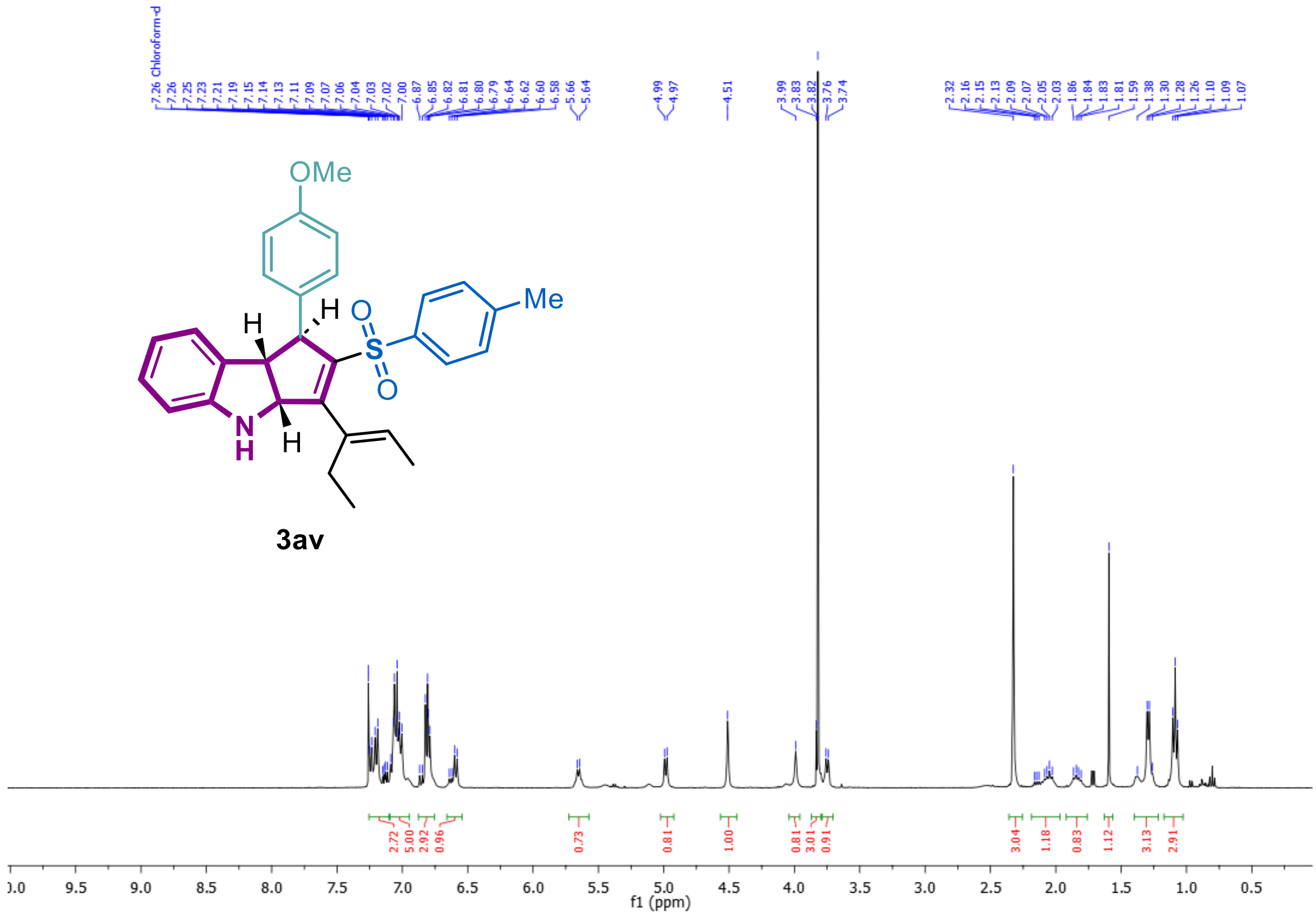

***Figure S145**: ^{1}H-NMR of **3av** in $CDCl_3$ (400 MHz)*

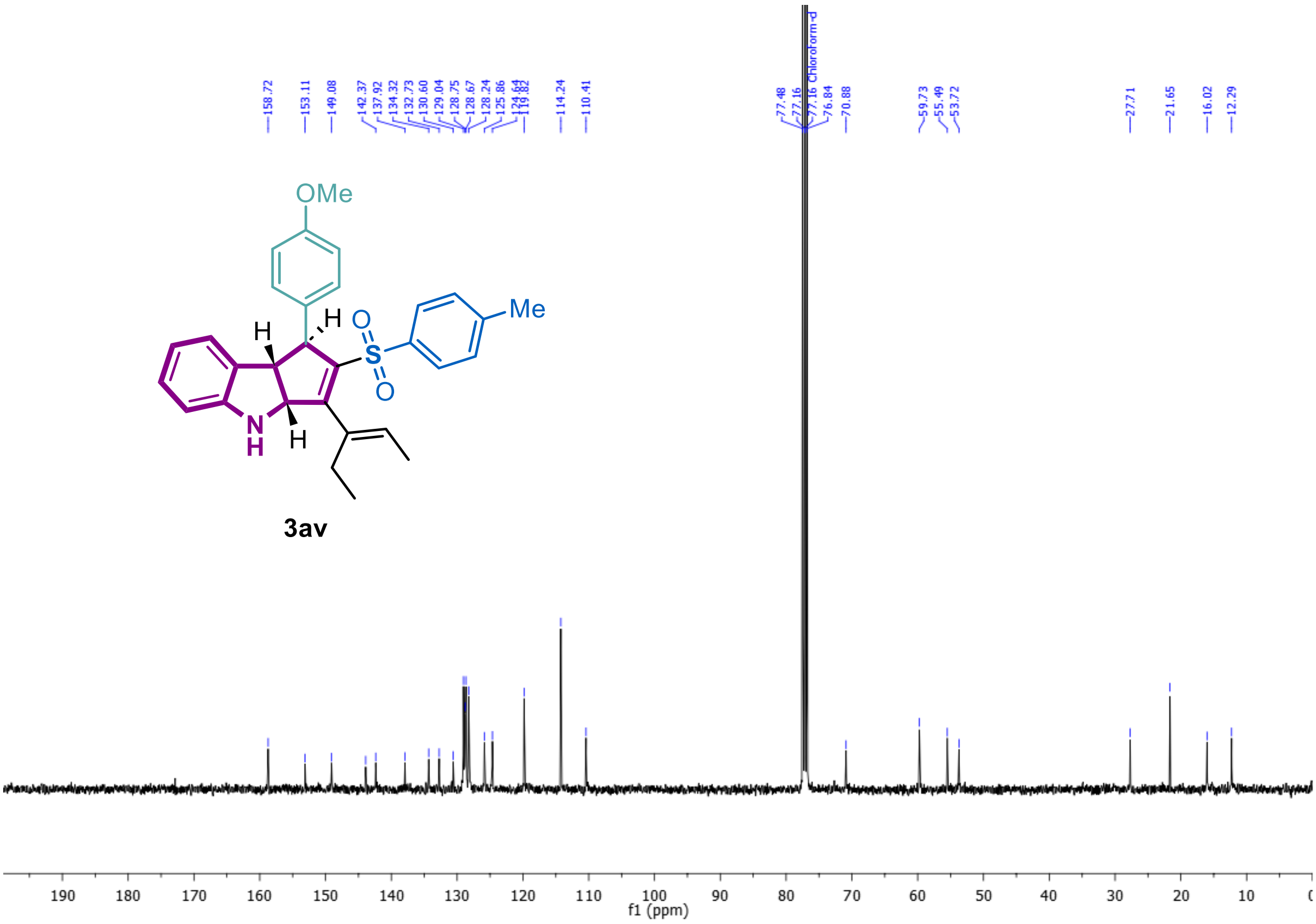

Figure S146: *^{13}C-NMR of **3av** in $CDCl_3$ (101 MHz)*

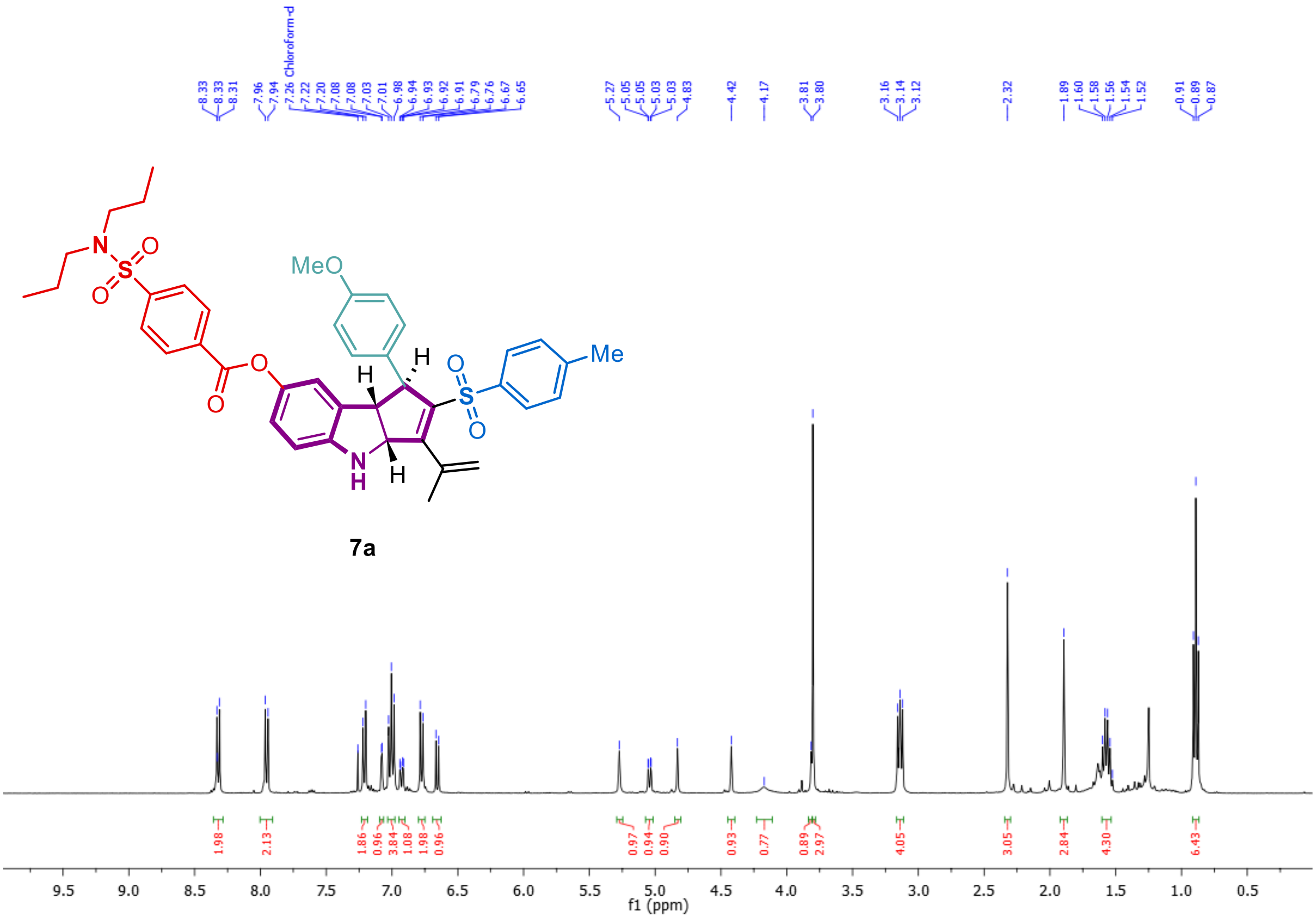

Figure S147: *^{1}H-NMR of **7a** in $CDCl_3$ (400 MHz)*

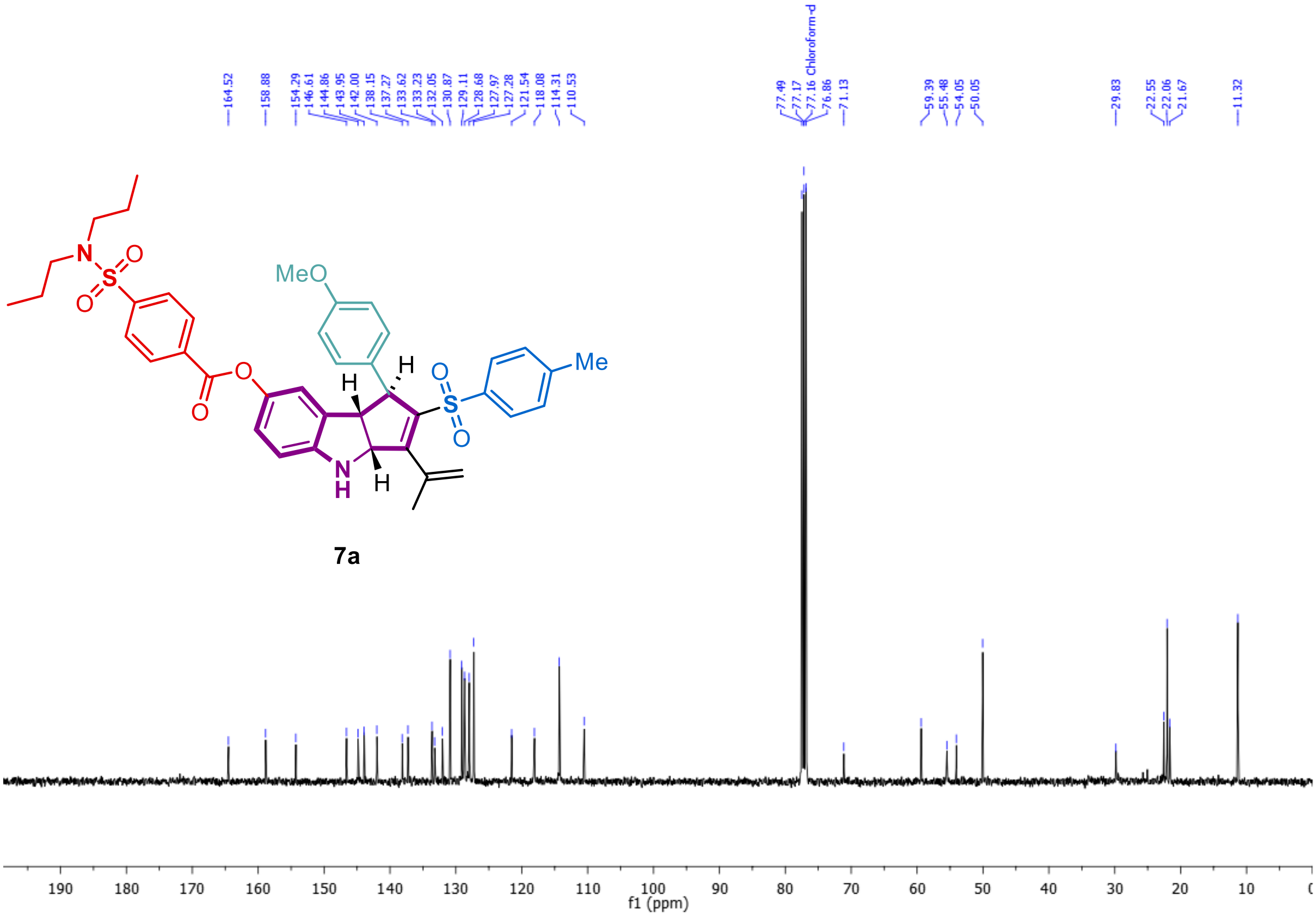

Figure S148: ^{13}C-NMR of **7a** in $CDCl_3$ (101 MHz)

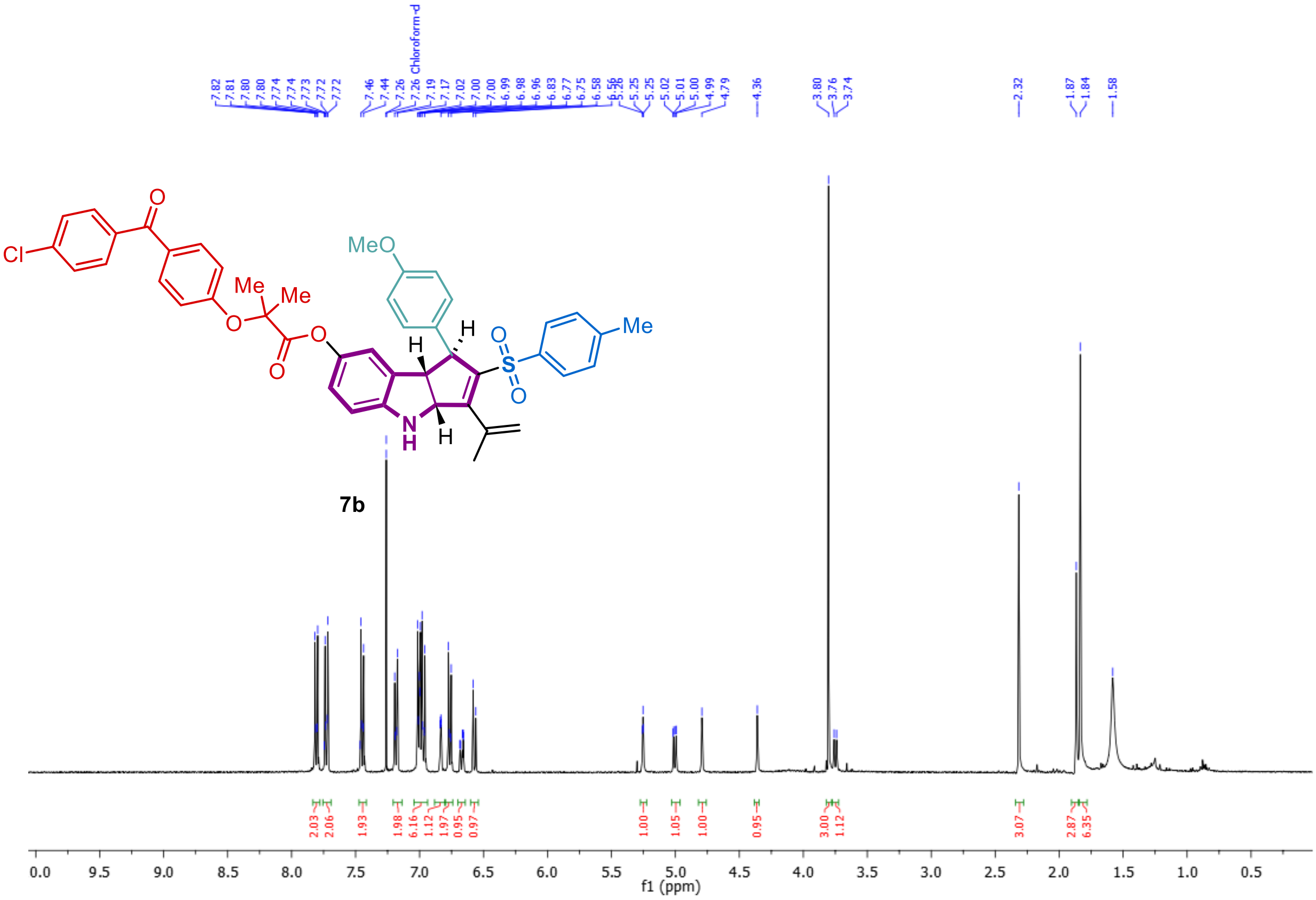

Figure S149: ^{1}H-NMR of **7b** in $CDCl_3$ (400 MHz)

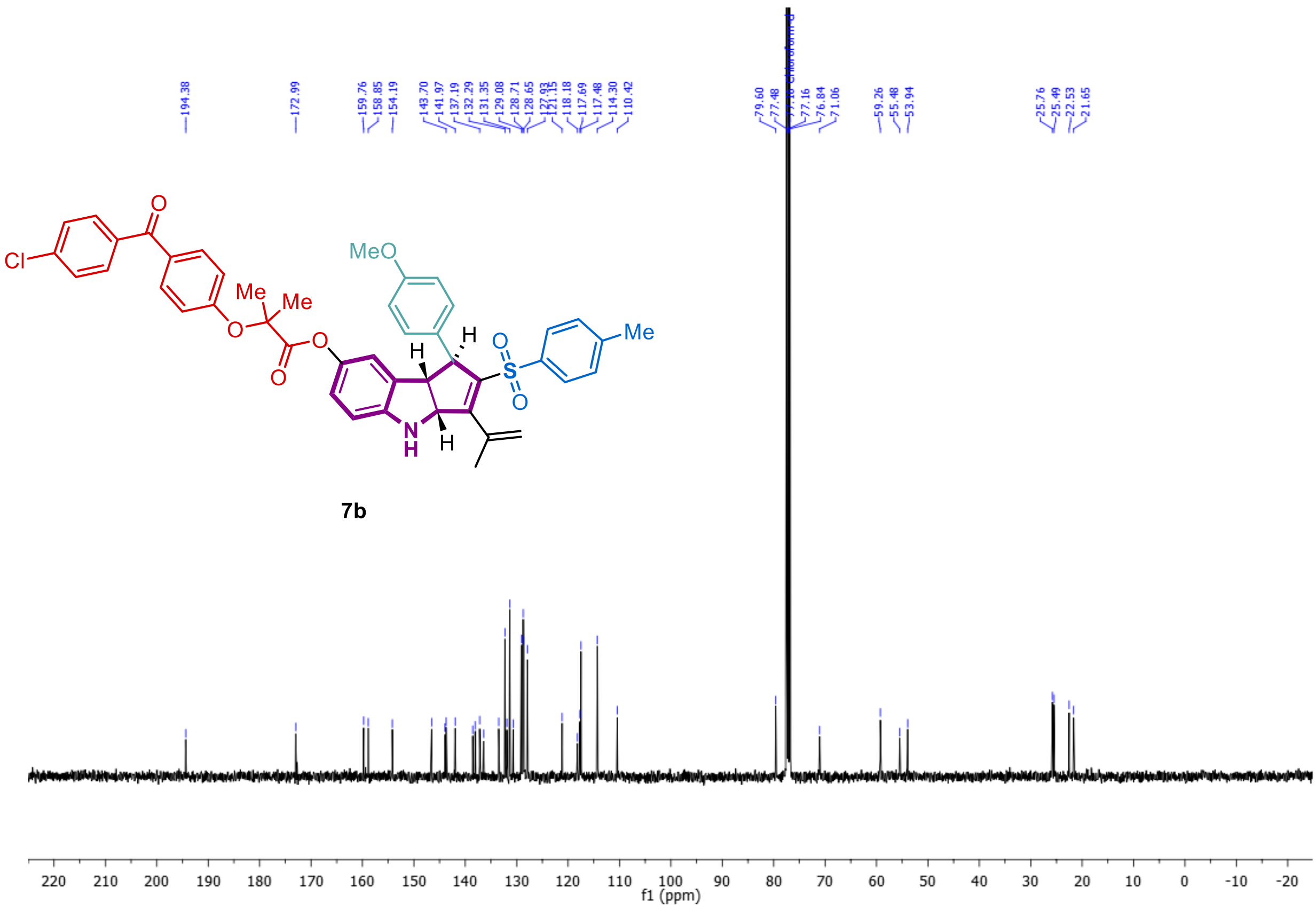

Figure S150: *^{13}C-NMR of **7b** in $CDCl_3$ (101 MHz)*

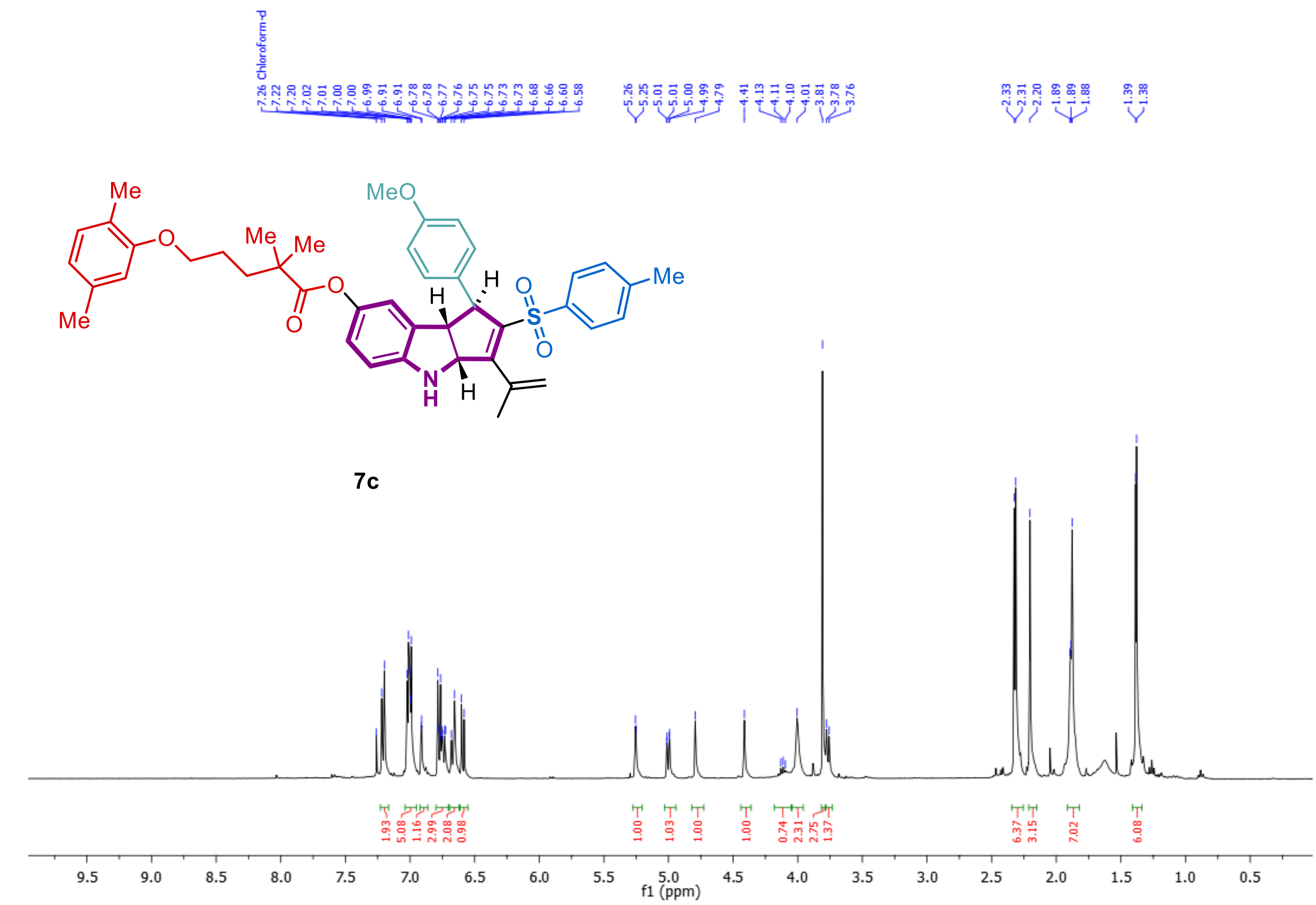

Figure S151: *^{1}H-NMR of **7c** in $CDCl_3$ (400 MHz)*

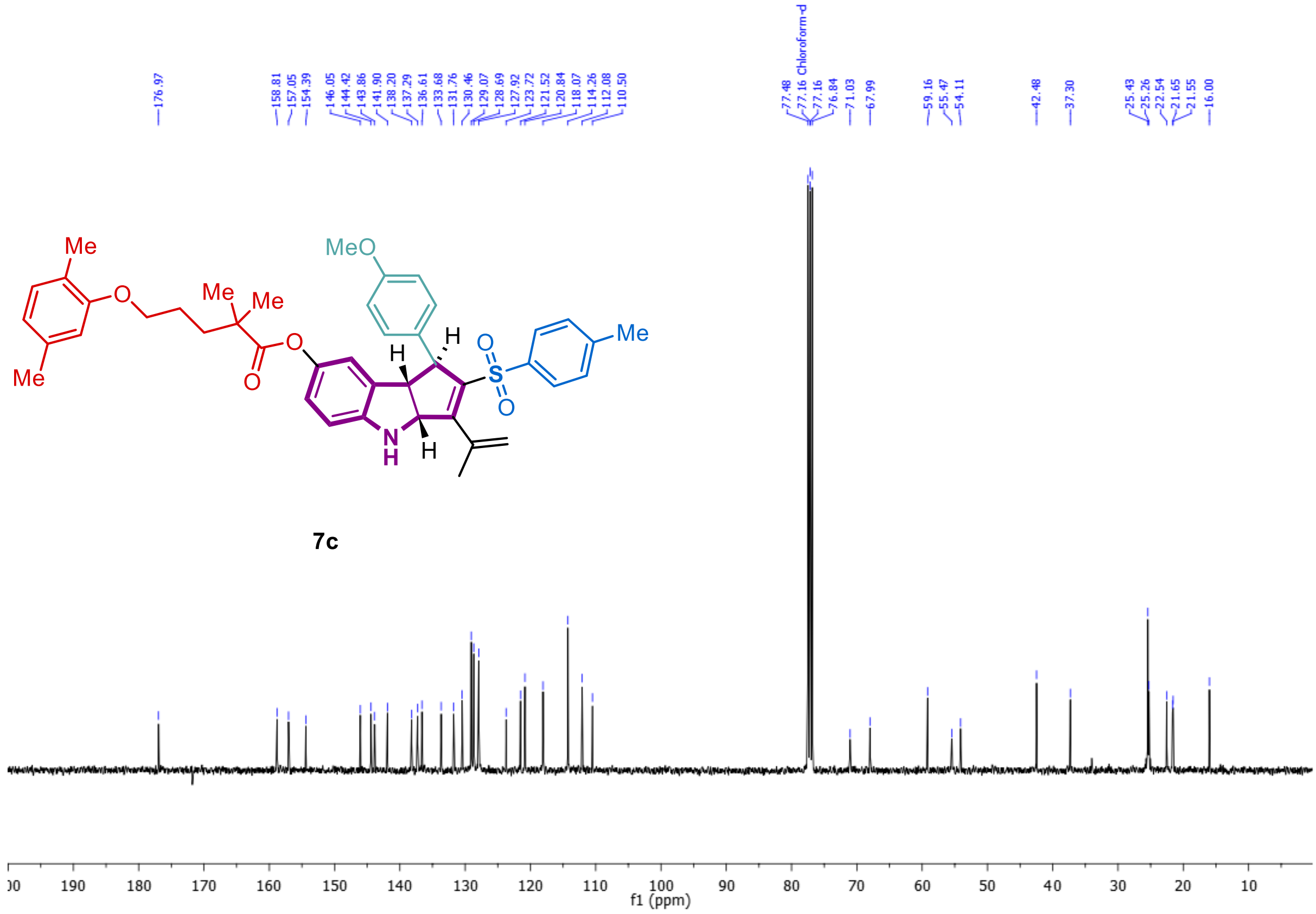

Figure S152: ^{13}C-NMR of **7c** in $CDCl_3$ (101 MHz)

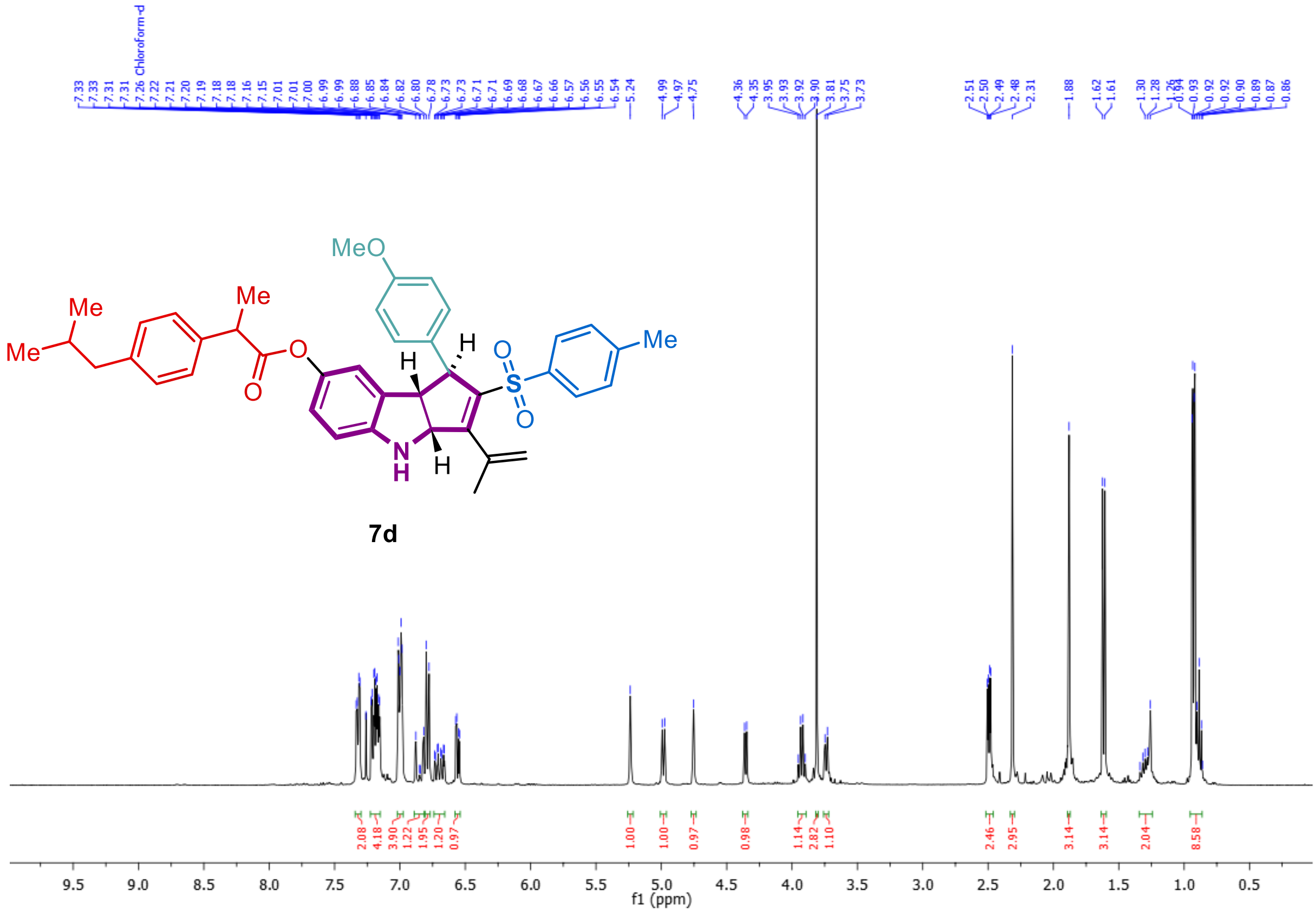

Figure S153: ^{1}H-NMR of **7d** in $CDCl_3$ (400 MHz)

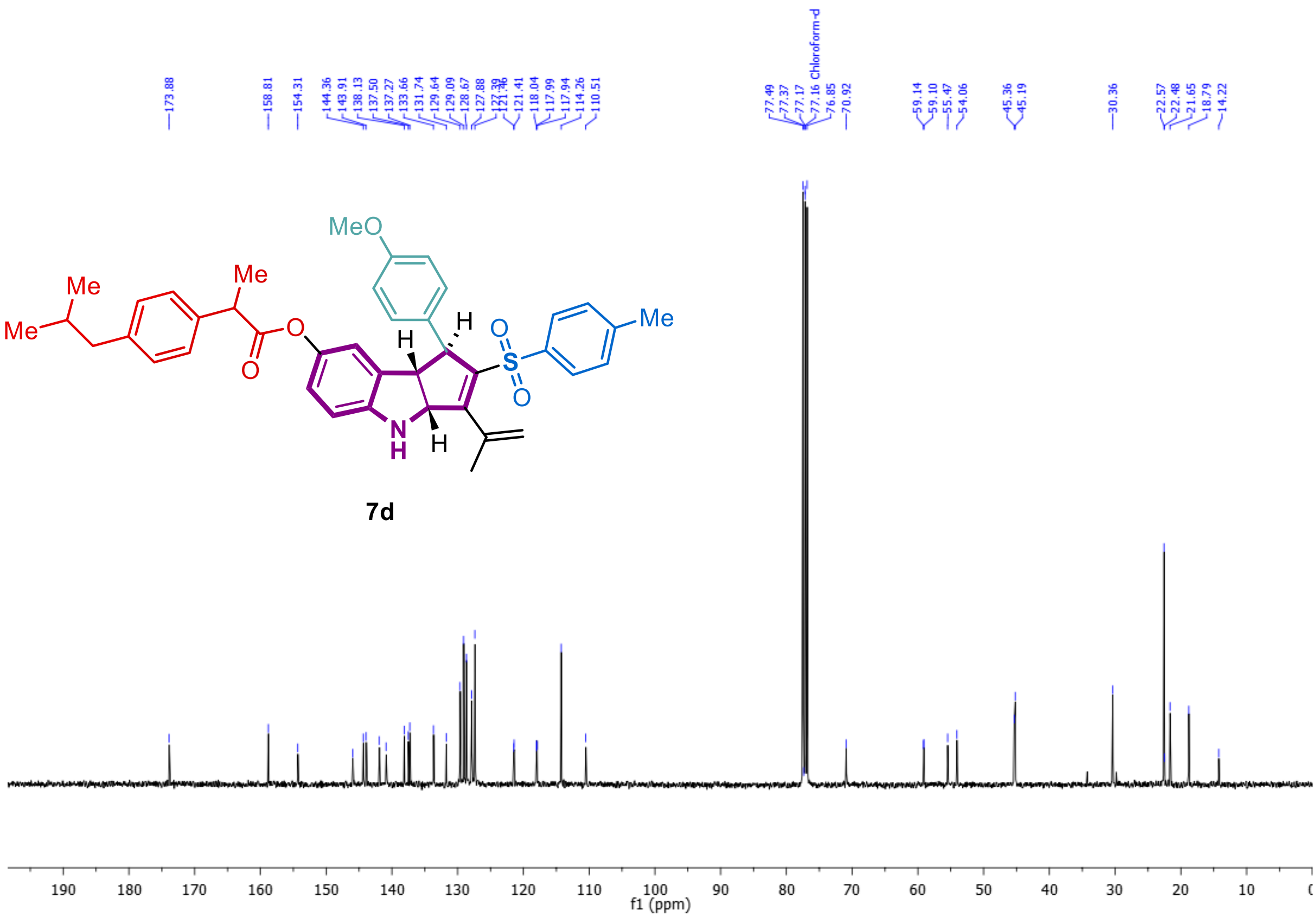

Figure S154: ^{13}C-NMR of ***7d*** in $CDCl_3$ (101 MHz)

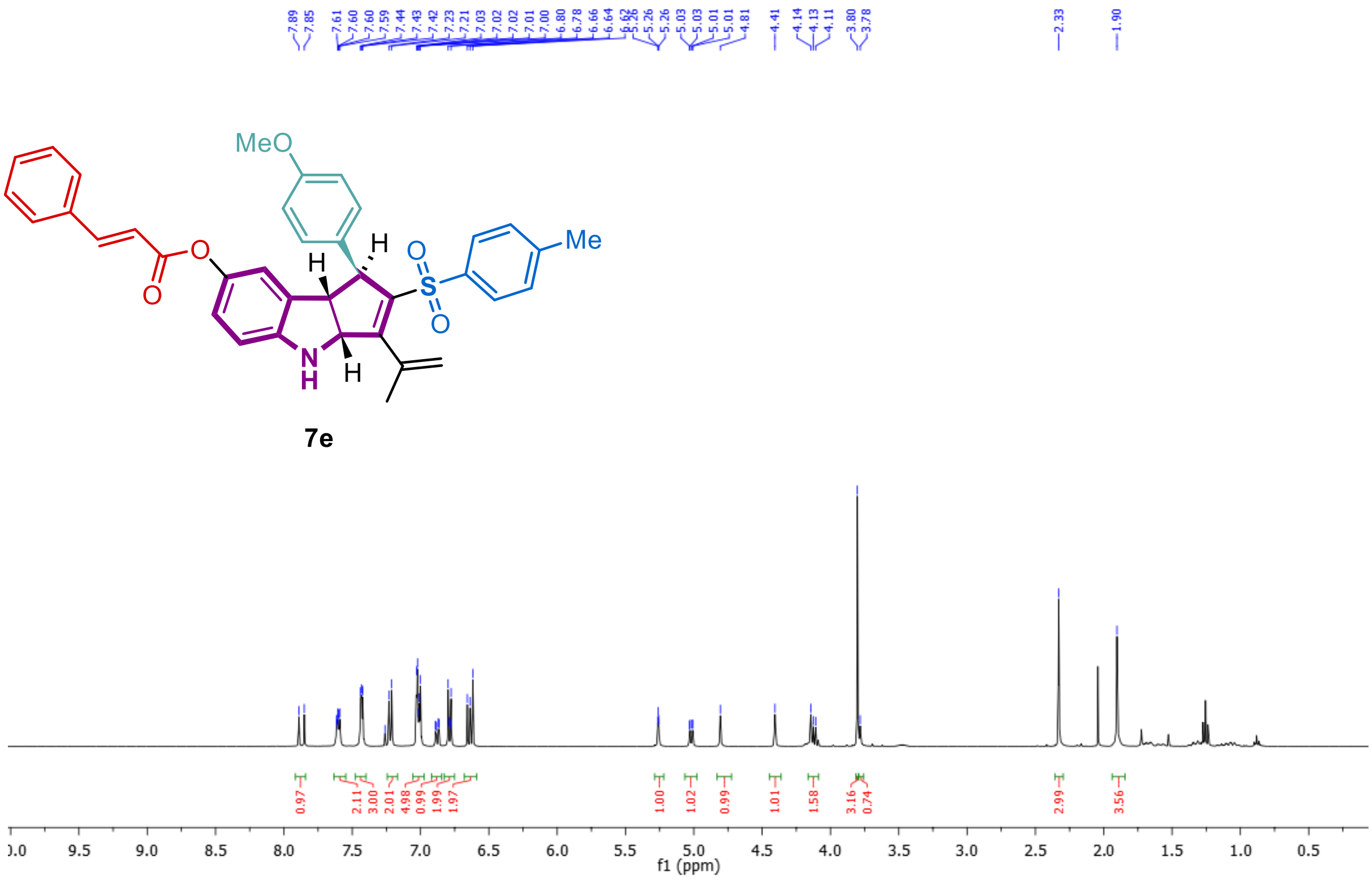

Figure S155: ^{1}H-NMR of ***7e*** in $CDCl_3$ (400 MHz)

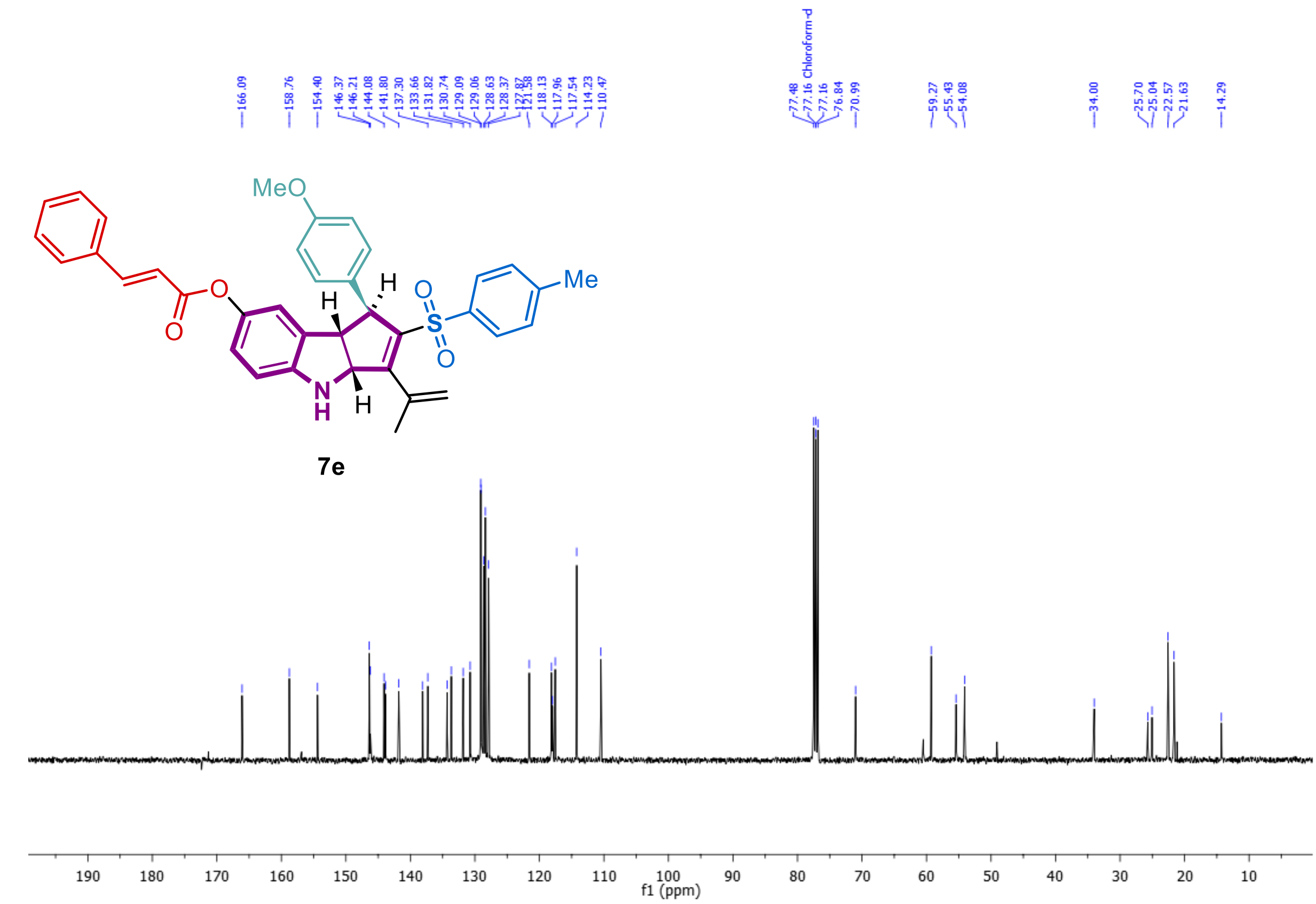

Figure S156: *^{13}C-NMR of **7e** in $CDCl_3$ (101 MHz)*

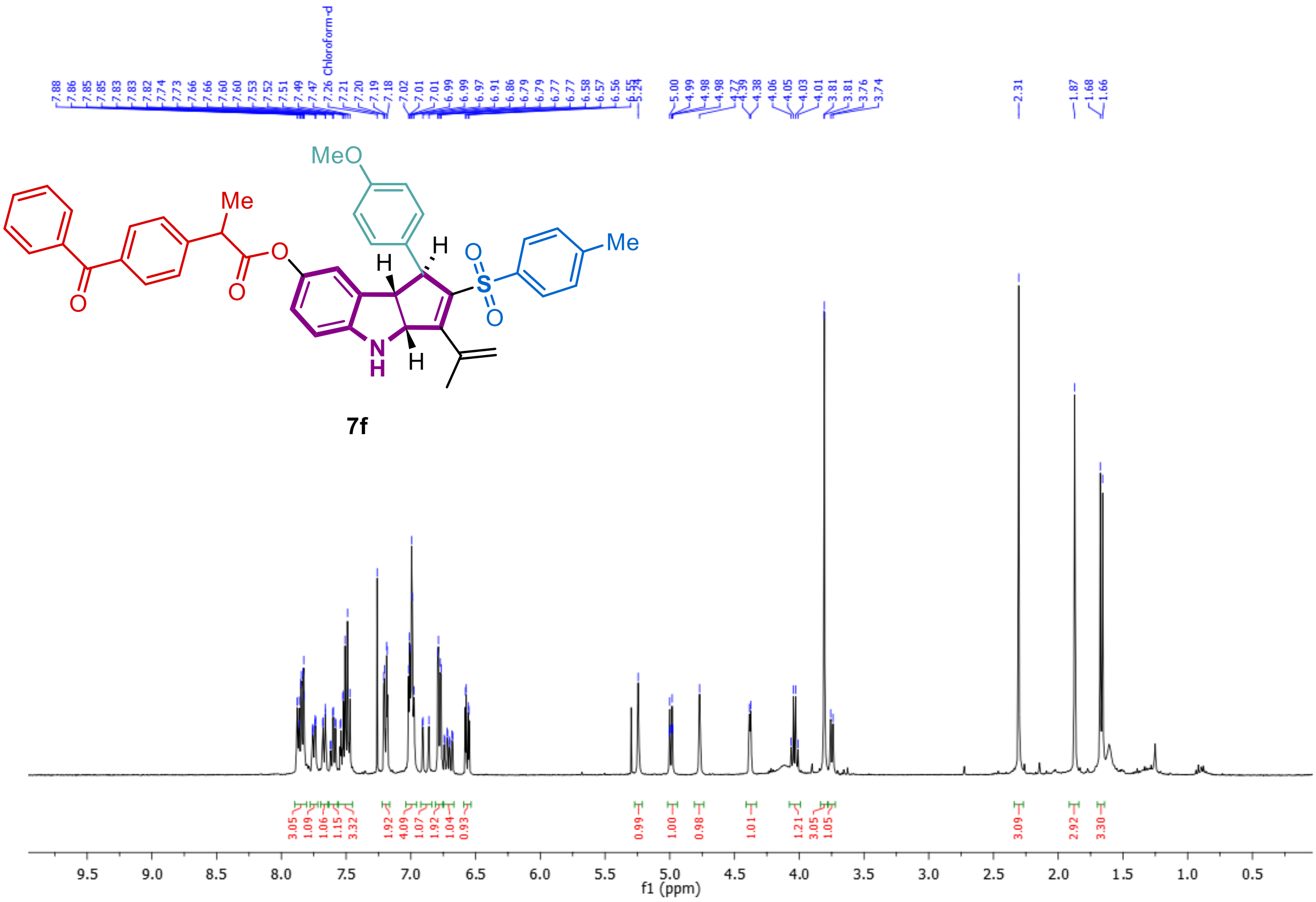

Figure S157: *^{1}H-NMR of **7f** in $CDCl_3$ (400 MHz)*

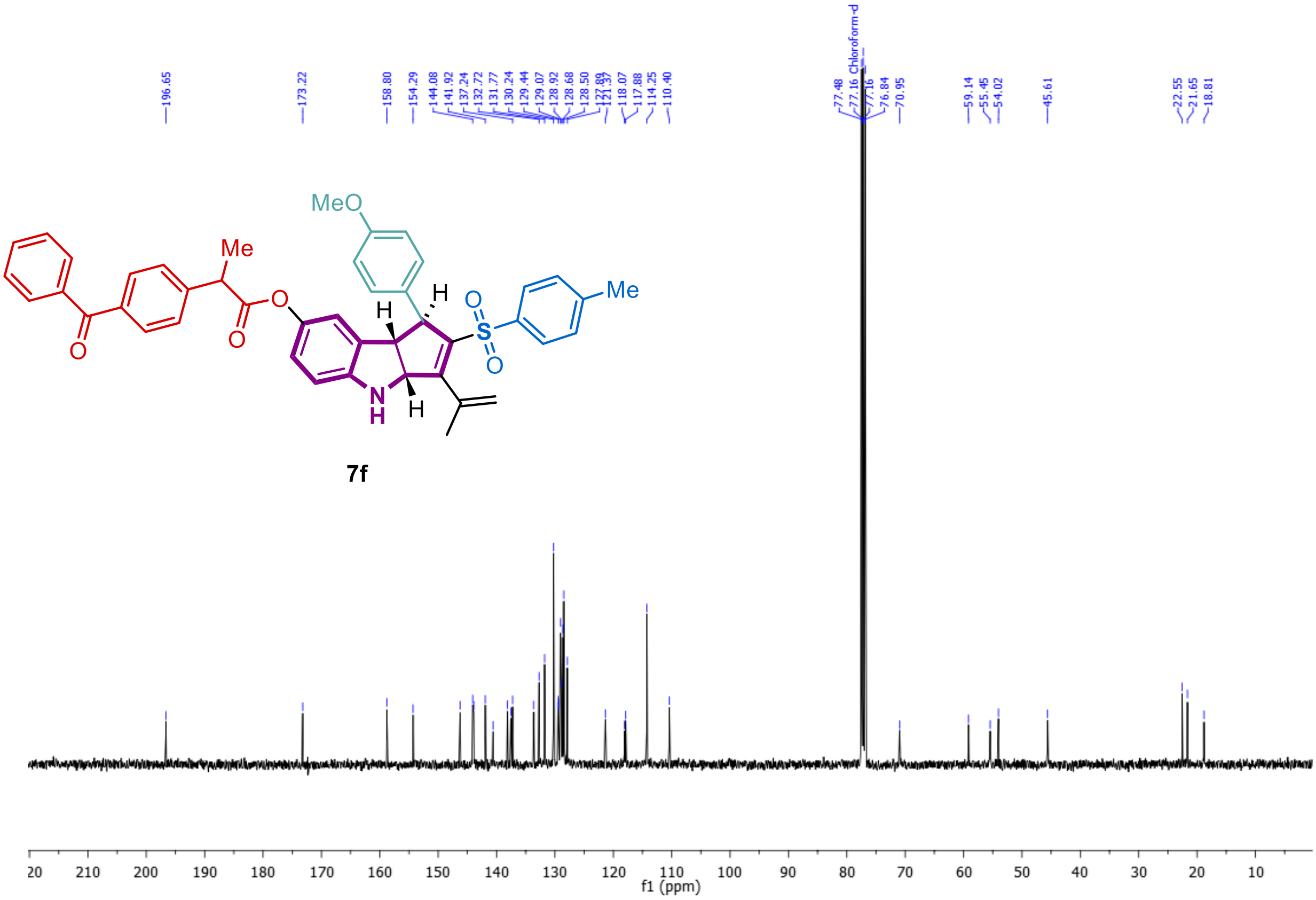

Figure S158: *^{13}C-NMR of **7f** in $CDCl_3$ (101 MHz)*

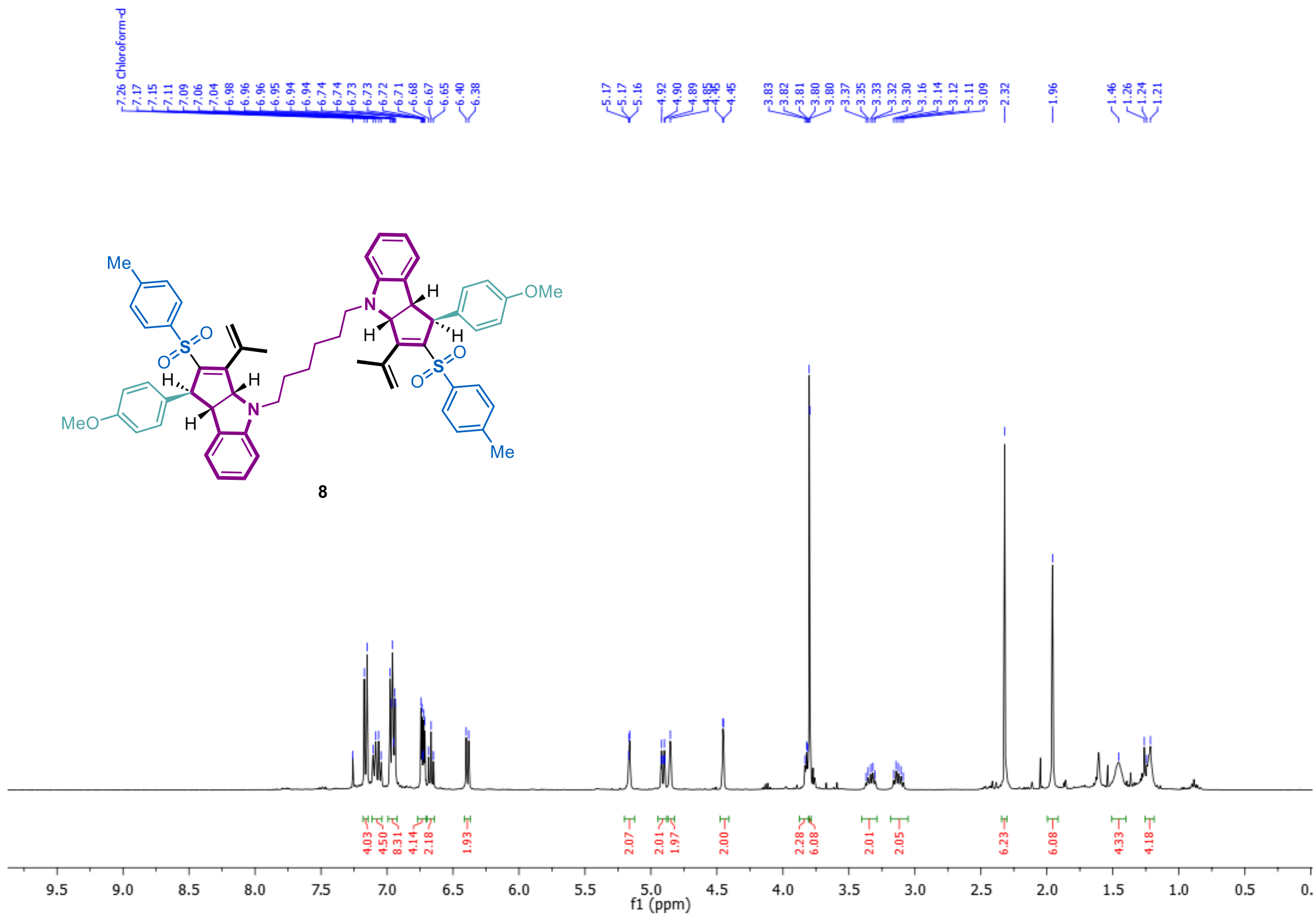

Figure S159: *^{1}H-NMR of **8** in $CDCl_3$ (400 MHz)*

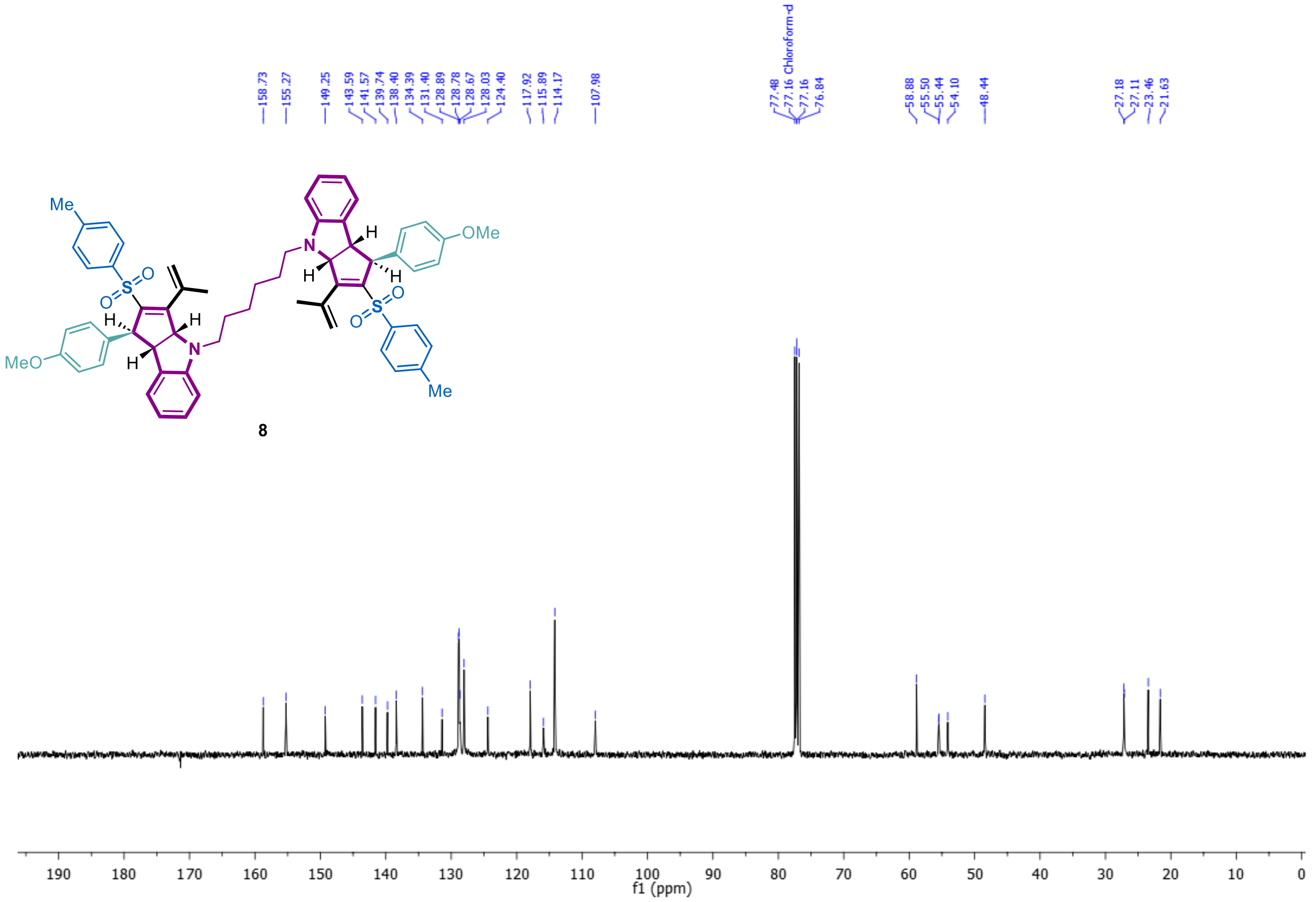

Figure S160: ^{13}C-NMR of **8** in $CDCl_3$ (101 MHz)

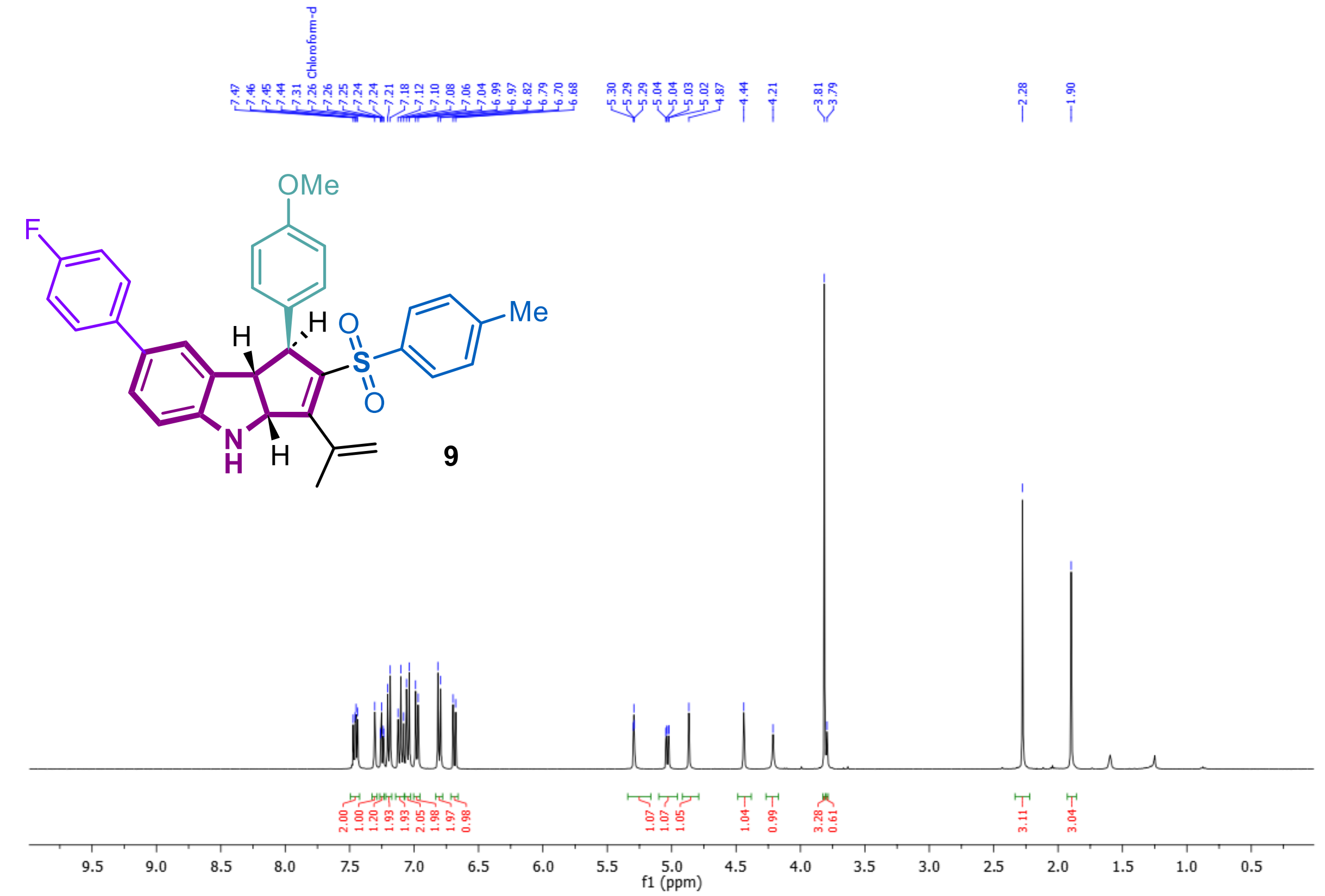

Figure S161: ^{1}H-NMR of **9** in $CDCl_3$ (400 MHz)

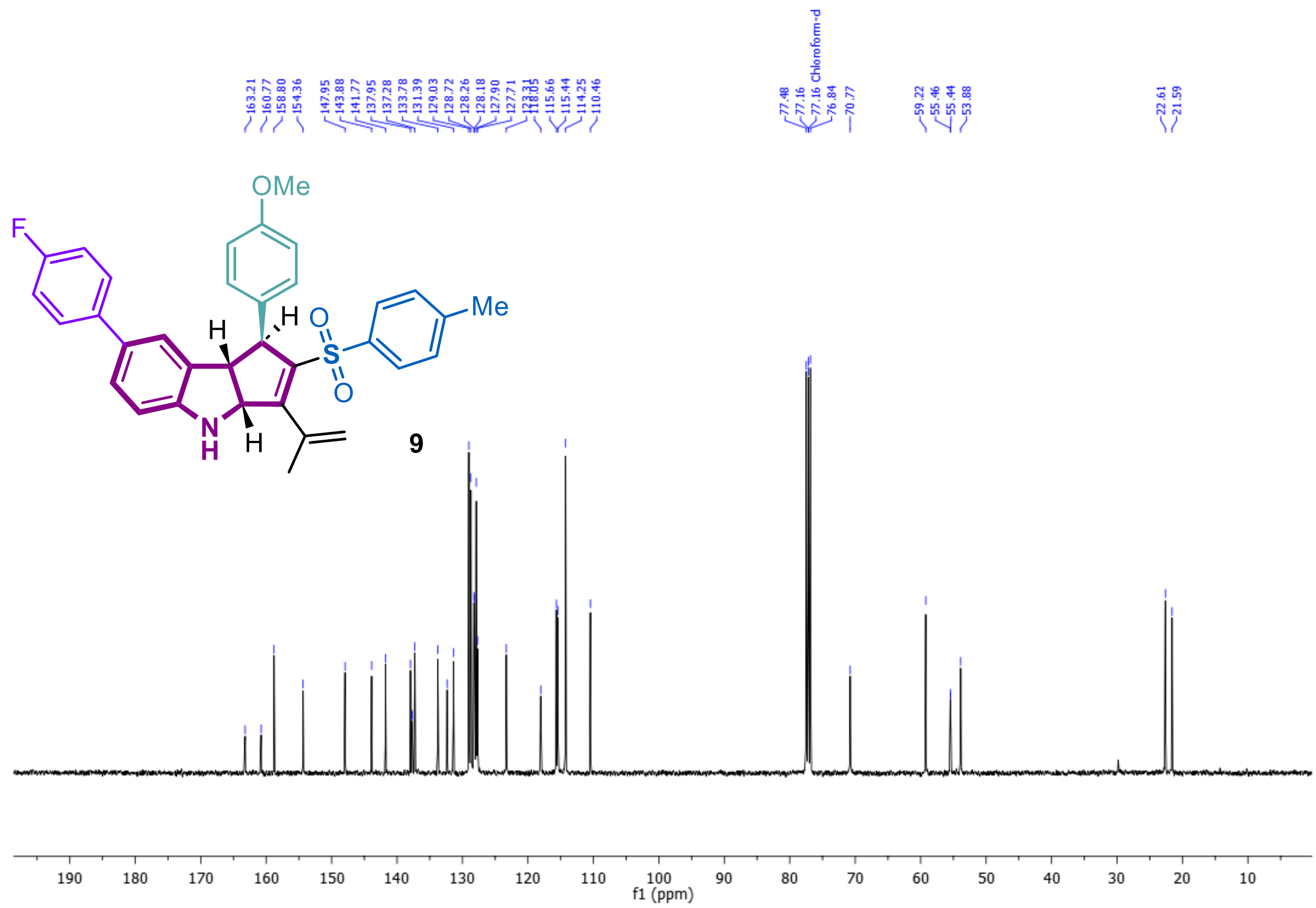

__Figure S162__: ^{13}C-NMR of __9__ in $CDCl_3$ (101 MHz)

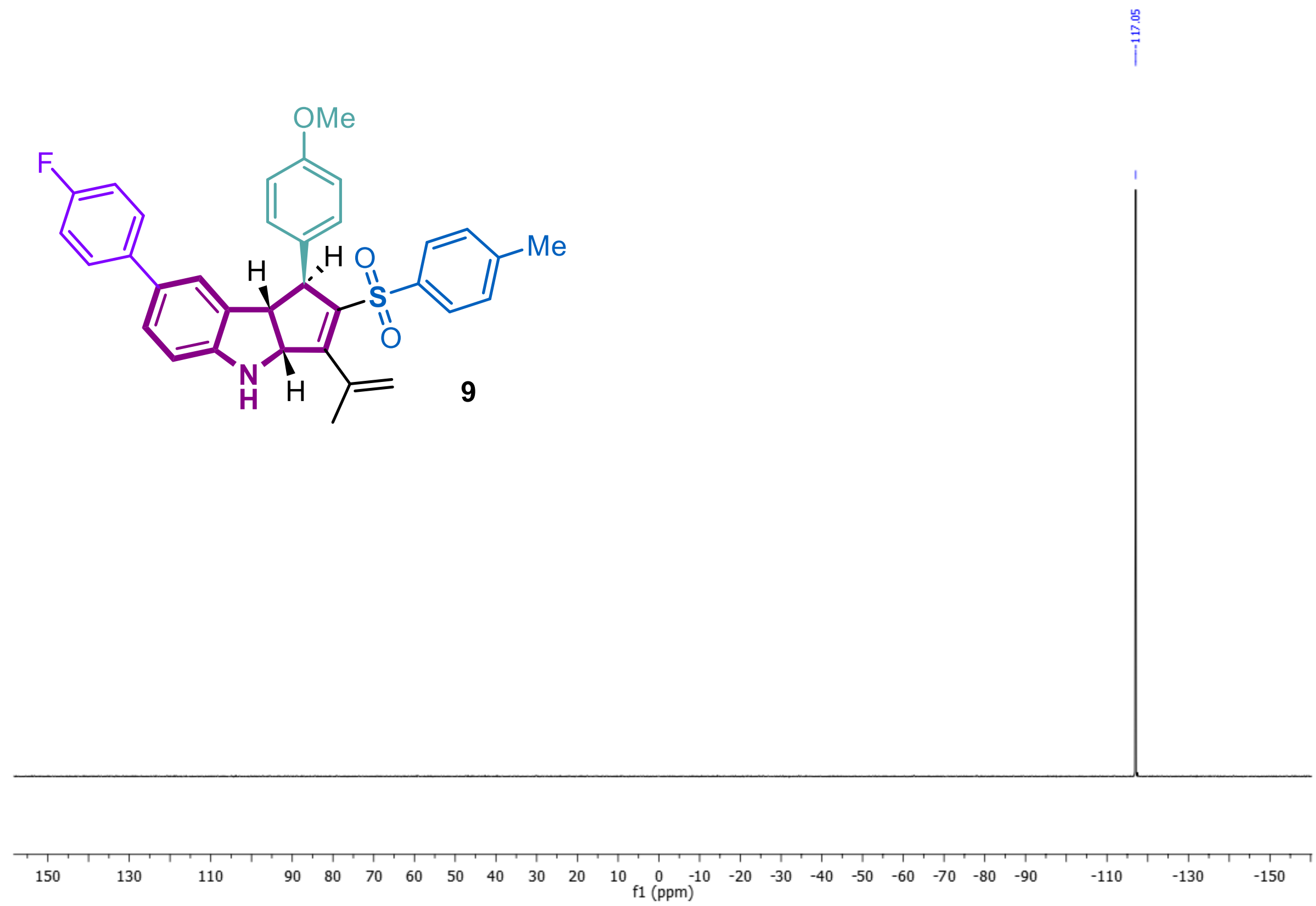

__Figure S163:__ ^{19}F-NMR of __9__ in $CDCl_3$ (376 MHz)

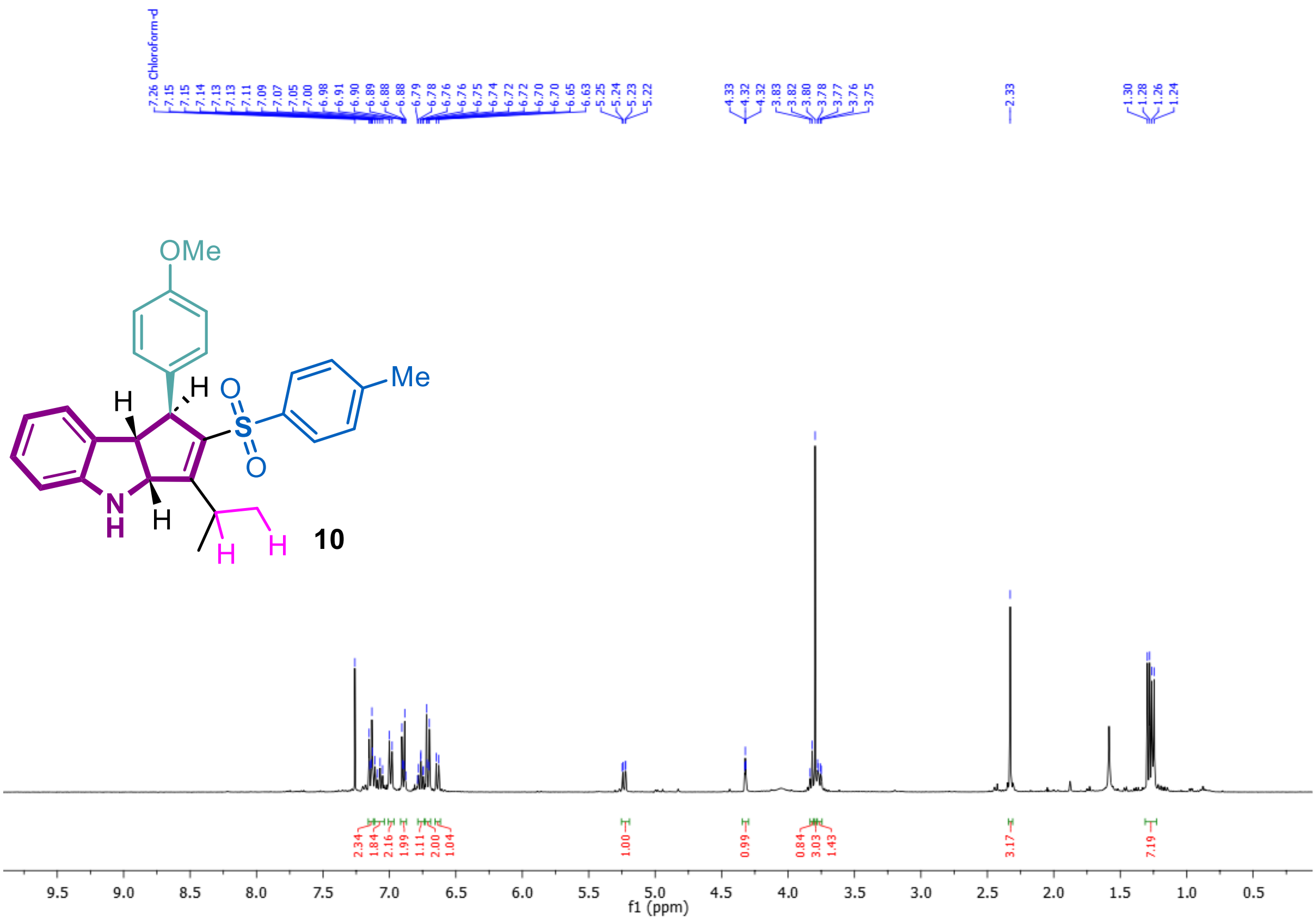

Figure S164: *^{1}H-NMR of **10** in $CDCl_3$ (400 MHz)*

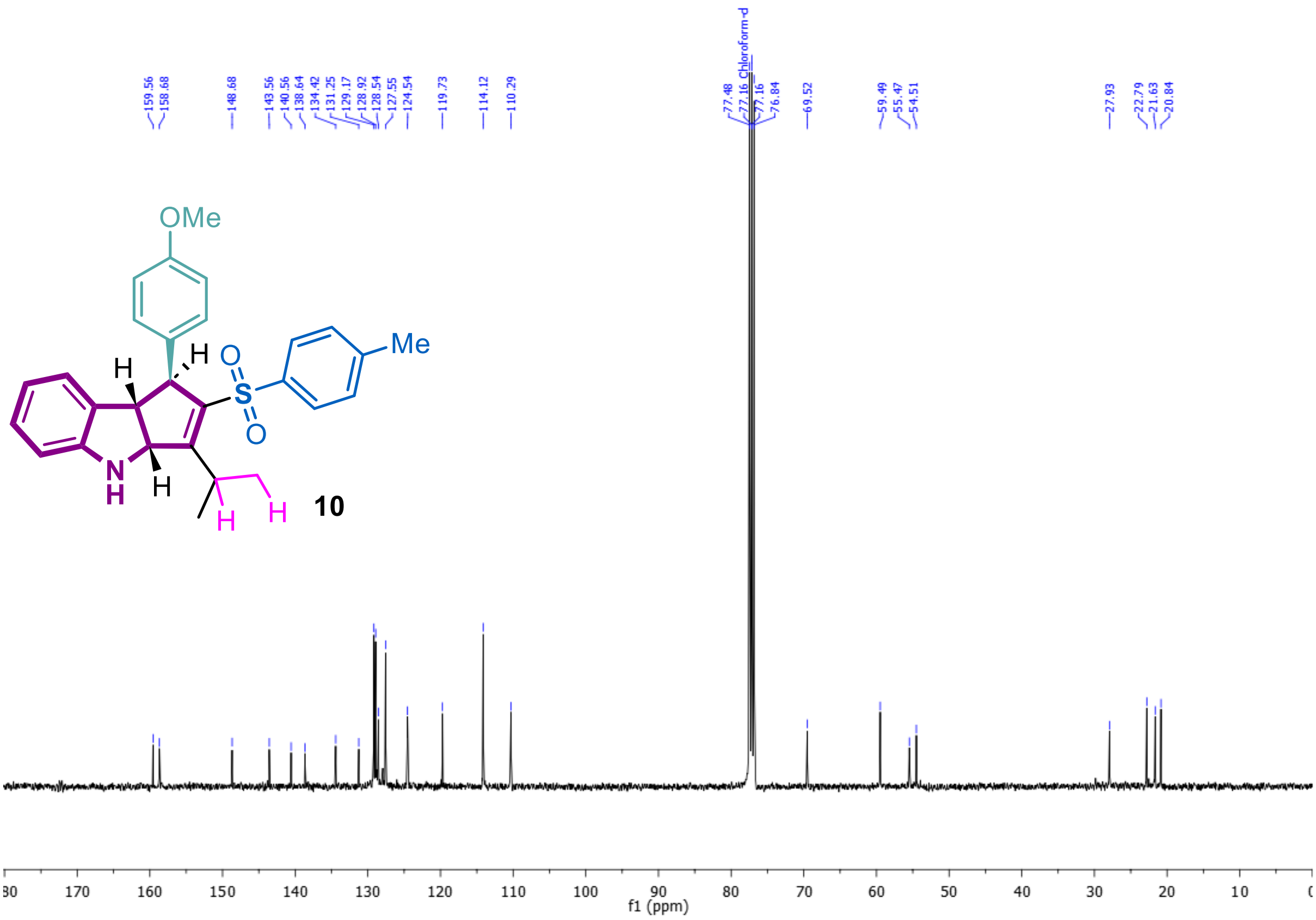

Figure S165: *^{13}C-NMR of **10** in $CDCl_3$ (101 MHz)*

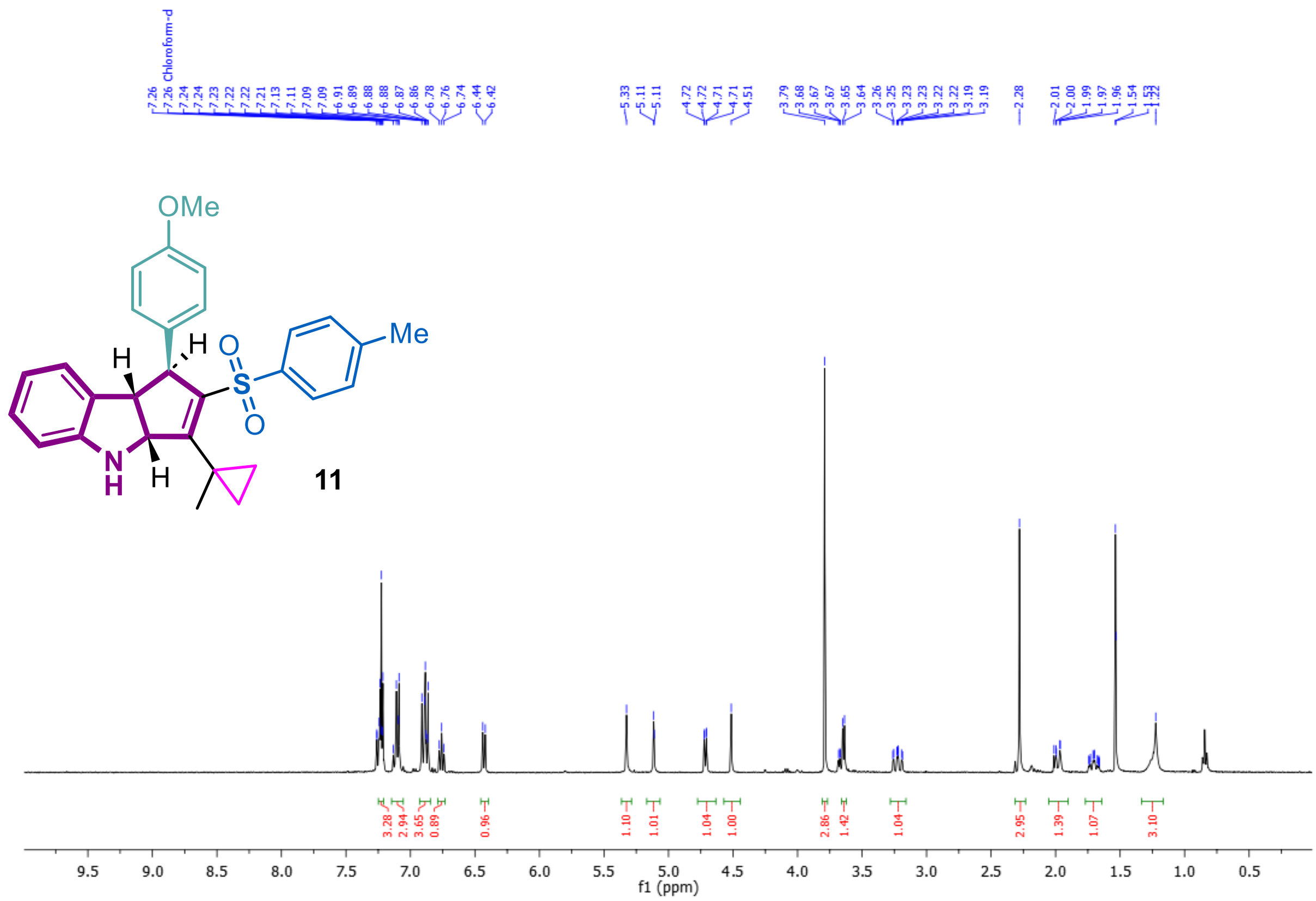

***Figure S166**: ^{1}H-NMR of **11** in $CDCl_3$ (400 MHz)*

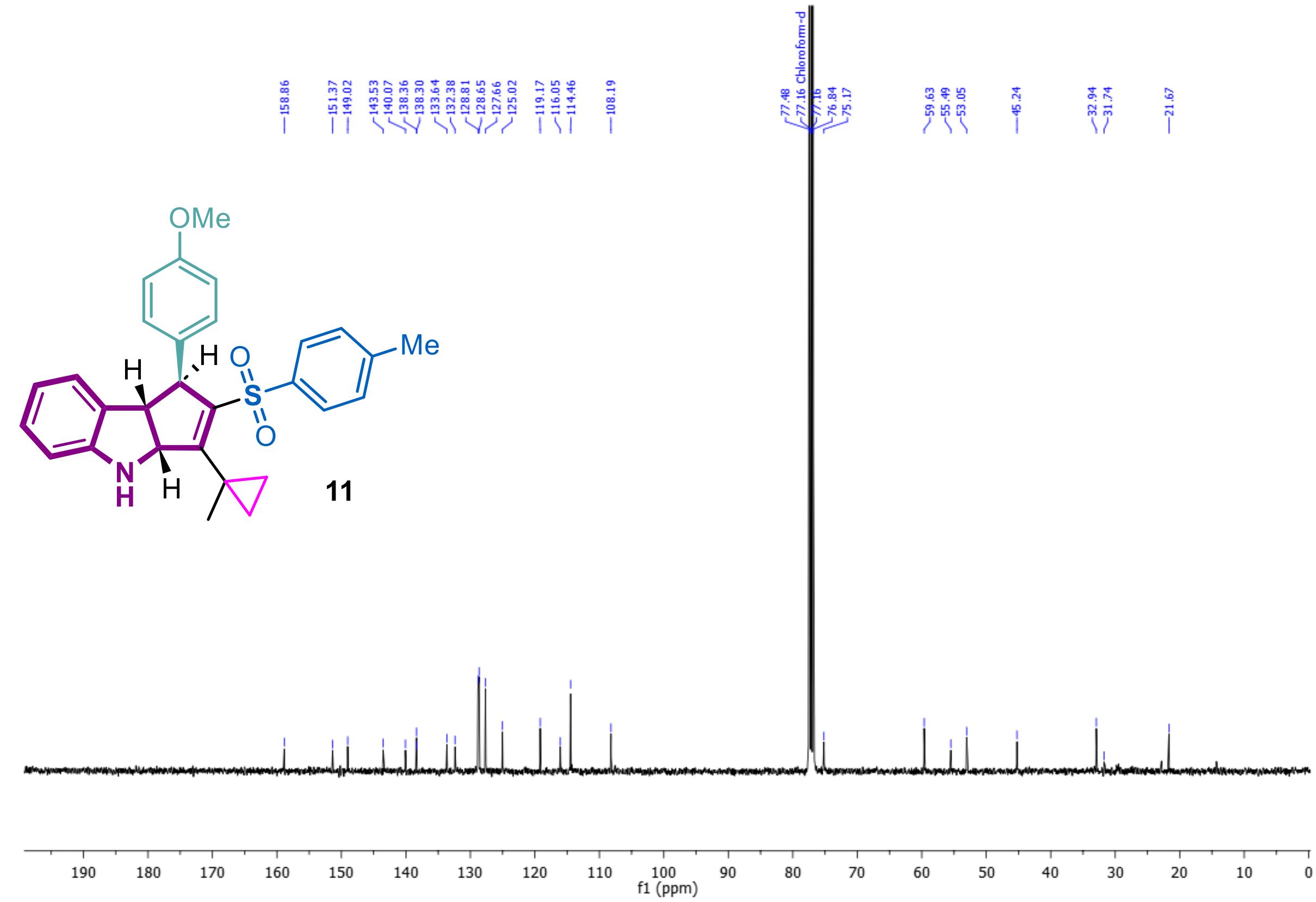

***Figure S167**: ^{13}C-NMR of **11** in $CDCl_3$ (101 MHz)*

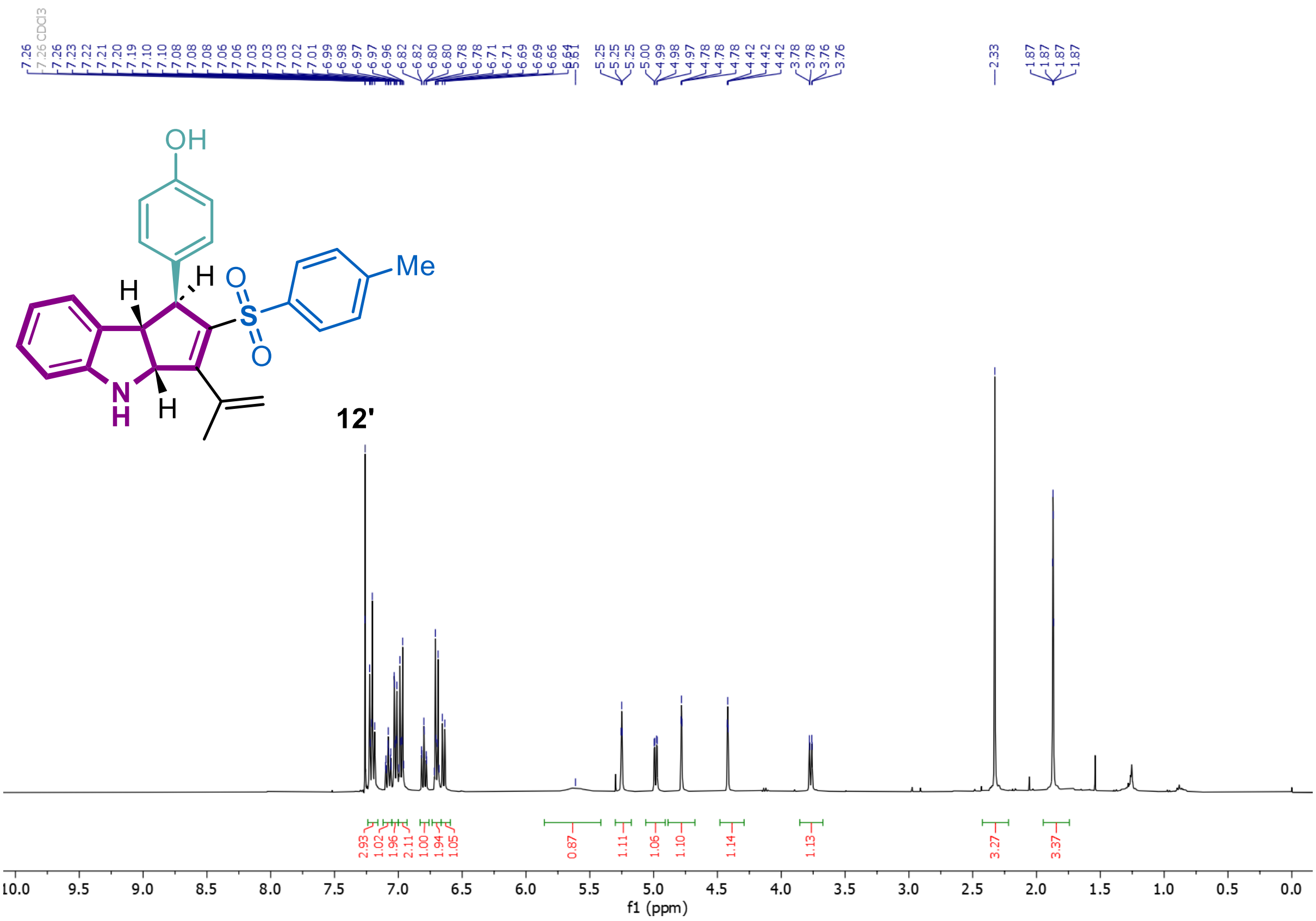

Figure S 168: *^{1}H-NMR of **12'** in $CDCl_3$ (400 MHz)*

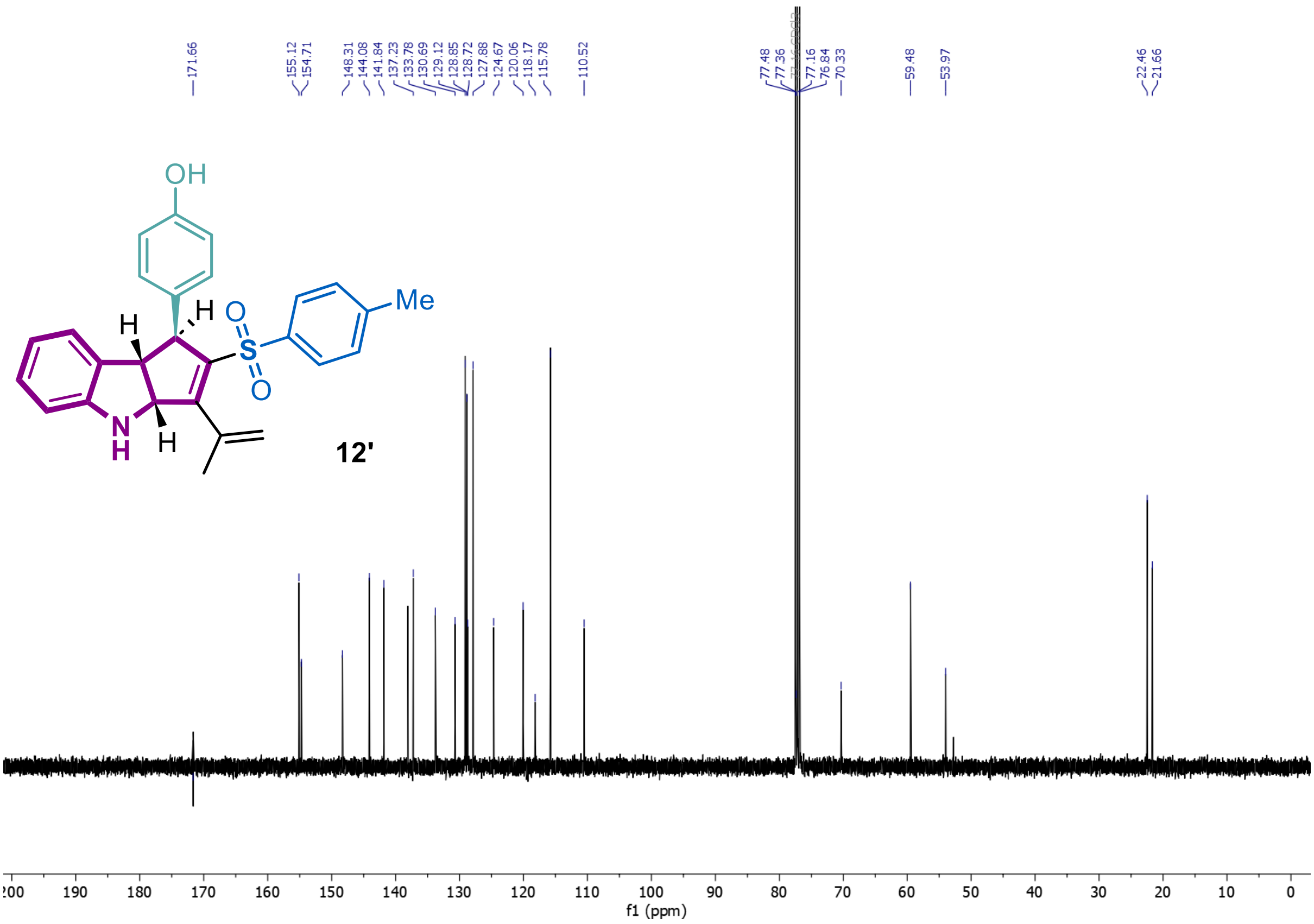

Figure S 169: *^{1}H-NMR of **12'** in $CDCl_3$ (400 MHz)*

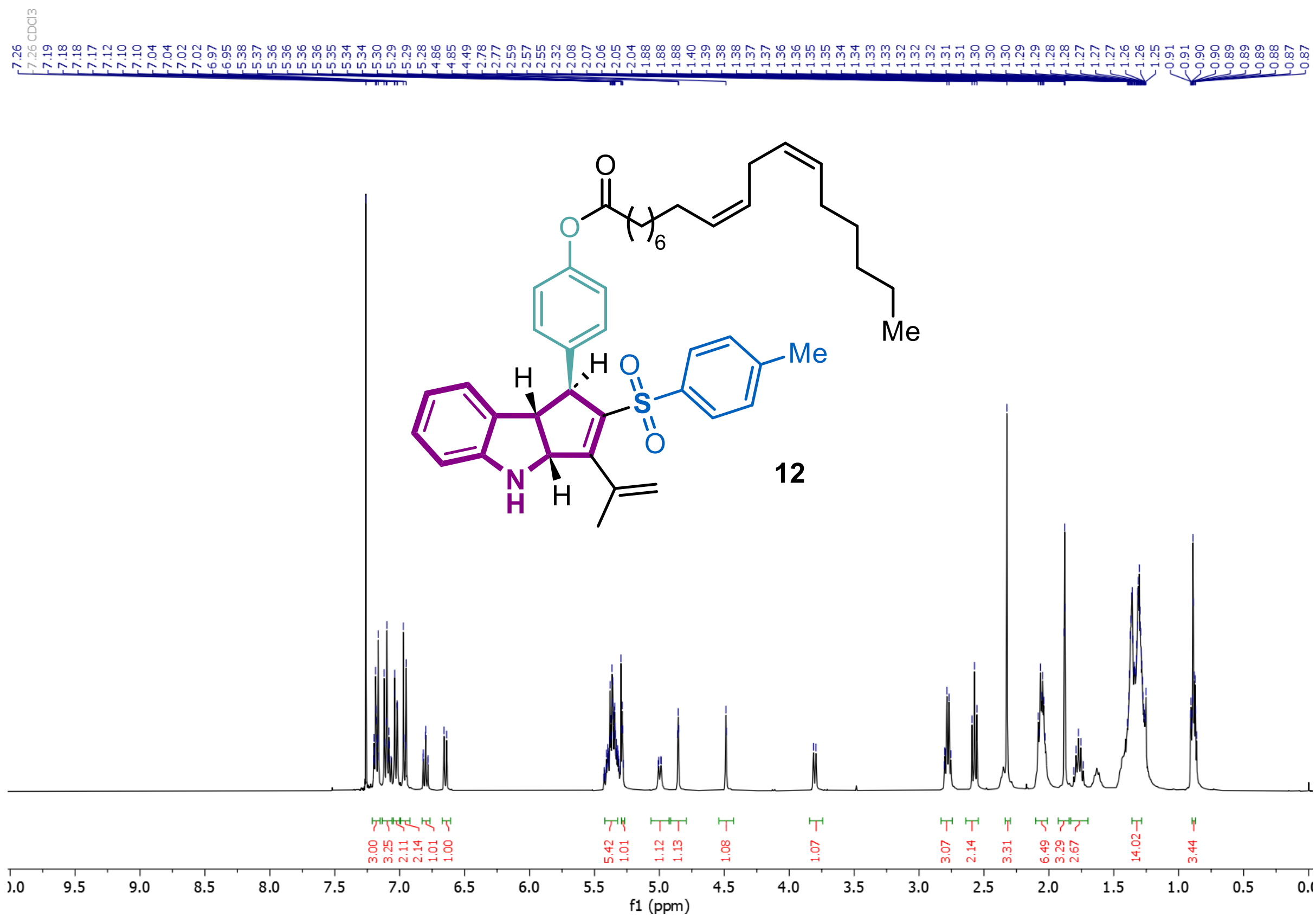

Figure S170: *^{1}H-NMR of **12** in CDCl₃ (400 MHz)*

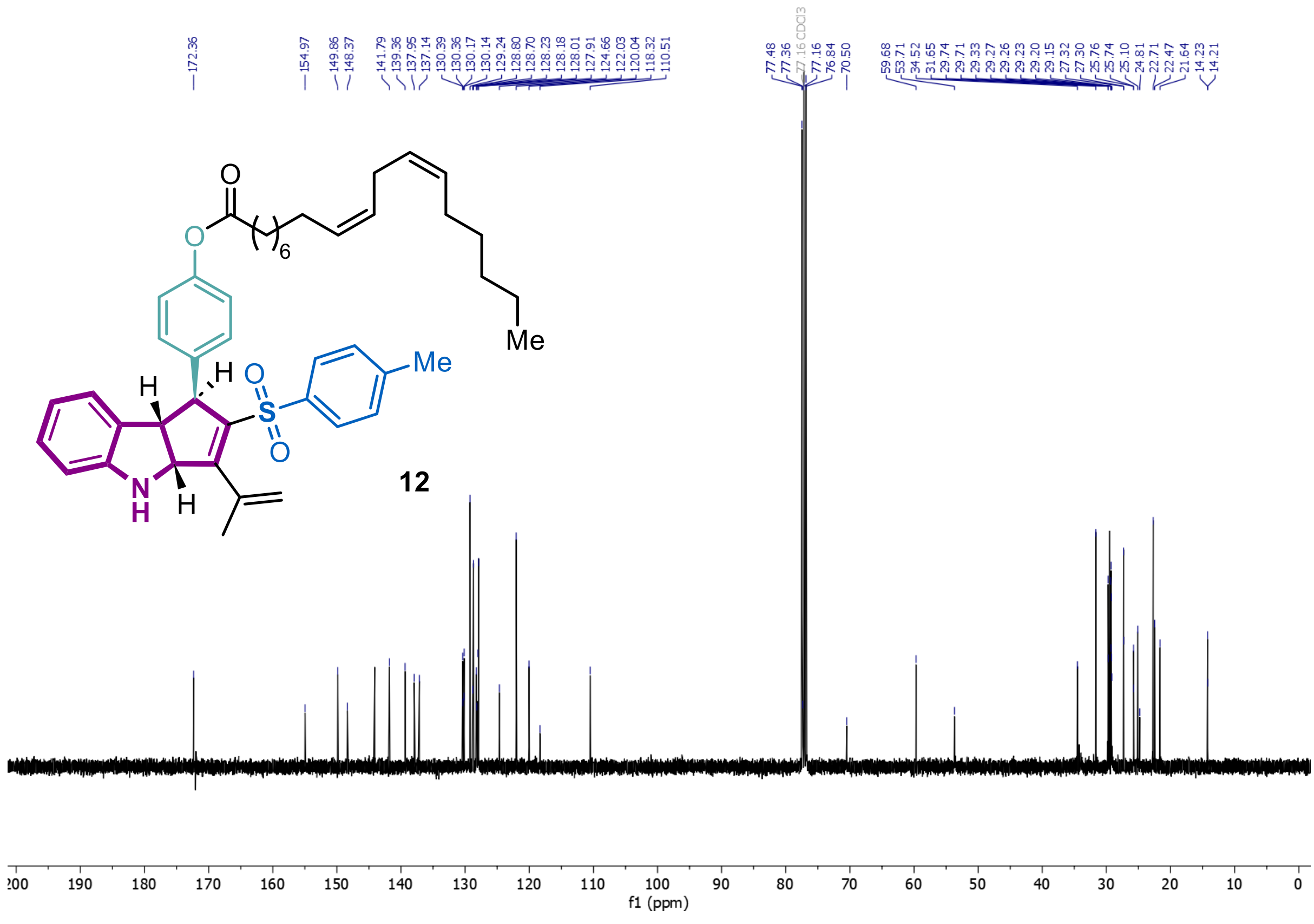

Figure S 171: : *^{13}C-NMR of **12** in $CDCl_3$ (101 MHz)*

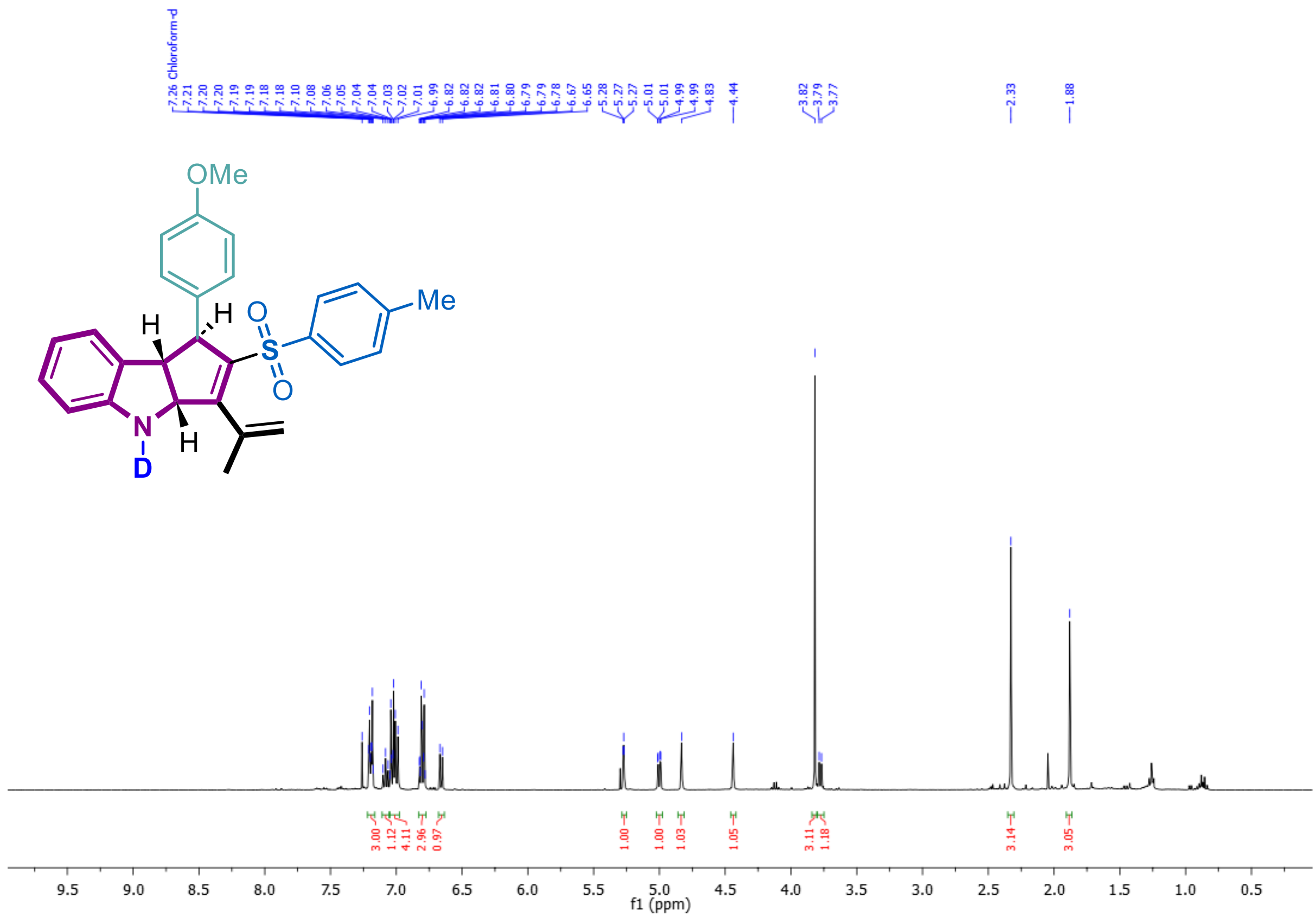

Figure S172: ^{1}H-NMR of **3a-N(D)** in $CDCl_3$ (400 MHz)

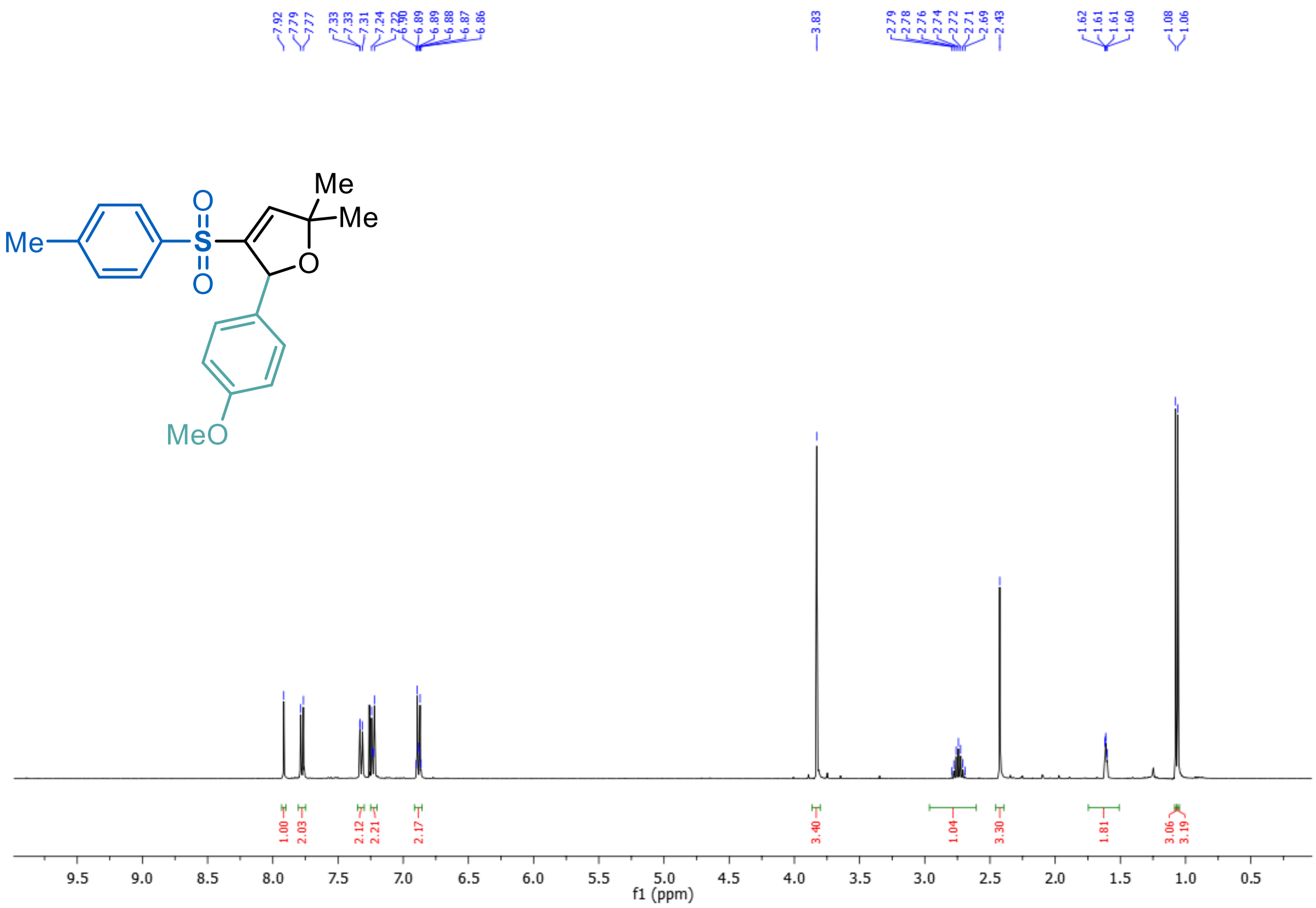

Figure S173: ^{1}H-NMR of **13** in $CDCl_3$ (400 MHz)

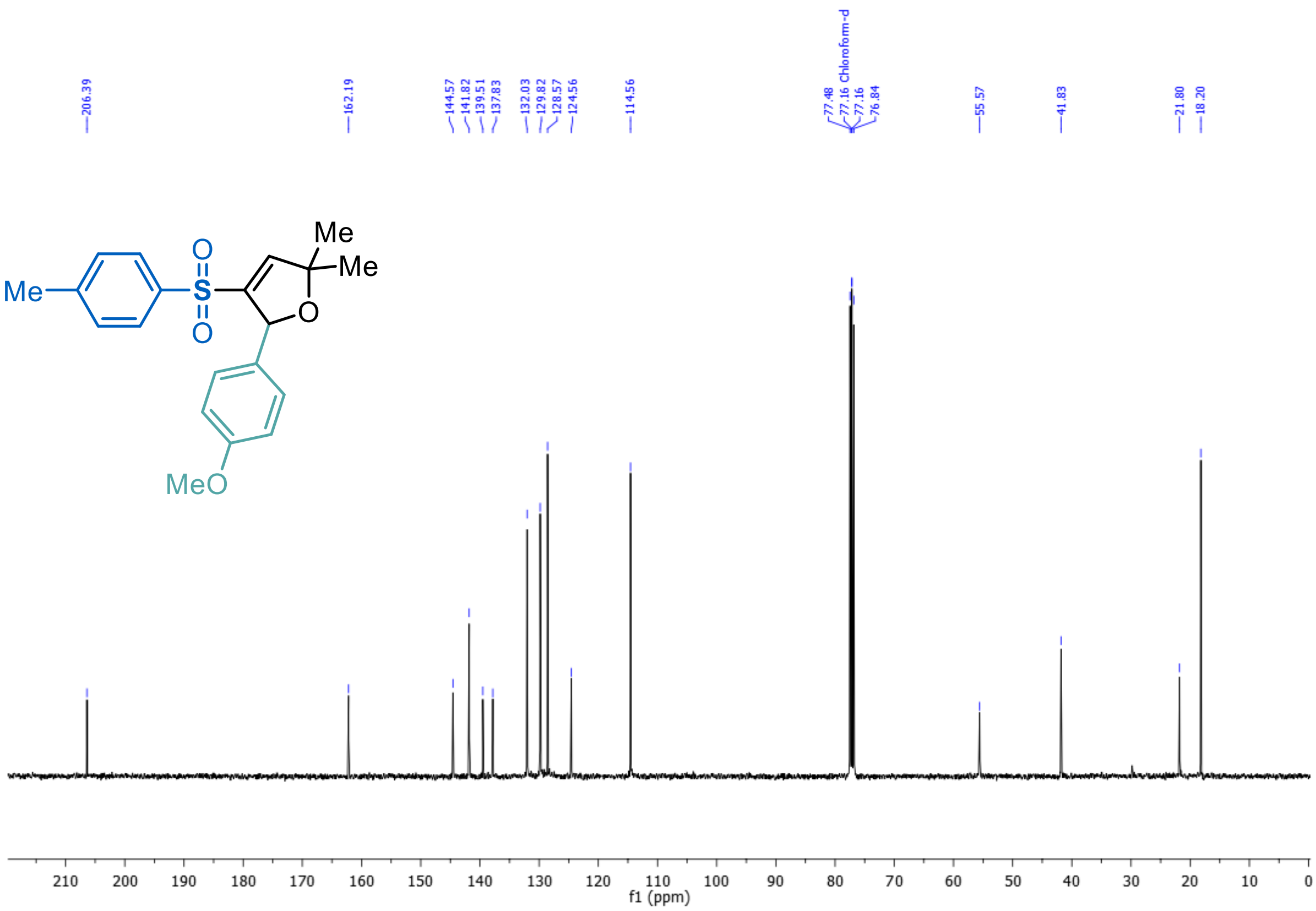

Figure S174: *^{13}C-NMR of **13** in $CDCl_3$ (101 MHz)*

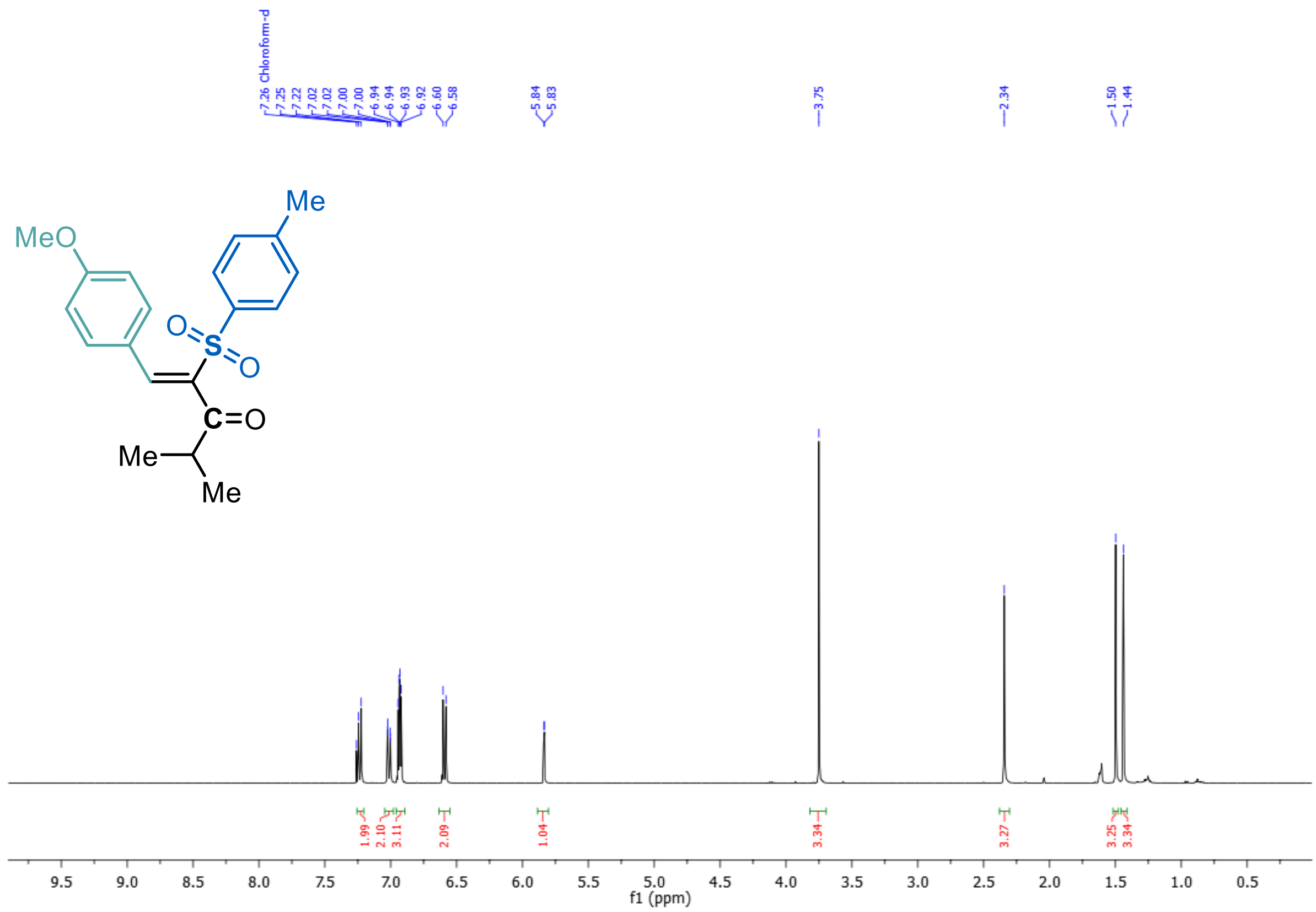

Figure S175: *^{1}H-NMR of **14** in $CDCl_3$ (400 MHz)*

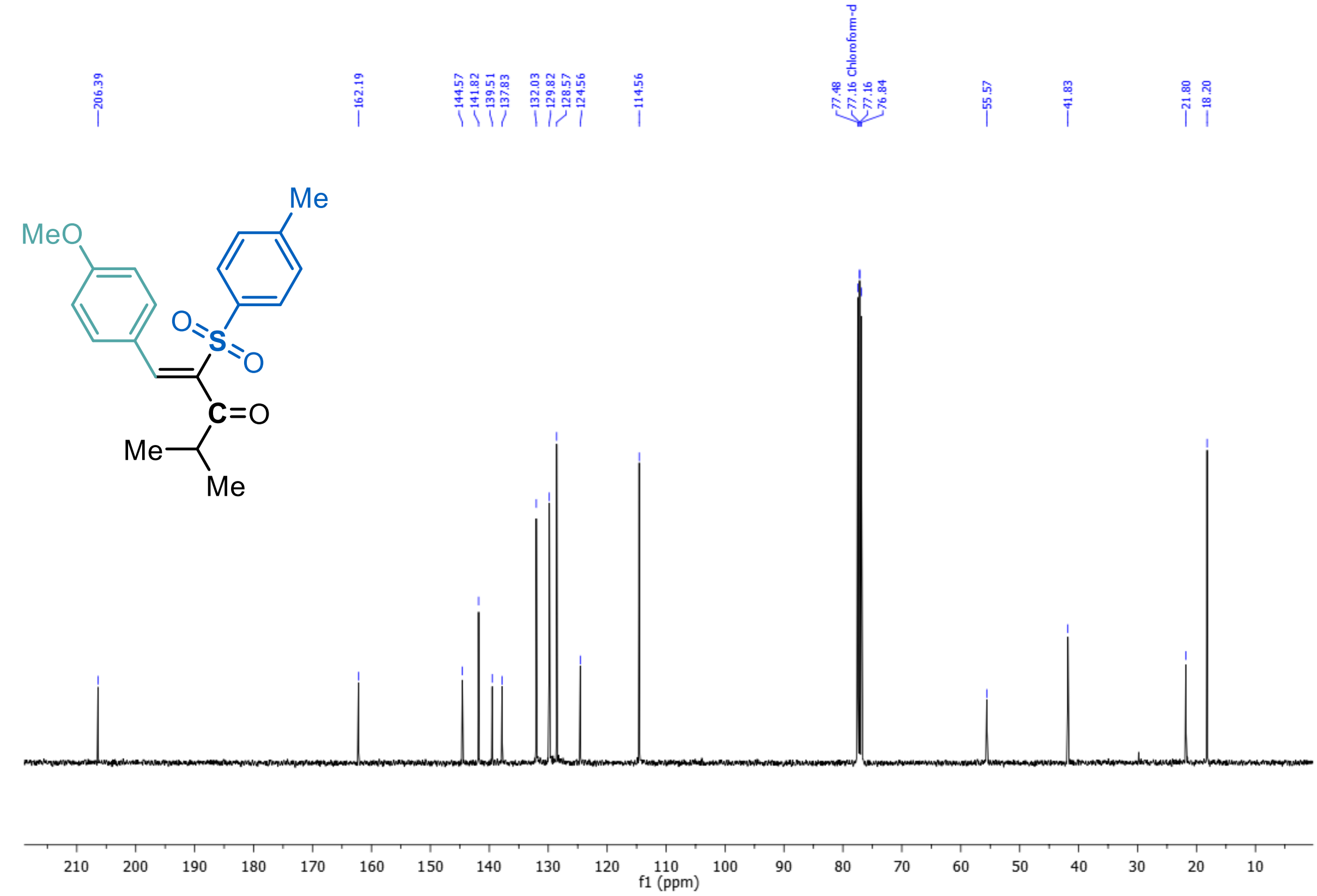

Figure S176: *^{13}C-NMR of **14** in $CDCl_3$ (101 MHz)*

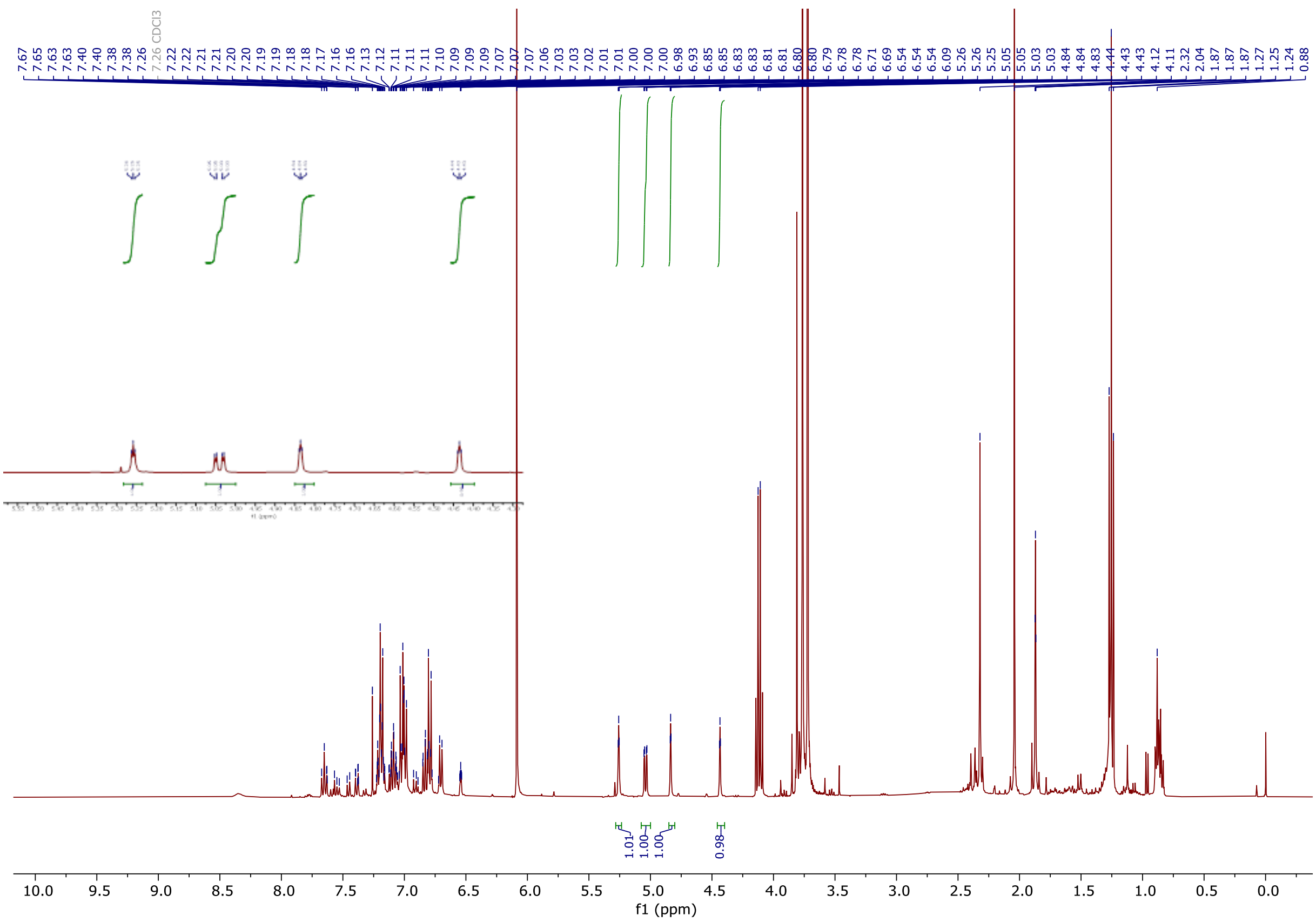

Figure S177: *Crude ^{1}H- NMR for model substrate **3a** showing single diastereoisomer formation.*
